# Supplementary material for: Nickel-catalysed selective migratory hydrothiolation of alkenes and alkynes with thiols
Source: Nat Commun. 2019 Apr 15;10:1752. doi: 10.1038/s41467-019-09783-w (PMC6465347; doi:10.1038/s41467-019-09783-w)
Supplement: Supplementary file 1 — Supplementary Information [file 41467_2019_9783_MOESM1_ESM.pdf]

Supplementary Information for

Nickel-Catalysed Selective Migratory Hydrothiolation of Alkenes and Alkynes with Thiols

Zhang et al.

Supplementary Methods

General reagent information. All solvents were purified and dried by passage through alumina and Q5 reactant-packed columns on a solvent purification system. HBpin (CAS 25015-63-8) was purchased from Energy Chemical and stored under nitrogen at –20 °C in glove box. NiI_2 (CAS 13462-90-3, Sigma-Aldrich), bathocuproine (CAS 4733-39-5, Energy Chemical) and Other commercial reagents were purchased from Sigma-Aldrich, Alfa Aesar, Acros, TCI, J&K, Energy Chemical, and were used as received. Flash chromatography was performed using glass columns with silica gel (Huanghai, 300-400 mesh).

General analytical information. All unknown compounds (starting materials and products) were characterized by ^{1}H NMR, ^{13}C NMR, IR spectroscopy, melting point (where applicable), and high-resolution mass spectrometry. ^{1}H NMR spectra were recorded on Bruker 400 MHz or 500 MHz spectrometer and are referenced relative to residual $CDCl_3$ proton signals at δ 7.26 ppm. ^{19}F NMR spectra were recorded on a Bruker 400 MHz or 500 MHz spectrometer and are referenced to $CFCl_3$ (δ 0.0 ppm). Data for ^{1}H and ^{19}F NMR are reported as follows: chemical shift (δ ppm), multiplicity (s = singlet, d = doublet, t = triplet, q = quartet, m = multiplet, ap = apparent), integration, and coupling constant (Hz). ^{13}C NMR spectra were recorded on a Bruker 400 MHz or 500 MHz spectrometer and are referenced to $CDCl_3$ at δ 77.16 ppm. The ^{13}C NMR spectra were obtained with ^{1}H decoupling. Data for ^{13}C NMR are reported in terms of chemical shift and multiplicity where appropriate. IR spectra were obtained on a Bruker Alpha and reported in terms of frequency of absorption (cm^{-1}). GC analyses were performed on an Agilent 7890B gas chromatograph or Shimadazu Nexis GC-2030 with an FID detector using a J & W DB-1 column (10 m, 0.1 mm I.D.). High Resolution Mass spectra were obtained on an Agilent 6540 Series Q-TOF system equipped with ESI. Reactions were monitored by GC analysis and thin-layer chromatography (TLC) was carried out on 0.20 mm Huanghai silica gel plates (HSGF254) using UV light as a visualizing agent. Organic solutions were concentrated using a Heidolph rotary evaporator.

Medium-sized screw-cap test tubes (8 mL) were used for all 0.20 mmol scale reactions: Fisher 13×100 mm tube (Cat. No. 14-959-35C)

Cap with Septa: Thermo Scientific ASM PHN CAP w/PTFE/SIL (Cat. No. 03378316)

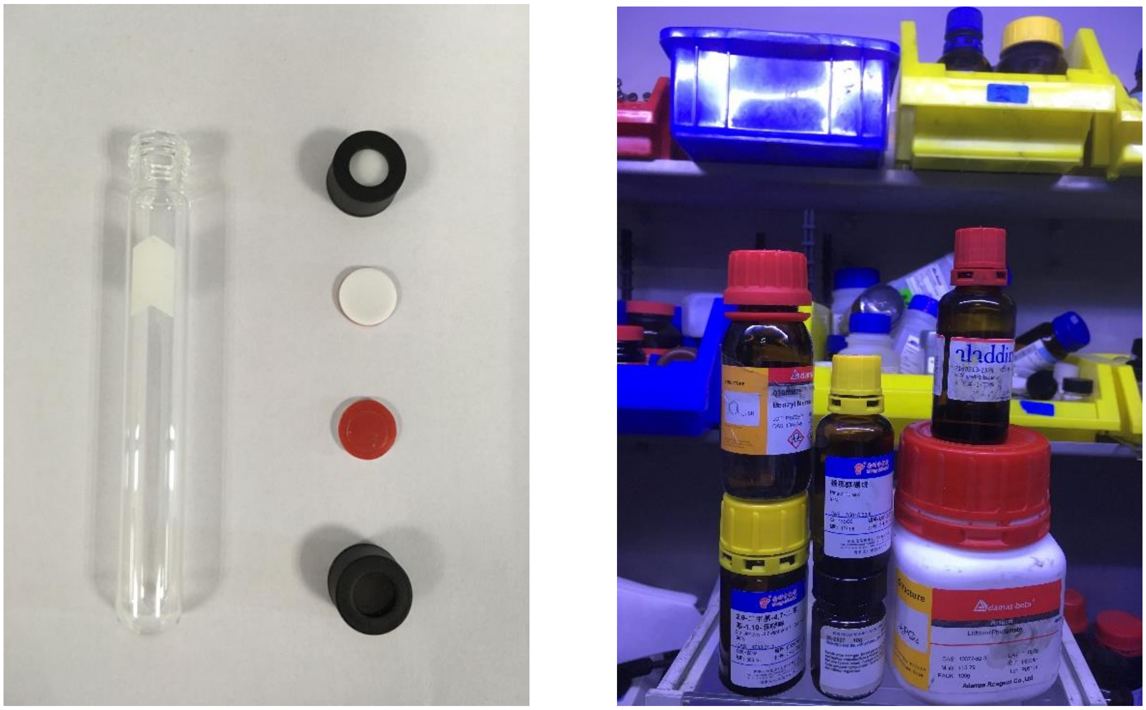

Supplementary Figure 1. Screw-cap vial, cap with septa, and raw materials to be used.

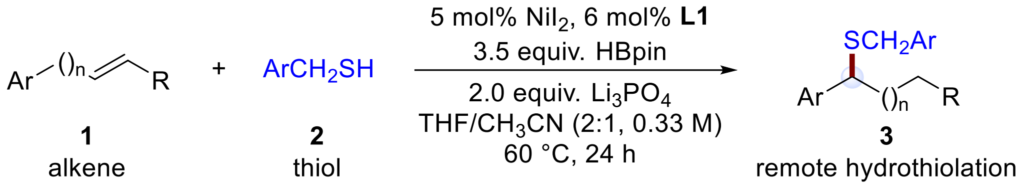

(1)

General procedure (A) for the NiH-catalysed remote hydrothiolation (Related to Figure 3)**.** To an oven-dried 8 mL screw-cap vial equipped with a magnetic stir bar was added NiI_2 (5.0 mol%) and bathocuproine (**L1**, 2,9-dimethyl-4,7-diphenyl-1,10-phenanthroline) (6.0 mol%). The vial was introduced into a nitrogen-filled glove box, anhydrous THF (0.40 mL) and CH_3CN (0.20 mL) were added, and the mixture was stirred for 10 min, at which time alkene (0.40 mmol, 2.0 equiv.), benzyl mercaptan (25.0 mg, 0.20 mmol, 1.0 equiv.), HBpin (pinacolborane, 100 µL, 0.70 mmol, 3.5 equiv.) and Li_3PO_4 (50 mg, 0.40 mmol, 2.0 equiv.) were added to the resulting mixture in this order. The tube was sealed with a teflon-lined screw cap, removed from the glove box and stirred at 60 ^{o}C for 24 h (the mixture was stirred at 750 rpm). After the reaction was complete, the reaction mixture was directly filtered through a short pad of silica gel (using ethyl acetate in hexanes) to give the crude product. *n*-Tetradecane (20 µL) was added as an internal standard for GC analysis. 1,1,2,2-Tetrachloroethane (10.5 µL, 0.10 mmol) was added as internal standard for ^{1}H NMR analysis of the crude material. The product was purified by chromatography on silica gel for each substrate. The yields reported are the average of at least two experiments, unless otherwise indicated.

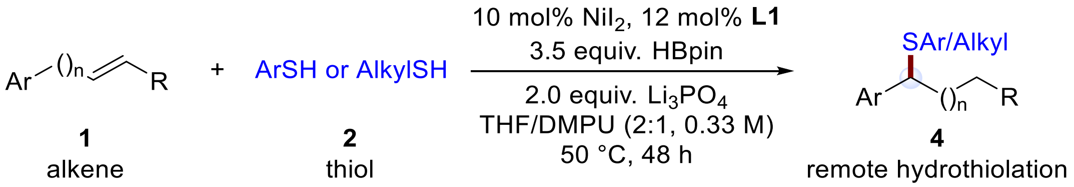

(2)

General procedure (B) for the NiH-catalysed remote hydrothiolation (Related to Figure 4)**.** To an oven-dried 8 mL screw-cap vial equipped with a magnetic stir bar was

added NiI_2 (10 mol%) and bathocuproine (**L1**, 2,9-dimethyl-4,7-diphenyl-1,10-phenanthroline) (12 mol%). The vial was introduced into a nitrogen-filled glove box, anhydrous THF (0.40 mL) and DMPU (0.20 mL) were added, and the mixture was stirred for 10 min, at which time 4-phenyl-1-butene (52.8 mg, 0.40 mmol, 2.0 equiv.), alkyl mercaptan or benzene thiol (0.20 mmol, 1.0 equiv.), HBpin (pinacolborane, 100 μL, 0.70 mmol, 3.5 equiv.) and Li_3PO_4 (50 mg, 0.40 mmol, 2.0 equiv.) were added to the resulting mixture in this order. The tube was sealed with a teflon-lined screw cap, removed from the glove box and stirred at 50 °C for 48 h (the mixture was stirred at 750 rpm). After the reaction was complete, the reaction mixture was directly filtered through a short pad of silica gel (using ethyl acetate in hexanes) to give the crude product. *n*-Tetradecane (20 μL) was added as an internal standard for GC analysis. 1,1,2,2-Tetrachloroethane (10.5 μL, 0.10 mmol) was added as internal standard for ^{1}H NMR analysis of the crude material. The product was purified by chromatography on silica gel for each substrate. The yields reported are the average of at least two experiments, unless otherwise indicated.

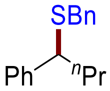

3a

benzyl(1-phenylbutyl)sulfane (Figure 3, **3a**): From 4-phenyl-1-butene (**1a**) (52.8 mg, 0.40 mmol, 2.0 equiv.), the title compound was prepared following the general procedure **A** using NiI_2 (3.2 mg, 0.010 mmol, 5.0 mol%), **L1** (4.0 mg, 0.012 mmol, 6.0 mol%), benzyl mercaptan (**2a**) (25.0 mg, 0.20 mmol, 1.0 equiv.), HBpin (100 μL, 0.70 mmol, 3.5 equiv.), Li_3PO_4 (50.0 mg, 0.40 mmol, 2.0 equiv.) and THF (0.4 mL)/CH_3CN (0.2 mL). The reaction mixture was stirred at 60 °C for 24 h. The crude material was purified by flash column chromatography (using 1–2% ethyl acetate in hexanes) to provide the title compound **3a** as a colorless liquid in 75% yield (38.4 mg), >99:1 r.r.. IR (neat, cm^{-1}): 2957, 2929, 1492, 1452, 1069, 1029, 767, 748, 696; ^{1}H NMR (400 MHz, $CDCl_3$): δ 7.26 – 7.16 (m, 6H), 7.14 – 7.11 (m, 4H), 3.55 (dd, *J* = 8.4, 6.4 Hz, 1H), 3.42 (d, *J* = 13.6 Hz, 1H), 3.31 (d, *J* = 13.2 Hz, 1H), 1.77 – 1.67 (m, 2H), 1.22 – 1.12 (m, 2H), 0.73 (t, *J* = 7.2 Hz, 3H); ^{13}C NMR (101 MHz, $CDCl_3$): δ 142.9, 138.6, 129.0, 128.5, 128.5, 128.2, 127.1,

126.9, 49.1, 38.7, 35.5, 20.9, 13.9; HRMS (ESI) calcd. for $C_{17}H_{20}SNa$ $[M+Na]^+$ *m/z* 279.1178, found 279.1183.

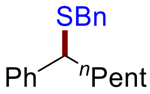

3b

benzyl(1-phenylhexyl)sulfane (Figure 3, **3b**): From hex-5-en-1-ylbenzene (**1b**) (64.0 mg, 0.40 mmol, 2.0 equiv.), the title compound was prepared following the general procedure **A** using NiI_2 (3.2 mg, 0.010 mmol, 5.0 mol%), **L1** (4.0 mg, 0.012 mmol, 6.0 mol%), benzyl mercaptan (**2a**) (25.0 mg, 0.20 mmol, 1.0 equiv.), HBpin (100 μL, 0.70 mmol, 3.5 equiv.), Li_3PO_4 (50.0 mg, 0.40 mmol, 2.0 equiv.) and THF (0.4 mL)/CH_3CN (0.2 mL). The reaction mixture was stirred at 60 °C for 24 h. The crude material was purified by flash column chromatography (using 1–2% ethyl acetate in hexanes) to provide the title compound **3b** as a colorless liquid in 63% yield (35.8 mg), >99:1 r.r.. IR (neat, cm^{-1}): 2955, 2927, 2856, 1493, 1452, 1070, 1028, 758, 695, 540; ^1H NMR (400 MHz, $CDCl_3$): δ 7.27 – 7.17 (m, 7H), 7.16 – 7.12 (m, 3H), 3.54 (dd, *J* = 8.4, 6.4 Hz, 1H), 3.42 (d, *J* = 13.6 Hz, 1H), 3.32 (d, *J* = 13.6 Hz, 1H), 1.80 – 1.65 (m, 2H), 1.22 – 1.06 (m, 6H), 0.74 (t, *J* = 6.8 Hz, 3H); ^{13}C NMR (101 MHz, $CDCl_3$): δ 142.9, 138.7, 129.1, 128.5, 128.5, 128.2, 127.1, 126.9, 49.4, 36.6, 35.6, 31.6, 27.4, 22.6, 14.1; HRMS (ESI) calcd. for $C_{19}H_{25}S$ $[M+H]^+$ *m/z* 285.1671, found 285.1665.

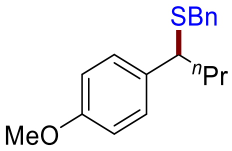

3c

benzyl(1-(4-methoxyphenyl)butyl)sulfane (Figure 3, **3c**): From 1-(but-3-en-1-yl)-4-methoxybenzene (**1c**) (64.8 mg, 0.40 mmol, 2.0 equiv.), the title compound was prepared following the general procedure **A** using NiI_2 (3.2 mg, 0.010 mmol, 5.0 mol%), **L1** (4.0 mg, 0.012 mmol, 6.0 mol%), benzyl mercaptan (**2a**) (25.0 mg, 0.20 mmol, 1.0 equiv.), HBpin (100 μL, 0.70 mmol, 3.5 equiv.), Li_3PO_4 (50 mg, 0.40 mmol, 2.0 equiv.) and THF (0.40 mL)/CH_3CN (0.20 mL). The reaction mixture was stirred at 60 °C for 24 h. The

crude material was purified by flash column chromatography (using 1–2% ethyl acetate in hexanes) to provide the title compound **3c** as a colorless liquid in 76% yield (43.5 mg), >99:1 r.r.. IR (neat, cm^{-1}): 2955, 2931, 1609, 1509, 1246, 1173, 1034, 831, 765, 696, 542; ^{1}H NMR (400 MHz, $CDCl_3$): δ 7.20 – 7.10 (m, 7H), 6.79 – 6.76 (m, 2H), 3.71 (s, 3H), 3.51 (dd, J = 8.8, 6.4 Hz, 1H), 3.40 (d, J = 13.6 Hz, 1H), 3.30 (d, J = 13.2 Hz, 1H), 1.74 – 1.62 (m, 2H), 1.19 – 1.12 (m, 2H), 0.72 (t, J = 7.4 Hz, 3H); ^{13}C NMR (101 MHz, $CDCl_3$): δ 158.6, 138.7, 134.7, 129.2, 129.0, 128.4, 126.8, 113.8, 55.3, 48.4, 38.8, 35.4, 20.9, 13.9; HRMS (ESI) calcd. for $C_{18}H_{23}OS$ $[M+H]^+$ *m/z* 287.1464, found 287.1460.

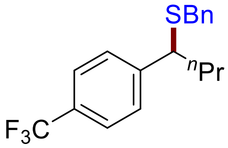

3d

benzyl(1-(4-(trifluoromethyl)phenyl)butyl)sulfane (Figure 3, **3d**): From 1-(but-3-en-1-yl)-4-(trifluoromethyl)benzene (**1d**) (80.0 mg, 0.40 mmol, 2.0 equiv.), the title compound was prepared following the general procedure **A** using NiI_2 (3.2 mg, 0.010 mmol, 5.0 mol%), **L1** (4.0 mg, 0.012 mmol, 6.0 mol%), benzyl mercaptan (**2a**) (25.0 mg, 0.20 mmol, 1.0 equiv.), HBpin (100 μL, 0.70 mmol, 3.5 equiv.), Li_3PO_4 (50 mg, 0.40 mmol, 2.0 equiv.) and THF (0.40 mL)/CH_3CN (0.20 mL). The reaction mixture was stirred at 60 ^{o}C for 24 h. The crude material was purified by flash column chromatography (using 1–2% ethyl acetate in hexanes) to provide the title compound **3d** as a colorless liquid in 90% yield (58.3), >99:1 r.r.. IR (neat, cm^{-1}): 2960, 2934, 1418, 1325, 1164, 1125, 1068, 1018, 846, 701; ^{1}H NMR (400 MHz, $CDCl_3$): δ 7.49 – 7.47 (m, 2H), 7.30 – 7.28 (m, 2H), 7.19 – 7.09 (m, 6H), 3.57 (dd, J = 8.4, 6.8 Hz, 1H), 3.43 (d, J = 13.6 Hz, 1H), 3.30 (d, J = 13.6 Hz, 1H), 1.77 – 1.63 (m, 2H), 1.23 – 1.07 (m, 2H), 0.73 (t, J = 7.4 Hz, 3H); ^{13}C NMR (101 MHz, $CDCl_3$): δ 147.3, 138.1, 129.3 (q, J_{C-F} = 32.7 Hz), 129.0, 128.6, 128.5, 127.1, 125.5 (q, J_{C-F} = 3.7 Hz), 124.3(q, J_{C-F} = 272.7 Hz), 48.6, 38.6, 35.6, 20.8, 13.8; ^{19}F NMR (471 MHz, $CDCl_3$): δ -62.3; HRMS (ESI) calcd. for $C_{18}H_{20}F_3S$ $[M+H]^+$ *m/z* 325.1232, found 325.1236.

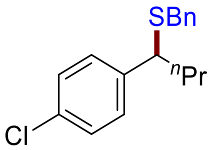

3e

benzyl(1-(4-chlorophenyl)butyl)sulfane (Figure 3, **3e**): From 1-(but-3-en-1-yl)-4-chlorobenzene (**1e**) (66.4 mg, 0.40 mmol, 2.0 equiv.), the title compound was prepared following the general procedure **A** using NiI_2 (3.2 mg, 0.010 mmol, 5.0 mol%), **L1** (4.0 mg, 0.012 mmol, 6.0 mol%), benzyl mercaptan (**2a**) (25.0 mg, 0.20 mmol, 1.0 equiv.), HBpin (100 µL, 0.70 mmol, 3.5 equiv.), Li_3PO_4 (50 mg, 0.40 mmol, 2.0 equiv.) and THF (0.40 mL)/CH_3CN (0.20 mL). The reaction mixture was stirred at 60 ^oC for 24 h. The crude material was purified by flash column chromatography (using 1–2% ethyl acetate in hexanes) to provide the title compound **3e** as a colorless liquid in 88% yield (51.0 mg), >99:1 r.r.. IR (neat, cm^{-1}): 2960, 2932, 1594, 1572, 1454, 1429, 1086, 904, 726, 694, 649; ^1H NMR (500 MHz, $CDCl_3$): δ 7.34 – 7.30 (m, 3H), 7.29 – 7.23 (m, 5H), 7.20 – 7.18 (m, 1H), 3.61 (dd, *J* = 8.5, 6.5 Hz, 1H), 3.56 (d, *J* = 13.5 Hz, 1H), 3.44 (d, *J* = 13.5 Hz, 1H), 1.87 – 1.73 (m, 2H), 1.35 – 1.20 (m, 2H), 0.86 (t, *J* = 7.5 Hz, 3H); ^{13}C NMR (126 MHz, $CDCl_3$): δ 145.2, 138.2, 134.4, 129.7, 129.0, 128.5, 128.2, 127.3, 127.0, 126.4, 48.6, 38.6, 35.6, 20.8, 13.8; HRMS (ESI) calcd. for $C_{17}H_{20}ClS$ $[M+H]^+$ *m/z* 291.0969, found 291.0963.

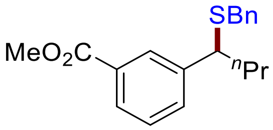

3f

methyl 3-(1-(benzylthio)butyl)benzoate (Figure 3, **3f**): From methyl 3-(but-3-en-1-yl)benzoate (**1f**) (76.0 mg, 0.40 mmol, 2.0 equiv.), the title compound was prepared following the general procedure **A** using NiI_2 (3.2 mg, 0.010 mmol, 5.0 mol%), **L1** (4.0 mg, 0.012 mmol, 6.0 mol%), benzyl mercaptan (**2a**) (25.0 mg, 0.20 mmol, 1.0 equiv.), HBpin (100 µL, 0.70 mmol, 3.5 equiv.), Li_3PO_4 (50 mg, 0.40 mmol, 2.0 equiv.) and THF (0.40 mL)/CH_3CN (0.20 mL). The reaction mixture was stirred at 60 ^oC for 24 h. The crude material was purified by flash column chromatography (using 1–2% ethyl acetate

in hexanes) to provide the title compound **3f** as a colorless liquid in 69% yield (43.3 mg), >99:1 r.r.. IR (neat, cm^{-1}): 2955, 2931, 1720, 1443, 1433, 1279, 1195, 1102, 981, 911, 749, 736, 693; ^1H NMR (400 MHz, $CDCl_3$): δ 7.85 – 7.83 (m, 2H), 7.41 – 7.40 (m, 1H), 7.33 – 7.29 (m, 1H), 7.20 – 7.17 (m, 2H), 7.14 – 7.09 (m, 3H), 3.84 (s, 3H), 3.59 (dd, *J* = 8.4, 6.8 Hz, 1H), 3.41 (d, *J* = 13.2 Hz, 1H), 3.29 (d, *J* = 13.2 Hz, 1H), 1.77 – 1.64 (m, 2H), 1.21 – 1.11 (m, 2H), 0.73 (t, *J* = 7.4 Hz, 3H); ^{13}C NMR (101 MHz, $CDCl_3$): δ 167.1, 143.5, 138.2, 132.7, 130.3, 129.3, 129.0, 128.7, 128.5, 128.4, 127.0, 52.2, 48.7, 38.5, 35.5, 20.8, 13.8; HRMS (ESI) calcd. for $C_{19}H_{22}O_2SNa$ $[M+Na]^+$ *m/z* 337.1233, found 337.1232.

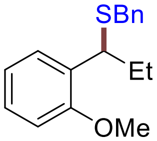

3g

benzyl(1-(2-methoxyphenyl)propyl)sulfane (Figure 3, **3g**): From 1-allyl-2-methoxybenzene (**1g**) (59.2 mg, 0.40 mmol, 2.0 equiv.), the title compound was prepared following the general procedure **A** using NiI_2 (3.2 mg, 0.010 mmol, 5.0 mol%), **L1** (4.0 mg, 0.012 mmol, 6.0 mol%), benzyl mercaptan (**2a**) (25.0 mg, 0.20 mmol, 1.0 equiv.), HBpin (100 μL, 0.70 mmol, 3.5 equiv.), Li_3PO_4 (50 mg, 0.40 mmol, 2.0 equiv.) and THF (0.40 mL)/CH_3CN (0.20 mL). The reaction mixture was stirred at 60 °C for 24 h. The crude material was purified by flash column chromatography (using 1–2% ethyl acetate in hexanes) to provide the title compound **3g** as a colorless liquid in 58% yield (31.6 mg), >99:1 r.r.. IR (neat, cm^{-1}): 3061, 2962, 1599, 1489, 1239, 1110, 1050, 1028, 751, 697, 473; ^1H NMR (400 MHz, $CDCl_3$): δ 7.40 – 7.38 (m, 1H), 7.24 – 7.16 (m, 2H), 7.13 – 7.10 (m, 4H), 6.92 – 6.88 (m, 1H), 6.79 – 6.76 (m, 1H), 4.17 (t, *J* = 7.6 Hz, 1H), 3.68 (s, 3H), 3.47 (d, *J* = 13.6 Hz, 1H), 3.43 (d, *J* = 13.6 Hz, 1H), 1.83 – 1.69 (m, 2H), 0.77 (t, *J* = 7.2 Hz, 3H); ^{13}C NMR (101 MHz, $CDCl_3$): δ 157.3, 138.9, 130.9, 129.0, 128.4, 128.3, 127.8, 126.8, 120.9, 110.6, 55.5, 43.2, 35.8, 29.0, 12.2; HRMS (ESI) calcd. for $C_{17}H_{21}OS$ $[M+H]^+$ *m/z* 273.1308, found 273.1305.

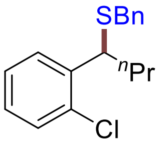

3h

benzyl(1-(2-chlorophenyl)butyl)sulfane (Figure 3, **3h**): From 1-(but-3-en-1-yl)-2-chlorobenzene (**1h**) (66.4 mg, 0.40 mmol, 2.0 equiv.), the title compound was prepared following the general procedure **A** using NiI_2 (3.2 mg, 0.010 mmol, 5.0 mol%), **L1** (4.0 mg, 0.012 mmol, 6.0 mol%), benzyl mercaptan (**2a**) (25.0 mg, 0.20 mmol, 1.0 equiv.), HBpin (100 µL, 0.70 mmol, 3.5 equiv.), Li_3PO_4 (50 mg, 0.40 mmol, 2.0 equiv.) and THF (0.40 mL)/CH_3CN (0.20 mL). The reaction mixture was stirred at 60 ^oC for 24 h. The crude material was purified by flash column chromatography (using 1–2% ethyl acetate in hexanes) to provide the title compound **3h** as a colorless liquid in 72% yield (41.8 mg), >99:1 r.r.. IR (neat, cm^{-1}): 3028, 2957, 2930, 1494, 1468, 1453, 1441, 1046, 1033, 750, 697; ^1H NMR (400 MHz, $CDCl_3$): δ 7.56 – 7.54 (m, 1H), 7.25 – 7.15 (m, 4H), 7.13 – 7.05 (m, 4H), 4.30 (t, J = 7.6 Hz, 1H), 3.44 (s, 2H), 1.75 – 1.62 (m, 2H), 1.32 – 1.12 (m, 2H), 0.75 (t, J = 7.2 Hz, 3H); ^{13}C NMR (101 MHz, $CDCl_3$): δ 140.5, 138.3, 134.0, 129.4, 129.3, 129.0, 128.5, 128.0, 127.4, 127.0, 44.8, 38.5, 35.8, 20.5, 13.9; HRMS (ESI) calcd. for $C_{17}H_{20}ClS$ $[M+H]^+$ m/z 291.0969, found 291.0970.

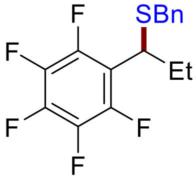

3i

benzyl(1-(perfluorophenyl)propyl)sulfane (Figure 3, **3i**): From 1-allyl-2,3,4,5,6-pentafluorobenzene (**1i**) (83.2 mg, 0.40 mmol, 2.0 equiv.), the title compound was prepared following the general procedure **A** using NiI_2 (3.2 mg, 0.010 mmol, 5.0 mol%), **L1** (4.0 mg, 0.012 mmol, 6.0 mol%), benzyl mercaptan (**2a**) (25.0 mg, 0.20 mmol, 1.0 equiv.), HBpin (100 µL, 0.70 mmol, 3.5 equiv.), Li_3PO_4 (50 mg, 0.40 mmol, 2.0 equiv.) and THF (0.40 mL)/CH_3CN (0.20 mL). The reaction mixture was stirred at 60 ^oC for 24 h. The crude material was purified by flash column chromatography (using 1–2% ethyl

acetate in hexanes) to provide the title compound **3i** as a colorless liquid in 85% yield (56.4 mg), >99:1 r.r.. IR (neat, cm^{-1}): 2972, 1520, 1499, 1417, 978, 951, 903, 723, 649; ^1H NMR (400 MHz, $CDCl_3$): δ 7.18 – 7.17 (m, 3H), 7.14 – 7.11 (m, 2H), 3.87 (dd, *J* = 8.4, 6.4 Hz, 1H), 3.64 (d, *J* = 14.8 Hz, 1H), 3.59 (d, *J* = 14.4 Hz, 1H), 1.92 – 1.85 (m, 2H), 0.79 (t, *J* = 7.4 Hz, 3H); ^{13}C NMR (101 MHz, $CDCl_3$): δ 146.2 (m) & 143.8 (m), 141.2 (m) & 138.8 (m), 137.6, 136.2 (m) & 130.1 (m), 128.8, 128.5, 127.3, 117.0 (m), 40.9, 37.2, 27.1, 12.7; ^{19}F NMR (471 MHz, $CDCl_3$): δ -141.0, -156.6, -162.4; HRMS (ESI) calcd. for $C_{16}H_{14}F_5S$ $[M+H]^+$ *m/z* 333.0731, found 333.0732.

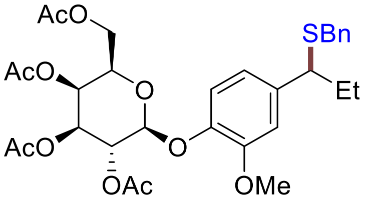

3j

(2R,3S,4S,5R,6S)-2-(acetoxymethyl)-6-(4-(1-(benzylthio)propyl)-2-methoxyphenoxy)tetrahydro-2*H*-pyran-3,4,5-triyl triacetate (Figure 3, **3j**): From (2R,3S,4S,5R,6S)-2-(acetoxymethyl)-6-(4-allyl-2-methoxyphenoxy)tetrahydro-2*H*-pyran-3,4,5-triyl triacetate (**1j**) (204.0 mg, 0.40 mmol, 2.0 equiv.), the title compound was prepared following the general procedure **A** using NiI_2 (3.2 mg, 0.010 mmol, 5.0 mol%), **L1** (4.0 mg, 0.012 mmol, 6.0 mol%), benzyl mercaptan (**2a**) (25.0 mg, 0.20 mmol, 1.0 equiv.), HBpin (100 μL, 0.70 mmol, 3.5 equiv.), Li_3PO_4 (50.0 mg, 0.40 mmol, 2.0 equiv.) and THF (0.4 mL)/CH_3CN (0.2 mL). The reaction mixture was stirred at 60 °C for 24 h. The crude material was purified by flash column chromatography (using 1–2% ethyl acetate in hexanes) to provide the title compound **3j** as a colorless liquid in 73% yield (92.6 mg), >99:1 r.r., 1:1 d.r.. IR (neat, cm^{-1}): 3055, 2254, 1750, 1372, 1227, 1076, 903, 722, 650; ^1H NMR (500 MHz, $CDCl_3$): δ 7.30 – 7.27 (m, 4H), 7.24 – 7.20 (m, 6H), 7.09 – 7.06 (m, 2H), 6.89 – 6.88 (m, 1H) & 6.83 – 6.82 (m, 1H), 6.79 – 6.77 (m, 1H) & 6.74 – 6.72 (m, 1H), 5.53 (d, *J* = 8.4 Hz, 1H) & 5.52 (d, *J* = 8.4 Hz, 1H), 5.46 – 5.45 (m, 2H), 5.14 – 5.13 (m, 1H) & 5.12 – 5.11 (m, 1H), 4.93 (d, *J* = 6.4 Hz, 1H) & 4.92 (d, *J* = 6.4 Hz, 1H), 4.26 (dd, *J* = 9.2, 5.2 Hz, 1H+1H), 4.19 – 4.15 (m, 2H) & 3.52 – 3.48 (m,

2H), 4.00 (dd, J = 9.6, 4.8 Hz, 1H+1H), 3.83 (s, 3H) & 3.82 (s, 3H), 3.54 (d, J = 10.8 Hz, 1H) & 3.44 (d, J = 11.2 Hz, 1H), 3.53 (d, J = 10.8 Hz, 1H) & 3.43 (d, J = 10.8 Hz, 1H), 2.19 (s, 3H+3H), 2.12 (s, 3H+3H), 2.05 (s, 3H+3H), 2.03 (s, 3H+3H), 1.90 – 1.84 (m, 1H+1H), 1.80 – 1.74 (m, 1H+1H), 0.85 (t, J = 5.8 Hz, 3H) & 0.84 (t, J = 5.8 Hz, 3H); ^{13}C NMR (126 MHz, $CDCl_3$): δ 170.4 & 170.4, 170.4, 170.3, 169.6, 150.7 & 150.6, 145.2 & 145.1, 139.1 & 139.1, 138.5 & 138.5, 129.0, 128.4, 126.9, 120.6 & 120.5, 119.7 & 119.6, 112.1 & 112.1, 101.5 & 101.4, 71.0, 70.8, 68.8, 67.0, 61.4 & 61.4, 56.1 & 56.1, 50.9 & 50.8, 35.5, 29.8, 20.9, 20.8 (2C), 20.7, 12.4 & 12.3; HRMS (ESI) calcd. for $C_{31}H_{38}NaO_{11}S$ $[M+Na]^+$ m/z 641.2027, found 641.2031.

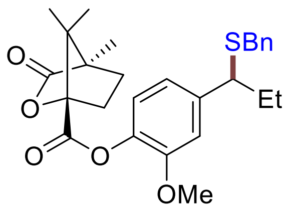

3k

4-(1-(benzylthio)propyl)-2-methoxyphenyl (1S,4R)-4,7,7-trimethyl-3-oxo-2-oxabicyclo[2.2.1]heptane-1-carboxylate (Figure 3, **3k**): From 4-allyl-2-methoxyphenyl (1S,4R)-7,7-dimethyl-3-oxobicyclo[2.2.1]heptane-1-carboxylate (**1k**) (137.7 mg, 0.40 mmol, 2.0 equiv.), the title compound was prepared following the general procedure **A** using NiI_2 (3.2 mg, 0.010 mmol, 5.0 mol%), **L1** (4.0 mg, 0.012 mmol, 6.0 mol%), benzyl mercaptan (**2a**) (25.0 mg, 0.20 mmol, 1.0 equiv.), HBpin (100 µL, 0.70 mmol, 3.5 equiv.), Li_3PO_4 (50 mg, 0.40 mmol, 2.0 equiv.) and THF (0.40 mL)/CH_3CN (0.20 mL). The reaction mixture was stirred at 60 °C for 24 h. The crude material was purified by flash column chromatography (using 1–2% ethyl acetate in hexanes) to provide the title compound **3k** as a colorless liquid in 75% yield (70.2 mg), >99:1 r.r.. IR (neat, cm^{-1}): 3028, 2254, 1783, 1510, 1268, 1169, 1121, 1098, 1049, 903, 722, 649; ^{1}H NMR (400 MHz, $CDCl_3$): δ 7.31 – 7.28 (m, 2H), 7.25 – 7.21 (m, 3H), 7.04 – 7.02 (m, 1H), 6.95 – 6.94 (m, 1H), 6.87 – 6.84 (m, 1H), 3.83 (s, 3H), 3.55 (d, J = 13.6 Hz, 1H), 3.54 (t, J = 7.6 Hz, 1H), 3.45 (d, J = 13.6 Hz, 1H), 2.66 – 2.59 (m, 1H), 2.26 – 2.19 (m, 1H), 2.06 – 1.98 (m, 1H), 1.92 – 1.85 (m, 1H), 1.83 – 1.75 (m, 2H), 1.20 (s, 3H), 1.19 (s, 3H), 1.17 (s, 3H),

0.88 (t, J = 7.2 Hz, 3H); ^{13}C NMR (101 MHz, $CDCl_3$): δ 178.1, 165.5, 150.7, 142.2, 138.4, 137.9, 129.0, 128.4, 126.9, 122.2, 120.7, 111.9, 91.2, 55.8, 55.0, 54.7, 51.0 & 50.9, 35.5, 30.9, 29.9, 29.1, 16.8, 16.7, 12.3, 9.9; HRMS (ESI) calcd. for $C_{27}H_{32}NaO_5S$ $[M+Na]^+$ *m/z* 491.1863, found 491.1867.

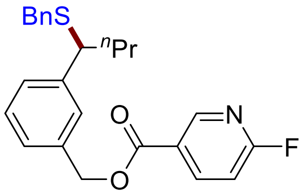

3l

3-(1-(benzylthio)butyl)benzyl 6-fluoronicotinate (Figure 3, **3l**): From 3-(but-3-en-1-yl)benzyl 6-fluoronicotinate (**1l**) (114.0 mg, 0.40 mmol, 2.0 equiv.), the title compound was prepared following the general procedure **A** using NiI_2 (3.2 mg, 0.010 mmol, 5.0 mol%), **L1** (4.0 mg, 0.012 mmol, 6.0 mol%), benzyl mercaptan (**2a**) (25.0 mg, 0.20 mmol, 1.0 equiv.), HBpin (100 µL, 0.70 mmol, 3.5 equiv.), Li_3PO_4 (50 mg, 0.40 mmol, 2.0 equiv.) and THF (0.40 mL)/CH_3CN (0.20 mL). The reaction mixture was stirred at 60 °C for 24 h. The crude material was purified by flash column chromatography (using 1–2% ethyl acetate in hexanes) to provide the title compound **3l** as a colorless liquid in 85% yield (69.5 mg), >99:1 r.r.. IR (neat, cm^{-1}): 2957, 2930, 1721, 1593, 1482, 1379, 1264, 1109, 1019, 775, 698; ^{1}H NMR (500 MHz, $CDCl_3$): δ 8.96 (d, J = 2.5 Hz, 1H), 8.47 – 8.44 (m, 1H), 7.40 – 7.27 (m, 5H), 7.24 – 7.20 (m, 4H), 7.04 – 7.02 (m, 1H), 5.41 (s, 2H), 3.67 (dd, J = 8.5, 6.5 Hz, 1H), 3.53 (d, J = 13.5 Hz, 1H), 3.42 (d, J = 13.5 Hz, 1H), 1.86 – 1.78 (m, 2H), 1.34 – 1.25 (m, 2H), 0.84 (t, J = 7.5 Hz, 3H); ^{13}C NMR (126 MHz, $CDCl_3$): δ 165.6 (d, $J_{C\text{-}F}$ = 282.8 Hz), 165.0, 150.6 (d, $J_{C\text{-}F}$ = 14.1 Hz), 143.6, 142.8 (d, $J_{C\text{-}F}$ = 7.1 Hz), 138.4, 135.5, 129.0, 128.9, 128.5, 128.4, 128.1, 127.1, 127.0, 124.5 (d, $J_{C\text{-}F}$ = 3.0 Hz), 109.7 (d, $J_{C\text{-}F}$ = 30.3 Hz), 67.4, 48.9, 38.6, 35.5, 20.9, 13.8; ^{19}F NMR (471 MHz, $CDCl_3$): δ -61.0; HRMS (ESI) calcd. for $C_{24}H_{25}FNO_2S$ $[M+H]^+$ *m/z* 410.1585, found 410.1583.

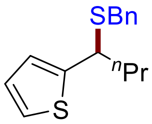

3m

2-(1-(benzylthio)butyl)thiophene (Figure 3, **3m**): From 2-(but-3-en-1-yl)thiophene (**1m**) (55.2 mg, 0.40 mmol, 2.0 equiv.), the title compound was prepared following the general procedure **A** using NiI_2 (3.2 mg, 0.010 mmol, 5.0 mol%), **L1** (4.0 mg, 0.012 mmol, 6.0 mol%), benzyl mercaptan (**2a**) (25.0 mg, 0.20 mmol, 1.0 equiv.), HBpin (100 μL, 0.70 mmol, 3.5 equiv.), Li_3PO_4 (50 mg, 0.40 mmol, 2.0 equiv.) and THF (0.40 mL)/CH_3CN (0.20 mL). The reaction mixture was stirred at 60 °C for 24 h. The crude material was purified by flash column chromatography (using 1–2% ethyl acetate in hexanes) to provide the title compound **3m** as a colorless liquid in 72% yield (37.7 mg), >99:1 r.r.. IR (neat, cm^{-1}): 2959, 2931, 1494, 1260, 1095, 1029, 904, 726, 697, 649; ^1H NMR (400 MHz, $CDCl_3$): δ 7.23 – 7.12 (m, 5H), 6.85 – 6.79 (m, 3H), 6.80 – 6.79 (m, 1H), 3.85 (dd, J = 8.4, 6.4 Hz, 1H), 3.53 (d, J = 13.2 Hz, 1H), 3.45 (d, J = 13.2 Hz, 1H), 1.79 – 1.72 (m, 2H), 1.29 – 1.21 (m, 2H), 0.76 (t, J = 7.4 Hz, 3H); ^{13}C NMR (101 MHz, $CDCl_3$): δ 148.0, 138.4, 129.1, 128.5, 127.0, 126.2, 125.5, 124.6, 44.2, 39.8, 35.7, 20.9, 13.7; HRMS (ESI) calcd. for $C_{15}H_{19}S_2$ $[M+H]^+$ m/z 263.0923, found 263.0920.

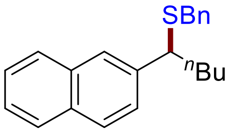

3n

benzyl(1-(naphthalen-2-yl)pentyl)sulfane (Figure 3, **3n**): From 2-(pent-3-en-1-yl)naphthalene (**1n**) (78.5 mg, 0.40 mmol, 2.0 equiv.), the title compound was prepared following the general procedure **A** using NiI_2 (3.2 mg, 0.010 mmol, 5.0 mol%), **L1** (4.0 mg, 0.012 mmol, 6.0 mol%), benzyl mercaptan (**2a**) (25.0 mg, 0.20 mmol, 1.0 equiv.), HBpin (100 μL, 0.70 mmol, 3.5 equiv.), Li_3PO_4 (50 mg, 0.40 mmol, 2.0 equiv.) and THF (0.40 mL)/CH_3CN (0.20 mL). The reaction mixture was stirred at 60 °C for 24 h. The crude material was purified by flash column chromatography (using 1–2% ethyl acetate in hexanes) to provide the title compound **3n** as a colorless liquid in 85% yield (54.4 mg), and >99:1 r.r.. IR (neat, cm^{-1}): 3059, 1599, 1494, 1453, 1382, 903, 820, 723, 649, 479; ^1H

NMR (500 MHz, $CDCl_3$): δ 7.89 – 7.84 (m, 3H), 7.56 – 7.51 (m, 3H), 7.36 – 7.23 (m, 6H), 3.82 (dd, *J* = 8.5, 6.5 Hz, 1H), 3.52 (d, *J* = 13.5 Hz, 1H), 3.42 (d, *J* = 13.5 Hz, 1H), 1.98 – 1.90 (m, 2H), 1.34 – 1.19 (m, 4H), 0.84 (t, *J* = 6.8 Hz, 3H); ^{13}C NMR (126 MHz, $CDCl_3$): δ 140.0, 138.5, 133.2, 132.9, 129.5, 129.0, 128.6, 128.6, 128.5, 127.8, 127.1, 126.9, 126.2, 125.8, 49.6, 36.0, 35.5, 29.9, 22.5, 14.0; HRMS (ESI) calcd. for $C_{22}H_{25}S$ $[M+H]^+$ *m/z* 321.1671, found 321.1675.

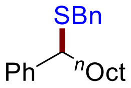

3o

benzyl(1-phenylpentyl)sulfane (Figure 3, **3o**): From non-3-en-1-ylbenzene (**1o**) (80.9 mg, 0.40 mmol, 2.0 equiv.), the title compound was prepared following the general procedure **A** using NiI_2 (3.2 mg, 0.010 mmol, 5.0 mol%), **L1** (4.0 mg, 0.012 mmol, 6.0 mol%), benzyl mercaptan (**2a**) (25.0 mg, 0.20 mmol, 1.0 equiv.), HBpin (100 µL, 0.70 mmol, 3.5 equiv.), Li_3PO_4 (50 mg, 0.40 mmol, 2.0 equiv.) and THF (0.40 mL)/CH_3CN (0.20 mL). The reaction mixture was stirred at 60 ^{o}C for 24 h. The crude material was purified by flash column chromatography (using 1–2% ethyl acetate in hexanes) to provide the title compound **3o** as a colorless liquid in 78% yield (42.1 mg), >99:1 r.r.. IR (neat, cm^{-1}): 2928, 2856, 1493, 1453, 1379, 904, 725, 699, 649; ^{1}H NMR (400 MHz, $CDCl_3$): δ 7.26 – 7.15 (m, 6H), 7.13 – 7.10 (m, 4H), 3.52 (dd, *J* = 8.4, 6.8 Hz, 1H), 3.41 (d, *J* = 13.6 Hz, 1H), 3.30 (d, *J* = 13.2 Hz, 1H), 1.78 – 1.66 (m, 2H), 1.18 – 1.10 (m, 12H), 0.77 (t, *J* = 7.0 Hz, 3H); ^{13}C NMR (101 MHz, $CDCl_3$): δ 142.9, 138.6, 129.0, 128.5, 128.4, 128.2, 127.1, 126.9, 49.3, 36.6, 35.5, 31.9, 29.5, 29.4, 29.3, 27.7, 22.8, 14.2; HRMS (ESI) calcd. for $C_{22}H_{31}S$ $[M+H]^+$ *m/z* 327.2141, found 327.2143.

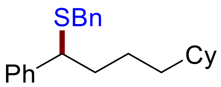

3p

benzyl(4-cyclohexyl-1-phenylbutyl)sulfane (Figure 3, **3p**): From (4-cyclohexylbut-3-en-1-yl)benzene (**1p**) (85.7 mg, 0.40 mmol, 2.0 equiv.), the title compound was prepared following the general procedure **A** using NiI_2 (3.2 mg, 0.010 mmol, 5.0 mol%), **L1** (4.0

mg, 0.012 mmol, 6.0 mol%), benzyl mercaptan (**2a**) (25.0 mg, 0.20 mmol, 1.0 equiv.), HBpin (100 µL, 0.70 mmol, 3.5 equiv.), Li_3PO_4 (50 mg, 0.40 mmol, 2.0 equiv.) and THF (0.40 mL)/CH_3CN (0.20 mL). The reaction mixture was stirred at 60 °C for 48 h. The crude material was purified by flash column chromatography (using 1–2% ethyl acetate in hexanes) to provide the title compound **3p** as a colorless liquid in 73% yield (49.3 mg), >99:1 r.r.. IR (neat, cm^{-1}): 2919, 2848, 1492, 1450, 1071, 1029, 764, 738, 695, 530; ^1H NMR (500 MHz, $CDCl_3$): δ 7.38 – 7.35 (m, 2H), 7.33 – 7.28 (m, 5H), 7.27 – 7.23 (m, 3H), 3.65 (dd, *J* = 8.5, 6.5 Hz, 1H), 3.53 (d, *J* = 13.5 Hz, 1H), 3.43 (d, *J* = 13.5 Hz, 1H), 1.87 – 1.76 (m, 2H), 1.68 – 1.61 (m, 5H), 1.36 – 1.27 (m, 1H), 1.24 – 1.08 (m, 7H), 0.85 – 0.76 (m, 2H); ^{13}C NMR (126 MHz, $CDCl_3$): δ 142.9, 138.6, 129.0, 128.5, 128.5, 128.2, 127.1, 126.9, 49.3, 37.5, 37.2, 36.8, 35.5, 33.5, 33.4, 26.8, 26.5, 26.5, 24.9; HRMS (ESI) calcd. for $C_{23}H_{30}SNa$ $[M+Na]^+$ *m/z* 361.1960, found 361.1963.

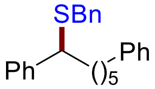

3q

benzyl(1,6-diphenylhexyl)sulfane (Figure 3, **3q**): From 1,6-diphenylhex-3-ene (**1q**) (94.5 mg, 0.40 mmol, 2.0 equiv.), the title compound was prepared following the general procedure **A** using NiI_2 (3.2 mg, 0.010 mmol, 5.0 mol%), **L1** (4.0 mg, 0.012 mmol, 6.0 mol%), benzyl mercaptan (**2a**) (25.0 mg, 0.20 mmol, 1.0 equiv.), HBpin (100 µL, 0.70 mmol, 3.5 equiv.), Li_3PO_4 (50 mg, 0.40 mmol, 2.0 equiv.) and THF (0.40 mL)/CH_3CN (0.20 mL). The reaction mixture was stirred at 60 °C for 24 h. The crude material was purified by flash column chromatography (using 1–2% ethyl acetate in hexanes) to provide the title compound **3q** as a colorless liquid in 91% yield (65.5 mg), >99:1 r.r.. IR (neat, cm^{-1}): 2927, 2853, 1493, 1452, 1261, 1070, 1028, 801, 695, 540; ^1H NMR (500 MHz, $CDCl_3$): δ 7.42 – 7.39 (m, 2H), 7.37 – 7.28 (m, 10H), 7.25 – 7.19 (m, 3H), 3.68 (dd, *J* = 8.5, 6.5 Hz, 1H), 3.57 (d, *J* = 13.5 Hz, 1H), 3.47 (d, *J* = 13.5 Hz, 1H), 2.60 (t, *J* = 7.8 Hz, 2H), 1.94 – 1.85 (m, 2H), 1.63 – 1.58 (m, 2H), 1.40 – 1.27 (m, 4H); ^{13}C NMR (126 MHz, $CDCl_3$): δ 142.8, 142.7, 138.6, 129.0, 128.5, 128.5, 128.4, 128.3, 128.1, 127.1, 126.9, 125.7, 49.2, 36.5, 35.9, 35.5, 31.3, 29.0, 27.5; HRMS (ESI) calcd. for $C_{25}H_{29}S$

[M+H]$^+$ *m/z* 361.1984, found 361.1986.

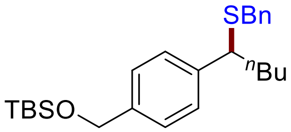

3r

((4-(1-(benzylthio)pentyl)benzyl)oxy)(tert-butyl)dimethylsilane (Figure 3, **3r**): From tert-butyldimethyl((4-(pent-3-en-1-yl)benzyl)oxy)silane (**1r**) (116.1 mg, 0.40 mmol, 2.0 equiv.), the title compound was prepared following the general procedure **A** using NiI_2 (3.2 mg, 0.010 mmol, 5.0 mol%), **L1** (4.0 mg, 0.012 mmol, 6.0 mol%), benzyl mercaptan (**2a**) (25.0 mg, 0.20 mmol, 1.0 equiv.), HBpin (100 µL, 0.70 mmol, 3.5 equiv.), Li_3PO_4 (50 mg, 0.40 mmol, 2.0 equiv.) and THF (0.40 mL)/CH_3CN (0.20 mL). The reaction mixture was stirred at 60 ^oC for 24 h. The crude material was purified by flash column chromatography (using 1–2% ethyl acetate in hexanes) to provide the title compound **3r** as a colorless liquid in 81% yield (67.1 mg), >99:1 r.r.. IR (neat, cm^{-1}): 2955, 2928, 2856, 1462, 1253, 1084, 836, 775, 698, 666; ^{1}H NMR (400 MHz, $CDCl_3$): δ 7.18 – 7.14 (m, 5H), 7.12 – 7.07 (m, 4H), 4.64 (s, 2H), 3.49 (dd, *J* = 8.4, 6.4 Hz, 1H), 3.37 (d, *J* = 13.6 Hz, 1H), 3.27 (d, *J* = 13.2 Hz, 1H), 1.74 – 1.63 (m, 2H), 1.16 – 0.99 (m, 4H), 0.84 (s, 9H), 0.69 (t, *J* = 7.0 Hz, 3H), 0.00 (s, 6H); ^{13}C NMR (101 MHz, $CDCl_3$): δ 141.4, 140.3, 138.7, 129.0, 128.5, 128.1, 126.9, 126.3, 65.0, 49.1, 36.3, 35.5, 29.9, 26.1, 22.5, 18.6, 14.0, -5.1; HRMS (ESI) calcd. for $C_{25}H_{39}OSSi$ [M+H]$^+$ *m/z* 415.2485, found 415.2496.

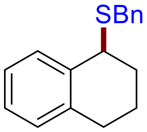

3s

benzyl(1,2,3,4-tetrahydronaphthalen-1-yl)sulfane (Figure 3, **3s**): From 1,4-dihydronaphthalene (**1s**) (52.0 mg, 0.40 mmol, 2.0 equiv.), the title compound was prepared following the general procedure **A** using NiI_2 (3.2 mg, 0.010 mmol, 5.0 mol%), **L1** (4.0 mg, 0.012 mmol, 6.0 mol%), benzyl mercaptan (**2a**) (25.0 mg, 0.20 mmol, 1.0 equiv.), HBpin (100 µL, 0.70 mmol, 3.5 equiv.), Li_3PO_4 (50 mg, 0.40 mmol, 2.0 equiv.)

and THF (0.40 mL)/CH_3CN (0.20 mL). The reaction mixture was stirred at 60 °C for 24 h. The crude material was purified by flash column chromatography (using 1–2% ethyl acetate in hexanes) to provide the title compound **3s** as a colorless liquid in 81% yield (41.1 mg), >99:1 r.r.. IR (neat, cm^{-1}): 3025, 2933, 1493, 1452, 1114, 934, 767, 733, 701; ^1H NMR (400 MHz, $CDCl_3$): δ 7.30 – 7.21 (m, 4H), 7.17 – 7.13 (m, 2H), 7.01 – 6.93 (m, 3H), 3.87 (dd, J = 8.4, 6.4 Hz, 1H), 3.70 (d, J = 13.6 Hz, 1H), 3.63 (d, J = 13.2 Hz, 1H), 2.75 – 2.68 (m, 1H), 2.66 – 2.57 (m, 1H), 2.09 – 1.88 (m, 3H), 1.71 – 1.63 (m, 1H); ^{13}C NMR (101 MHz, $CDCl_3$): δ 138.6, 137.6, 136.4, 130.4, 129.2, 129.0, 128.6, 127.0, 126.8, 125.7, 43.2, 36.2, 29.2, 28.8, 19.2; HRMS (ESI) calcd. for $C_{17}H_{19}S$ [M+H]$^+$ m/z 255.1202, found 255.1206.

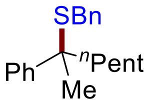

3t

benzyl(2-phenylheptan-2-yl)sulfane (Figure 3, **3t**): From hept-4-en-2-ylbenzene (**1t**) (69.7 mg, 0.40 mmol, 2.0 equiv.), the title compound was prepared following the general procedure **A** using NiI_2 (3.2 mg, 0.010 mmol, 5.0 mol%), **L1** (4.0 mg, 0.012 mmol, 6.0 mol%), benzyl mercaptan (**2a**) (25.0 mg, 0.20 mmol, 1.0 equiv.), HBpin (100 μL, 0.70 mmol, 3.5 equiv.), Li_3PO_4 (50 mg, 0.40 mmol, 2.0 equiv.) and THF (0.40 mL)/CH_3CN (0.20 mL). The reaction mixture was stirred at 60 °C for 24 h. The crude material was purified by flash column chromatography (using 1–2% ethyl acetate in hexanes) to provide the title compound **3t** as a colorless liquid in 73% yield (43.5 mg), >99:1 r.r.. IR (neat, cm^{-1}): 2954, 2859, 1493, 1453, 1445, 1376, 1056, 760, 694; ^1H NMR (400 MHz, $CDCl_3$): δ 7.48 – 7.46 (m, 2H), 7.28 – 7.24 (m, 2H), 7.16 – 7.03 (m, 6H), 3.37 (d, J = 12.0 Hz, 1H), 3.19 (d, J = 12.0 Hz, 1H), 1.95 – 1.88 (m, 1H), 1.80 – 1.73 (m, 1H), 1.65 (s, 3H), 1.32 – 1.19 (m, 1H), 1.14 – 1.08 (m, 4H), 1.06 – 0.95 (m, 1H), 0.75 (t, J = 7.0 Hz, 3H); ^{13}C NMR (101 MHz, $CDCl_3$): δ 145.2, 138.3, 129.1, 128.4, 128.2, 127.3, 126.8, 126.5, 52.6, 43.0, 34.0, 32.3, 26.2, 24.4, 22.6, 14.2; HRMS (ESI) calcd. for $C_{20}H_{27}S$ [M+H]$^+$ m/z 299.1828, found 299.1831.

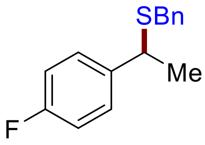

3u

benzyl(1-(4-fluorophenyl)ethyl)sulfane (Figure 3, **3u**): From 1-fluoro-4-vinylbenzene (**1u**) (48.8 mg, 0.40 mmol, 2.0 equiv.), the title compound was prepared following the general procedure **A** using NiI_2 (3.2 mg, 0.010 mmol, 5.0 mol%), **L1** (4.0 mg, 0.012 mmol, 6.0 mol%), benzyl mercaptan (**2a**) (25.0 mg, 0.20 mmol, 1.0 equiv.), HBpin (100 μL, 0.70 mmol, 3.5 equiv.), Li_3PO_4 (50 mg, 0.40 mmol, 2.0 equiv.) and THF (0.40 mL)/CH_3CN (0.20 mL). The reaction mixture was stirred at 60 °C for 24 h. The crude material was purified by flash column chromatography (using 1–2% ethyl acetate in hexanes) to provide the title compound **3u** as a colorless liquid in 73% yield (35.9 mg), >99:1 r.r.. IR (neat, cm^{-1}): 2969, 1601, 1506, 1452, 1221, 1156, 834, 696, 519, 471; ^1H NMR (400 MHz, $CDCl_3$): δ 7.23 – 7.13 (m, 7H), 6.95 – 6.91 (m, 2H), 3.71 (q, J = 7.1 Hz, 1H), 3.47 (d, J = 13.6 Hz, 1H), 3.35 (d, J = 13.6 Hz, 1H), 1.42 (d, J = 6.8 Hz, 3H); ^{13}C NMR (101 MHz, $CDCl_3$): δ 161.9 (d, $J_{C\text{-}F}$ = 245.4 Hz), 139.6 (d, $J_{C\text{-}F}$ = 3.0 Hz), 138.4, 129.1 (d, $J_{C\text{-}F}$ = 8.1 Hz), 129.0, 128.6, 127.0, 115.4 (d, $J_{C\text{-}F}$ = 22.2 Hz), 42.9, 35.8, 22.8; ^{19}F NMR (471 MHz, $CDCl_3$): δ -115.6; HRMS (ESI) calcd. for $C_{15}H_{16}FS$ $[M+H]^+$ *m/z* 247.0951, found 247.0948.

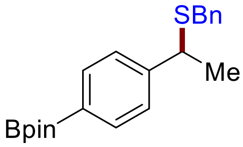

3v

2-(4-(1-(benzylthio)ethyl)phenyl)-4,4,5,5-tetramethyl-1,3,2-dioxaborolane (Figure 3, **3v**): From 4,4,5,5-tetramethyl-2-(4-vinylphenyl)-1,3,2-dioxaborolane (**1v**) (92.1 mg, 0.40 mmol, 2.0 equiv.), the title compound was prepared following the general procedure **A** using NiI_2 (3.2 mg, 0.010 mmol, 5.0 mol%), **L1** (4.0 mg, 0.012 mmol, 6.0 mol%), benzyl mercaptan (**2a**) (25.0 mg, 0.20 mmol, 1.0 equiv.), HBpin (100 μL, 0.70 mmol, 3.5 equiv.), Li_3PO_4 (50 mg, 0.40 mmol, 2.0 equiv.) and THF (0.40 mL)/CH_3CN (0.20 mL). The reaction mixture was stirred at 60 °C for 48 h. The crude material was purified by flash

column chromatography (using 1–2% ethyl acetate in hexanes) to provide the title compound **3v** as a colorless liquid in 74% yield (52.4 mg), >99:1 r.r.. IR (neat, cm^{-1}): 2976, 2925, 1609, 1453, 1397, 1357, 1142, 1089, 858, 710, 697, 657; ^{1}H NMR (400 MHz, $CDCl_3$): δ 7.73 – 7.71 (m, 2H), 7.27 – 7.18 (m, 4H), 7.15 – 7.12 (m, 4H), 3.72 (q, *J* = 7.2 Hz, 1H), 3.43 (d, *J* = 13.6 Hz, 1H), 3.32 (d, *J* = 13.6 Hz, 1H), 1.43 (d, *J* = 7.2 Hz, 3H), 1.28 (s, 12H); ^{13}C NMR (101 MHz, $CDCl_3$): δ 147.2, 138.4, 135.2 (2C), 129.0, 128.5, 127.0, 127.0, 83.9, 43.7, 35.8, 25.0, 22.5; HRMS (ESI) calcd. for $C_{21}H_{27}BO_2SNa$ $[M+Na]^+$ *m/z* 377.1717, found 377.1716.

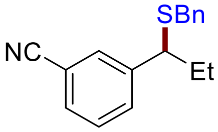

3w

3-(1-(benzylthio)propyl)benzonitrile (Figure 3, **3w**): From 3-allylbenzonitrile (**1w**) (57.2 mg, 0.40 mmol, 2.0 equiv.), the title compound was prepared following the general procedure **A** using NiI_2 (3.2 mg, 0.010 mmol, 5.0 mol%), **L1** (4.0 mg, 0.012 mmol, 6.0 mol%), benzyl mercaptan (**2a**) (25.0 mg, 0.20 mmol, 1.0 equiv.), HBpin (100 μL, 0.70 mmol, 3.5 equiv.), Li_3PO_4 (50 mg, 0.40 mmol, 2.0 equiv.) and THF (0.40 mL)/CH_3CN (0.20 mL). The reaction mixture was stirred at 60 °C for 24 h. The crude material was purified by flash column chromatography (using 1–2% ethyl acetate in hexanes) to provide the title compound **3w** as a colorless liquid in 92% yield (49.1 mg), >99:1 r.r.. IR (neat, cm^{-1}): 2965, ,2929, 2229, 1494, 1453, 1431, 1103, 796, 760, 691, 486; ^{1}H NMR (400 MHz, $CDCl_3$): δ 7.46 – 7.41 (m, 3H), 7.36 – 7.32 (m, 1H), 7.23 – 7.15 (m, 3H), 7.10 – 7.08 (m, 2H), 3.46 (d, *J* = 14.0 Hz, 1H), 3.44 (dd, *J* = 8.4, 6.4 Hz, 1H), 3.31 (d, *J* = 13.6 Hz, 1H), 1.85 – 1.78 (m, 1H), 1.72 – 1.65 (m, 1H), 0.75 (t, *J* = 7.4 Hz, 3H); ^{13}C NMR (101 MHz, $CDCl_3$): δ 144.5, 137.8, 132.7, 131.8, 130.8, 129.3, 128.9, 128.6, 127.2, 118.9, 112.6, 50.3, 35.7, 29.6, 12.2; HRMS (ESI) calcd. for $C_{17}H_{18}NS$ $[M+H]^+$ *m/z* 268.1154, found 268.1160.

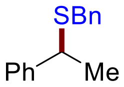 **3x**

benzyl(1-phenylethyl)sulfane (Figure 3, **3x**): From styrene (**1x**) (41.6 mg, 0.40 mmol, 2.0 equiv.), the title compound was prepared following the general procedure **A** using NiI_2 (3.2 mg, 0.010 mmol, 5.0 mol%), **L1** (4.0 mg, 0.012 mmol, 6.0 mol%), benzyl mercaptan (**2a**) (25.0 mg, 0.20 mmol, 1.0 equiv.), HBpin (100 µL, 0.70 mmol, 3.5 equiv.), Li_3PO_4 (50 mg, 0.40 mmol, 2.0 equiv.) and THF (0.40 mL)/CH_3CN (0.20 mL). The reaction mixture was stirred at 60 °C for 24 h. The crude material was purified by flash column chromatography (using 1–2% ethyl acetate in hexanes) to provide the title compound **3x** as a colorless liquid in 72% yield (32.8 mg), >99:1 r.r.. IR (neat, cm^{-1}): 2969, 1493, 1453, 1261, 1027, 904, 725, 649, 530; ^1H NMR (400 MHz, $CDCl_3$): δ 7.25 – 7.19 (m, 6H), 7.17 – 7.13 (m, 4H), 3.72 (q, *J* = 7.1 Hz, 1H), 3.46 (d, *J* = 13.6 Hz, 1H), 3.36 (d, *J* = 13.6 Hz, 1H), 1.45 (d, *J* = 6.8 Hz, 3H); ^{13}C NMR (101 MHz, $CDCl_3$): δ 143.9, 138.5, 129.0, 128.6, 128.5, 127.6, 127.2, 127.0, 43.7, 35.8, 22.7; HRMS (ESI) calcd. for $C_{15}H_{17}S$ $[M+H]^+$ *m/z* 229.1045, found 229.1037.

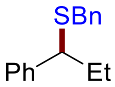 **3y**

benzyl(1-phenylpropyl)sulfane (Figure 3, **3y**): From (*E*)-prop-1-en-1-ylbenzene (**1y**) (47.2 mg, 0.40 mmol, 2.0 equiv.), the title compound was prepared following the general procedure **A** using NiI_2 (3.2 mg, 0.010 mmol, 5.0 mol%), **L1** (4.0 mg, 0.012 mmol, 6.0 mol%), benzyl mercaptan (**2a**) (25.0 mg, 0.20 mmol, 1.0 equiv.), HBpin (100 µL, 0.70 mmol, 3.5 equiv.), Li_3PO_4 (50 mg, 0.40 mmol, 2.0 equiv.) and THF (0.40 mL)/CH_3CN (0.20 mL). The reaction mixture was stirred at 60 °C for 24 h. The crude material was purified by flash column chromatography (using 1–2% ethyl acetate in hexanes) to provide the title compound **3y** as a colorless liquid in 76% yield (36.7 mg), 98:2 r.r.. IR (neat, cm^{-1}): 2963, 2928, 1452, 1416, 1260, 1069, 1027, 907, 757, 695; ^1H NMR (400 MHz, $CDCl_3$): δ 7.27 – 7.16 (m, 6H), 7.15 – 7.11 (m, 41H), 3.46 (dd, *J* = 8.4, 6.4 Hz, 1H), 3.42 (d, *J* = 13.6 Hz, 1H), 3.32 (d, *J* = 13.6 Hz, 1H), 1.86 – 1.69 (m, 2H), 0.76 (t, *J* = 7.4

Hz, 3H); ^{13}C NMR (101 MHz, $CDCl_3$): δ 142.5, 138.6, 129.0, 128.5, 128.5, 128.3, 127.1, 126.9, 51.1, 35.5, 29.7, 12.4; HRMS (ESI) calcd. for $C_{16}H_{18}SNa$ $[M+Na]^+$ *m/z* 265.1021, found 265.1023.

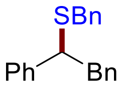

3z

benzyl(1,2-diphenylethyl)sulfane (Figure 3, **3z**): From (*E*)-1,2-diphenylethene (**1z**) (72.0 mg, 0.40 mmol, 2.0 equiv.), the title compound was prepared following the general procedure **A** using NiI_2 (3.2 mg, 0.010 mmol, 5.0 mol%), **L1** (4.0 mg, 0.012 mmol, 6.0 mol%), benzyl mercaptan (**2a**) (25.0 mg, 0.20 mmol, 1.0 equiv.), HBpin (100 µL, 0.70 mmol, 3.5 equiv.), Li_3PO_4 (50 mg, 0.40 mmol, 2.0 equiv.) and THF (0.40 mL)/CH_3CN (0.20 mL). The reaction mixture was stirred at 60 °C for 24 h. The crude material was purified by flash column chromatography (using 1–2% ethyl acetate in hexanes) to provide the title compound **3z** as a colorless liquid in 95% yield (57.7 mg). IR (neat, cm^{-1}): 3061, 3027, 1601, 1493, 1452, 1070, 1029, 766, 747, 696; ^{1}H NMR (400 MHz, $CDCl_3$): δ 7.20 – 7.11 (m, 7H), 7.07 – 7.04 (m, 6H), 6.87 – 6.85 (m, 2H), 3.78 (dd, *J* = 8.4, 6.4 Hz, 1H), 3.40 (d, *J* = 13.2 Hz, 1H), 3.33 (d, *J* = 13.6 Hz, 1H), 3.08 – 2.96 (m, 2H); ^{13}C NMR (101 MHz, $CDCl_3$): δ 141.8, 138.9, 138.2, 129.3, 129.0, 128.5 (2C), 128.3, 128.2, 127.3, 127.0, 126.4, 51.0, 43.2, 35.8; HRMS (ESI) calcd. for $C_{21}H_{21}S$ $[M+H]^+$ *m/z* 305.1358, found 305.1355.

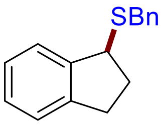

3a'

benzyl(2,3-dihydro-1*H*-inden-1-yl)sulfane (Figure 3, **3a'**): From 1*H*-indene (**1a'**) (46.4 mg, 0.40 mmol, 2.0 equiv.), the title compound was prepared following the general procedure **A** using NiI_2 (3.2 mg, 0.010 mmol, 5.0 mol%), **L1** (4.0 mg, 0.012 mmol, 6.0 mol%), benzyl mercaptan (**2a**) (25.0 mg, 0.20 mmol, 1.0 equiv.), HBpin (100 µL, 0.70 mmol, 3.5 equiv.), Li_3PO_4 (50 mg, 0.40 mmol, 2.0 equiv.) and THF (0.40 mL)/CH_3CN (0.20 mL). The reaction mixture was stirred at 60 °C for 24 h. The crude material was

purified by flash column chromatography (using 1–2% ethyl acetate in hexanes) to provide the title compound **3a'** as a colorless liquid in 78% yield (37.4 mg), >99:1 r.r.. IR (neat, cm^{-1}): 2945, 2928, 1494, 1454, 1071, 1027, 903, 724, 649; ^{1}H NMR (400 MHz, $CDCl_3$): δ 7.27 – 7.20 (m, 5H), 7.17 – 7.08 (m, 4H), 4.16 (dd, *J* = 7.2, 5.2 Hz, 1H), 3.69 (d, *J* = 13.2 Hz, 1H), 3.65 (d, *J* = 13.2 Hz, 1H), 3.01 – 2.93 (m, 1H), 2.79 – 2.72 (m, 1H), 2.41 – 2.32 (m, 1H), 2.08 – 2.01 (m, 1H); ^{13}C NMR (101 MHz, $CDCl_3$): δ 143.8, 143.3, 138.6, 129.0, 128.6, 127.6, 127.0, 126.6, 124.9, 124.8, 48.7, 35.9, 34.0, 31.1; HRMS (ESI) calcd. for $C_{16}H_{17}S$ $[M+H]^+$ *m/z* 241.1045, found 241.1042.

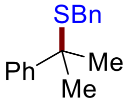

3b'

benzyl(2-phenylpropan-2-yl)sulfane (Figure 3, **3b'**): From prop-1-en-2-ylbenzene (**1b'**) (47.2 mg, 0.40 mmol, 2.0 equiv.), the title compound was prepared following the general procedure **A** using NiI_2 (3.2 mg, 0.010 mmol, 5.0 mol%), **L1** (4.0 mg, 0.012 mmol, 6.0 mol%), benzyl mercaptan (**2a**) (25.0 mg, 0.20 mmol, 1.0 equiv.), HBpin (100 µL, 0.70 mmol, 3.5 equiv.), Li_3PO_4 (50 mg, 0.40 mmol, 2.0 equiv.) and THF (0.40 mL)/CH_3CN (0.20 mL). The reaction mixture was stirred at 60 °C for 48 h. The crude material was purified by flash column chromatography (using 1–2% ethyl acetate in hexanes) to provide the title compound **3b'** as a colorless liquid in 69% yield (33.4 mg), 97:3 r.r.. IR (neat, cm^{-1}): 2975, 2942, 1494, 1452, 1236, 1133, 1029, 768, 748, 697; ^{1}H NMR (400 MHz, $CDCl_3$): δ 7.52 – 7.50 (m, 2H), 7.30 – 7.26 (m, 3H), 7.19 – 7.04 (m, 5H), 3.32 (s, 2H), 1.64 (s, 6H); ^{13}C NMR (101 MHz, $CDCl_3$): δ 146.4, 138.2, 129.0, 128.5, 128.3, 126.8, 126.8, 126.7, 48.7, 34.6, 30.4; HRMS (ESI) calcd. for $C_{16}H_{19}S$ $[M+H]^+$ *m/z* 243.1202, found 243.1200.

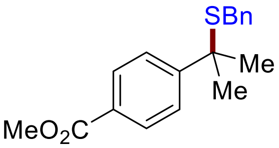

3c'

methyl 4-(2-(benzylthio)propan-2-yl)benzoate (Figure 3, **3c'**): From methyl 4-(prop-1-en-2-yl)benzoate (**1c'**) (70.4 mg, 0.40 mmol, 2.0 equiv.), the title compound was prepared following the general procedure **A** using NiI_2 (3.2 mg, 0.010 mmol, 5.0 mol%), **L1** (4.0 mg, 0.012 mmol, 6.0 mol%), benzyl mercaptan (**2a**) (25.0 mg, 0.20 mmol, 1.0 equiv.), HBpin (100 μL, 0.70 mmol, 3.5 equiv.), Li_3PO_4 (50 mg, 0.40 mmol, 2.0 equiv.) and THF (0.40 mL)/CH_3CN (0.20 mL). The reaction mixture was stirred at 60 °C for 24 h. The crude material was purified by flash column chromatography (using 1–2% ethyl acetate in hexanes) to provide the title compound **3c'** as a colorless liquid in 68% yield (40.8 mg), >99:1 r.r.. IR (neat, cm^{-1}): 2951, 2924, 1719, 1607, 1275, 1187, 1112, 1017, 855, 754, 705, 665, 560; ^1H NMR (400 MHz, $CDCl_3$): δ 7.94 – 7.92 (m, 2H), 7.57 – 7.55 (m, 2H), 7.13 – 7.01 (m, 5H), 3.84 (s, 3H), 3.29 (s, 2H), 1.64 (s, 6H); ^{13}C NMR (101 MHz, $CDCl_3$): δ 167.0, 151.8, 137.8, 129.6, 129.0, 128.5, 128.4, 126.9, 126.8, 52.2, 48.5, 34.6, 30.1; HRMS (ESI) calcd. for $C_{18}H_{21}O_2S$ $[M+H]^+$ *m/z* 301.1257, found 301.1257.

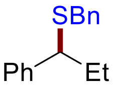

3d'

benzyl(1-phenylpropyl)sulfane (Figure 3, **3d'**): From prop-1-yn-1-ylbenzene (**1d'**) (46.4 mg, 0.40 mmol, 2.0 equiv.), the title compound was prepared following the general procedure **A** using NiI_2 (3.2 mg, 0.010 mmol, 5.0 mol%), **L1** (4.0 mg, 0.012 mmol, 6.0 mol%), benzyl mercaptan (**2a**) (25.0 mg, 0.20 mmol, 1.0 equiv.), HBpin (100 μL, 0.70 mmol, 3.5 equiv.), Li_3PO_4 (50 mg, 0.40 mmol, 2.0 equiv.) and THF (0.40 mL)/CH_3CN (0.20 mL). The reaction mixture was stirred at 60 °C for 48 h. The crude material was purified by flash column chromatography (using 1–2% ethyl acetate in hexanes) to provide the title compound **3d'** as a colorless liquid in 76% yield (36.7 mg), 98:2 r.r.. IR (neat, cm^{-1}): 2963, 2928, 1452, 1416, 1260, 1069, 1027, 907, 757, 695; ^1H NMR (400 MHz, $CDCl_3$): δ 7.26 – 7.16 (m, 6H), 7.14 – 7.11 (m, 4H), 3.46 (dd, J = 8.4, 6.4 Hz, 1H),

3.42 (d, J = 13.6 Hz, 1H), 3.32 (d, J = 13.6 Hz, 1H), 1.84 – 1.68 (m, 2H), 0.75 (t, J = 7.4 Hz, 3H); ^{13}C NMR (101 MHz, $CDCl_3$): δ 142.5, 138.6, 129.0, 128.5, 128.5, 128.3, 127.1, 126.9, 51.1, 35.5, 29.7, 12.4; HRMS (ESI) calcd. for $C_{16}H_{18}SNa$ $[M+Na]^+$ *m/z* 265.1021, found 265.1023.

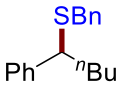

3e'

benzyl(1-phenylpentyl)sulfane (Figure 3, **3e'**): From pent-3-yn-1-ylbenzene (**1e'**) (57.6 mg, 0.40 mmol, 2.0 equiv.), the title compound was prepared following the general procedure **A** using NiI_2 (3.2 mg, 0.010 mmol, 5.0 mol%), **L1** (4.0 mg, 0.012 mmol, 6.0 mol%), benzyl mercaptan (**2a**) (25.0 mg, 0.20 mmol, 1.0 equiv.), HBpin (100 μL, 0.70 mmol, 3.5 equiv.), Li_3PO_4 (50 mg, 0.40 mmol, 2.0 equiv.) and THF (0.40 mL)/CH_3CN (0.20 mL). The reaction mixture was stirred at 60 ^{o}C for 48 h. The crude material was purified by flash column chromatography (using 1–2% ethyl acetate in hexanes) to provide the title compound **3e'** as a colorless liquid in 78% yield (42.1 mg), >99:1 r.r.. IR (neat, cm^{-1}): 2956, 2928, 1492, 1453, 1070, 1029, 764, 697, 541; ^{1}H NMR (400 MHz, $CDCl_3$): δ 7.26 – 7.16 (m, 6H), 7.14 – 7.11 (m, 4H), 3.53 (dd, J = 8.4, 6.8 Hz, 1H), 3.41 (d, J = 13.2 Hz, 1H), 3.31 (d, J = 13.6 Hz, 1H), 1.80 – 1.65 (m, 2H), 1.21 – 1.05 (m, 4H), 0.73 (t, J = 7.0 Hz, 3H); ^{13}C NMR (101 MHz, $CDCl_3$): δ 142.9, 138.6, 129.0, 128.5, 128.5, 128.2, 127.1, 126.9, 49.4, 36.3, 35.5, 29.9, 22.5, 14.0; HRMS (ESI) calcd. for $C_{18}H_{23}S$ $[M+H]^+$ *m/z* 271.1515, found 271.1517.

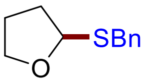

3f'

2-(benzylthio)tetrahydrofuran (Figure 3, **3f'**): From 2,5-dihydrofuran (**1f'**) (28.0 mg, 0.40 mmol, 2.0 equiv.), the title compound was prepared following the general procedure **A** using NiI_2 (3.2 mg, 0.010 mmol, 5.0 mol%), **L1** (4.0 mg, 0.012 mmol, 6.0 mol%), benzyl mercaptan (**2a**) (25.0 mg, 0.20 mmol, 1.0 equiv.), HBpin (100 μL, 0.70 mmol, 3.5 equiv.), Li_3PO_4 (50 mg, 0.40 mmol, 2.0 equiv.) and THF (0.40 mL)/CH_3CN (0.20 mL).

The reaction mixture was stirred at 60 °C for 24 h. The crude material was purified by flash column chromatography (using 1–2% ethyl acetate in hexanes) to provide the title compound **3f'** as a colorless liquid in 64% yield (24.8 mg), >99:1 r.r.. IR (neat, cm^{-1}): 2976, 2946, 1494, 1453, 1045, 934, 904, 769, 699, 648; ^{1}H NMR (500 MHz, $CDCl_3$): δ 7.40 – 7.38 (m, 2H), 7.36 – 7.33 (m, 2H), 7.28 – 7.25 (m, 1H), 5.26 (dd, *J* = 7.2, 3.6 Hz, 1H), 4.03 – 3.94 (m, 2H), 3.93 (d, *J* = 13.4 Hz, 1H), 3.79 (d, *J* = 13.4 Hz, 1H), 2.26 – 2.19 (m, 1H), 2.07 – 1.99 (m, 1H), 1.90 – 1.79 (m, 2H); ^{13}C NMR (126 MHz, $CDCl_3$): δ 138.6, 129.0, 128.5, 126.9, 83.0, 66.8, 35.0, 32.1, 24.9; HRMS (ESI) calcd. for $C_{11}H_{14}OSNa$ $[M+Na]^+$ *m/z* 217.0658, found 217.0664.

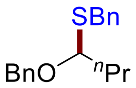

3g'

benzyl(1-(benzyloxy)butyl)sulfane (Figure 3, **3g'**): From ((but-3-en-1-yloxy)methyl)benzene (**1g'**) (64.8 mg, 0.40 mmol, 2.0 equiv.), the title compound was prepared following the general procedure **A** using NiI_2 (3.2 mg, 0.010 mmol, 5.0 mol%), **L1** (4.0 mg, 0.012 mmol, 6.0 mol%), benzyl mercaptan (**2a**) (25.0 mg, 0.20 mmol, 1.0 equiv.), HBpin (100 µL, 0.70 mmol, 3.5 equiv.), Li_3PO_4 (50 mg, 0.40 mmol, 2.0 equiv.) and THF (0.40 mL)/CH_3CN (0.20 mL). The reaction mixture was stirred at 60 °C for 24 h. The crude material was purified by flash column chromatography (using 1–2% ethyl acetate in hexanes) to provide the title compound **3g'** as a colorless liquid in 65% yield (37.2 mg), >99:1 r.r.. IR (neat, cm^{-1}): 2975, 2249, 2124, 1053, 1024, 1006, 820, 757, 679, 622; ^{1}H NMR (400 MHz, DMSO-d_6): δ 7.38 – 7.34 (m, 2H), 7.31 – 7.28 (m, 6H), 7.25 – 7.21 (m, 2H), 4.66 (d, *J* = 11.6 Hz, 1H), 4.61 (dd, *J* = 7.2, 6.4 Hz, 1H), 4.45 (d, *J* = 12.0 Hz, 1H), 3.82 (d, *J* = 13.2 Hz, 1H), 3.78 (d, *J* = 12.8 Hz, 1H), 1.89 – 1.80 (m, 1H), 1.73 – 1.64 (m, 1H), 1.41 – 1.31 (m, 2H), 0.81 (t, *J* = 7.4 Hz, 3H); ^{13}C NMR (101 MHz, DMSO-d_6): δ 138.9, 138.0, 128.9, 128.4, 128.3, 127.8, 127.6, 126.8, 84.6, 68.3, 37.6, 32.2, 19.0, 13.5; HRMS (ESI) calcd. for $C_{18}H_{22}OSNa$ $[M+Na]^+$ *m/z* 309.1284, found 309.1281.

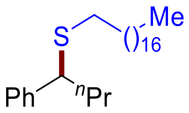

4b

octadecyl(1-phenylbutyl)sulfane (Figure 4, **4b**): From octadecane-1-thiol (**2b**) (57.3 mg, 0.20 mmol, 1.0 equiv.), the title compound was prepared following the general procedure **B** using NiI_2 (6.4 mg, 0.020 mmol, 10 mol%), **L1** (8.0 mg, 0.024 mmol, 12 mol%), 4-phenyl-1-butene (**1a**) (52.8 mg, 0.40 mmol, 2.0 equiv.), HBpin (100 μL, 0.70 mmol, 3.5 equiv.), Li_3PO_4 (50 mg, 0.40 mmol, 2.0 equiv.) and THF (0.40 mL)/DMPU (0.20 mL). The reaction mixture was stirred at 50 °C for 48 h. The crude material was purified by flash column chromatography (using 1–2% ethyl acetate in hexanes) to provide the title compound **4b** as a colorless liquid in 82% yield (68.7 mg), >99:1 r.r.. IR (neat, cm^{-1}): 2955, 2921, 2851, 1491, 1464, 748, 722, 697; ^{1}H NMR (500 MHz, $CDCl_3$): δ 7.33 – 7.32 (m, 4H), 7.26 – 7.22 (m, 1H), 3.78 (dd, *J* = 8.5, 6.5 Hz, 1H), 2.33 – 2.28 (m, 1H), 2.26 – 2.20 (m, 1H), 1.90 – 1.80 (m, 2H), 1.51 – 1.44 (m, 2H), 1.34 – 1.23 (m, 32H), 0.91 (t, *J* = 6.8 Hz, 3H), 0.90 (t, *J* = 7.5 Hz, 3H); ^{13}C NMR (126 MHz, $CDCl_3$): δ 143.3, 128.5, 128.0, 127.0, 49.6, 38.9, 32.1, 31.2, 29.9 (5C), 29.8, 29.8, 29.8, 29.7, 29.6, 29.5 (2C), 29.3, 29.1, 22.9, 21.0, 14.3, 14.0; HRMS (ESI) calcd. for $C_{28}H_{50}SNa$ $[M+Na]^+$ *m/z* 441.3525, found 441.3515.

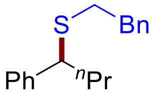

4c

phenethyl(1-phenylbutyl)sulfane (Figure 4, **4c**): From 2-phenylethane-1-thiol (**2c**) (27.6 mg, 0.20 mmol, 1.0 equiv.), the title compound was prepared following the general procedure **B** using NiI_2 (6.4 mg, 0.020 mmol, 10 mol%), **L1** (8.0 mg, 0.024 mmol, 12 mol%), 4-phenyl-1-butene (**1a**) (52.8 mg, 0.40 mmol, 2.0 equiv.), HBpin (100 μL, 0.70 mmol, 3.5 equiv.), Li_3PO_4 (50 mg, 0.40 mmol, 2.0 equiv.) and THF (0.40 mL)/DMPU (0.20 mL). The reaction mixture was stirred at 50 °C for 48 h. The crude material was purified by flash column chromatography (using 1–2% ethyl acetate in hexanes) to provide the title compound **4c** as a colorless liquid in 90% yield (48.6 mg), >99:1 r.r.. IR (neat, cm^{-1}): 3026, 2957, 2930, 1495, 1453, 1030, 748, 724, 698; ^{1}H NMR (500 MHz,

CDCl3): δ 7.36 – 7.31 (m, 4H), 7.29 – 7.26 (m, 3H), 7.22 – 7.19 (m, 1H), 7.10 – 7.09 (m, 2H), 3.80 (dd, J = 8.5, 6.5 Hz, 1H), 2.83 – 2.77 (m, 1H), 2.75 – 2.69 (m, 1H), 2.58 – 2.48 (m, 2H), 1.91 – 1.79 (m, 2H), 1.39 – 1.25 (m, 2H), 0.90 (t, J = 7.5 Hz, 3H); ^{13}C NMR (126 MHz, $CDCl_3$): δ 143.0, 140.9, 128.6, 128.6, 128.5, 128.0, 127.1, 126.3, 49.8, 38.8, 36.4, 32.6, 21.0, 13.9; HRMS (ESI) calcd. for $C_{18}H_{23}S$ $[M+H]^+$ *m/z* 271.1515, found 271.1516.

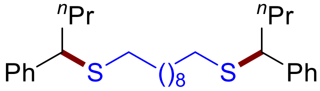

4d

1,10-bis((1-phenylbutyl)thio)decane (Figure 4, **4d**): From decane-1,10-dithiol (**2d**) (20.6 mg, 0.10 mmol, 0.5 equiv.), the title compound was prepared following the general procedure **B** using NiI_2 (6.4 mg, 0.020 mmol, 10 mol%), **L1** (8.0 mg, 0.024 mmol, 12 mol%), 4-phenyl-1-butene (**1a**) (52.8 mg, 0.40 mmol, 2.0 equiv.), HBpin (100 µL, 0.70 mmol, 3.5 equiv.), Li_3PO_4 (50 mg, 0.40 mmol, 2.0 equiv.) and THF (0.40 mL)/DMPU (0.20 mL). The reaction mixture was stirred at 50 °C for 48 h. The crude material was purified by flash column chromatography (using 1–2% ethyl acetate in hexanes) to provide the title compound **4d** as a colorless liquid in 87% yield (41.0 mg), >95:5 r.r.. IR (neat, cm^{-1}): 2956, 2925, 2853, 1491, 1453, 1029, 749, 723, 698; ^{1}H NMR (500 MHz, $CDCl_3$): δ 7.34 – 7.30 (m, 8H), 7.26 – 7.22 (m, 2H), 3.78 (dd, J = 8.5, 6.5 Hz, 2H), 2.33 – 2.27 (m, 2H), 2.25 – 2.20 (m, 2H), 1.90 – 1.80 (m, 4H), 1.52 – 1.41 (m, 4H), 1.39 – 1.24 (m, 8H), 1.24 – 1.19 (m, 8H), 0.90 (t, J = 7.5 Hz, 6H); ^{13}C NMR (126 MHz, $CDCl_3$): δ 143.3 (2C), 128.5 (4C), 128.0 (4C), 127.0 (2C), 49.6 (2C), 38.9 (2C), 31.1 (2C), 29.5 (4C), 29.3 (2C), 29.0 (2C), 21.0 (2C), 14.0 (2C); HRMS (ESI) calcd. for $C_{30}H_{46}S_2Na$ $[M+Na]^+$ *m/z* 493.2933, found 493.2933.

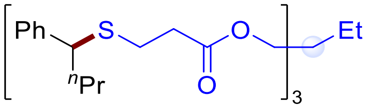

4e

2-ethyl-2-(((3-((1-phenylbutyl)thio)propanoyl)oxy)methyl)propane-1,3-diyl bis(3-((1-phenylbutyl)thio)propanoate) (Figure 4, **4e**): From 2-ethyl-2-(((3-

mercaptopropanoyl)oxy)methyl)propane-1,3-diyl bis(3-mercaptopropanoate) (**2e**) (39.8 mg, 0.10 mmol, 0.5 equiv.), the title compound was prepared following the general procedure **B** using NiI_2 (6.4 mg, 0.020 mmol, 10 mol%), **L1** (8.0 mg, 0.024 mmol, 12 mol%), 4-phenyl-1-butene (**1a**) (79.2 mg, 0.60 mmol, 3.0 equiv.), HBpin (100 μL, 0.70 mmol, 3.5 equiv.), Li_3PO_4 (50 mg, 0.40 mmol, 2.0 equiv.) and THF (0.40 mL)/DMPU (0.20 mL). The reaction mixture was stirred at 50 °C for 48 h. The crude material was purified by flash column chromatography (using 1–2% ethyl acetate in hexanes) to provide the title compound **4e** as a colorless liquid in 78% yield (62.0 mg), >95:5 r.r.. IR (neat, cm^{-1}): 2958, 2930, 1737, 1453, 1350, 1239, 1139, 750, 722, 699; ^1H NMR (500 MHz, $CDCl_3$): δ 7.37 – 7.30 (m, 12H), 7.26 – 7.23 (m, 3H), 3.96 (s, 6H), 3.81 (dd, *J* = 8.5, 6.5 Hz, 3H), 2.53 – 2.50 (m, 6H), 2.43 – 2.40 (m, 6H), 1.87 – 1.79 (m, 6H), 1.41 (q, *J* = 7.5 Hz, 2H), 1.38 – 1.25 (m, 6H), 0.89 (t, *J* = 7.3 Hz, 9H), 0.84 (t, *J* = 7.8 Hz, 3H); ^{13}C NMR (126 MHz, $CDCl_3$): δ 171.6 (3C), 142.6 (3C), 128.6 (6C), 128.0 (6C), 127.3 (3C), 63.9 (3C), 49.8 (3C), 40.7, 38.6 (3C), 34.6 (3C), 26.1 (3C), 23.0, 21.0 (3C), 13.9 (3C), 7.5; HRMS (ESI) calcd. for $C_{45}H_{62}O_6S_3Na$ $[M+Na]^+$ *m/z* 817.3601, found 817.3603.

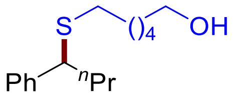

4f

6-((1-phenylbutyl)thio)hexan-1-ol (Figure 4, **4f**): From 6-mercaptohexan-1-ol (**2f**) (26.8 mg, 0.20 mmol, 1.0 equiv.), the title compound was prepared following the general procedure **B** using NiI_2 (6.4 mg, 0.020 mmol, 10 mol%), **L1** (8.0 mg, 0.024 mmol, 12 mol%), 4-phenyl-1-butene (**1a**) (52.8 mg L, 0.40 mmol, 2.0 equiv.), HBpin (100 μL, 0.70 mmol, 3.5 equiv.), Li_3PO_4 (50 mg, 0.40 mmol, 2.0 equiv.) and THF (0.40 mL)/DMPU (0.20 mL). The reaction mixture was stirred at 50 °C for 48 h. The crude material was purified by flash column chromatography (using 1–2% ethyl acetate in hexanes) to provide the title compound **4f** as a colorless liquid in 89% yield (47.4), >95:5 r.r.. IR (neat, cm^{-1}): 3351, 2928, 2856, 1491, 1453, 1053, 1029, 749, 723, 698; ^1H NMR (500 MHz, $CDCl_3$): δ 7.34 – 7.30 (m, 4H), 7.26 – 7.22 (m, 1H), 3.77 (dd, *J* = 8.5, 6.5 Hz, 1H), 3.62 (t, *J* = 6.5 Hz, 2H), 2.35 – 2.29 (m, 1H), 2.26 – 2.21 (m, 1H), 1.89 – 1.79 (m, 2H),

1.56 – 1.47 (m, 4H), 1.38 – 1.26 (m, 6H), 0.90 (t, *J* = 7.5 Hz, 3H); ^{13}C NMR (126 MHz, $CDCl_3$): δ 143.2, 128.5, 127.9, 127.0, 63.0, 49.6, 38.8, 32.7, 31.0, 29.4, 28.7, 25.4, 21.0, 13.9; HRMS (ESI) calcd. for $C_{16}H_{27}OS$ $[M+H]^+$ *m/z* 267.1777, found 267.1775.

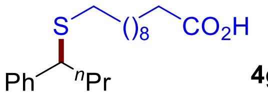

4g

11-((1-phenylbutyl)thio)undecanoic acid (Figure 4, **4g**): From 11-mercaptoundecanoic acid (**2g**) (43.6 mg, 0.20 mmol, 1.0 equiv.), the title compound was prepared following the general procedure **B** using NiI_2 (6.4 mg, 0.020 mmol, 10 mol%), **L1** (8.0 mg, 0.024 mmol, 12 mol%), 4-phenyl-1-butene (**1a**) (52.8 mg, 0.40 mmol, 2.0 equiv.), HBpin (100 µL, 0.70 mmol, 3.5 equiv.), Li_3PO_4 (50 mg, 0.40 mmol, 2.0 equiv.) and THF (0.40 mL)/DMPU (0.20 mL). The reaction mixture was stirred at 50 °C for 48 h. The crude material was purified by flash column chromatography (using 1–2% ethyl acetate in hexanes) to provide the title compound **4g** as a colorless liquid in 66% yield (46.2 mg), >95:5 r.r.. IR (neat, cm^{-1}): 2924, 2853, 1708, 1491, 1454, 1278, 1233, 749, 723, 698; ^{1}H NMR (500 MHz, $CDCl_3$): δ 7.34 – 7.31 (m, 4H), 7.26 – 7.22 (m, 1H), 3.78 (dd, *J* = 8.5, 6.5 Hz, 1H), 2.37 (t, *J* = 7.5 Hz, 2H), 2.33 – 2.28 (m, 1H), 2.26 – 2.20 (m, 1H), 1.91 – 1.78 (m, 2H), 1.68 – 1.62 (m, 2H), 1.53 – 1.43 (m, 2H), 1.39 – 1.23 (m, 15H), 0.90 (t, *J* = 7.5 Hz, 3H); ^{13}C NMR (126 MHz, $CDCl_3$): δ 179.9, 143.3, 128.5, 128.0, 127.0, 49.6, 38.9, 34.1, 31.1, 29.5, 29.5, 29.5, 29.3, 29.3, 29.2, 29.0, 24.8, 21.0, 14.0; HRMS (ESI) calcd. for $C_{21}H_{35}O_2S$ $[M+H]^+$ *m/z* 351.2352, found 351.2351.

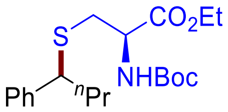

4h

ethyl *N*-(tert-butoxycarbonyl)-*S*-((*S*)-1-phenylbutyl)-*L*-cysteinate (Figure 4, **4h**): From ethyl (*tert*-butoxycarbonyl)-*L*-cysteinate (**2h**) (49.8 mg, 0.20 mmol, 1.0 equiv.), the title compound was prepared following the general procedure **B** using NiI_2 (6.4 mg, 0.020 mmol, 10.0 mol%), **L1** (8.0 mg, 0.024 mmol, 12.0 mol%), 4-phenyl-1-butene (**1a**) (52.8 mg, 0.40 mmol, 2.0 equiv.), HBpin (100 µL, 0.70 mmol, 3.5 equiv.), Li_3PO_4 (50 mg, 0.4

mmol, 2.0 equiv.) and THF (0.40 mL)/DMPU (0.20 mL). The reaction mixture was stirred at 50 °C for 48 h. The crude material was purified by flash column chromatography (using 1–2% ethyl acetate in hexanes) to provide the title compound **4h** as a colorless liquid in 70% yield (49.4 mg), 1:1 d.r. and >99:1 r.r.. IR (neat, cm^{-1}): 2978, 2932, 1737, 1493, 1343, 1161, 1126, 1025, 721, 699; ^{1}H NMR (400 MHz, $CDCl_3$): δ 7.26 – 7.19 (m, 7H), 7.18 – 7.14 (m, 3H), 5.18 – 5.16 (m, 1H) & 5.11 – 5.09 (m, 1H), 4.13 (q, *J* = 6.8 Hz, 2H) & 4.06 (q, *J* = 6.8 Hz, 2H), 3.75 (dd, *J* = 8.4, 6.8 Hz, 1H) & 3.69 (dd, *J* = 8.4, 6.8 Hz, 1H), 2.71 – 2.61 (m, 2H) & 2.61 – 2.49 (m, 2H), 1.81 – 1.65 (m, 4H) & 1.29 – 1.15 (m, 4H), 1.40 (s, 9H) & 1.36 (s, 9H), 1.21 (t, *J* = 7.0 Hz, 3H) & 1.15 (t, *J* = 7.0 Hz, 3H), 0.80 (t, *J* = 7.4 Hz, 3H) & 0.80 (t, *J* = 7.4 Hz, 3H); ^{13}C NMR (126 MHz, $CDCl_3$): δ 171.3 & 171.2, 155.4 & 155.2, 142.4 & 142.3, 128.7 & 128.6, 128.0 & 128.0, 127.4 & 127.3, 80.1 & 80.1, 61.9 & 61.7, 53.3 & 53.2, 50.2 & 49.8, 38.8 & 38.7, 33.4 & 33.4, 28.5 & 28.3, 21.0 & 20.9, 14.3 & 14.2, 13.9 & 13.9; HRMS (ESI) calcd. for $C_{20}H_{31}NO_4SNa$ $[M+Na]^+$ *m/z* 404.1866, found 404.1870.

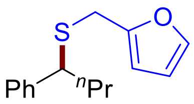

4i

2-(((1-phenylbutyl)thio)methyl)furan (Figure 4, **4i**): From furan-2-ylmethanethiol (**2i**) (22.8 mg, 0.20 mmol, 1.0 equiv.), the title compound was prepared following the general procedure **B** using NiI_2 (6.4 mg, 0.020 mmol, 10 mol%), **L1** (8.0 mg, 0.024 mmol, 12 mol%), 4-phenyl-1-butene (**1a**) (52.8 mg, 0.40 mmol, 2.0 equiv.), HBpin (100 μL, 0.70 mmol, 3.5 equiv.), Li_3PO_4 (50 mg, 0.40 mmol, 2.0 equiv.) and THF (0.40 mL)/DMPU (0.20 mL). The reaction mixture was stirred at 50 °C for 48 h. The crude material was purified by flash column chromatography (using 1–2% ethyl acetate in hexanes) to provide the title compound **4i** as a colorless liquid in 63% yield (31.0 mg), >99:1 r.r.. IR (neat, cm^{-1}): 2958, 2928, 1503, 1453, 1151, 1070, 1010, 934, 734, 699; ^{1}H NMR (500 MHz, $CDCl_3$): δ 7.37 – 7.33 (m, 5H), 7.28 – 7.25 (m, 1H), 6.31 – 6.30 (m, 1H), 6.08 – 6.07 (m, 1H), 3.78 (dd, *J* = 8.5, 6.5 Hz, 1H), 3.51 (d, *J* = 15.0 Hz, 1H), 3.41 (d, *J* = 15.0 Hz, 1H), 1.88 – 1.80 (m, 2H), 1.35 – 1.23 (m, 2H), 0.86 (t, *J* = 7.3 Hz, 3H); ^{13}C NMR

(126 MHz, $CDCl_3$): δ 152.1, 142.5, 142.1, 128.6, 128.2, 127.2, 110.4, 107.4, 49.2, 38.5, 27.6, 21.0, 13.9; HRMS (ESI) calcd. for $C_{15}H_{19}OS$ $[M+H]^+$ *m/z* 247.1151, found 247.1148.

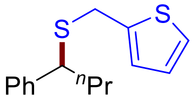

4j

2-(((1-phenylbutyl)thio)methyl)thiophene (Figure 4, **4j**): From thiophen-2-ylmethanethiol (**2j**) (26.0 mg, 0.20 mmol, 1.0 equiv.), the title compound was prepared following the general procedure **B** using NiI_2 (6.4 mg, 0.020 mmol, 10 mol%), **L1** (8.0 mg, 0.024 mmol, 12 mol%), 4-phenyl-1-butene (**1a**) (52.8 mg, 0.40 mmol, 2.0 equiv.), HBpin (100 μL, 0.70 mmol, 3.5 equiv.), Li_3PO_4 (50 mg, 0.40 mmol, 2.0 equiv.) and THF (0.40 mL)/DMPU (0.20 mL). The reaction mixture was stirred at 50 °C for 48 h. The crude material was purified by flash column chromatography (using 1–2% ethyl acetate in hexanes) to provide the title compound **4j** as a colorless liquid in 70% yield (36.7 mg), >99:1 r.r.. IR (neat, cm^{-1}): 2958, 2930, 1453, 1438, 909, 850, 733, 697; ^1H NMR (500 MHz, $CDCl_3$): δ 7.37 – 7.31 (m, 4H), 7.28 – 7.27 (m, 1H), 7.22 – 7.21 (m, 1H), 6.93 – 6.91 (m, 1H), 6.83 – 6.82 (m, 1H), 3.76 (dd, *J* = 8.5, 7.0 Hz, 1H), 3.70 (d, *J* = 14.5 Hz, 1H), 3.61 (d, *J* = 14.5 Hz, 1H), 1.89 – 1.79 (m, 2H), 1.36 – 1.23 (m, 2H), 0.86 (t, *J* = 7.5 Hz, 3H); ^{13}C NMR (126 MHz, $CDCl_3$): δ 142.6, 142.5, 128.6, 128.2, 127.2, 126.7, 126.0, 124.8, 49.1, 38.5, 29.8, 21.0, 13.9; HRMS (ESI) calcd. for $C_{15}H_{19}S_2$ $[M+H]^+$ *m/z* 263.0923, found 263.0930.

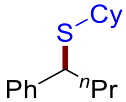

4k

cyclohexyl(1-phenylbutyl)sulfane (Figure 4, **4k**): From cyclohexanethiol (**2k**) (23.2 mg, 0.20 mmol, 1.0 equiv.), the title compound was prepared following the general procedure **B** using NiI_2 (6.4 mg, 0.020 mmol, 10 mol%), **L1** (8.0 mg, 0.024 mmol, 12 mol%), 4-phenyl-1-butene (**1a**) (52.8 mg, 0.40 mmol, 2.0 equiv.), HBpin (100 μL, 0.70 mmol, 3.5 equiv.), Li_3PO_4 (50 mg, 0.40 mmol, 2.0 equiv.) and THF (0.40 mL)/DMPU (0.20 mL).

The reaction mixture was stirred at 50 °C for 48 h. The crude material was purified by flash column chromatography (using 1–2% ethyl acetate in hexanes) to provide the title compound **4k** as a colorless liquid in 77% yield (38.3 mg), >99:1 r.r.. IR (neat, cm^{-1}): 2956, 2926, 2852, 1491, 1449, 1263, 749, 723, 698; ^{1}H NMR (500 MHz, $CDCl_3$): δ 7.33 – 7.32 (m, 4H), 7.26 – 7.22 (m, 1H), 3.88 (dd, *J* = 8.5, 6.5 Hz, 1H), 2.38 – 2.32 (m, 1H), 2.00 – 1.98 (m, 1H), 1.97 – 1.78 (m, 2H), 1.74 – 1.64 (m, 3H), 1.57 – 1.54 (m, 1H), 1.37 – 1.16 (m, 7H), 0.89 (t, *J* = 7.4 Hz, 3H); ^{13}C NMR (126 MHz, $CDCl_3$): δ 143.8, 128.4, 127.9, 126.9 , 47.9, 42.5, 39.3, 34.1, 33.4, 26.2, 26.0, 25.9, 21.0, 13.9; HRMS (ESI) calcd. for $C_{16}H_{24}SNa$ [M+Na]$^+$ *m/z* 271.1491, found 271.1493.

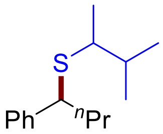

4l

(3-methylbutan-2-yl)(1-phenylbutyl)sulfane (Figure 4, **4l**): From 3-methylbutane-2-thiol (**2l**) (20.8 mg, 0.20 mmol, 1.0 equiv.), the title compound was prepared following the general procedure **B** using NiI_2 (6.4 mg, 0.020 mmol, 10 mol%), **L1** (8.0 mg, 0.024 mmol, 12 mol%), 4-phenyl-1-butene (**1a**) (52.8 mg, 0.40 mmol, 2.0 equiv.), HBpin (100 μL, 0.70 mmol, 3.5 equiv.), Li_3PO_4 (50 mg, 0.40 mmol, 2.0 equiv.) and THF (0.40 mL)/DMPU (0.20 mL). The reaction mixture was stirred at 50 °C for 48 h. The crude material was purified by flash column chromatography (using 1–2% ethyl acetate in hexanes) to provide the title compound **4l** as a colorless liquid in 89% yield (42.0 mg), 1:1 d.r. and >99:1 r.r.. IR (neat, cm^{-1}): 2958, 2930, 1491, 1452, 1374, 1068, 749, 724, 698, 541; ^{1}H NMR (500 MHz, $CDCl_3$): δ 7.35 – 7.30 (m, 8H), 7.25 – 7.22 (m, 2H), 3.80 (dd, *J* = 8.5, 6.5, 1H) & 3.78 (dd, *J* = 8.5, 6.5, 1H), 2.44 – 2.39 (m, 1H) & 2.29 – 2.24 (m, 1H), 1.89 – 1.78 (m, 4H), 1.68 – 1.63 (m, 1H), 1.39 – 1.25 (m, 5H), 1.22 (d, *J* = 7.0 Hz, 3H) & 0.97 (d, *J* = 7.0 Hz, 3H), 0.96 (d, *J* = 7.0 Hz, 3H) & 0.88 (d, *J* = 7.0 Hz, 3H), 0.90 (t, *J* = 7.5 Hz, 6H), 0.85 (d, *J* = 7.0 Hz, 3H) & 0.83 (d, *J* = 6.5 Hz, 3H); ^{13}C NMR (126 MHz, $CDCl_3$): δ 143.8 & 143.6, 128.4 & 128.4, 128.0 & 128.0, 126.9, 49.4 & 48.5, 45.7 & 45.6, 39.4 & 39.2, 33.2 & 32.7, 21.1 & 21.0, 19.9 & 19.6, 19.3 & 18.5, 18.0 & 17.2, 14.0;

HRMS (ESI) calcd. for $C_{15}H_{25}S$ $[M+H]^+$ *m/z* 237.1671, found 237.1677.

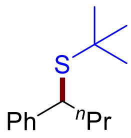
4m

***tert*-butyl(1-phenylbutyl)sulfane** (Figure 4, **4m**): From 1,2-di-*tert*-butyldisulfane (**2m**) (17.8 mg, 0.10 mmol, 0.5 equiv.), the title compound was prepared following the general procedure **B** using NiI_2 (6.4 mg, 0.020 mmol, 10 mol%), **L1** (8.0 mg, 0.024 mmol, 12 mol%), 4-phenyl-1-butene (**1a**) (52.8 mg, 0.40 mmol, 2.0 equiv.), HBpin (100 μL, 0.70 mmol, 3.5 equiv.), Li_3PO_4 (50 mg, 0.40 mmol, 2.0 equiv.) and THF (0.40 mL)/DMPU (0.20 mL). The reaction mixture was stirred at 50 °C for 48 h. The crude material was purified by flash column chromatography (using 1–2% ethyl acetate in hexanes) to provide the title compound **4m** as a colorless liquid in 90% yield (43.9 mg), >99:1 r.r.. IR (neat, cm^{-1}): 2958, 2925, 2863, 1491, 1453, 1363, 1162, 749, 723, 698; ^1H NMR (500 MHz, $CDCl_3$): δ 7.35 – 7.30 (m, 4H), 7.23 – 7.17 (m, 1H), 3.84 (dd, *J* = 8.5, 6.5 Hz, 1H), 1.87 – 1.74 (m, 2H), 1.38 – 1.26 (m, 2H), 1.22 (s, 9H), 0.89 (t, *J* = 7.5 Hz, 3H); ^{13}C NMR (126 MHz, $CDCl_3$): δ 145.7, 128.4, 127.8, 126.6, 47.7, 43.8, 41.1, 31.6, 21.0, 13.9; HRMS (ESI) calcd. for $C_{14}H_{22}S$ $[M+H]^+$ *m/z* 245.1334, found 245.1335.

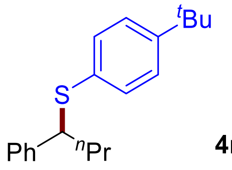
4n

(4-(*tert*-butyl)phenyl)(1-phenylbutyl)sulfane (Figure 4, **4n**): From 4-(*tert*-butyl)benzenethiol (**2n**) (33.2 mg, 0.20 mmol, 1.0 equiv.), the title compound was prepared following the general procedure **B** using NiI_2 (6.4 mg, 0.020 mmol, 10 mol%), **L1** (8.0 mg, 0.024 mmol, 12 mol%), 4-phenyl-1-butene (**1a**) (52.8 mg, 0.40 mmol, 2.0 equiv.), HBpin (100 μL, 0.70 mmol, 3.5 equiv.), Li_3PO_4 (50 mg, 0.40 mmol, 2.0 equiv.) and THF (0.40 mL)/DMPU (0.20 mL). The reaction mixture was stirred at 50 °C for 48 h. The crude material was purified by flash column chromatography (using 1–2% ethyl

acetate in hexanes) to provide the title compound **4n** as a colorless liquid in 95% yield (56.8 mg), >99:1 r.r.. IR (neat, cm^{-1}): 2959, 2933, 2870, 1490, 1438, 1120, 1014, 829, 747, 697; ^{1}H NMR (500 MHz, $CDCl_3$): δ 7.30 – 7.25 (m, 4H), 7.25 – 7.22 (m, 3H), 7.22 – 7.19 (m, 2H), 4.12 (dd, J = 8.5, 6.5 Hz, 1H), 2.01 – 1.88 (m, 2H), 1.42 – 1.32 (m, 2H), 1.30 (s, 9H), 0.90 (t, J = 7.3 Hz, 3H); ^{13}C NMR (126 MHz, $CDCl_3$): δ 150.3, 142.6, 132.2, 131.8, 128.4, 127.9, 127.0, 125.8, 53.6, 38.6, 34.6, 31.4, 20.9, 13.9; HRMS (ESI) calcd. for $C_{20}H_{26}SNa$ $[M+Na]^+$ m/z 321.1647, found 321.1646.

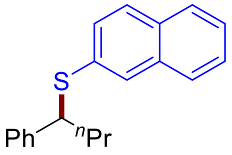

4o

naphthalen-2-yl(1-phenylbutyl)sulfane (Figure 4, **4o**): From naphthalene-2-thiol (**2o**) (32.0 mg, 0.20 mmol, 1.0 equiv.), the title compound was prepared following the general procedure **B** using NiI_2 (6.4 mg, 0.020 mmol, 10 mol%), **L1** (8.0 mg, 0.024 mmol, 12 mol%), 4-phenyl-1-butene (**1a**) (52.8 mg, 0.40 mmol, 2.0 equiv.), HBpin (100 µL, 0.70 mmol, 3.5 equiv.), Li_3PO_4 (50 mg, 0.40 mmol, 2.0 equiv.) and THF (0.40 mL)/DMPU (0.20 mL). The reaction mixture was stirred at 50 °C for 48 h. The crude material was purified by flash column chromatography (using 1–2% ethyl acetate in hexanes) to provide the title compound **4o** as a colorless liquid in 95% yield (55.5 mg), >99:1 r.r.. IR (neat, cm^{-1}): 2957, 2929, 1585, 1452, 1264, 905, 811, 740, 723, 696, 473; ^{1}H NMR (500 MHz, $CDCl_3$): δ 7.79 – 7.77 (m, 2H), 7.73 – 7.69 (m, 3H), 7.48 – 7.44 (m, 2H), 7.37 – 7.35 (m, 1H), 7.32 – 7.26 (m, 4H), 7.23 – 7.20 (m, 1H), 4.29 (dd, J = 8.5, 6.5 Hz, 1H), 2.06 – 1.93 (m, 2H), 1.46 – 1.34 (m, 2H), 0.92 (t, J = 7.3 Hz, 3H); ^{13}C NMR (126 MHz, $CDCl_3$): δ 142.3, 133.7, 132.8, 132.3, 131.0, 129.8, 128.5, 128.2, 128.0, 127.7, 127.5, 127.2, 126.4, 126.1, 53.4, 38.6, 21.0, 13.9; HRMS (ESI) calcd. for $C_{20}H_{21}S$ $[M+H]^+$ m/z 293.1358, found 293.1358.

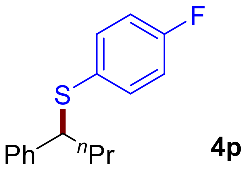

4p

(4-fluorophenyl)(1-phenylbutyl)sulfane (Figure 4, **4p**): From 4-fluorobenzenethiol (**2p**) (25.6 mg, 0.20 mmol, 1.0 equiv.), the title compound was prepared following the general procedure **B** using NiI_2 (6.4 mg, 0.020 mmol, 10 mol%), **L1** (8.0 mg, 0.024 mmol, 12 mol%), 4-phenyl-1-butene (**1a**) (52.8 mg, 0.40 mmol, 2.0 equiv.), HBpin (100 μL, 0.70 mmol, 3.5 equiv.), Li_3PO_4 (50 mg, 0.40 mmol, 2.0 equiv.) and THF (0.40 mL)/DMPU (0.20 mL). The reaction mixture was stirred at 50 °C for 48 h. The crude material was purified by flash column chromatography (using 1–2% ethyl acetate in hexanes) to provide the title compound **4p** as a colorless liquid in 83% yield (43.2 mg), >99:1 r.r.. IR (neat, cm^{-1}): 2958, 2931, 1589, 1488, 1453, 1225, 1155, 830, 748, 697; ^1H NMR (500 MHz, $CDCl_3$): δ 7.28 – 7.25 (m, 2H), 7.22 – 7.17 (m, 5H), 6.91 – 6.88 (m, 2H), 4.04 (dd, J = 8.5, 6.5 Hz, 1H), 1.97 – 1.90 (m, 2H), 1.42 – 1.31 (m, 2H), 0.91 (t, J = 7.5 Hz, 3H); ^{13}C NMR (126 MHz, $CDCl_3$): δ 162.5 (d, $J_{C\text{-}F}$ = 245.0 Hz), 142.2, 135.7 (d, $J_{C\text{-}F}$ = 8.8 Hz), 129.8 (d, $J_{C\text{-}F}$ = 3.8 Hz), 128.4, 128.0, 127.2, 115.8 (d, $J_{C\text{-}F}$ = 21.4 Hz), 54.5, 38.0, 20.9, 13.9; ^{19}F NMR (471 MHz, $CDCl_3$): δ -114.2; HRMS (ESI) calcd. for $C_{16}H_{18}FS$ $[M+H]^+$ *m/z* 261.1108, found 261.1121.

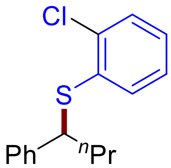

4q

(2-chlorophenyl)(1-phenylbutyl)sulfane (Figure 4, **4q**): From 2-chlorobenzenethiol (**2q**) (28.8 mg, 0.20 mmol, 1.0 equiv.), the title compound was prepared following the general procedure **B** using NiI_2 (6.4 mg, 0.020 mmol, 10 mol%), **L1** (8.0 mg, 0.024 mmol, 12 mol%), 4-phenyl-1-butene (**1a**) (52.8 mg L, 0.40 mmol, 2.0 equiv.), HBpin (100 μL, 0.70 mmol, 3.5 equiv.), Li_3PO_4 (50 mg, 0.40 mmol, 2.0 equiv.) and THF (0.40 mL)/DMPU (0.20 mL). The reaction mixture was stirred at 50 °C for 48 h. The crude material was

purified by flash column chromatography (using 1–2% ethyl acetate in hexanes) to provide the title compound **4q** as a colorless liquid in 76% yield (42.0 mg), >99:1 r.r.. IR (neat, cm^{-1}): 2958, 2931, 2871, 1492, 1450, 1432, 1116, 1036, 744, 698; ^{1}H NMR (500 MHz, $CDCl_3$): δ 7.36 – 7.35 (m, 1H), 7.32 – 7.31 (m, 2H), 7.29 – 7.26 (m, 2H), 7.22 – 7.19 (m, 2H), 7.12 – 7.04 (m, 2H), 4.33 (dd, J = 8.0, 6.5 Hz, 1H), 2.01 – 1.95 (m, 2H), 1.45 – 1.34 (m, 2H), 0.92 (t, J = 7.3 Hz, 3H); ^{13}C NMR (126 MHz, $CDCl_3$): δ 141.8, 135.8, 134.7, 132.6, 129.8, 128.5, 127.9, 127.7, 127.3, 126.9, 51.7, 38.6, 20.9, 13.9; HRMS (ESI) calcd. for $C_{16}H_{18}ClS$ $[M+H]^+$ m/z 277.0812, found 277.0809.

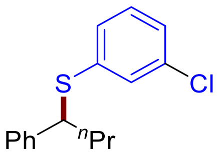

4r

(3-chlorophenyl)(1-phenylbutyl)sulfane (Figure 4, **4r**): From 3-chlorobenzenethiol (**2r**) (28.8 mg, 0.20 mmol, 1.0 equiv.), the title compound was prepared following the general procedure **B** using NiI_2 (6.4 mg, 0.020 mmol, 10 mol%), **L1** (8.0 mg, 0.024 mmol, 12 mol%), 4-phenyl-1-butene (**1a**) (52.8 mg, 0.40 mmol, 2.0 equiv.), HBpin (100 μL, 0.70 mmol, 3.5 equiv.), Li_3PO_4 (50 mg, 0.40 mmol, 2.0 equiv.) and THF (0.40 mL)/DMPU (0.20 mL). The reaction mixture was stirred at 50 °C for 48 h. The crude material was purified by flash column chromatography (using 1–2% ethyl acetate in hexanes) to provide the title compound **4r** as a colorless liquid in 77% yield (42.5 mg), >99:1 r.r.. IR (neat, cm^{-1}): 2958, 2931, 1576, 1491, 1460, 1397, 779, 748, 697, 680; ^{1}H NMR (500 MHz, $CDCl_3$): δ 7.31 – 7.22 (m, 6H), 7.16 – 7.10 (m, 3H), 4.18 (dd, J = 8.5, 6.5 Hz, 1H), 2.00 – 1.88 (m, 2H), 1.43 – 1.30 (m, 2H), 0.92 (t, J = 7.5 Hz, 3H); ^{13}C NMR (126 MHz, $CDCl_3$): δ 141.9, 137.5, 134.3, 131.5, 129.9, 129.7, 128.5, 127.9, 127.4, 127.0, 53.3, 38.5, 20.9, 13.9; HRMS (ESI) calcd. for $C_{16}H_{18}ClS$ $[M+H]^+$ m/z 277.0812, found 277.0813.

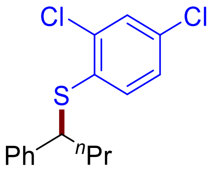

4s

(2,4-dichlorophenyl)(1-phenylbutyl)sulfane (Figure 4, **4s**): From 2,4-dichlorobenzenethiol (**2s**) (35.6 mg, 0.20 mmol, 1.0 equiv.), the title compound was prepared following the general procedure **B** using NiI_2 (6.4 mg, 0.020 mmol, 10 mol%), **L1** (8.0 mg, 0.024 mmol, 12 mol%), 4-phenyl-1-butene (**1a**) (52.8 mg, 0.40 mmol, 2.0 equiv.), HBpin (100 μL, 0.70 mmol, 3.5 equiv.), Li_3PO_4 (50 mg, 0.40 mmol, 2.0 equiv.) and THF (0.40 mL)/DMPU (0.20 mL). The reaction mixture was stirred at 50 ^{o}C for 48 h. The crude material was purified by flash column chromatography (using 1–2% ethyl acetate in hexanes) to provide the title compound **4s** as a colorless liquid in 68% yield (42.2 mg), >99:1 r.r.. IR (neat, cm^{-1}): 2958, 2932, 1568, 1452, 1369, 1096, 1034, 866, 811, 698; ^{1}H NMR (500 MHz, $CDCl_3$): δ 7.37 (d, J = 2.0 Hz, 1H), 7.28 – 7.27 (m, 4H), 7.23 – 7.20 (m, 1H), 7.10 – 7.08 (m, 1H), 7.04 – 7.02 (m, 1H), 4.28 (dd, J = 8.0, 7.0 Hz, 1H), 1.99 – 1.93 (m, 2H), 1.44 – 1.34 (m, 2H), 0.93 (t, J = 7.3 Hz, 3H); ^{13}C NMR (126 MHz, $CDCl_3$): δ 141.5, 136.8, 133.7, 133.3, 133.1, 129.6, 128.6, 127.9, 127.4, 127.2, 52.1, 38.5, 20.9, 13.9; HRMS (ESI) calcd. for $C_{16}H_{17}Cl_2S$ $[M+H]^+$ m/z 311.0423, found 311.0412.

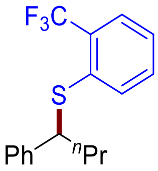

4t

(1-phenylbutyl)(2-(trifluoromethyl)phenyl)sulfane (Figure 4, **4t**): From 2-(trifluoromethyl)benzenethiol (**2t**) (35.6 mg, 0.20 mmol, 1.0 equiv.), the title compound was prepared following the general procedure **B** using NiI_2 (6.4 mg, 0.020 mmol, 10 mol%), **L1** (8.0 mg, 0.024 mmol, 12 mol%), 4-phenyl-1-butene (**1a**) (52.8 mg, 0.40 mmol, 2.0 equiv.), HBpin (100 μL, 0.70 mmol, 3.5 equiv.), Li_3PO_4 (50 mg, 0.40 mmol, 2.0 equiv.) and THF (0.40 mL)/DMPU (0.20 mL). The reaction mixture was stirred at 50

°C for 48 h. The crude material was purified by flash column chromatography (using 1–2% ethyl acetate in hexanes) to provide the title compound **4t** as a colorless liquid in 75% yield (46.5 mg), >99:1 r.r.. IR (neat, cm^{-1}): 2956, 2931, 1604, 1464, 1432, 905, 746, 740, 696; ^{1}H NMR (500 MHz, $CDCl_3$): δ 7.63 – 7.61 (m, 1H), 7.32 – 7.25 (m, 7H), 7.24 – 7.19 (m, 1H), 4.25 (dd, J = 8.5, 6.5 Hz, 1H), 2.01 – 1.92 (m, 2H), 1.42 – 1.31 (m, 2H), 0.91 (t, J = 7.3 Hz, 3H); ^{13}C NMR (126 MHz, $CDCl_3$): δ 141.7, 135.0, 134.4 (2C), 131.6, 128.5, 128.1, 127.4, 126.8 (q, J_{C-F} = 6.3 Hz), 126.7, 123.9 (q, J_{C-F} = 273.4 Hz), 53.8, 38.6, 20.9, 13.9; ^{19}F NMR (471 MHz, $CDCl_3$): δ -60.3; HRMS (ESI) calcd. for $C_{17}H_{18}F_3S$ $[M+H]^+$ m/z 311.1076, found 311.1073.

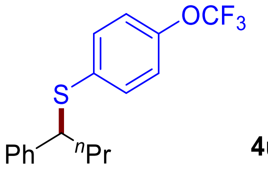

4u

(1-phenylbutyl)(4-(trifluoromethoxy)phenyl)sulfane (Figure 4, **4u**): From 4-(trifluoromethoxy)benzenethiol (**2u**) (38.8 mg, 0.20 mmol, 1.0 equiv.), the title compound was prepared following the general procedure **B** using NiI_2 (6.4 mg, 0.020 mmol, 10 mol%), **L1** (8.0 mg, 0.024 mmol, 12 mol%), 4-phenyl-1-butene (**1a**) (52.8 mg, 0.40 mmol, 2.0 equiv.), HBpin (100 μL, 0.70 mmol, 3.5 equiv.), Li_3PO_4 (50 mg, 0.40 mmol, 2.0 equiv.) and THF (0.40 mL)/DMPU (0.20 mL). The reaction mixture was stirred at 50 °C for 48 h. The crude material was purified by flash column chromatography (using 1–2% ethyl acetate in hexanes) to provide the title compound **4u** as a colorless liquid in 88% yield (57.4 mg), >99:1 r.r.. IR (neat, cm^{-1}): 2960, 2933, 1489, 1252, 1205, 1162, 1091, 1017, 698; ^{1}H NMR (500 MHz, $CDCl_3$): δ 7.29 – 7.26 (m, 2H), 7.25 – 7.21 (m, 5H), 7.05 – 7.03 (m, 2H), 4.12 (dd, J = 9.0, 6.5 Hz, 1H), 1.99 – 1.89 (m, 2H), 1.43 – 1.31 (m, 2H), 0.92 (t, J = 7.5 Hz, 3H); ^{13}C NMR (126 MHz, $CDCl_3$): δ 148.4, 142.0, 133.9, 133.9, 128.5, 127.9, 127.3, 121.2, 120.5 (q, J_{C-F} = 259.6 Hz) 53.9, 38.4, 20.9, 13.9; ^{19}F NMR (471 MHz, $CDCl_3$): δ -57.9; HRMS (ESI) calcd. for $C_{17}H_{18}F_3OS$ $[M+H]^+$ m/z 327.1025, found 327.1024.

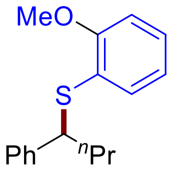

4v

(2-methoxyphenyl)(1-phenylbutyl)sulfane (Figure 4, **4v**): From 2-methoxybenzenethiol (**2v**) (28.0 mg, 0.20 mmol, 1.0 equiv.), the title compound was prepared following the general procedure **B** using NiI_2 (6.4 mg, 0.020 mmol, 10 mol%), **L1** (8.0 mg, 0.024 mmol, 12 mol%), 4-phenyl-1-butene (**1a**) (52.8 mg, 0.40 mmol, 2.0 equiv.), HBpin (100 µL, 0.70 mmol, 3.5 equiv.), Li_3PO_4 (50 mg, 0.40 mmol, 2.0 equiv.) and THF (0.40 mL)/DMPU (0.20 mL). The reaction mixture was stirred at 50 ^oC for 48 h. The crude material was purified by flash column chromatography (using 1–2% ethyl acetate in hexanes) to provide the title compound **4v** as a colorless liquid in 95% yield (51.7 mg), >99:1 r.r.. IR (neat, cm^{-1}): 2956, 2931, 1580, 1474, 1432, 1271, 1241, 1070, 1025, 746, 697; ^1H NMR (500 MHz, $CDCl_3$): δ 7.29 – 7.23 (m, 4H), 7.19 – 7.16 (m, 3H), 6.82 – 6.78 (m, 2H), 4.31 (dd, J = 8.5, 6.5 Hz, 1H), 3.87 (s, 3H), 2.01 – 1.91 (m, 2H), 1.42 – 1.30 (m, 2H), 0.91 (t, J = 7.3 Hz, 3H); ^{13}C NMR (126 MHz, $CDCl_3$): δ 158.5, 142.4, 133.1, 128.3, 128.3, 127.9, 127.0, 123.4, 120.8, 110.6, 55.8, 50.9, 38.6, 20.9, 13.9; HRMS (ESI) calcd. for $C_{17}H_{21}OS$ $[M+H]^+$ m/z 273.1308, found 273.1312.

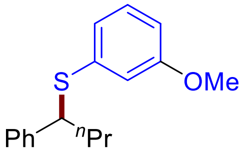

4w

(3-methoxyphenyl)(1-phenylbutyl)sulfane (Figure 4, **4w**): From 3-methoxybenzenethiol (**2w**) (28.0 mg, 0.20 mmol, 1.0 equiv.), the title compound was prepared following the general procedure **B** using NiI_2 (6.4 mg, 0.020 mmol, 10 mol%), **L1** (8.0 mg, 0.024 mmol, 12 mol%), 4-phenyl-1-butene (**1a**) (52.8 mg, 0.40 mmol, 2.0 equiv.), HBpin (100 µL, 0.70 mmol, 3.5 equiv.), Li_3PO_4 (50 mg, 0.40 mmol, 2.0 equiv.) and THF (0.40 mL)/DMPU (0.20 mL). The reaction mixture was stirred at 50 ^oC for 48 h. The crude material was purified by flash column chromatography (using 1–2% ethyl acetate in hexanes) to provide the title compound **4w** as a colorless liquid in 98% yield

(53.3 mg), >99:1 r.r.. IR (neat, cm^{-1}): 2958, 2932, 1587, 1574, 1477, 1262, 1038, 905, 724, 697; ^{1}H NMR (500 MHz, $CDCl_3$): δ 7.31 – 7.27 (m, 4H), 7.24 – 7.20 (m, 1H), 7.15 – 7.12 (m, 1H), 6.89 – 6.87 (m, 1H), 6.76 – 6.72 (m, 2H), 4.18 (dd, *J* = 8.5, 6.5 Hz, 1H), 3.70 (s, 3H), 2.02 – 1.88 (m, 2H), 1.43 – 1.30 (m, 2H), 0.91 (t, *J* = 7.3 Hz, 3H); ^{13}C NMR (126 MHz, $CDCl_3$): δ 159.6, 142.5, 136.6, 129.5, 128.5, 128.0, 127.2, 124.3, 116.9, 113.2, 55.3, 53.3, 38.6, 20.9, 13.9; HRMS (ESI) calcd. for $C_{17}H_{21}OS$ $[M+H]^+$ *m/z* 273.1308, found 273.1312.

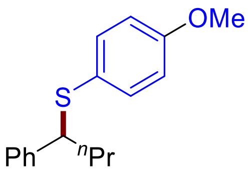

4x

(4-methoxyphenyl)(1-phenylbutyl)sulfane (Figure 4, **4x**): From 4-methoxybenzenethiol (**2x**) (28.0 mg, 0.20 mmol, 1.0 equiv.), the title compound was prepared following the general procedure **B** using NiI_2 (6.4 mg, 0.020 mmol, 10 mol%), **L1** (8.0 mg, 0.024 mmol, 12 mol%), 4-phenyl-1-butene (**1a**) (52.8 mg, 0.40 mmol, 2.0 equiv.), HBpin (100 μL, 0.70 mmol, 3.5 equiv.), Li_3PO_4 (50 mg, 0.40 mmol, 2.0 equiv.) and THF (0.40 mL)/DMPU (0.20 mL). The reaction mixture was stirred at 50 °C for 48 h. The crude material was purified by flash column chromatography (using 1–2% ethyl acetate in hexanes) to provide the title compound **4x** as a colorless liquid in 90% yield (49.0 mg), >99:1 r.r.. IR (neat, cm^{-1}): 2957, 2931, 1591, 1492, 1284, 1243, 1171, 1030, 826, 697; ^{1}H NMR (500 MHz, $CDCl_3$): δ 7.28 – 7.25 (m, 2H), 7.22 – 7.16 (m, 5H), 6.77 – 6.74 (m, 2H), 3.98 (dd, *J* = 8.5, 6.5 Hz, 1H), 3.79 (s, 3H), 1.97 – 1.88 (m, 2H), 1.40 – 1.30 (m, 2H), 0.90 (t, *J* = 7.5 Hz, 3H); ^{13}C NMR (126 MHz, $CDCl_3$): δ 159.6, 142.6, 136.0, 128.3, 128.0, 127.0, 125.2, 114.2, 55.4, 54.8, 37.9, 21.0, 13.9; HRMS (ESI) calcd. for $C_{17}H_{21}OS$ $[M+H]^+$ *m/z* 273.1308, found 273.1312.

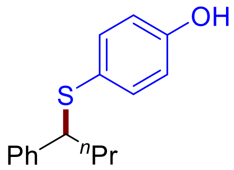

4y

4-((1-phenylbutyl)thio)phenol (Figure 4, **4y**): From 4-mercaptophenol (**2y**) (25.2 mg, 0.20 mmol, 1.0 equiv.), the title compound was prepared following the general procedure **B** using NiI_2 (6.4 mg, 0.020 mmol, 10 mol%), **L1** (8.0 mg, 0.024 mmol, 12 mol%), 4-phenyl-1-butene (**1a**) (52.8 mg, 0.40 mmol, 2.0 equiv.), HBpin (100 µL, 0.70 mmol, 3.5 equiv.), Li_3PO_4 (50 mg, 0.40 mmol, 2.0 equiv.) and THF (0.40 mL)/DMPU (0.20 mL). The reaction mixture was stirred at 50 °C for 48 h. The crude material was purified by flash column chromatography (using 1–2% ethyl acetate in hexanes) to provide the title compound **4y** as a colorless liquid in 88% yield (45.4 mg), >99:1 r.r.. IR (neat, cm^{-1}): 2957, 2930, 1582, 1492, 1263, 1214, 1168, 906, 829, 722, 698; ^{1}H NMR (500 MHz, $CDCl_3$): δ 7.28 – 7.24 (m, 2H), 7.22 – 7.19 (m, 1H), 7.17 – 7.12 (m, 4H), 6.69 – 6.66 (m, 2H), 3.98 (dd, *J* = 8.5, 6.5 Hz, 1H), 1.96 – 1.89 (m, 2H), 1.38 – 1.30 (m, 2H), 0.90 (t, *J* = 7.3 Hz, 3H); ^{13}C NMR (126 MHz, $CDCl_3$): δ 155.6, 142.5, 136.3, 128.3, 128.0, 127.0, 125.3, 115.8, 54.8, 37.9, 21.0, 13.9; HRMS (ESI) calcd. for $C_{16}H_{19}OS$ $[M+H]^+$ *m/z* 259.1151, found 259.1154.

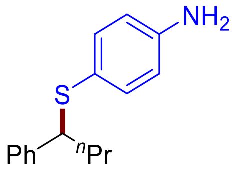

4z

4-((1-phenylbutyl)thio)aniline (Figure 4, **4z**): From 4-aminobenzenethiol (**2z**) (25.0 mg, 0.20 mmol, 1.0 equiv.), the title compound was prepared following the general procedure **B** using NiI_2 (6.4 mg, 0.020 mmol, 10 mol%), **L1** (8.0 mg, 0.024 mmol, 12 mol%), 4-phenyl-1-butene (**1a**) (52.8 mg, 0.40 mmol, 2.0 equiv.), HBpin (100 µL, 0.70 mmol, 3.5 equiv.), Li_3PO_4 (50 mg, 0.40 mmol, 2.0 equiv.) and THF (0.40 mL)/DMPU (0.20 mL). The reaction mixture was stirred at 50 °C for 48 h. The crude material was purified by flash column chromatography (using 1–2% ethyl acetate in hexanes) to provide the title

compound **4z** as a colorless liquid in 92% yield (47.3 mg), >99:1 r.r.. IR (neat, cm^{-1}): 2956, 2929, 1619, 1596, 1492, 1281, 1176, 823, 697, 513; ^{1}H NMR (500 MHz, $CDCl_3$): δ 7.28 – 7.24 (m, 2H), 7.21 – 7.16 (m, 3H), 7.07 – 7.06 (m, 2H), 6.57 – 6.56 (m, 2H), 3.93 (dd, J = 8.5, 6.5 Hz, 1H), 3.29 (brs, 2H), 1.96 – 1.86 (m, 2H), 1.39 – 1.29 (m, 2H), 0.89 (t, J = 7.3 Hz, 3H); ^{13}C NMR (126 MHz, $CDCl_3$): δ 145.7, 142.7, 136.3, 128.3, 128.0, 126.9, 122.8, 115.7, 54.8, 37.8, 21.0, 13.9; HRMS (ESI) calcd. for $C_{16}H_{20}NS$ $[M+H]^+$ *m/z* 258.1311, found 258.1313.

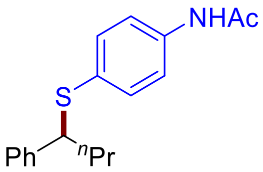

4a'

***N*-(4-((1-phenylbutyl)thio)phenyl)acetamide** (Figure 4, **4a'**): From *N*-(4-mercaptophenyl)acetamide (**2a'**) (33.4 mg, 0.20 mmol, 1.0 equiv.), the title compound was prepared following the general procedure **B** using NiI_2 (6.4 mg, 0.020 mmol, 10 mol%), **L1** (8.0 mg, 0.024 mmol, 12 mol%), 4-phenyl-1-butene (**1a**) (52.8 mg, 0.40 mmol, 2.0 equiv.), HBpin (100 μL, 0.70 mmol, 3.5 equiv.), Li_3PO_4 (50 mg, 0.40 mmol, 2.0 equiv.) and THF (0.40 mL)/DMPU (0.20 mL). The reaction mixture was stirred at 50 ^{o}C for 48 h. The crude material was purified by flash column chromatography (using 1–2% ethyl acetate in hexanes) to provide the title compound **4a'** as a colorless liquid in 94% yield (56.2 mg), >99:1 r.r.. IR (neat, cm^{-1}): 2957, 2930, 1662, 1590, 1525, 1491, 1393, 1310, 825, 747, 697; ^{1}H NMR (500 MHz, $CDCl_3$): δ 7.37 – 7.35 (m, 3H), 7.27 – 7.24 (m, 2H), 7.21 – 7.19 (m, 4H), 4.07 (dd, J = 9.0, 6.5 Hz, 1H), 2.16 (s, 3H), 1.97 – 1.87 (m, 2H), 1.39 – 1.29 (m, 2H), 0.90 (t, J = 7.5 Hz, 3H); ^{13}C NMR (126 MHz, $CDCl_3$): δ 168.5, 142.3, 137.4, 134.2, 130.0, 128.4, 127.9, 127.1, 119.9, 54.1, 38.2, 24.8, 20.9, 13.9; HRMS (ESI) calcd. for $C_{18}H_{22}NOS$ $[M+H]^+$ *m/z* 300.1417, found 300.1419.

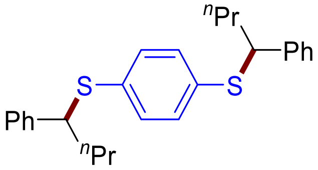

4b'

1,4-bis((1-phenylbutyl)thio)benzene (Figure 4, **4b'**): From benzene-1,4-dithiol (**2b'**) (14.2 mg, 0.1 mmol, 0.5 equiv.), the title compound was prepared following the general procedure **B** using NiI_2 (6.4 mg, 0.020 mmol, 10 mol%), **L1** (8.0 mg, 0.024 mmol, 12 mol%), 4-phenyl-1-butene (**1a**) (52.8 mg, 0.40 mmol, 2.0 equiv.), HBPin (100 μL, 0.70 mmol, 3.5 equiv.), Li_3PO_4 (50 mg, 0.40 mmol, 2.0 equiv.) and THF (0.40 mL)/DMPU (0.20 mL). The reaction mixture was stirred at 50 °C for 48 h. The crude material was purified by flash column chromatography (using 1–2% ethyl acetate in hexanes) to provide the title compound **4b'** as a colorless liquid in 76% yield (30.9 mg), >99:1 r.r.. IR (neat, cm^{-1}): 2958, 2932, 1575, 1483, 1397, 1038, 905, 748, 697; ^1H NMR (500 MHz, $CDCl_3$): δ 7.27 – 7.24 (m, 4H), 7.22 – 7.19 (m, 6H), 7.05 – 7.04 (m, 4H), 4.09 (dd, *J* = 8.5, 6.5 Hz, 2H), 1.96 – 1.84 (m, 4H), 1.39 – 1.28 (m, 4H), 0.90 (t, *J* = 7.3 Hz, 6H); ^{13}C NMR (126 MHz, $CDCl_3$): δ 142.2 (2C), 134.0 (2C), 132.6 (2C), 132.5 (2C), 128.4 (4C), 127.9 (4C), 127.2 (2C), 53.5 (2C), 38.3 (2C), 20.9 (2C), 13.9 (2C); HRMS (ESI) calcd. for $C_{26}H_{31}S_2$ $[M+H]^+$ *m/z* 407.1862, found 407.1861.

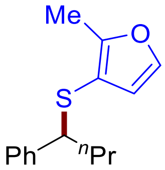

4c'

2-methyl-3-((1-phenylbutyl)thio)furan (Figure 4, **4c'**): From 2-methylfuran-3-thiol (**2c'**) (22.8 mg, 0.20 mmol, 1.0 equiv.), the title compound was prepared following the general procedure **B** using NiI_2 (6.4 mg, 0.020 mmol, 10 mol%), **L1** (8.0 mg, 0.024 mmol, 12 mol%), 4-phenyl-1-butene (**1a**) (52.8 mg, 0.40 mmol, 2.0 equiv.), HBpin (100 μL, 0.70 mmol, 3.5 equiv.), Li_3PO_4 (50 mg, 0.40 mmol, 2.0 equiv.) and THF (0.40 mL)/DMPU (0.20 mL). The reaction mixture was stirred at 50 °C for 48 h. The crude material was purified by flash column chromatography (using 1–2% ethyl acetate in hexanes) to

provide the title compound **4c'** as a colorless liquid in 78% yield (38.4 mg), >99:1 r.r.. IR (neat, cm^{-1}): 2958, 2931, 1514, 1453, 1223, 1126, 1088, 888, 731, 697; ^{1}H NMR (500 MHz, $CDCl_3$): δ 7.28 – 7.21 (m, 4H), 7.13 – 7.11 (m, 2H), 6.12 – 6.11 (m, 1H), 3.80 (dd, *J* = 8.0, 7.0 Hz, 1H), 1.95 (s, 3H), 1.94 – 1.90 (m, 2H), 1.44 – 1.32 (m, 2H), 0.92 (t, *J* = 7.5 Hz, 3H); ^{13}C NMR (126 MHz, $CDCl_3$): δ 156.8, 142.5, 140.2, 128.2, 127.9, 127.0, 116.0, 109.0, 53.7, 37.1, 21.0, 13.9, 11.4; HRMS (ESI) calcd. for $C_{15}H_{19}OS$ $[M+H]^+$ *m/z* 247.1151, found 247.1148.

Supplementary Discussion

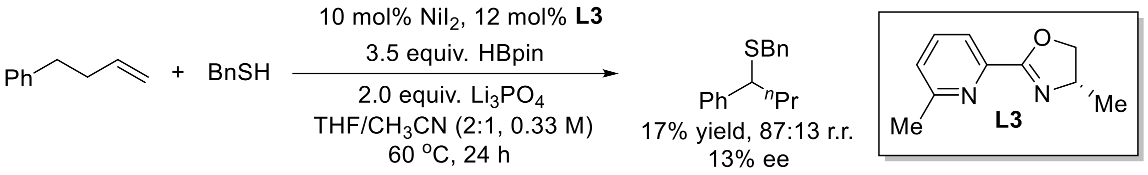

Supplementary Figure 2. Preliminary results of asymmetric reaction, Related to Figure 2.

Supplementary Note 1. Currently, using a chiral pyrox ligand (**L3**) has demonstrated a not excellent level of enantioselective (13% ee). Progress in this area will be reported in due course.

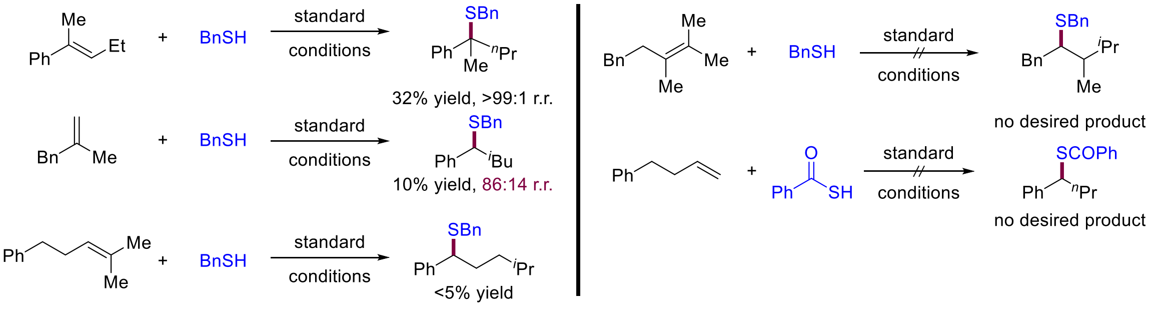

Supplementary Figure 3. Unsuccessful substrates, Related to Figure 3.

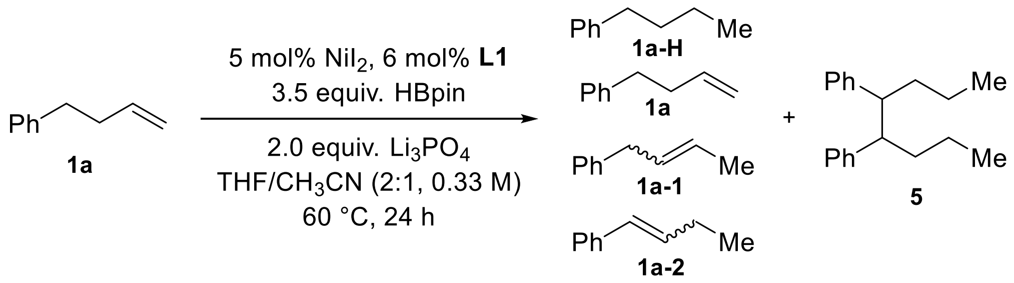

entry	time (h)	**1a-H** (GC yield/%)	**1a** (GC yield/%)	**1a-1** (GC yield/%)	**1a-2** (GC yield/%)	**5** (GC yield/%)
1	0.5	3.8	71.0	4.2	17.5	2.0
2	1	4.3	49.5	7.2	30.7	4.0
3	2	4.2	44.2	7.4	32.6	6.0
4	4	4.1	40.8	8.0	35.0	6.0
5	16	4.5	26.4	9.6	41.4	9.0
6	24	3.8	15.9	10.0	47.3	11.0
7	48	4.1	12.8	10.2	48.3	13.0

Supplementary Figure 4. Olefin isomerization in the absence of thiol (in N_2 atmosphere), Related to Figure 5a.

Supplementary Note 2. A significant amount of other olefin isomers arising from the olefin isomerization is observed within 1 h, which indicates that occurrence of olefin isomerization does not depend on the presence of the thiol and also suggests that olefin isomerization is unrelated to C–S coupling. We propose that the compound 5 is formed through the homocoupling of two benzylic radicals.

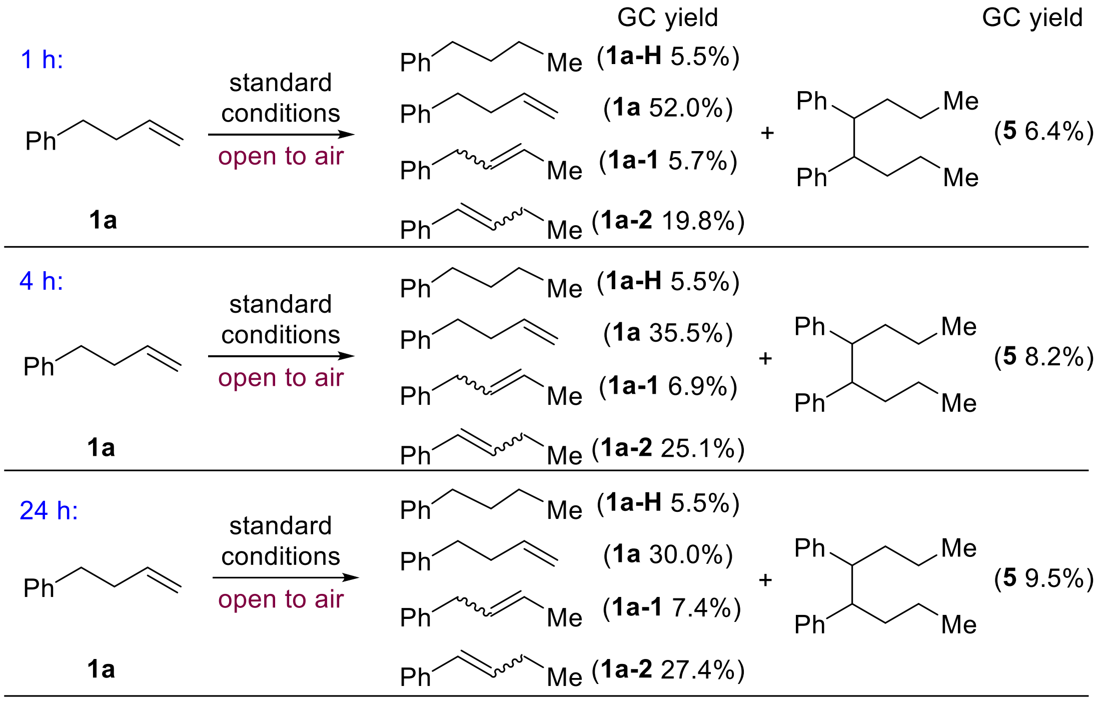

Supplementary Figure 5. Olefin isomerization in the absence of thiol (open to air), Related to Figure 5a.

Supplementary Note 3. Alkene isomerization (a mixture of olefins is observed) could still proceed when the reaction system is open to air. This results indicate that alkene isomerization is not sensitive to air.

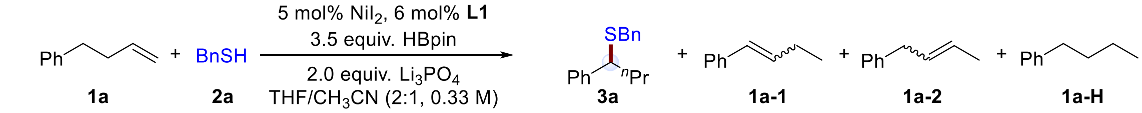

	1a alkene GC yield/%	2a thiol GC yield/%	3a remote hydrothiolation GC yield/%	1a-1 alkene GC yield/%	1a-2 alkene GC yield/%	1a-H alkane GC yield/%
1 h	79.5	96.7	2.1	12.5	3.4	5.6
2 h	66.1	95.0	3.2	16.5	4.8	8.9
4 h	45.3	90.0	7.3	30.6	7.3	16.5
8 h	3.2	73.4	23.5	32.9	7.6	42.8
18 h	0.0	16.9	70.0	7.9	0.0	48.4
24 h	0.0	4.9	86.0	0.0	0.0	50.9

Supplementary Figure 6. Tracing the alkene isomerization of standard reaction, Related to Figure 5a.

Supplementary Note 4. A mixture of olefins is observed when the reaction is run to partial conversion, indicating that olefin isomerization proceeds with fast dissociation and reassociation of the NiH species.

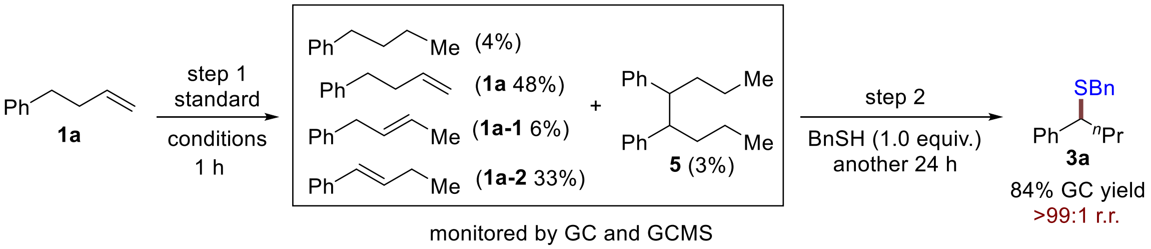

Supplementary Figure 7. Stepwise operation of olefin isomerization-hydrothiolation reaction, Related to Figure 5a.

To an oven-dried 8 mL screw-cap vial equipped with a magnetic stir bar was added NiI_2 (5.0 mol%) and bathocuproine (**L1**) (6.0 mol%). The vial was introduced into a nitrogen-filled glove box, anhydrous THF (0.40 mL) and CH_3CN (0.20 mL) were added, and the mixture was stirred for 10 min, at which time 4-phenyl-1-butene **(1a)** (52.8 mg, 0.40 mmol, 2.0 equiv.), HBpin (pinacolborane, 100 μL, 0.70 mmol, 3.5 equiv.) and Li_3PO_4 (50 mg, 0.40 mmol, 2.0 equiv.) were added to the resulting mixture in this order. The tube

was sealed with a teflon-lined screw cap, removed from the glove box and stirred at 60 °C for 1 h (the mixture was stirred at 750 rpm). A significant amount of other olefin isomers was detected as above demonstration by GC and GC-MS analysis. Then, the reaction vial was transfered to the glove box again with addition of BnSH (24.8 mg, 0.2 mmol, 1.0 equiv.). The tube was sealed with a teflon-lined screw cap, removed from the glove box and stirred at 60 °C for another 24 h (the mixture was stirred at 750 rpm).

Supplementary Note 5. Through a stepwise operation, the desired product **3a** was obtained in 84% GC yield and >99:1 r.r.. This result is is similar to the standard reaction. This result again indicates that the alkene isomerization was preceded to C-S bond formation.

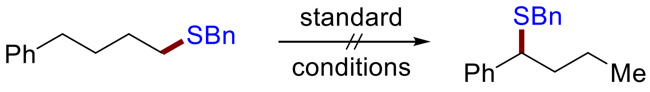

Supplementary Figure 8: Potential migratory process, Related to Figure 5c.

Following the general procedure **A** and similar to the preparation of **3a** while using benzyl(4-phenylbutyl)sulfane **3A** (51.2 mg, 0.2 mmol, 1.0 equiv.). GC and GC-MS analysis revealed that the desired product **3a** was not detected in this experiment, the thioether **3A** is fully recovered after the reaction.

Supplementary Note 6: No migratory reaction takes place when the linear sulfide (**3A**) is resubjected to the standard conditions, suggesting that chainwalking preceeds the C–S coupling.

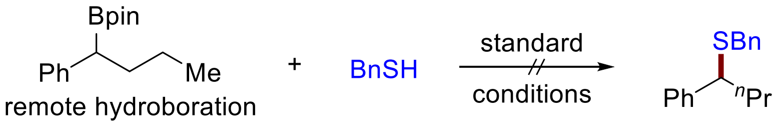

Supplementary Figure 9. Potential intermediate of alkene, Related to Figure 5c.

Following the general procedure **A** and similar to the preparation of **3a** while using 4,4,5,5-tetramethyl-2-(1-phenylbutyl)-1,3,2-dioxaborolane **6** (52.8 mg, 0.40 mmol, 2.0

equiv.). GC and GC-MS analysis revealed that the desired product **3a** was not detected in this experiment, the substrate **6** is fully recovered after the reaction.

Supplementary Note 7. Hydroboration intermediate **6** was resubjected to the standard conditions. However, no desired thiolation product was observed, suggesting that the C–S coupling step does not proceed through the remote hydroboration intermediate.

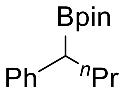

6

Substrate **6** was prepared following the known procedure accroding to the literature.[1]

4,4,5,5-tetramethyl-2-(1-phenylbutyl)-1,3,2-dioxaborolane:[1] ^{1}H NMR (400 MHz, CD_3CN): δ 7.26 – 7.22 (m, 2H), 7.17 – 7.10 (m, 3H), 2.27 (t, J = 7.8 Hz, 1H), 1.81 – 1.72 (m, 1H), 1.62 – 1.53 (m, 1H), 1.33 – 1.20 (m, 2H), 1.17 (s, 6H), 1.16 (s, 6H), 0.87 (t, J = 7.2 Hz, 3H); ^{13}C NMR (101 MHz, CD_3CN): δ 144.8, 129.2, 126.1, 118.3, 84.2, 35.6, 25.0, 23.0, 14.3; ^{11}B NMR (128 MHz, CD_3CN): δ 33.4; HRMS (ESI) calcd. for $C_{16}H_{26}BO_2$ $[M+H]^+$ m/z 261.2020, found 261.2020.

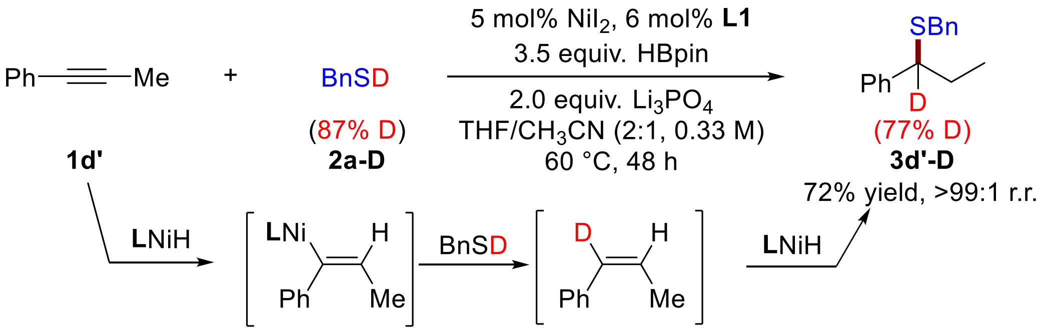

Supplementary Figure 10. Isotopic labelling experiment of alkyne **1d'** using deuterated thiol, Related to Figure 3 **3d'**.

Following the general procedure **A** and similar to the preparation of **3d'** while using prop-1-yn-1-ylbenzene (**1d'**) (46.4 mg, 0.40 mmol, 2.0 equiv.) and deuterated benzyl mercaptan (BnSD) **2a-D** (25.0 mg, 0.20 mmol, 1.0 equiv., 87% D). NMR and GC-MS analysis of the desired product **3d'-D** revealed that duterium incorporated at the benzylic position (77% D).

Supplementary Note 8. The vinylnickel intermediate formed upon hydrometallation of the alkyne is selectively captured by a proton source (thiol) forming an alkene. Isotope labelling experiments indicated that the source of protons in this reaction is mainly the thiol, while the alkylnickel intermediate formed upon hydrometallation of this alkene selectively engages with the chainwalking-thiolation reaction.

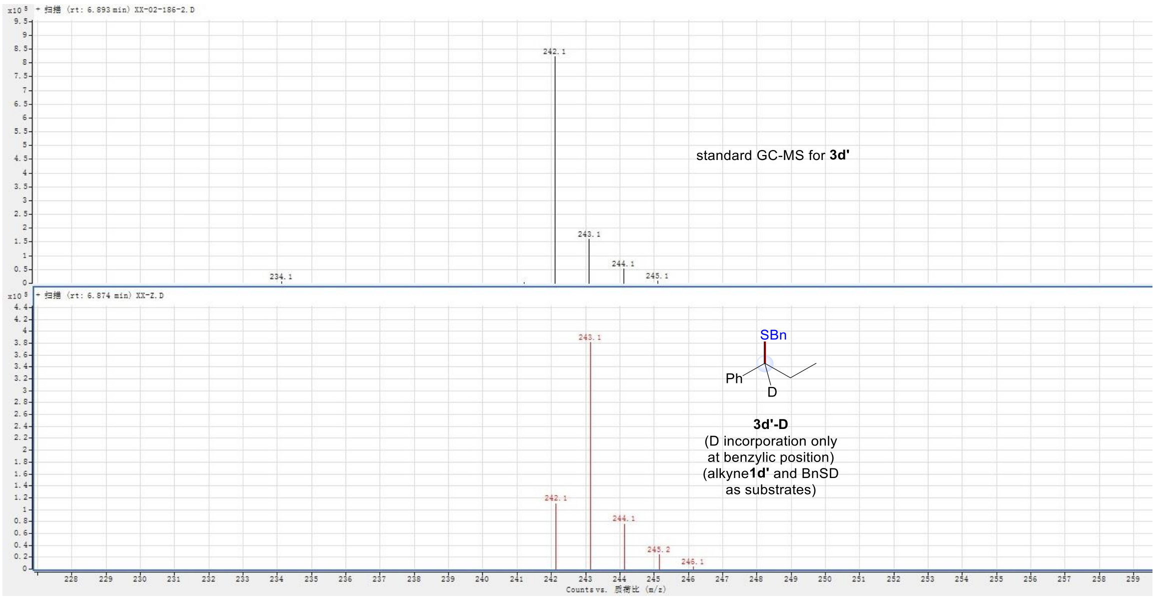

Supplementary Figure 11. GC-MS spectrum of **3d'** *vs* **3d'-D**, Related to Figure 3 **3d'**.

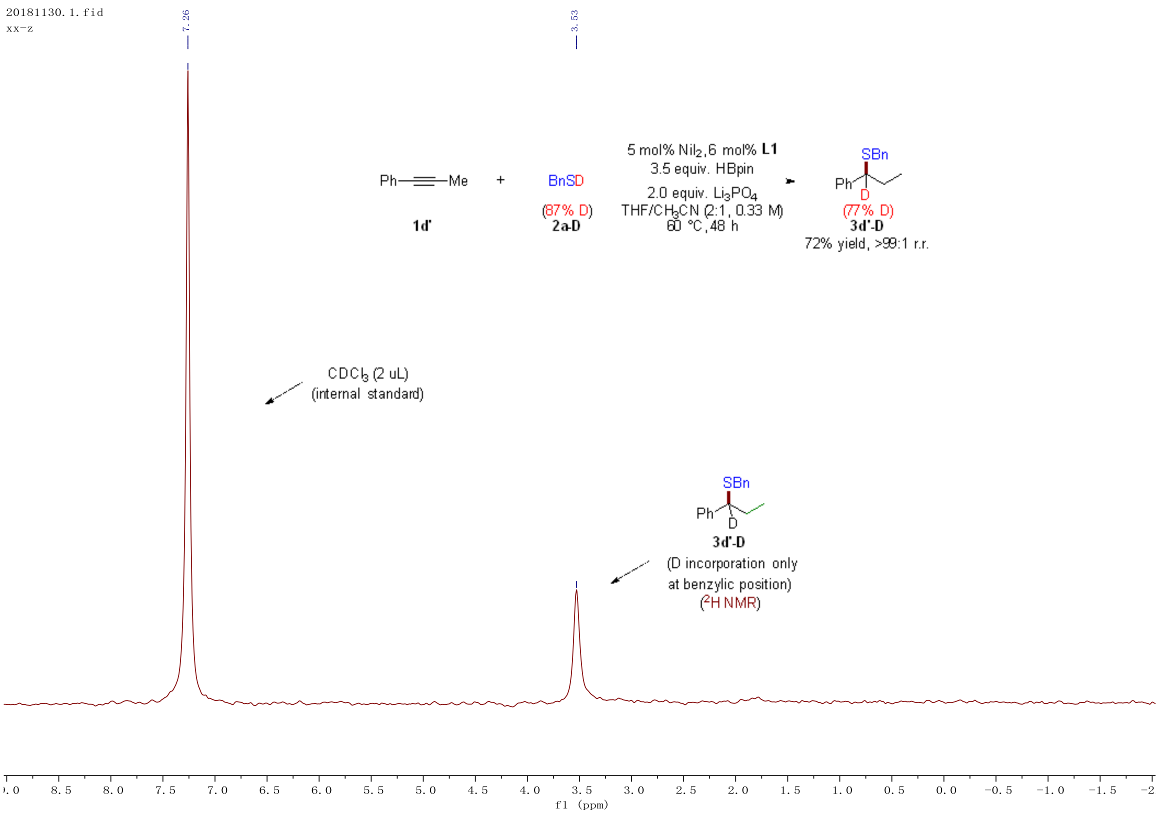

Supplementary Figure 12. ^{2}H NMR spectrum of **3d'-D**, Related to Figure 3 **3d'**.

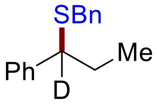

3d'-D

benzyl(1-phenylpropyl)sulfane-*d*: ^{1}H NMR (400 MHz, $CDCl_3$): δ 7.28 – 7.17 (m, 7H), 7.16 – 7.12 (m, 3H), 3.50 – 3.45 (m, 0.23H), 3.43 (d, J = 13.6 Hz, 1H), 3.33 (d, J = 13.6 Hz, 1H), 1.85 – 1.70 (m, 2H), 0.75 (t, J = 7.4 Hz, 3H); ^{13}C NMR (101 MHz, $CDCl_3$): δ 142.5, 138.6, 129.0, 128.5, 128.5, 128.3, 127.1, 126.9, 51.1, (35.5 & 35.5), (29.7 & 29.6), (12.4 & 12.4); ^{2}H NMR (92 MHz, $CHCl_3$ + $CDCl_3$): δ 3.53.

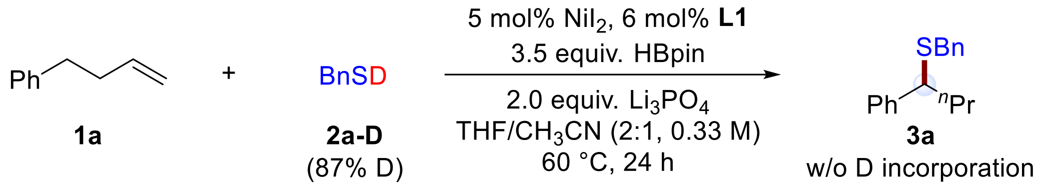

Supplementary Figure 13. Isotopic labelling experiment using deuterated thiol, Related to Figure 5b.

Following the general procedure **A** and similar to the preparation of **3a** while using deuterated benzyl mercaptan (BnSD) **2a-D** (25.0 mg, 0.20 mmol, 1.0 equiv., 87% D). NMR and GC-MS analysis revealed that no deuterium incorporation in the desired product **3a**.

Supplementary Note 9. No deuterium incorporation in the desired product is noted when deuterothiol is used, indicating that the thiol is not involved in chainwalking process.

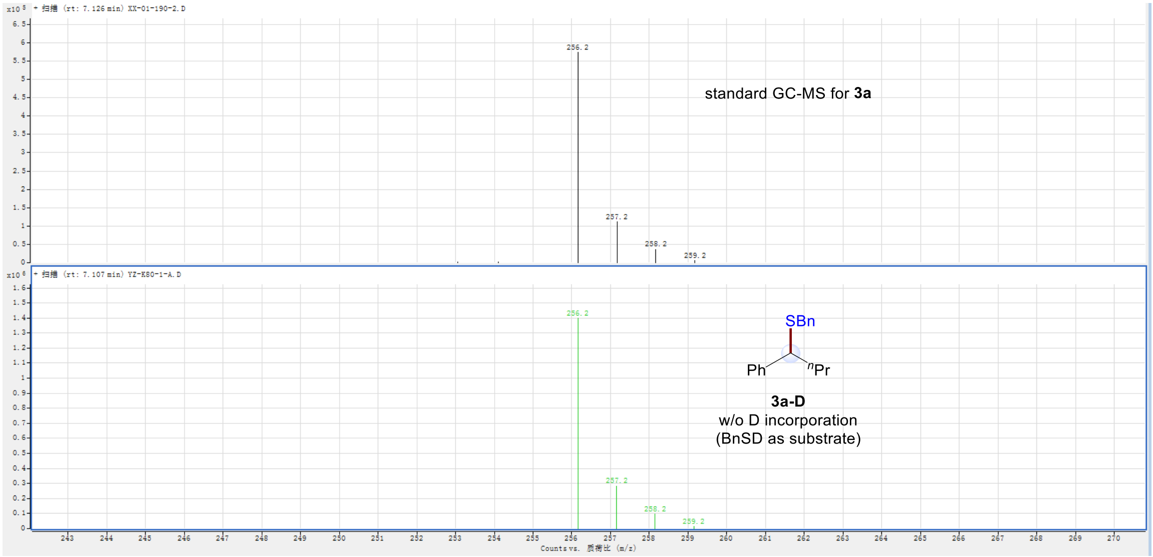

Supplementary Figure 14. GC-MS spectrum of **3a** *vs* **3a-D** in the reaction of Supplementary Figure 13, Related to Figure 5b.

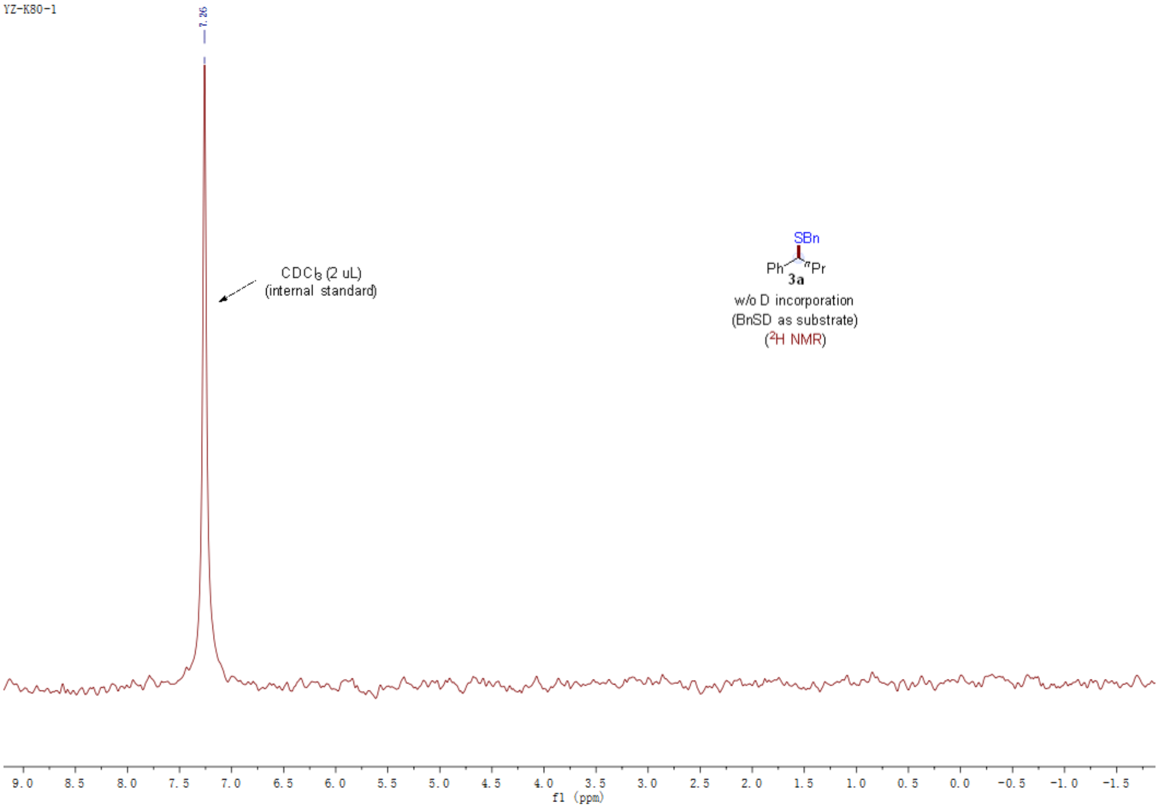

Supplementary Figure 15. ^{2}H NMR spectrum of **3a-D** in the reaction of Supplementary Figure 13, Related to Figure 5b.

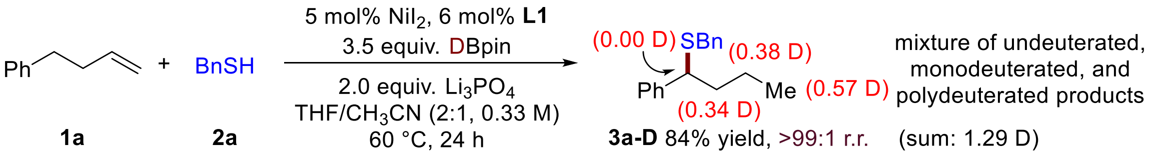

Supplementary Figure 16. Isotopic labelling experiment using deuterated DBpin, Related to Figure 5b.

Following the general procedure **A** and similar to the preparation of **3a** while using deuterated DBpin (100 uL, 0.70 mmol, >99% D, 3.5 equiv.). NMR analysis of the desired product **3a-D** revealed that deuterium scrambling and duterium incorporated at all positions except the benzylic position along the hydrocarbon chain. GC-MS analysis revealed that a mixture of undeuterated, monodeuterated, and poly deuterated products was obtained.

Supplementary Note 10: As expected, when DBpin is used, deuterium scrambling and deuterium incorporation is observed at all positions along the aliphatic chain with the exception of the benzylic position. Mass spectrometric analysis revealed that a mixture of undeuterated, monodeuterated, and polydeuterated products is obtained. This is consistent with the hypothesis that chainwalking occurs with dissociation and reassociation of free NiH/NiD from the NiH/NiD-alkene complex.

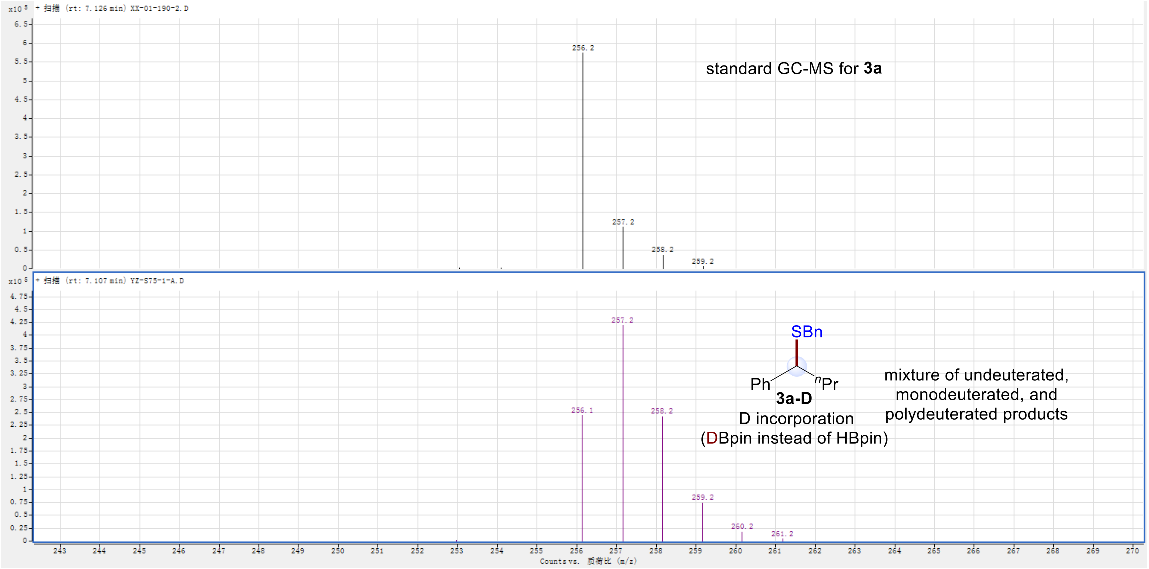

Supplementary Figure 17. GC-MS spectrum of **3a** *vs* **3a-D** in the reaction of Supplementary Figure 16, Related to Figure 5b.

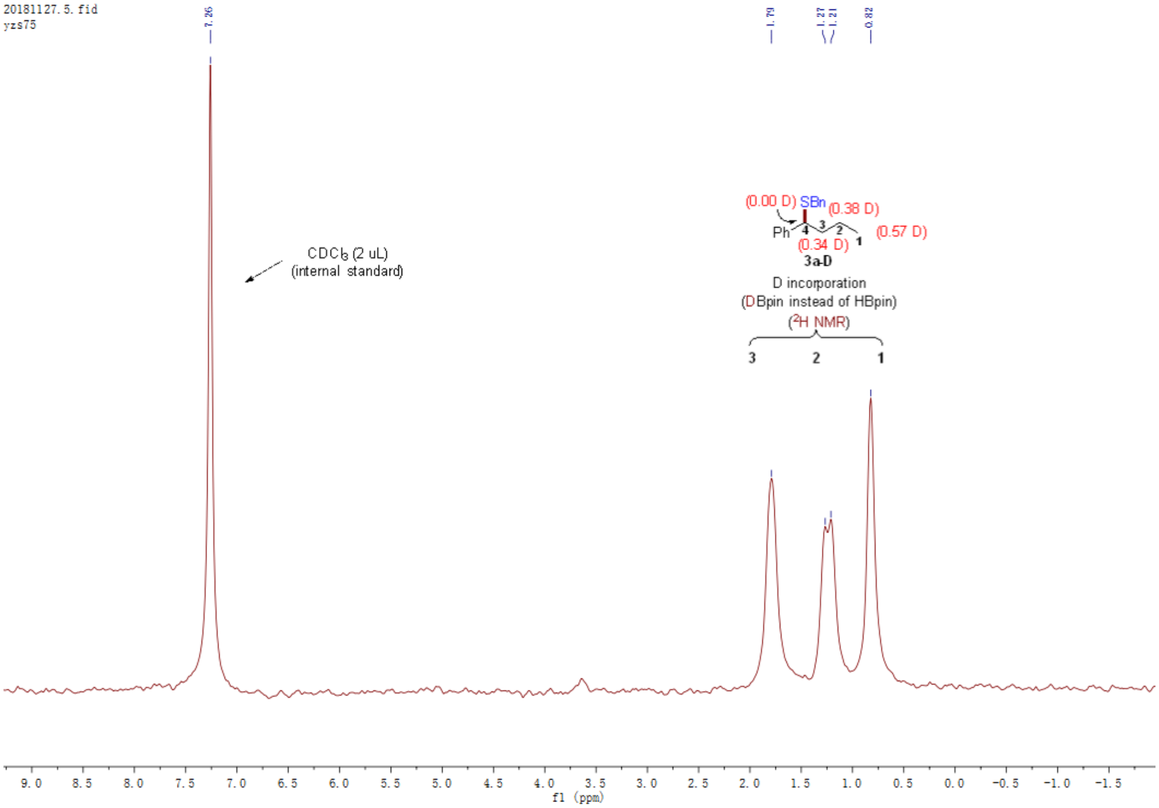

Supplementary Figure 18: ^{2}H NMR spectrum of **3a-D** in the reaction of Supplementary Figure 16, Related to Figure 5b.

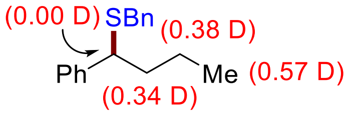

3a-D

benzyl(1-phenylbutyl)sulfane-*d*: ^{1}H NMR (400 MHz, $CDCl_3$): δ 7.26 – 7.16 (m, 6H), 7.14 – 7.11 (m, 4H), 3.53 (dd, *J* = 8.4, 6.4 Hz, 1H), 3.41 (d, *J* = 13.6 Hz, 1H), 3.31 (d, *J* = 13.6 Hz, 1H), 1.78 – 1.63 (m, 1.66 H), 1.25 – 1.06 (m, 1.62H), 0.75 – 0.71 (m, 2.43H).; ^{13}C NMR (101 MHz, $CDCl_3$): δ 142.9, 138.6, 129.0, 128.5, 128.5, 128.2, 127.1, 126.9, (49.1 & 49.0), (38.7 & 38.7 & 38.6), 35.5, (20.9 & 20.8 & 20.7), (13.9 & 13.9 & 13.8); ^{2}H NMR (92 MHz, $CHCl_3$ + $CDCl_3$): δ 1.79, 1.27, 1.21, 0.82.

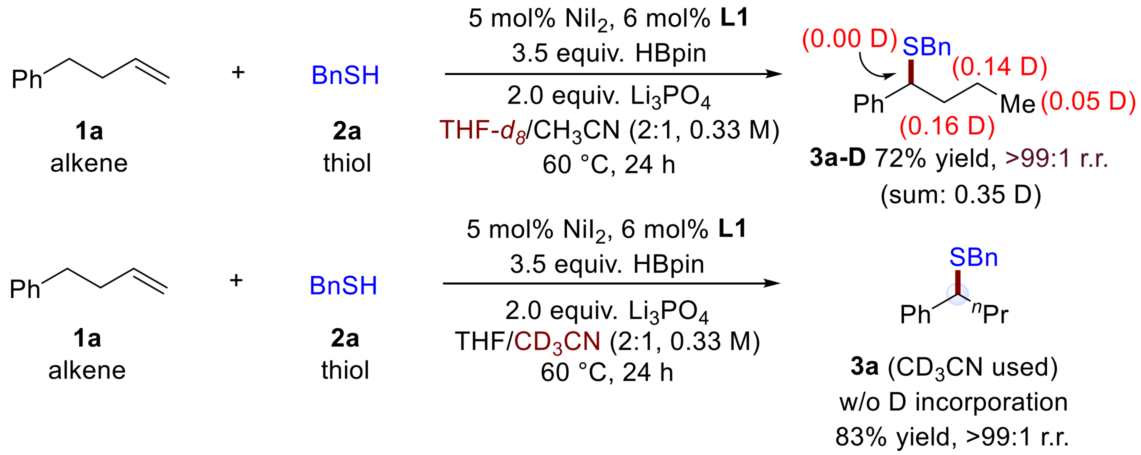

Supplementary Figure 19. Isotopic labelling experiment using deuterated solvents, Related to Figure 8d.

Following the general procedure **A** and similar to the preparation of **3a** while using anhydrous mixed solvents (0.40 mL THF-d_8 and 0.20 mL CH_3CN) or (0.40 mL THF and 0.20 mL CD_3CN).

a) When CD_3CN was used, NMR and GC-MS analysis revealed that no deuterium incorporation in the desired product **3a**.

b) When THF-d_8 was used, NMR analysis of the desired product **3a-D** revealed that a small amount of deuterium scrambling and duterium incorporated at all positions except the benzylic position along the hydrocarbon chain. GC-MS analysis revealed that a mixture of undeuterated, monodeuterated, and poly deuterated products was obtained.

Supplementary Note 11. This indicates that a small amount of NiD is involved in the chainwalking process and the small amount of NiD should come from the deuterated THF-d_8.

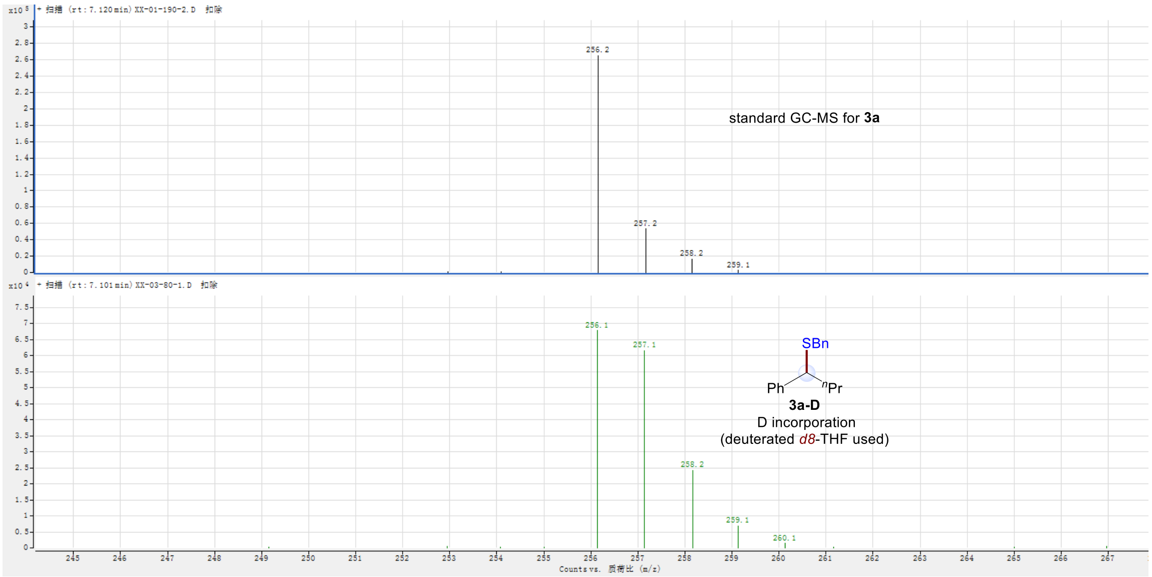

Supplementary Figure 20. GC-MS spectrum of **3a** *vs* **3a-D** in the reaction of Supplementary Figure 19b, Related to Figure 8d.

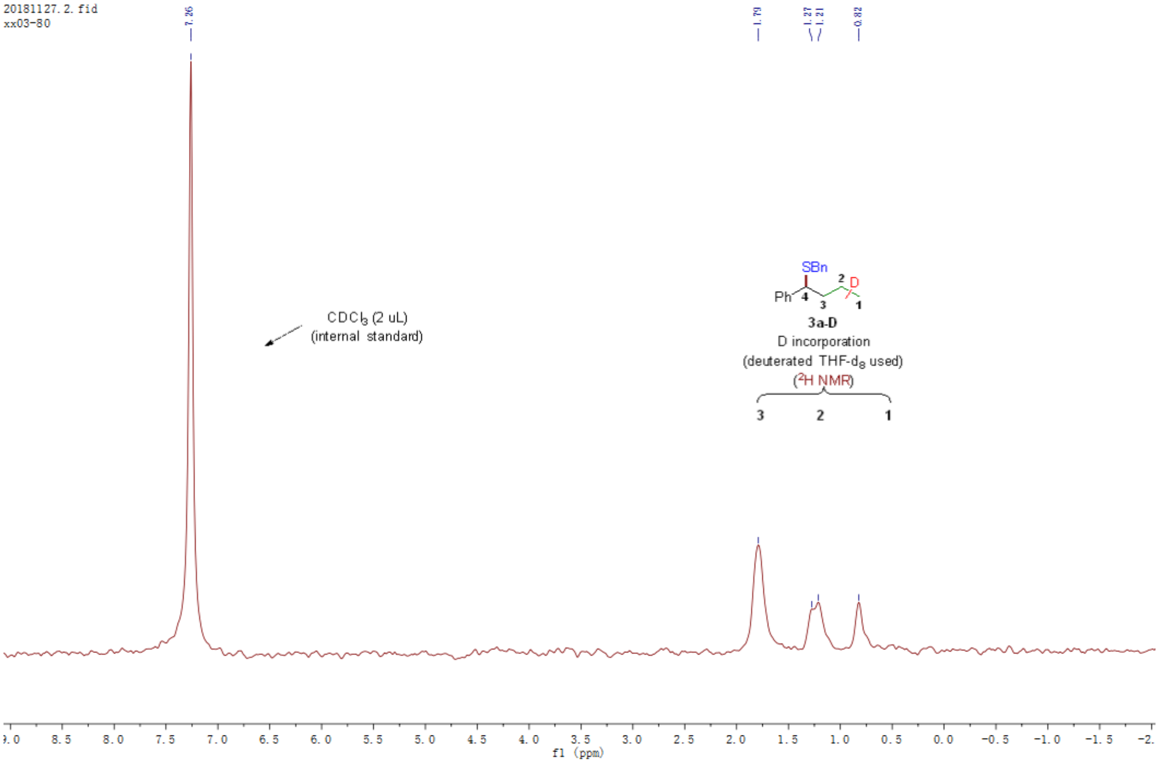

Supplementary Figure 21. ^{2}H NMR spectrum of **3a-D** in the reaction of Supplementary Figure 19b, Related to Figure 8d.

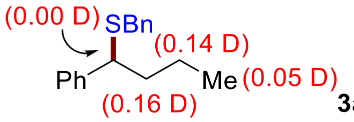

3a-D

benzyl(1-phenylbutyl)sulfane-*d* (also see Figure 8d, THF-d_8 was used)**:** ^{1}H NMR (400 MHz, $CDCl_3$): δ 7.27 – 7.17 (m, 7H), 7.16 – 7.11 (m, 3H), 3.55 (dd, J = 8.4, 6.4 Hz, 1H), 3.41 (d, J = 13.6 Hz, 1H), 3.31 (d, J = 13.6 Hz, 1H), 1.78 – 1.64 (m, 1.84H), 1.25 – 1.06 (m, 1.86H), 0.75 – 0.72 (m, 2.95H); ^{13}C NMR (101 MHz, $CDCl_3$): δ 142.9, 138.6, 129.0, 128.5, 128.5, 128.2, 127.1, 126.9, 49.1, 38.7, 35.5, 20.9, 13.9; ^{2}H NMR (92 MHz, $CHCl_3$ + $CDCl_3$): δ 1.79, 1.27, 1.21, 0.82.

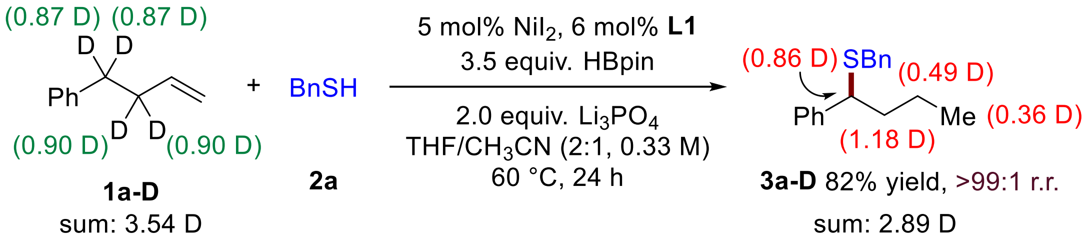

Supplementary Figure 22. Isotopic labelling experiment using deuterated alkene **1a-D**.

Following the general procedure **A** and similar to the preparation of **3a** while using deuterated 4-phenyl-1-butene **(1a-D)** (52.8 mg, 0.40 mmol, 2.0 equiv.). NMR and GC-MS analysis of the desired product **3a-D** revealed that deuterium scrambling and duterium incorporated at all positions along the hydrocarbon chain.

Supplementary Note 12. This is consistent with the hypothesis that chainwalking occurs through β-hydride elimination/reinsertion.

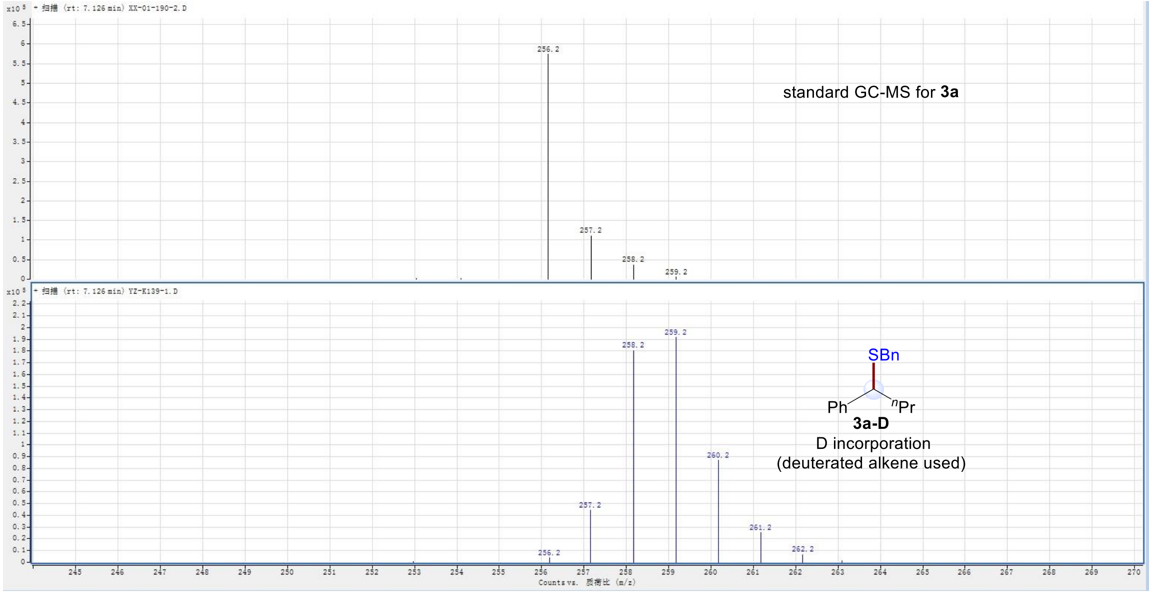

Supplementary Figure 23. GC-MS spectrum of **3a** *vs* **3a-D** in the reaction of Supplementary Figure 22.

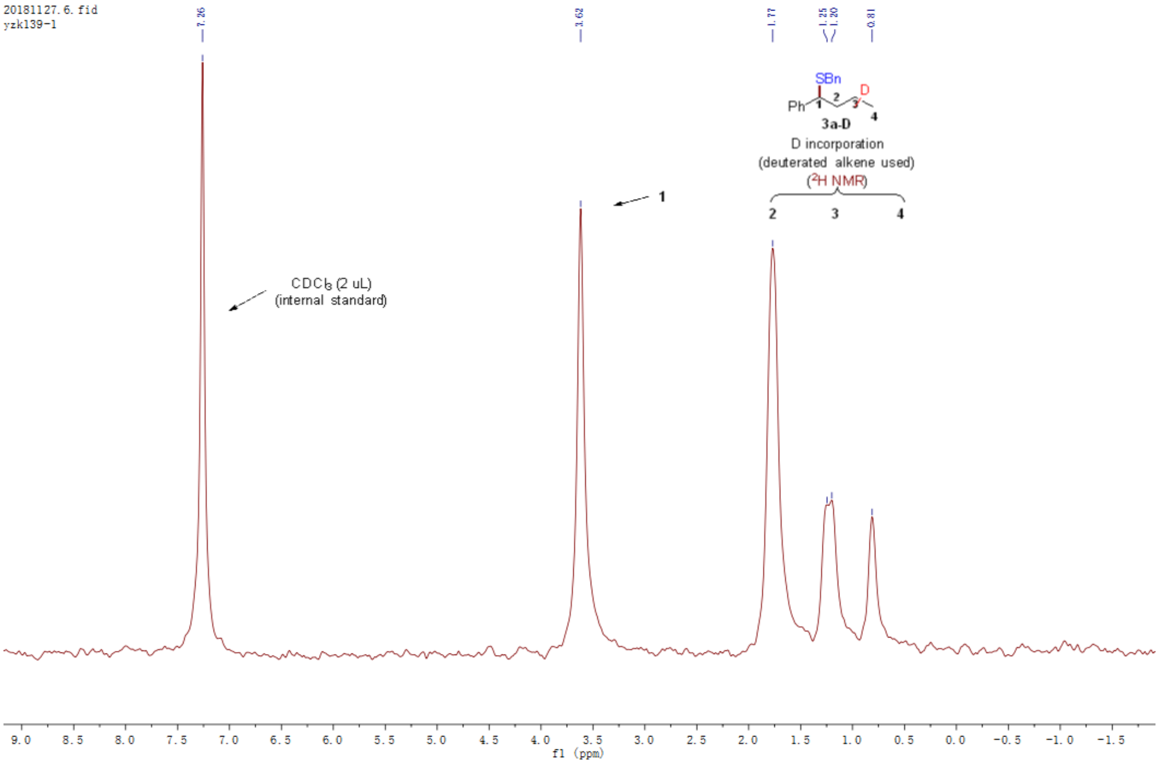

Supplementary Figure 24. ^{2}H NMR spectrum of **3a-D** in the reaction of Supplementary Figure 22.

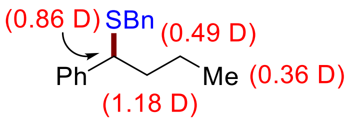

3a-D

benzyl(1-phenylbutyl)sulfane-*d*: ^{1}H NMR (500 MHz, $CDCl_3$): δ 7.37 – 7.28 (m, 7H), 7.26 – 7.23 (m, 3H), 3.68 – 3.63 (m, 0.14H), 3.53 (d, J = 13.5 Hz, 1H), 3.42 (d, J = 13.5 Hz, 1H), 1.86 – 1.76 (m, 0.82H), 1.35 – 1.19 (m, 1.51H), 0.86 – 0.81 (m, 2.64H); ^{13}C NMR (101 MHz, $CDCl_3$): δ 142.8, 138.6, 129.0, 128.5, 128.5, 128.2, 127.1, 126.9, (49.1 & 49.0 & 49.0), (38.6 & 38.5 & 38.3 & 38.2 & 37.9), 35.5, (20.9 & 20.8 & 20.7), (13.9 & 13.8 & 13.7 & 13.7); ^{2}H NMR (92 MHz, $CHCl_3$ + $CDCl_3$): δ 3.62, 1.77, 1.25, 1.20, 0.81.

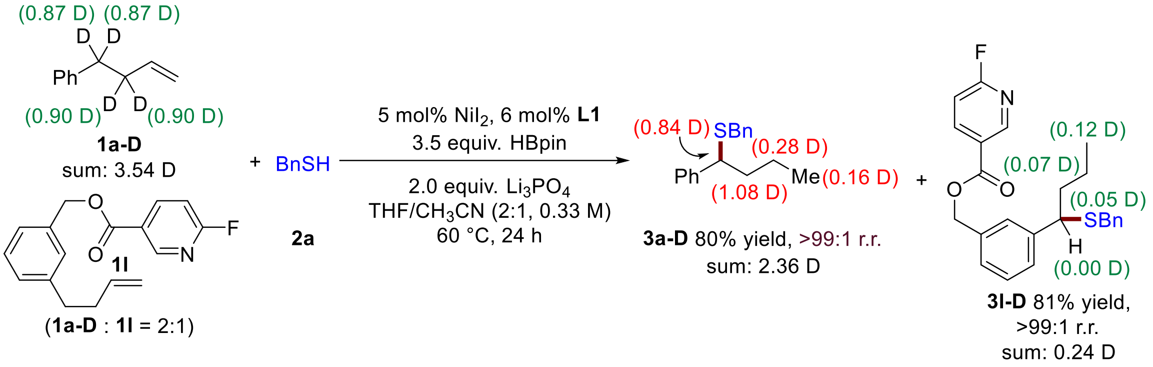

Supplementary Figure 25. Crossover experiment with a mixture of deuterated and undeuterated alkenes.

Experimental procedure: To an oven-dried 8 mL screw-cap vial equipped with a magnetic stir bar was added NiI_2 (5.0 mol%) and bathocuproine (**L1**) (6.0 mol%). The vial was introduced into a nitrogen-filled glove box, anhydrous THF (1.2 mL) and CH_3CN (0.6 mL) were added, and the mixture was stirred for 10 min, at which time deuterated 4-phenyl-1-butene **(1a-D)** (105.6 mg, 0.8 mmol, 4.0 equiv.), 3-(but-3-en-1-yl)benzyl 6-fluoronicotinate (**1l**) (114.0 mg, 0.40 mmol, 2.0 equiv.), benzyl mercaptan **2a** (0.4 mmol, 1.0 equiv.), HBpin (pinacolborane, 300 µL, 2.1 mmol, 3.5 equiv.) and Li_3PO_4 (150 mg, 1.2 mmol, 6.0 equiv.) were added to the resulting mixture in this order. The tube was sealed with a teflon-lined screw cap, removed from the glove box and stirred at

60 °C for 24 h (the mixture was stirred at 750 rpm). After the reaction was completed, the reaction mixture was directly filtered through a short pad of silica gel (using ethyl acetate in hexanes) to give the crude product. *n*-Tetradecane (20 μL) was added as an internal standard for GC analysis. The crude material was purified by flash column chromatography (1–2% EtOAc in hexanes) to provide the title compound as a colorless oil. NMR and GC-MS analysis of **3l-D** revealed that deuterium scrambling and duterium incorporated at all positions except the benzylic position along the hydrocarbon chain.

Supplementary Note 13. This is consistent with the hypothesis that chainwalking occurs with dissociation and reassociation of free NiH/NiD from the NiH/NiD-alkene complex.

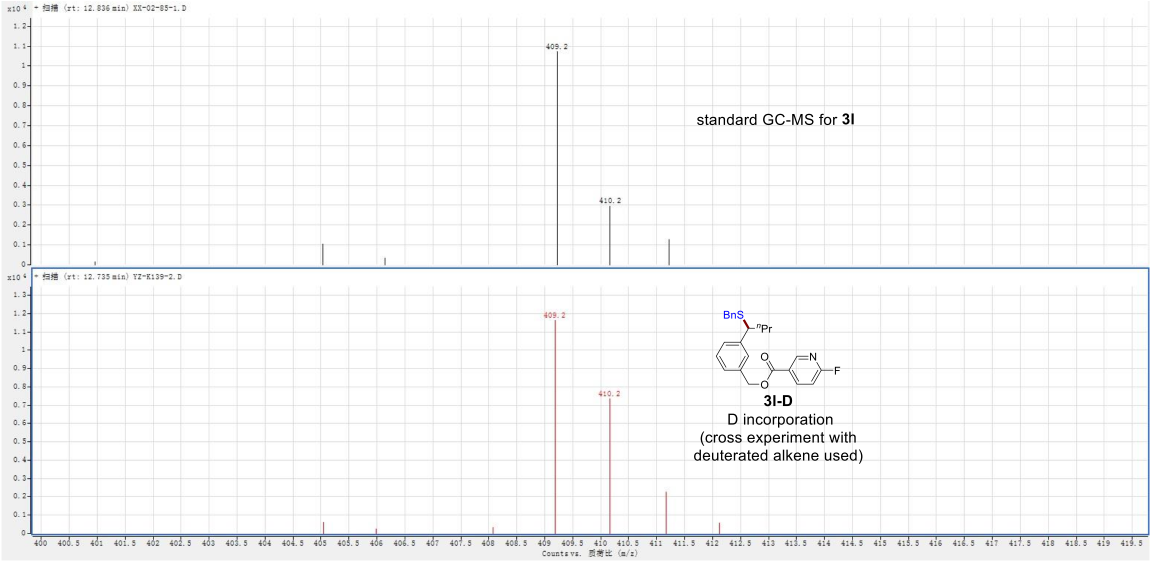

Supplementary Figure 26. GC-MS spectrum of **3l** *vs* **3l-D**.

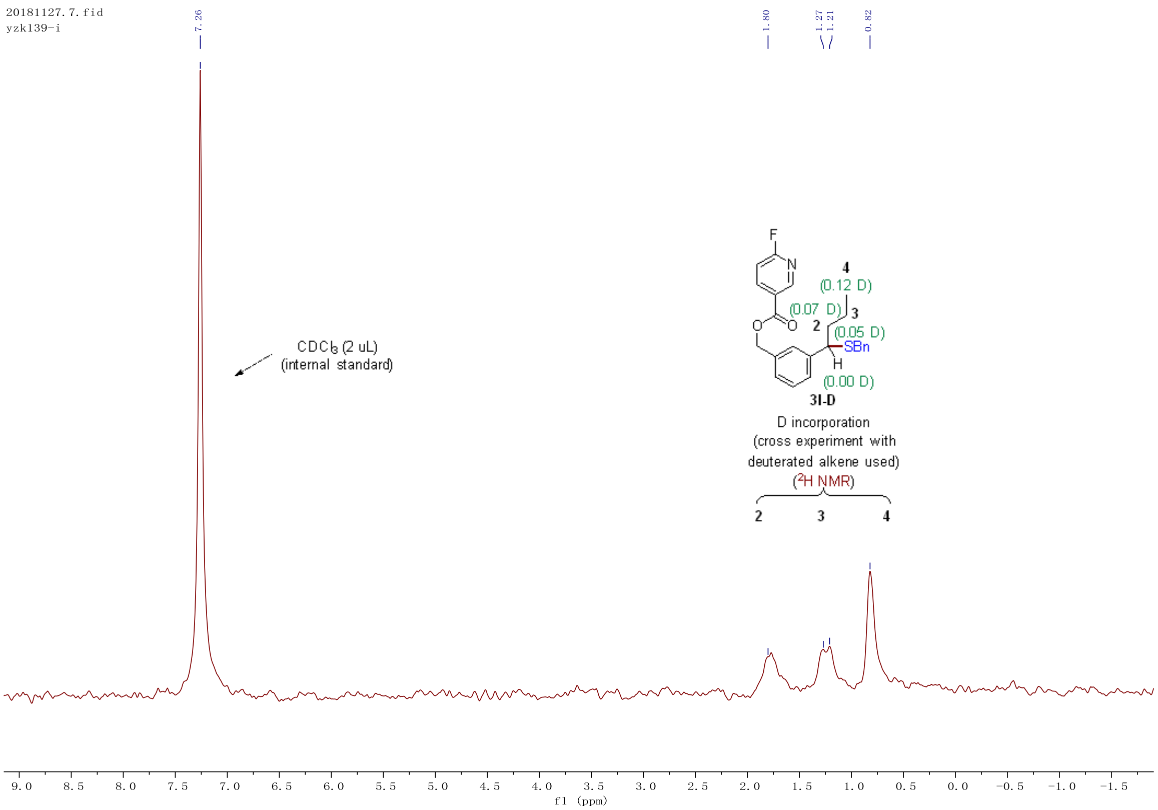

Supplementary Figure 27. ^{2}H NMR spectrum of **3l-D** in the reaction of Supplementary Figure 25.

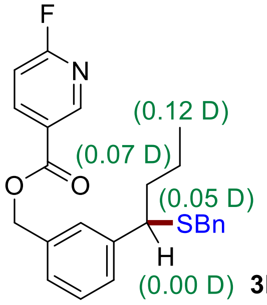

3l-D

3-(1-(benzylthio)butyl)benzyl 6-fluoronicotinate-*d*: ^{1}H NMR (500 MHz, $CDCl_3$): δ 8.96 (d, J = 2.5 Hz, 1H), 8.48 – 8.44 (m, 1H), 7.40 – 7.34 (m, 3H), 7.32 – 7.27 (m, 3H), 7.24 – 7.20 (m, 3H), 7.03 (dd, J = 8.5, 2.5 Hz, 1H), 5.41 (s, 2H), 3.67 (dd, J = 8.5, 6.5 Hz, 1H), 3.53 (d, J = 13.5 Hz, 1H), 3.42 (d, J = 13.5 Hz, 1H), 1.89 – 1.75 (m, 1.95H), 1.36 – 1.19 (m, 1.93H), 0.84 (t, J = 7.4 Hz, 2.88H); ^{13}C NMR (101 MHz, $CDCl_3$): δ 165.6 (d, J_{C-F} = 282.8 Hz), 165.0, 150.6 (d, J_{C-F} = 14.1 Hz), 143.6, 142.8 (d, J_{C-F} = 7.1 Hz), 138.4,

135.5, 129.0, 128.9, 128.5, 128.4, 128.1, 127.1, 127.0, 124.5 (d, $J_{C\text{-}F}$ = 3.0 Hz), 109.7 (d, $J_{C\text{-}F}$ = 30.3 Hz), 67.4, (48.9 & 48.8), (38.7 & 38.6), 35.6, (20.9 & 20.8), (13.9 & 13.7); ^{2}H NMR (92 MHz, $CHCl_3$ + $CDCl_3$): δ 1.80, 1.27, 1.21, 0.82.

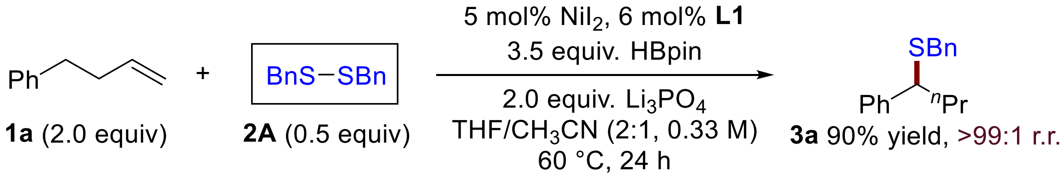

Supplementary Figure 28. Potential intermediates of thiol, Related to Figure 6a.

Following the general procedure **A** and similar to the preparation of **3a** while using 1,2-dibenzyldisulfane (**2A**) (25.0 mg, 0.10 mmol, 0.50 equiv.).

Supplementary Note 14. When 0.5 equiv. of a symmetrical disulfide is used instead of 1.0 equiv. of the corresponding thiol, the desired remote hydrothiolation product is obtained in a comparable yield, indicating that the disulfide might be the potential reactive intermediate of the thiol.

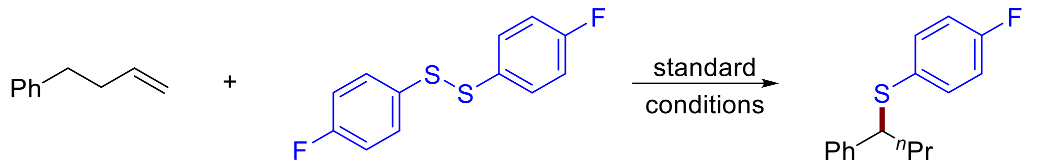

1a **2P** **4p** 86% yield, >99:1 r.r.

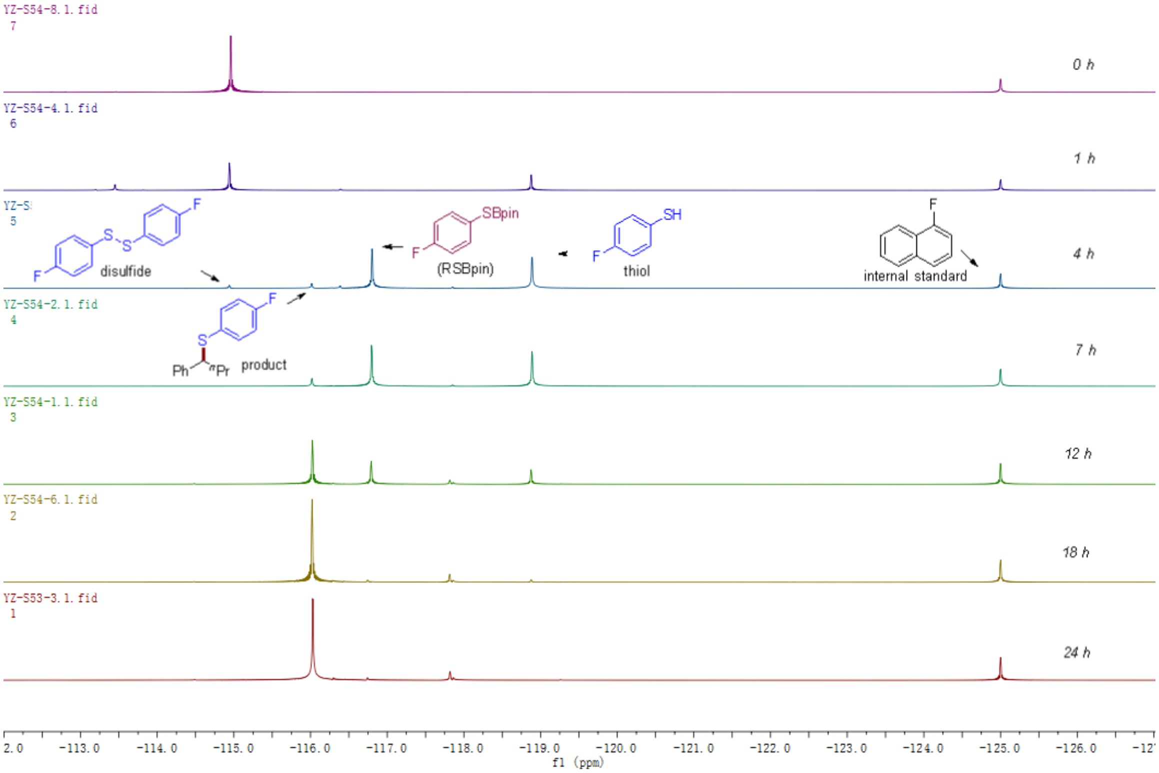

Supplementary Figure 29. Disulfide reaction progress monitored by *in situ* ^{19}F NMR (376 MHz, CD_3CN), Related to Figure 6b. Chemical shifts of the four peaks (δ = –114.9, –116.0, –116.8, and –118.9 ppm).

Following the general procedure **B** and similar to the preparation of **4p** while using 1,2-bis(4-fluorophenyl)disulfane (**2P**) (25.4 mg, 0.10 mmol, 0.50 equiv.). 1-Fluoronaphthalene (5 uL) was added as an internal standard for ^{19}F NMR analysis.

Supplementary Note 15. Monitoring the remote hydrothiolation reaction of a disulfide by ^{19}F NMR however indicates that the disulfide (δ = –114.9) is first transformed into an RS−Bpin intermediate (δ = –116.8) which is illustrated below. This results suggests that the *in situ* generated RS−Bpin might be the actual thiolation reagent.

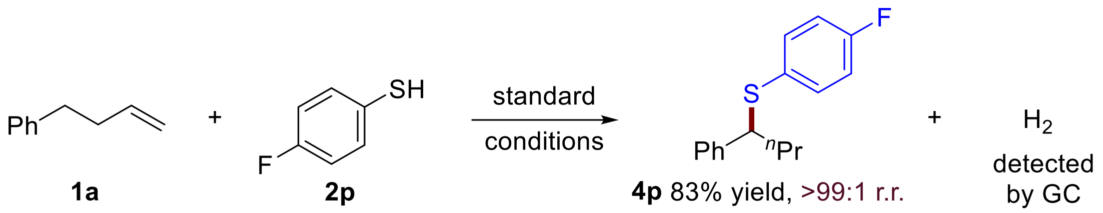

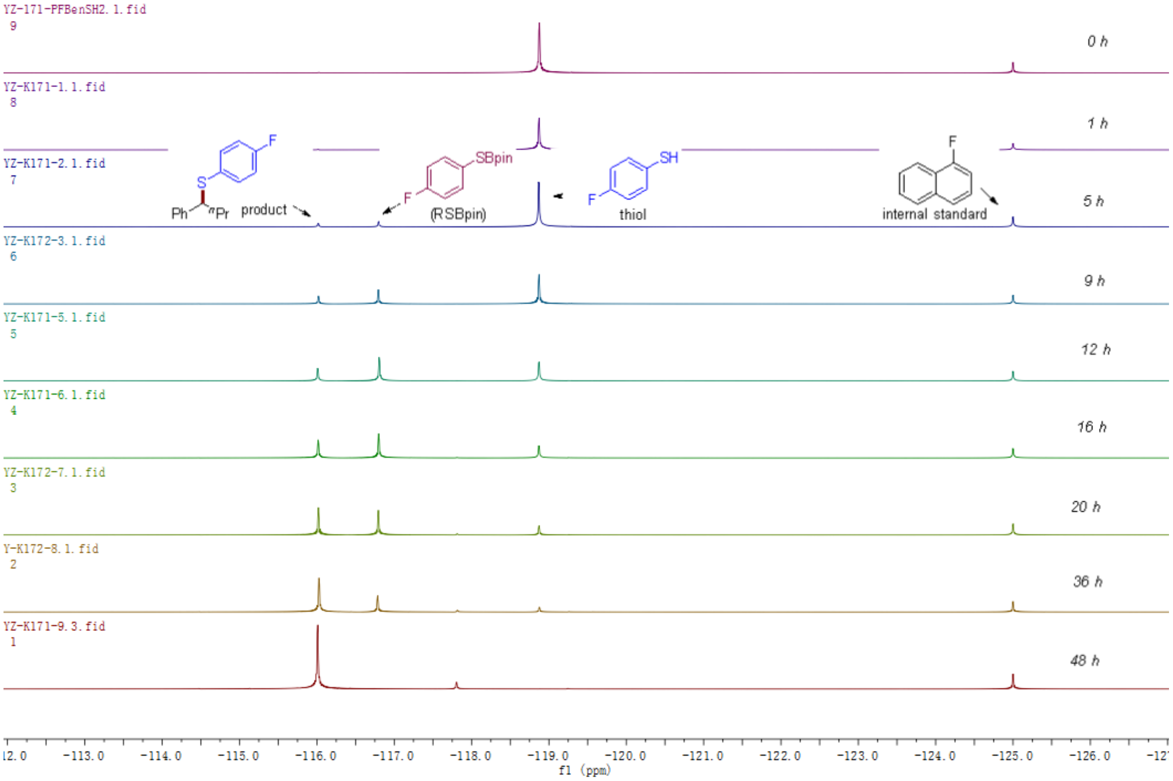

Supplementary Figure 30. Standard reaction progress monitored by *in situ* ^{19}F NMR (376 MHz, CD_3CN), Related to Figure 6c. Chemical shifts of the three peaks (δ = –116.0, –116.8, and –118.9 ppm).

Following the general procedure **B** and similar to the preparation of **4p**. 1-Fluoronaphthalene (5 μL) was added as an internal standard for ^{19}F NMR analysis.

Supplementary Note 16. Analogous experiments on the corresponding thiol substrate (δ = –118.9) also reveal the generation of this RS−Bpin intermediate (δ = –116.8) with no trace of disulfide detected which is illustrated below. These results reinforce the notion

that the disulfide is not involved as the active intermediates from the thiol, and suggests that the *in situ* generated RS−Bpin might be the actual thiolation reagent.

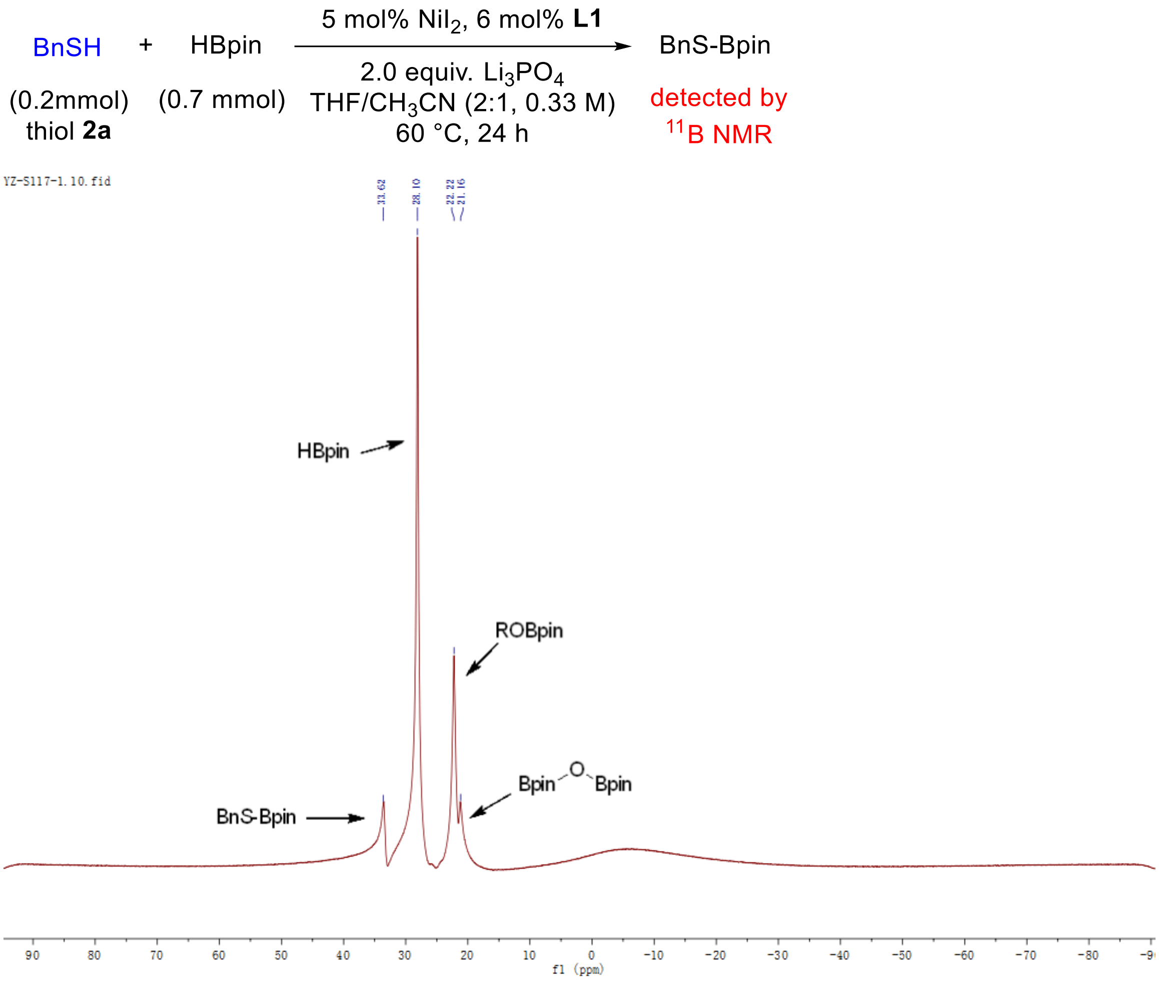

Supplementary Figure 31. Progress of the standard reaction monitored by *in situ* ^{11}B NMR in the absence of alkene **1a**, Related to Figure 8e.

Following the general procedure **A** in the absence of alkene **1a**. Pinacolborane (5 μL) was added as an internal standard for ^{11}B NMR analysis.

Supplementary Note 17. In the absence of alkene **1a**, the generation of RS−Bpin intermediate (δ = 33.6) is confirmed in the standard reaction by ^{11}B NMR spectroscopic analysis.

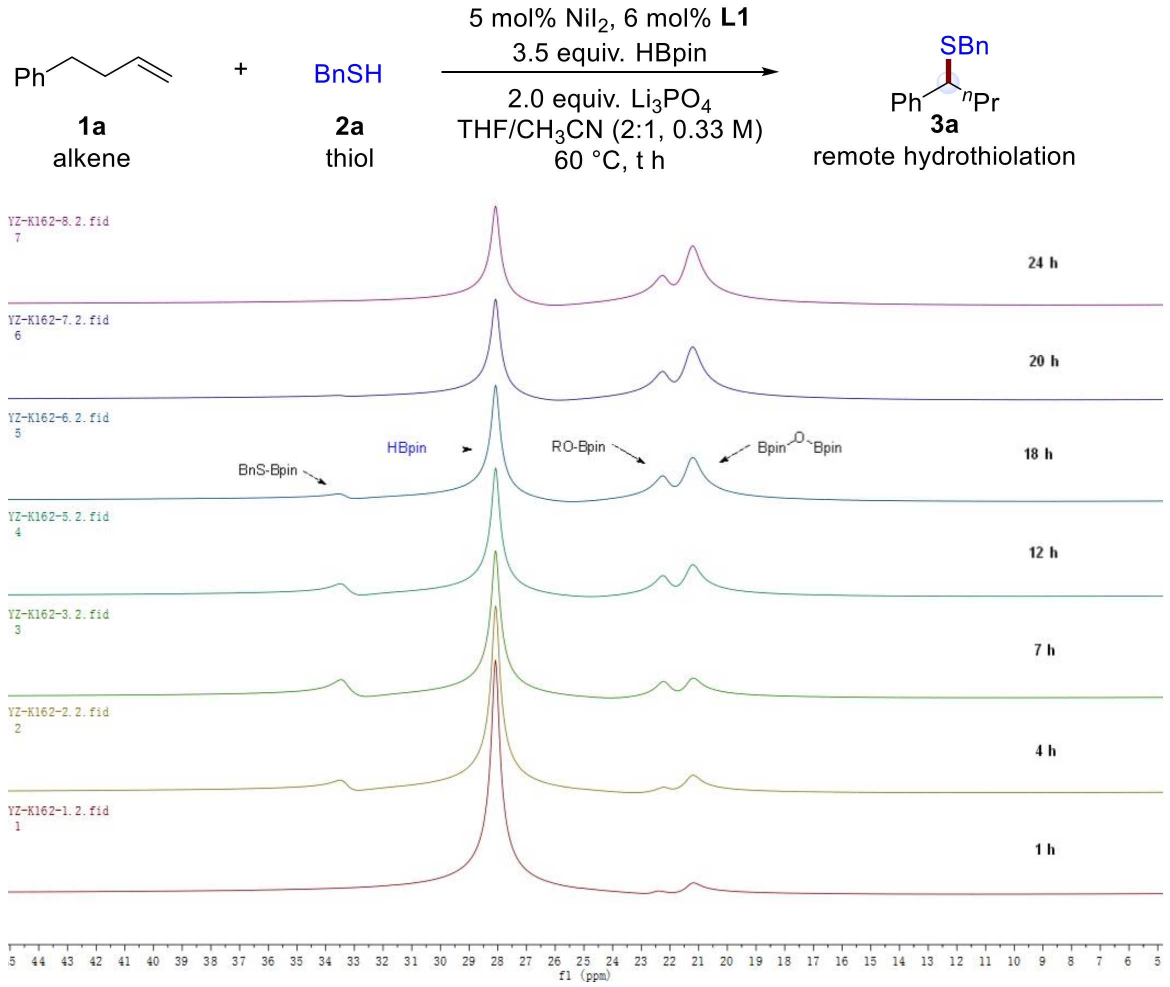

Supplementary Figure 32. Progress of the standard reaction monitored by *in situ* ^{11}B NMR, Related to Figure 8e. Stacked ^{11}B NMR (128 MHz, CD_3CN) spectra of standard reaction, chemical shifts of the four peaks (δ = 33.6, 28.1, 22.3, and 21.2 ppm).

Following the general procedure **A** and similar to the preparation of **3a**. Pinacolborane (5 μL) was added as an internal standard for ^{11}B NMR analysis.

Supplementary Note 18. The generation of RS−Bpin intermediate (δ = 33.6) is confirmed again in the standard reaction by ^{11}B NMR spectroscopic analysis. We could also observe two new boron signals accompanied with the consumption of THF, which matches the signals of ROBpin (δ = 22.2) and BpinOBpin (δ = 21.2).

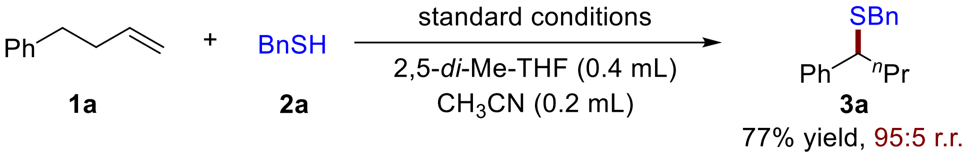

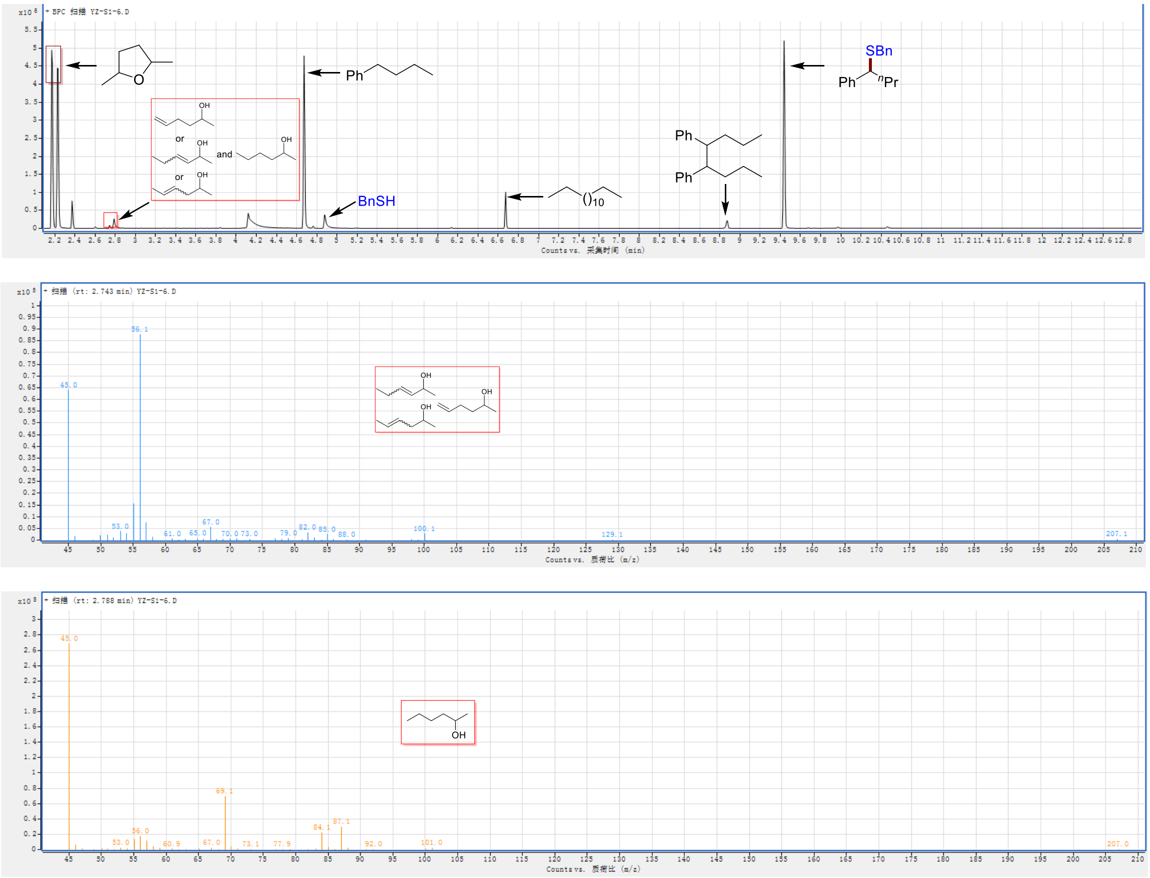

Supplementary Figure 33. Standard reaction detected by GC and GC-MS, Related to Figure 8c.

Supplementary Note 19. We observe the beta-hydride elimination product by GC and GC-MS in the form of alcohol.

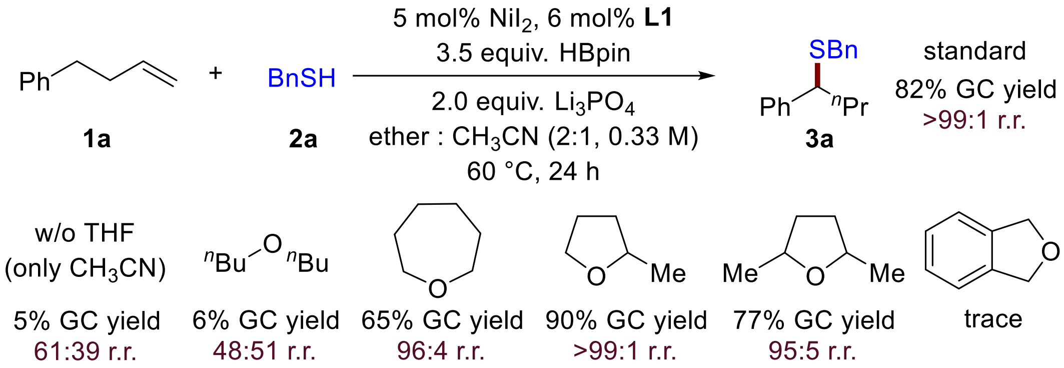

Supplementary Figure 34. Variation of ether solvent (only change THF), Related to Figure 8a.

Following the general procedure **A** and similar to the preparation of **3a** while using anhydrous ether (0.4 mL) and CH_3CN (0.2 mL).

Supplementary Note 20: A different reactivity is observed when the solvent THF is replaced by a variety of other ethers in the standard reaction conditions. The nature of the ether backbone plays a crucial role; we found that only cyclic ethers with a β-hydride can produce the desired product in a reasonable yield. In contrast, acyclic ethers or cyclic ethers lacking a β-hydride do not have such a profound effect on reactivity. We postulated that the ether solvent might participate in the catalytic cycle.

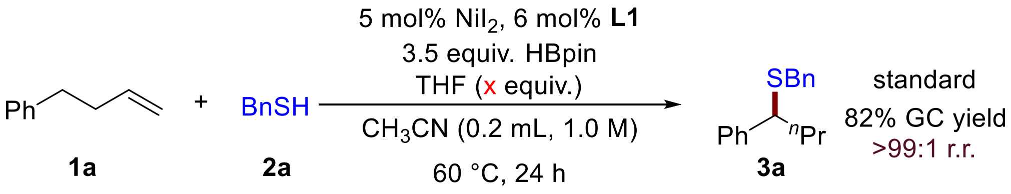

entry	equiv. of THF	GC yield	r.r.	entry	equiv. of THF	GC yield	r.r.
1	0	5%	61:39	6	3.0	68%	96:4
2	0.3	26%	84:16	7	6.0	72%	96:4
3	0.5	43%	91:9	8	12.0	75%	97:3
4	1.0	54%	93:7	9	18.0	78%	98:2
5	1.5	61%	94:6	10	24.0	82%	>99:1

Supplementary Figure 35. Relationship between the yield and the amount of THF, Related to Figure 8b.

Following the general procedure **A** and similar to the preparation of **3a** while using THF (x equiv.) and CH_3CN (0.20 mL, 1.0 M).

Supplementary Note 21: Both the yields and regioselectivities improve when the amount of THF is increased. We postulate that the THF might participate in the catalytic cycle.

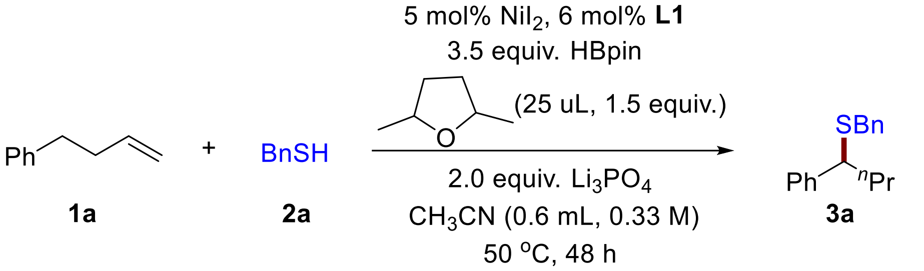

entry	time/h	consumption of ether/equiv.	yield for **3a**/%	r.r.
1	3	0.01	0.6	43:57
2	6	0.02	1.2	51:49
3	12	0.2	14	74:26
4	18	0.8	27	85:15
5	24	0.9	34	86:14
6	36	1.3	42	90:10
7	42	1.4	47	91:9
8	48	1.5	52	91:9

Supplementary Figure 36. Consumption of ether, Related to Figure 8c.

Following the general procedure **A** and similar to the preparation of **3a** while using anhydrous ether (25 uL, 1.5 equiv.) and CH_3CN (0.60 mL, 0.33 M).

Supplementary Note 22: The consumption of ether could also be observed during the reaction process. Only trace amounts of product (~1% yield) are produced during the first 6 h and the regioselectivity is poor in the first 12 h of the reaction. Subsequently, the yield of desired product increases significantly but the yield of other regioisomers (linear isomer) fails to increase after the first 12 h. The origin of this apparent induction period as well as initial low regioselectivity is still under investigation.

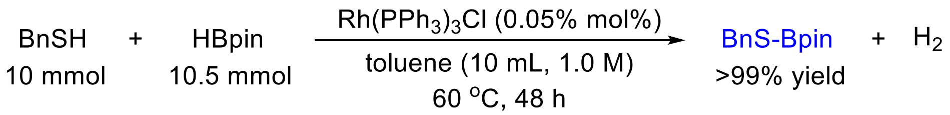

Supplementary Figure 37. Preparation of BnS-Bpin reagent according to literature.

Known experimental procedure for the preparation of BnS-Bpin reagent:[2] An oven-dried 100 mL round-bottom flask equipped with a magnetic stir bar was charged with $Rh(PPh_3)_3Cl$ (4.6 mg, 0.005 mmol, 0.05 mol%) and benzyl mercaptan BnSH (1.24 g, 10 mmol, 1.0 equiv.) and 4,4,5,5-tetramethyl-1,3,2-dioxaborolane HBpin (1.35 g, 10.5 mmol, 1.05 equiv.). The flask was evacuated and backfilled with argon (this process was repeated for a total of three times). Anhydrous toluene (10.0 mL, 1.0 M) were added, and the mixture was stirred for 48 h at 60 ^oC. After the reaction was complete, the reaction flask was removed from the oil bath. Solvent was removed in vacuo and the crude reaction mixture was purified by recrystylation (using Et_2O at –30 ^oC) to provide the title compound BnS-Bpin. **2-(benzylthio)-4,4,5,5-tetramethyl-1,3,2-dioxaborolane**:[2] ^1H NMR (400 MHz, CD_3CN): δ 7.39 – 7.32 (m, 3H), 7.29 – 7.24 (m, 2H), 3.90 (s, 2H), 1.31 (s, 12H); ^{13}C NMR (101 MHz, CD_3CN): δ 141.8, 129.4, 127.8, 118.2, 86.2, 31.0, 24.8; ^{11}B NMR (128 MHz, CD_3CN): δ 33.4.

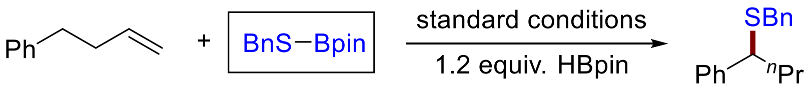

1a (2.0 equiv) **2A'** (1.0 equiv) **3a**

entry	deviation from above conditions	GC yield	rr
1	none	95%	>99:1 r.r.
2	$(MeO)_2MeSiH$ instead of HBpin	40%	>99:1 r.r.
3	1.0 equiv. HBpin	95%	>99:1 r.r.
4	**1a** : **2A'** = 1:1	84%	>99:1 r.r.
5	**1a** : **2A'** = 1:1.2	88%	>99:1 r.r.
6	**1a** : **2A'** = 1.2:1	93%	>99:1 r.r.

Supplementary Figure 38. Optimized reaction condition while using RS-Bpin as the thiolation reagent, Related to Figure 7.

Following the general procedure **A** and similar to the preparation of **3a** while using 2-(benzylthio)-4,4,5,5-tetramethyl-1,3,2-dioxaborolane (**2A'**).

Supplementary Note 23: We wondered whether the pre-generated RS−Bpin reagent could be employed directly instead of a thiol. Indeed, as shown above, changing the thiolation reagent from a thiol to RS−Bpin **2A'**, generated *in situ* from the thiol and HBpin, a competent yield of **3a** is obtained. In this case, only a stoichiometric amount of the alkene is required and the desired thiolation product could still be obtained in comparable yield (entries 3−6). For instance, **3a** is obtained in 84% yield when 1:1 stoichiometry of alkene and RS−Bpin is used and the yields are even better (88% and 93%, respectively) when a slightly excess of alkene or RS−Bpin is used (1.2 equiv.). This finding, together with the results disclosed in Supplementary Figures 28−30 (also see Figure 6 in the manuscript), suggests that the reactive intermediate of thiol is the RS−Bpin complex.

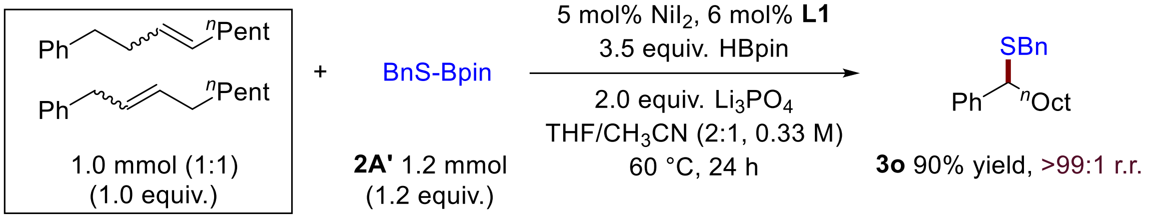

Supplementary Figure 39: Regioconvergent experiment for isomeric mixture of acyclic alkene substrates, Related to Figure 10a.

benzyl(1-phenylpentyl)sulfane (Figure 10a, **3o**). From non-3-en-1-ylbenzene (**1o**) (101.1 mg, 0.5 mmol) and non-2-en-1-ylbenzene (101.1 mg, 0.5 mmol), the title compound was prepared following the general procedure **A** using NiI_2 (16.0 mg, 0.05 mmol, 5.0 mol%), **L1** (20.0 mg, 0.06 mmol, 6.0 mol%), BnS-Bpin (300.0 mg, 1.2 mmol, 1.2 equiv.), HBpin (500 μL, 3.5 mmol, 3.5 equiv.), Li_3PO_4 (250.0 mg, 2.0 mmol, 2.0 equiv.) and THF (2.0 mL)/CH_3CN (1.0 mL). The reaction mixture was stirred at 60 °C for 24 h. The crude material was purified by flash column chromatography (using 1–2% ethyl acetate in hexanes) to provide the title compound **3o** as a colorless liquid in 90% yield (293.6 mg), >99:1 r.r..

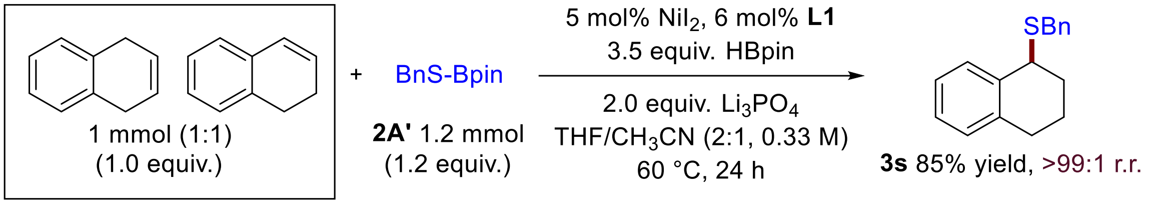

Supplementary Figure 40. Regioconvergent experiment for isomeric mixture of cyclic alkene substrates, Related to Figure 10a.

benzyl(1,2,3,4-tetrahydronaphthalen-1-yl)sulfane (Figure 10a, **3s**). From 1,4-dihydronaphthalene (**1s**) (65.0 mg, 0.5 mmol) and 1,2-dihydronaphthalene (65.0 mg, 0.5 mmol), the title compound was prepared following the general procedure **A** NiI_2 (16.0 mg, 0.05 mmol, 5.0 mol%), **L1** (20.0 mg, 0.06 mmol, 6.0 mol%), BnS-Bpin (300.0 mg, 1.2 mmol, 1.2 equiv.), HBpin (500 μL, 3.5 mmol, 3.5 equiv.), Li_3PO_4 (250.0 mg, 2.0 mmol, 2.0 equiv.) and THF (2.0 mL)/CH_3CN (1.0 mL). The reaction mixture was stirred at 60 ^{o}C for 24 h. The crude material was purified by flash column chromatography (1–2% EtOAc in hexanes) to provide the title compound **3s** as a colorless oil in 85% yield (216.0 mg), >99:1 r.r..

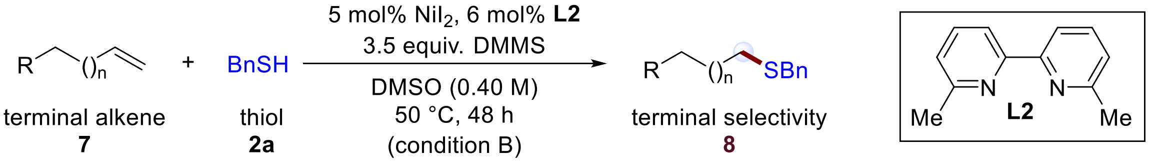

Supplementary Figure 41. NiH-catalysed *anti*-Markovnikov hydrothiolation, Related to Figure 10b.

General procedure (condition B) for the NiH-catalysed *anti*-Markovnikov hydrothiolation. To an oven-dried 8 mL screw-cap vial equipped with a magnetic stir bar was added NiI_2 (5.0 mol%) and 6,6'-dimethyl-2,2'-bipyridine (**L2**) (6.0 mol%). The vial was introduced into a nitrogen-filled glove box, anhydrous DMSO (0.5 mL) were added, and the mixture was stirred for 10 min, at which time alkene (**7**) (0.40 mmol, 2.0 equiv.), benzyl mercaptan (0.20 mmol, 1.0 equiv.), and DMMS (dimethoxy(methyl)silane,

100 μL, 0.70 mmol, 3.5 equiv.) were added to the resulting mixture in this order. The tube was sealed with a teflon-lined screw cap, removed from the glove box and stirred at 50 °C for 48 h (the mixture was stirred at 750 rpm). After the reaction was complete, the reaction mixture was directly filtered through a short pad of silica gel (using ethyl acetate in hexanes) to give the crude product. *n*-Tetradecane (20 μL) was added as an internal standard for GC analysis. 1,1,2,2-Tetrachloroethane (10.5 μL, 0.10 mmol) was added as internal standard for ^{1}H NMR analysis of the crude material. The product was purified by chromatography on silica gel for each substrate. The yields reported are the average of at least two experiments, unless otherwise indicated.

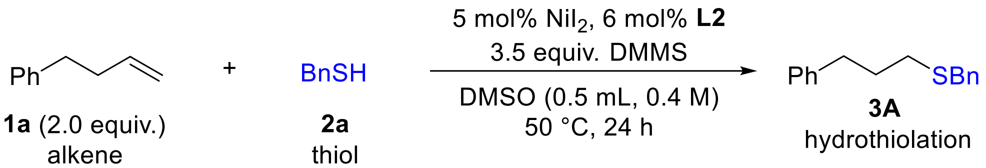

1a (2.0 equiv.) alkene

2a thiol

3A hydrothiolation

entry	deviation from standard conditions	yield of **3A** (%)[a]	r.r.[b]
1	none	88(85)	>99:1
2	w/o NiI_2	25	>99:1
3	w/o **L2**	16	>99:1
4	w/o DMMS	17	>99:1
5	w/o NiI_2 and **L2**	25	>99:1
6	THF instead of DMSO	0	-

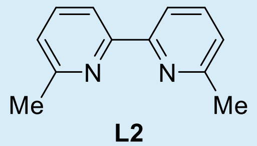

L2

Supplementary Figure 42. Control experiments of *anti*-Markovnikov thiolation reaction in nitrogen atmosphere, Related to Figure 10b. [a]Yields were determined by GC using *n*-tetradecane as the internal standard. [b]r.r. refers to regioisomeric ratio, represents the ratio of the **3A** product to the sum of all other isomers as determined by GC analysis.

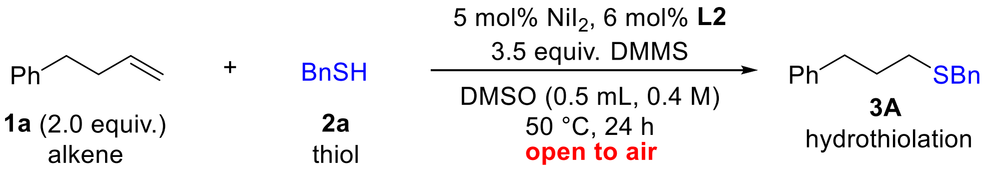

entry	deviation from standard conditions	yield of **3A** (%)[a]	r.r.[b]
1	none	14	>99:1
2	w/o NiI_2	10	>99:1
3	w/o **L2**	8	>99:1
4	w/o DMMS	20	>99:1
5	w/o NiI_2 and **L2**	8	>99:1
6	THF instead of DMSO	0	-

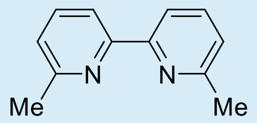

L2

Supplementary Figure 43. Control experiments of *anti*-Markovnikov thiolation reaction in air atmosphere, Related to Figure 10b. Yield and r.r. are as defined in Supplementary Figure 42.

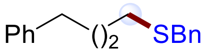

8a

benzyl(4-phenylbutyl)sulfane (Figure 10b, **8a**): From 4-phenyl-1-butene (**1a**) (52.8 mg, 0.40 mmol, 2.0 equiv.), the title compound was prepared following the above general procedure (condition B) using NiI_2 (3.2 mg, 0.010 mmol, 5.0 mol%), **L2** (2.2 mg, 0.012 mmol, 6.0 mol%), benzyl mercaptan **2a** (25.0 mg, 0.20 mmol, 1.0 equiv.), DMMS (100 μL, 0.70 mmol, 3.5 equiv.) and DMSO (0.50 mL). The reaction mixture was stirred at 50 °C for 48 h. The crude material was purified by flash column chromatography (using 1–2% ethyl acetate in hexanes) to provide the title compound **8a** as a colorless liquid in 85% yield (43.5 mg), >99:1 r.r.. IR (neat, cm^{-1}): 3026, 2929, 2855, 1495, 1453, 1071, 1029, 745, 698; ^1H NMR (500 MHz, $CDCl_3$): δ 7.35 – 7.26 (m, 7H), 7.22 – 7.17 (m, 3H), 3.71 (s, 2H), 2.61 (t, *J* = 7.8 Hz, 2H), 2.45 (t, *J* = 7.3 Hz, 2H), 1.74 – 1.68 (m, 2H), 1.65 – 1.60 (m, 2H); ^{13}C NMR (126 MHz, $CDCl_3$): δ 142.3, 138.7, 129.0, 128.6, 128.5, 128.4, 127.0, 125.9, 36.4, 35.6, 31.3, 30.6, 28.8; HRMS (ESI) calcd. for $C_{17}H_{21}S$ $[M+H]^+$ *m/z* 257.1358, found 257.1353.

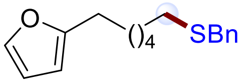
8b

2-(6-(benzylthio)hexyl)furan (Figure 10b, **8b**): From 2-(hex-5-en-1-yl)furan (**7b**) (60.0 mg, 0.40 mmol, 2.0 equiv.), the title compound was prepared following the above general procedure (condition B) using NiI_2 (3.2 mg, 0.010 mmol, 5.0 mol%), **L2** (2.2 mg, 0.012 mmol, 6.0 mol%), benzyl mercaptan **2a** (25.0 mg, 0.20 mmol, 1.0 equiv.), DMMS (100 μL, 0.70 mmol, 3.5 equiv.) and DMSO (0.50 mL). The reaction mixture was stirred at 50 ^{o}C for 48 h. The crude material was purified by flash column chromatography (using 1–2% ethyl acetate in hexanes) to provide the title compound **8b** as a colorless liquid in 62% yield (34.0 mg), >99:1 r.r.. IR (neat, cm^{-1}): 2927, 2855, 1598, 1454, 1146, 1008, 913, 729, 700; ^{1}H NMR (500 MHz, $CDCl_3$): δ 7.35 – 7.33 (m, 4H), 7.32 – 7.31 (m, 1H), 6.30 – 6.29 (m, 1H), 5.99 – 5.98 (m, 1H), 3.73 (s, 2H), 2.62 (t, *J* = 7.5 Hz, 2H), 2.43 (t, *J* = 7.3 Hz, 2H), 1.67 – 1.61 (m, 2H), 1.60 – 1.55 (m, 2H), 1.43 – 1.27 (m, 4H); ^{13}C NMR (126 MHz, $CDCl_3$): δ 156.5, 140.8, 138.8, 129.0, 128.6, 127.0, 110.2, 104.7, 36.4, 31.4, 29.2, 28.8, 28.7, 28.0; HRMS (ESI) calcd. for $C_{17}H_{23}OS$ $[M+H]^+$ *m/z* 275.1464, found 275.1461.

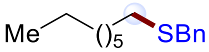
8c

benzyl(octyl)sulfane (Figure 10b, **8c**): From oct-1-ene (**7c**) (44.9 mg, 0.40 mmol, 2.0 equiv.), the title compound was prepared following the above general procedure (condition B) using NiI_2 (3.2 mg, 0.010 mmol, 5.0 mol%), **L2** (2.2 mg, 0.012 mmol, 6.0 mol%), benzyl mercaptan **2a** (25.0 mg, 0.20 mmol, 1.0 equiv.), DMMS (100 μL, 0.70 mmol, 3.5 equiv.) and DMSO (0.50 mL). The reaction mixture was stirred at 50 ^{o}C for 48 h. The crude material was purified by flash column chromatography (using 1–2% ethyl acetate in hexanes) to provide the title compound **8c** as a colorless liquid in 77% yield (49.4 mg), >99:1 r.r.. IR (neat, cm^{-1}): 2954, 2923, 2853, 1494, 1454, 1420, 767, 698; ^{1}H NMR (500 MHz, $CDCl_3$): δ 7.34 – 7.33 (m, 4H), 7.28 – 7.24 (m, 1H), 3.73 (s, 2H), 2.43 (t, *J* = 7.3 Hz, 2H), 1.61 – 1.55 (m, 2H), 1.37 – 1.28 (m, 10H), 0.91 (t, *J* = 7.0 Hz, 3H); ^{13}C NMR (126 MHz, $CDCl_3$): δ 138.8, 129.0, 128.6, 127.0, 36.4, 32.0, 31.5, 29.4, 29.3,

29.3, 29.0, 22.8, 14.3; HRMS (ESI) calcd. for $C_{15}H_{24}SNa$ $[M+Na]^+$ *m/z* 259.1491, found 259.1497.

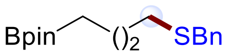

8d

2-(4-(benzylthio)butyl)-4,4,5,5-tetramethyl-1,3,2-dioxaborolane (Figure 10b, **8d**): From 2-(but-3-en-1-yl)-4,4,5,5-tetramethyl-1,3,2-dioxaborolane (**7d**) (72.9 mg, 0.40 mmol, 2.0 equiv.), the title compound was prepared following the above general procedure (condition B) using NiI_2 (3.2 mg, 0.010 mmol, 5.0 mol%), **L2** (2.2 mg, 0.012 mmol, 6.0 mol%), benzyl mercaptan **2a** (25.0 mg, 0.20 mmol, 1.0 equiv.), DMMS (100 μL, 0.70 mmol, 3.5 equiv.) and DMSO (0.50 mL). The reaction mixture was stirred at 50 ^{o}C for 48 h. The crude material was purified by flash column chromatography (using 1–2% ethyl acetate in hexanes) to provide the title compound **8d** as a colorless liquid in 71% yield (43.5 mg), >99:1 r.r.. IR (neat, cm^{-1}): 2977, 2926, 1372, 1320, 1144, 968, 846, 766, 748; ^{1}H NMR (400 MHz, $CDCl_3$): δ 7.24 – 7.19 (m, 3H), 7.18 – 7.13 (m, 2H), 3.62 (s, 2H), 2.33 (t, J = 7.4 Hz, 2H), 1.55 – 1.47 (m, 2H), 1.43 – 1.36 (m, 2H), 1.17 (s, 12H), 0.69 (t, J = 7.6 Hz, 2H); ^{13}C NMR (101 MHz, $CDCl_3$): δ 138.8, 129.0, 128.6, 127.0, 83.1, 36.3, 32.0, 31.2, 25.0, 23.6; HRMS (ESI) calcd. for $C_{17}H_{17}BO_2S$ $[M+H]^+$ *m/z* 307.1898, found 307.1895.

Supplementary Note 24. Spectroscopic Data (GC Traces)

1) n-Tetradecane (t_r = 2.6 min in Agilent 7890B; *t_r = 2.9 min in Shimadazu Nexis GC-2030) was used as internal standard for GC yield.*

2) GC analysis was performed on Agilent 7890B gas chromatograph or Shimadazu Nexis GC-2030 gas chromatograph with an FID detector using a J & W DB-1 column (10 m, 0.1 mm I.D.). GC-MS analysis was performed on an Agilent 7890B gas chromatograph with 5977A MSD mass spectrum using an HP-5 MS column (30 m, 0.25 mm I.D.).

3) GC method: 100 method starts at 100 °C holds the oven at this temperature for 1 minute, then ramp of 50 °C/min till 250 °C and hold the oven at this temperature for 3 minutes (or 5 minutes for 100B method, or 16 minutes for 100C method).

4) r.r. refers to regioisomeric ratio, represents the ratio of major isomer product to the sum of all other isomers as determined by GC, all isomers' peaks were confirmed by GC-MS analysis.

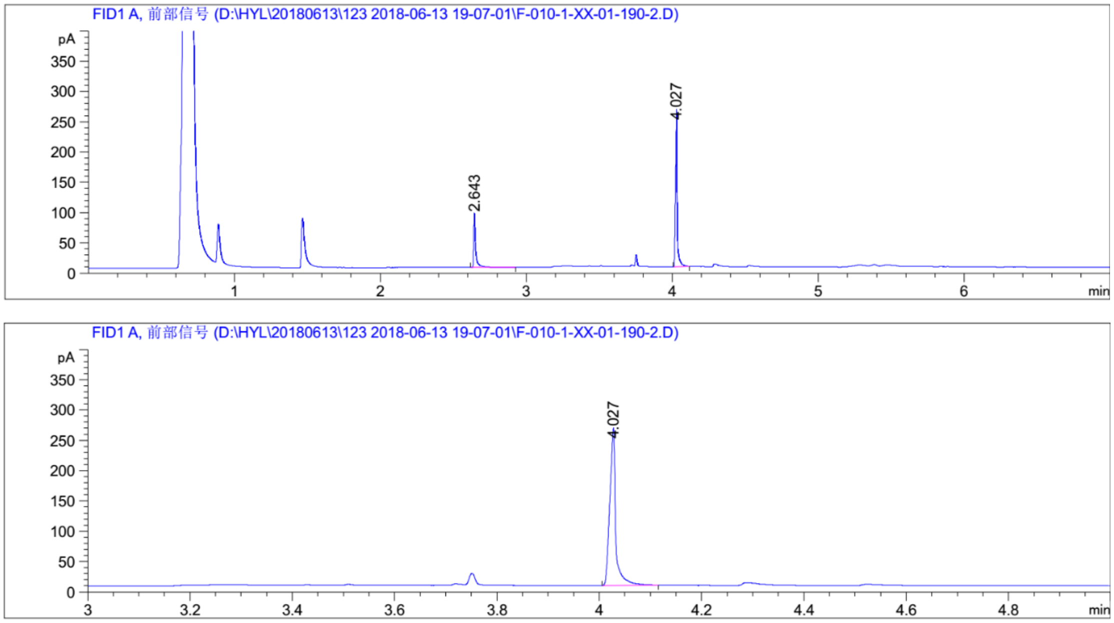

Supplementary Figure 44. GC spectra of compound **3a**, Related to Figure 3.

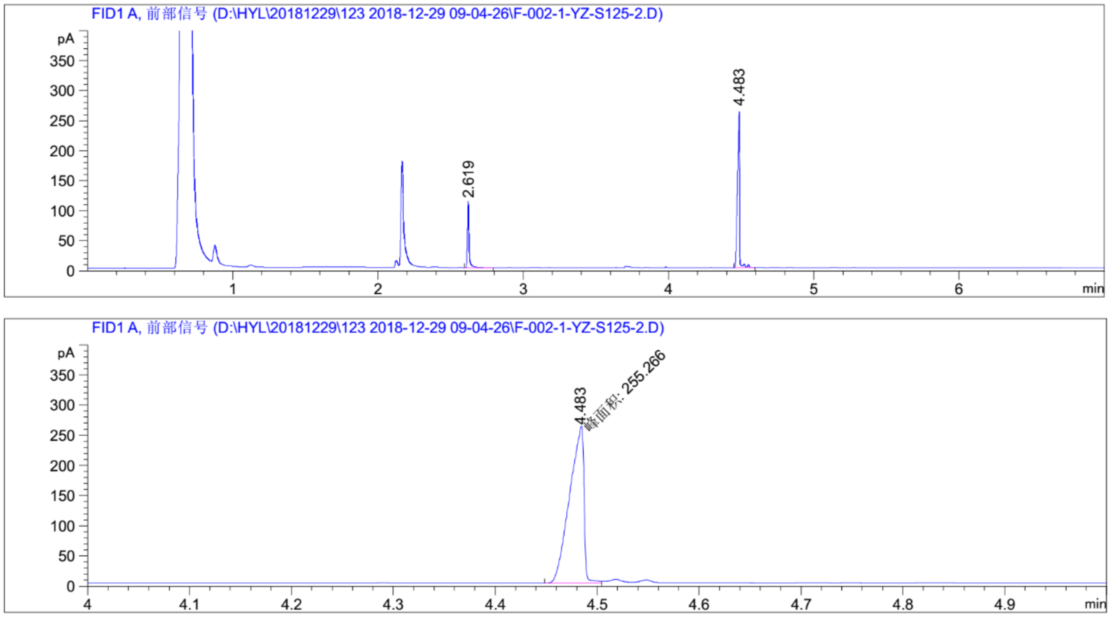

Supplementary Figure 45. GC spectra of compound **3b**, Related to Figure 3.

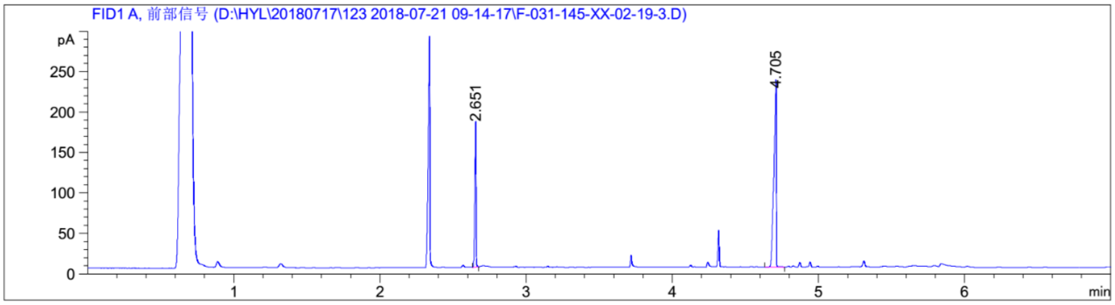

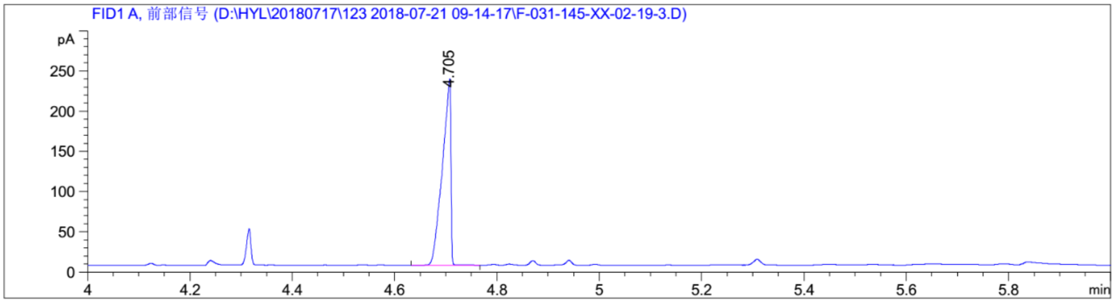

Supplementary Figure 46. GC spectra of compound **3c**, Related to Figure 3.

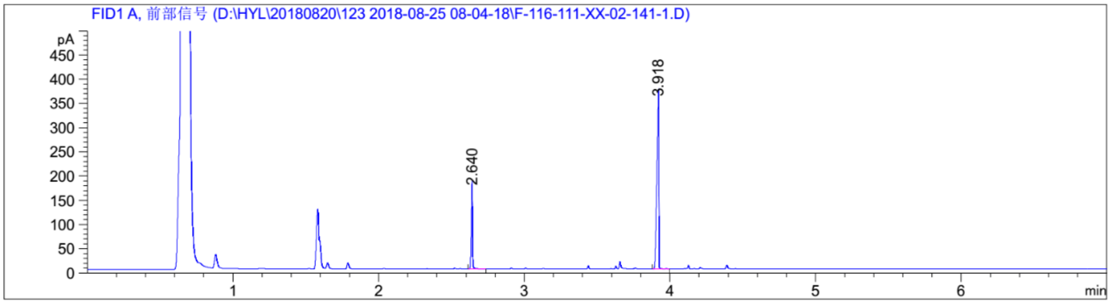

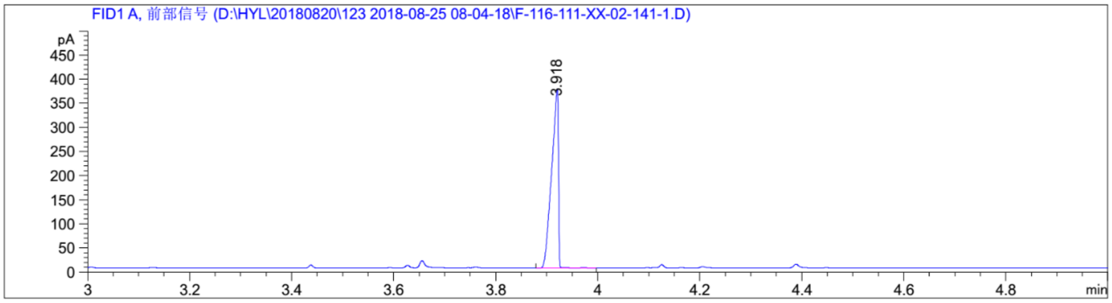

Supplementary Figure 47. GC spectra of compound **3d**, Related to Figure 3.

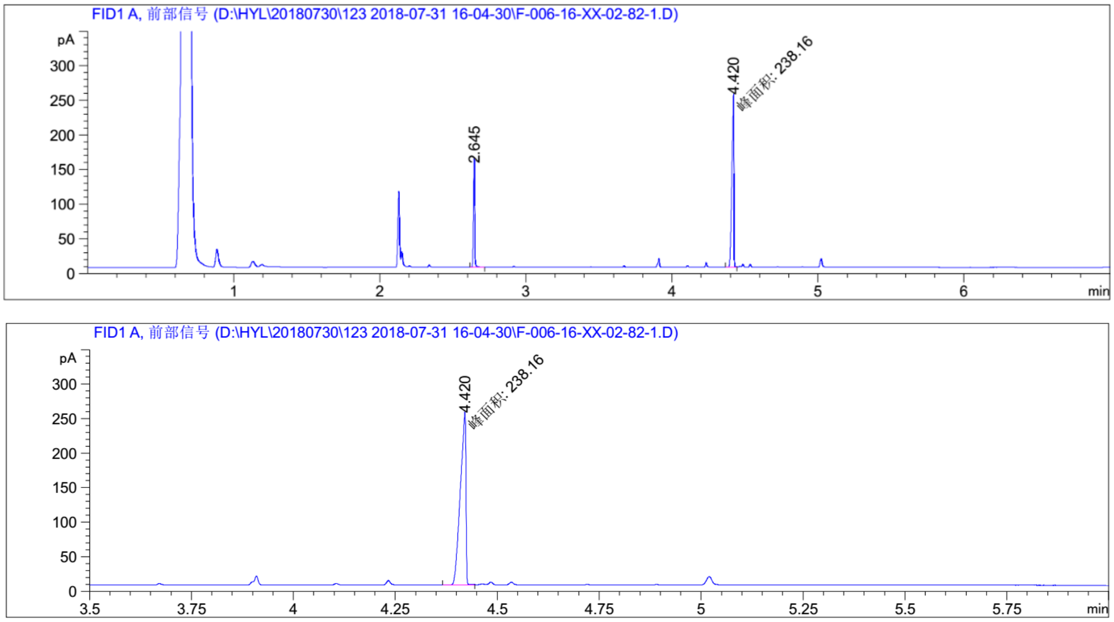

Supplementary Figure 48. GC spectra of compound **3e**, Related to Figure 3.

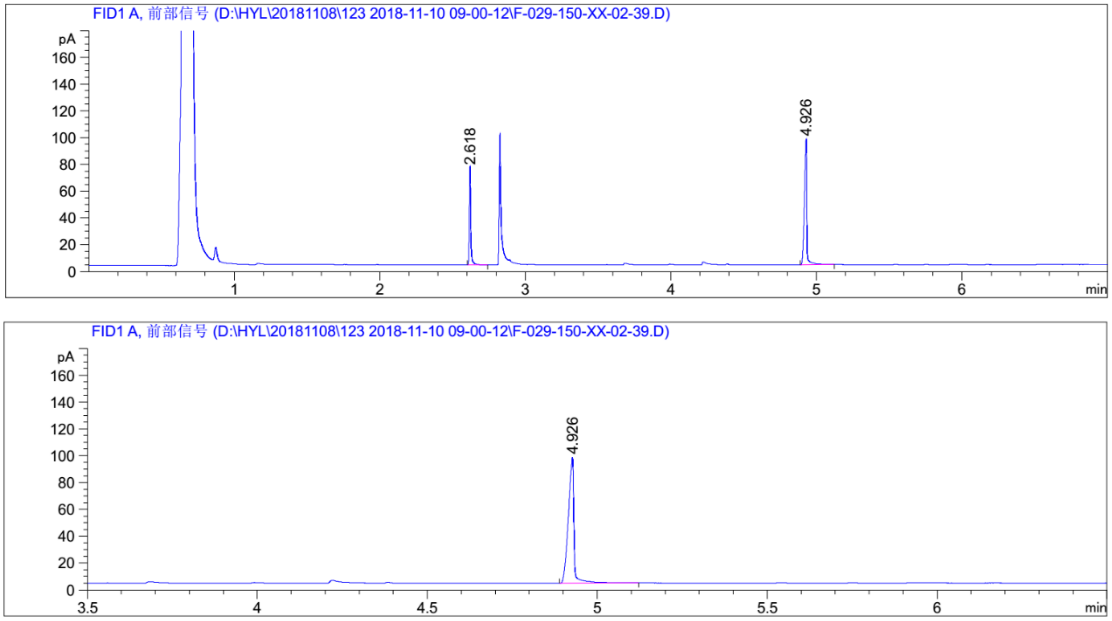

Supplementary Figure 49. GC spectra of compound **3f**, Related to Figure 3.

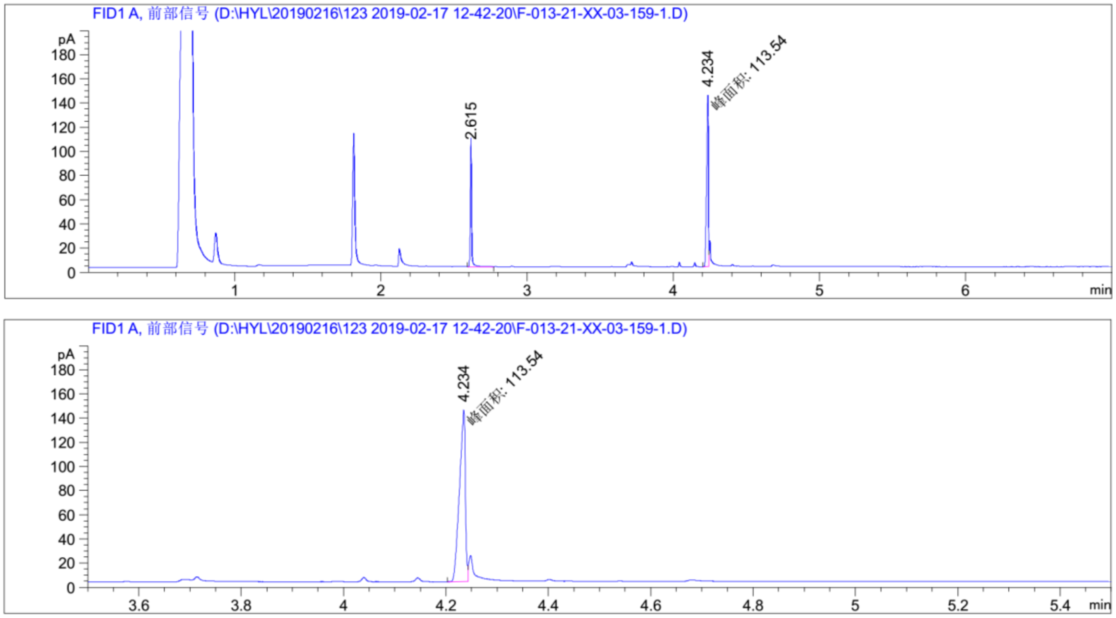

Supplementary Figure 50. GC spectra of compound **3g**, Related to Figure 3.

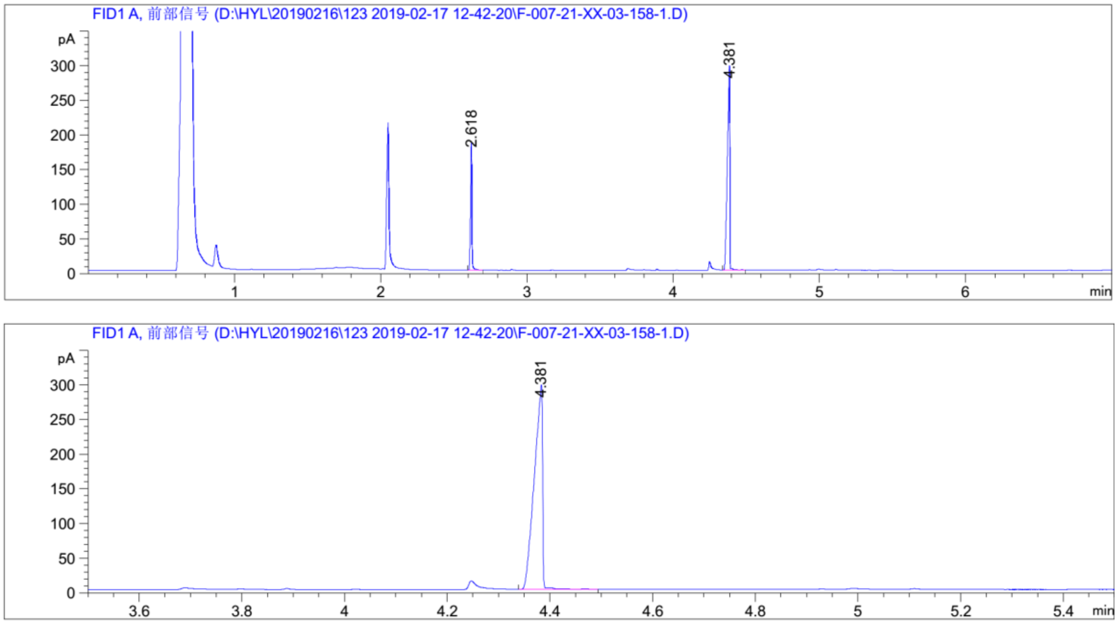

Supplementary Figure 51. GC spectra of compound **3h**, Related to Figure 3.

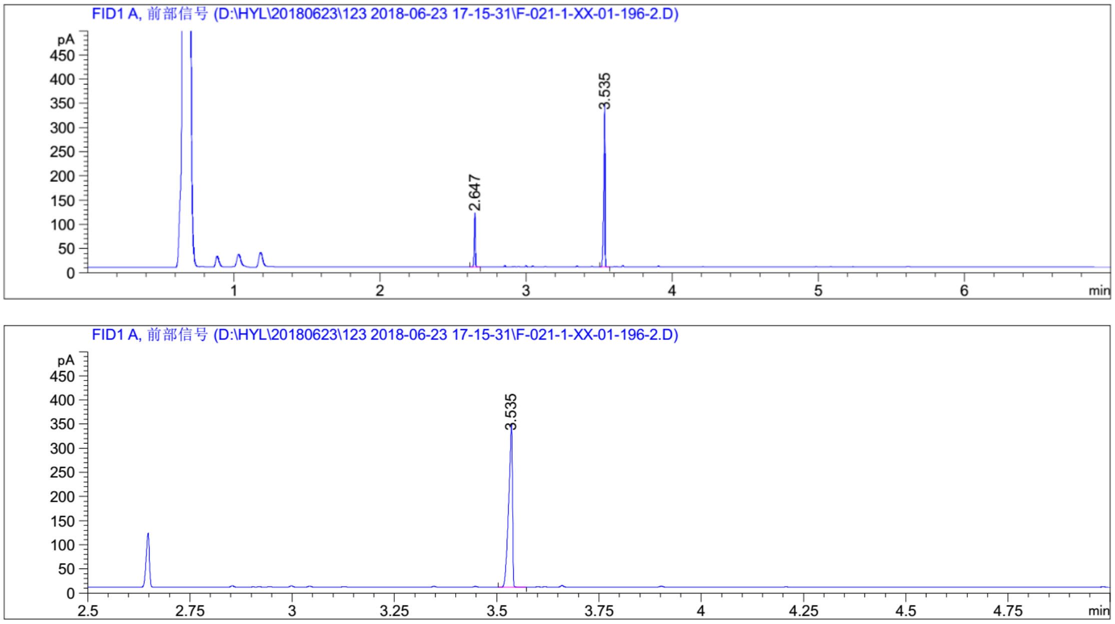

Supplementary Figure 52. GC spectra of compound **3i**, Related to Figure 3.

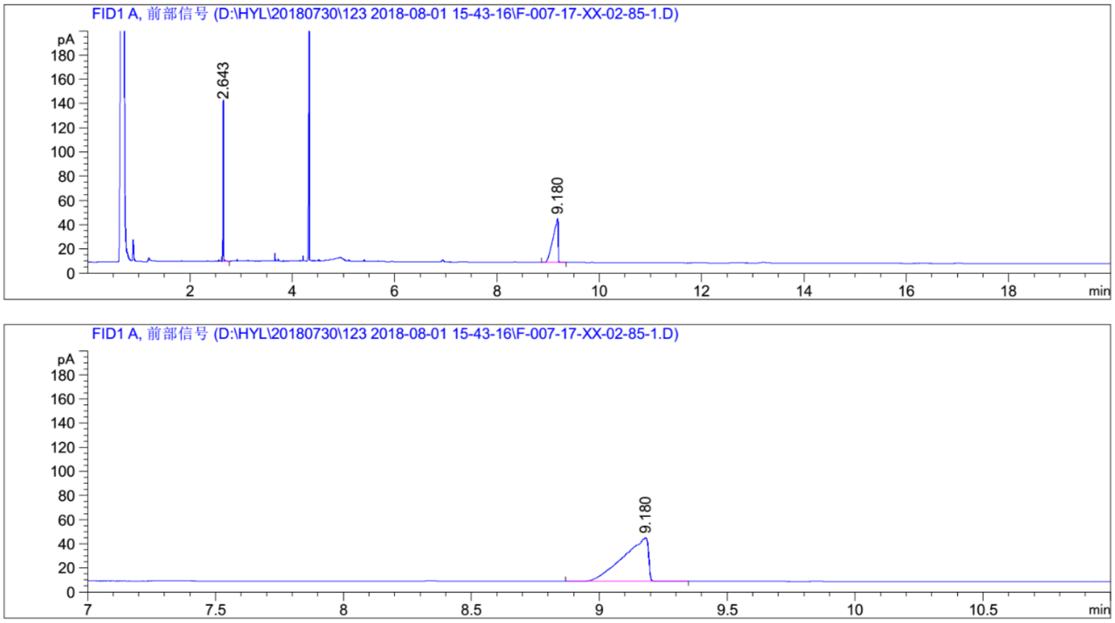

Supplementary Figure 53. GC spectra of compound **3l**, Related to Figure 3.

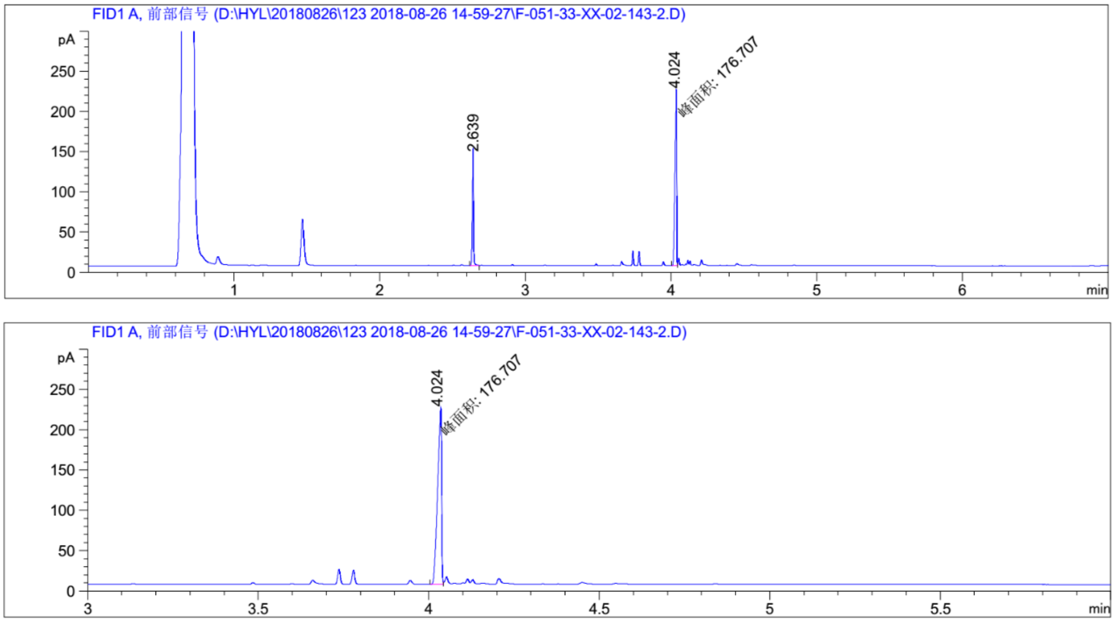

Supplementary Figure 54. GC spectra of compound **3m**, Related to Figure 3.

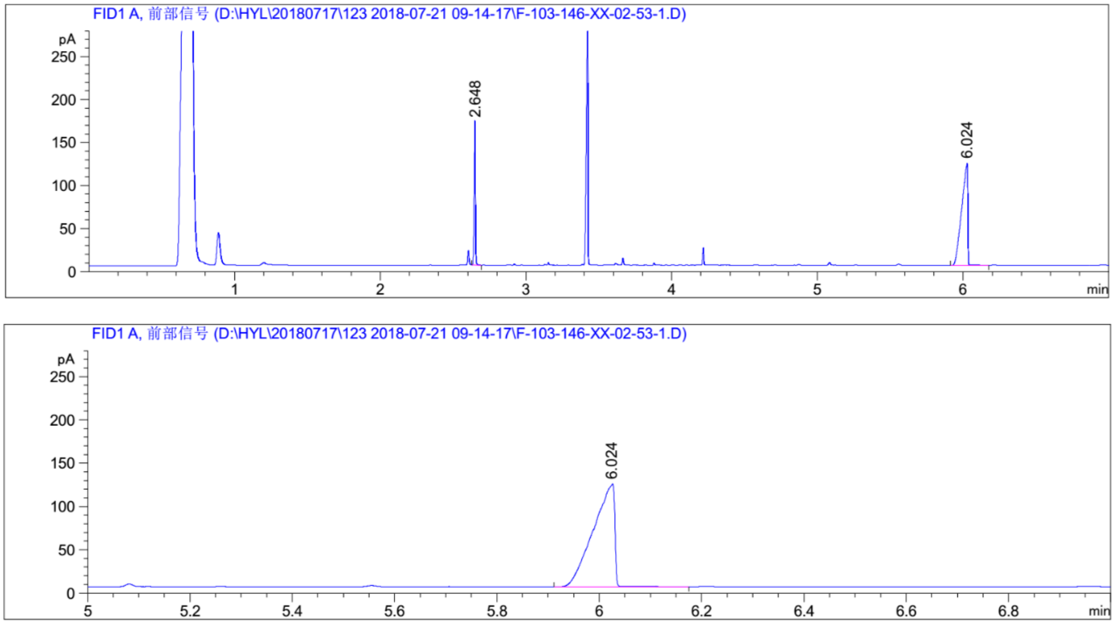

Supplementary Figure 55. GC spectra of compound **3n**, Related to Figure 3.

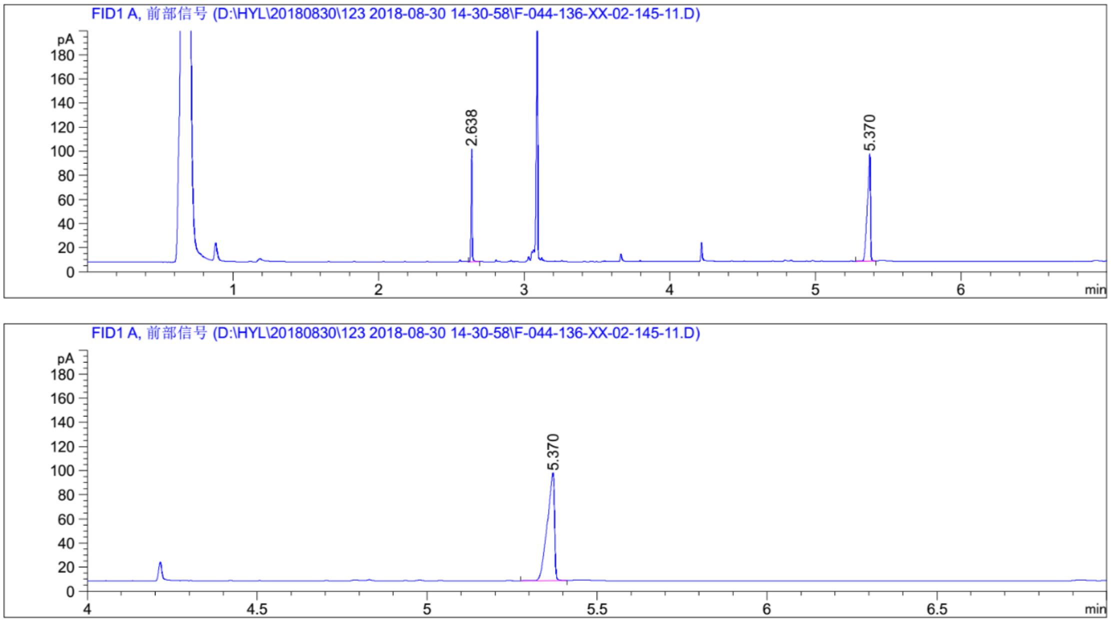

Supplementary Figure 56. GC spectra of compound **3o**, Related to Figure 3.

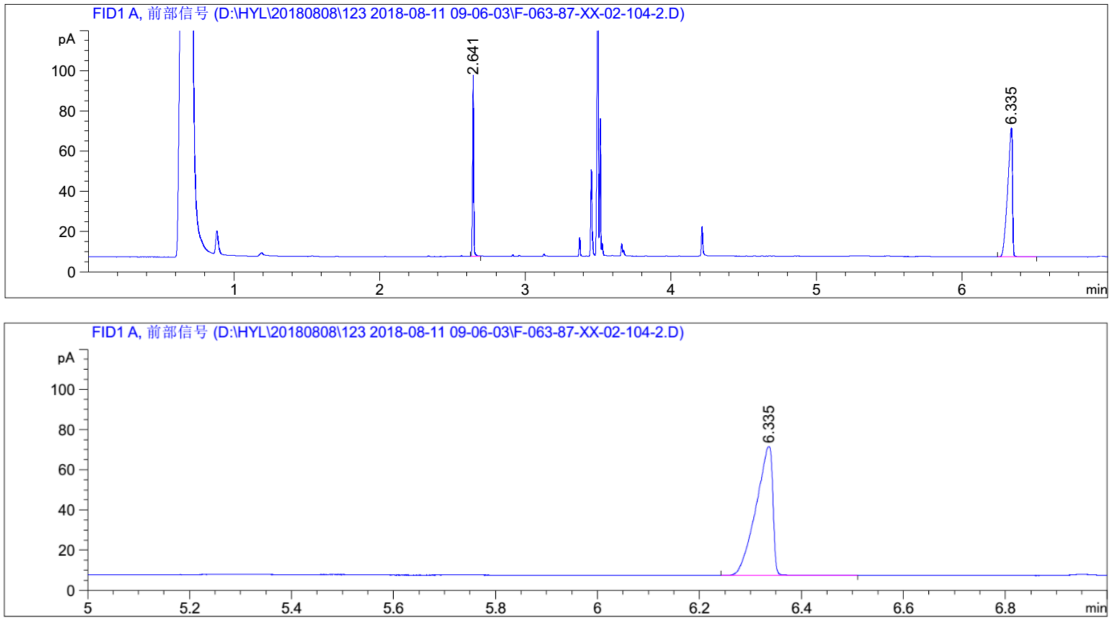

Supplementary Figure 57. GC spectra of compound **3p**, Related to Figure 3.

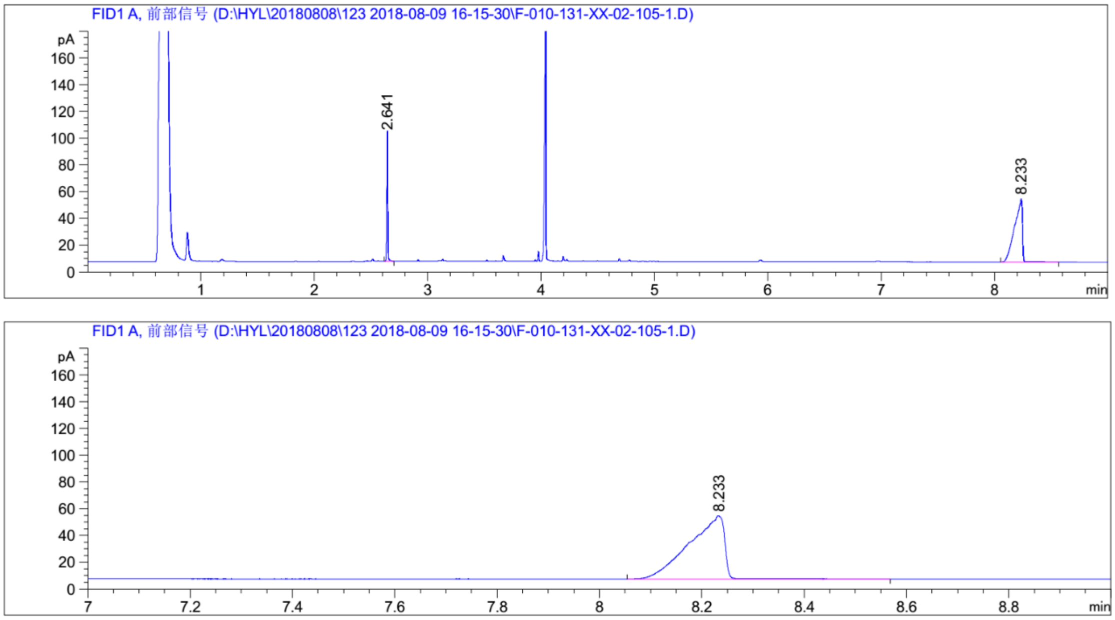

Supplementary Figure 58. GC spectra of compound **3q**, Related to Figure 3.

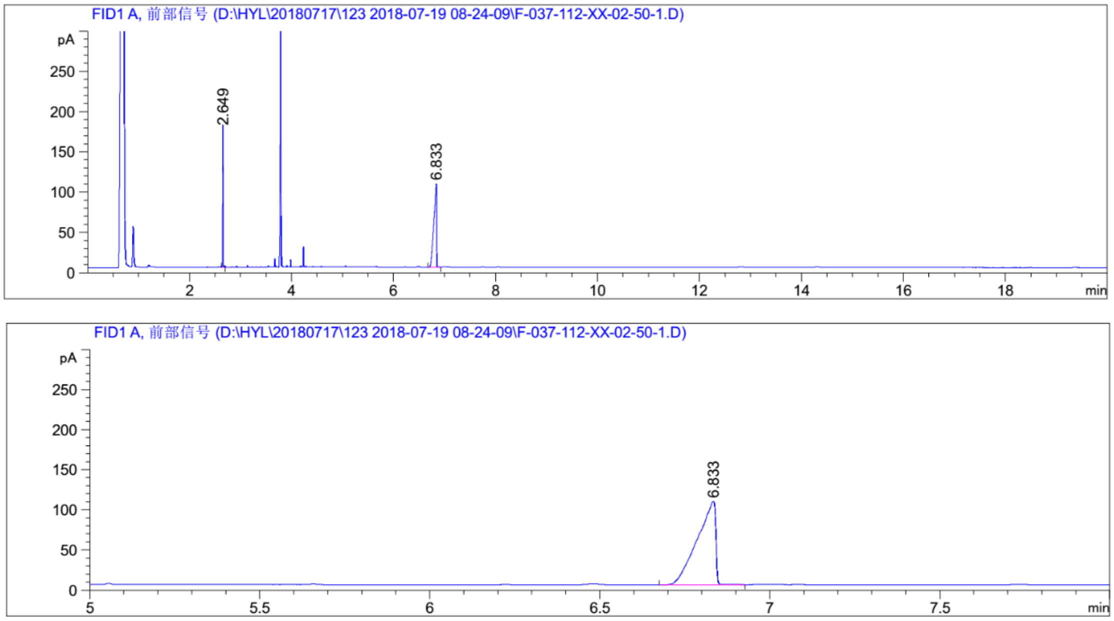

Supplementary Figure 59. GC spectra of compound **3r**, Related to Figure 3.

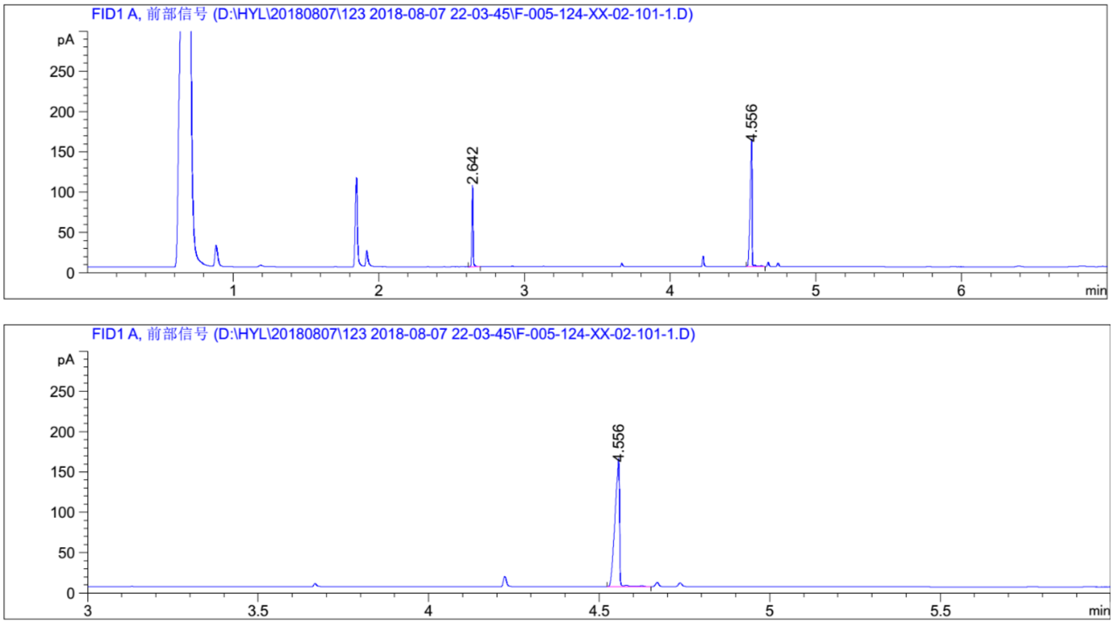

Supplementary Figure 60. GC spectra of compound **3s**, Related to Figure 3.

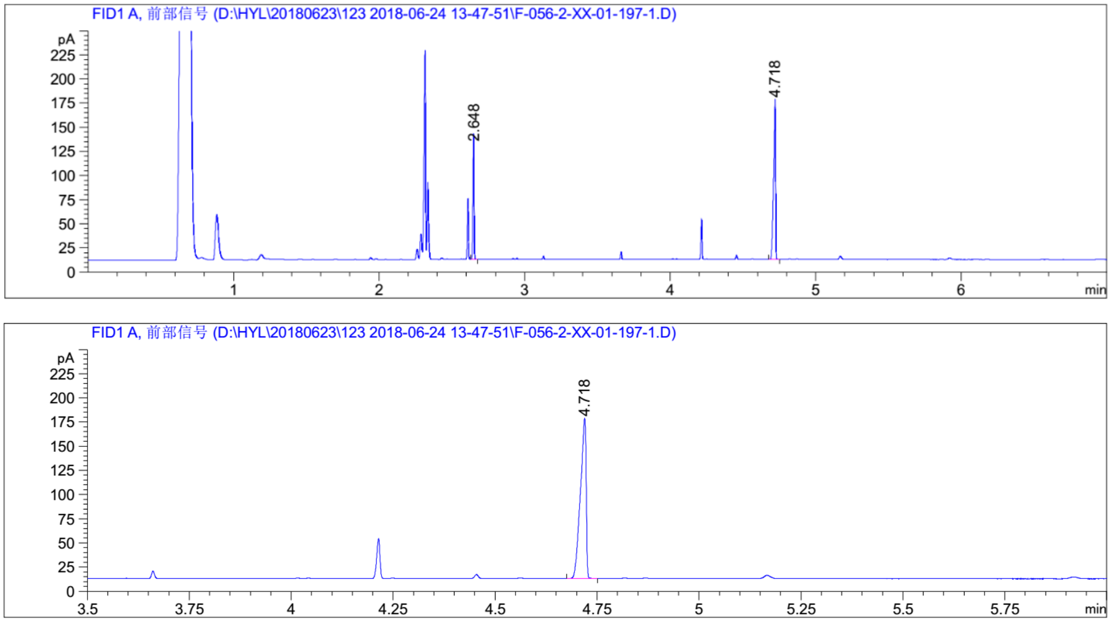

Supplementary Figure 61. GC spectra of compound **3t**, Related to Figure 3.

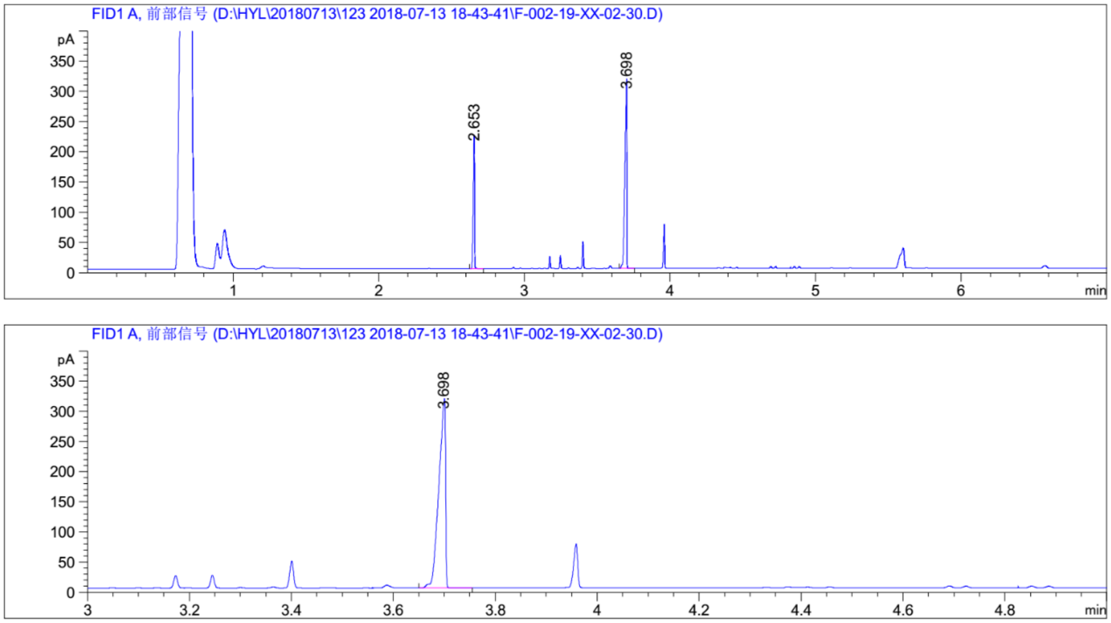

Supplementary Figure 62. GC spectra of compound **3u**, Related to Figure 3.

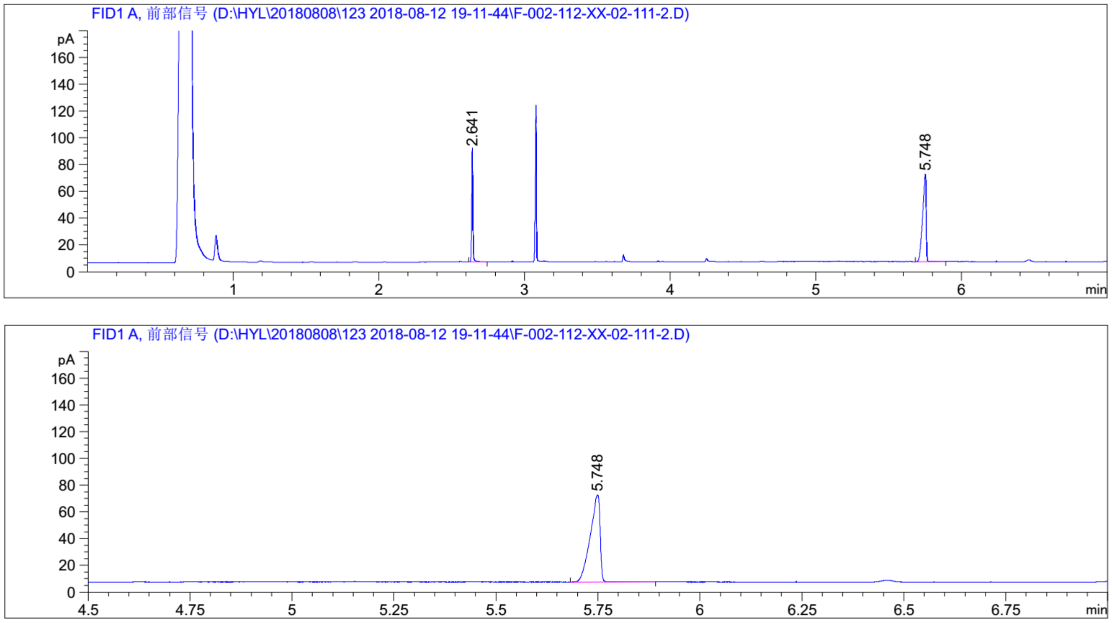

Supplementary Figure 63. GC spectra of compound **3v**, Related to Figure 3.

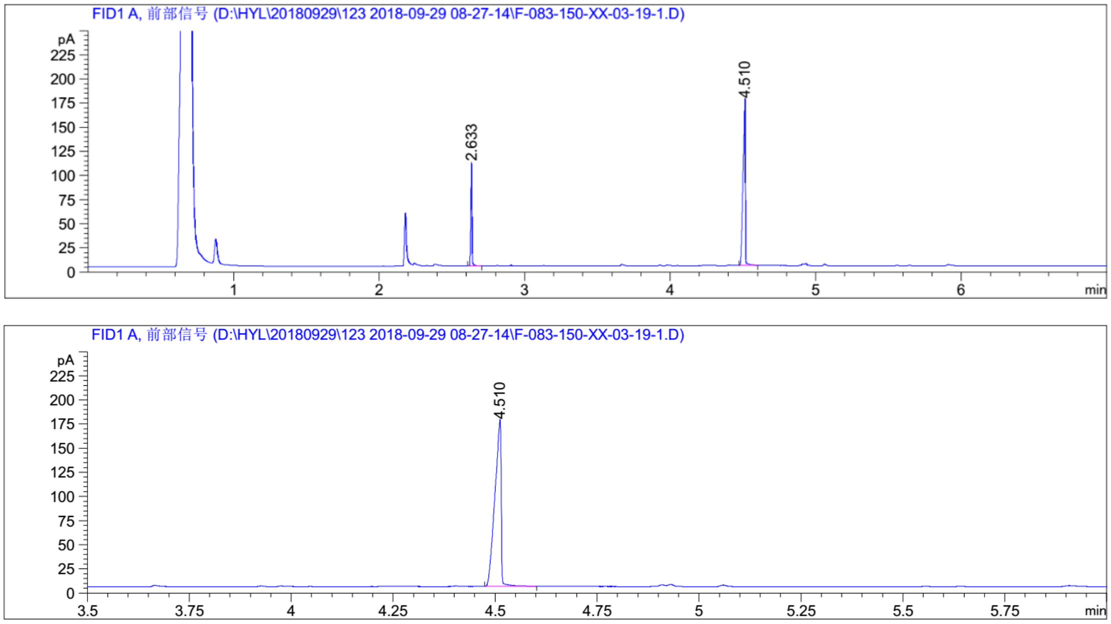

Supplementary Figure 64. GC spectra of compound **3w**, Related to Figure 3.

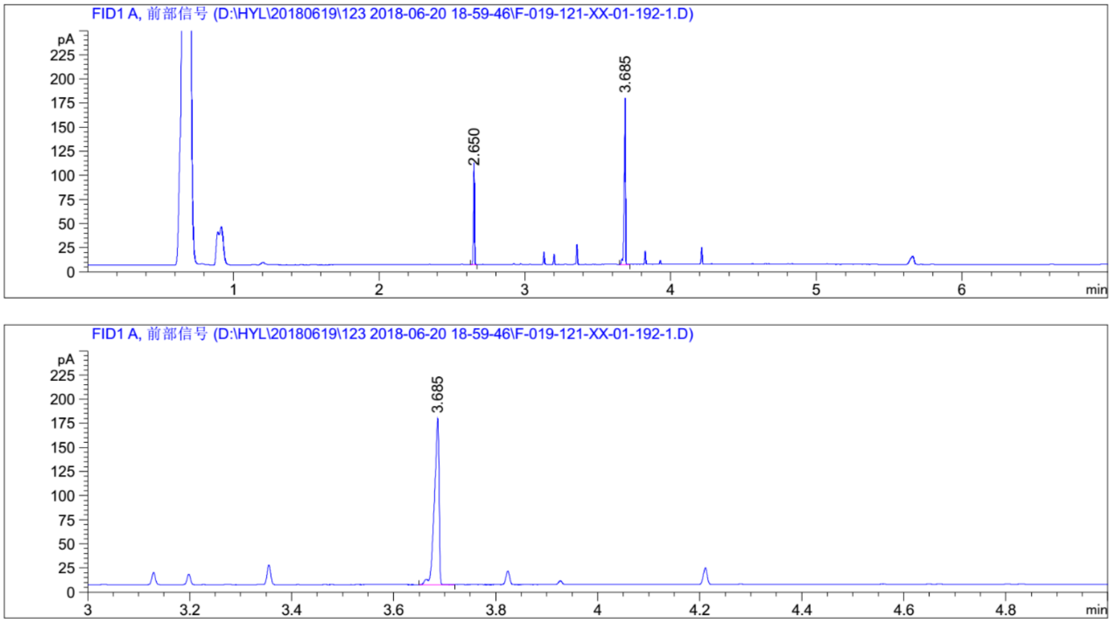

Supplementary Figure 65. GC spectra of compound **3x**, Related to Figure 3.

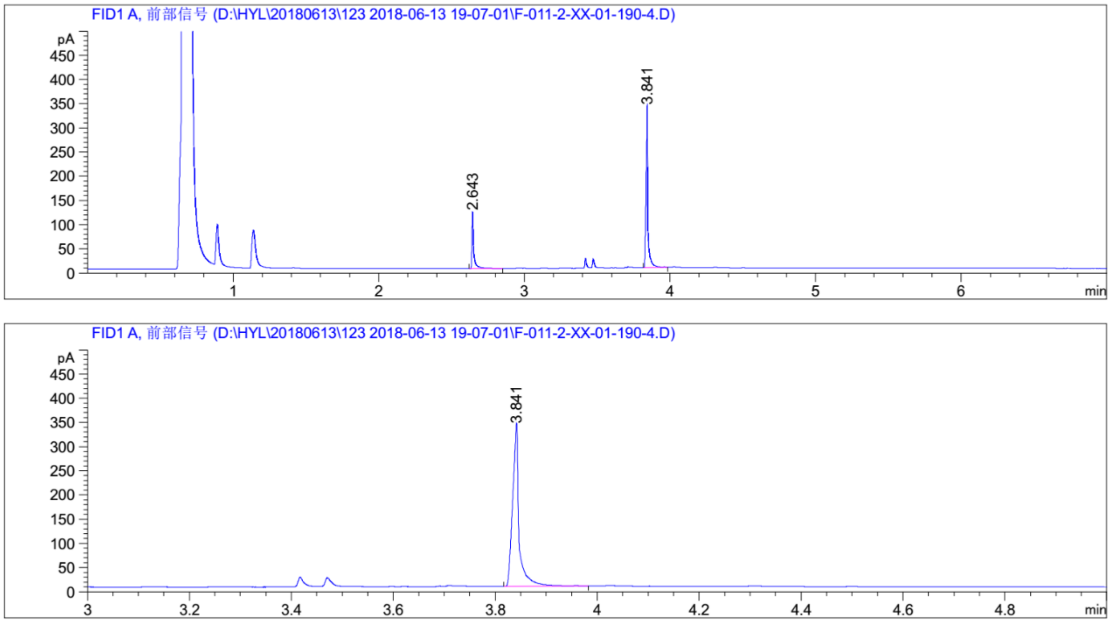

Supplementary Figure 66. GC spectra of compound **3y**, Related to Figure 3.

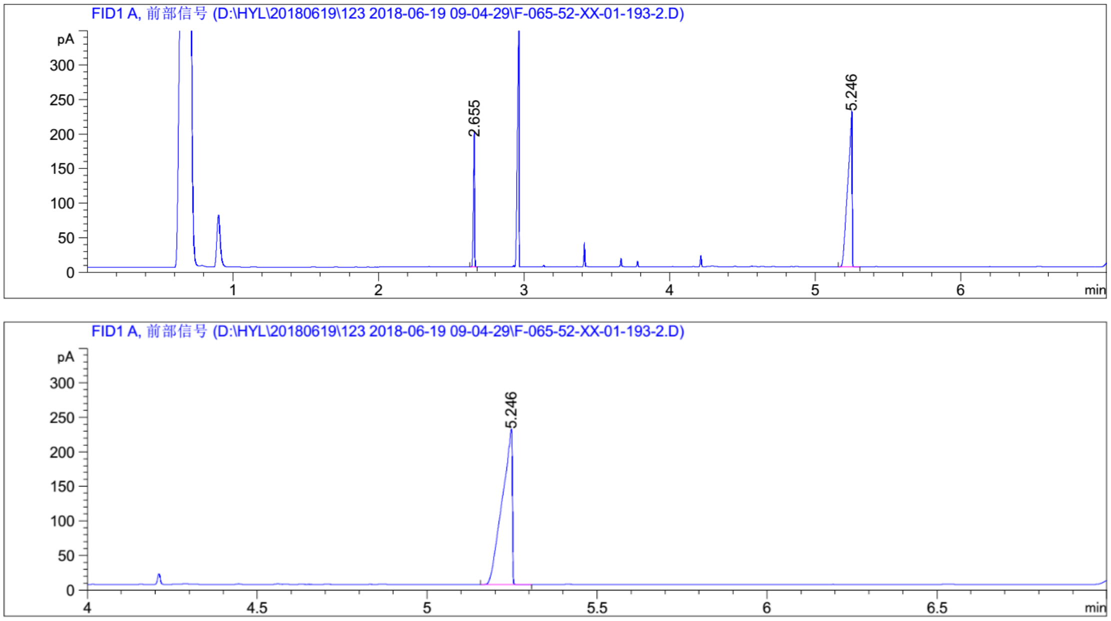

Supplementary Figure 67. GC spectra of compound **3z**, Related to Figure 3.

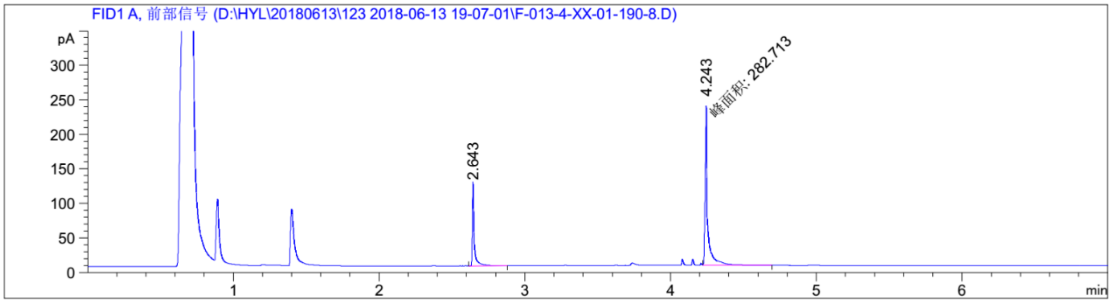

Supplementary Figure 68. GC spectra of compound **3a'**, Related to Figure 3.

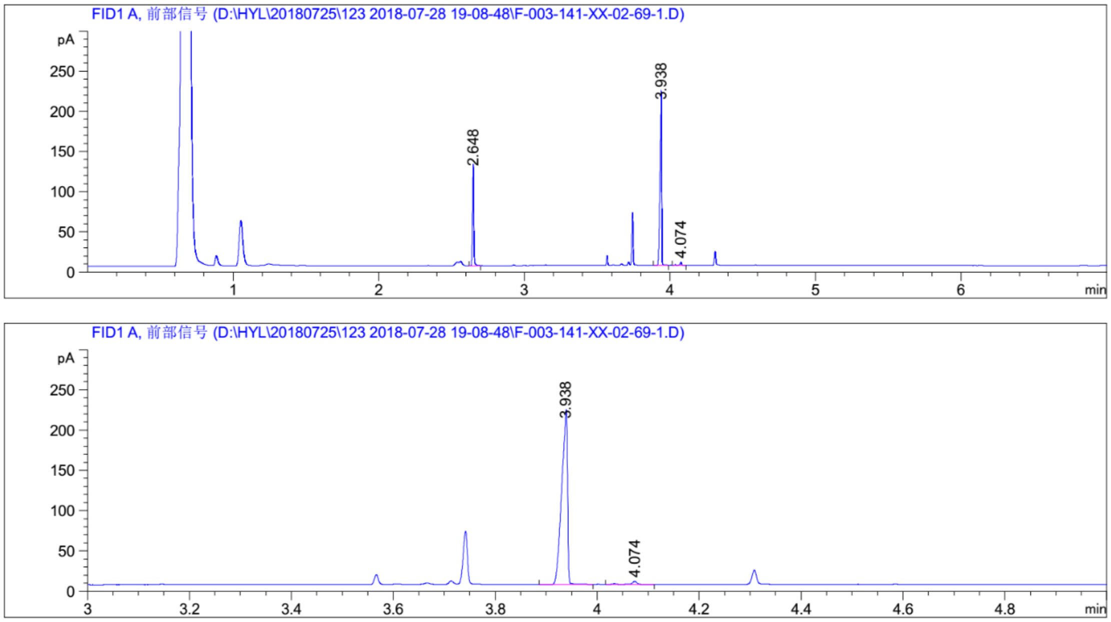

峰 #	保留时间 [min]	类型	峰宽 [min]	峰面积 [pA*s]	峰高 [pA]	峰面积 %
1	3.938	BB	0.0133	172.85965	200.43442	98.35989
2	4.074	BB	0.0115	2.88236	3.70472	1.64011

Supplementary Figure 69. GC spectra of compound **3b'**, Related to Figure 3.

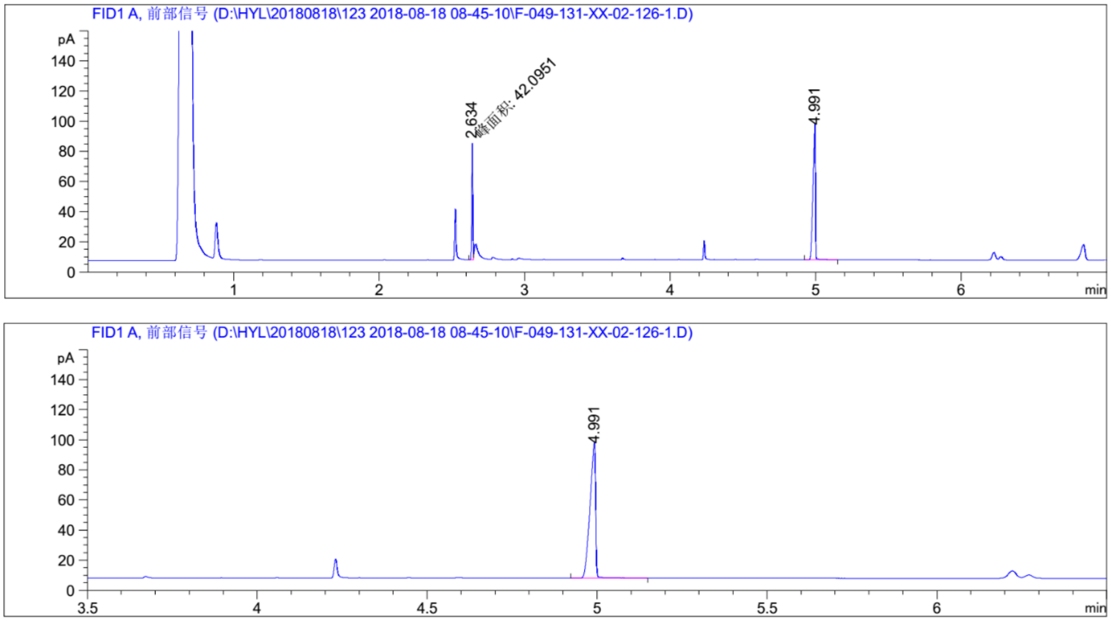

Supplementary Figure 70. GC spectra of compound **3c'**, Related to Figure 3.

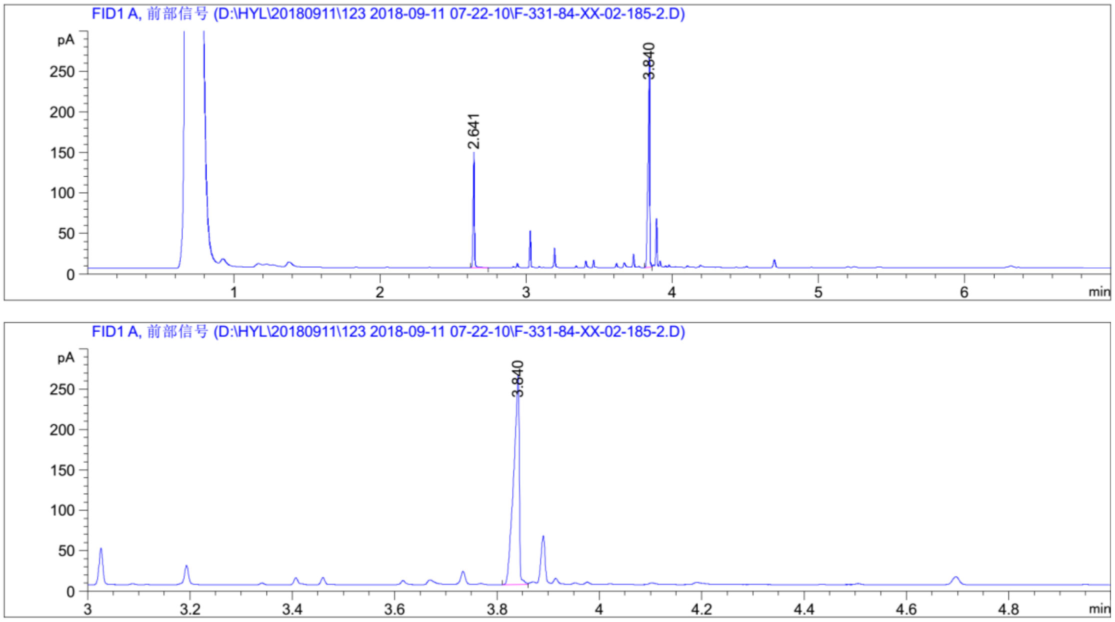

Supplementary Figure 71. GC spectra of compound **3d'**, Related to Figure 3.

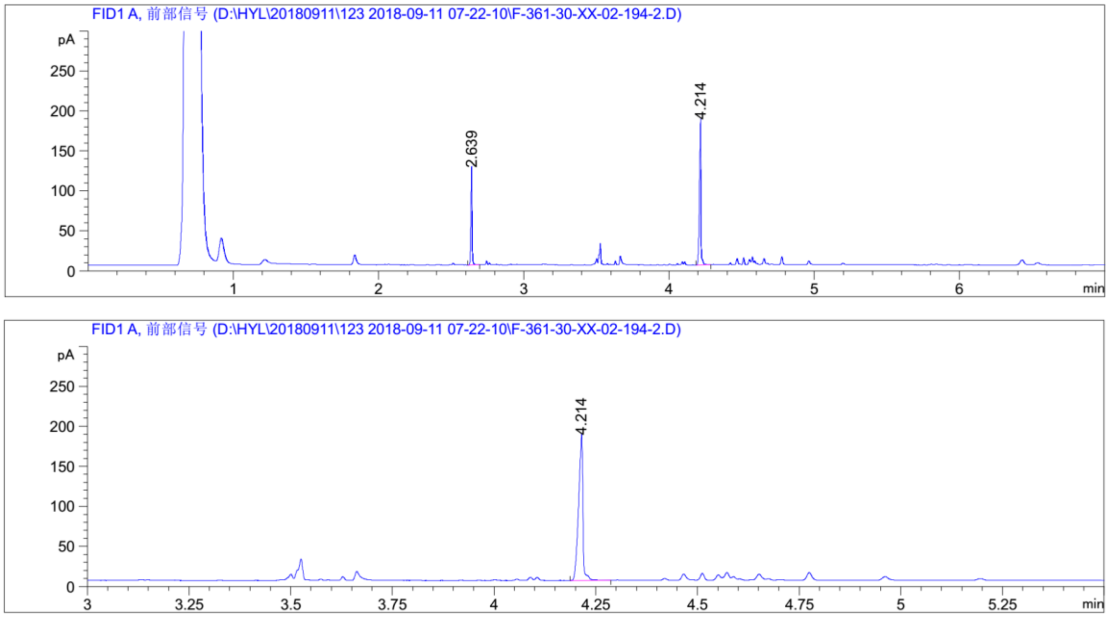

Supplementary Figure 72. GC spectra of compound **3e'**, Related to Figure 3.

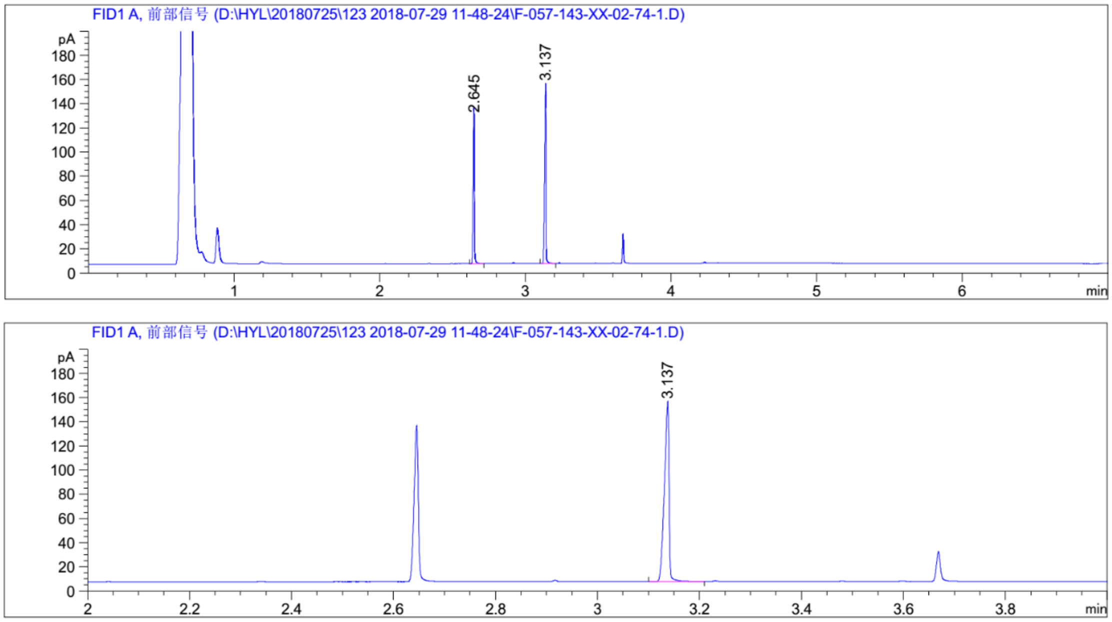

Supplementary Figure 73. GC spectra of compound **3f'**, Related to Figure 3.

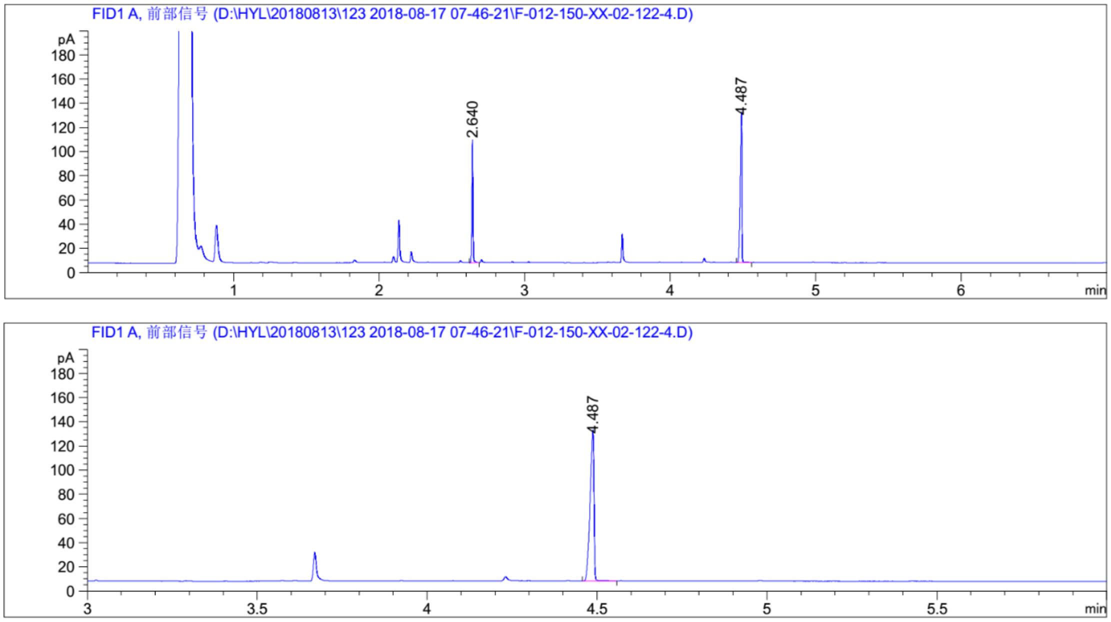

Supplementary Figure 74. GC spectra of compound **3g'**, Related to Figure 3.

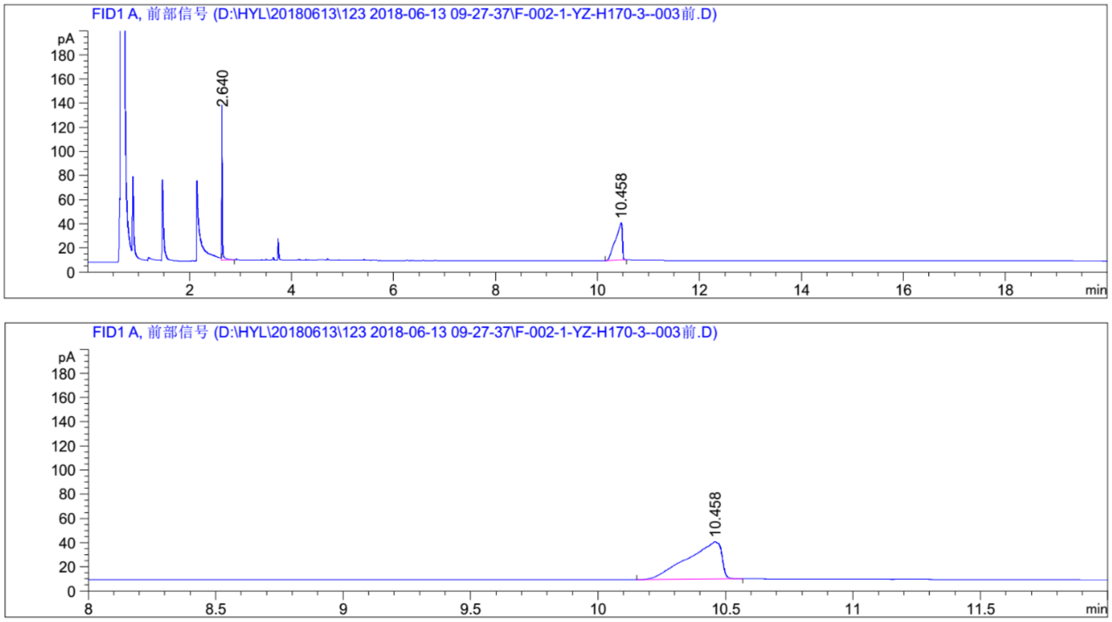

Supplementary Figure 75. GC spectra of compound **4b**, Related to Figure 4.

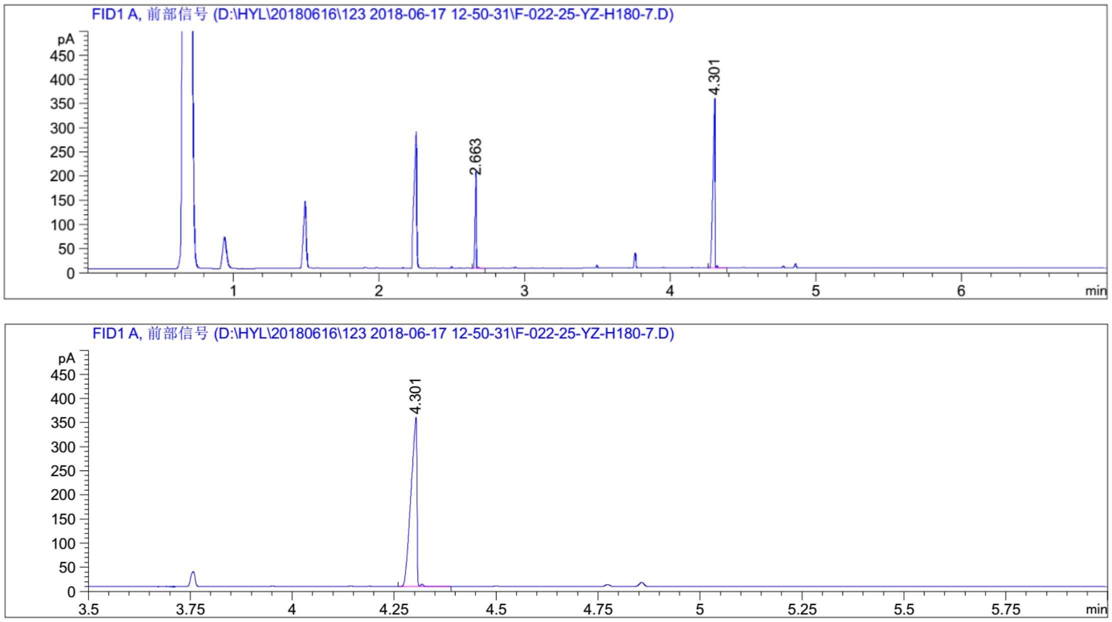

Supplementary Figure 76. C spectra of compound **4c**, Related to Figure 4.

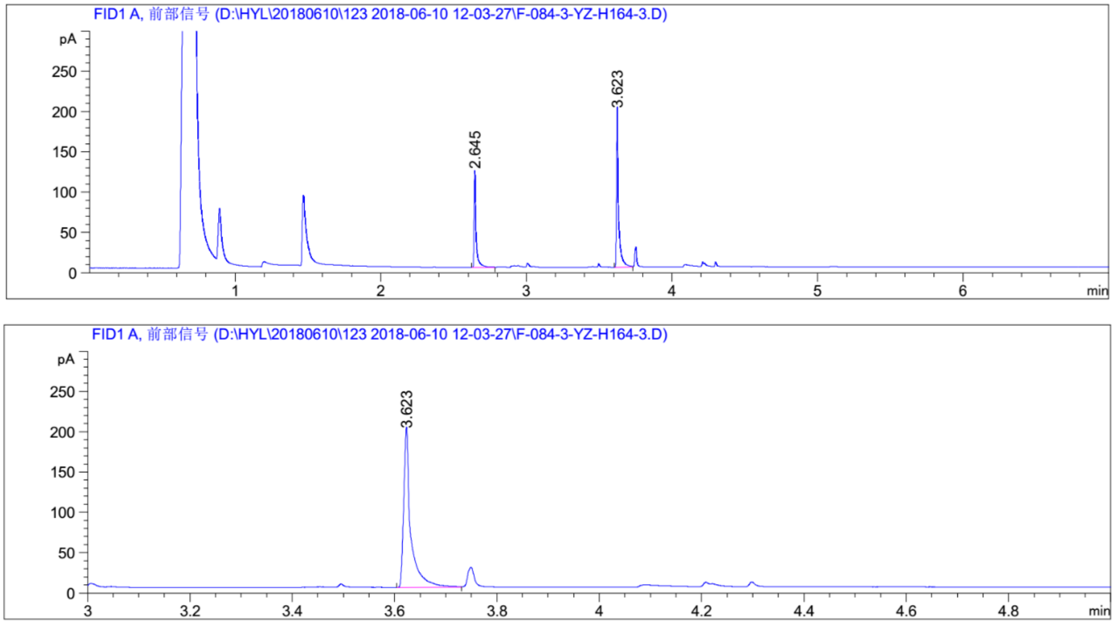

Supplementary Figure 77. GC spectra of compound **4i**, Related to Figure 4.

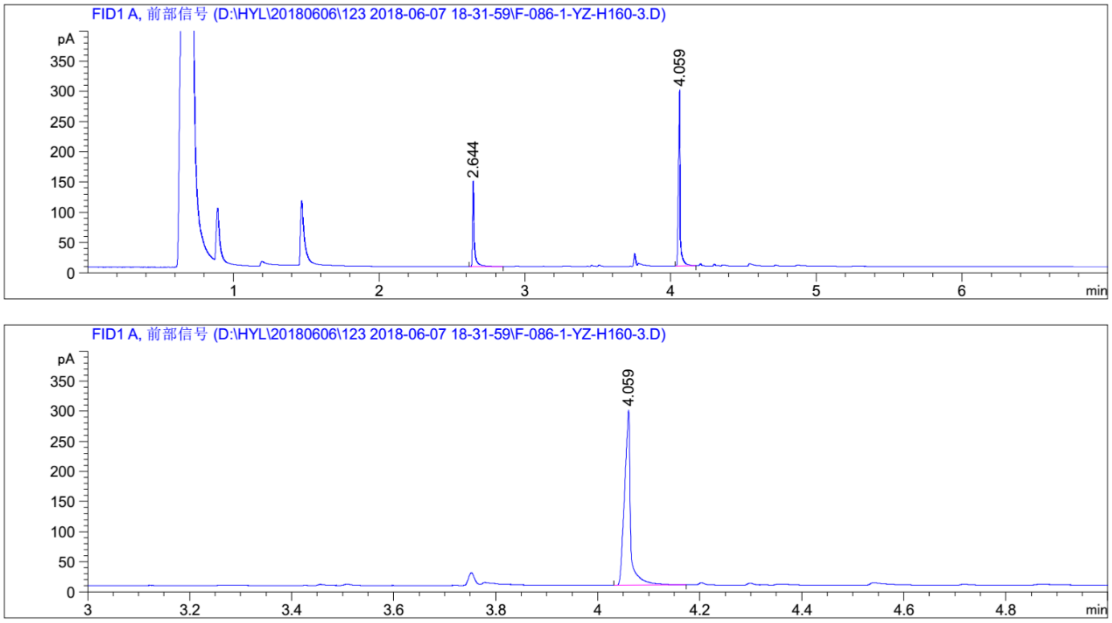

Supplementary Figure 78. GC spectra of compound **4j**, Related to Figure 4.

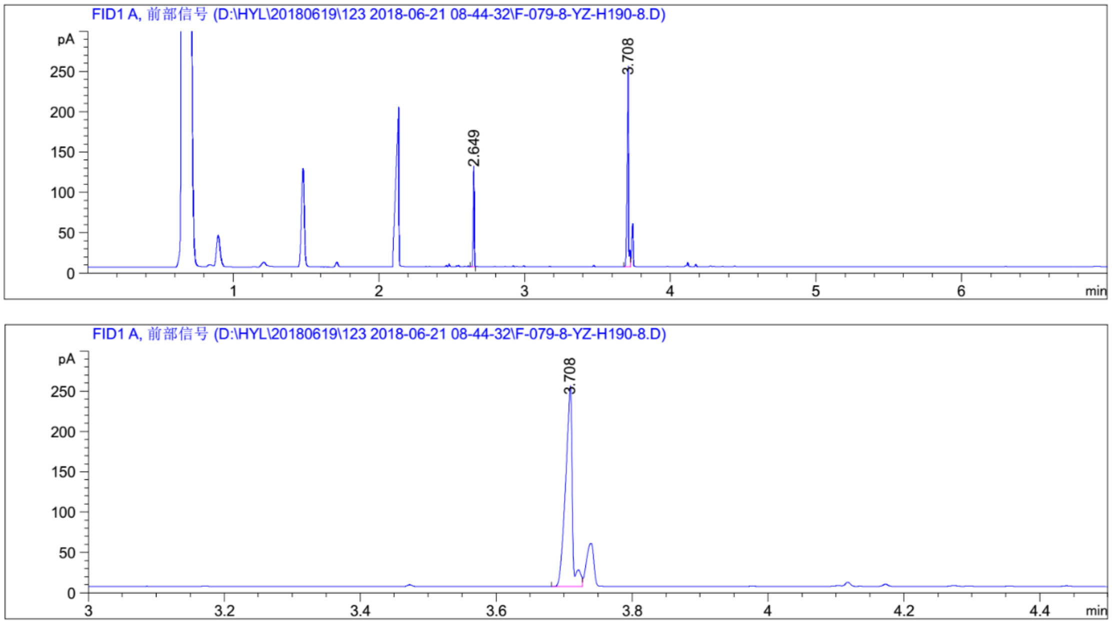

Supplementary Figure 79. GC spectra of compound **4k**, Related to Figure 4.

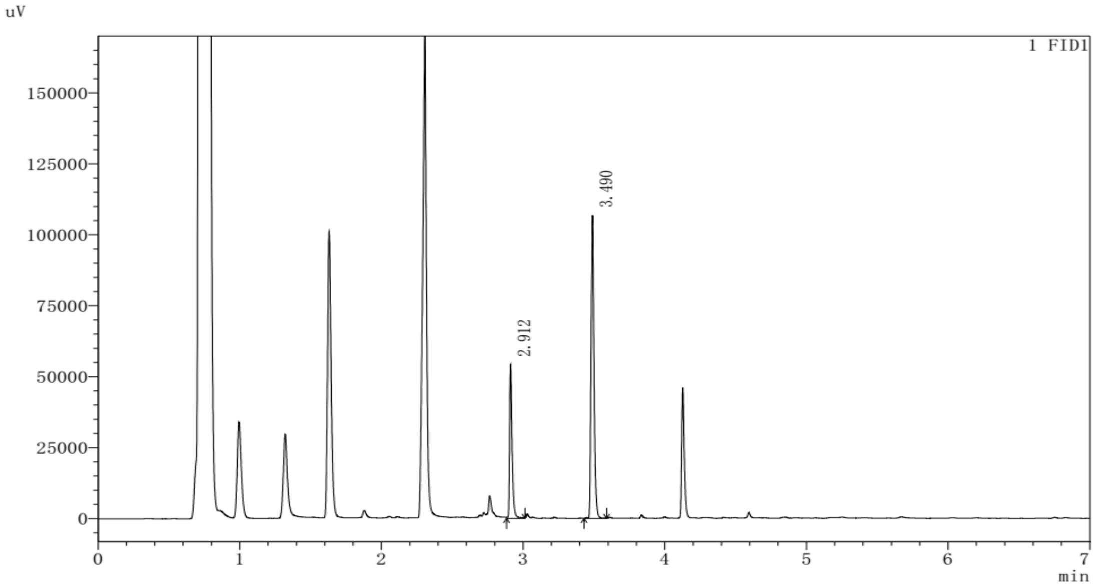

Supplementary Figure 80. GC spectra of compound **4l**, Related to Figure 4.

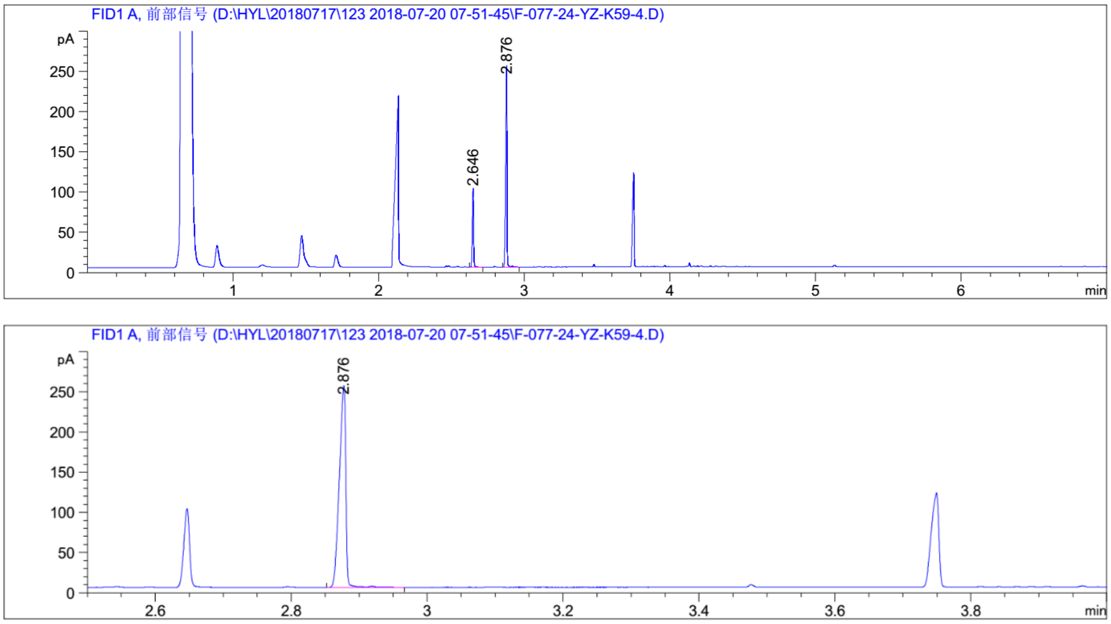

Supplementary Figure 81. GC spectra of compound **4m**, Related to Figure 4.

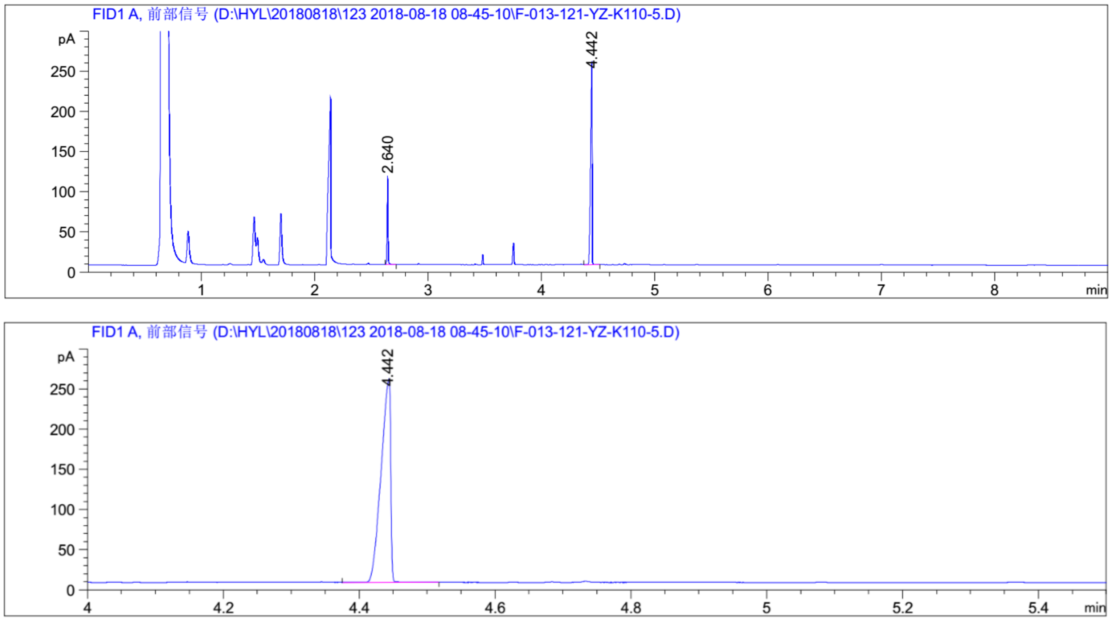

Supplementary Figure 82. GC spectra of compound **4n**, Related to Figure 4.

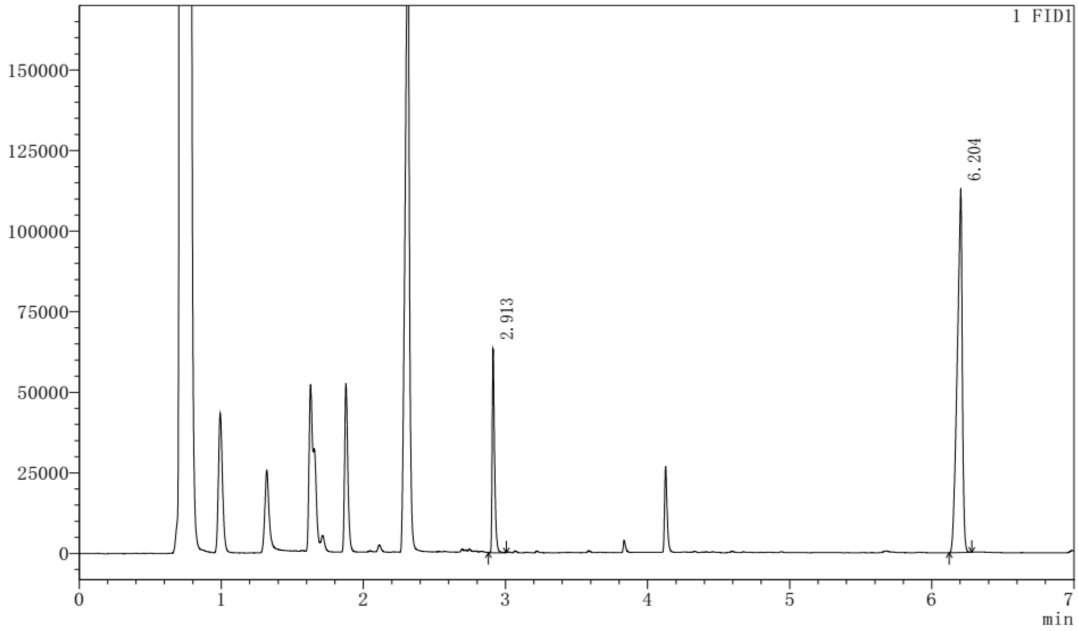

Supplementary Figure 83. GC spectra of compound **4o**, Related to Figure 4.

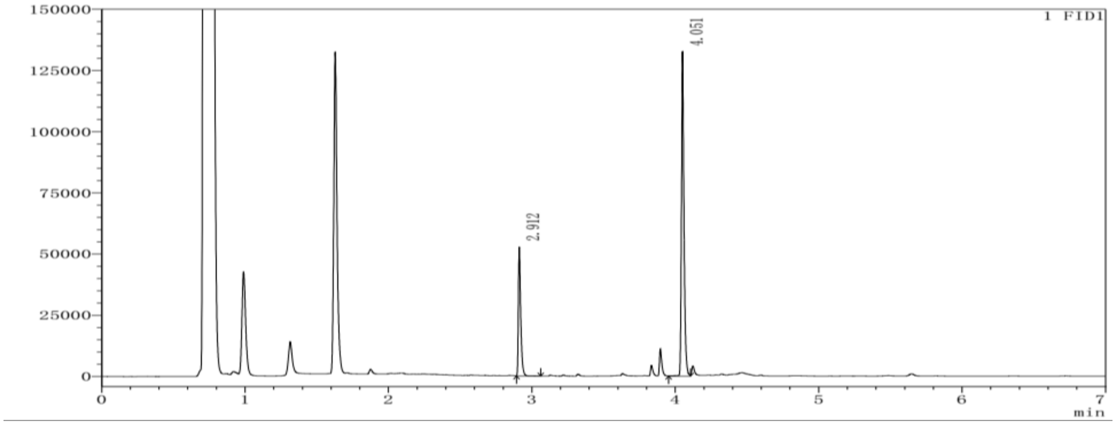

Supplementary Figure 84. GC spectra of compound **4p**, Related to Figure 4.

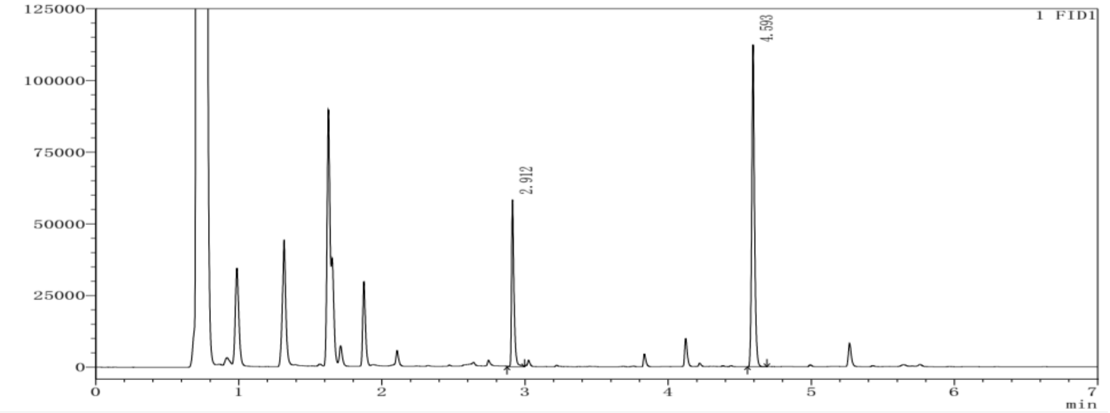

Supplementary Figure 85. GC spectra of compound **4q**, Related to Figure 4.

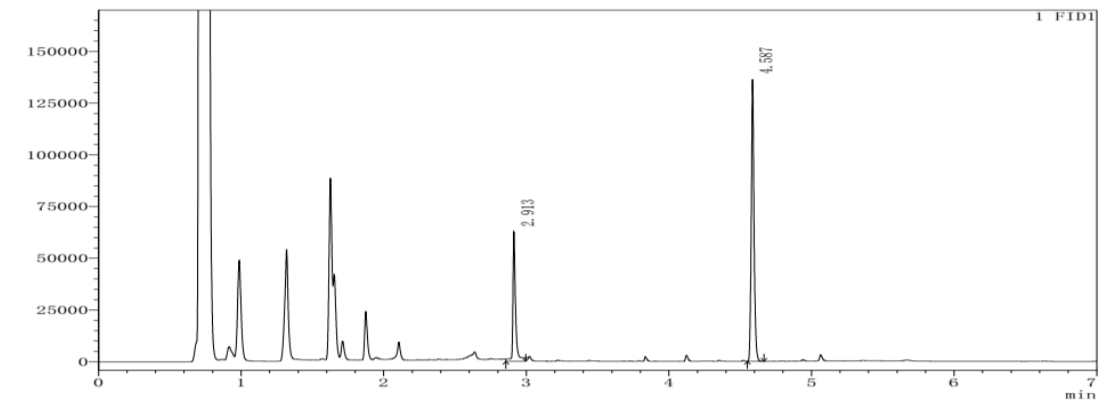

Supplementary Figure 86. GC spectra of compound **4r**, Related to Figure 4.

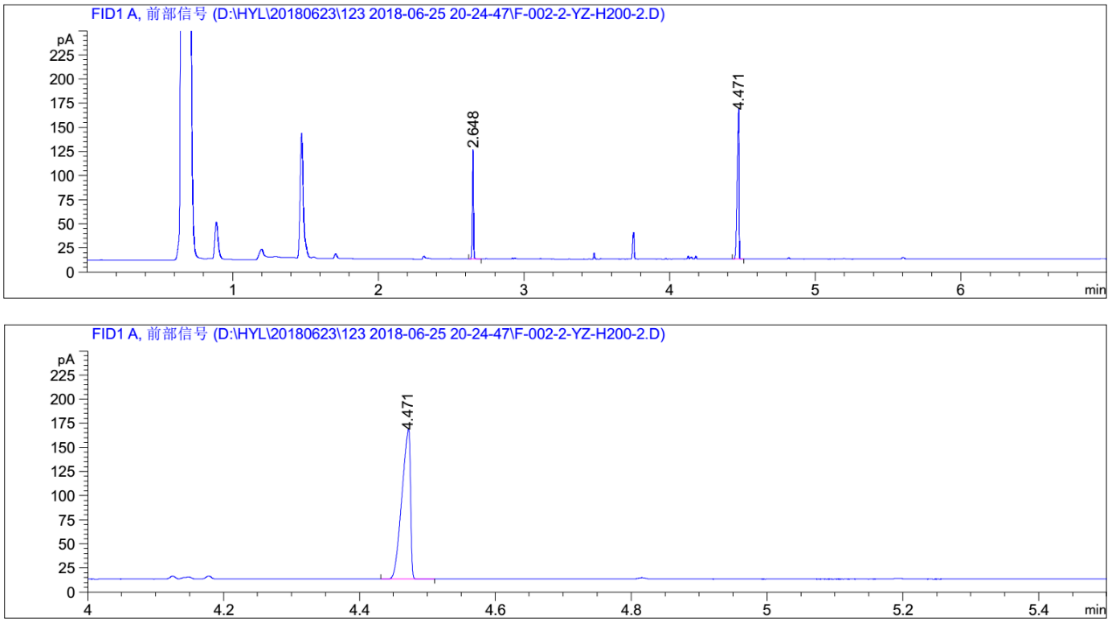

Supplementary Figure 87. GC spectra of compound **4s**, Related to Figure 4.

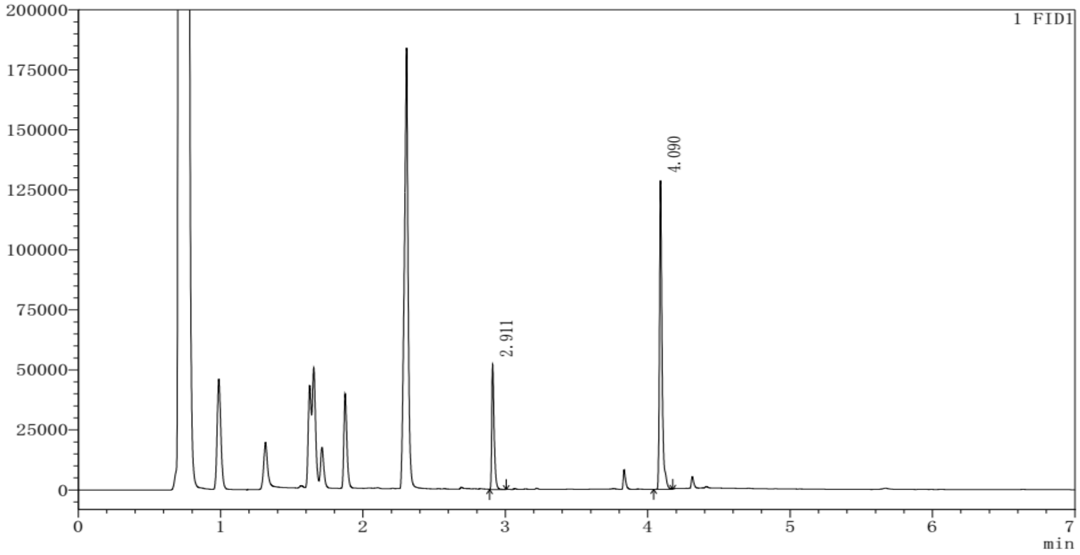

Supplementary Figure 88. GC spectra of compound **4t**, Related to Figure 4.

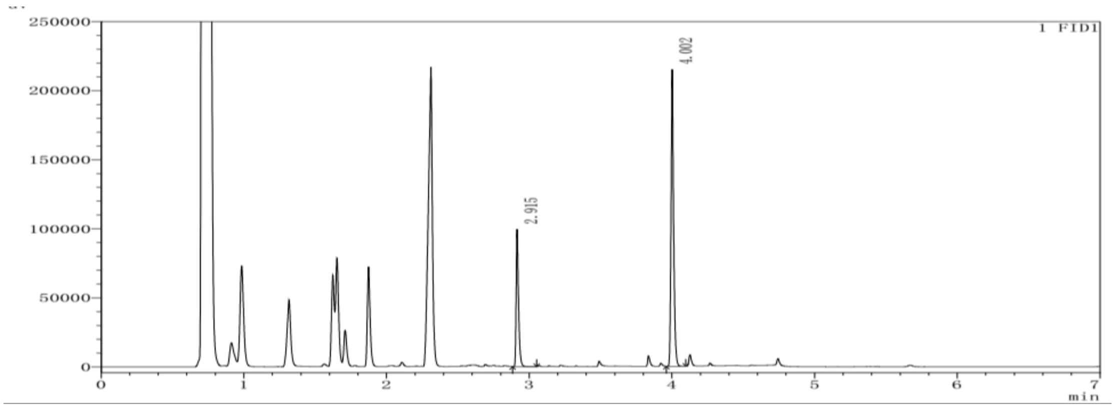

Supplementary Figure 89. GC spectra of compound **4u**, Related to Figure 4.

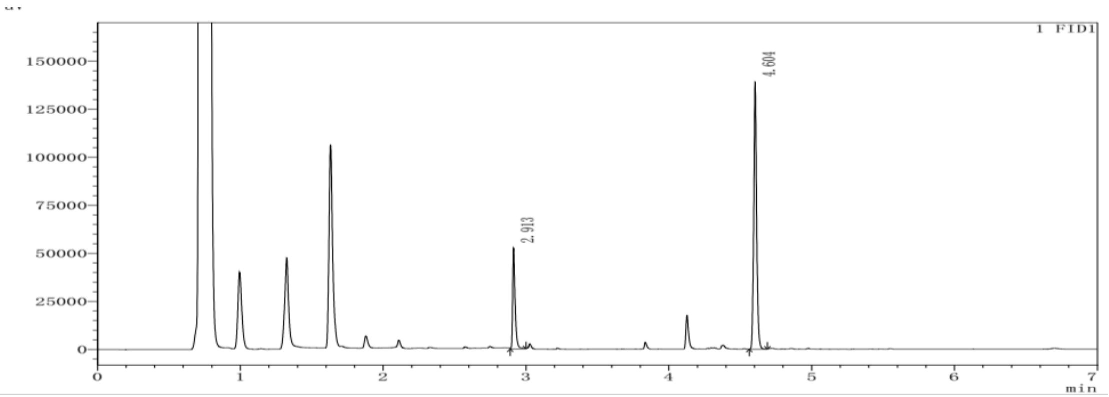

Supplementary Figure 90. GC spectra of compound **4v**, Related to Figure 4.

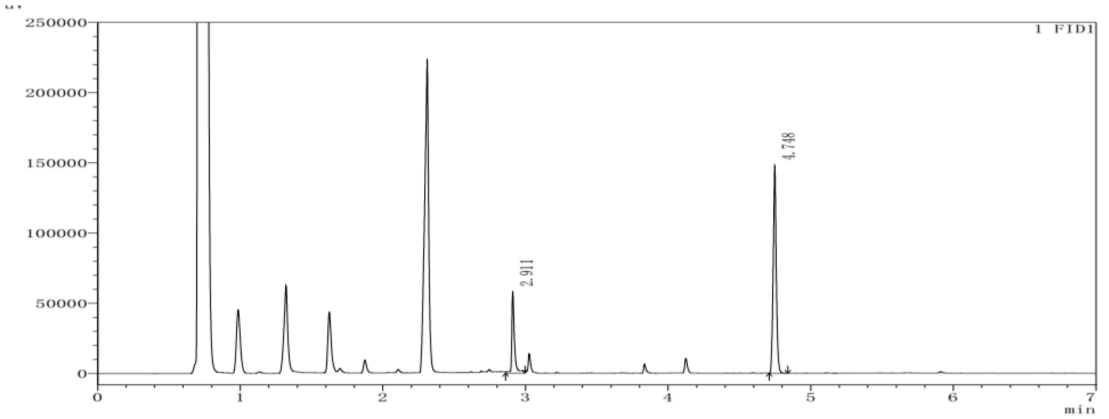

Supplementary Figure 91. GC spectra of compound **4w**, Related to Figure 4.

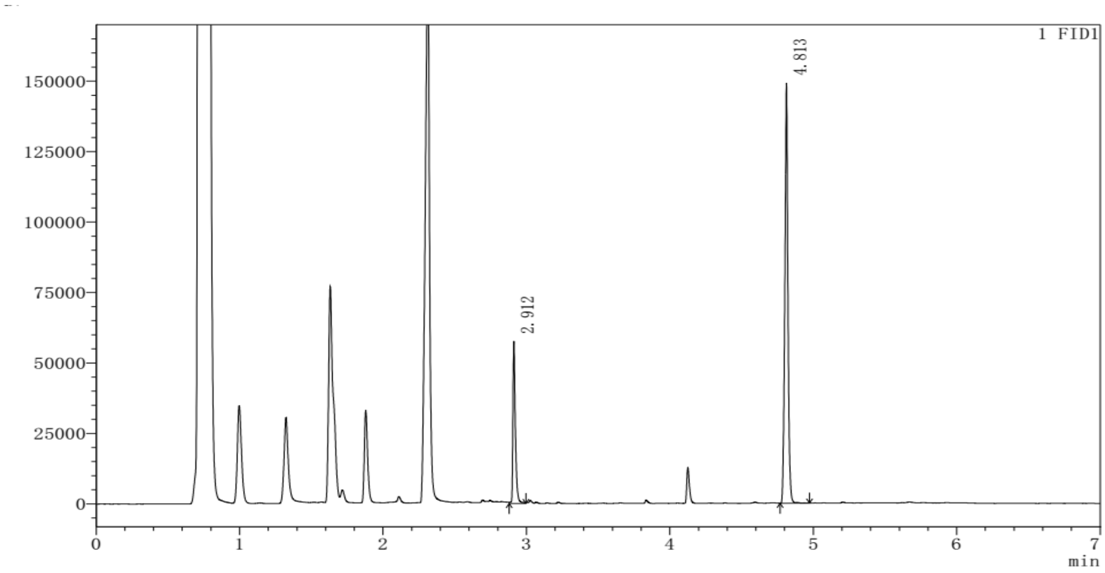

Supplementary Figure 92. GC spectra of compound **4x**, Related to Figure 4.

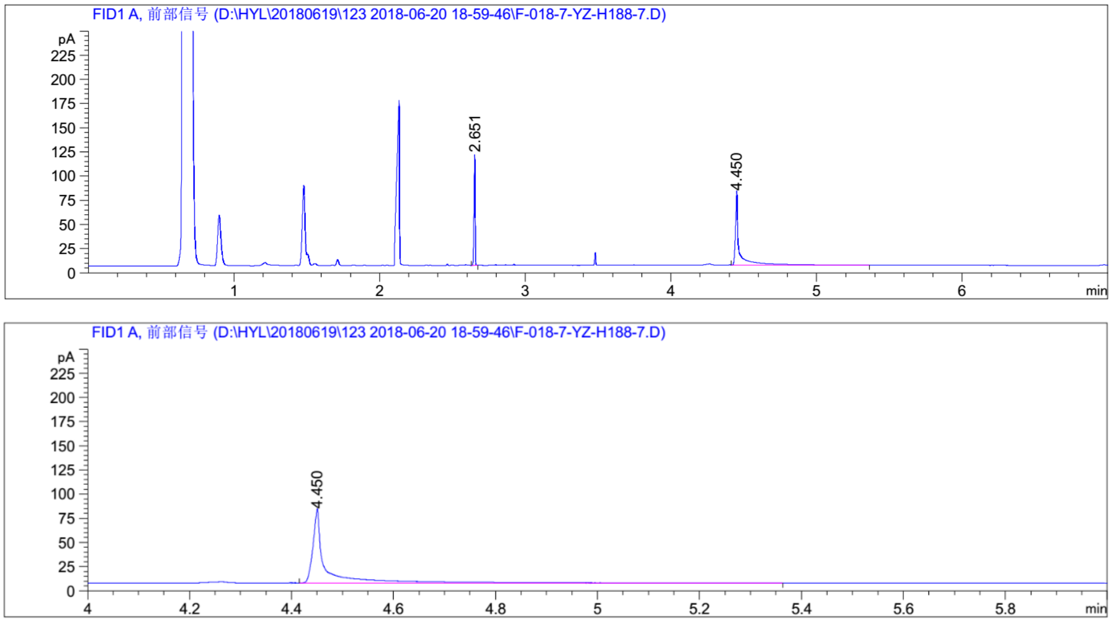

Supplementary Figure 93. GC spectra of compound **4y**, Related to Figure 4.

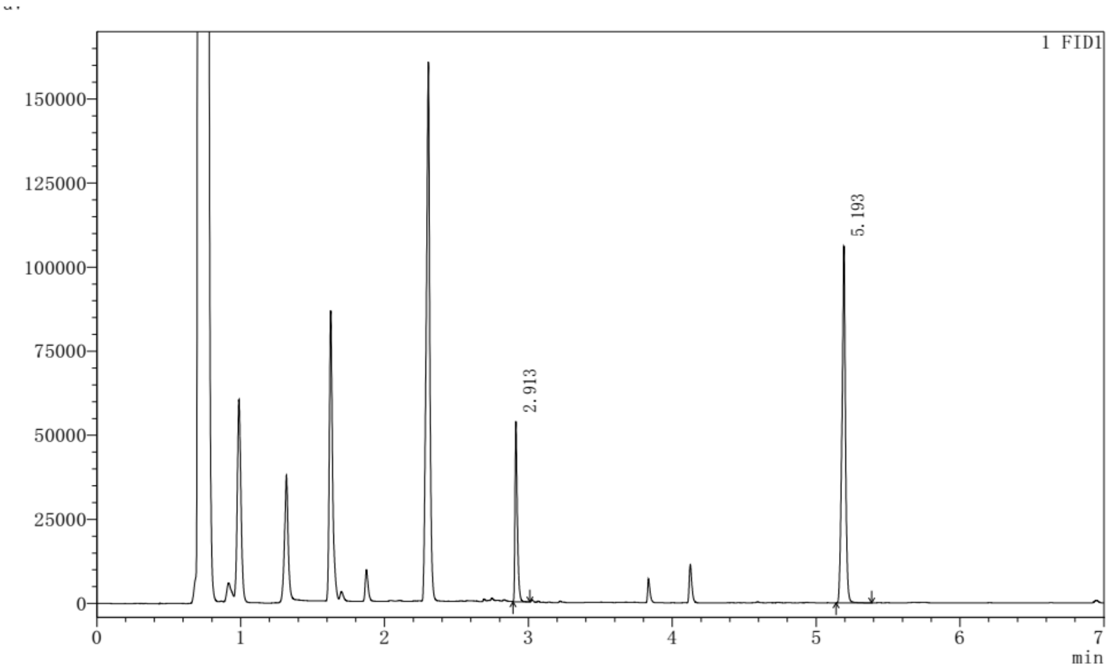

Supplementary Figure 94. GC spectra of compound **4z**, Related to Figure 4.

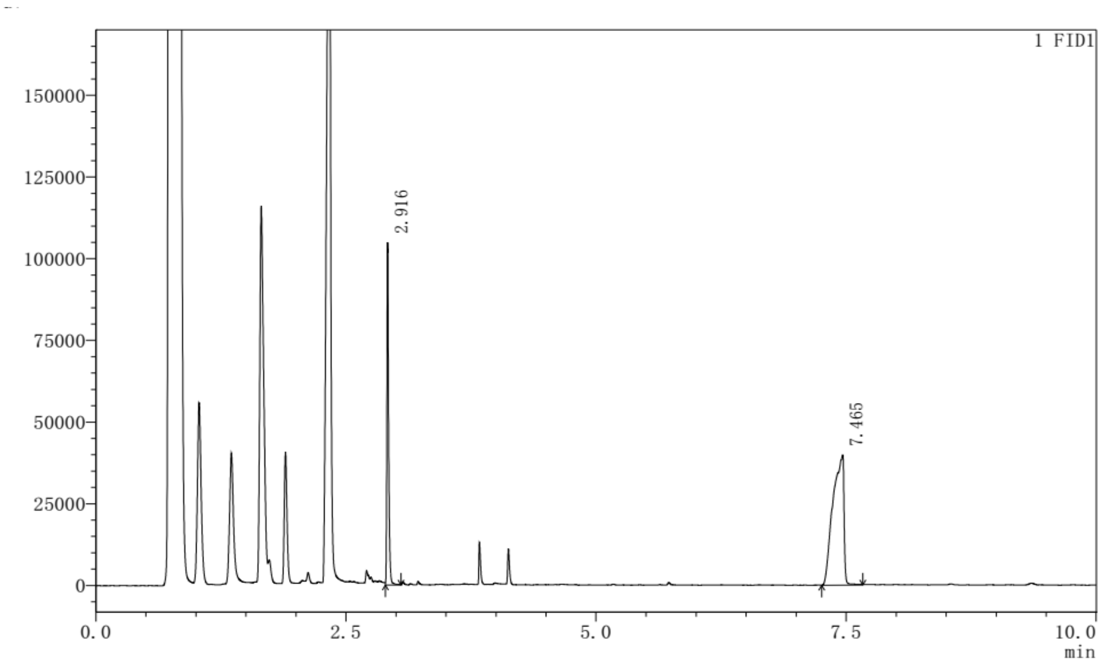

Supplementary Figure 95. GC spectra of compound **4a'**, Related to Figure 4.

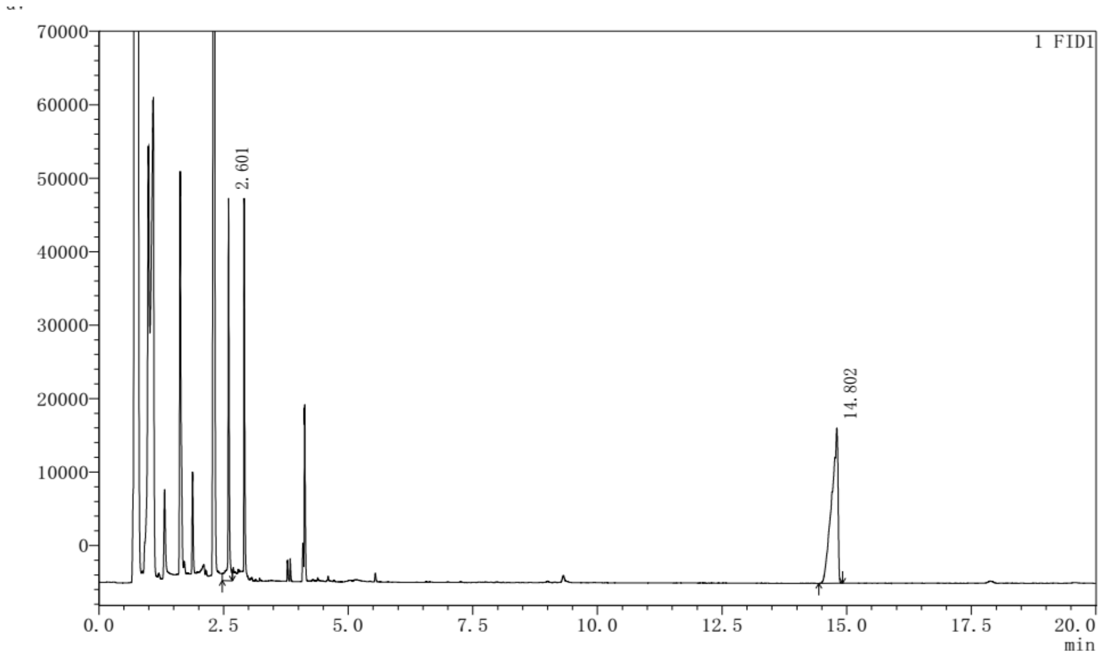

Supplementary Figure 96. GC spectra of compound **4b'**, Related to Figure 4.

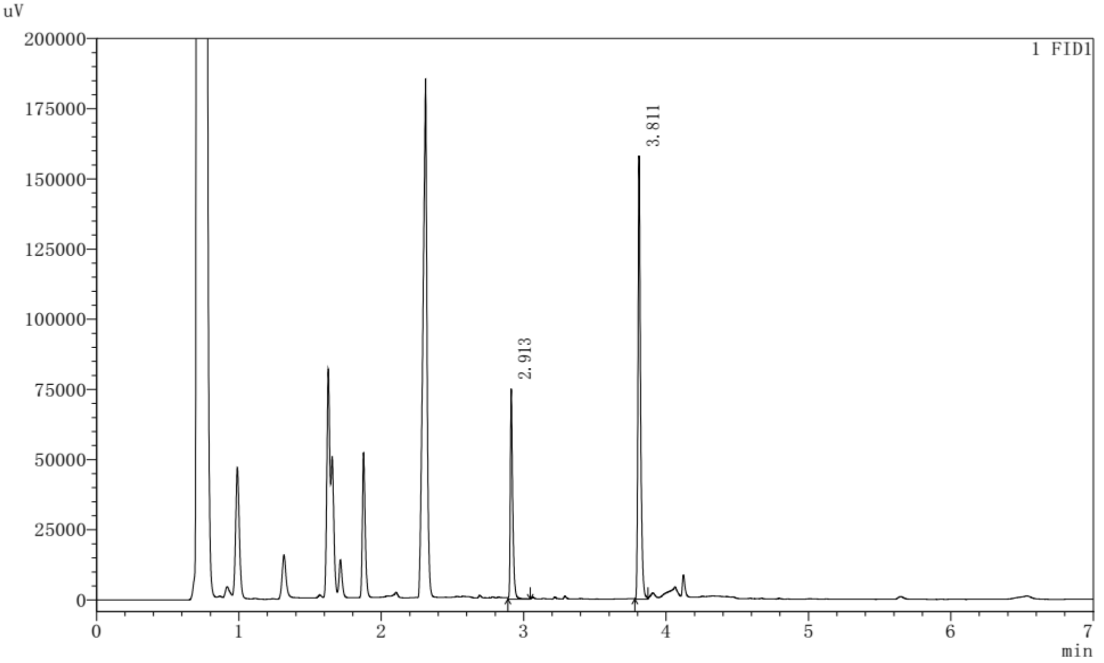

Supplementary Figure 97. GC spectra of compound **4c'**, Related to Figure 4.

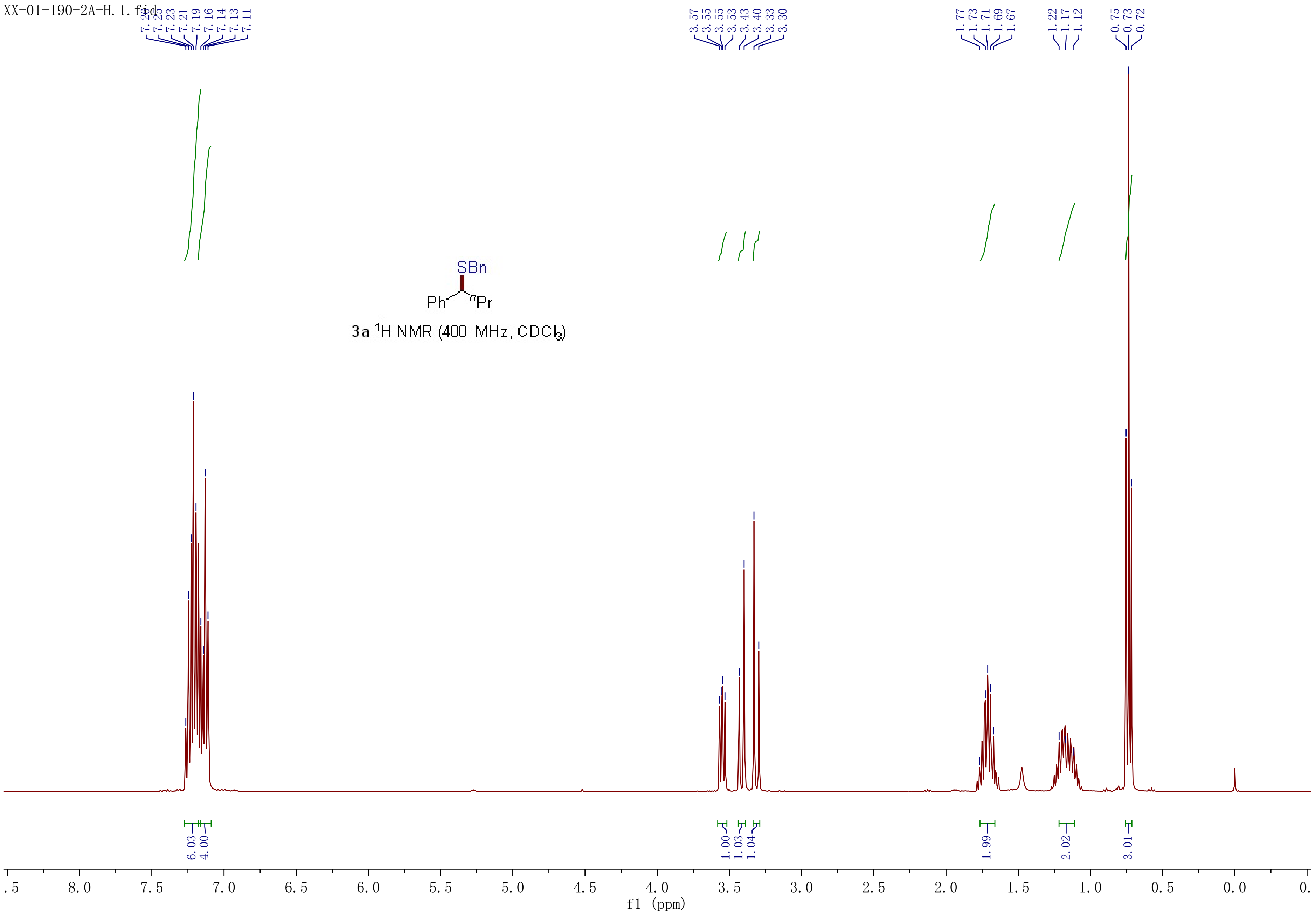

Supplementary Figure 98. ¹H NMR (400 MHz, $CDCl_3$) spectra for compound **3a**

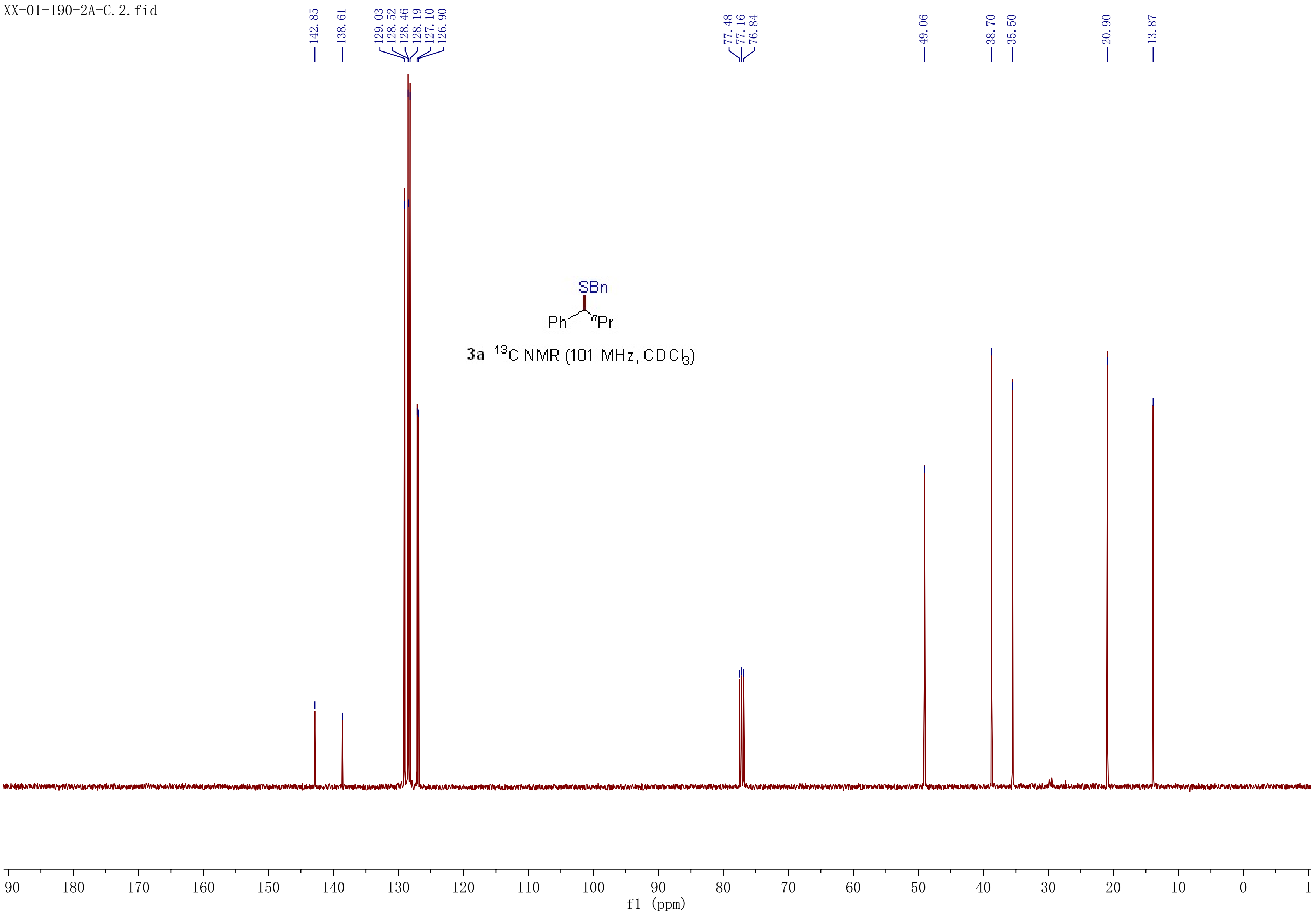

Supplementary Figure 99. ^{13}C NMR (101 MHz, $CDCl_3$) spectra for compound **3a**

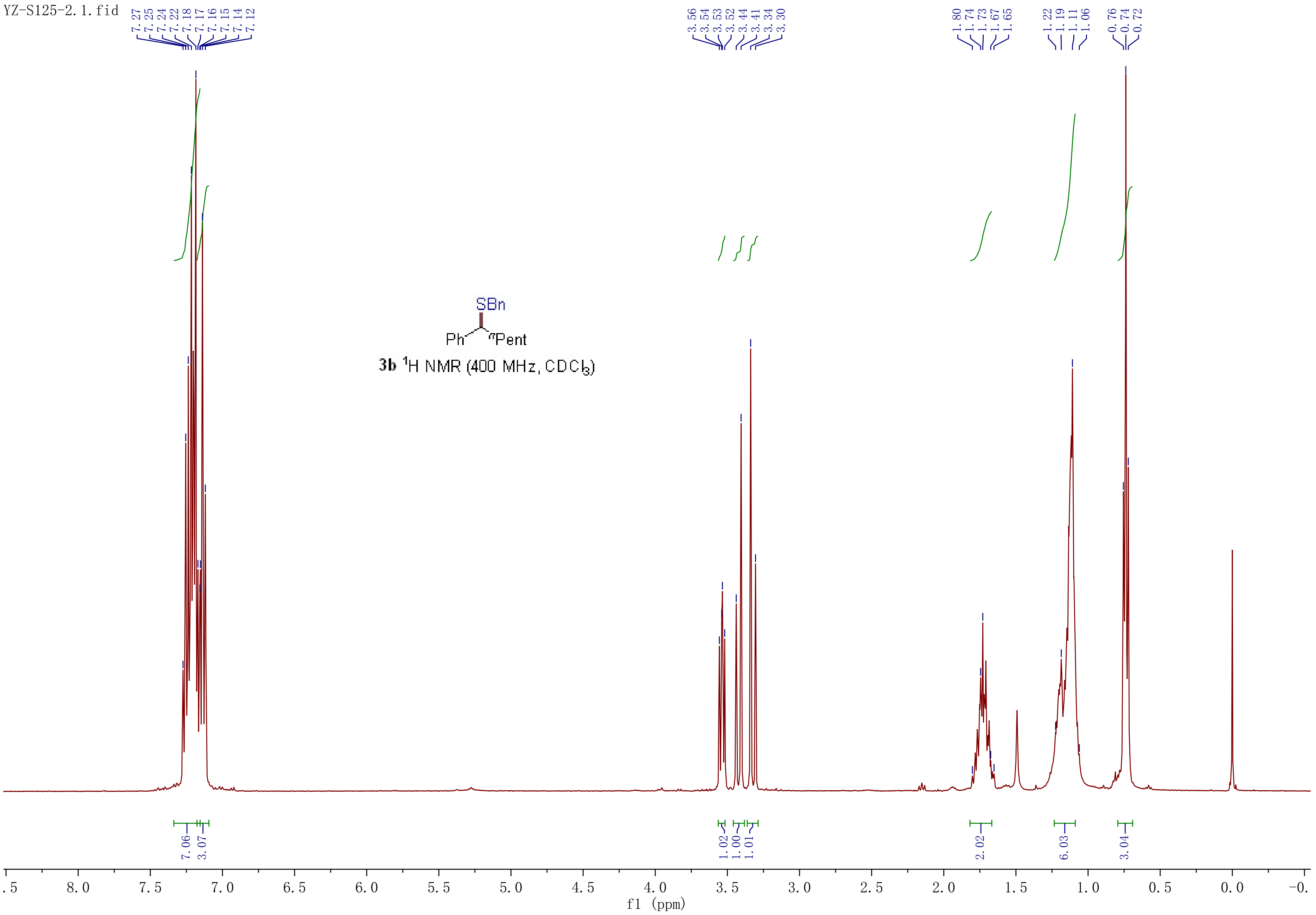

Supplementary Figure 100. ^{1}H NMR (400 MHz, $CDCl_3$) spectra for compound **3b**

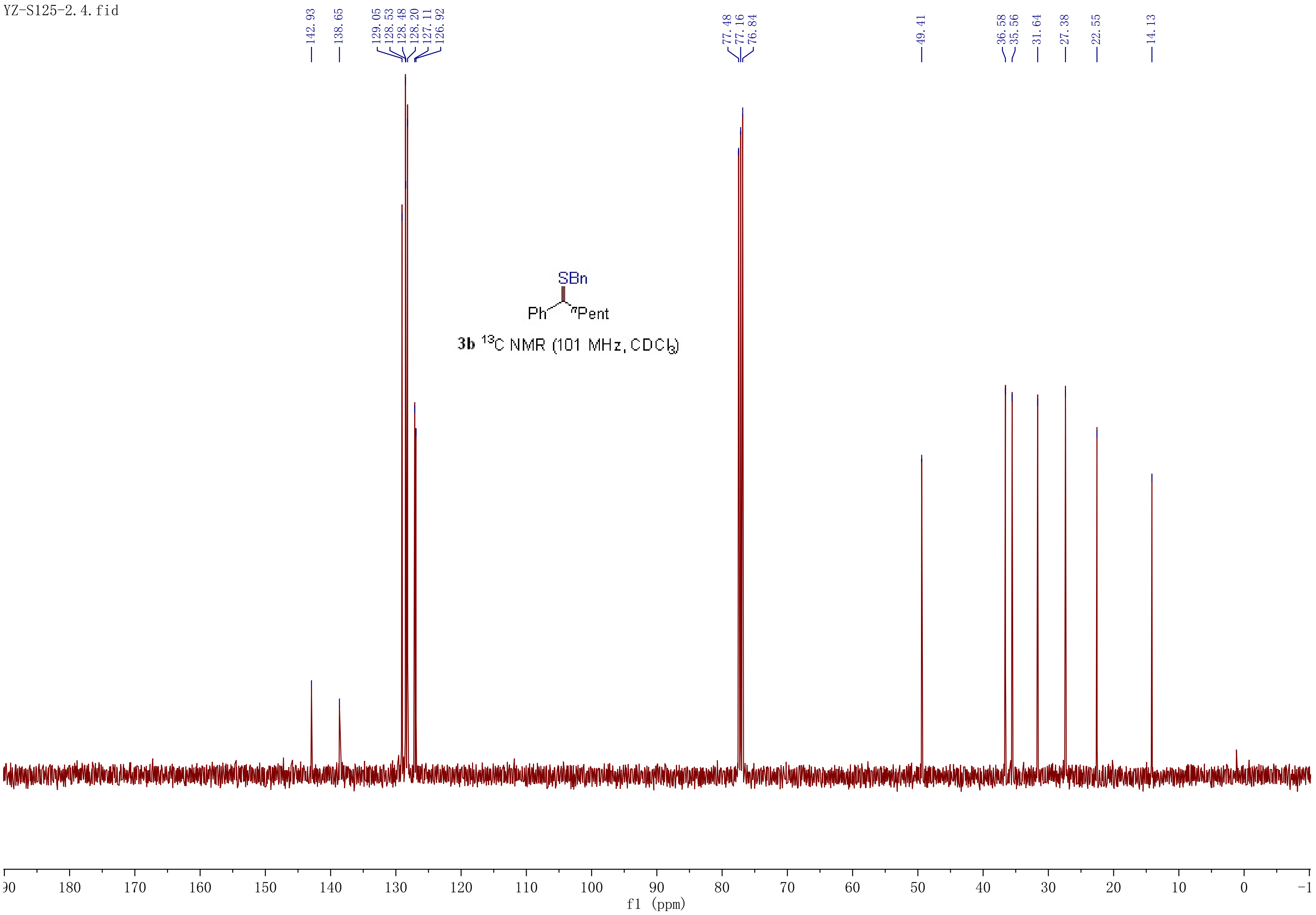

Supplementary Figure 101. ^{13}C NMR (101 MHz, $CDCl_3$) spectra for compound **3b**

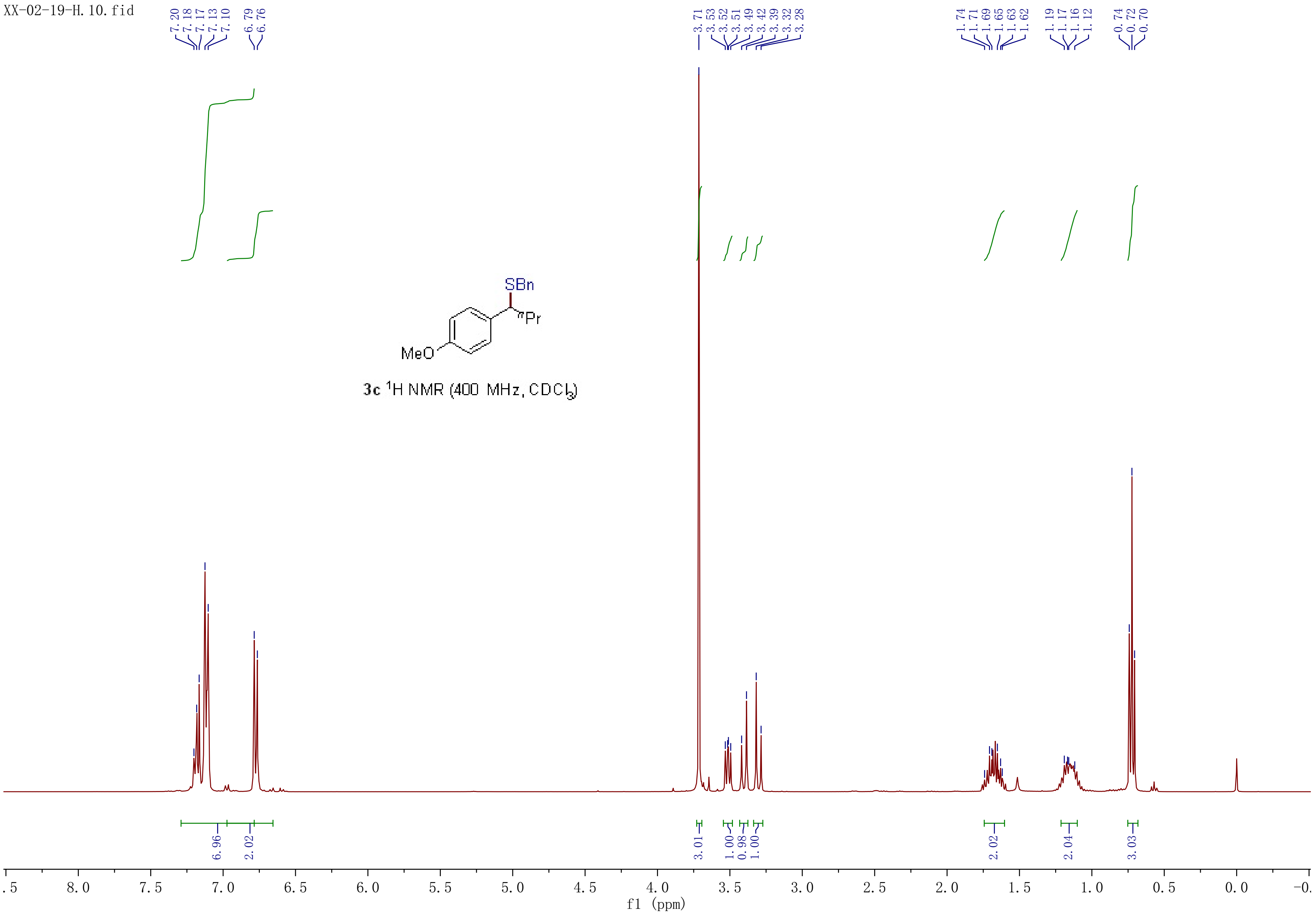

Supplementary Figure 102. ^{1}H NMR (400 MHz, $CDCl_3$) spectra for compound **3c**

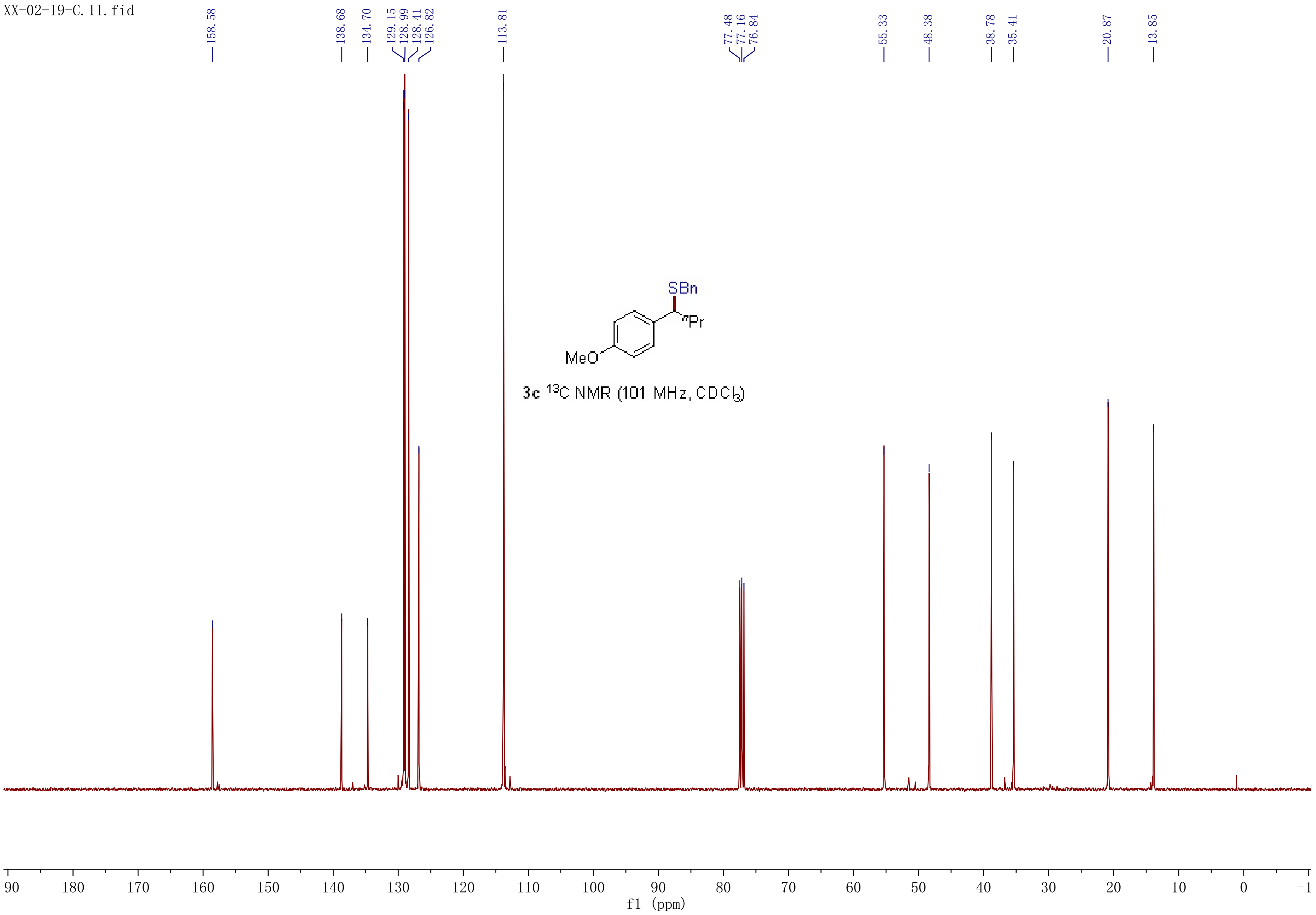

Supplementary Figure 103. ^{13}C NMR (101 MHz, $CDCl_3$) spectra for compound **3c**

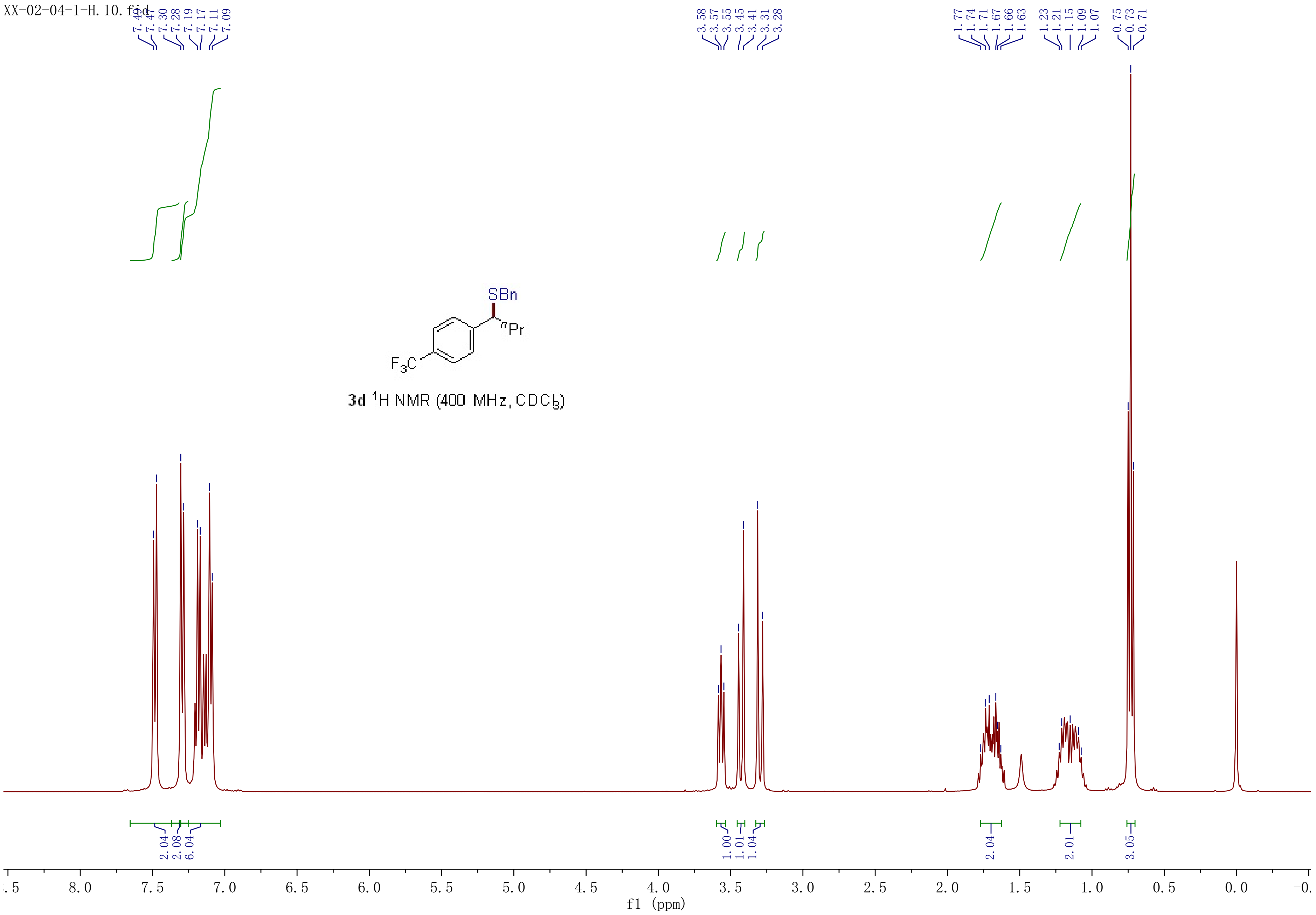

Supplementary Figure 104. ^{1}H NMR (400 MHz, $CDCl_3$) spectra for compound **3d**

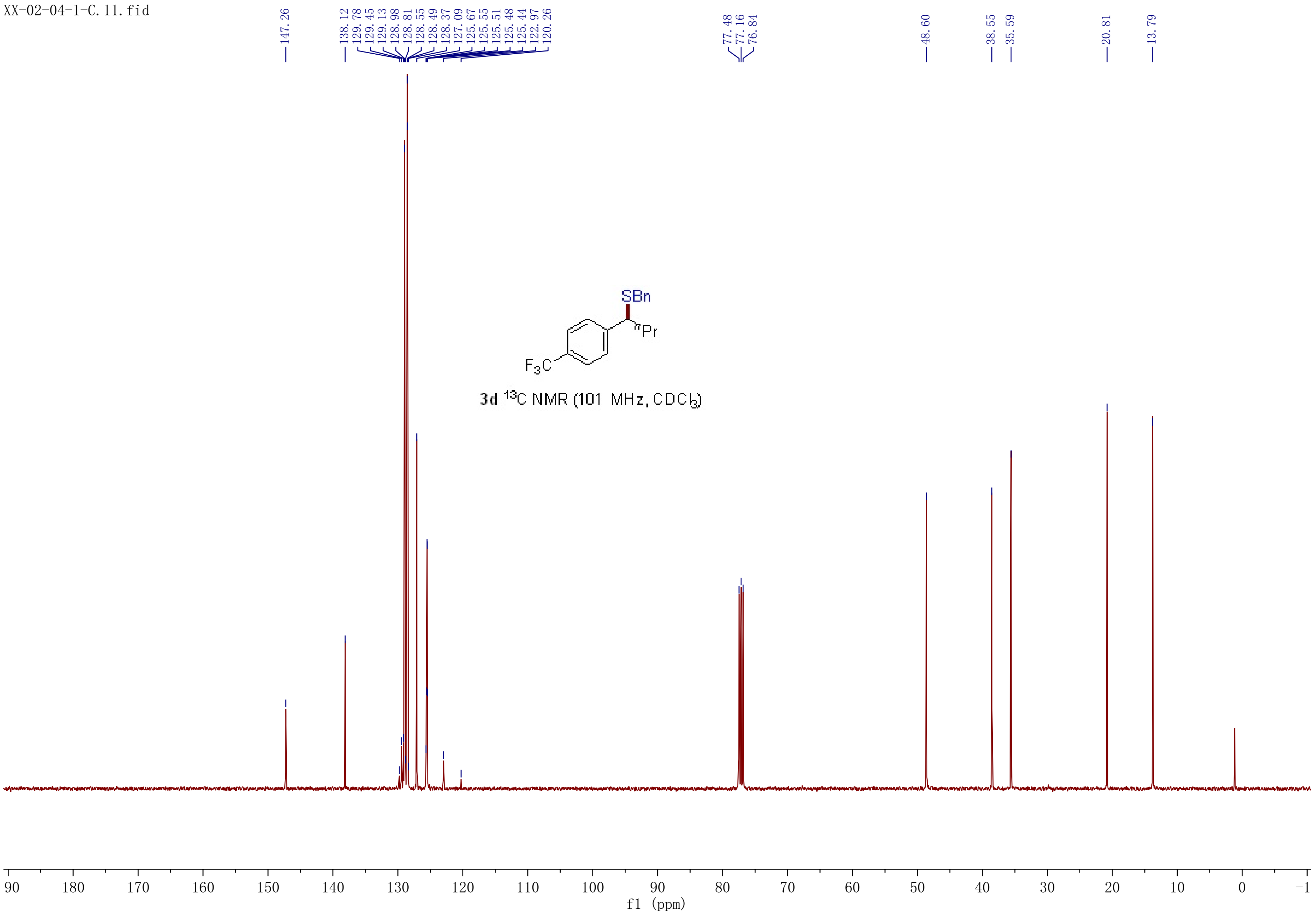

Supplementary Figure 105. ^{13}C NMR (101 MHz, $CDCl_3$) spectra for compound **3d**

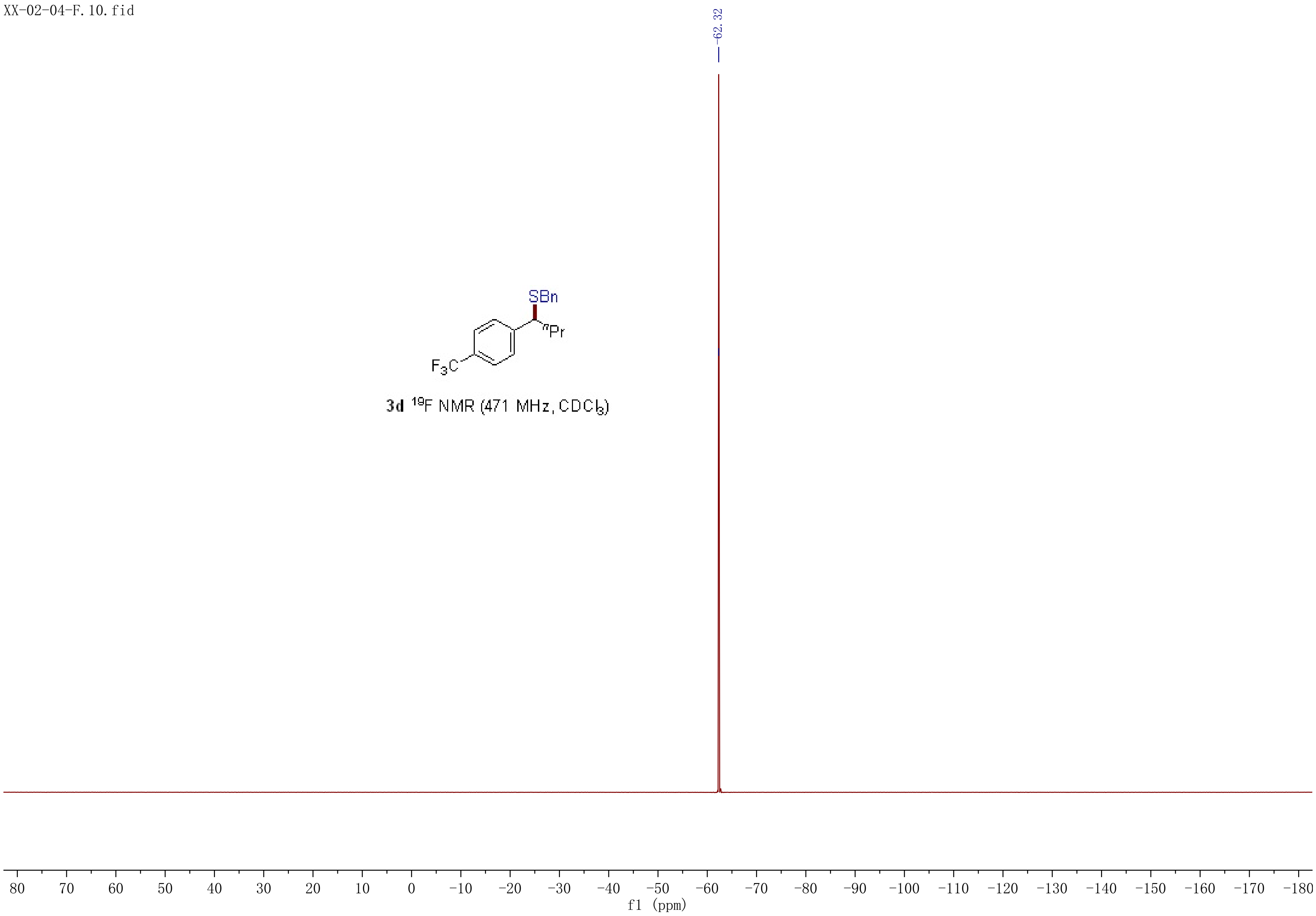

Supplementary Figure 106. ^{19}F NMR (471 MHz, $CDCl_3$) spectra for compound **3d**

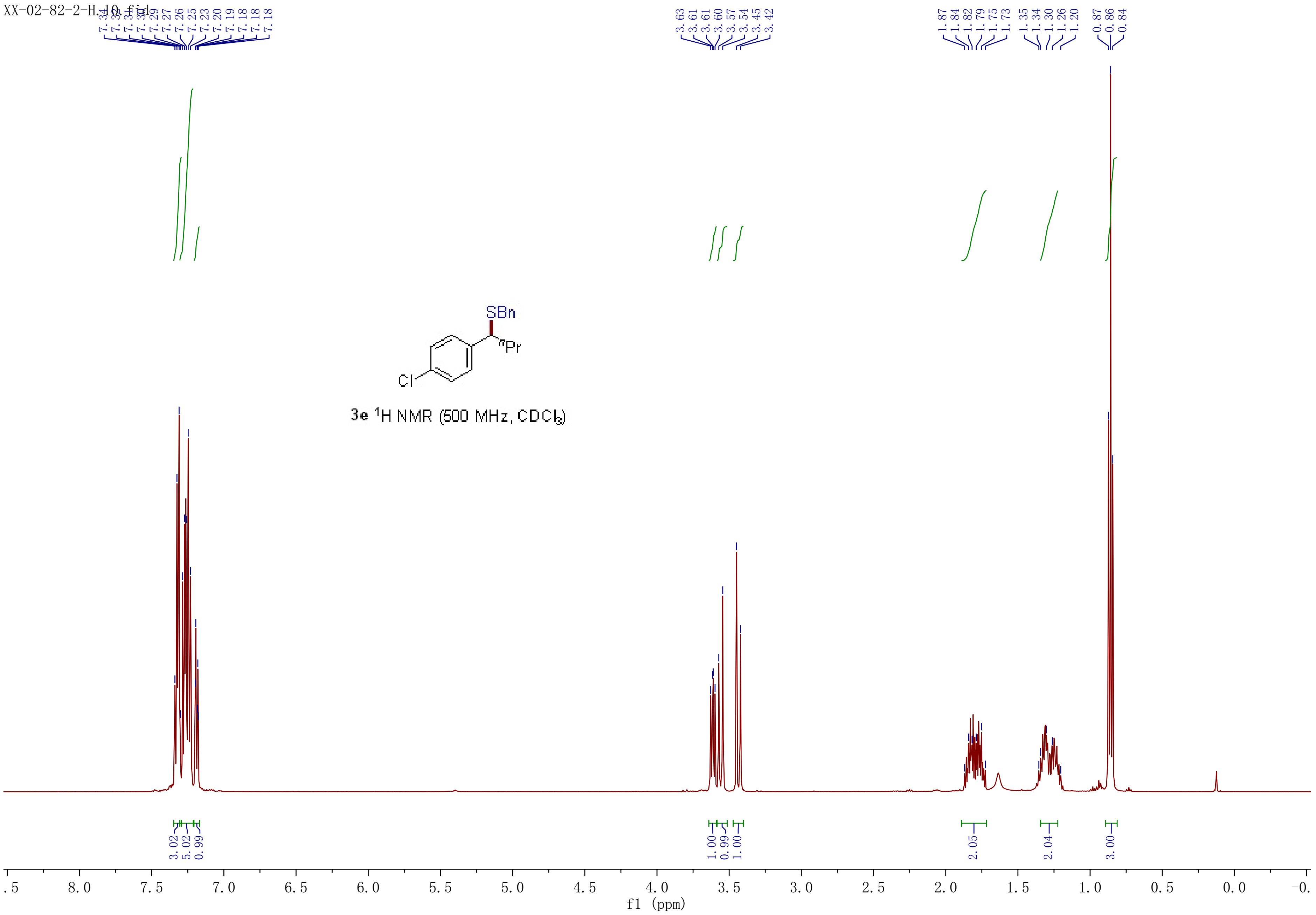

Supplementary Figure 107. ^{1}H NMR (500 MHz, $CDCl_3$) spectra for compound **3e**

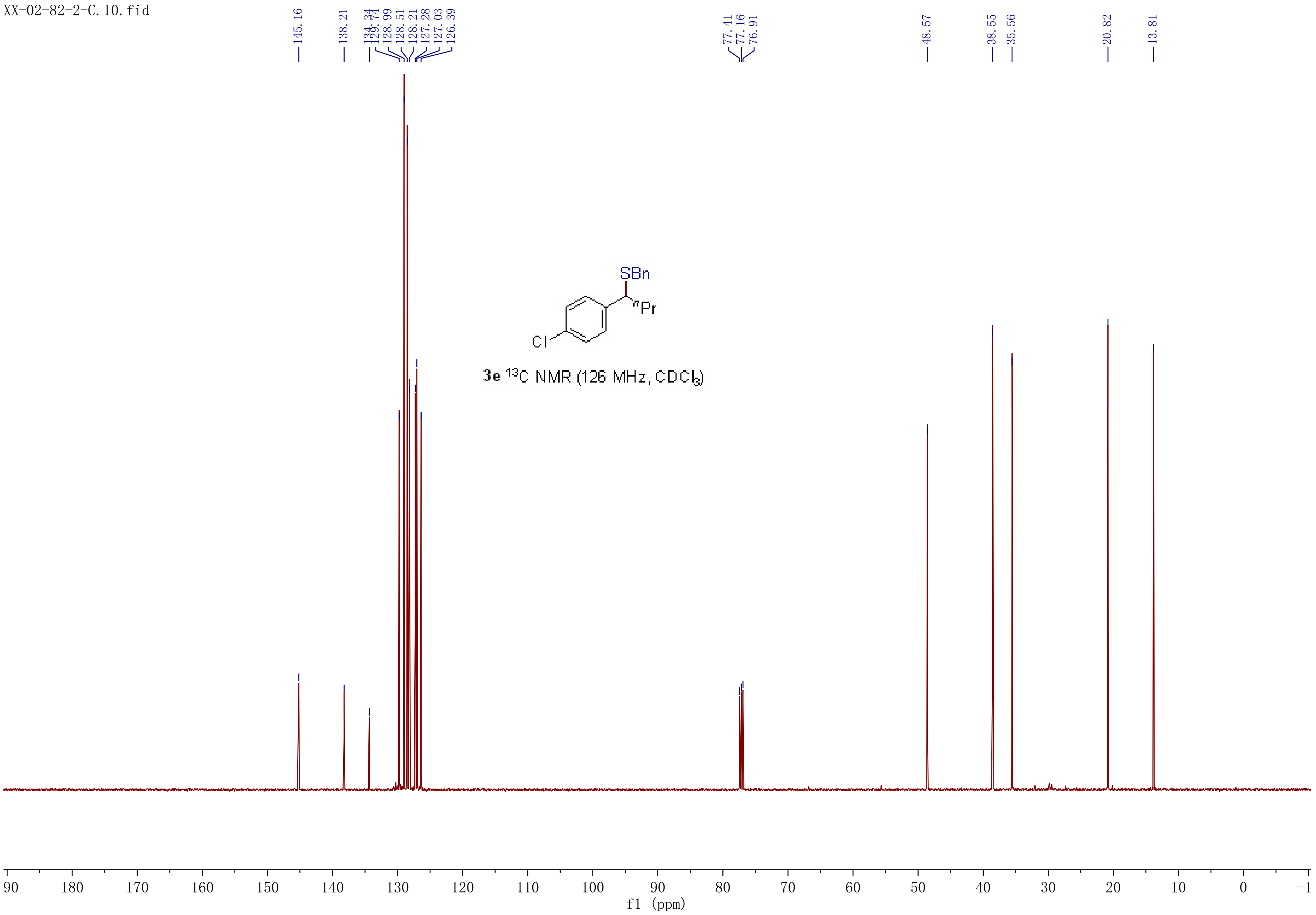

Supplementary Figure 108. ^{13}C NMR (126 MHz, $CDCl_3$) spectra for compound **3e**

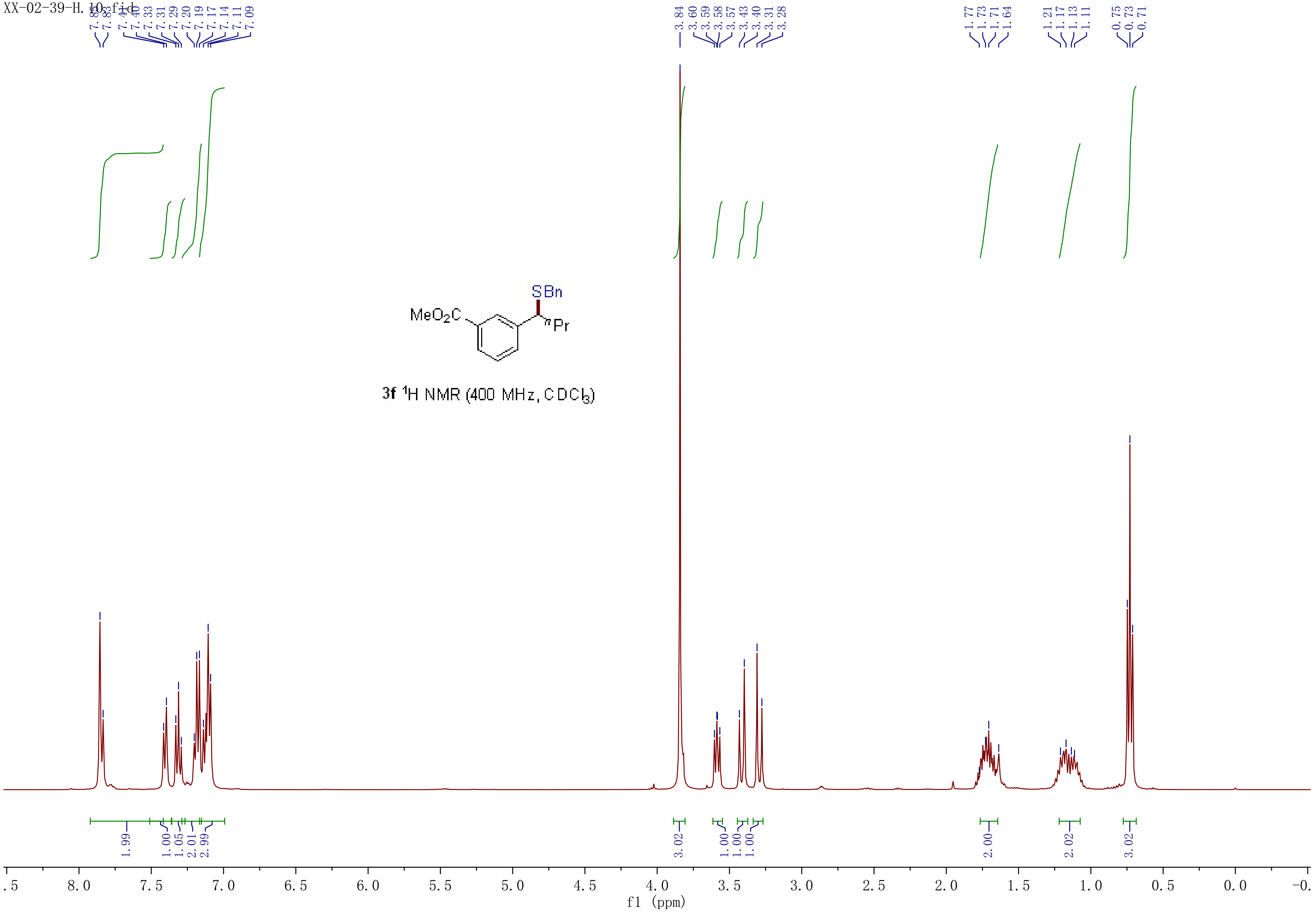

Supplementary Figure 109. ^{1}H NMR (400 MHz, $CDCl_3$) spectra for compound **3f**

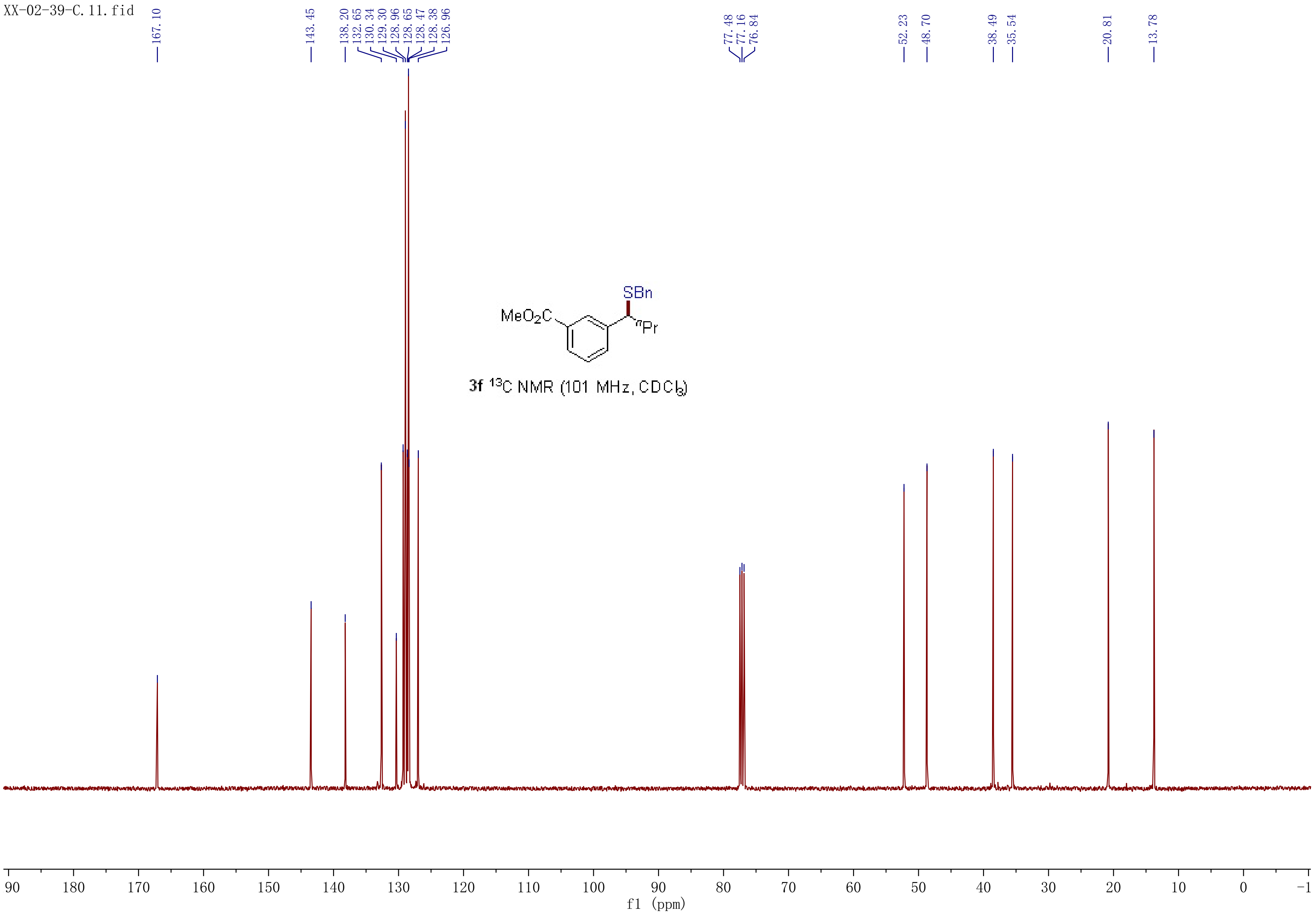

Supplementary Figure 110. ^{13}C NMR (101 MHz, $CDCl_3$) spectra for compound **3f**

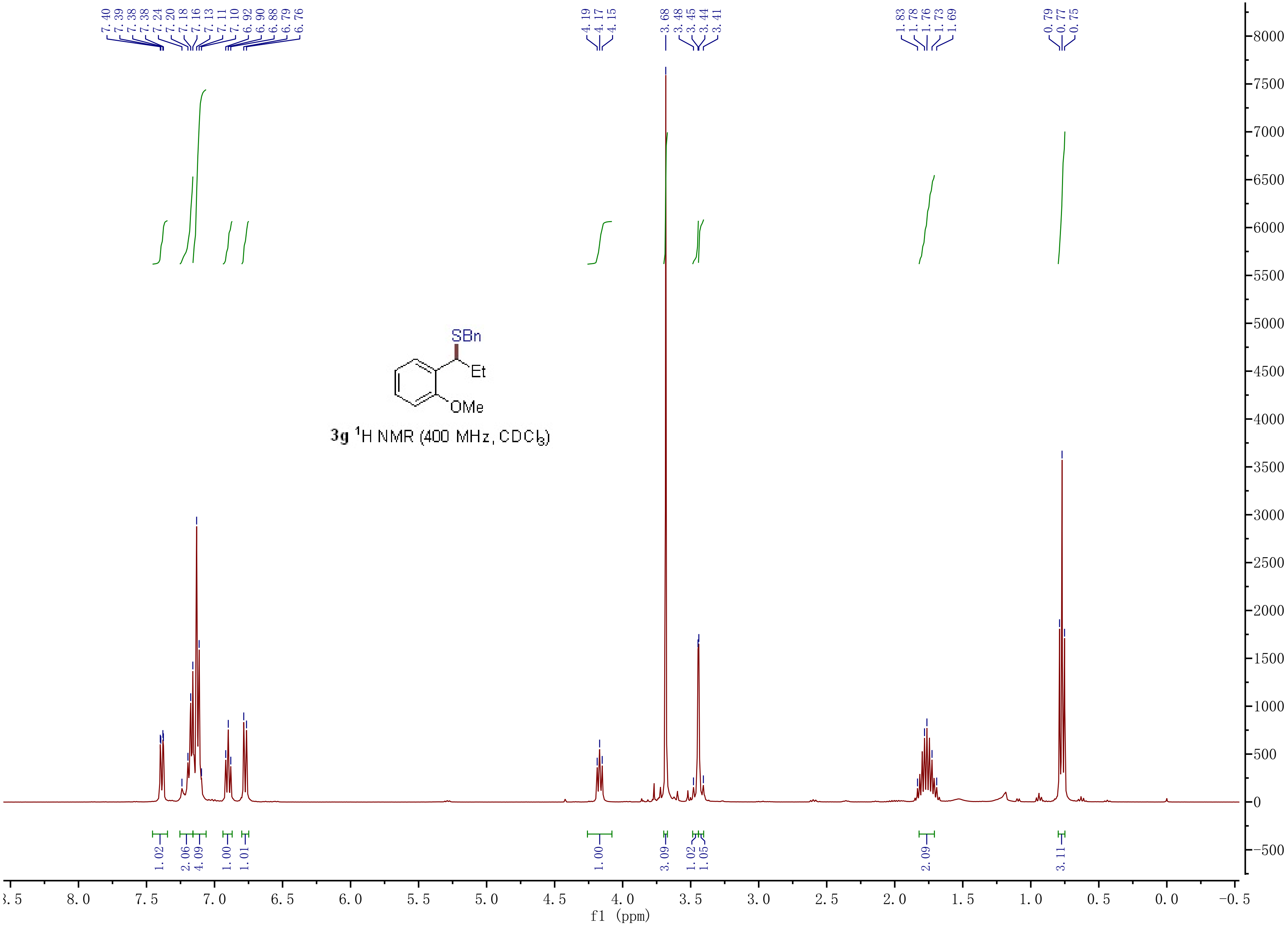

Supplementary Figure 111. ^{1}H NMR (400 MHz, $CDCl_3$) spectra for compound **3g**

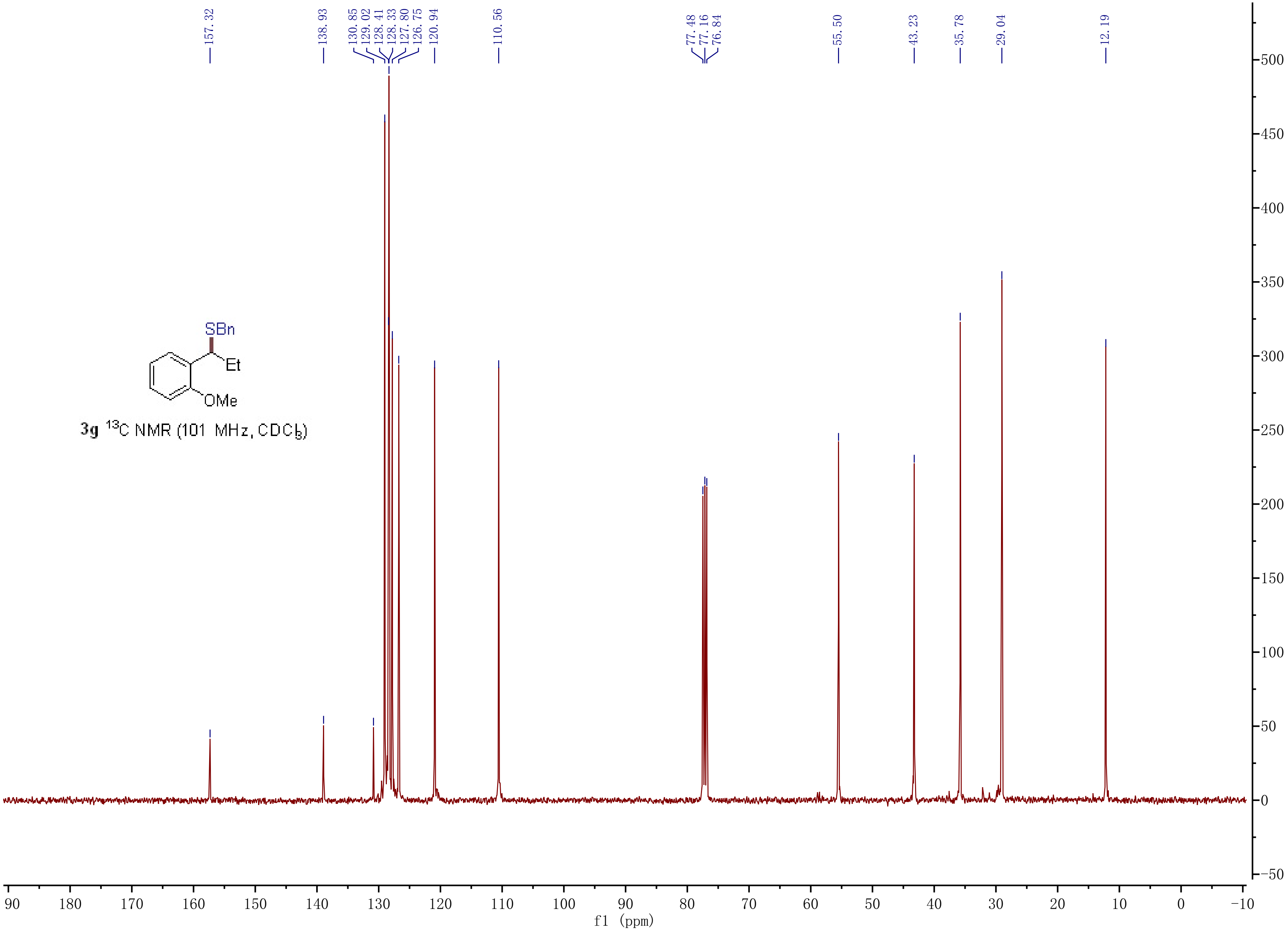

Supplementary Figure 112. ^{13}C NMR (101 MHz, $CDCl_3$) spectra for compound **3g**

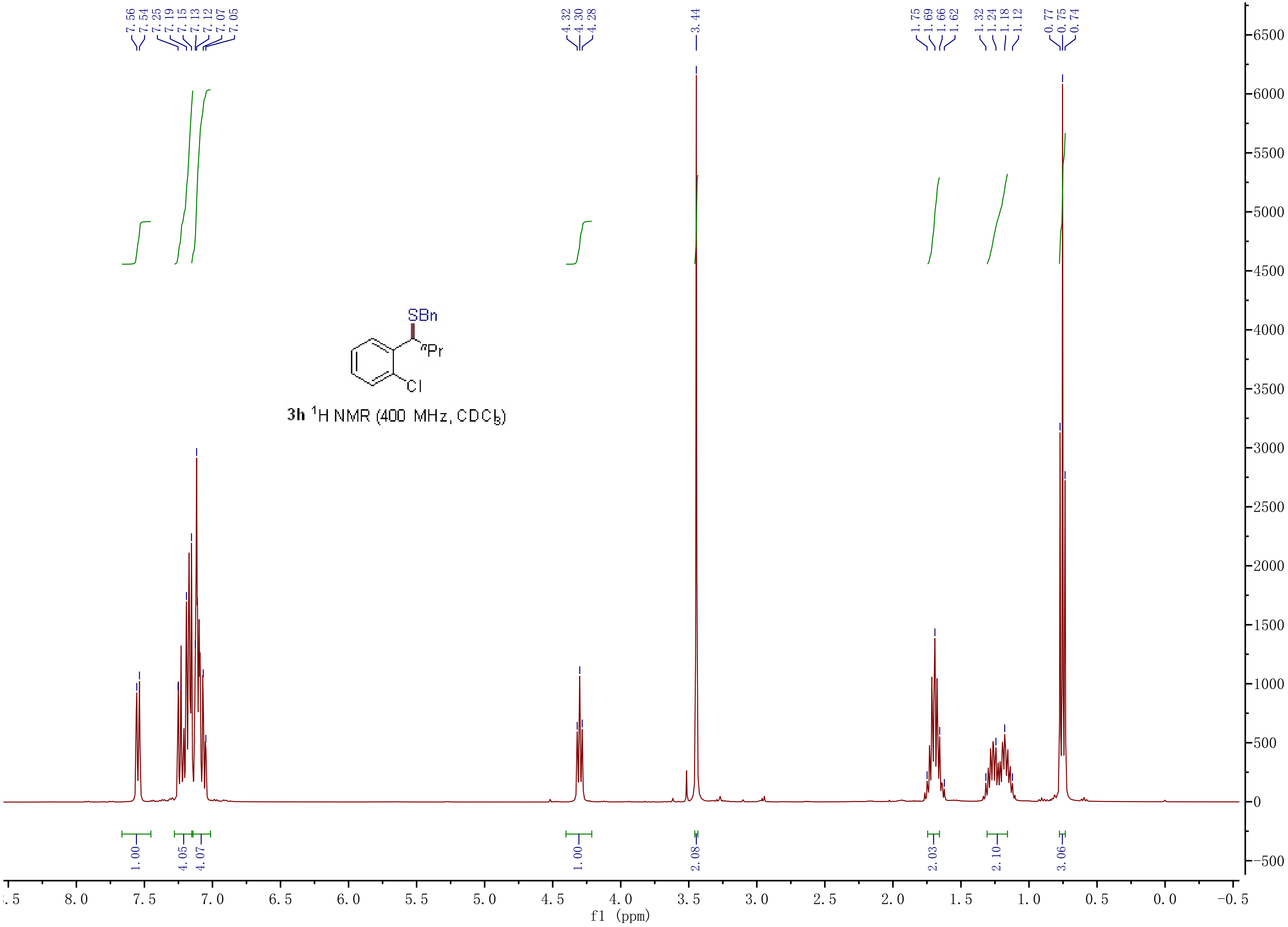

Supplementary Figure 113. ¹H NMR (400 MHz, $CDCl_3$) spectra for compound **3h**

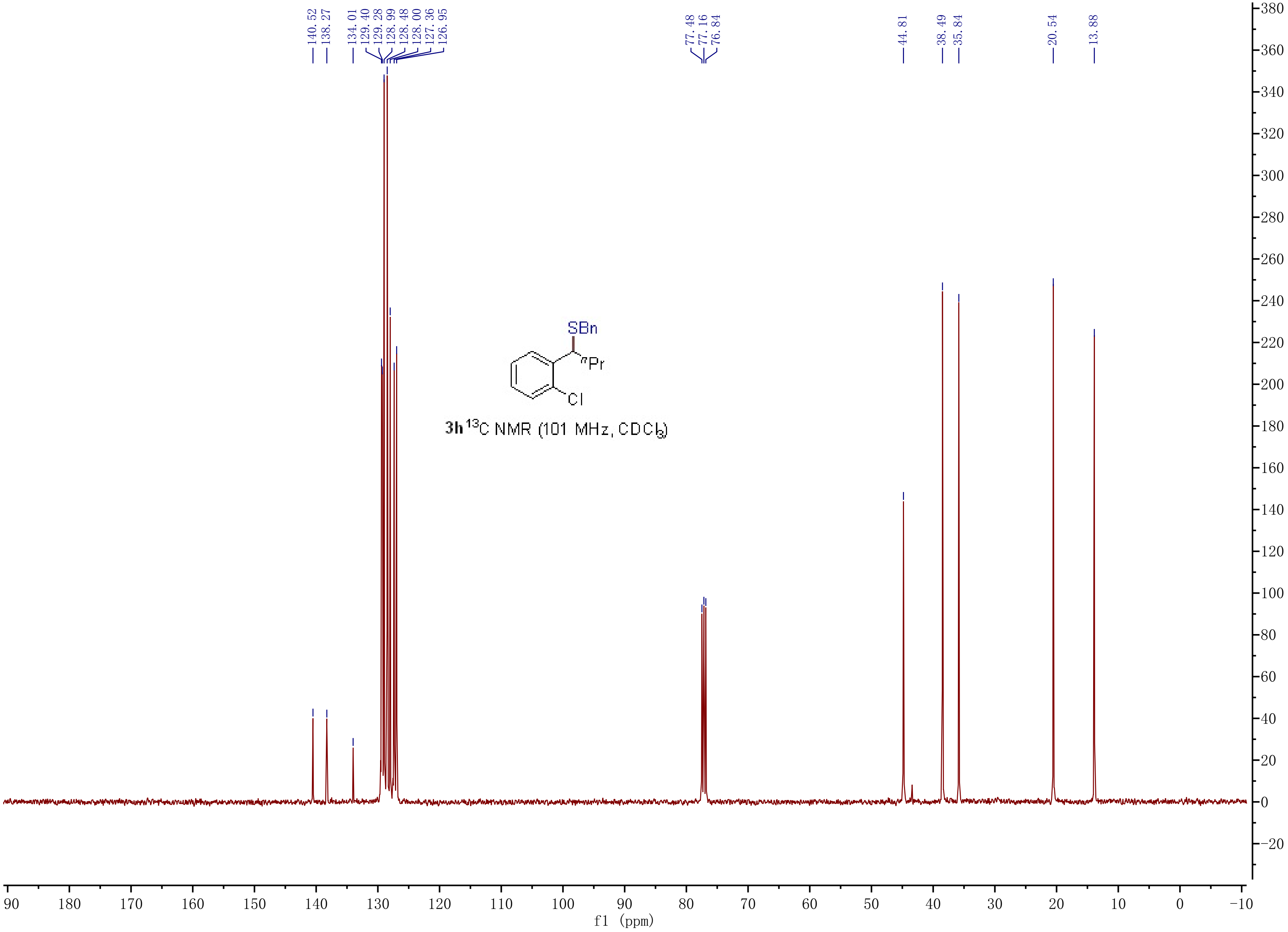

Supplementary Figure 114. ^{13}C NMR (101 MHz, CDCl$_3$) spectra for compound **3h**

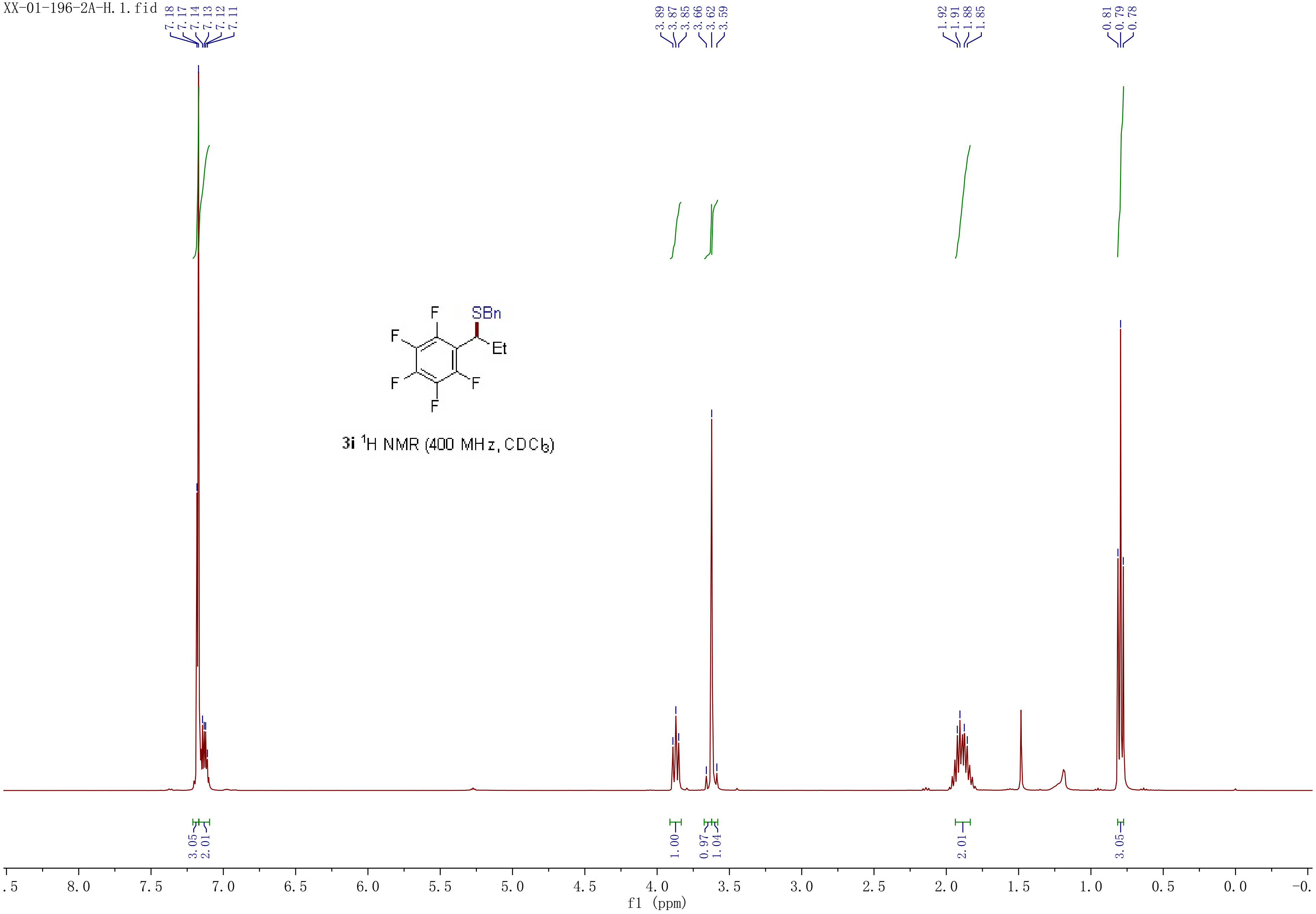

Supplementary Figure 115. ^{1}H NMR (400 MHz, $CDCl_3$) spectra for compound **3i**

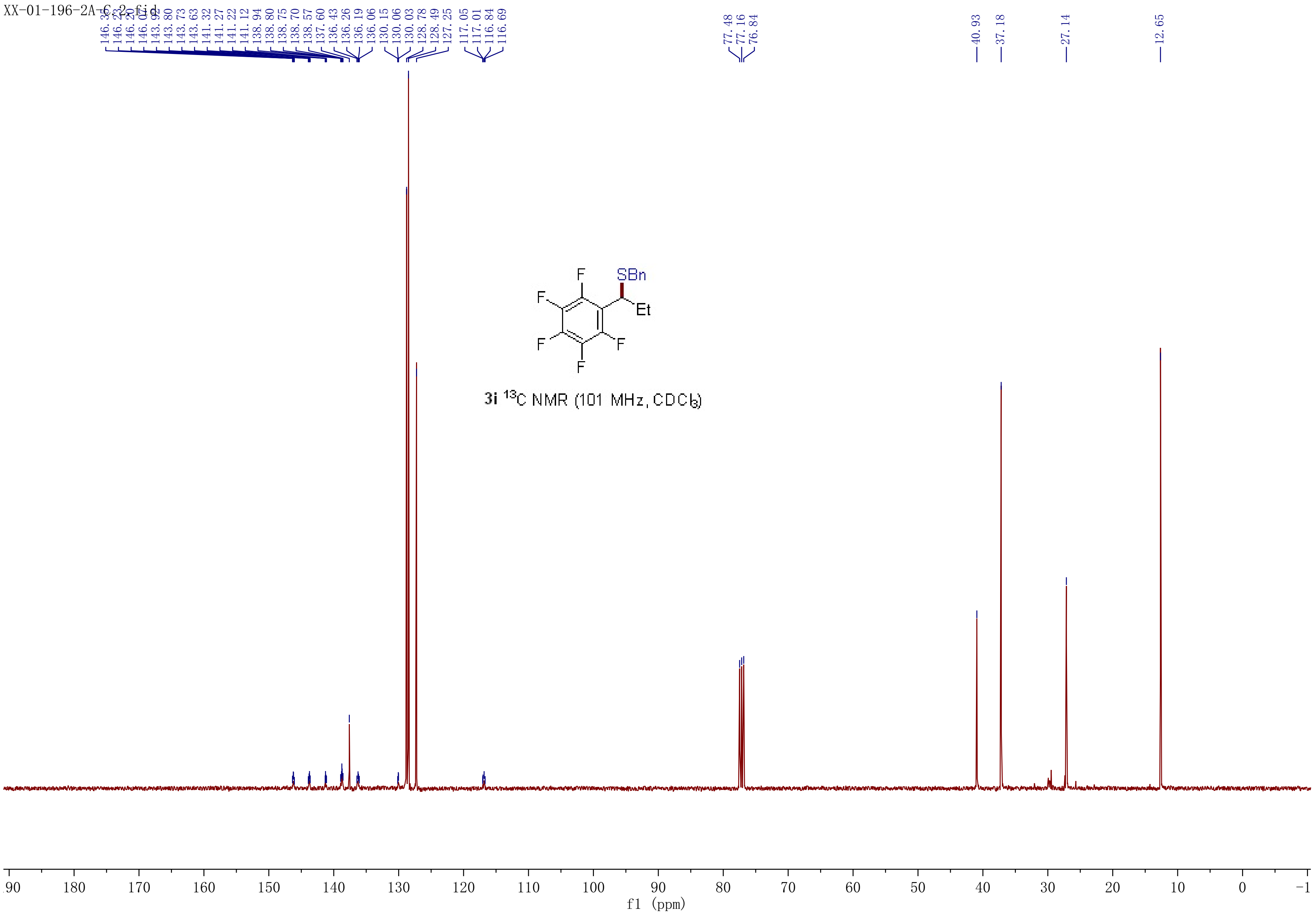

Supplementary Figure 116. ^{13}C NMR (101 MHz, CDCl$_3$) spectra for compound **3i**

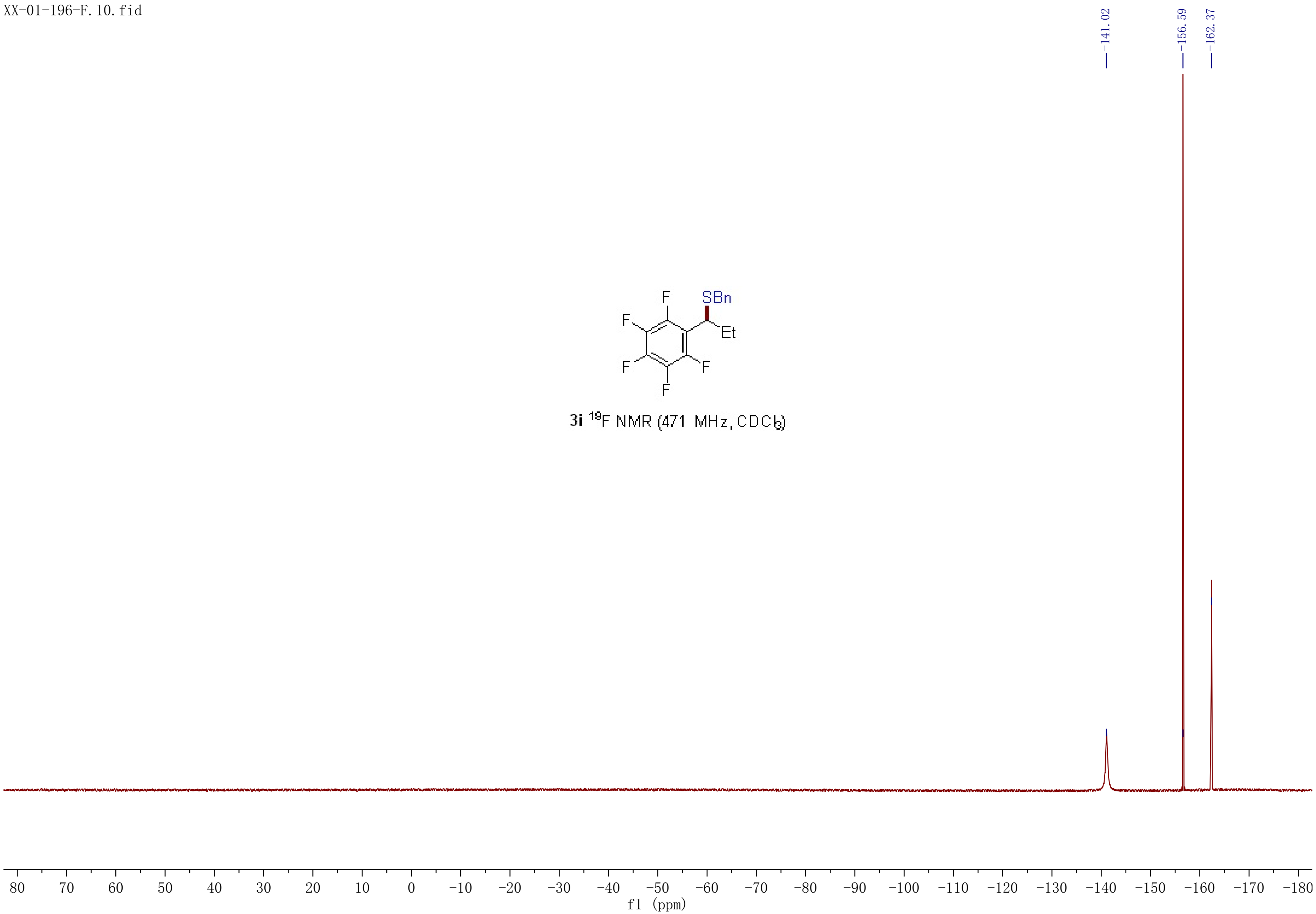

Supplementary Figure 117. ^{19}F NMR (471 MHz, $CDCl_3$) spectra for compound **3i**

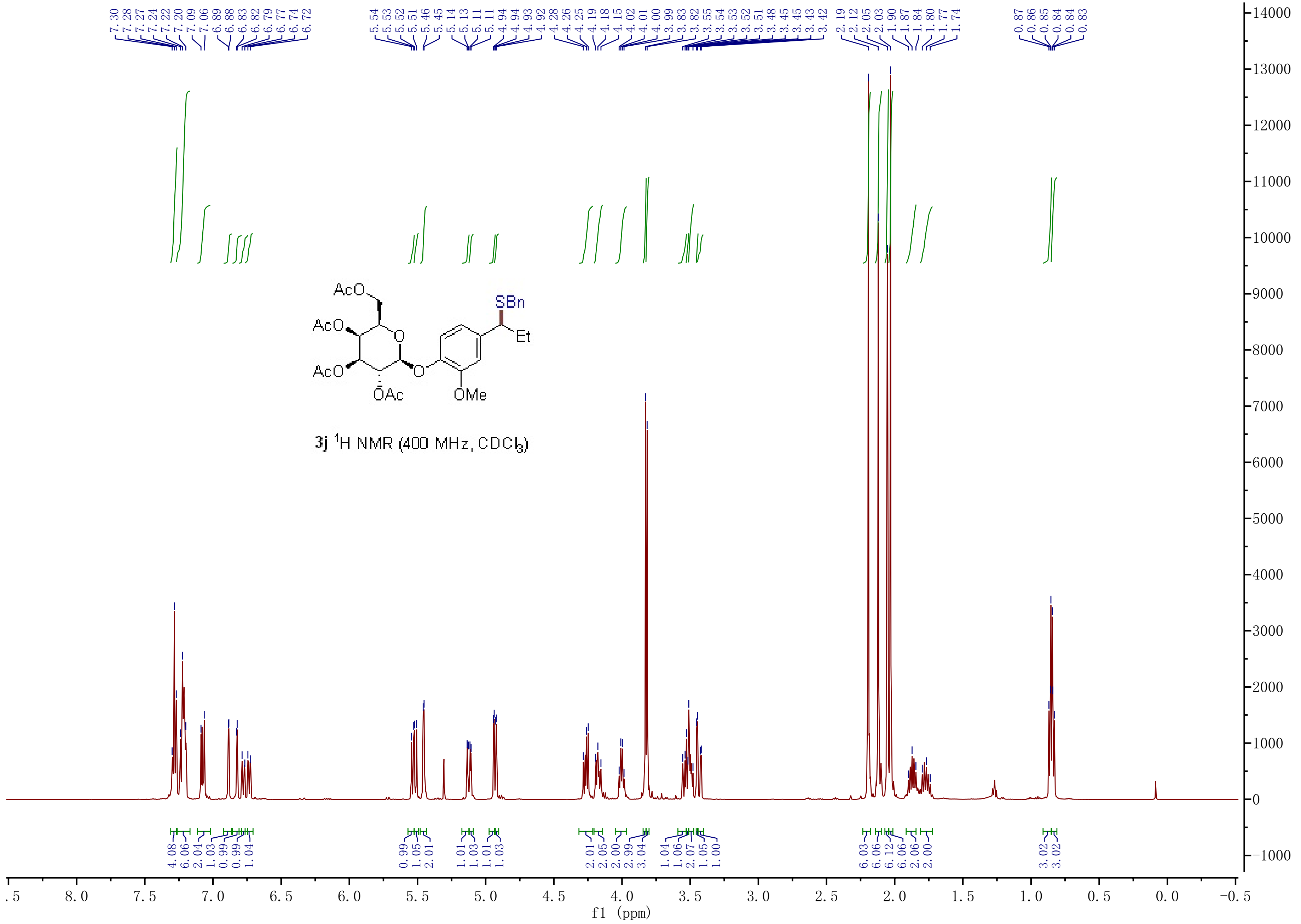

Supplementary Figure 118. ^{1}H NMR (400 MHz, $CDCl_3$) spectra for compound **3j**

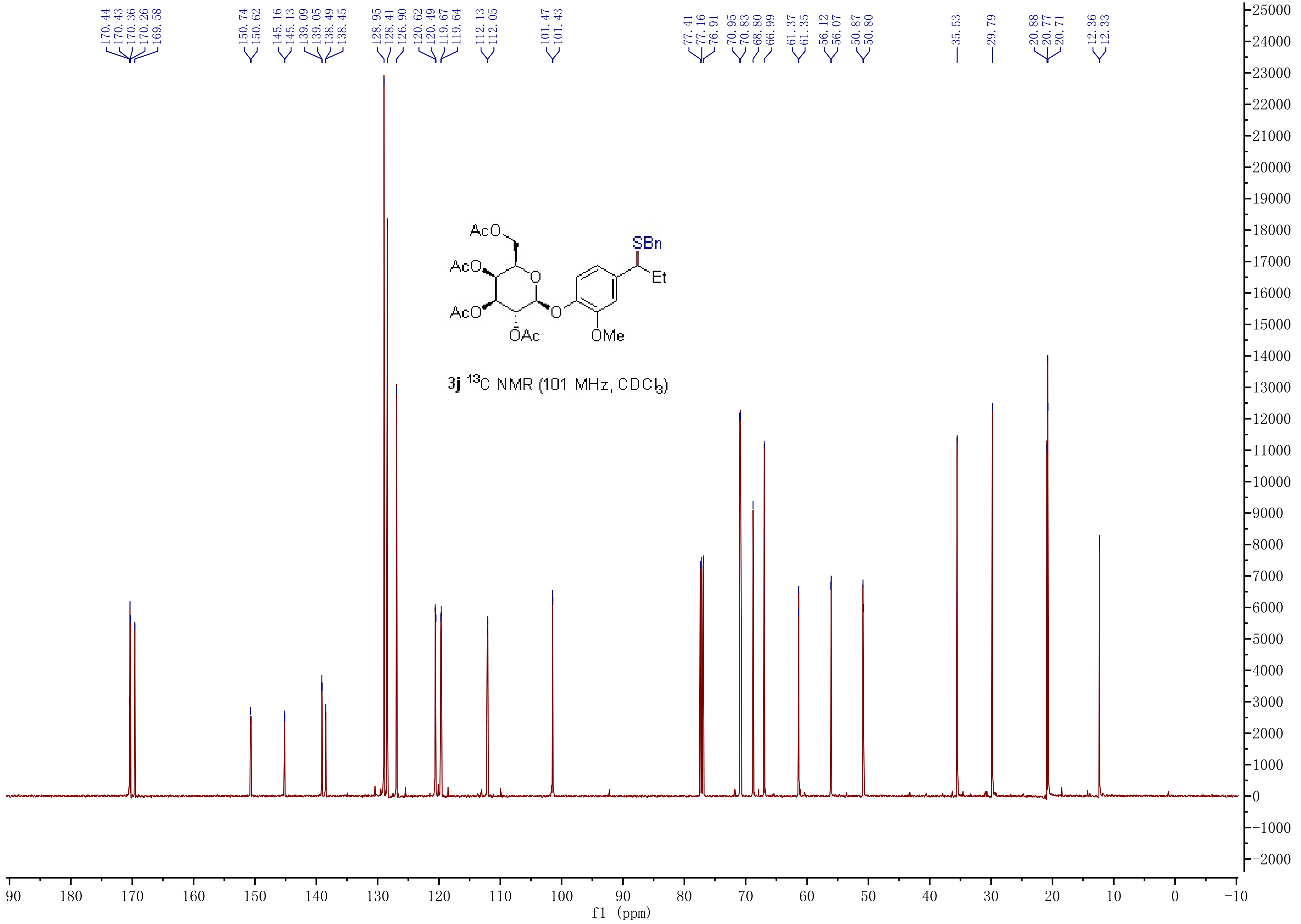

Supplementary Figure 119. ^{13}C NMR (101 MHz, $CDCl_3$) spectra for compound **3j**

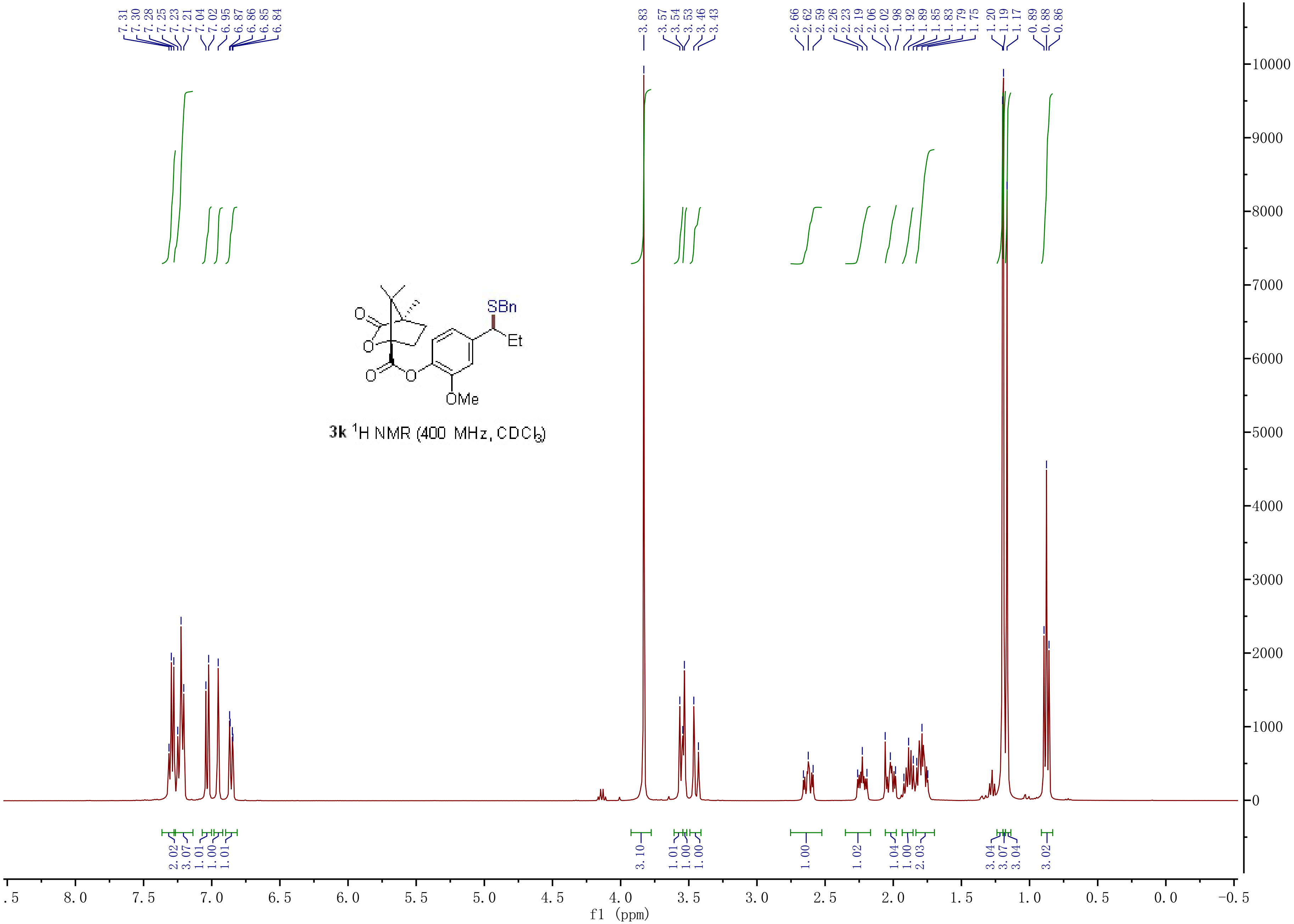

Supplementary Figure 120. ^{1}H NMR (400 MHz, $CDCl_3$) spectra for compound **3k**

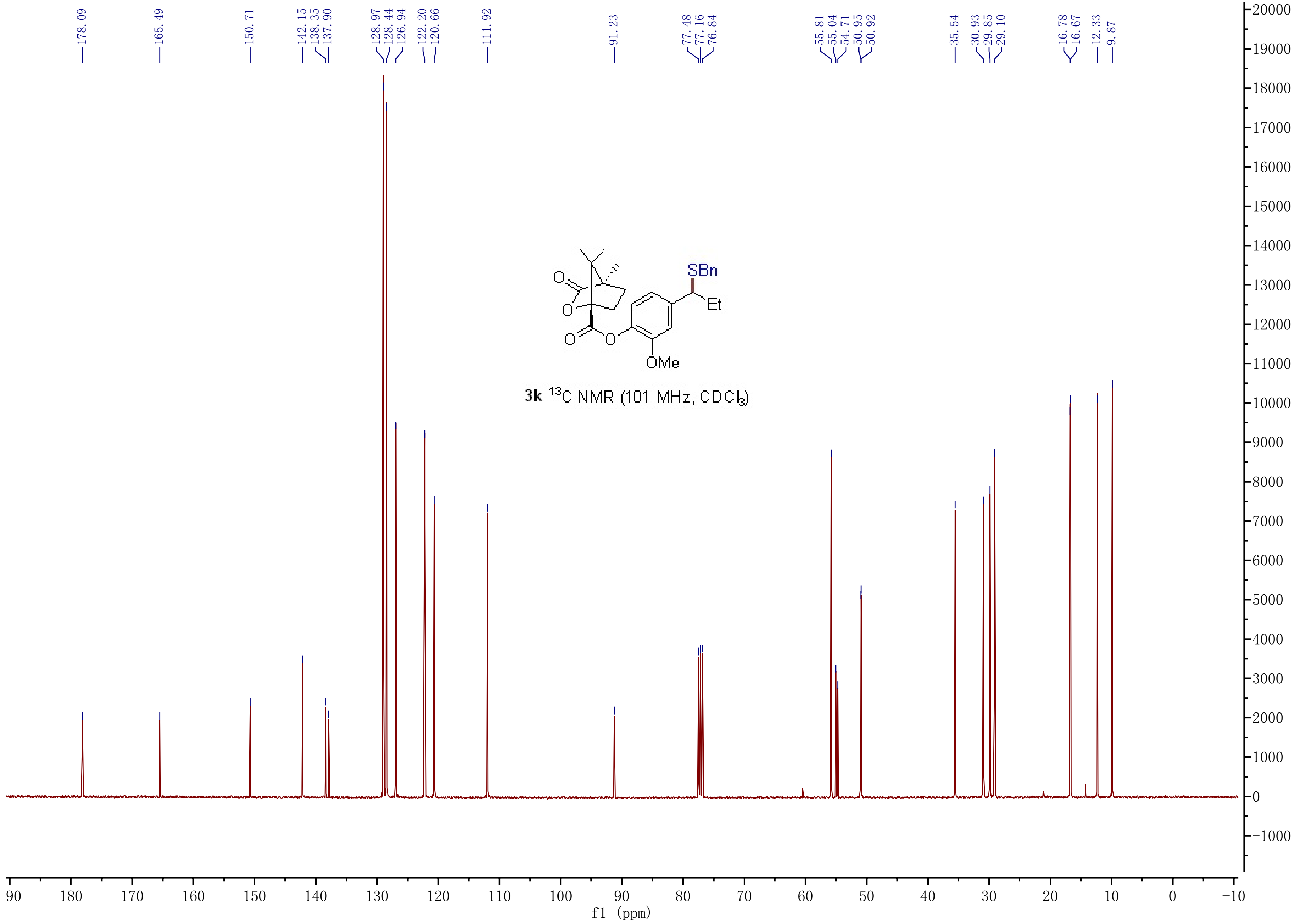

Supplementary Figure 121. ^{13}C NMR (101 MHz, $CDCl_3$) spectra for compound **3k**

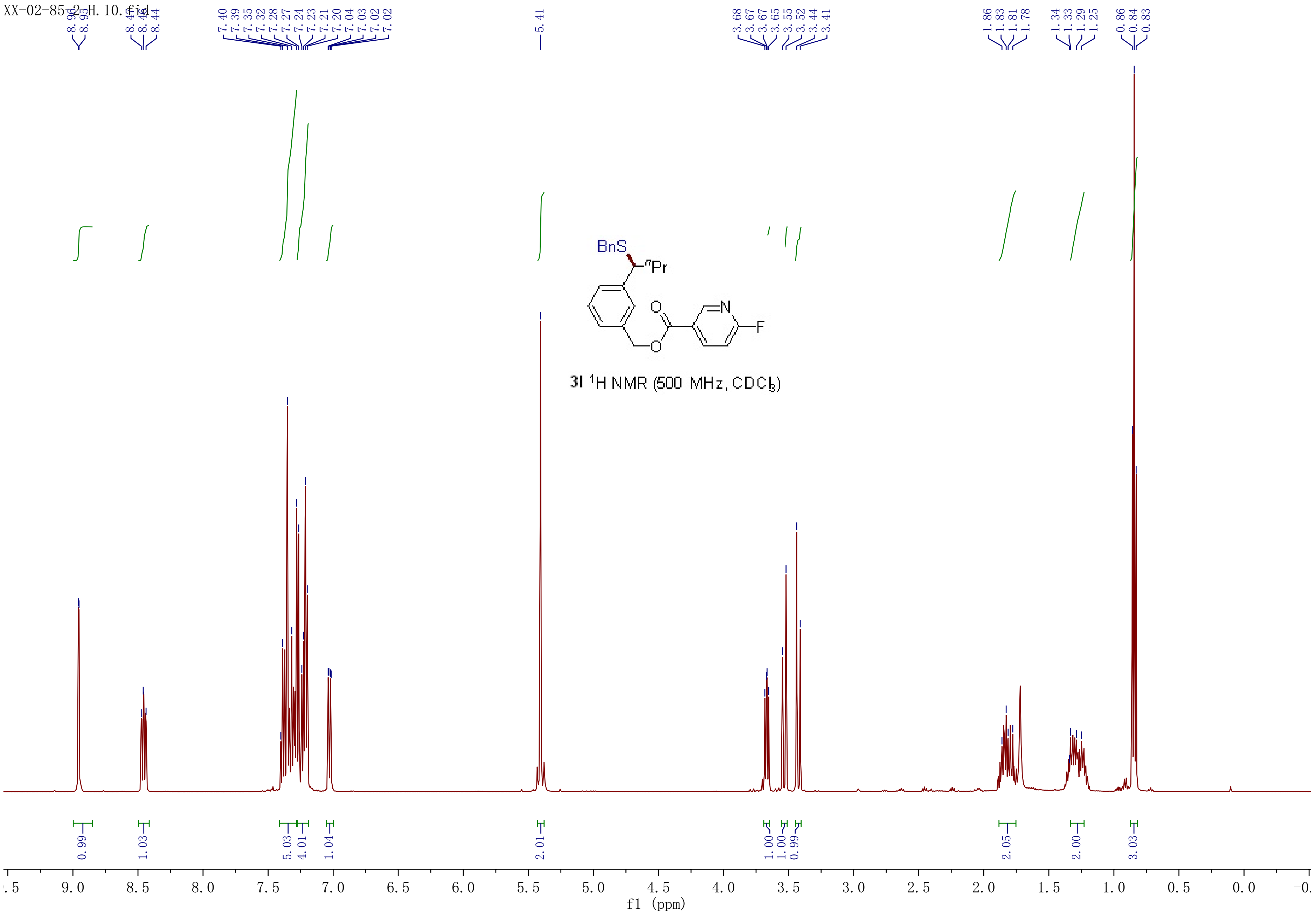

Supplementary Figure 122. ^{1}H NMR (500 MHz, $CDCl_3$) spectra for compound **3l**

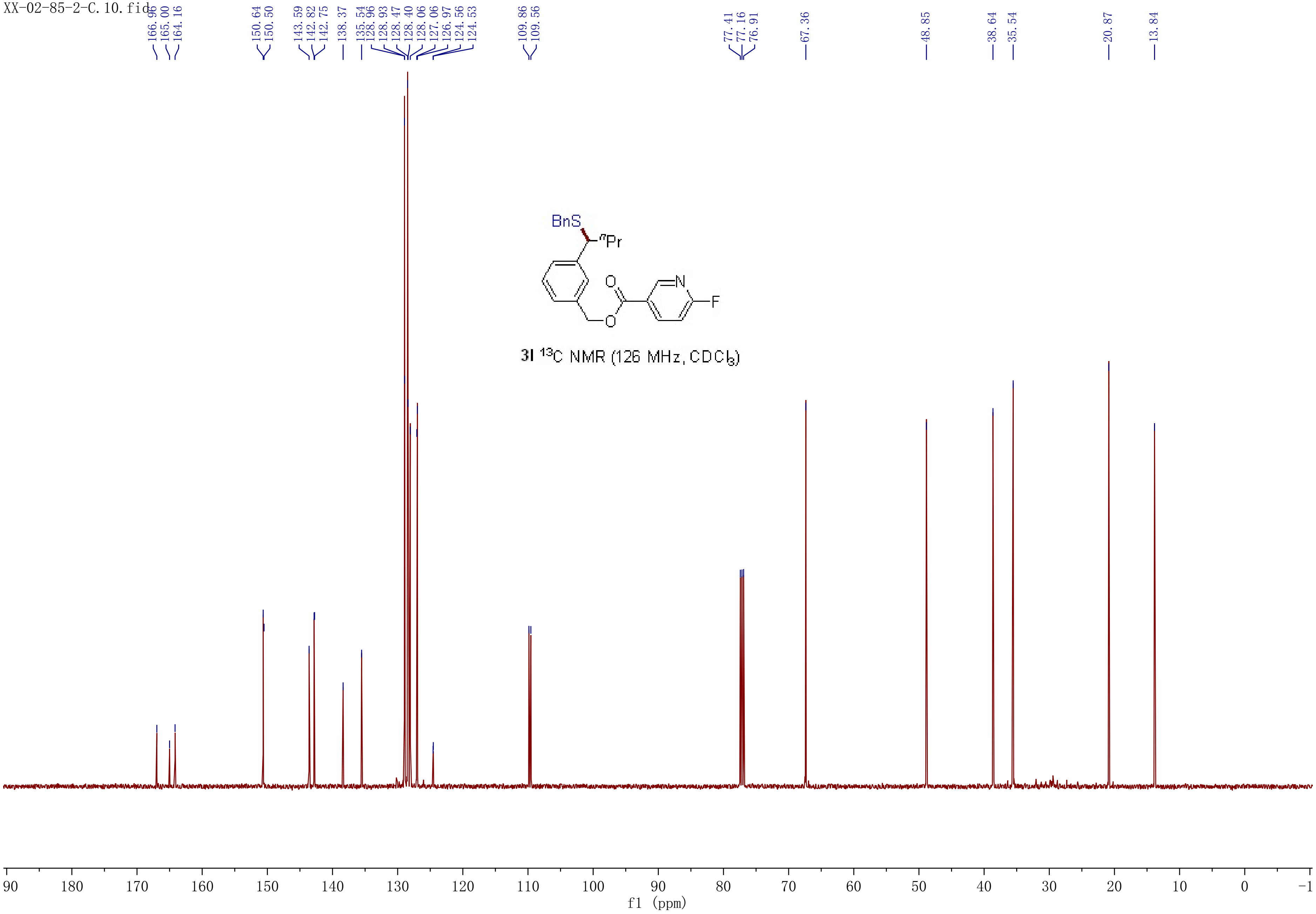

Supplementary Figure 123. ^{13}C NMR (126 MHz, CDCl$_3$) spectra for compound **3l**

XX-02-85-2-F.10.fid

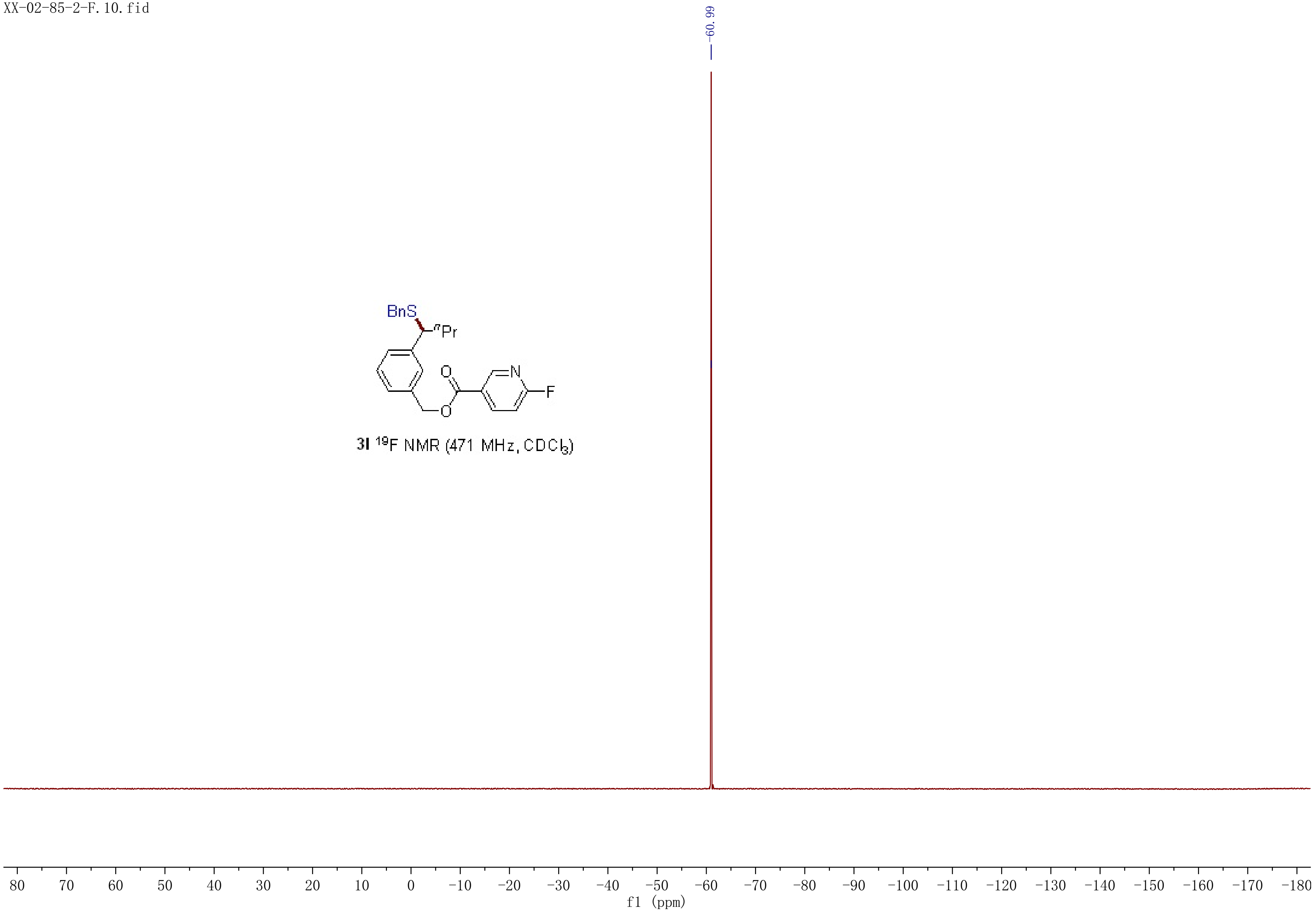

Supplementary Figure 124. ^{19}F NMR (471 MHz, $CDCl_3$) spectra for compound **3l**

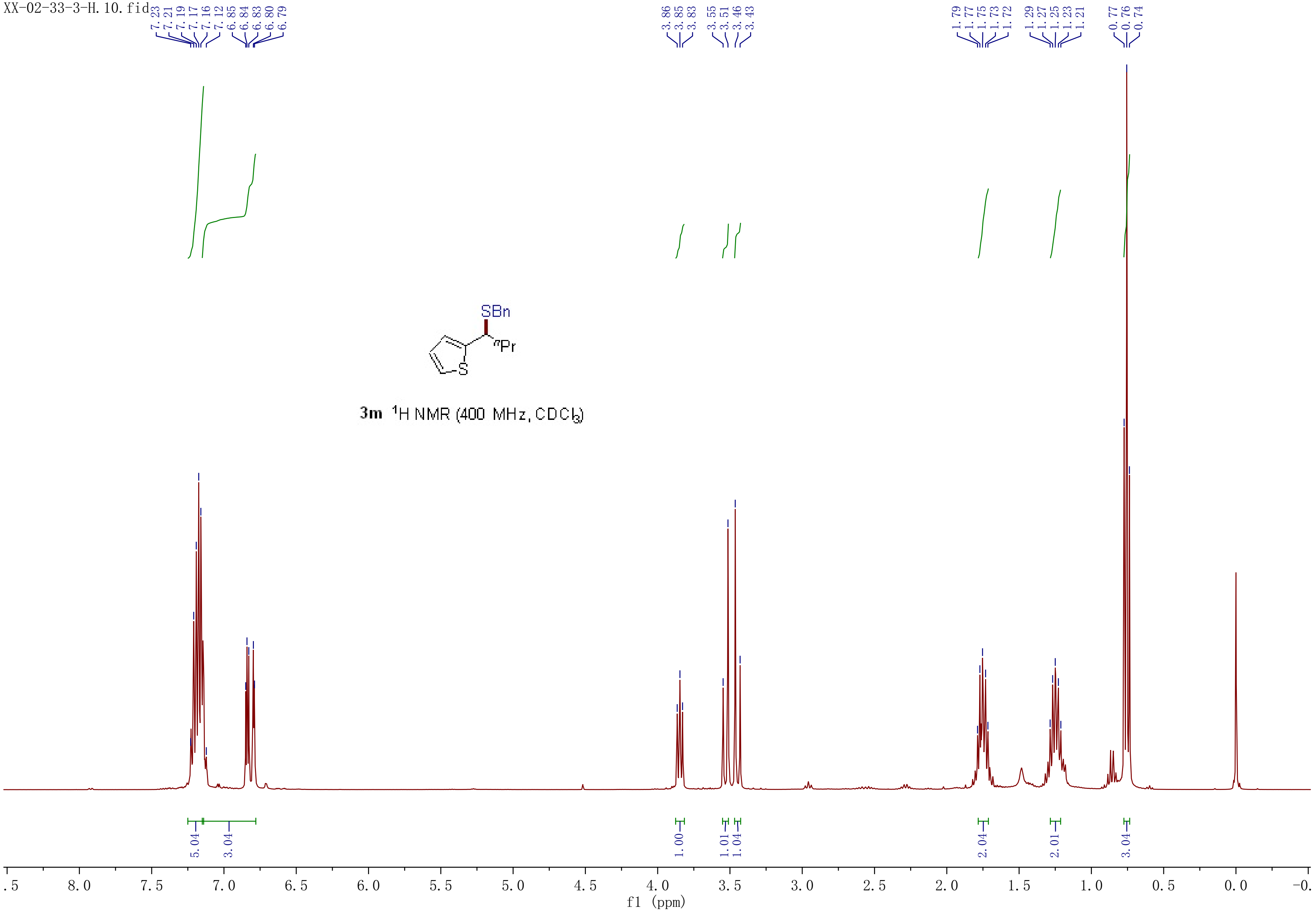

Supplementary Figure 125. ^{1}H NMR (400 MHz, $CDCl_3$) spectra for compound **3m**

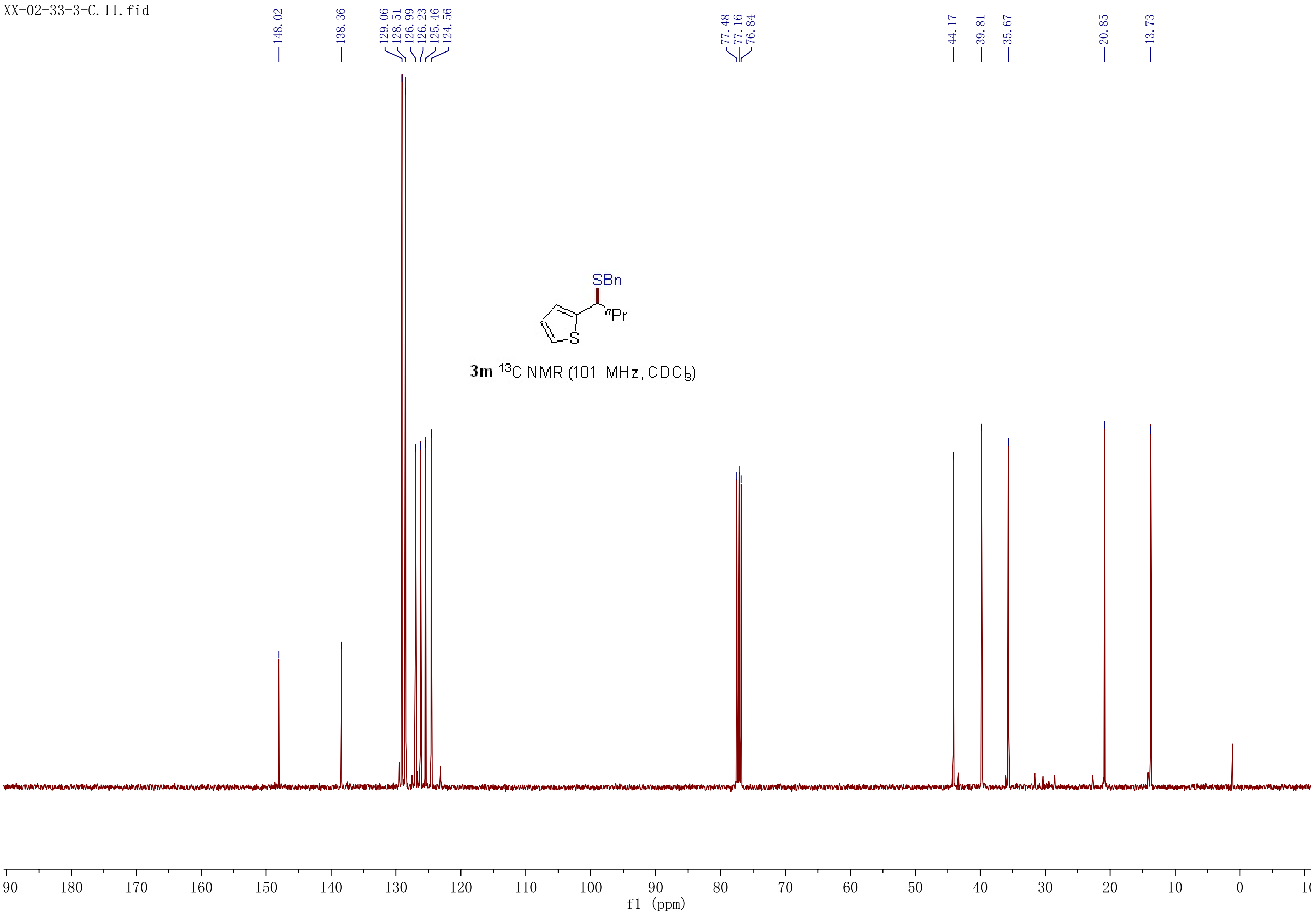

Supplementary Figure 126. ^{13}C NMR (101 MHz, $CDCl_3$) spectra for compound **3m**

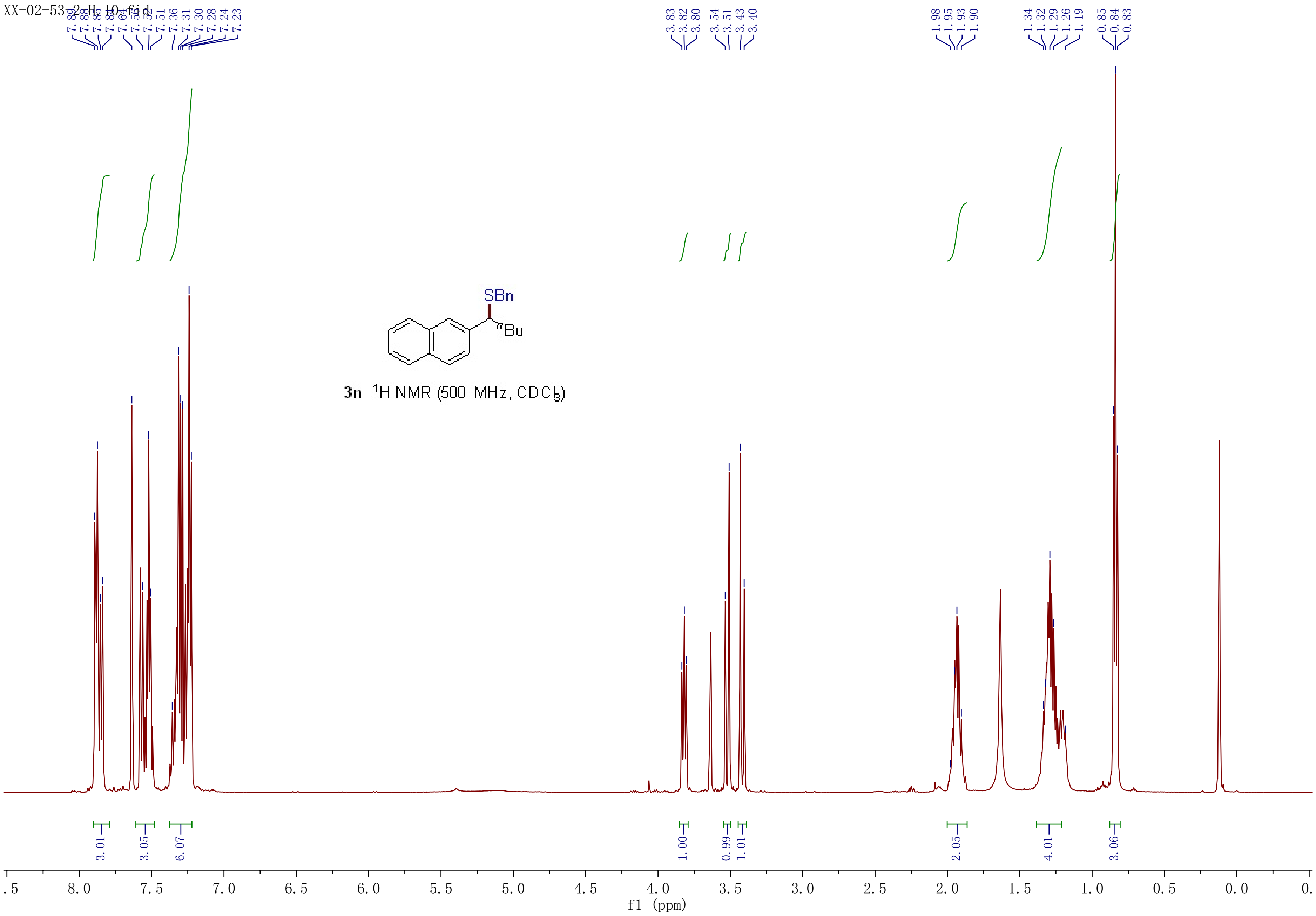

Supplementary Figure 127. ^{1}H NMR (500 MHz, $CDCl_3$) spectra for compound **3n**

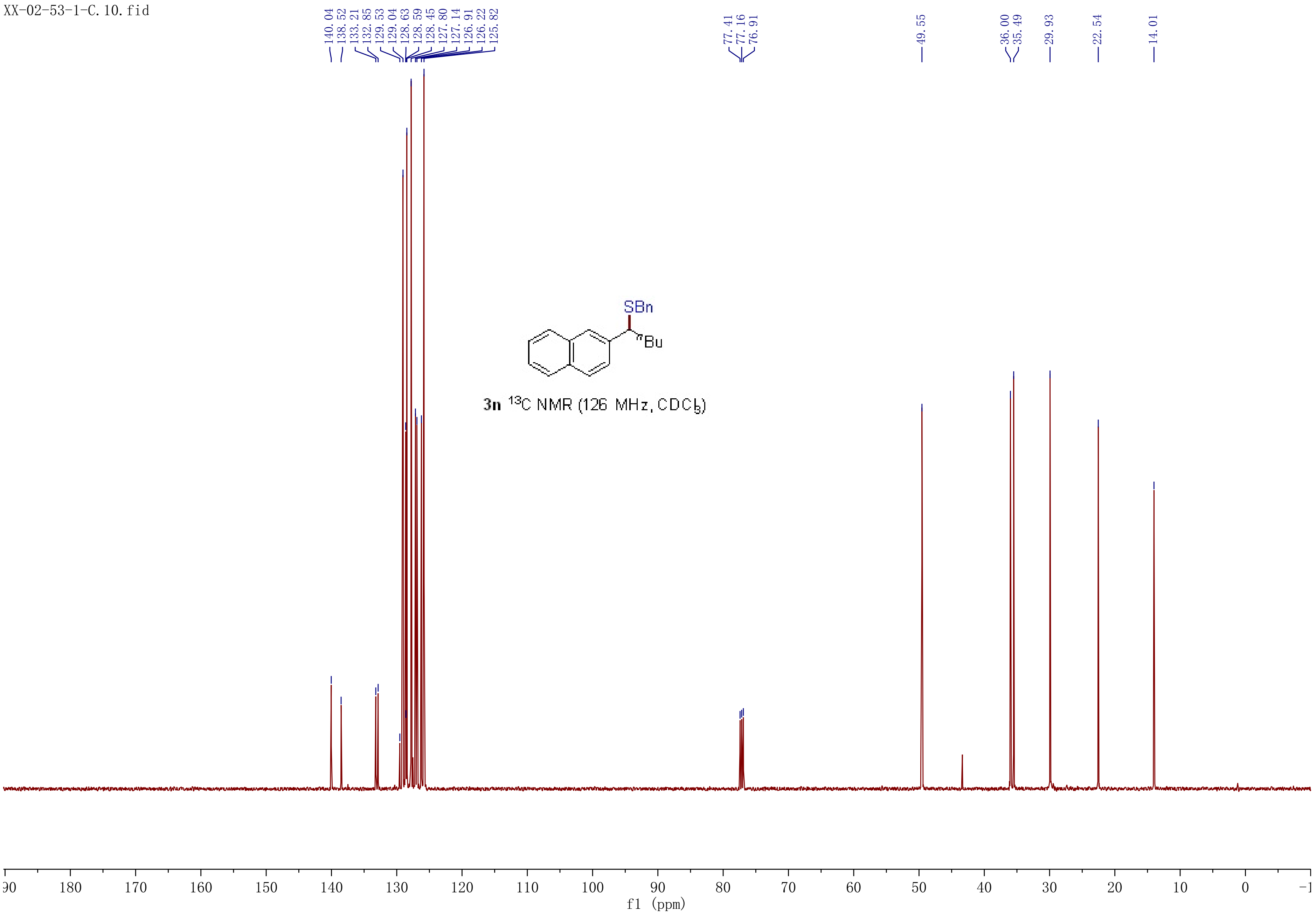

Supplementary Figure 128. ^{13}C NMR (126 MHz, $CDCl_3$) spectra for compound **3n**

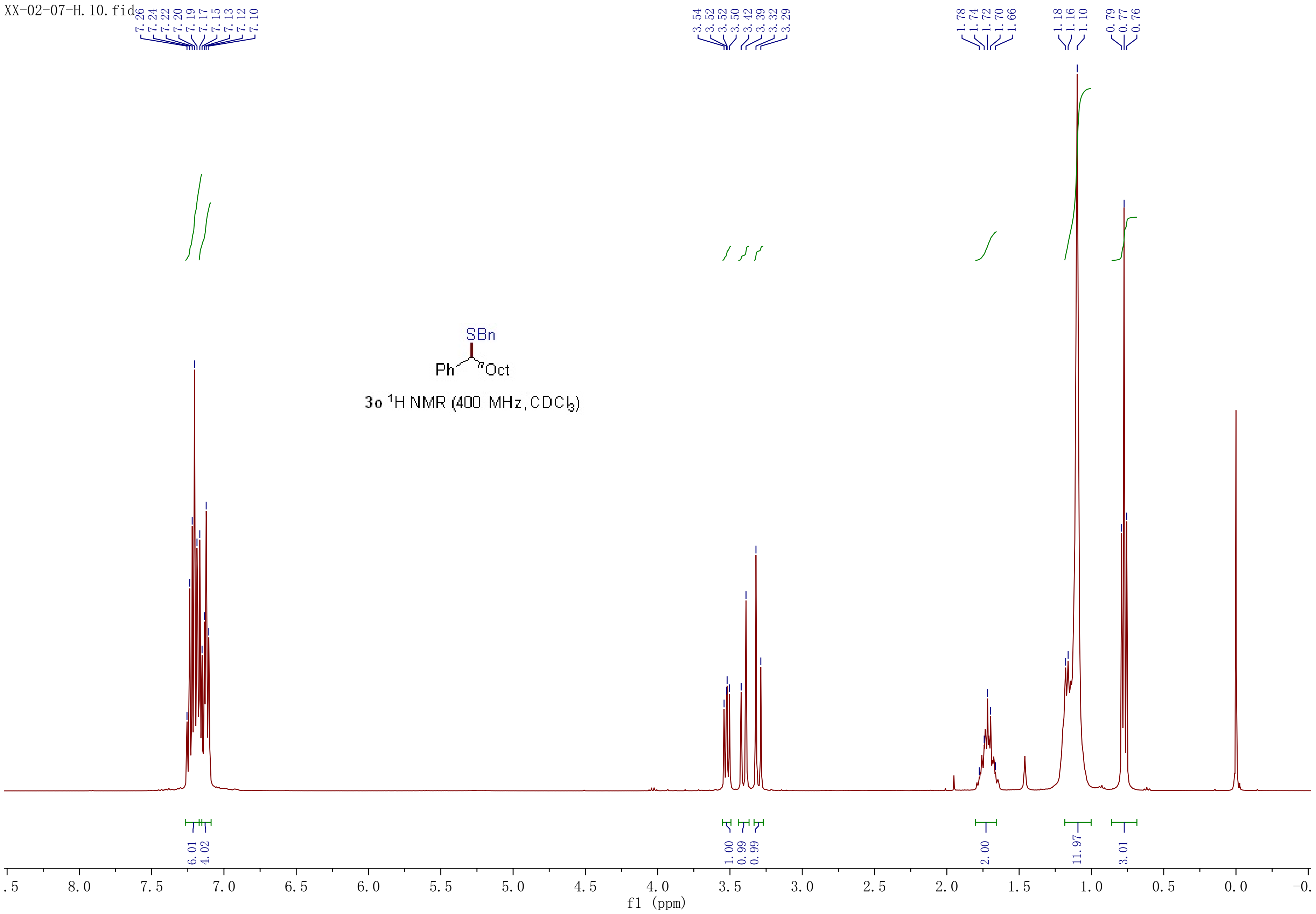

Supplementary Figure 129. ¹H NMR (400 MHz, $CDCl_3$) spectra for compound **3o**

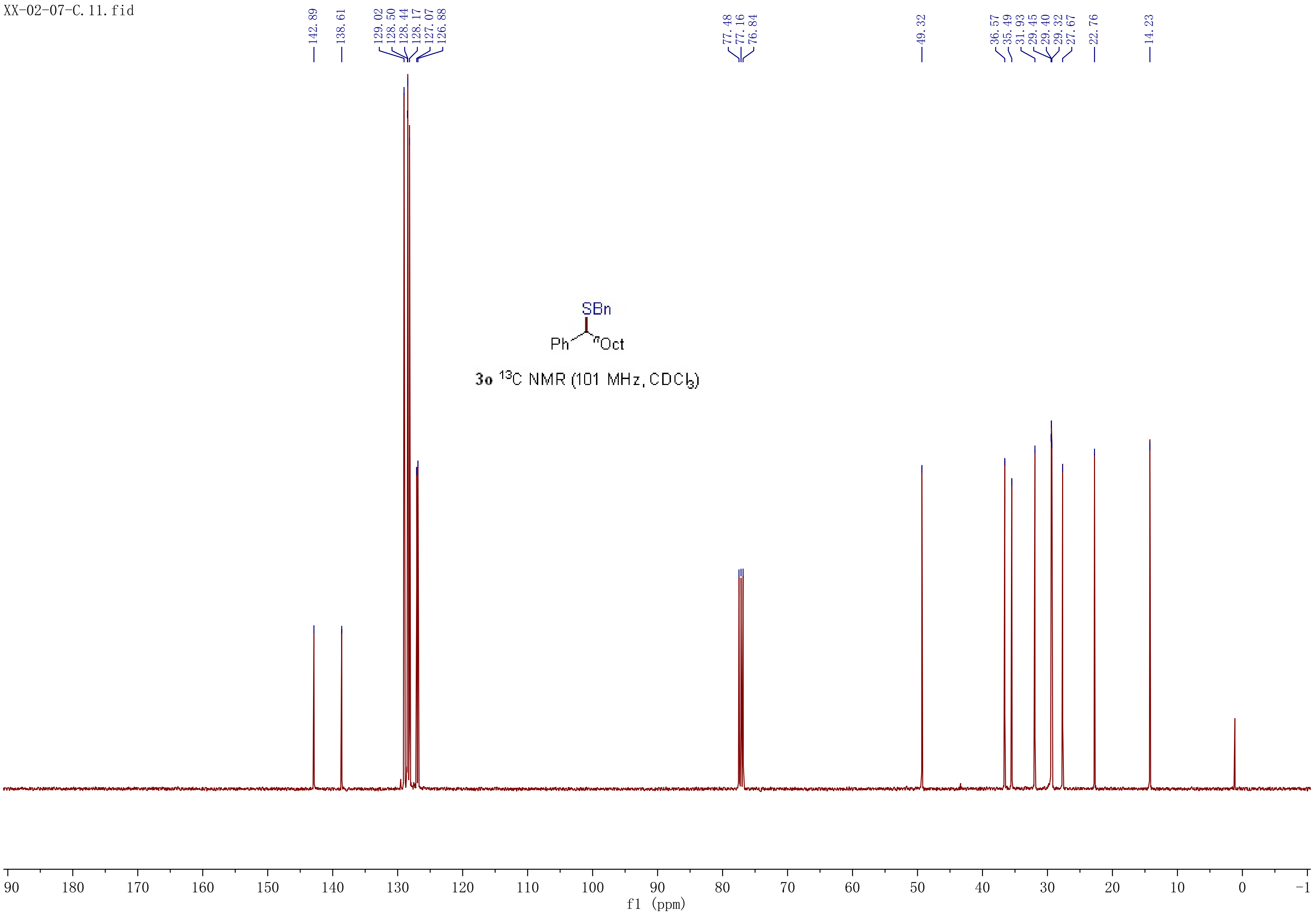

Supplementary Figure 130. ^{13}C NMR (101 MHz, $CDCl_3$) spectra for compound **3o**

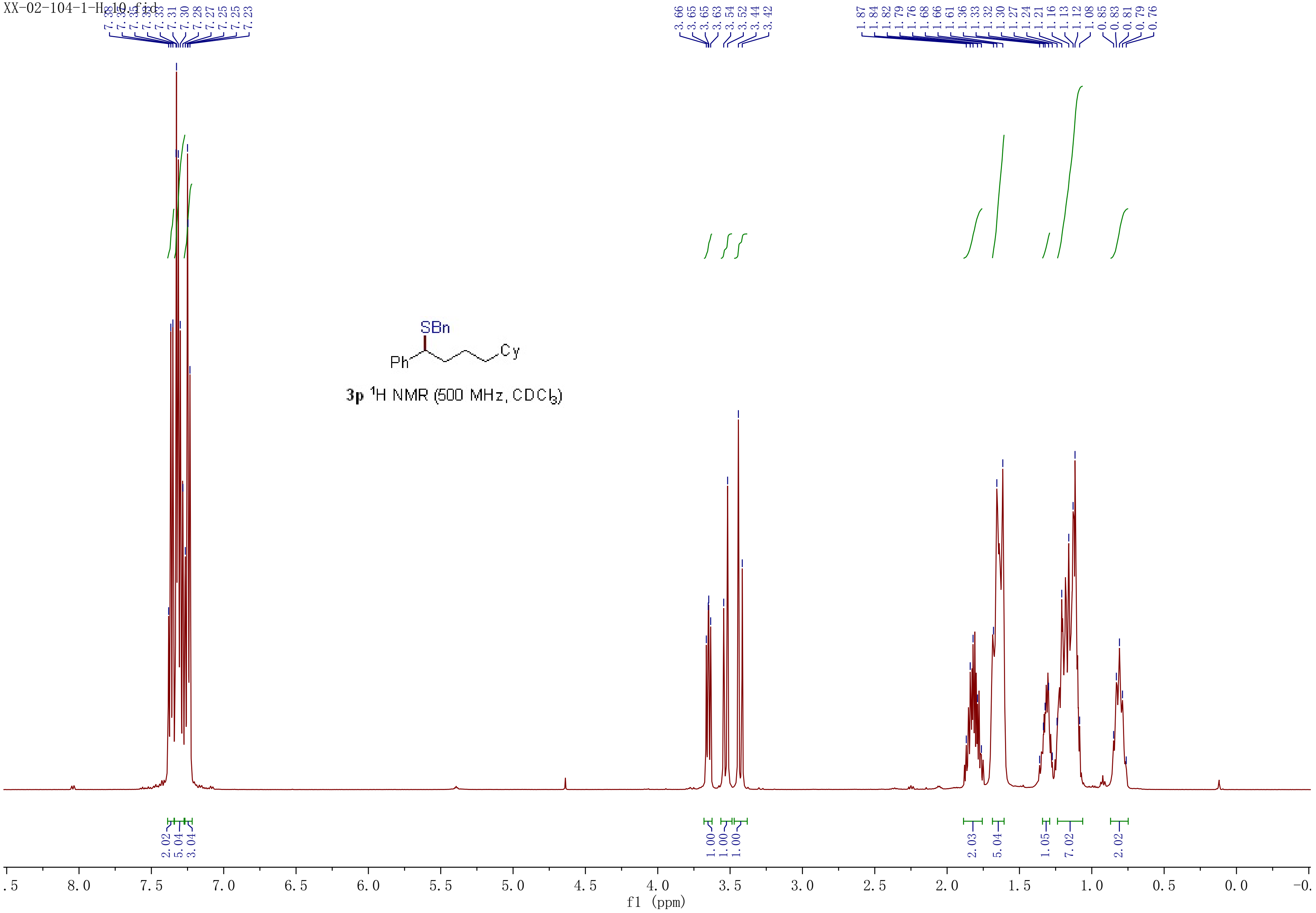

Supplementary Figure 131. ^{1}H NMR (500 MHz, $CDCl_3$) spectra for compound **3p**

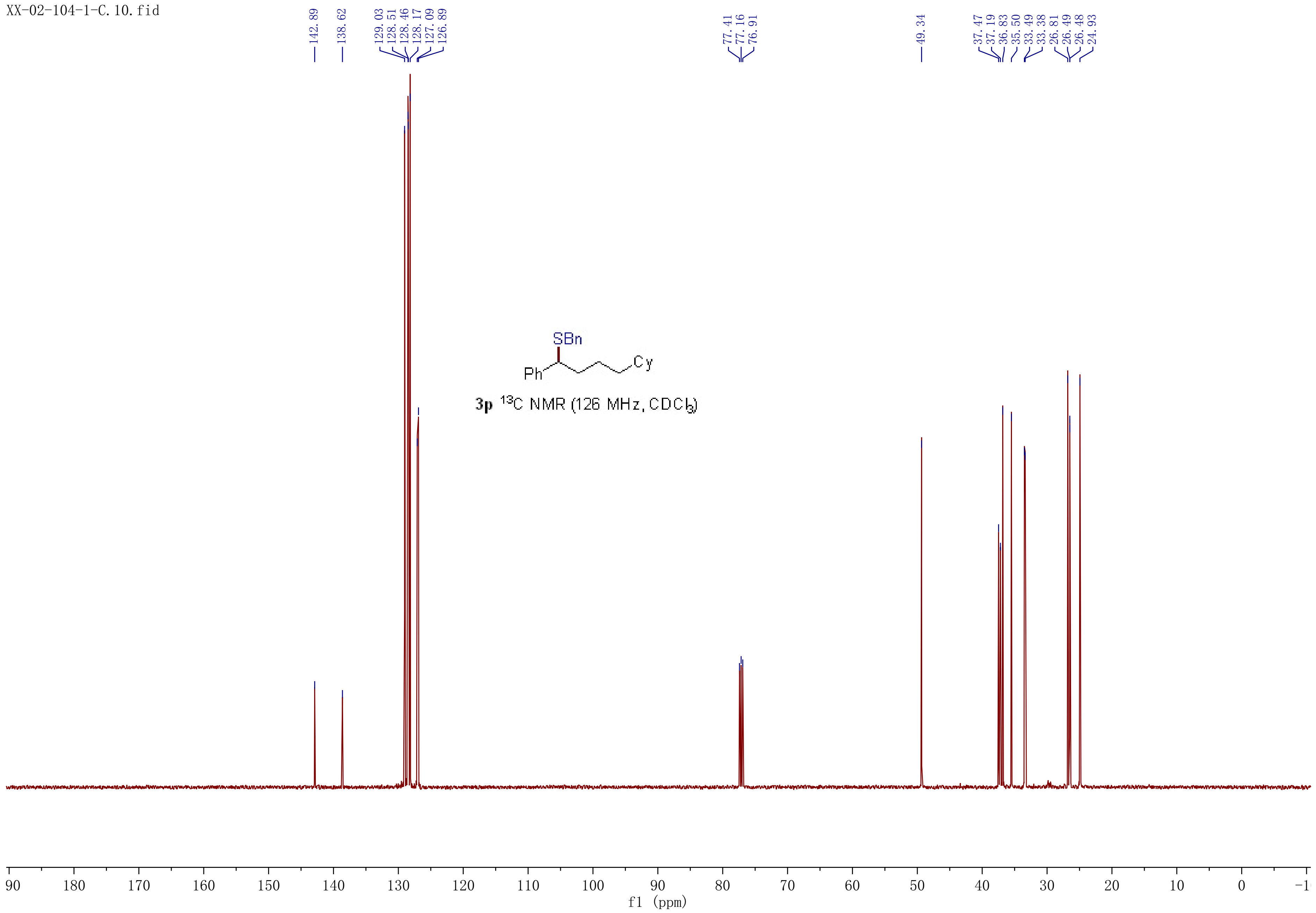

Supplementary Figure 132. ^{13}C NMR (126 MHz, $CDCl_3$) spectra for compound **3p**

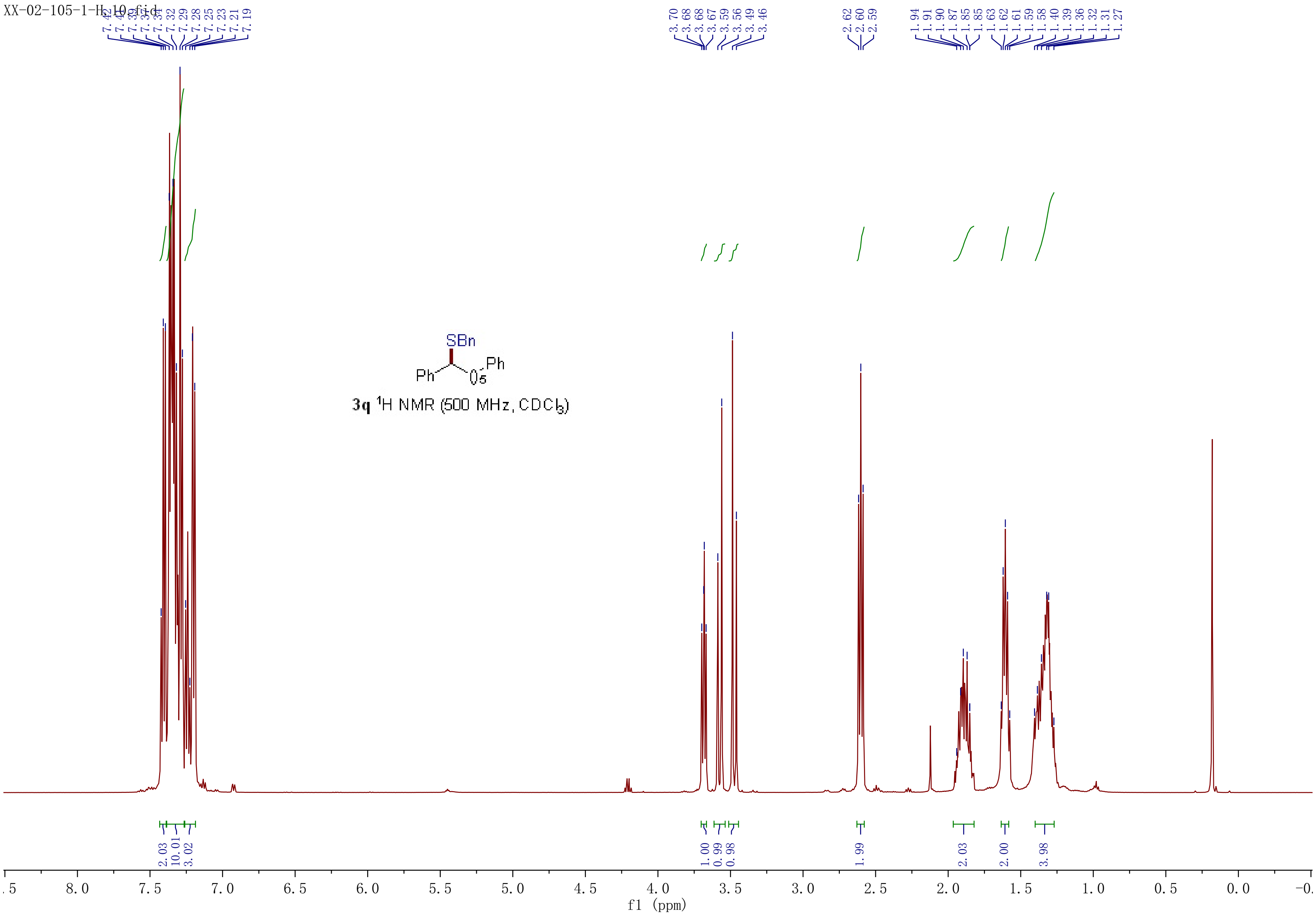

Supplementary Figure 133. ^{1}H NMR (500 MHz, $CDCl_3$) spectra for compound **3q**

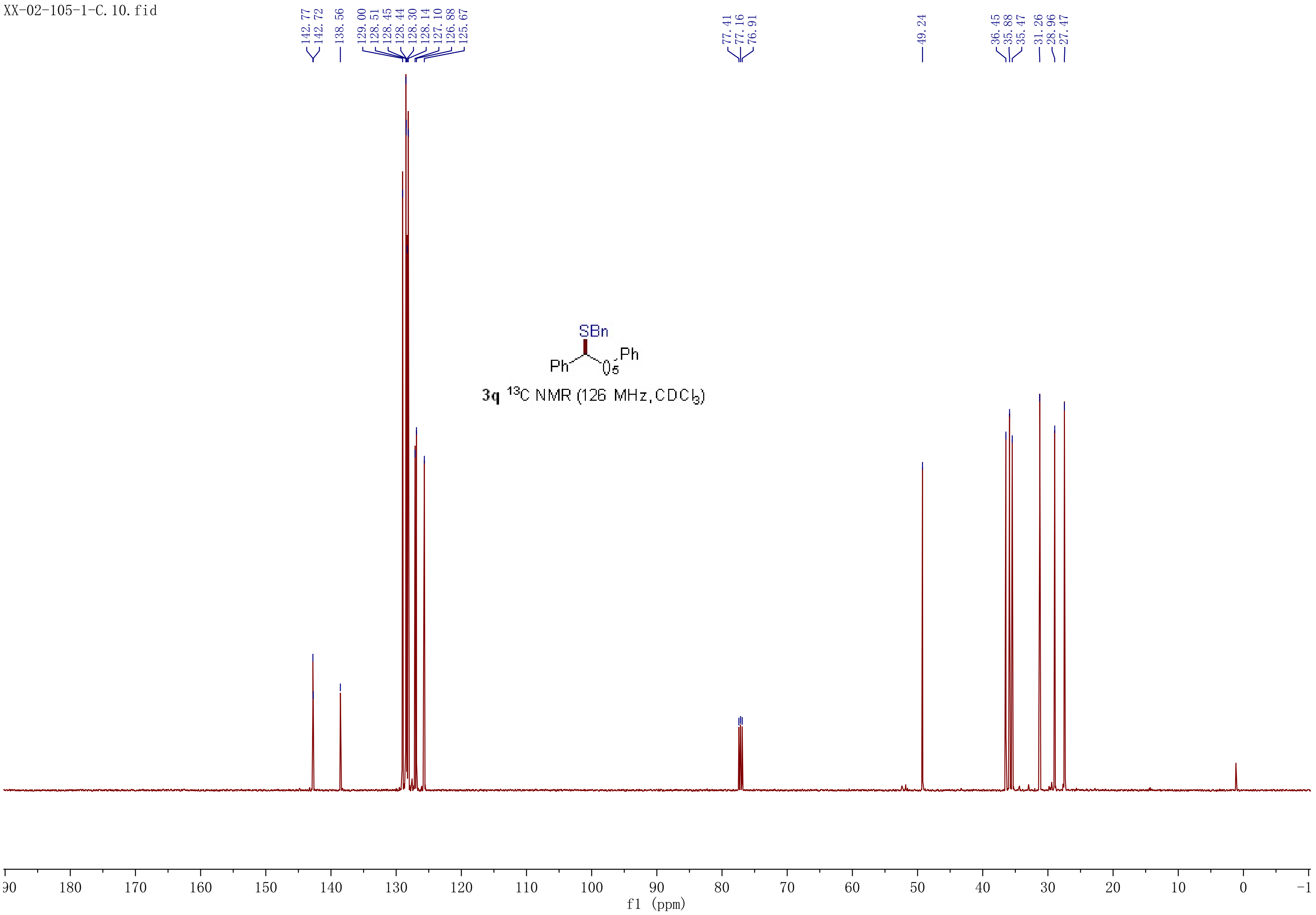

Supplementary Figure 134. ^{13}C NMR (126 MHz, $CDCl_3$) spectra for compound **3q**

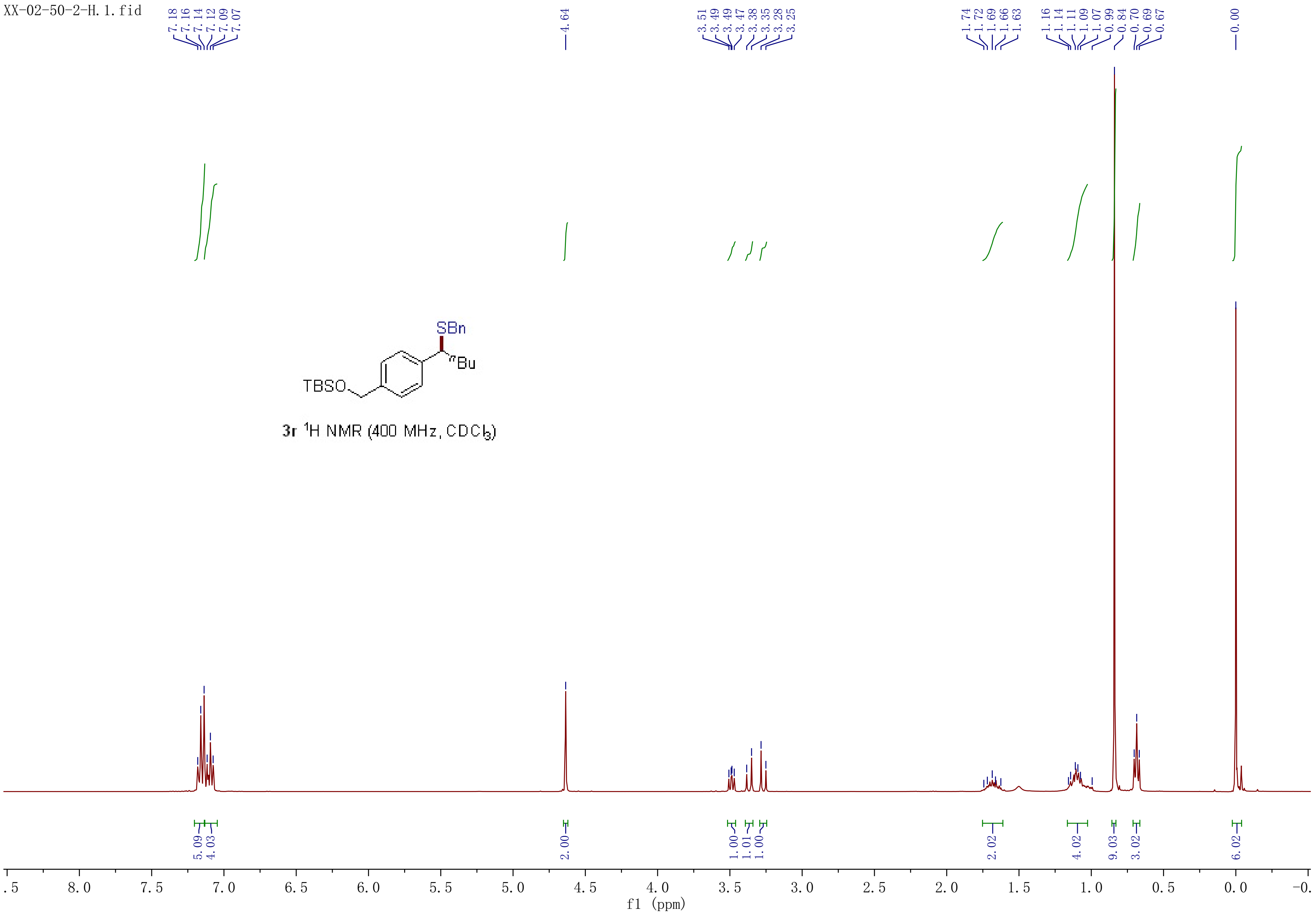

Supplementary Figure 135. ^{1}H NMR (400 MHz, $CDCl_3$) spectra for compound **3r**

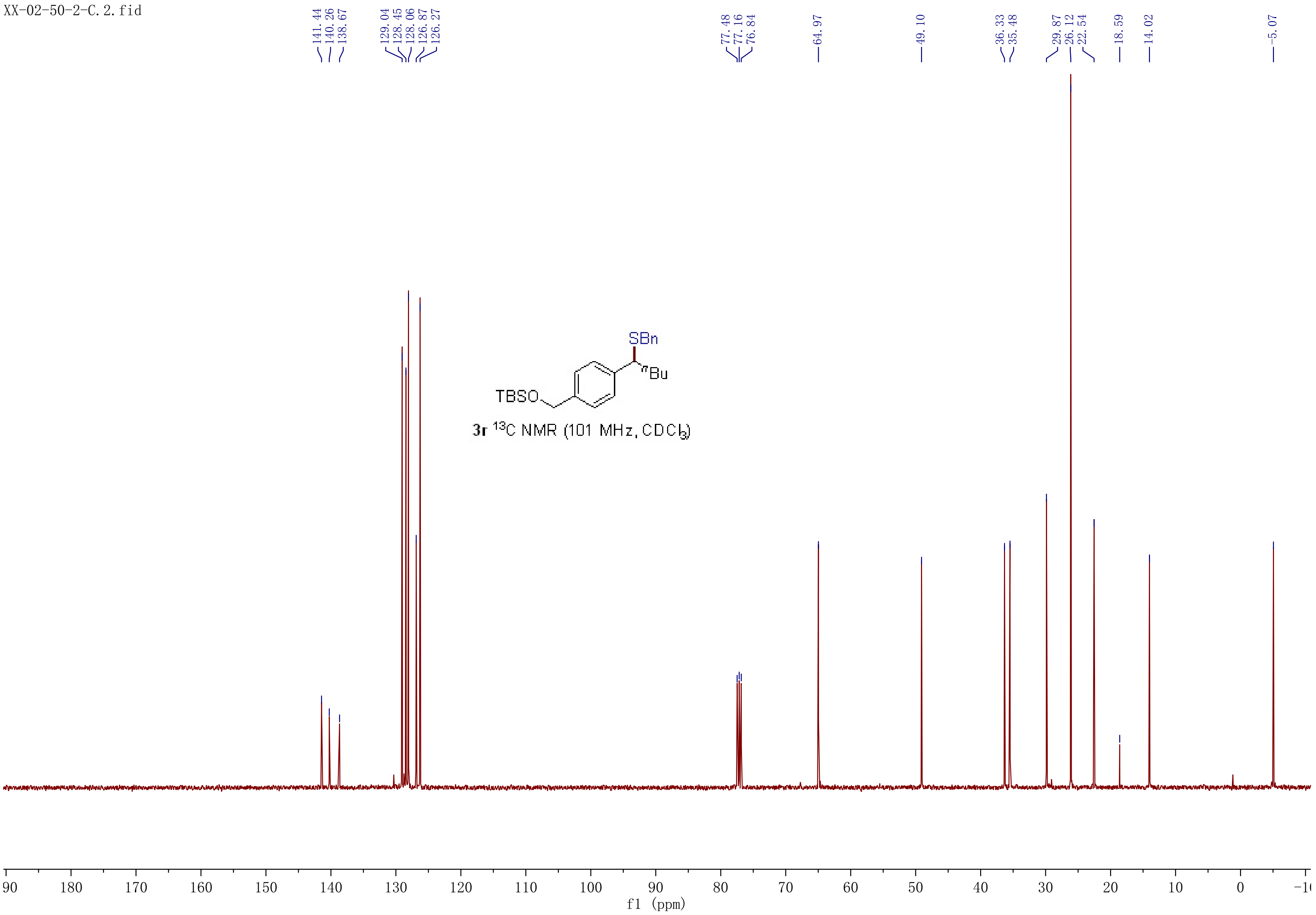

Supplementary Figure 136. ^{13}C NMR (101 MHz, $CDCl_3$) spectra for compound **3r**

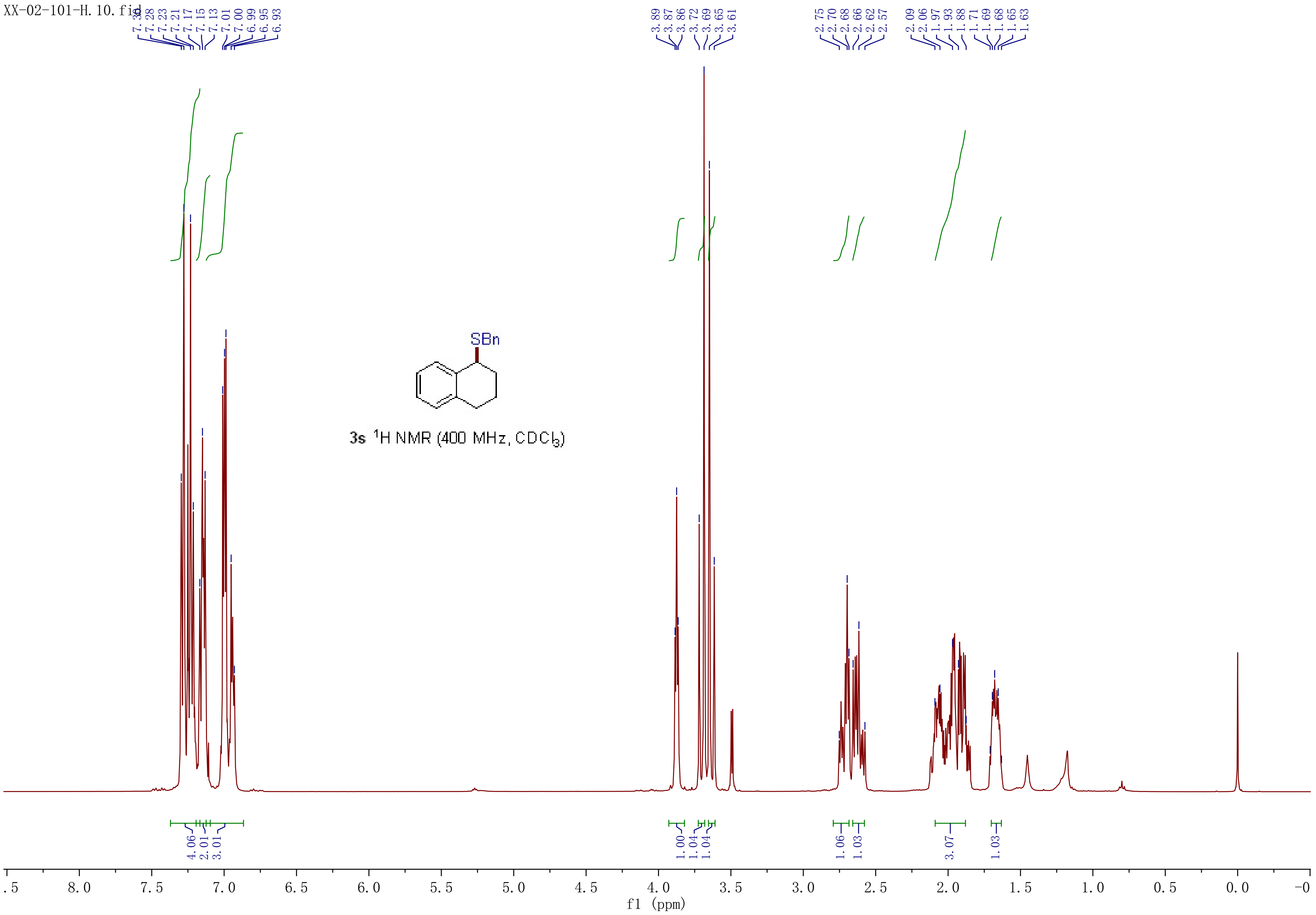

Supplementary Figure 137. ^{1}H NMR (400 MHz, $CDCl_3$) spectra for compound **3s**

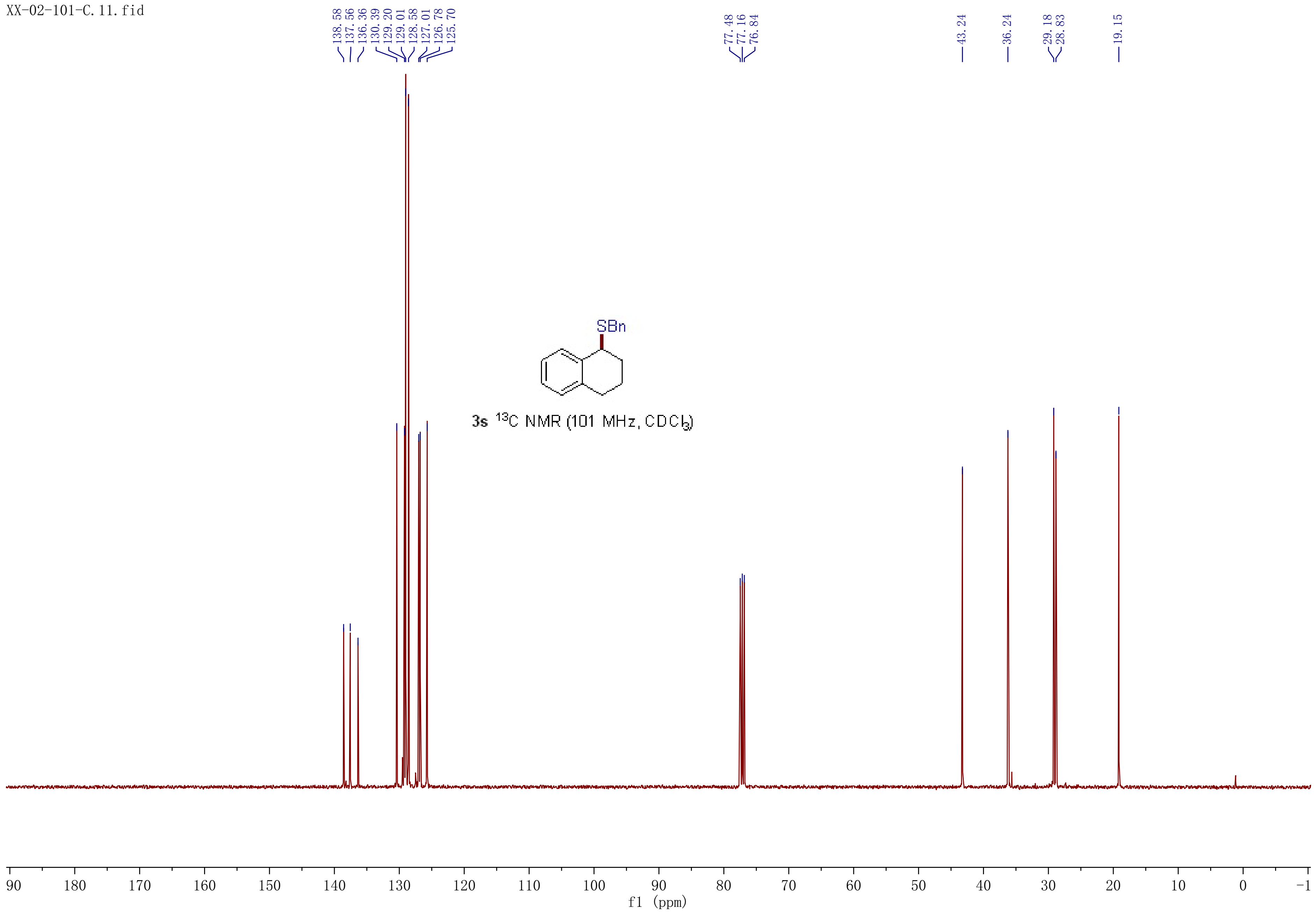

Supplementary Figure 138. ^{13}C NMR (101 MHz, $CDCl_3$) spectra for compound **3s**

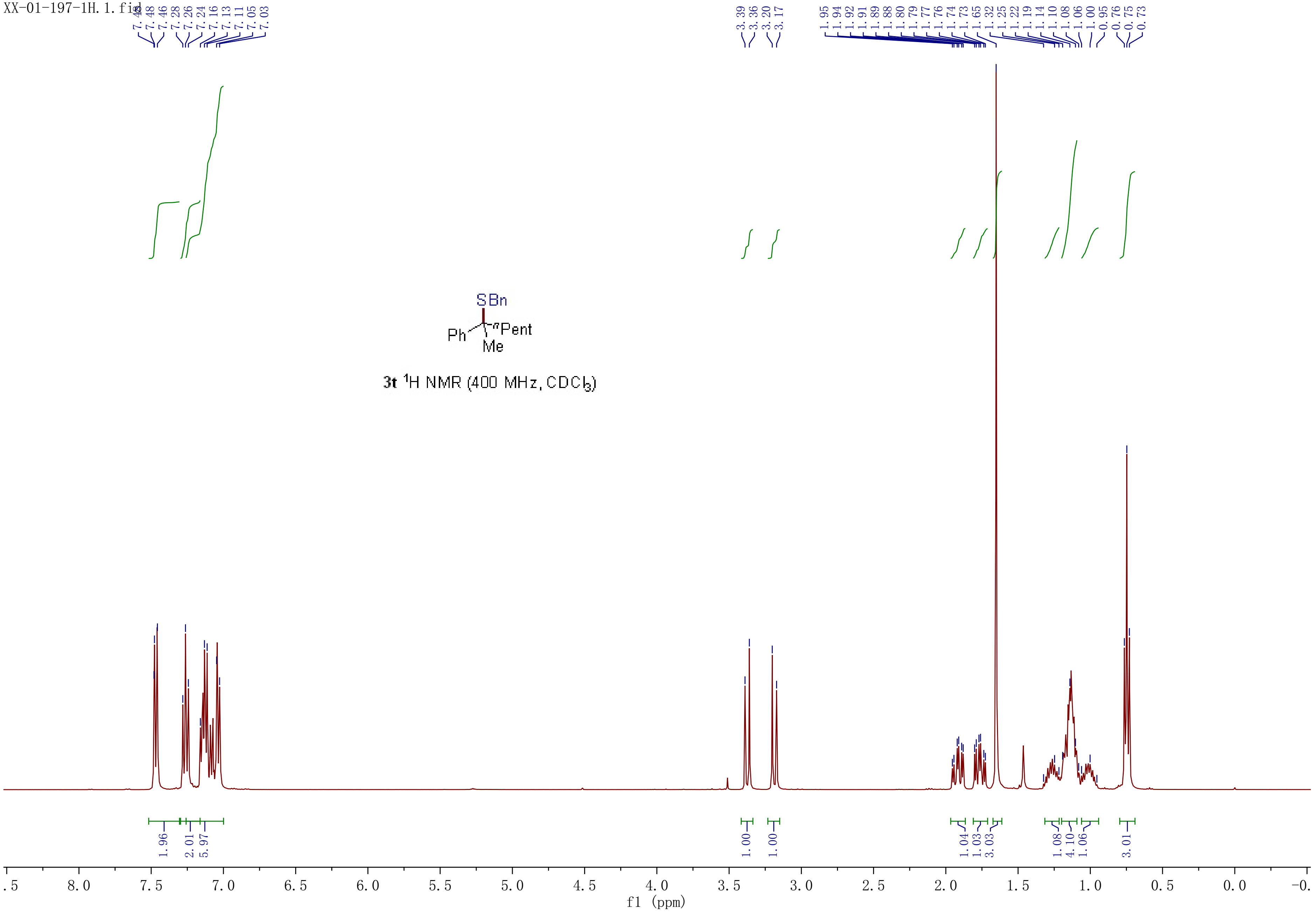

Supplementary Figure 139. ^{1}H NMR (400 MHz, $CDCl_3$) spectra for compound **3t**

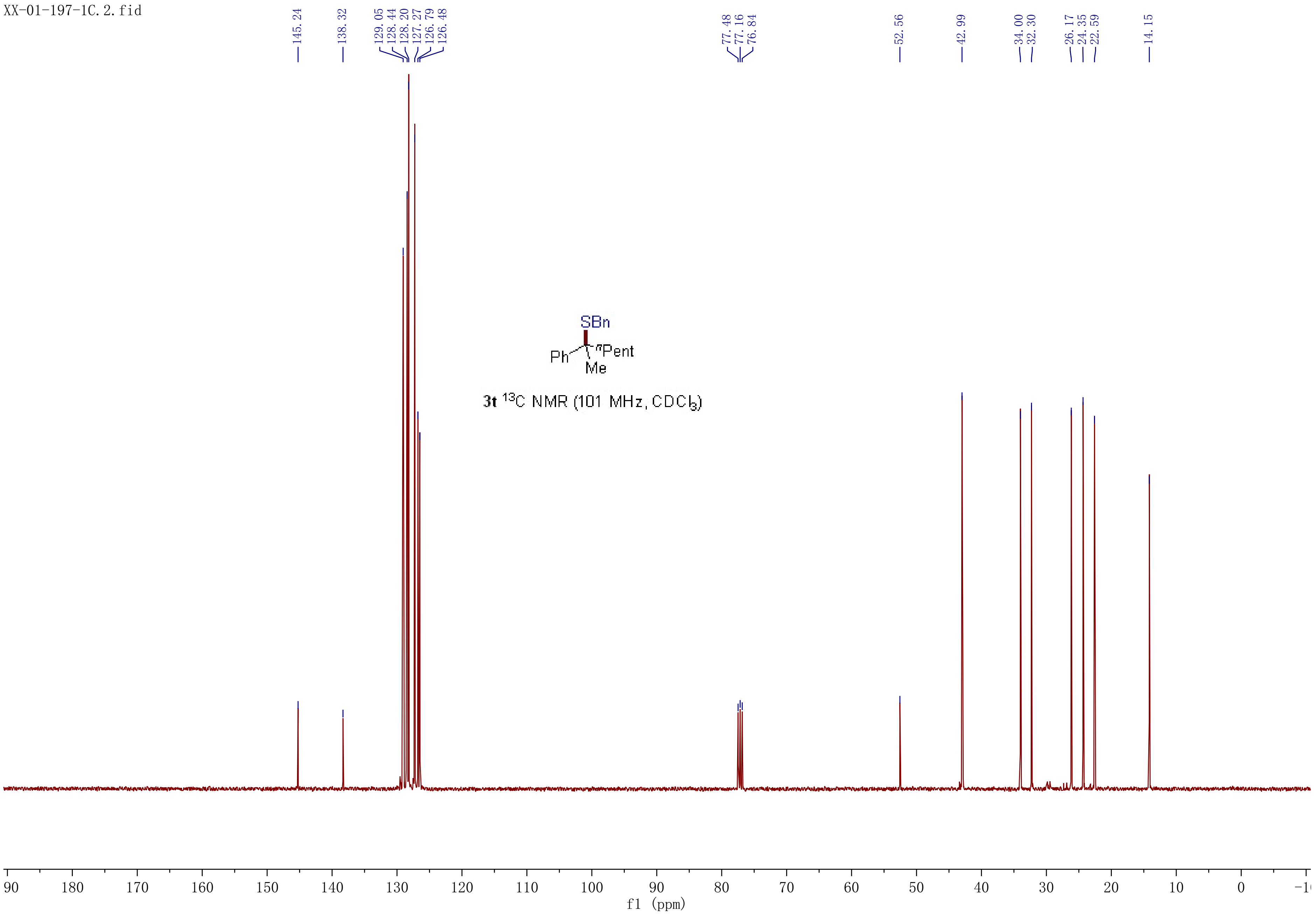

Supplementary Figure 140. ^{13}C NMR (101 MHz, $CDCl_3$) spectra for compound **3t**

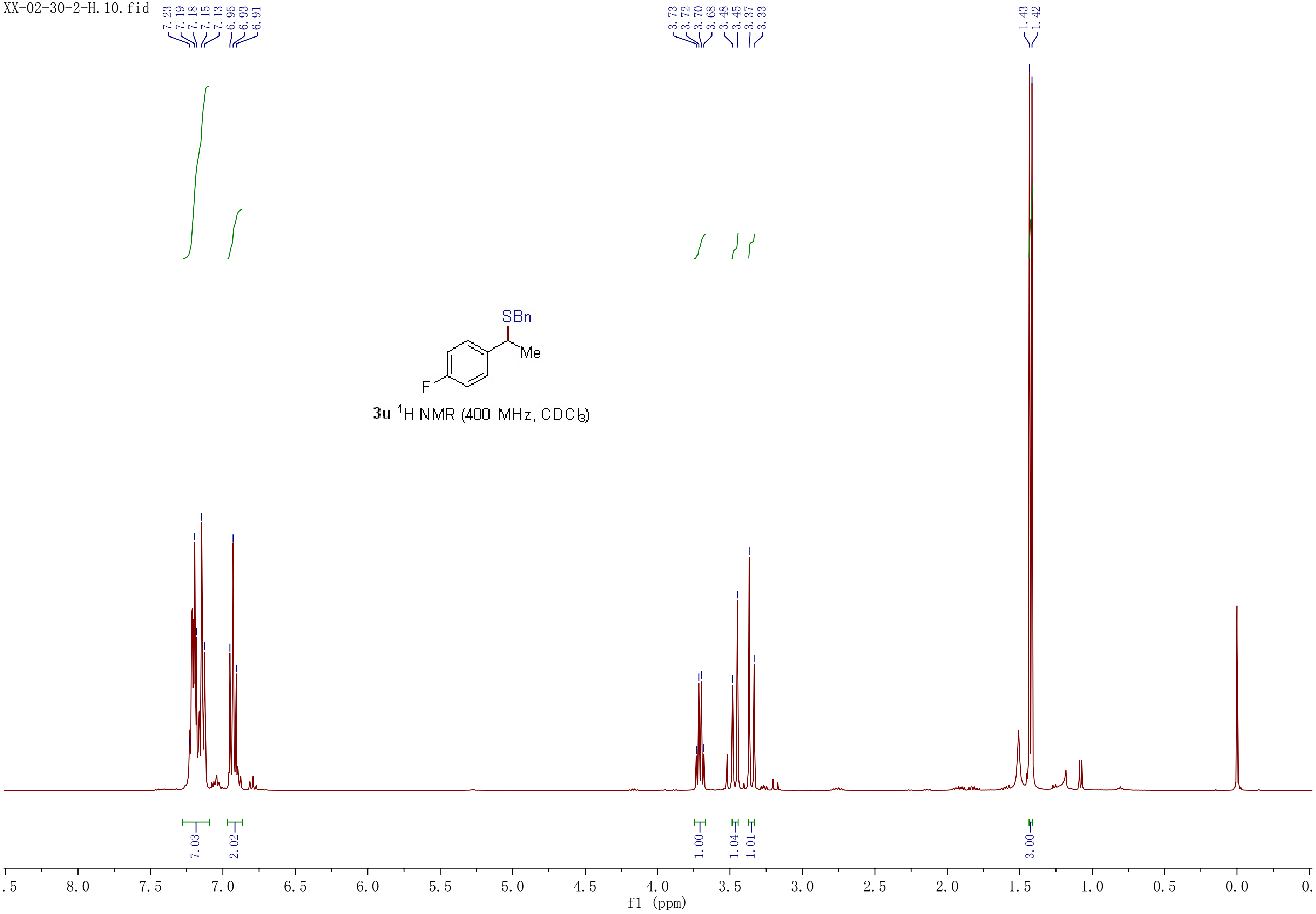

Supplementary Figure 141. ^{1}H NMR (400 MHz, $CDCl_3$) spectra for compound **3u**

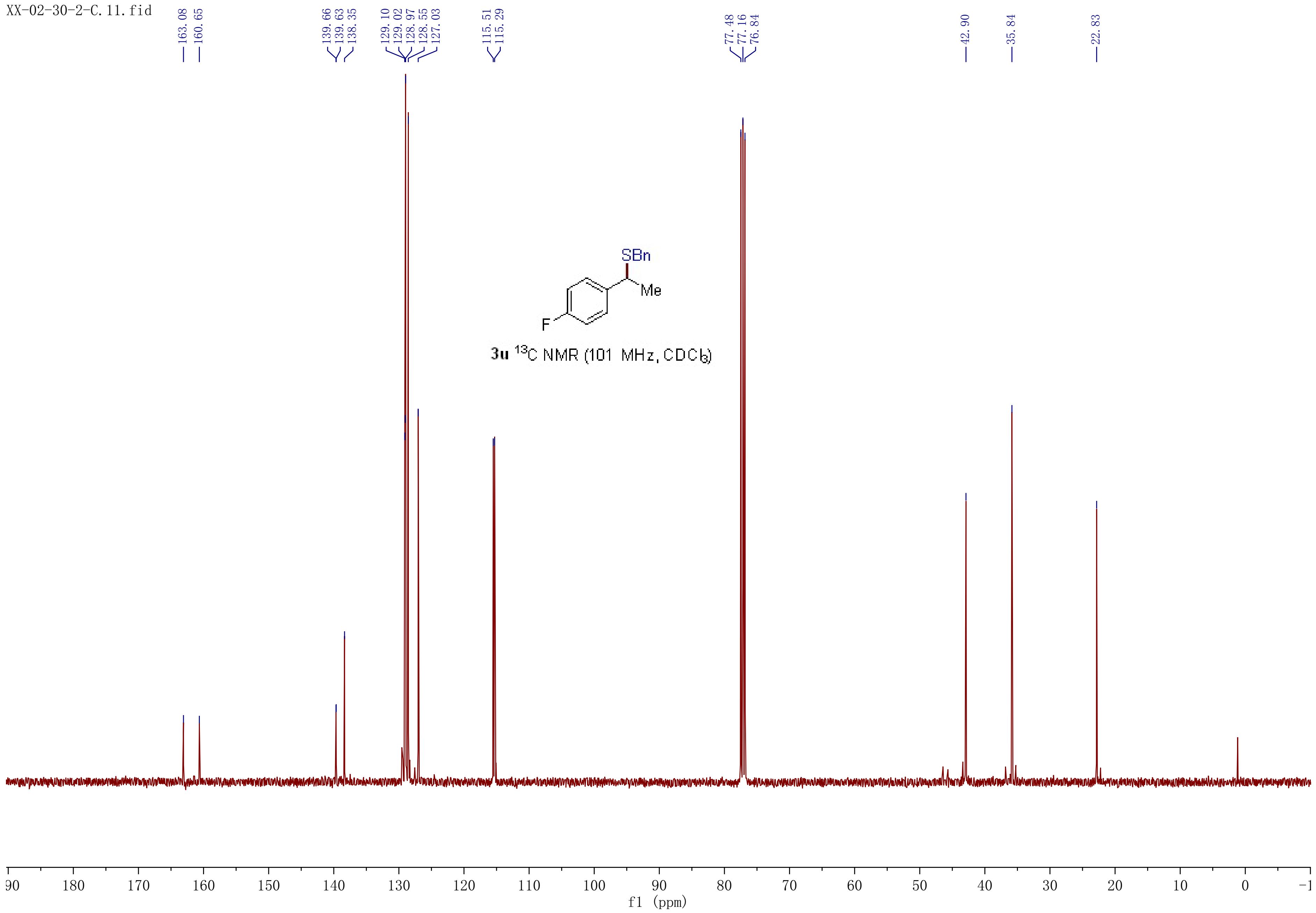

Supplementary Figure 142. ^{13}C NMR (101 MHz, $CDCl_3$) spectra for compound **3u**

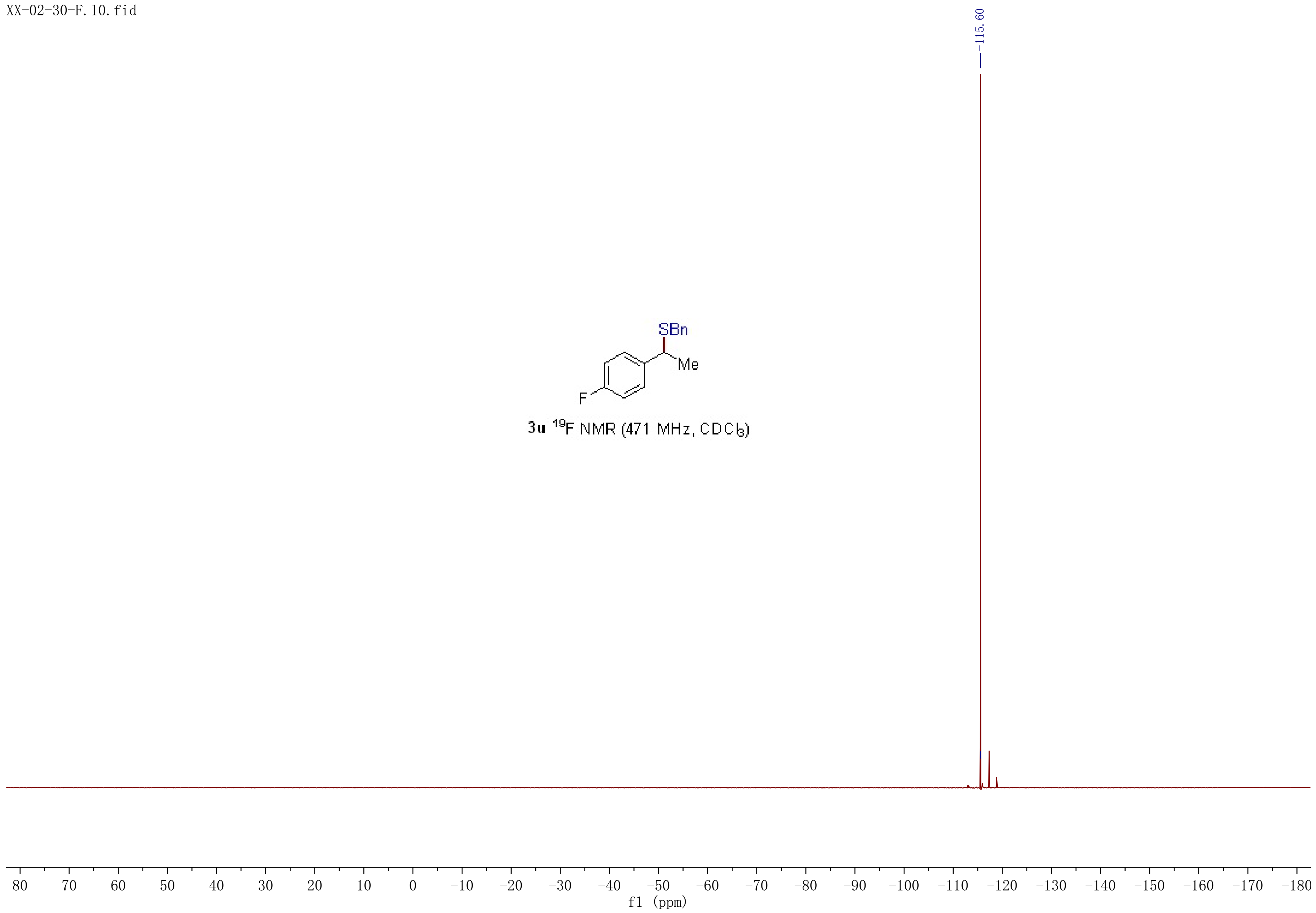

Supplementary Figure 143. ^{19}F NMR (471 MHz, CDCl$_3$) spectra for compound **3u**

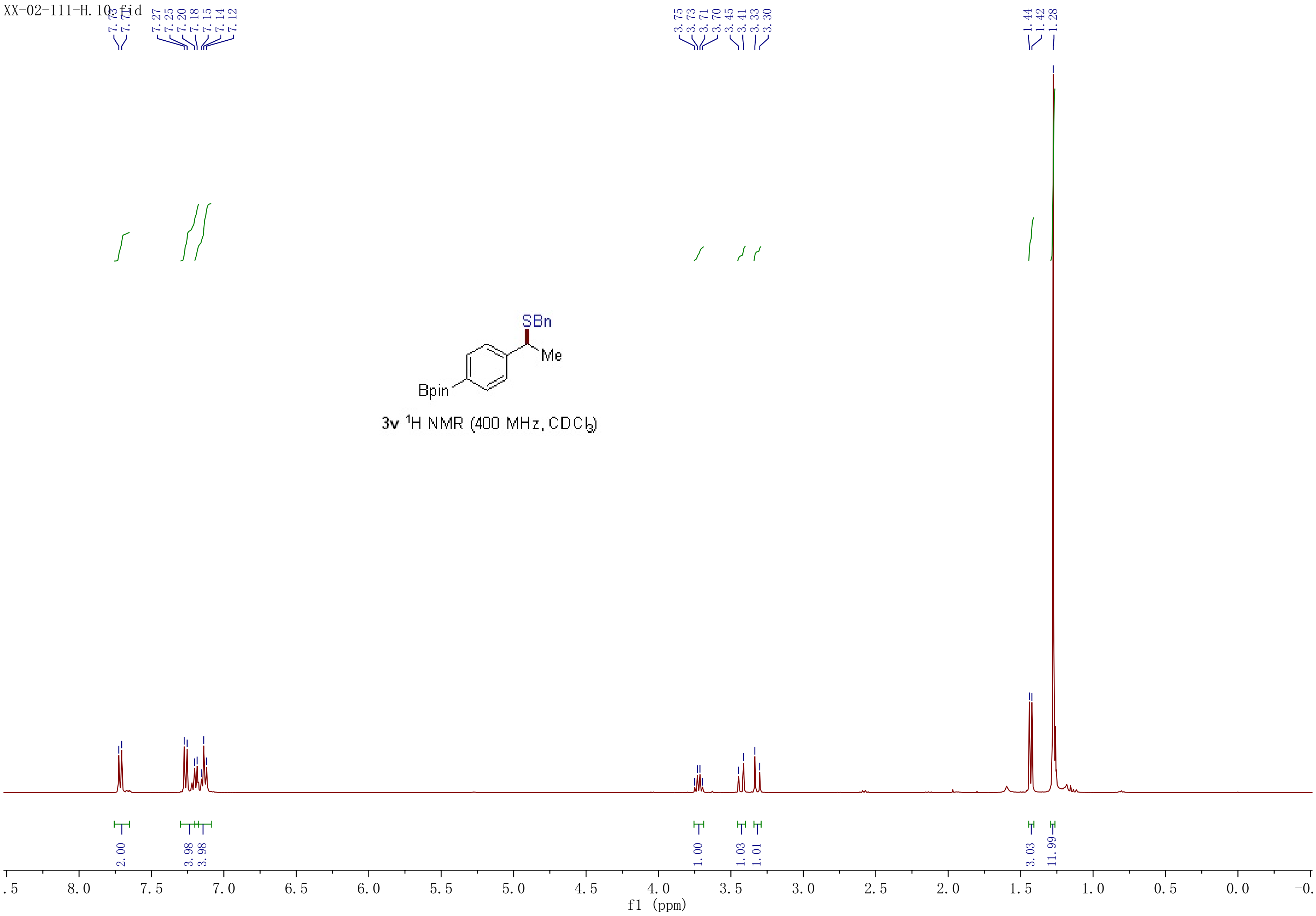

Supplementary Figure 144. ^{1}H NMR (400 MHz, $CDCl_3$) spectra for compound **3v**

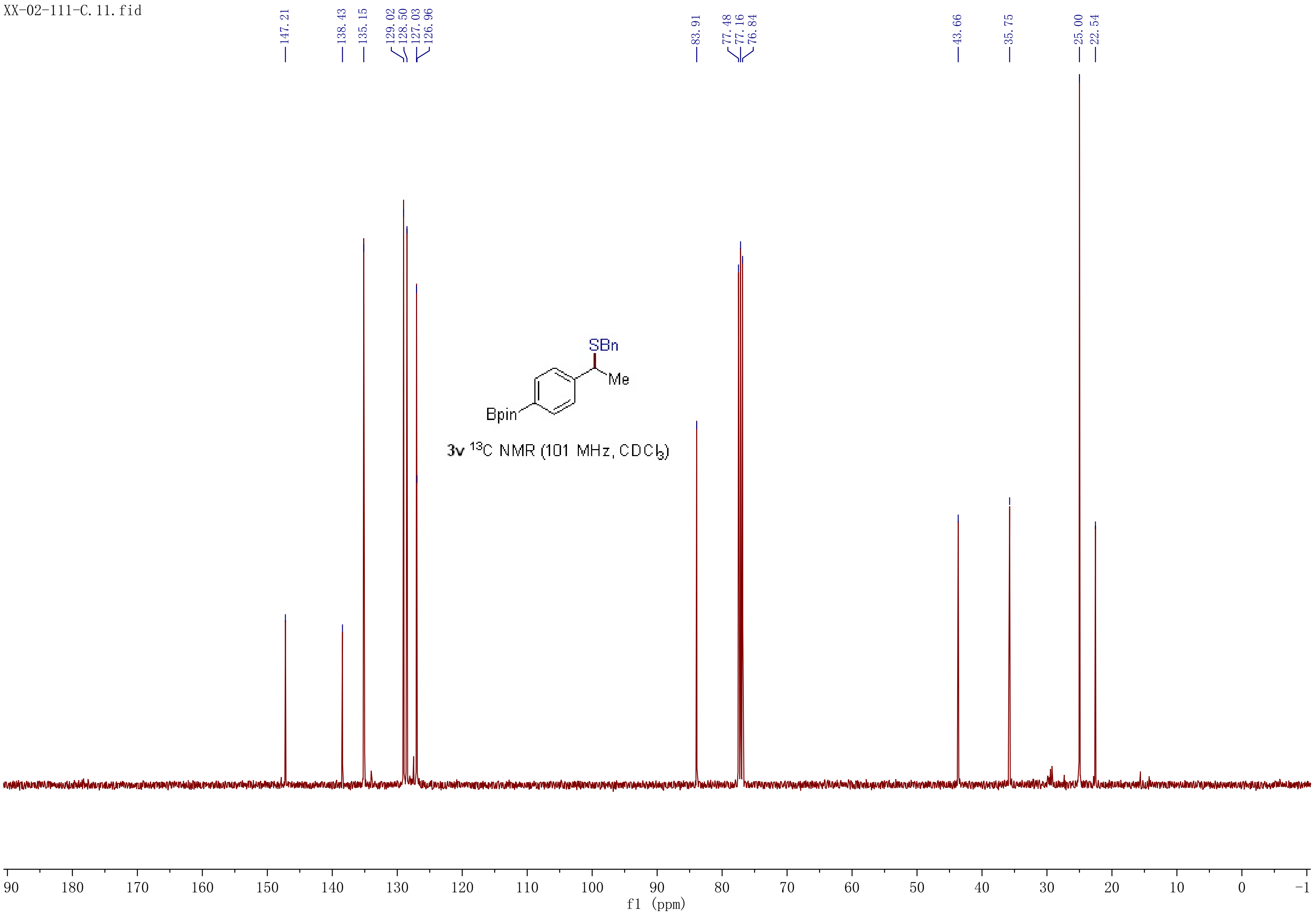

Supplementary Figure 145. ^{13}C NMR (101 MHz, $CDCl_3$) spectra for compound **3v**

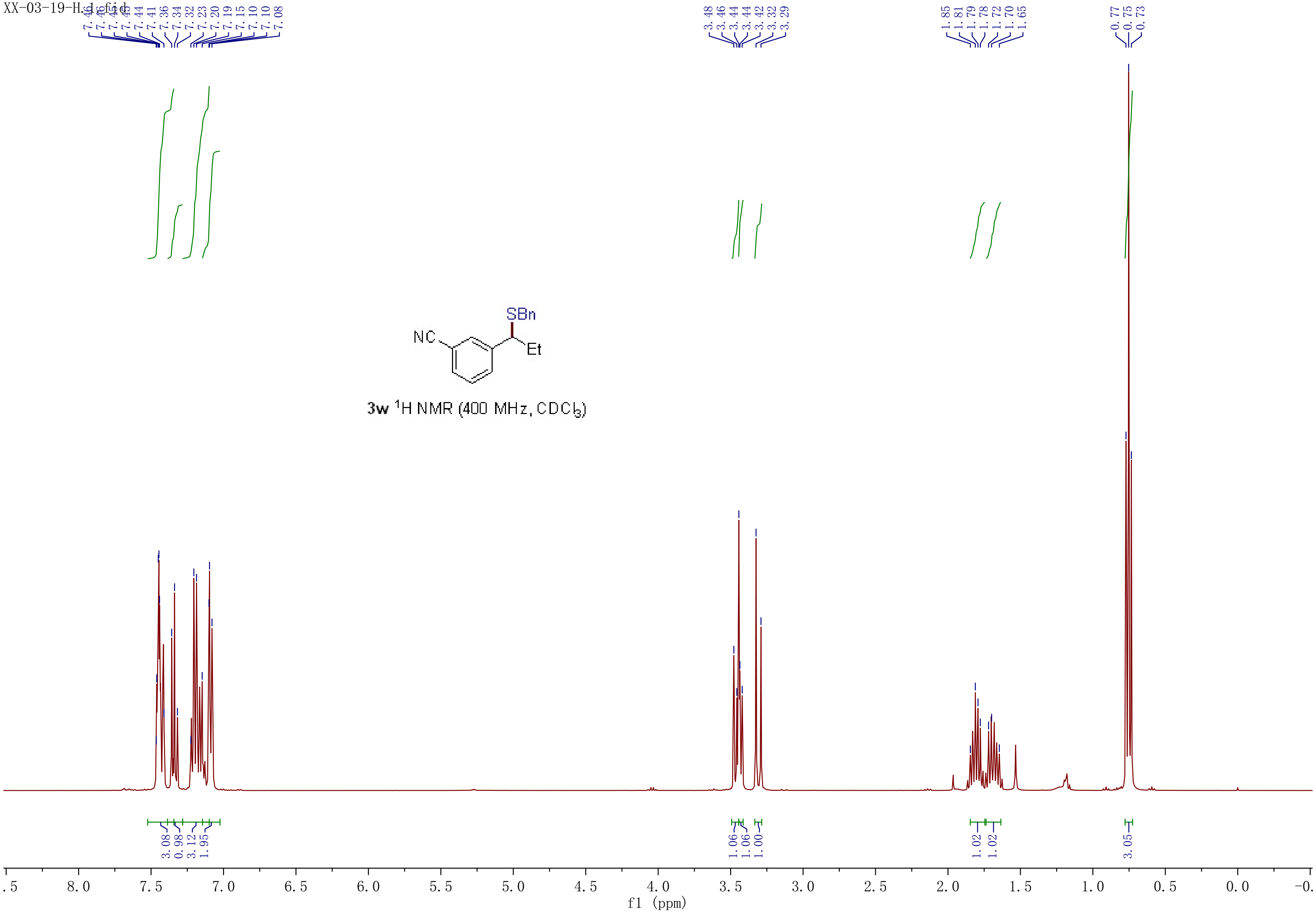

Supplementary Figure 146. ^{1}H NMR (400 MHz, $CDCl_3$) spectra for compound **3w**

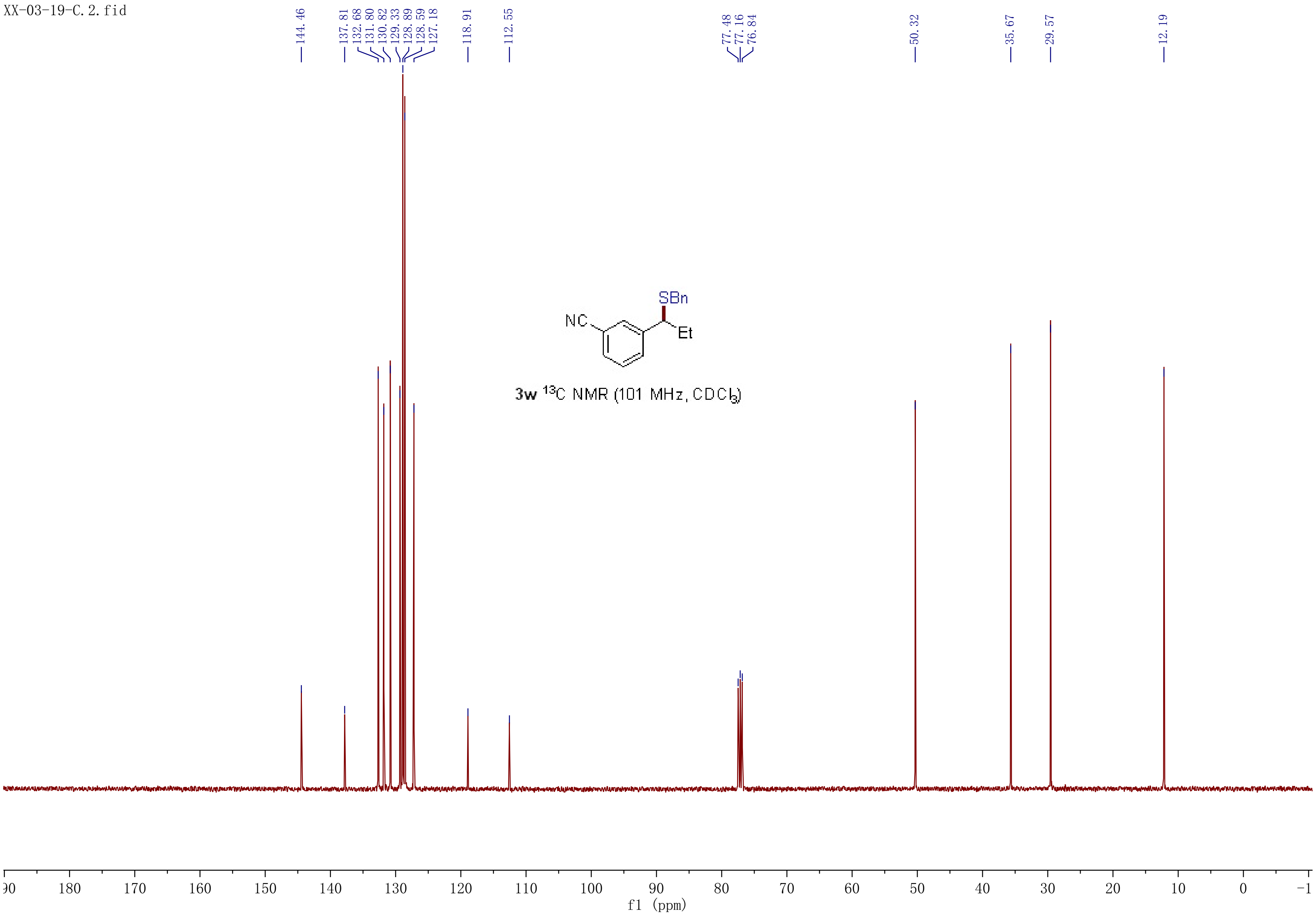

Supplementary Figure 147. ^{13}C NMR (101 MHz, $CDCl_3$) spectra for compound **3w**

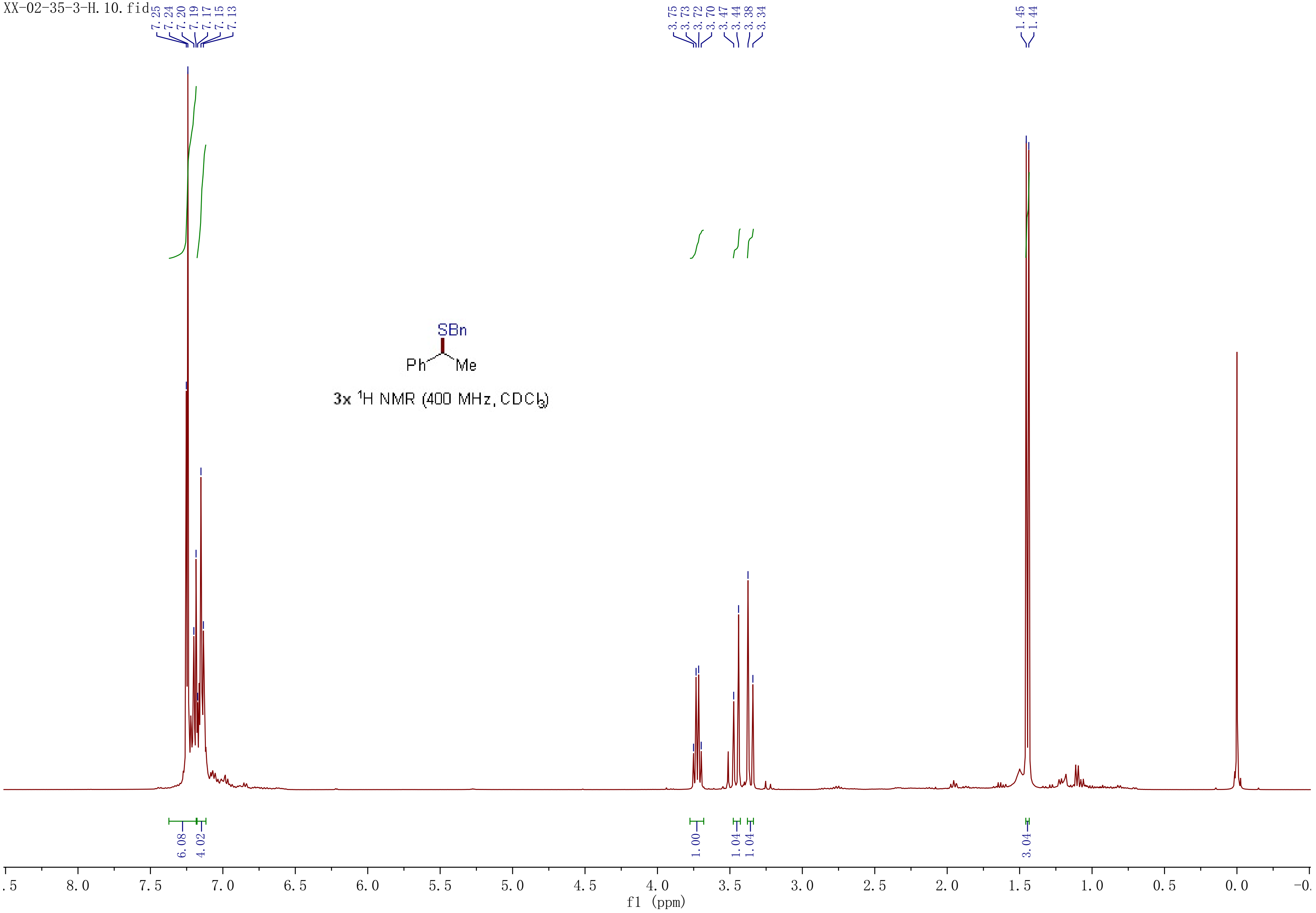

Supplementary Figure 148. ^{1}H NMR (400 MHz, $CDCl_3$) spectra for compound **3x**

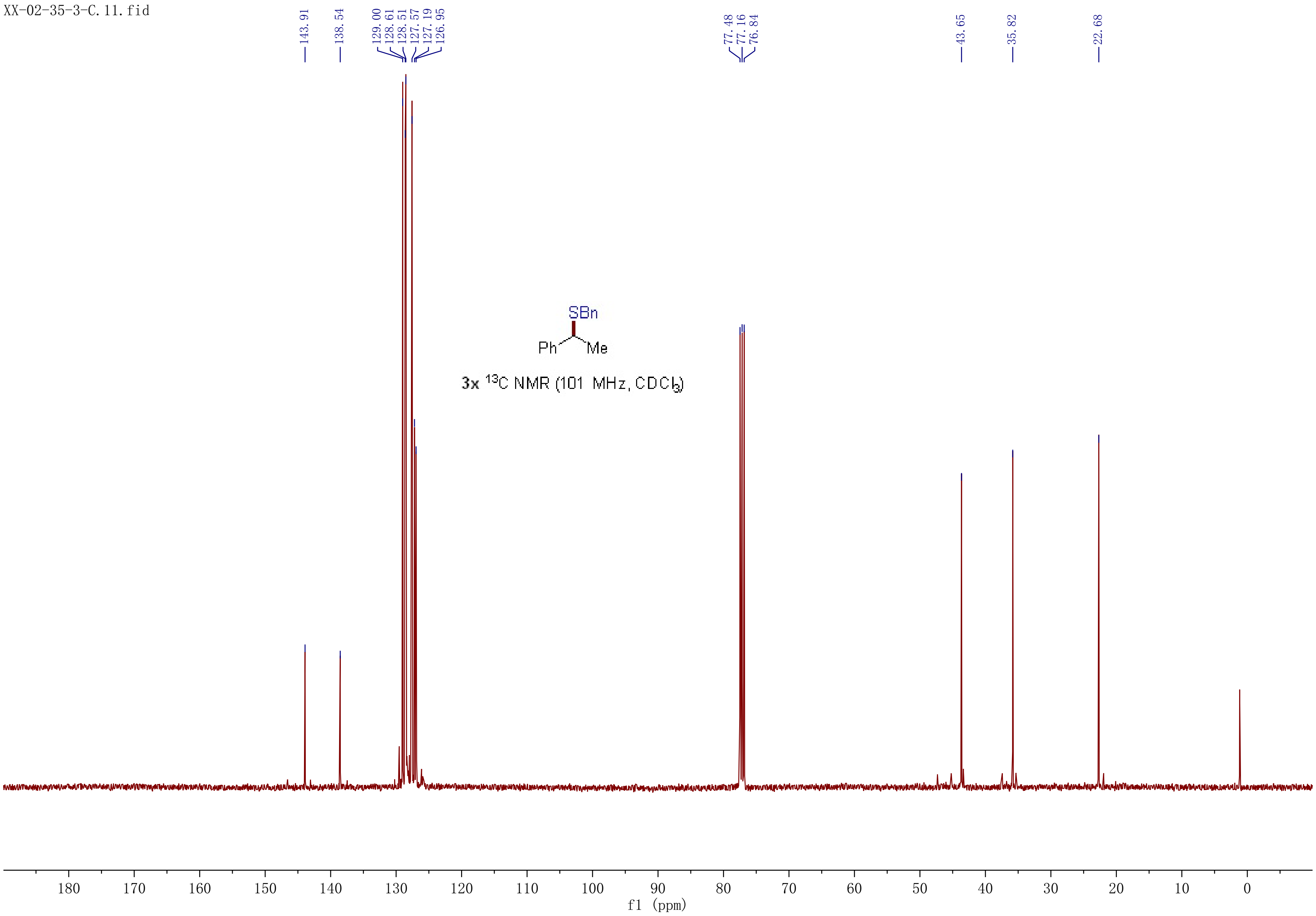

Supplementary Figure 149. ^{13}C NMR (101 MHz, $CDCl_3$) spectra for compound **3x**

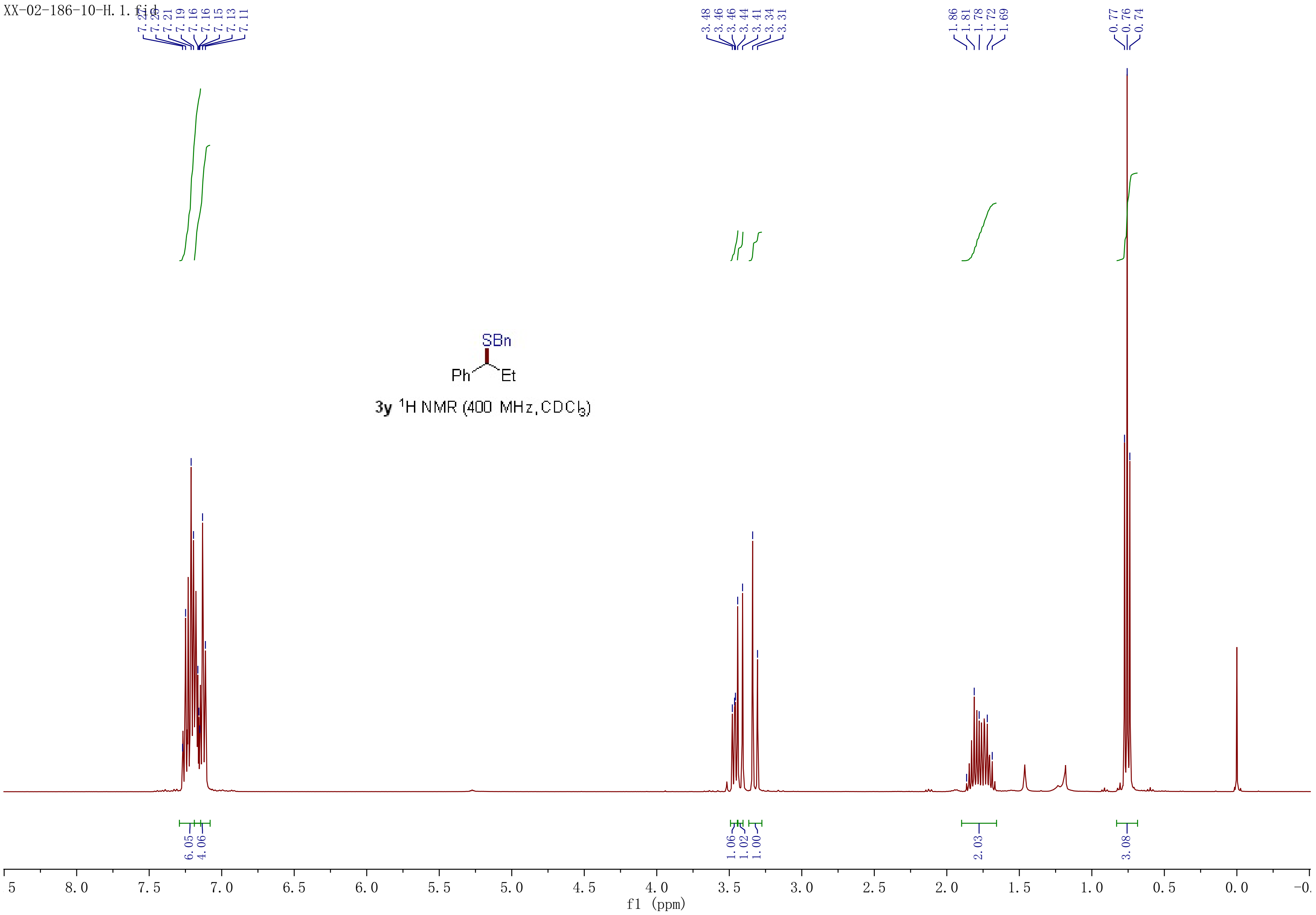

Supplementary Figure 150. ^{1}H NMR (400 MHz, $CDCl_3$) spectra for compound **3y**

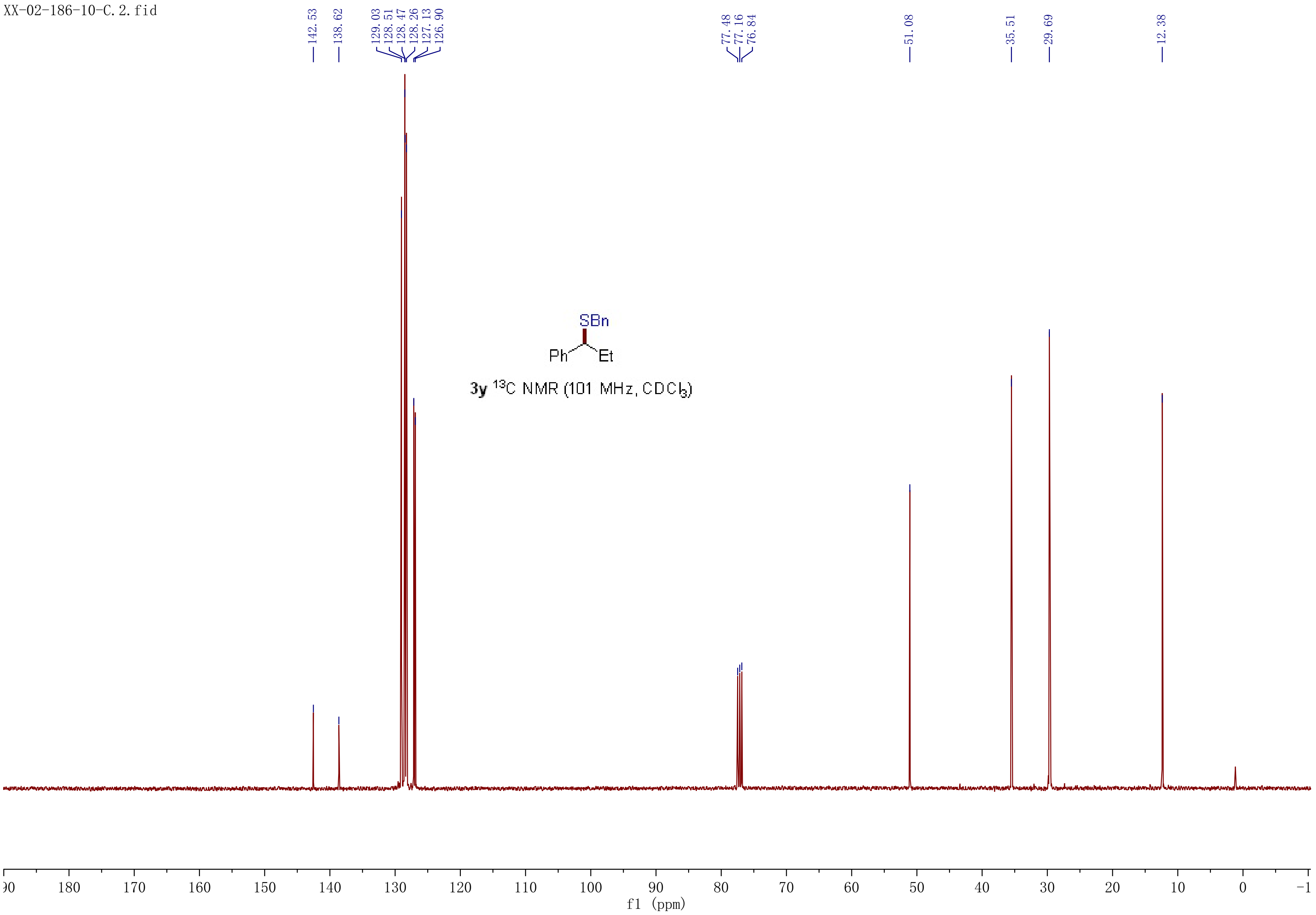

Supplementary Figure 151. ^{13}C NMR (101 MHz, $CDCl_3$) spectra for compound **3y**

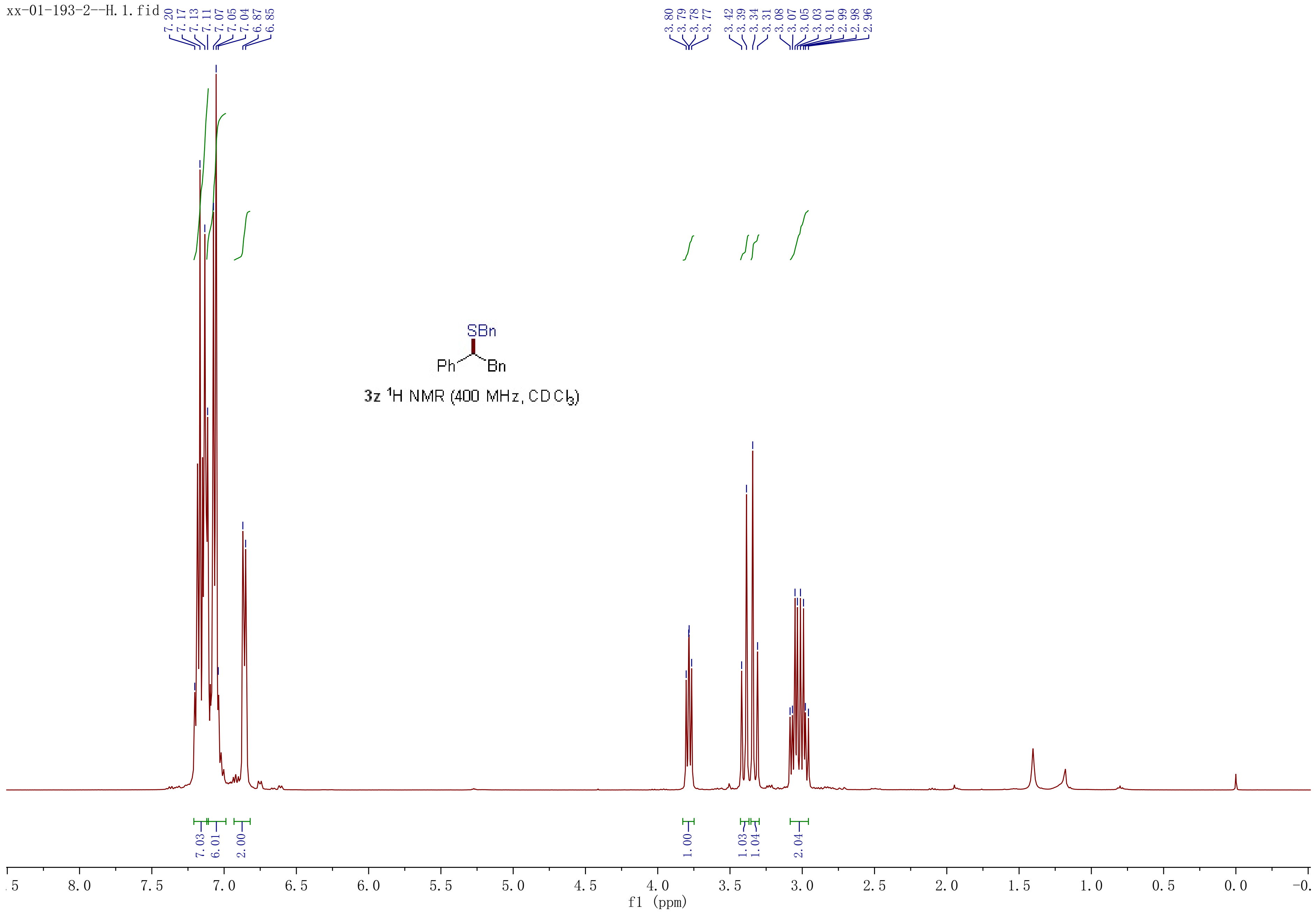

Supplementary Figure 152. ^{1}H NMR (400 MHz, $CDCl_3$) spectra for compound **3z**

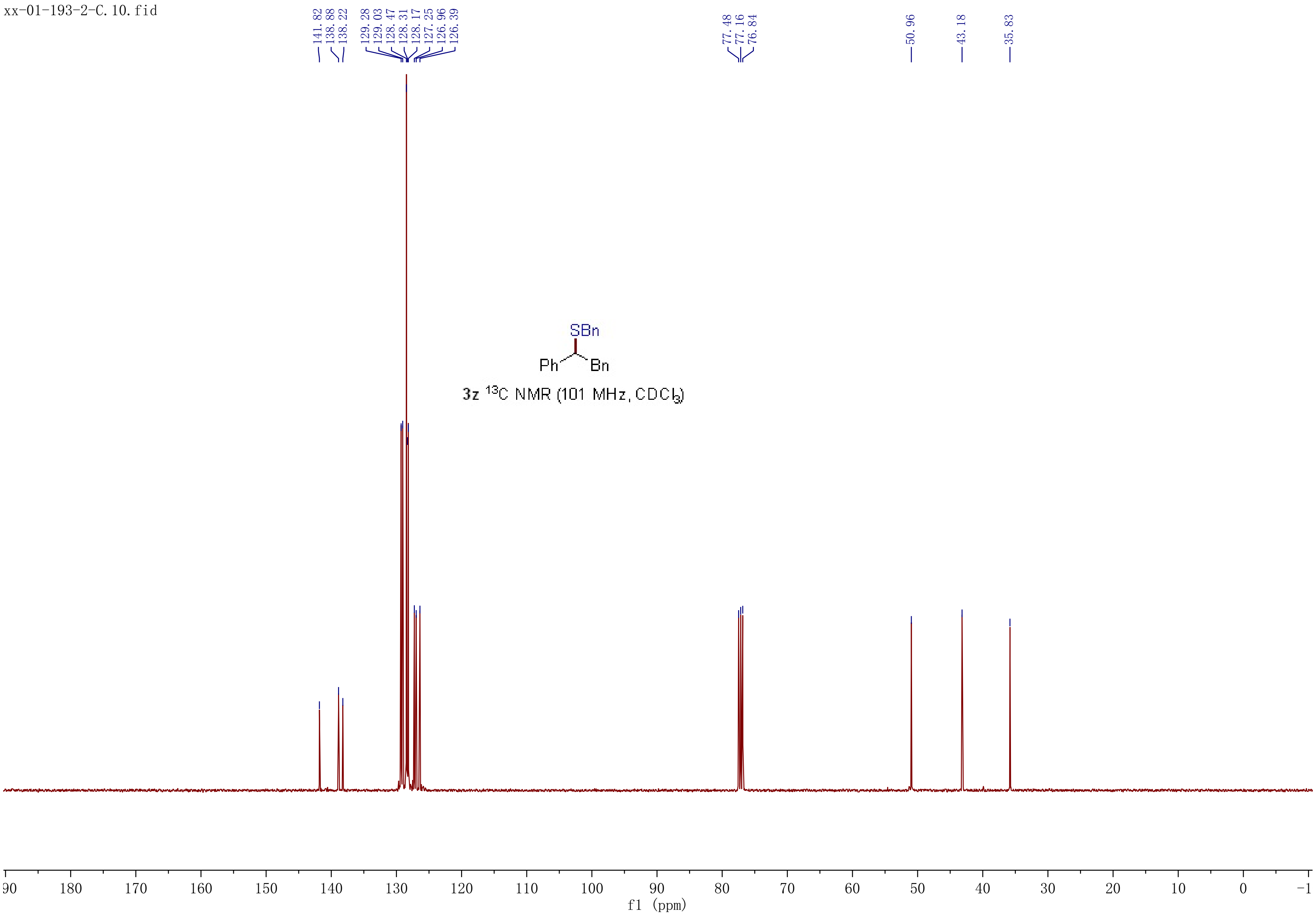

Supplementary Figure 153. ^{13}C NMR (101 MHz, $CDCl_3$) spectra for compound **3z**

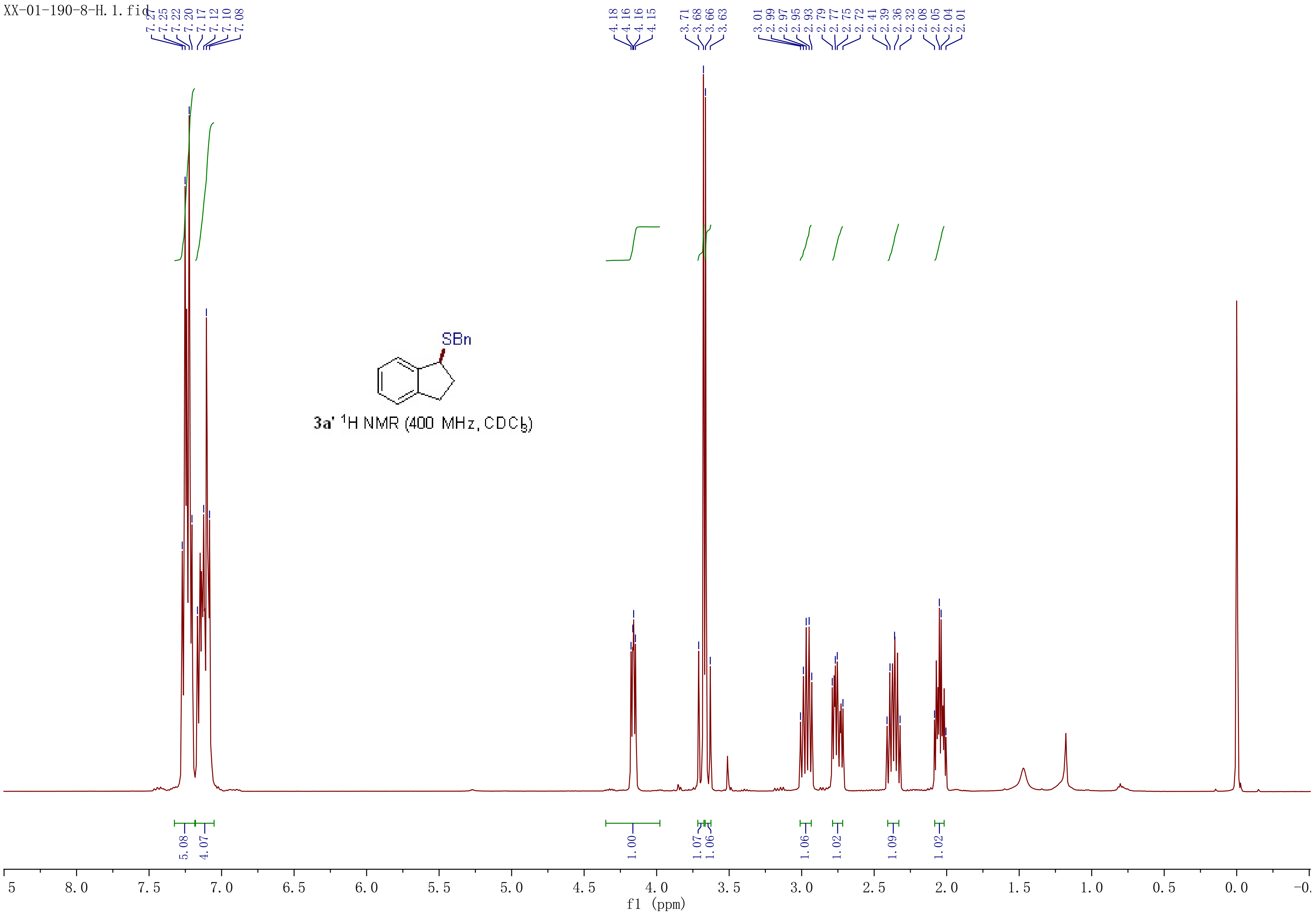

Supplementary Figure 154. ^{1}H NMR (400 MHz, $CDCl_3$) spectra for compound **3a'**

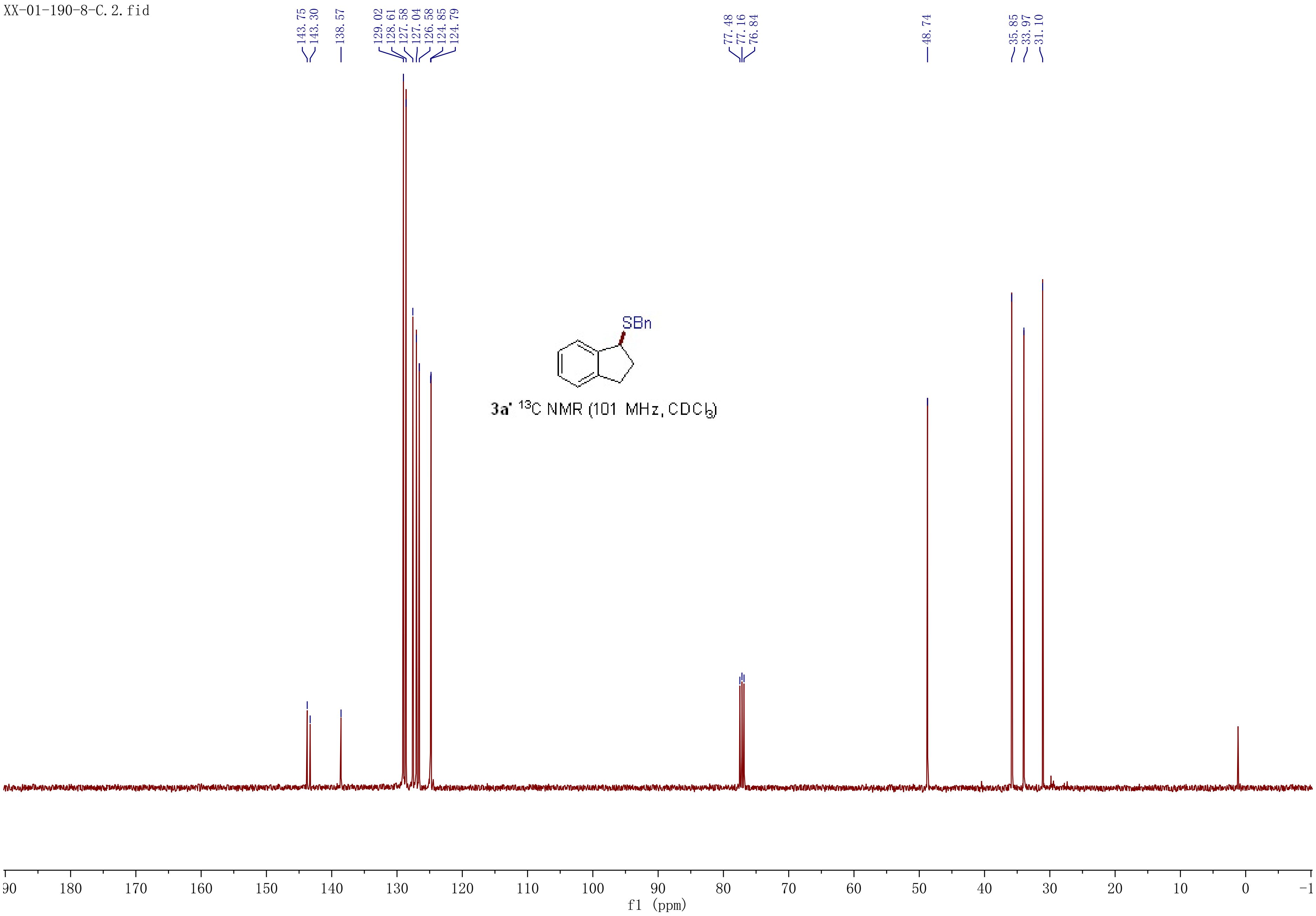

Supplementary Figure 155. ^{13}C NMR (101 MHz, $CDCl_3$) spectra for compound **3a'**

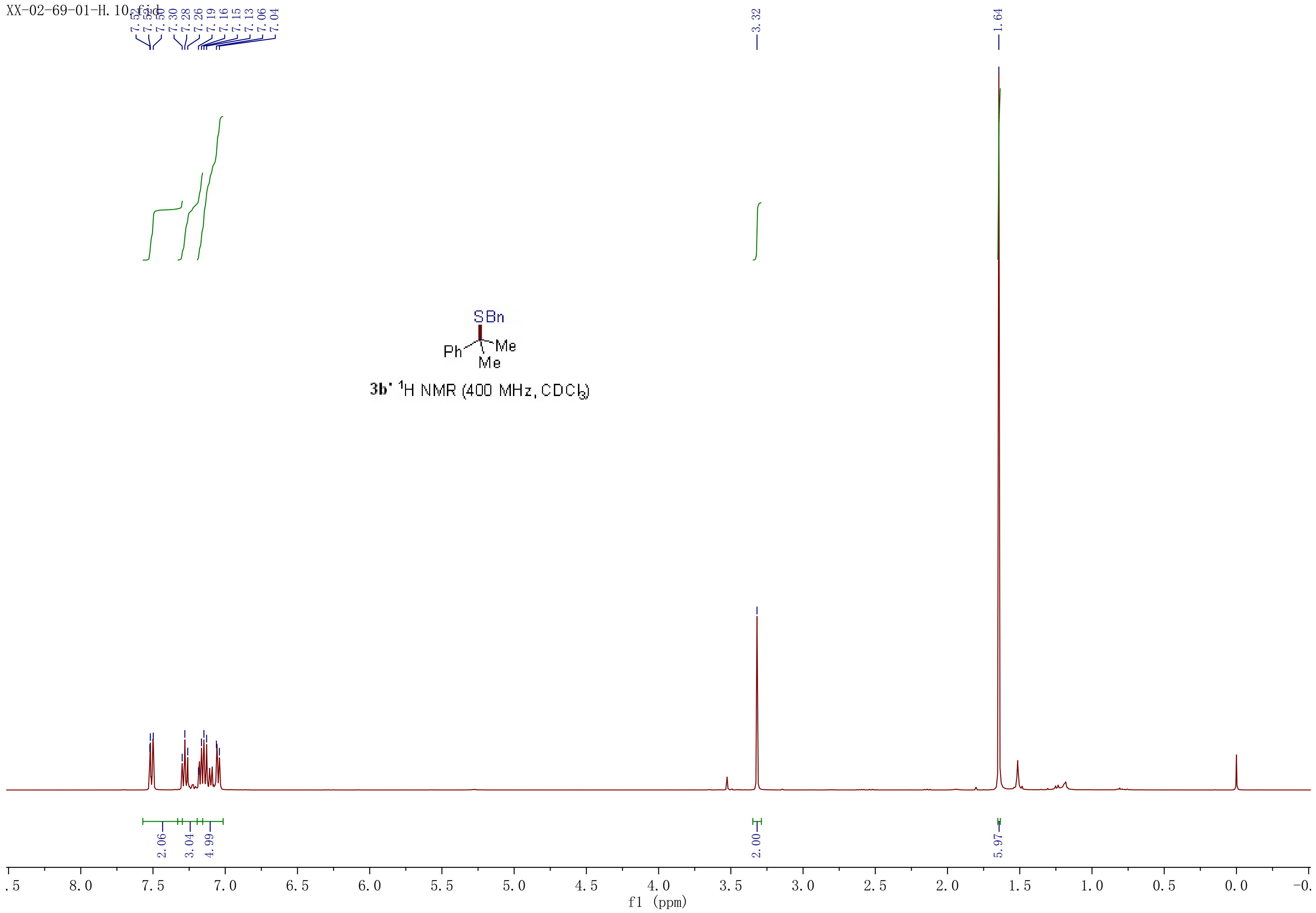

Supplementary Figure 156. ^{1}H NMR (400 MHz, $CDCl_3$) spectra for compound **3b'**

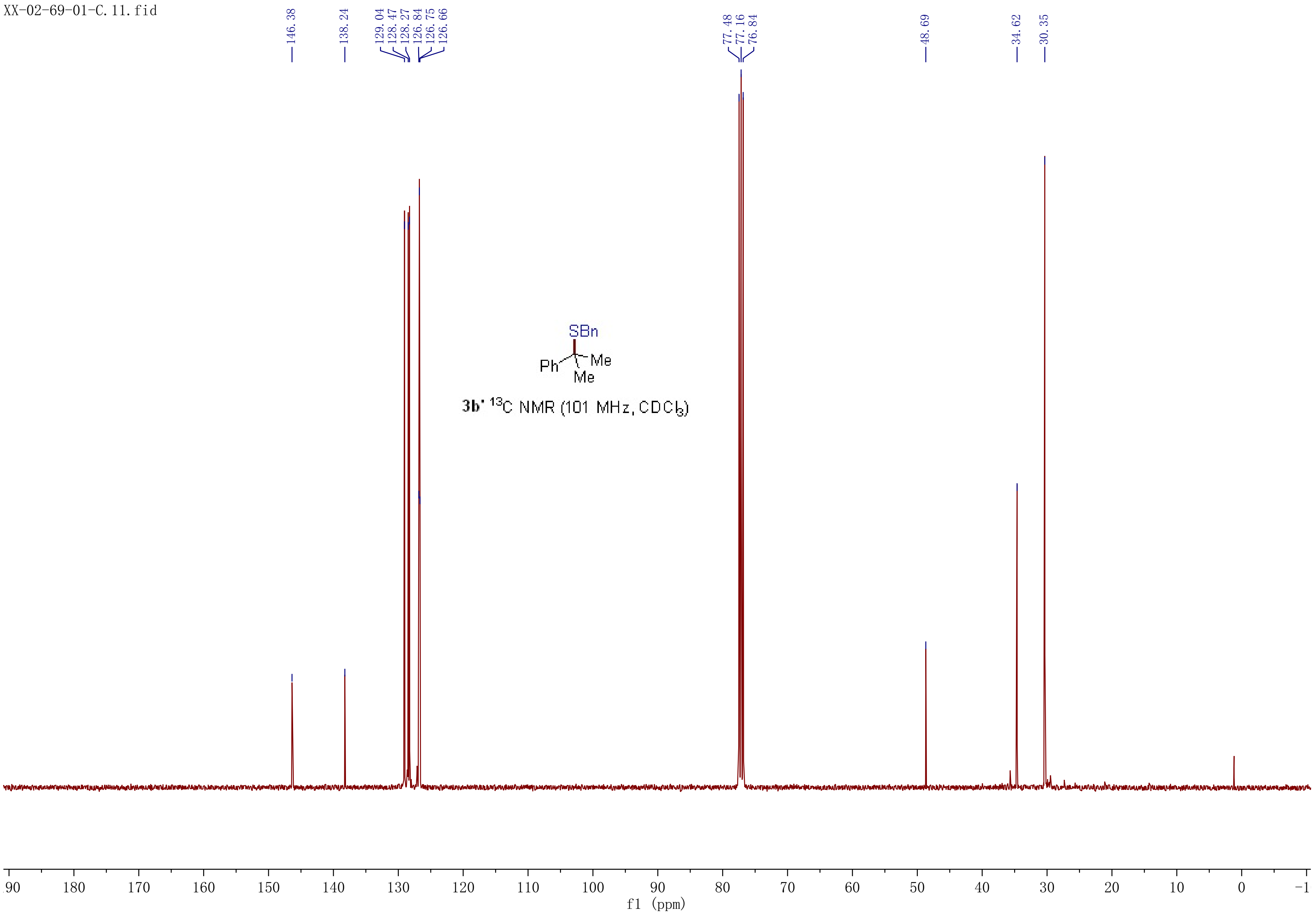

Supplementary Figure 157. ^{13}C NMR (101 MHz, $CDCl_3$) spectra for compound **3b'**

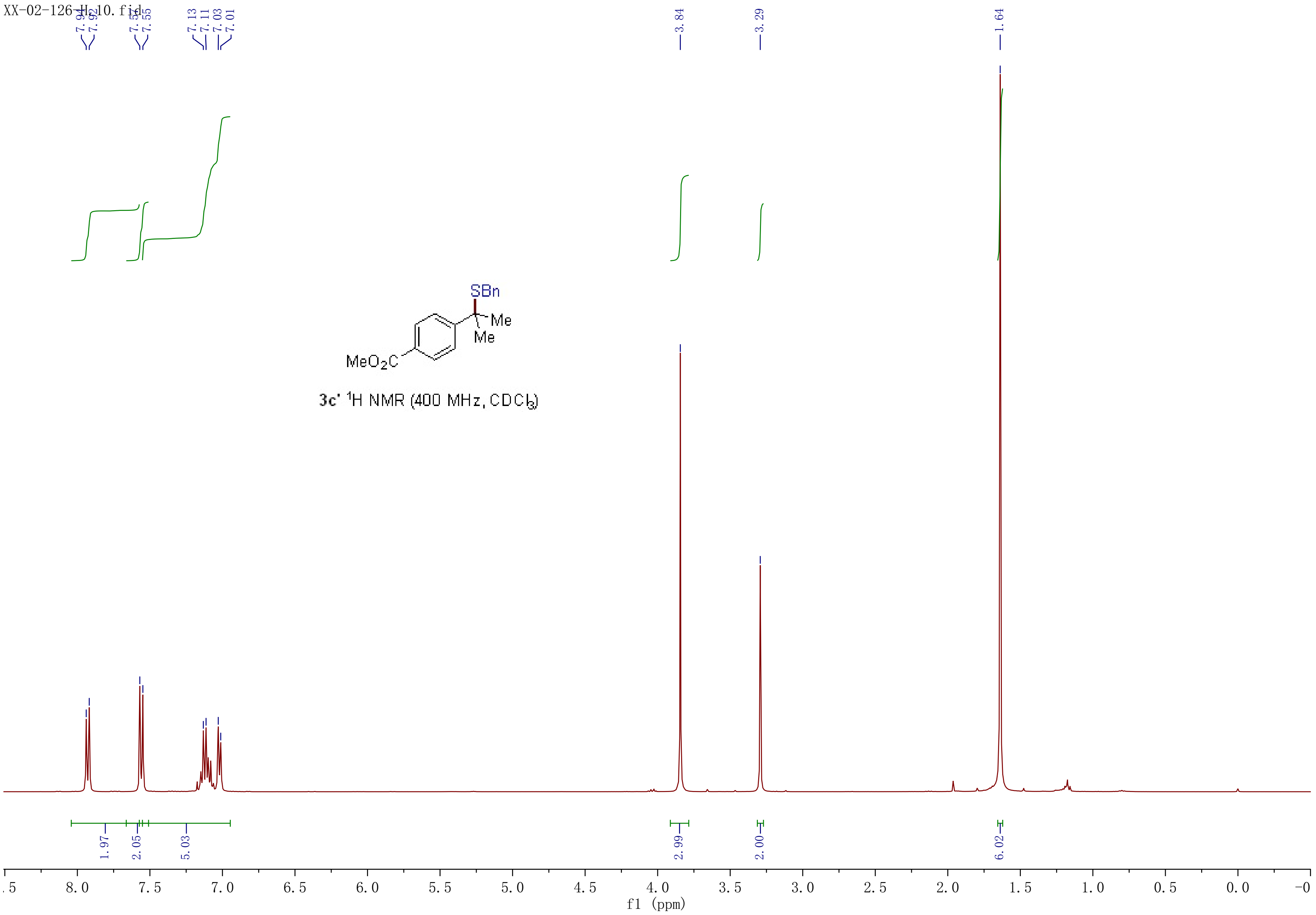

Supplementary Figure 158. ^{1}H NMR (400 MHz, $CDCl_3$) spectra for compound **3c'**

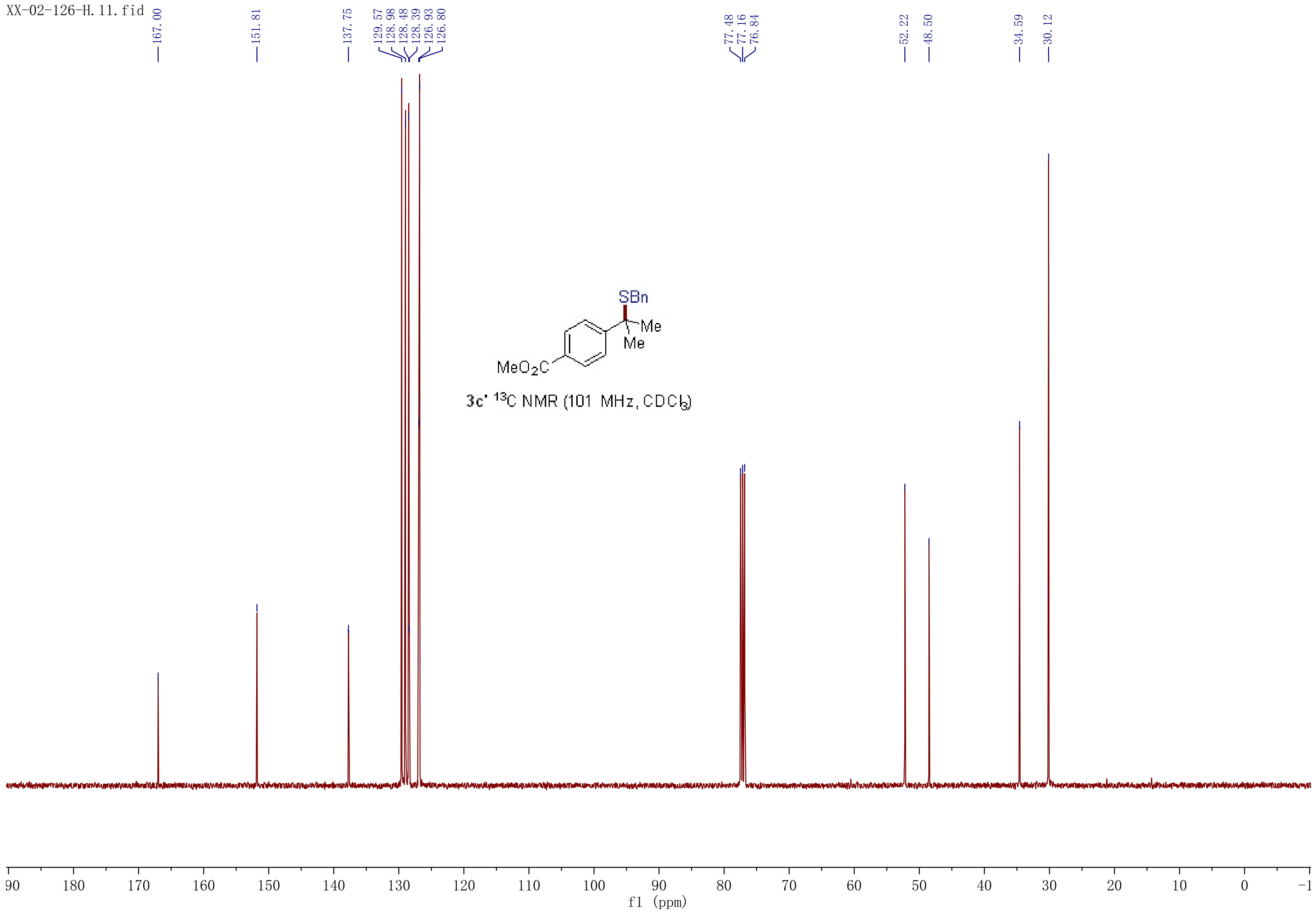

Supplementary Figure 159. ^{13}C NMR (101 MHz, $CDCl_3$) spectra for compound **3c'**

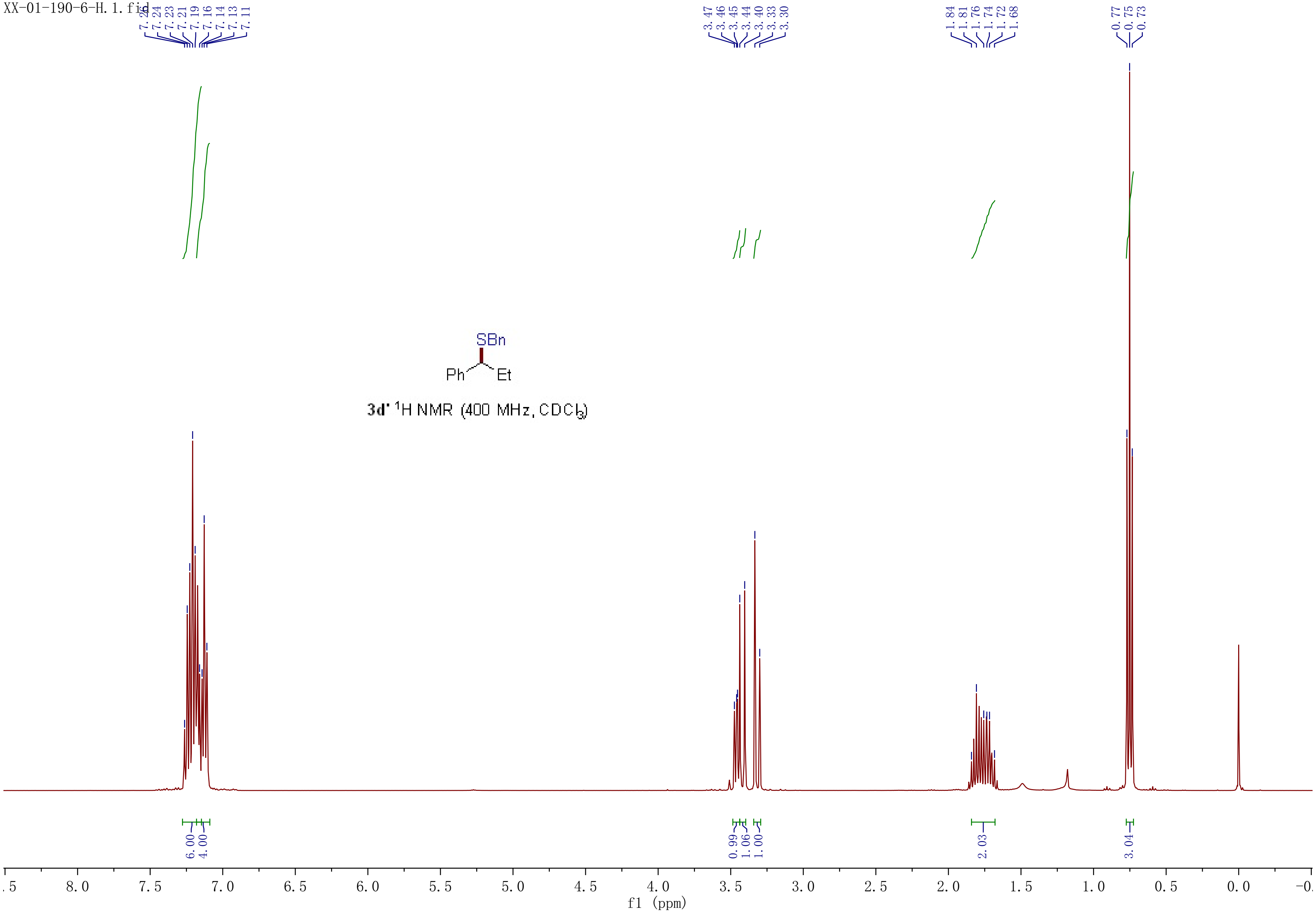

Supplementary Figure 160. ^{1}H NMR (400 MHz, $CDCl_3$) spectra for compound **3d'**

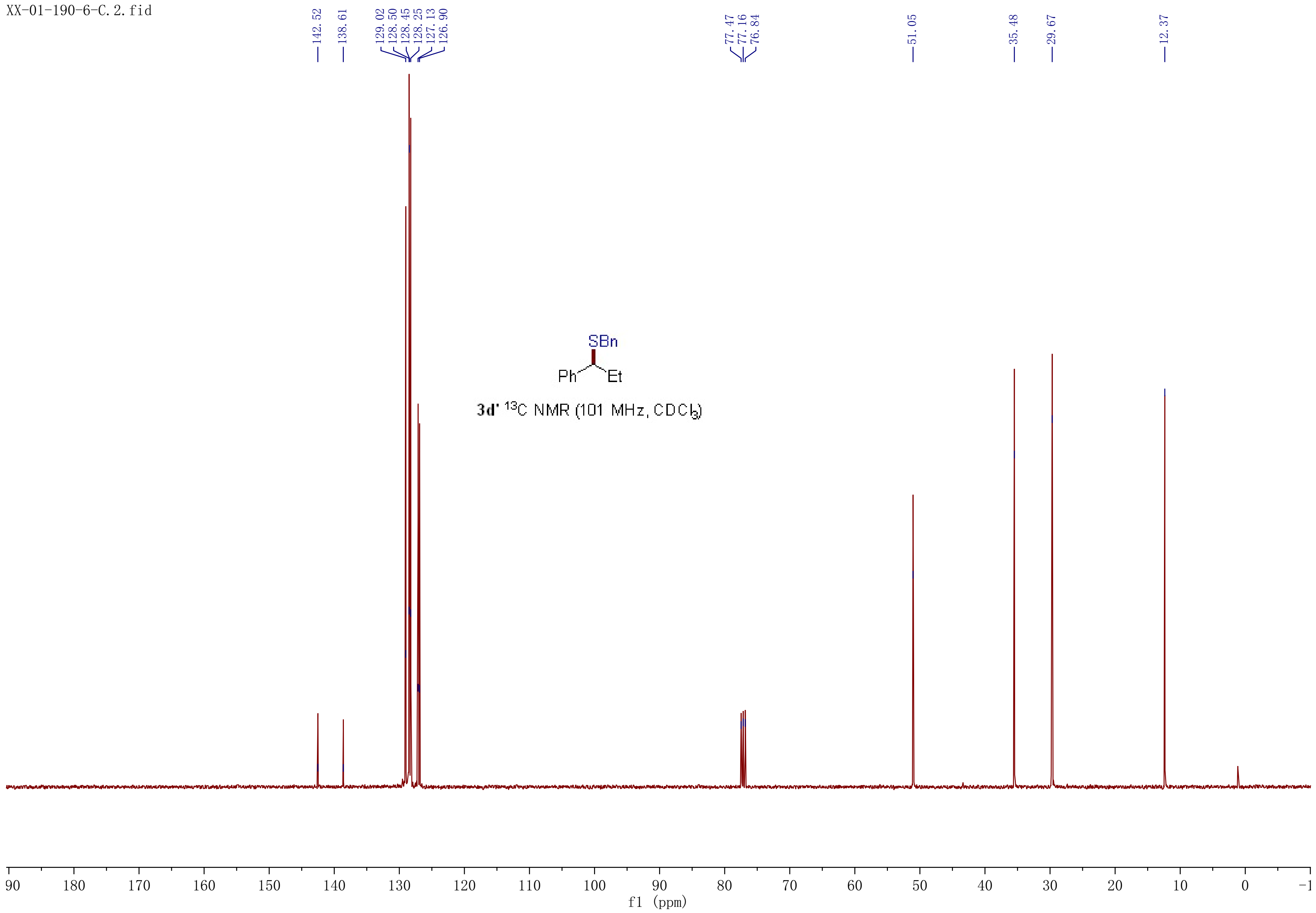

Supplementary Figure 161. ^{13}C NMR (101 MHz, $CDCl_3$) spectra for compound **3d'**

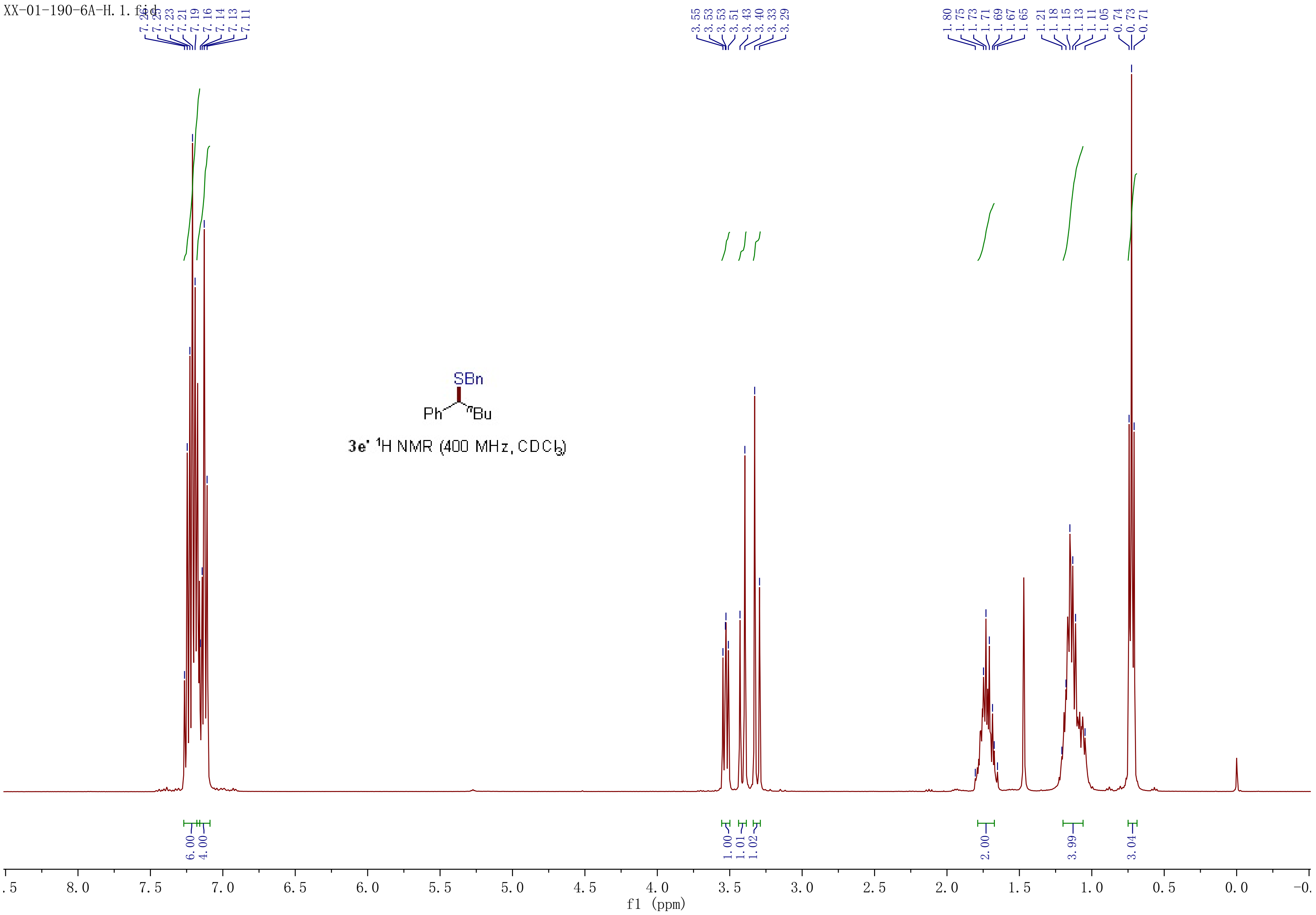

Supplementary Figure 162. ^{1}H NMR (400 MHz, $CDCl_3$) spectra for compound **3e'**

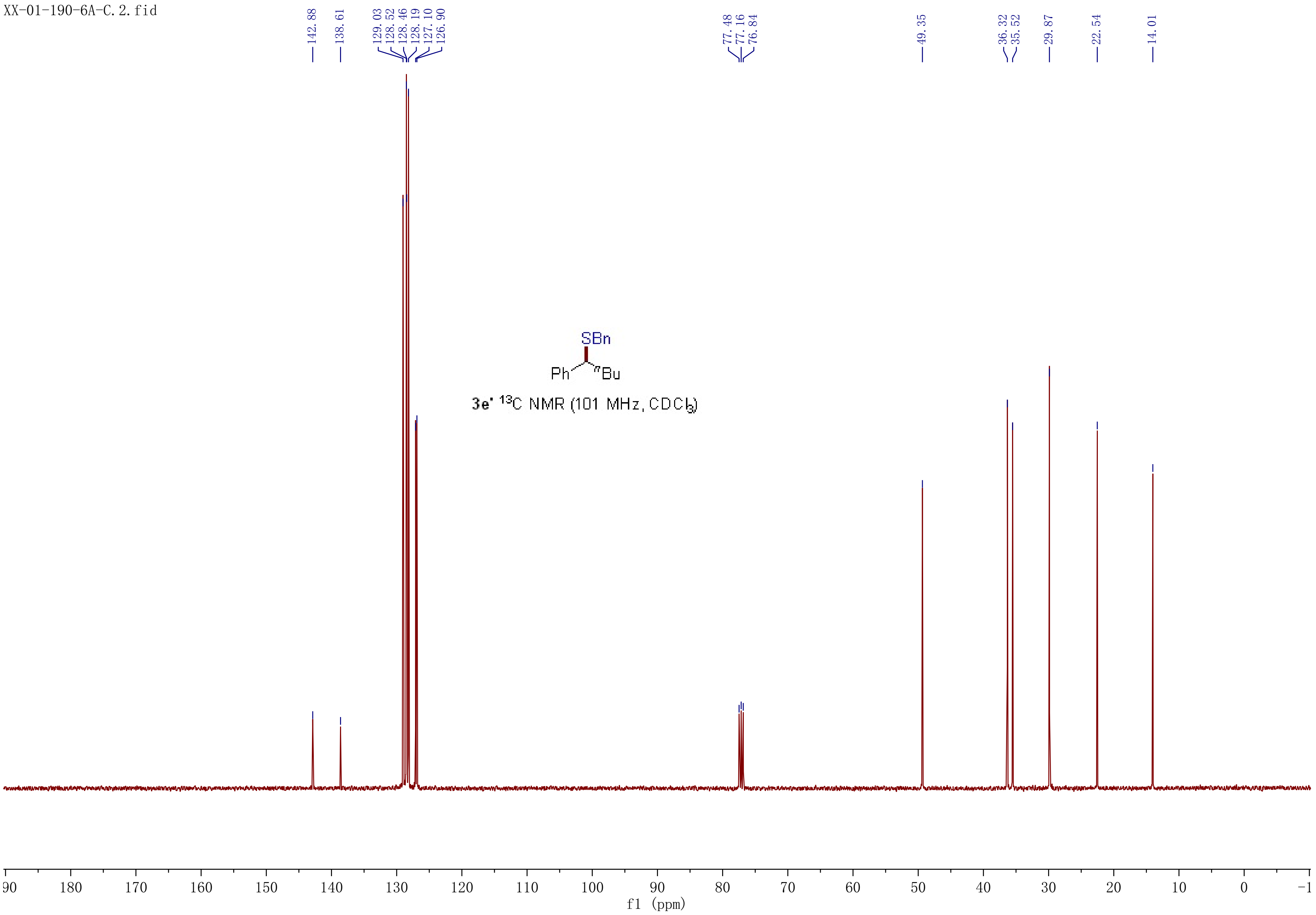

Supplementary Figure 163. ^{13}C NMR (101 MHz, $CDCl_3$) spectra for compound **3e'**

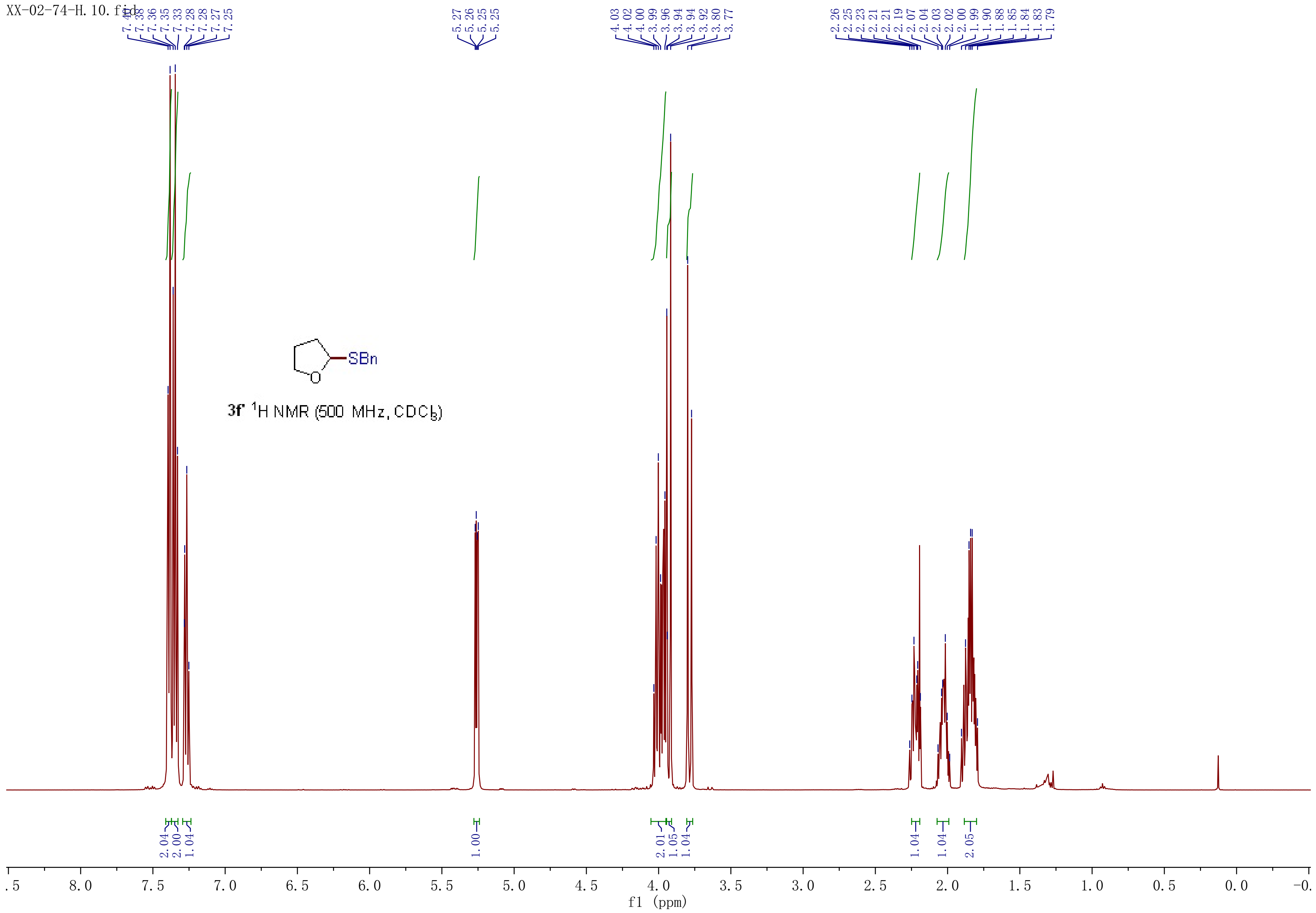

Supplementary Figure 164. ^{1}H NMR (500 MHz, $CDCl_3$) spectra for compound **3f'**

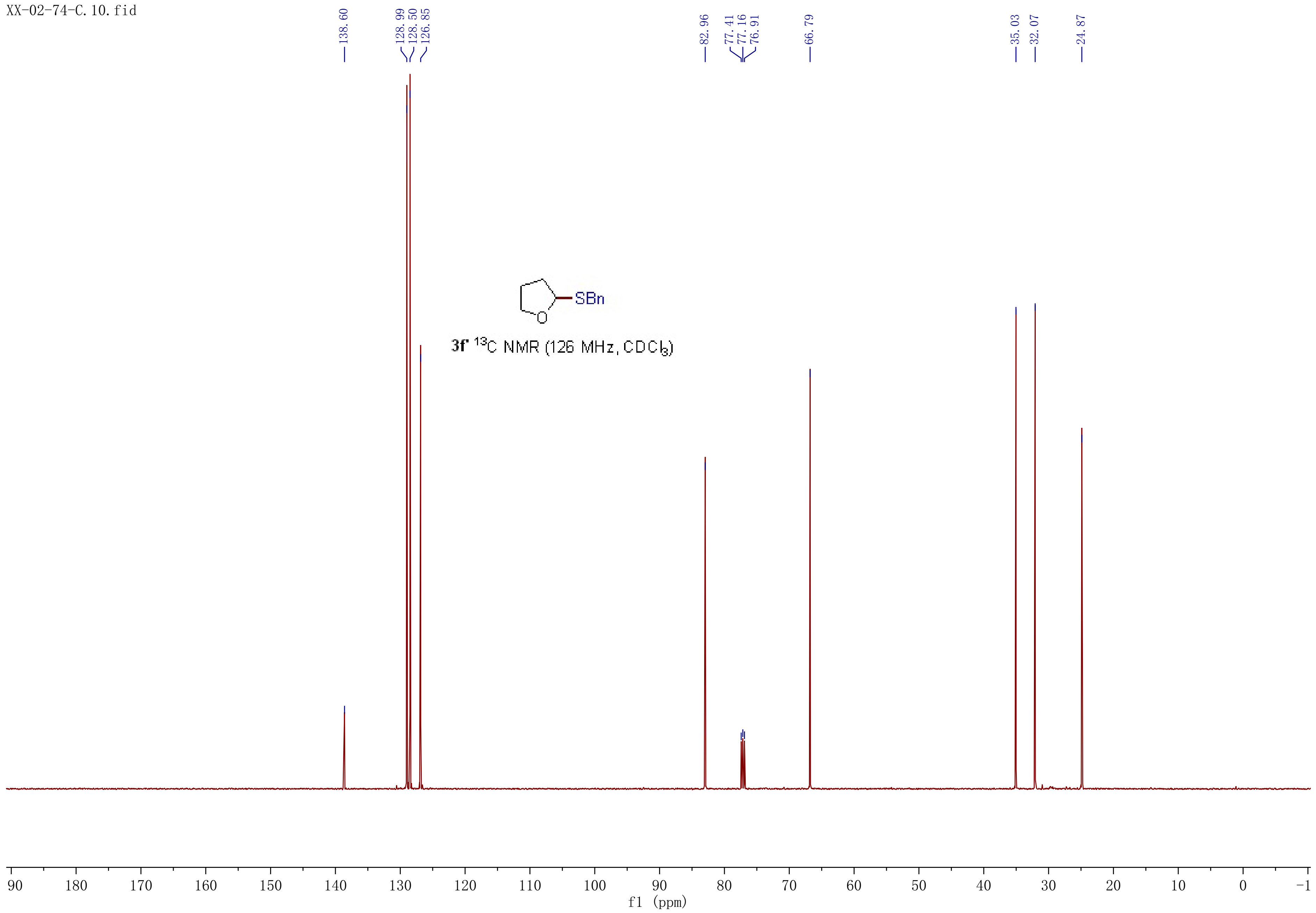

Supplementary Figure 165. ^{13}C NMR (126 MHz, $CDCl_3$) spectra for compound **3f'**

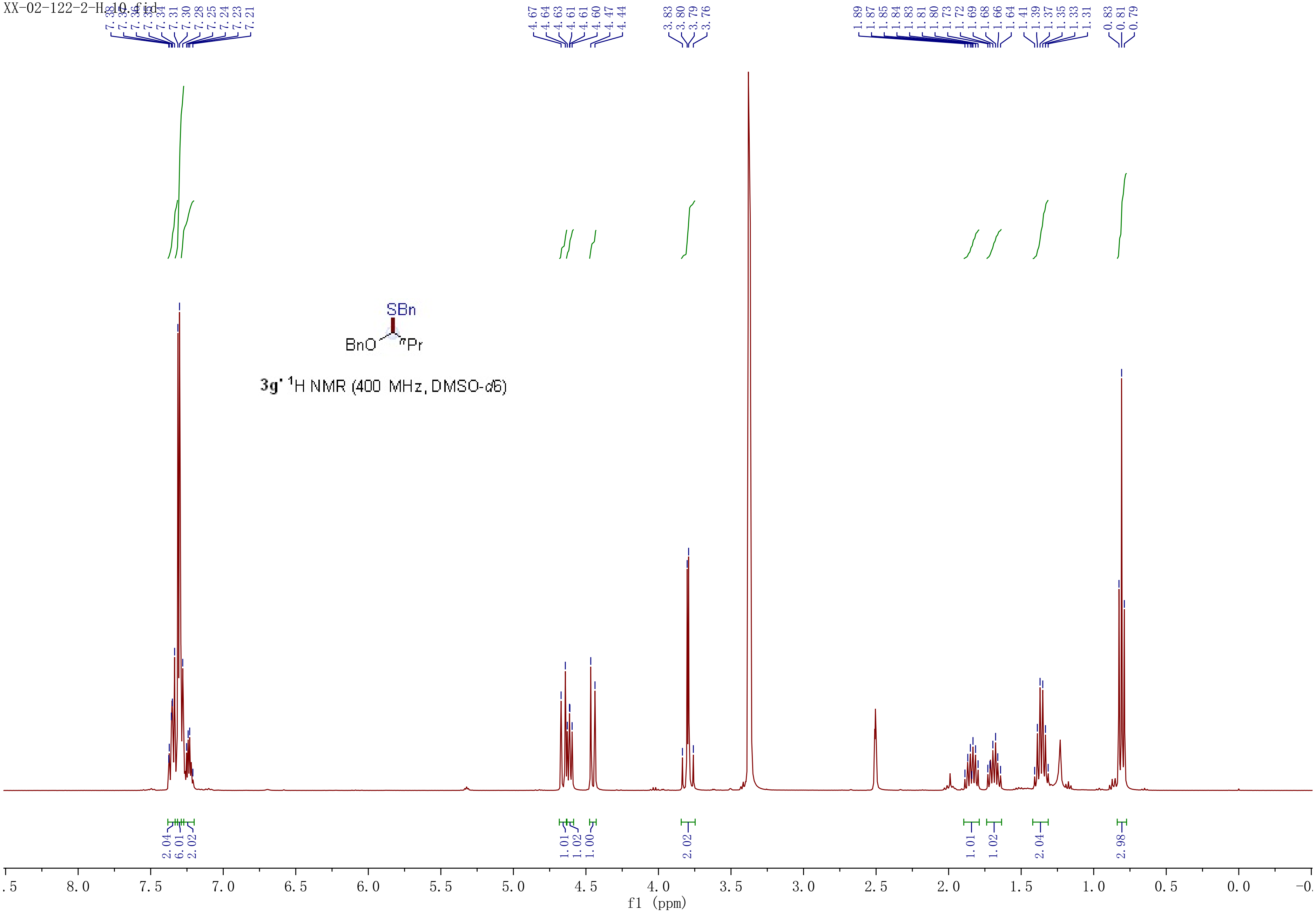

Supplementary Figure 166. ^{1}H NMR (400 MHz, DMSO-d_6) spectra for compound **3g'**

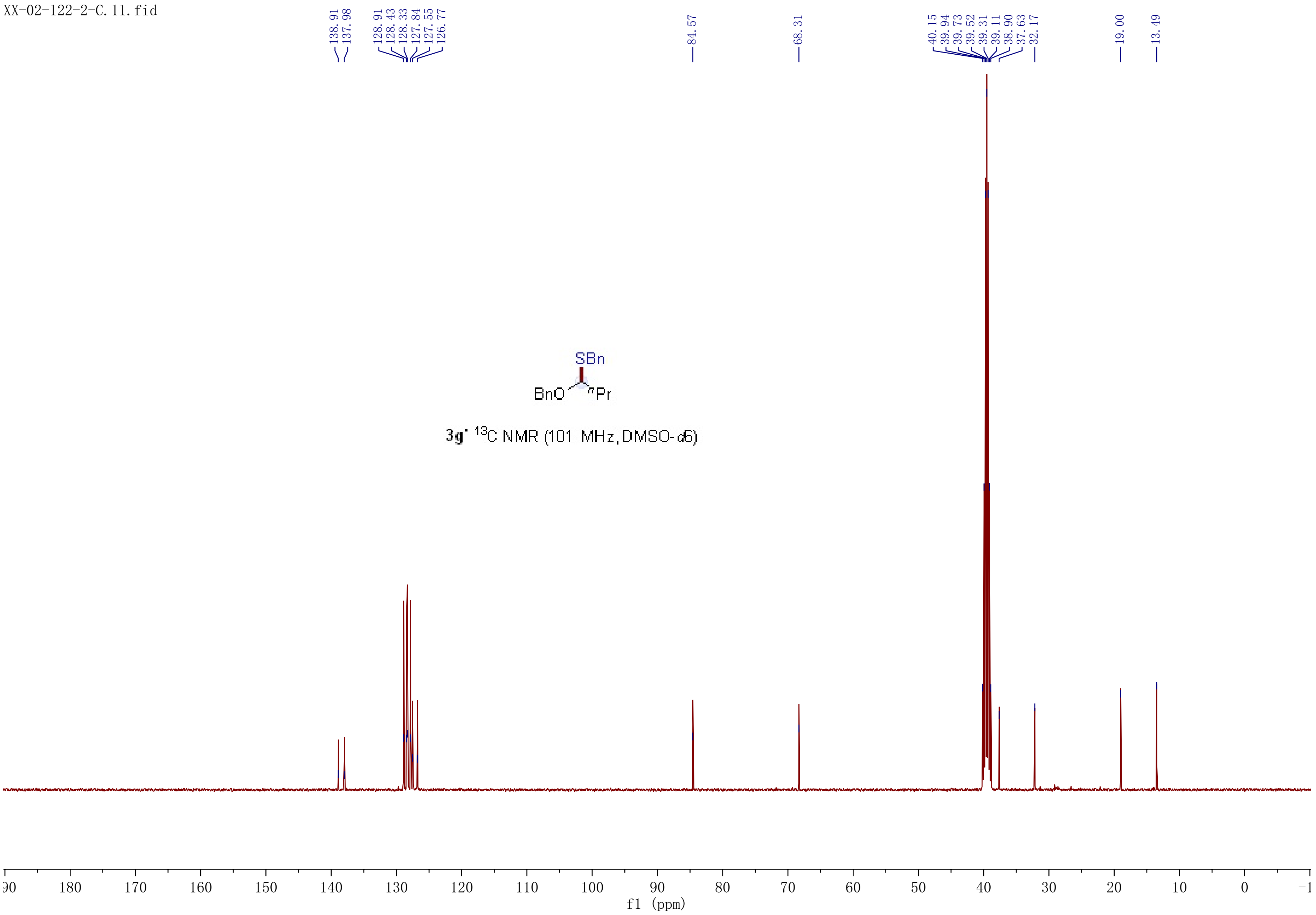

Supplementary Figure 167. ^{13}C NMR (101 MHz, DMSO-d_6) spectra for compound **3g'**

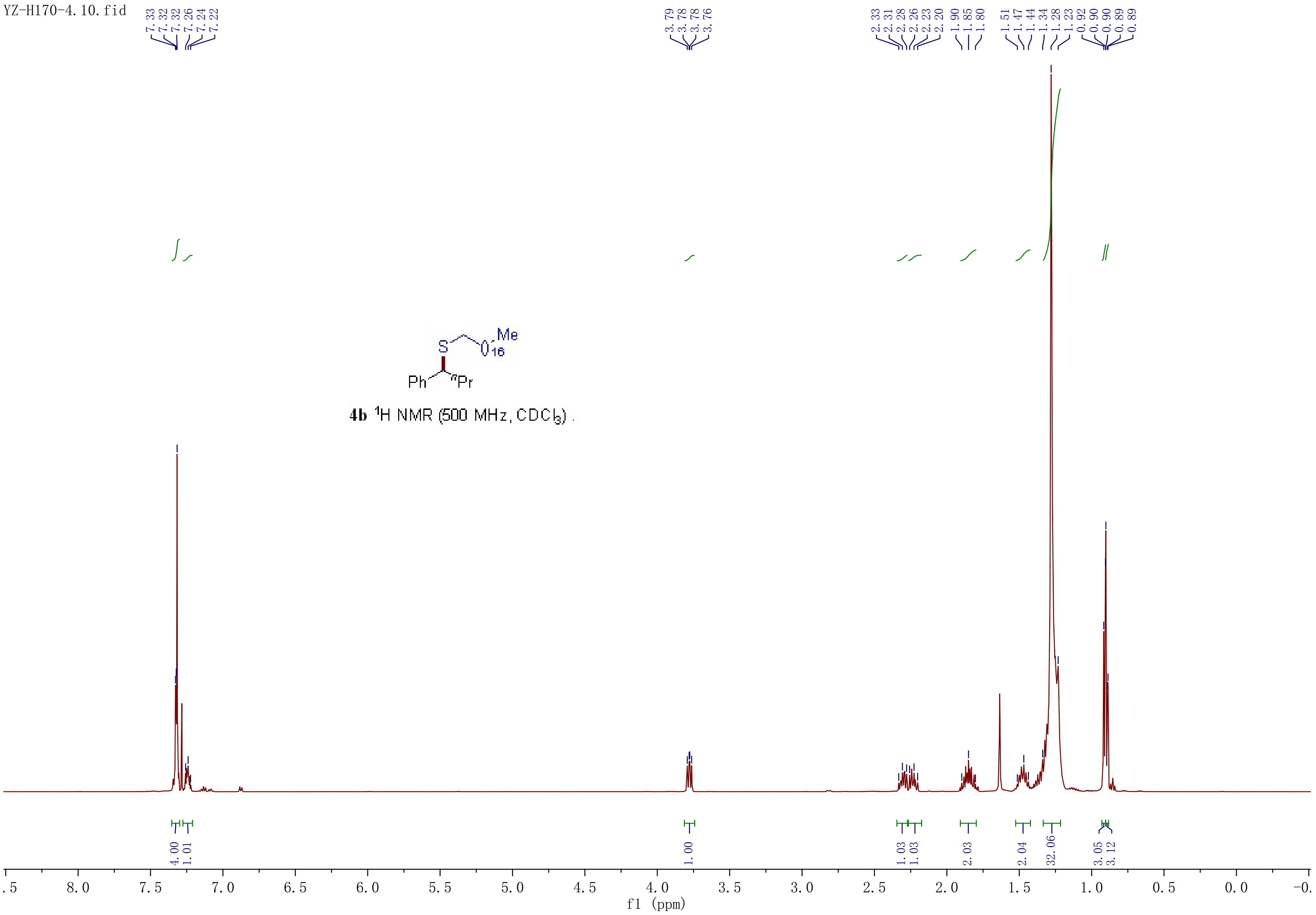

Supplementary Figure 168. ¹H NMR (500 MHz, $CDCl_3$) spectra for compound **4b**

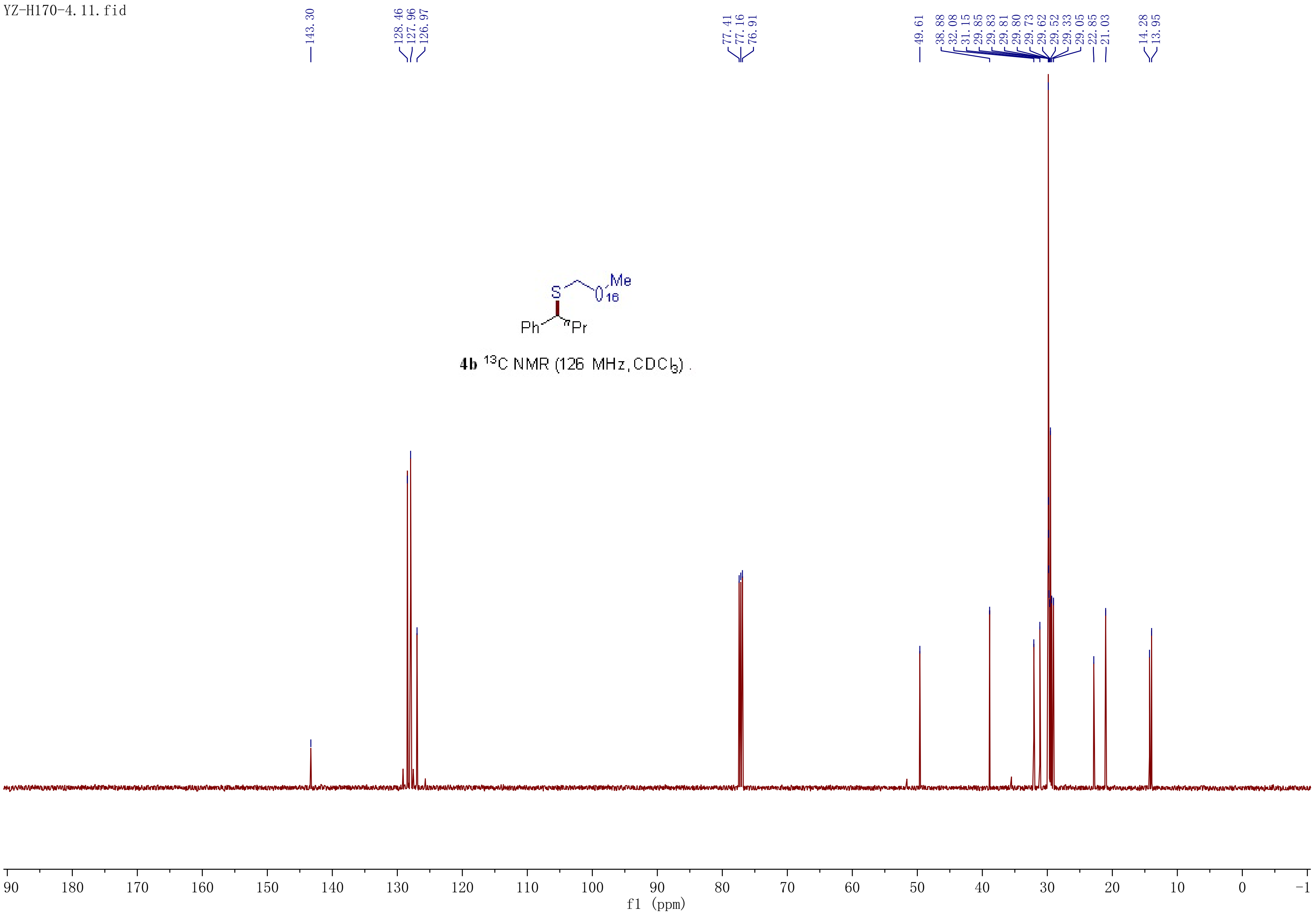

Supplementary Figure 169. ^{13}C NMR (126 MHz, $CDCl_3$) spectra for compound **4b**

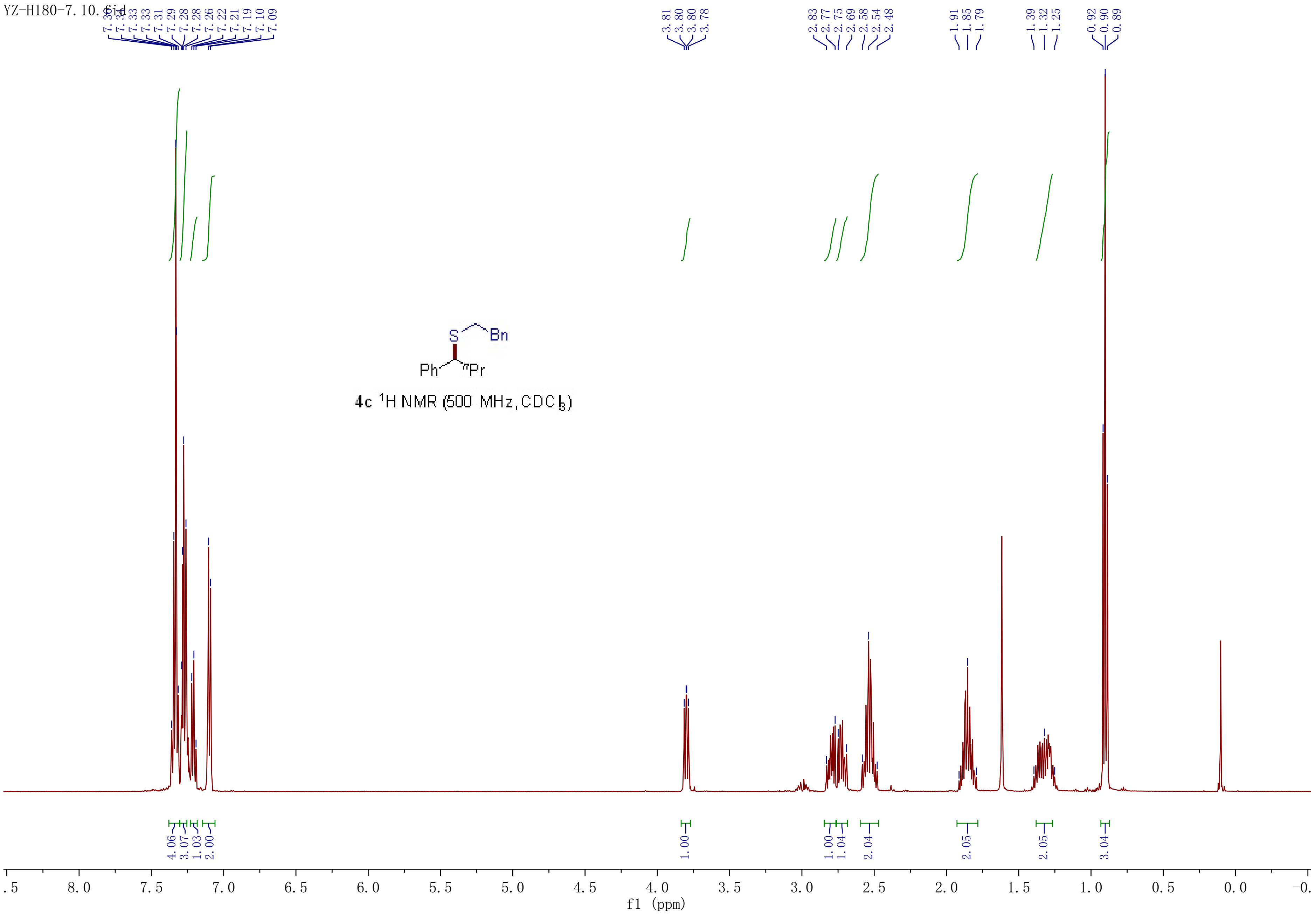

Supplementary Figure 170. ^{1}H NMR (500 MHz, $CDCl_3$) spectra for compound **4c**

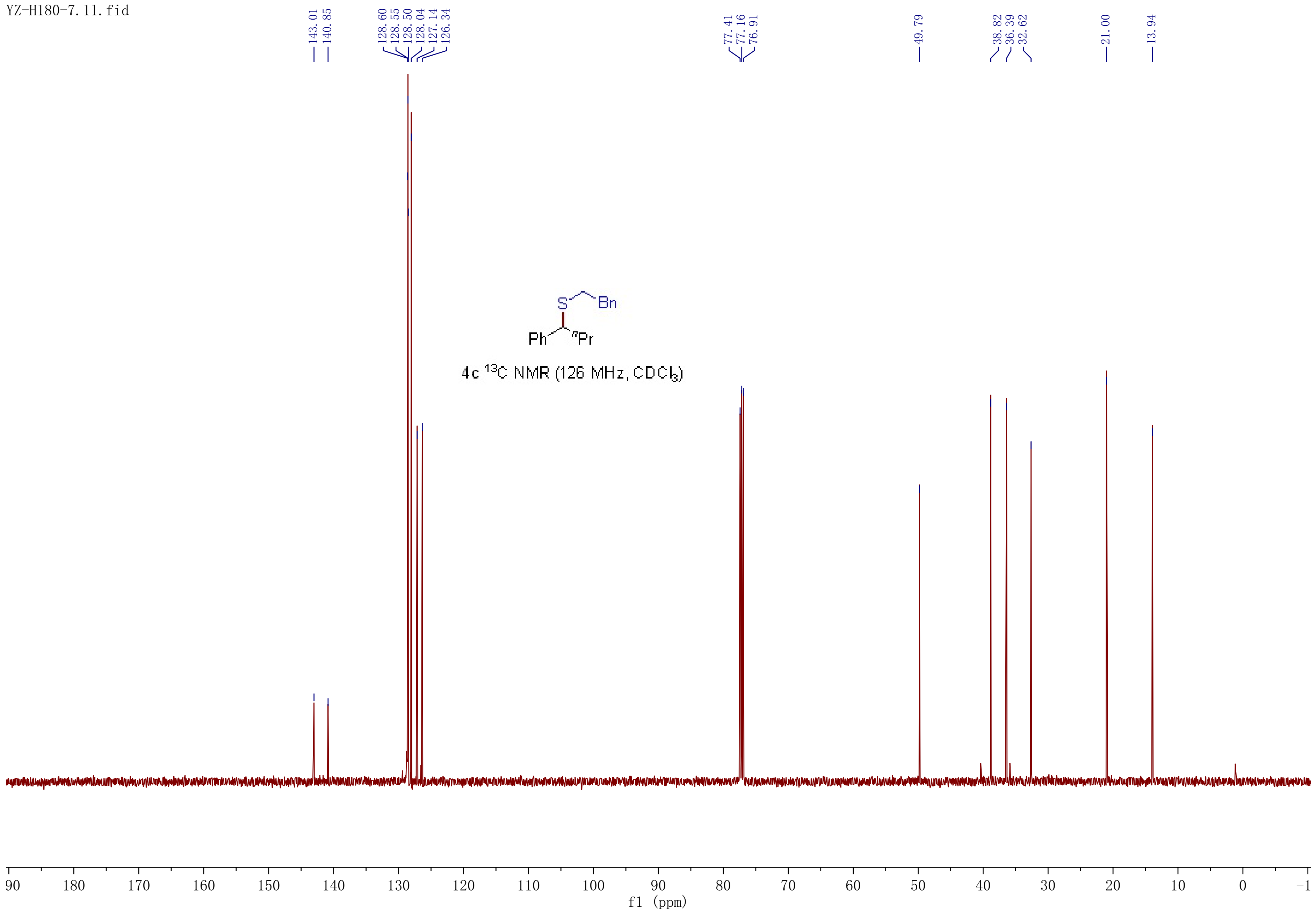

Supplementary Figure 171. ^{13}C NMR (126 MHz, $CDCl_3$) spectra for compound **4c**

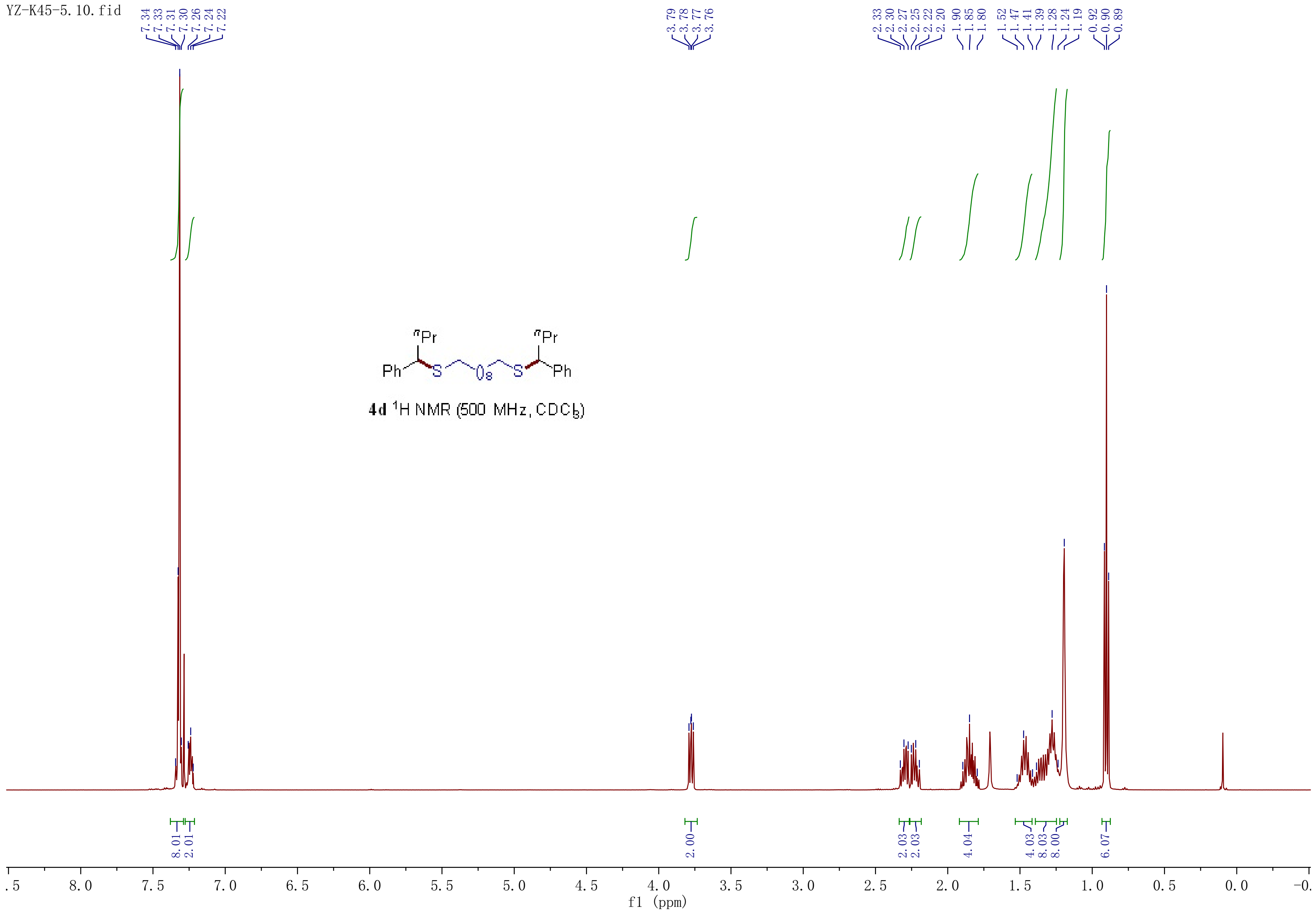

Supplementary Figure 172. ^{1}H NMR (500 MHz, $CDCl_3$) spectra for compound **4d**

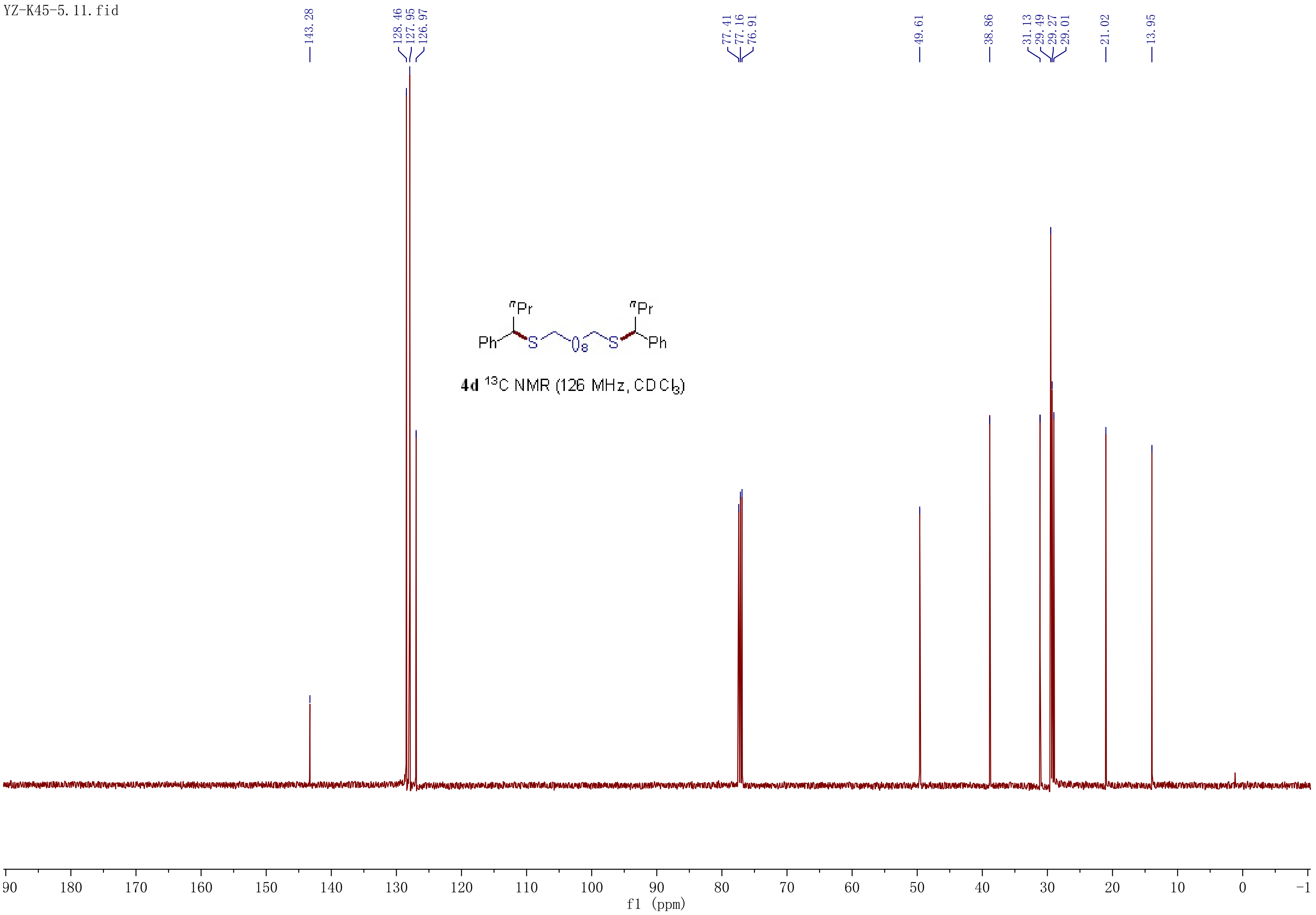

Supplementary Figure 173. ^{13}C NMR (126 MHz, $CDCl_3$) spectra for compound **4d**

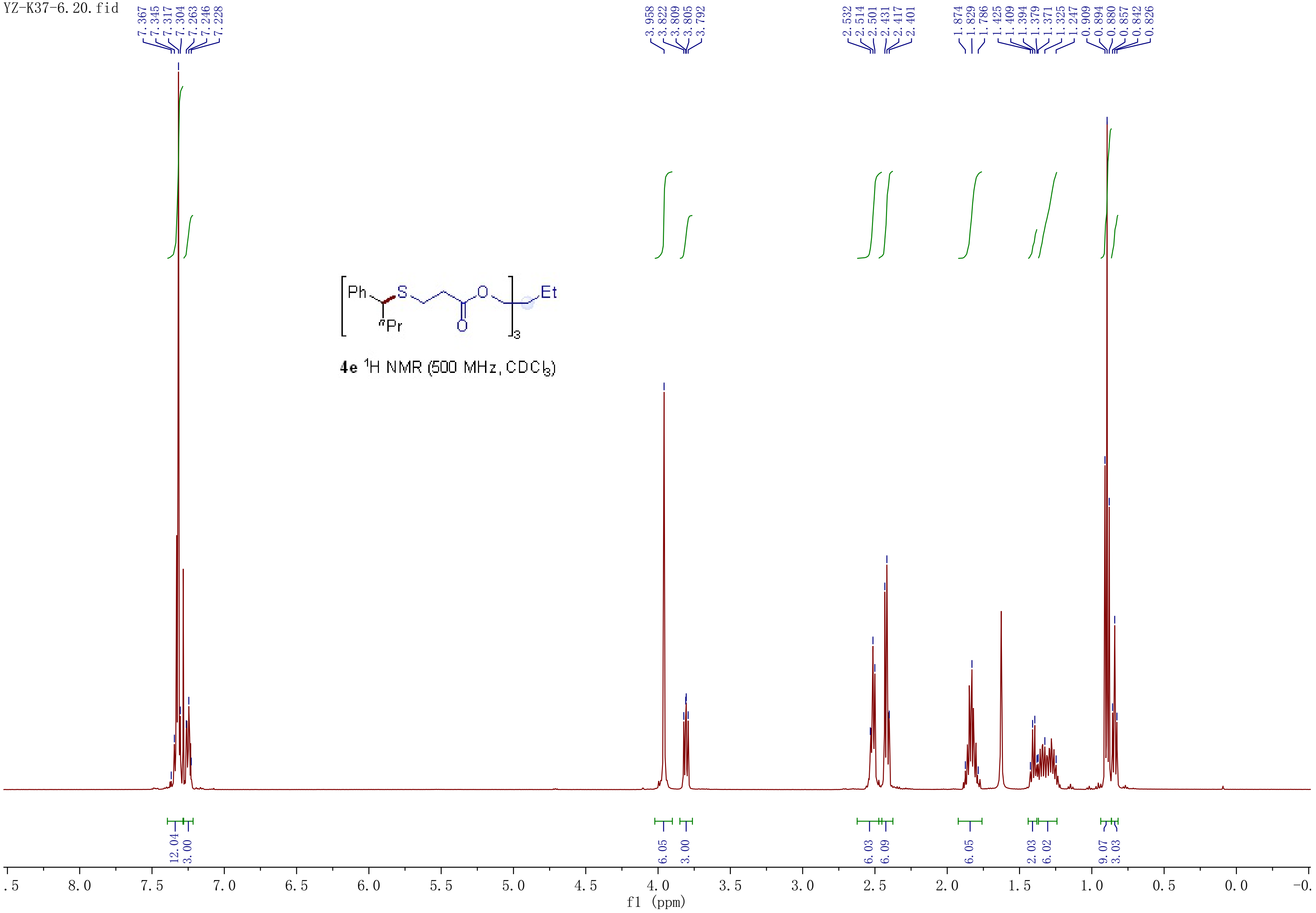

Supplementary Figure 174. ^{1}H NMR (500 MHz, $CDCl_3$) spectra for compound **4e**

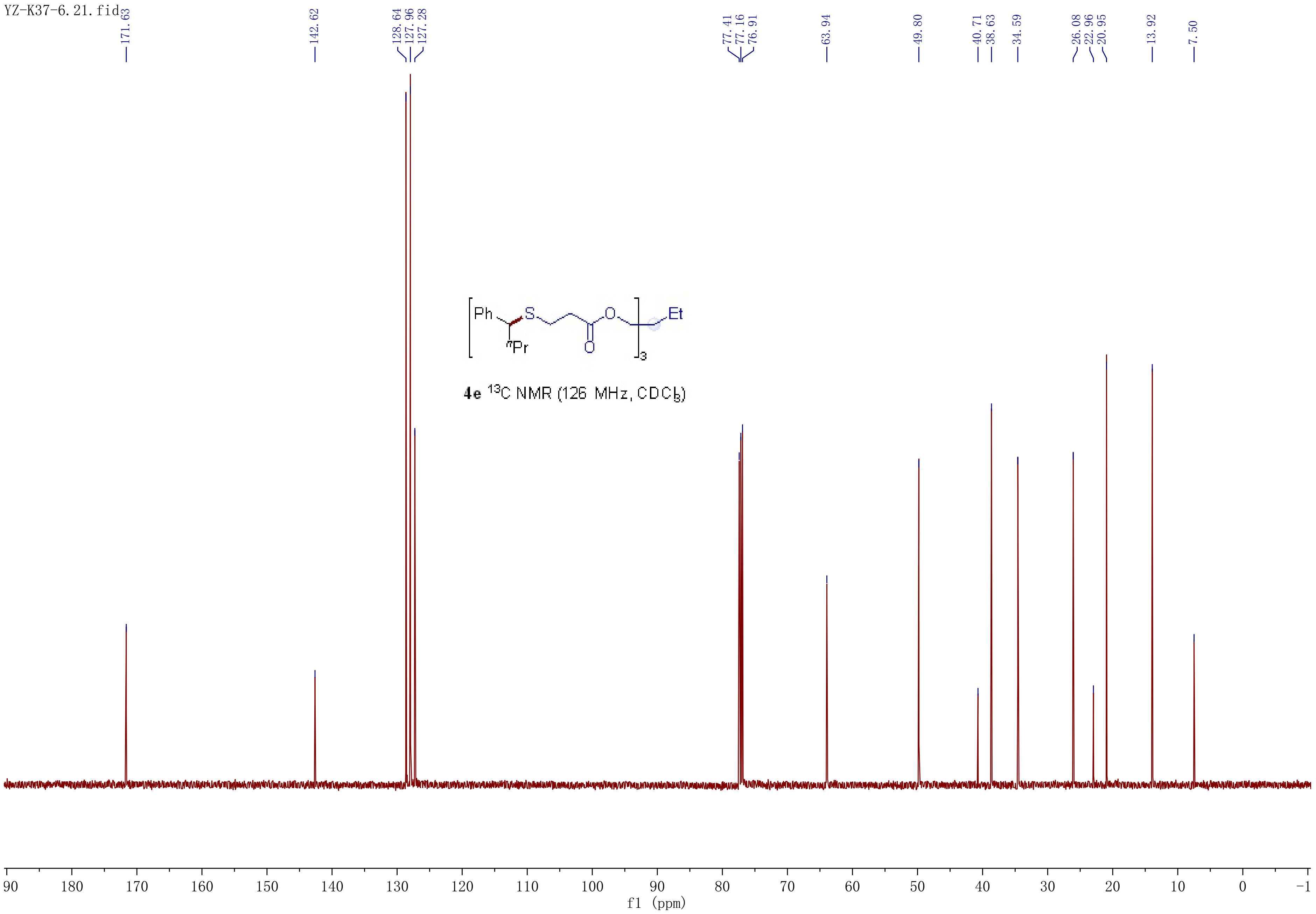

Supplementary Figure 175. ^{13}C NMR (126 MHz, $CDCl_3$) spectra for compound **4e**

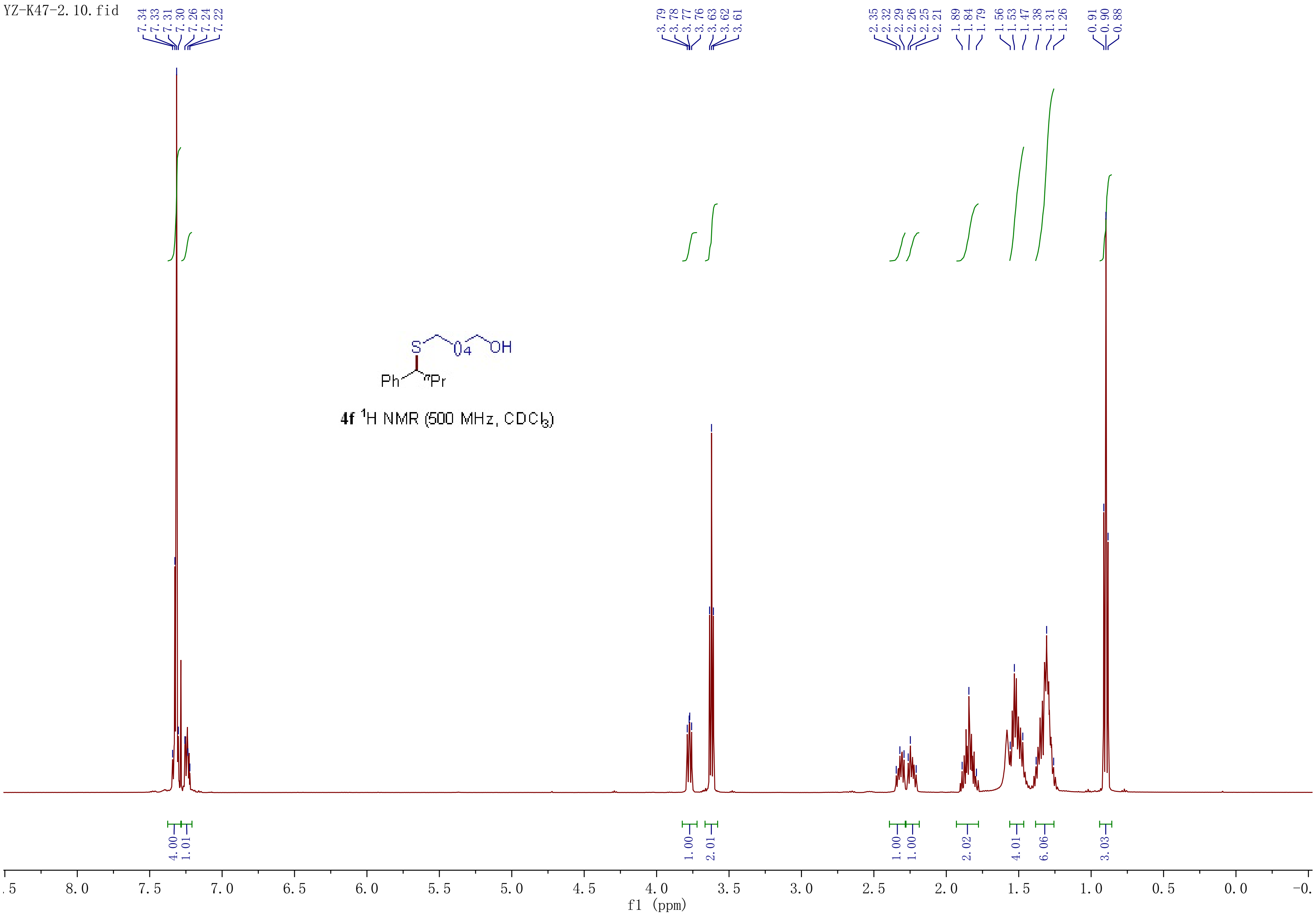

Supplementary Figure 176. ^{1}H NMR (500 MHz, $CDCl_3$) spectra for compound **4f**

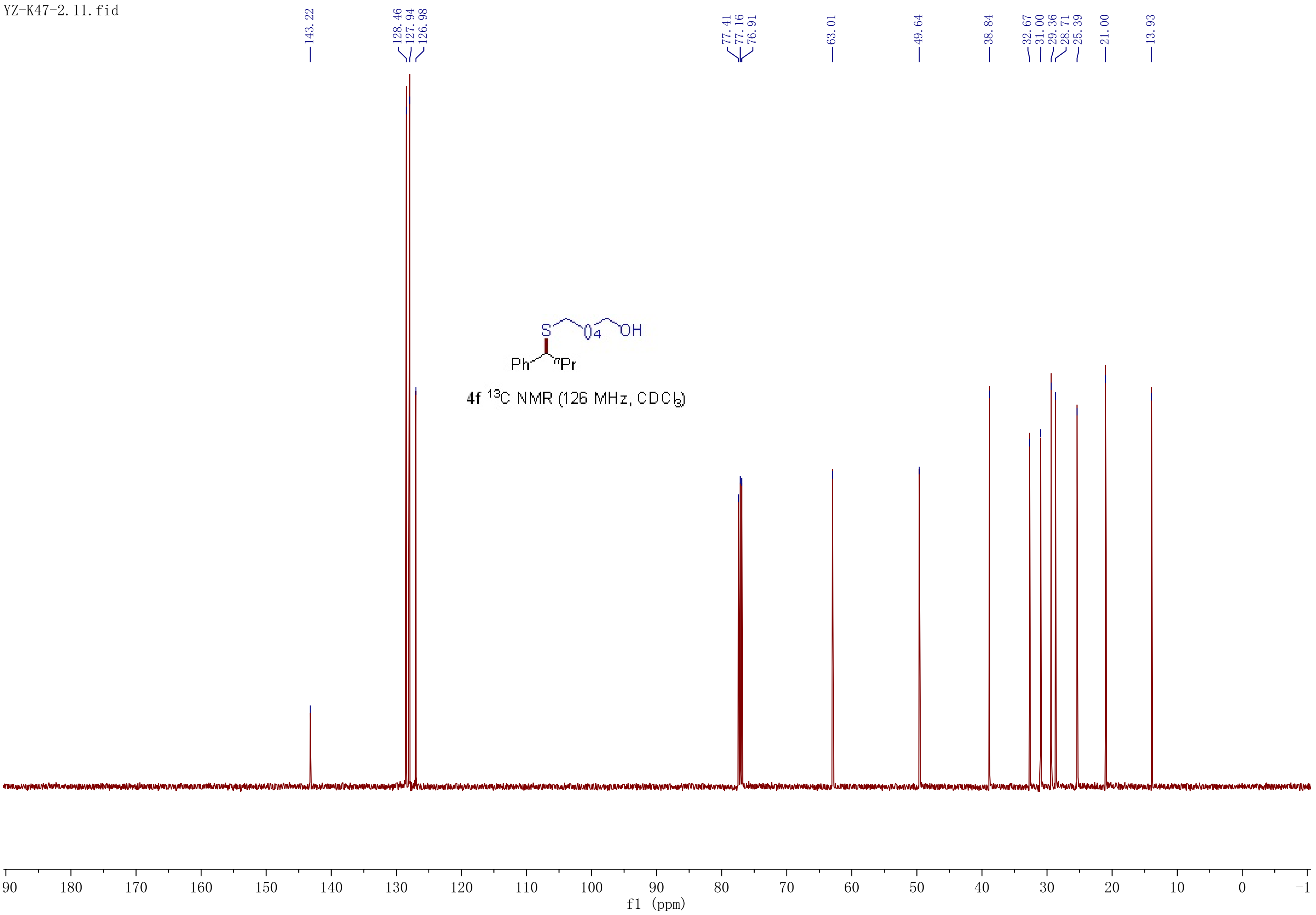

Supplementary Figure 177. ^{13}C NMR (126 MHz, $CDCl_3$) spectra for compound **4f**

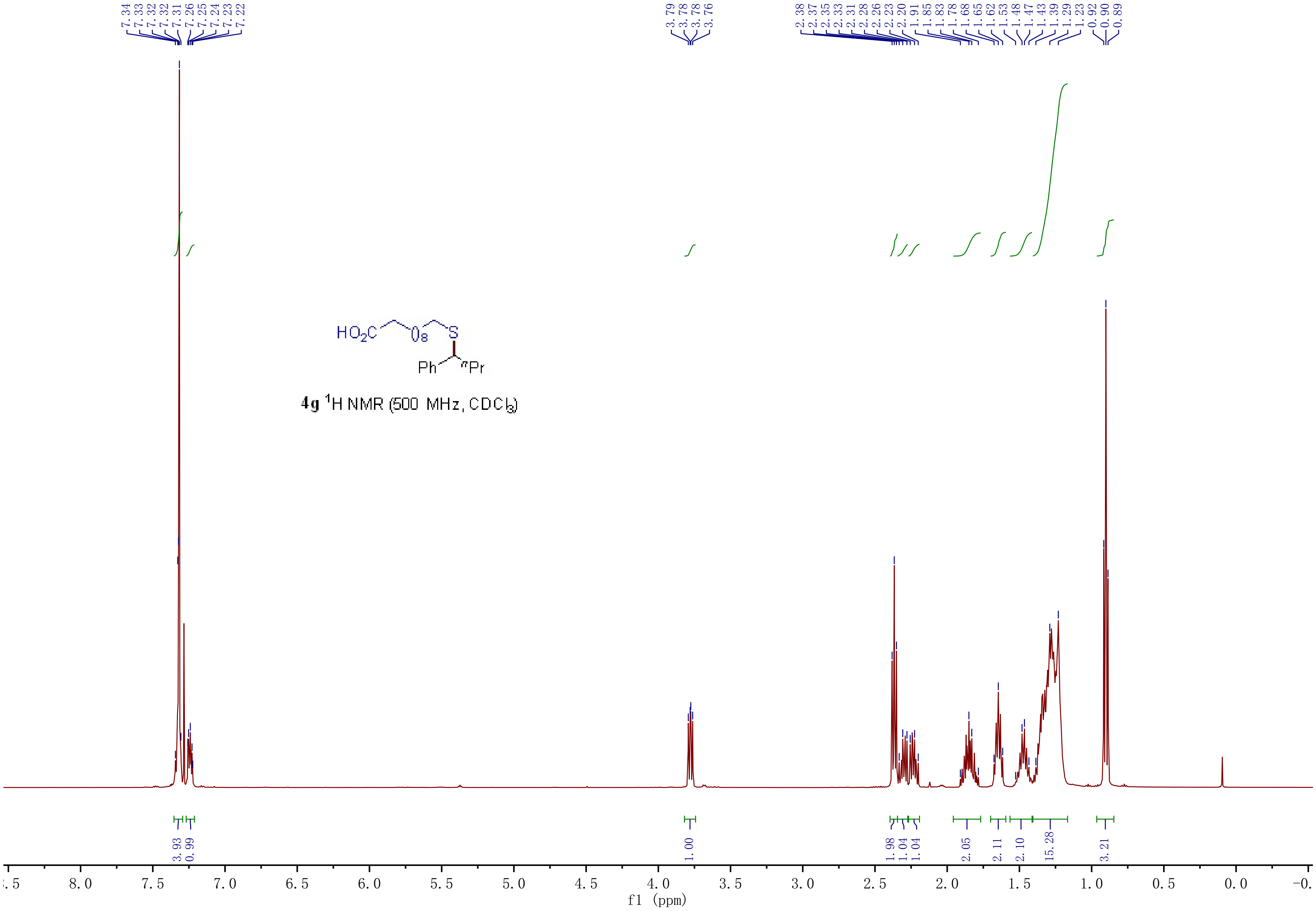

Supplementary Figure 178. ^{1}H NMR (500 MHz, $CDCl_3$) spectra for compound **4g**

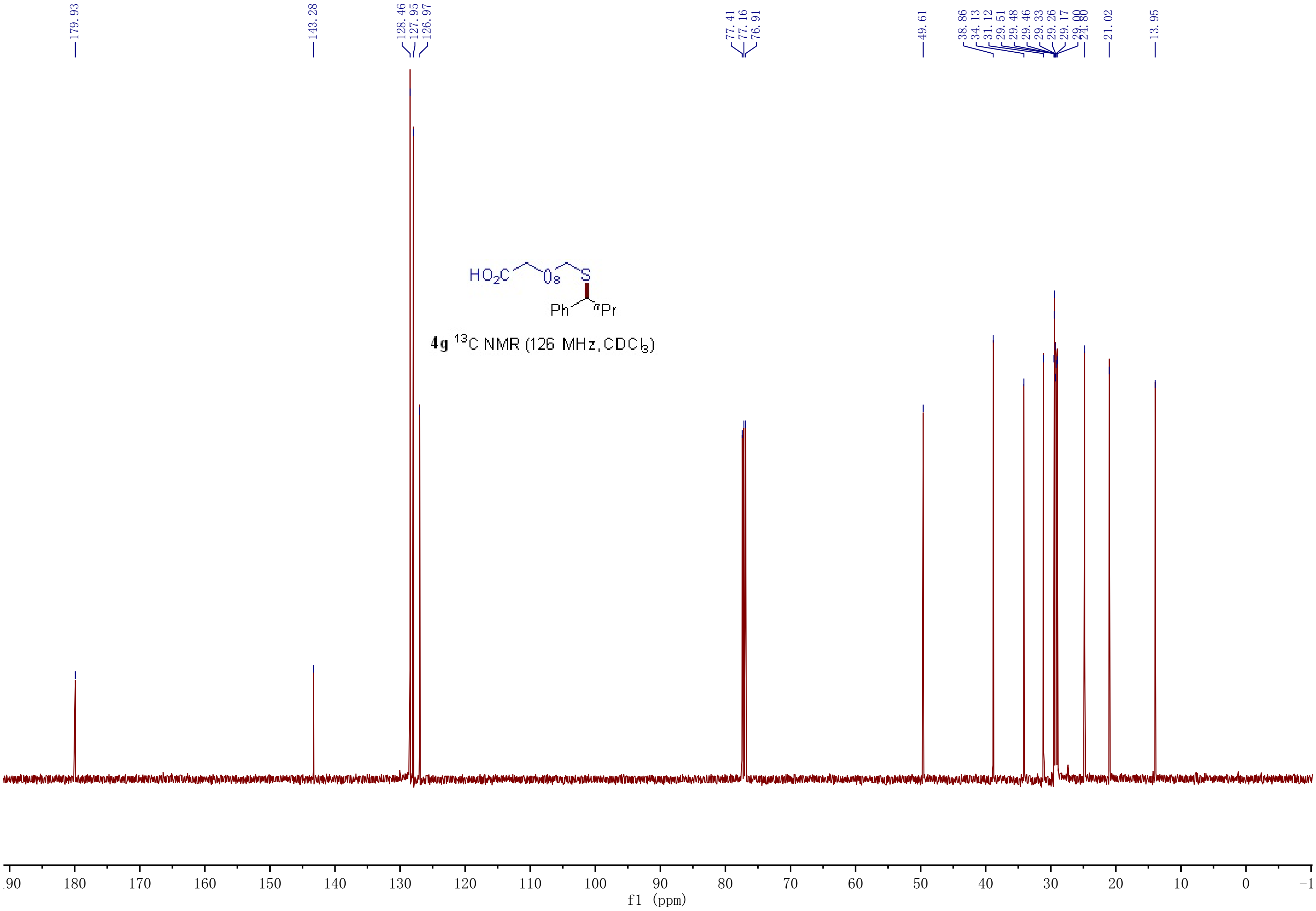

Supplementary Figure 179. ^{13}C NMR (126 MHz, $CDCl_3$) spectra for compound **4g**

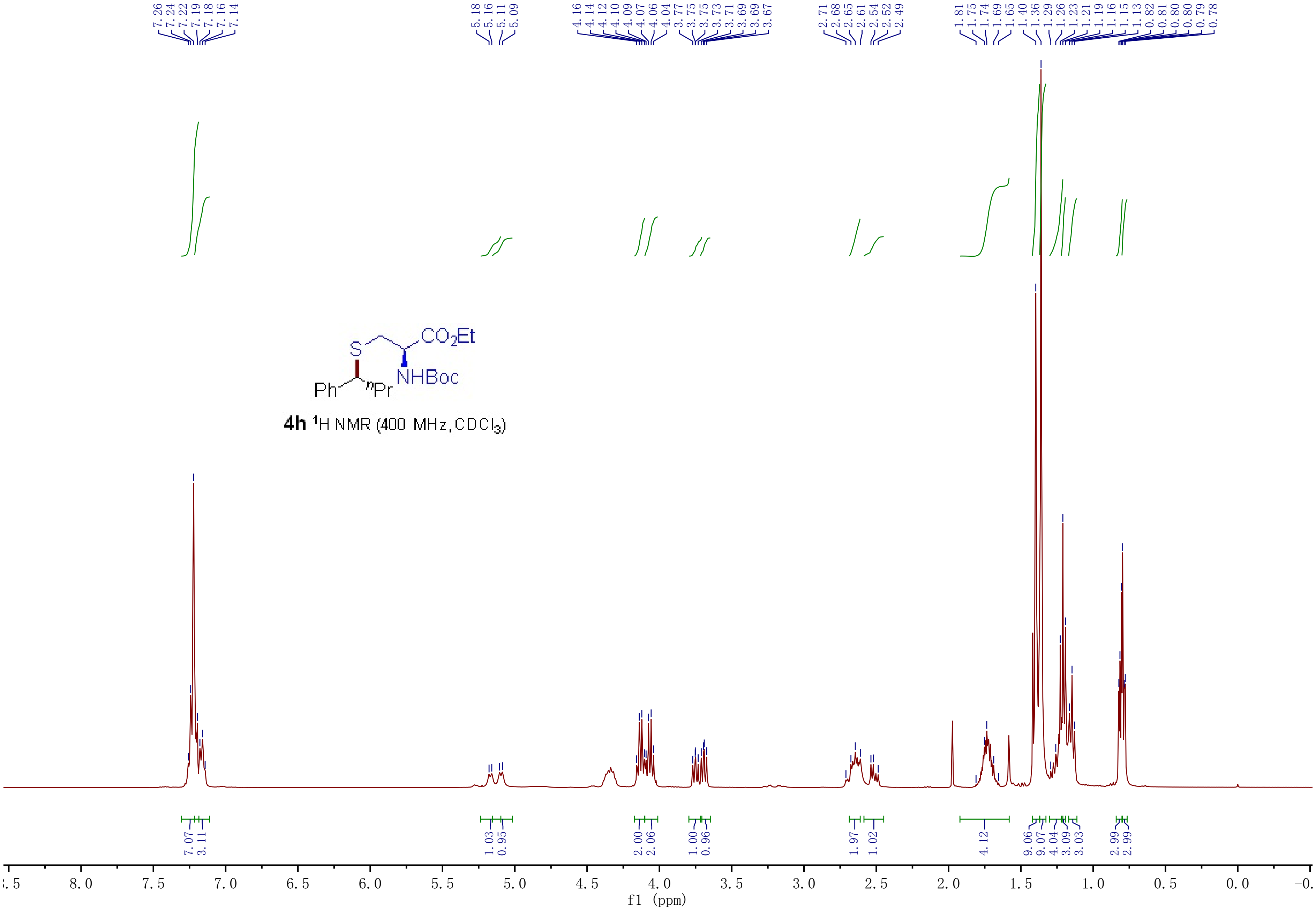

Supplementary Figure 180. ¹H NMR (400 MHz, $CDCl_3$) spectra for compound **4h**

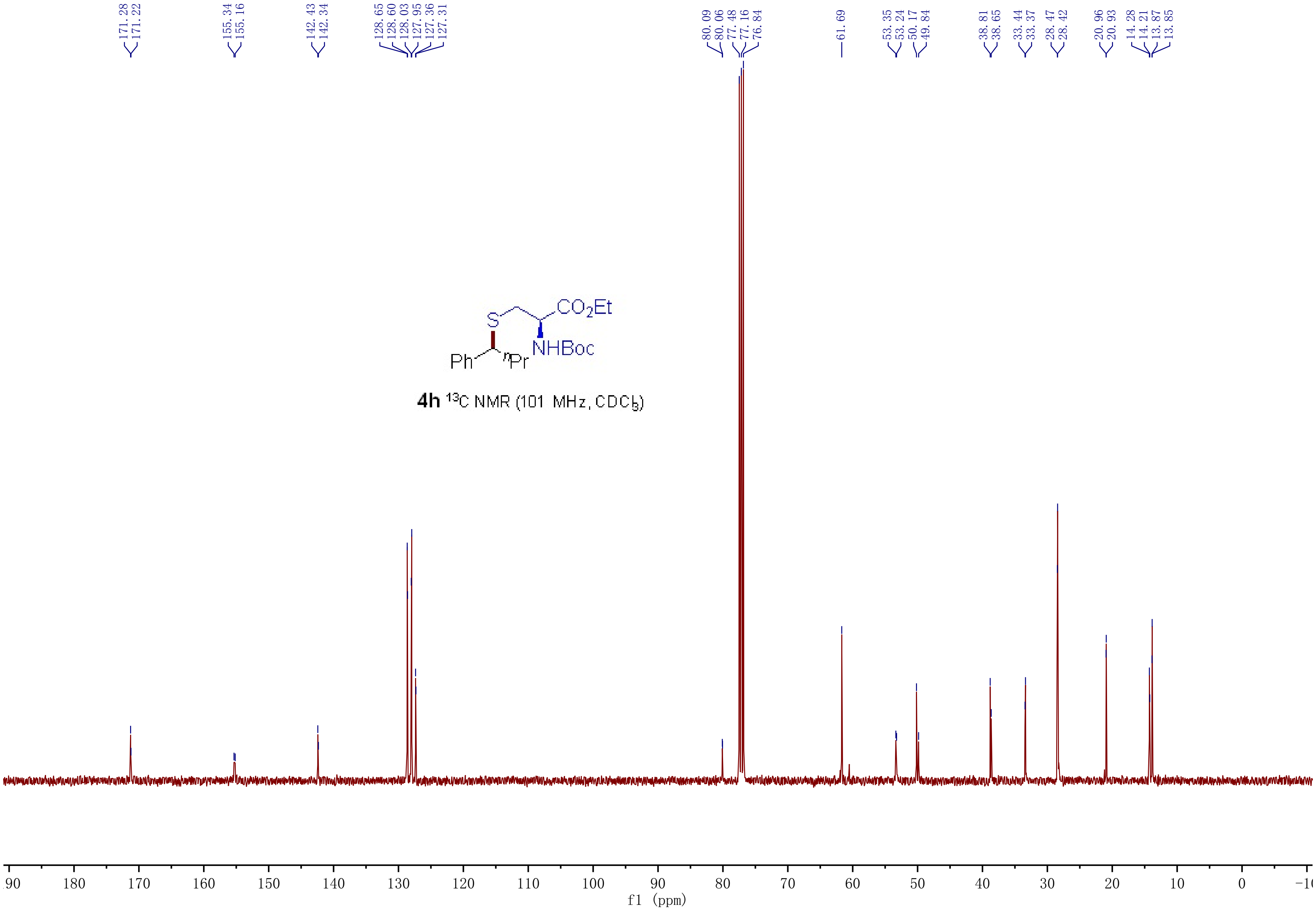

Supplementary Figure 181. ^{13}C NMR (101 MHz, $CDCl_3$) spectra for compound **4h**

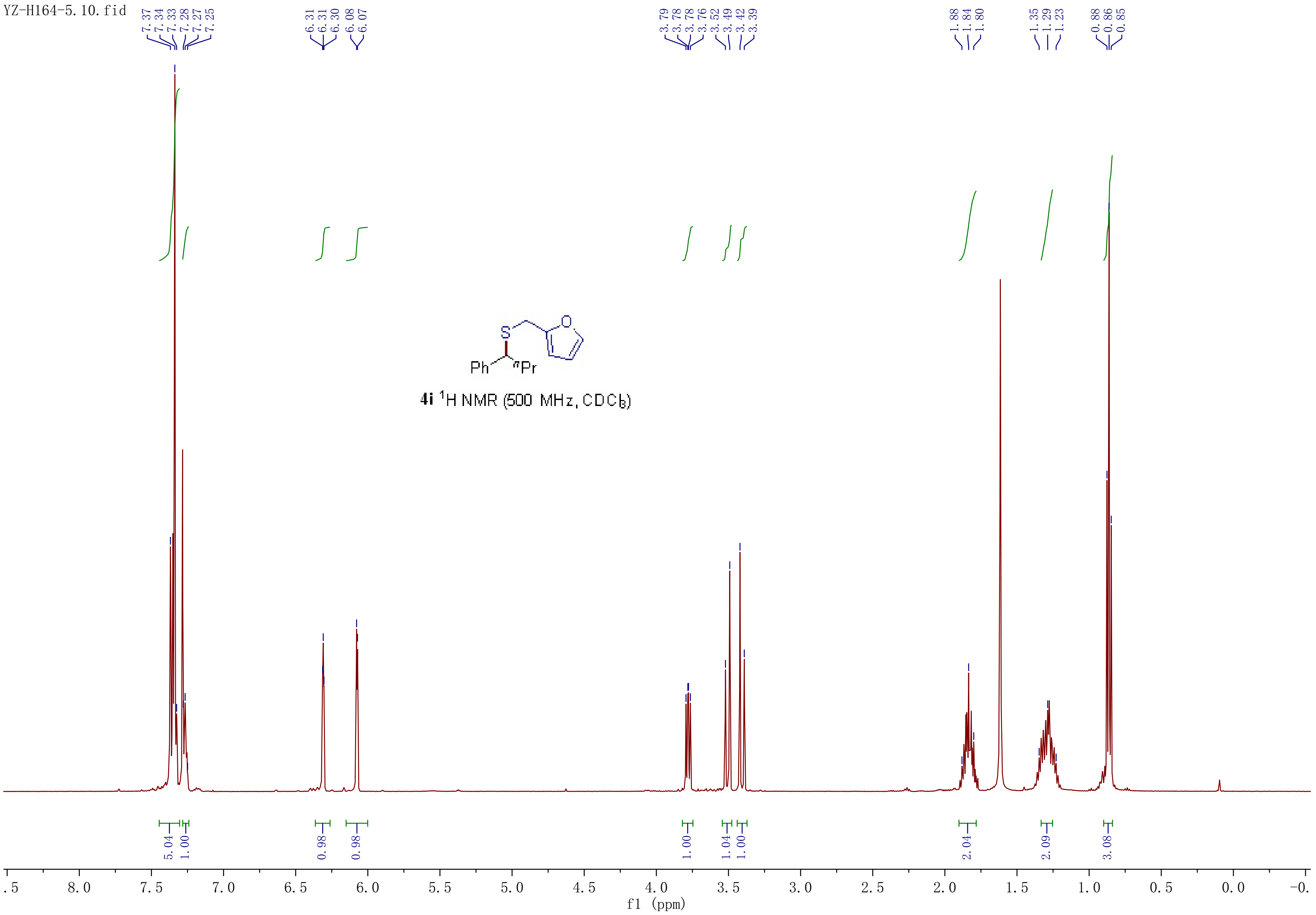

Supplementary Figure 182. ^{1}H NMR (500 MHz, $CDCl_3$) spectra for compound **4i**

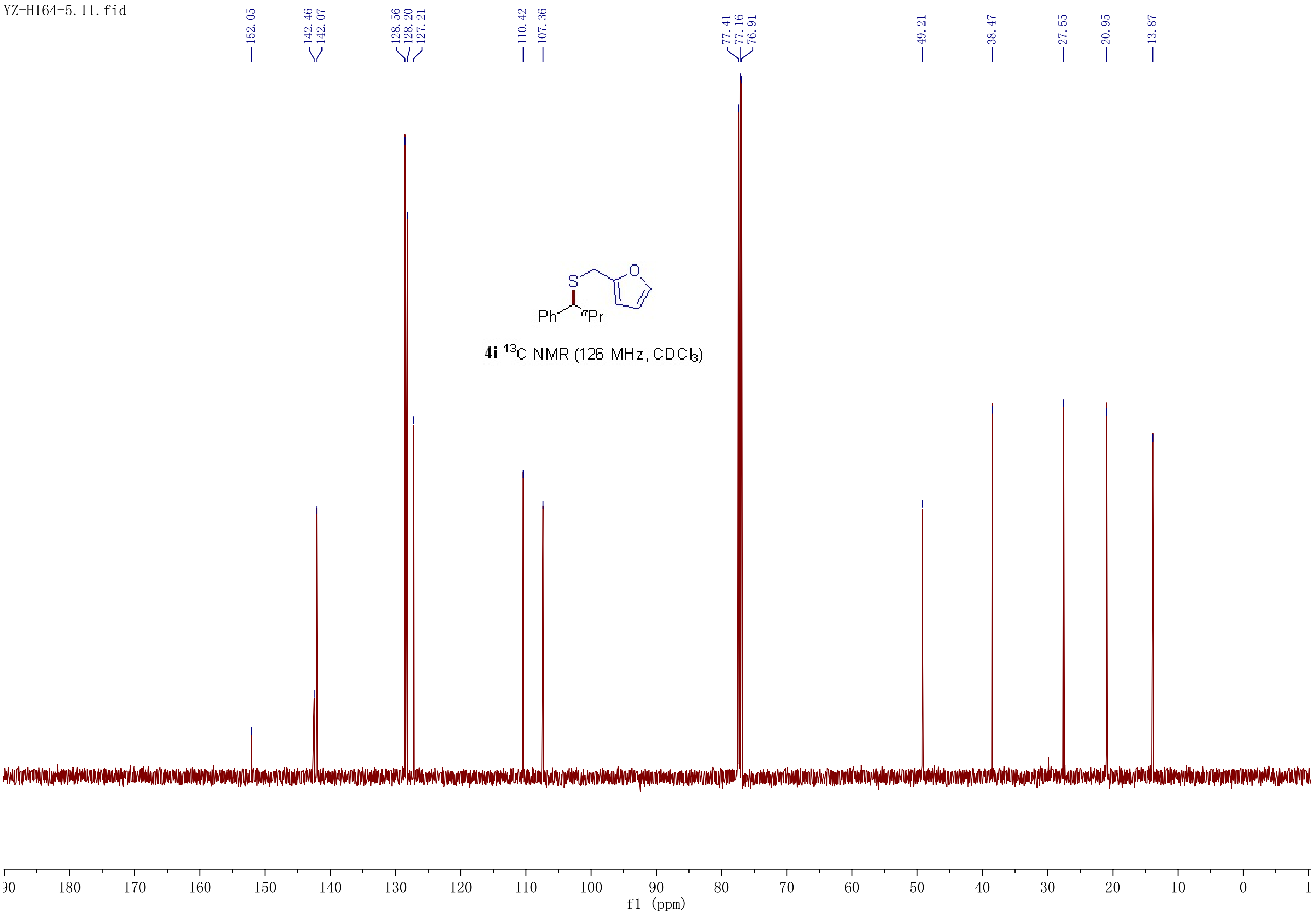

Supplementary Figure 183. ^{13}C NMR (126 MHz, $CDCl_3$) spectra for compound **4i**

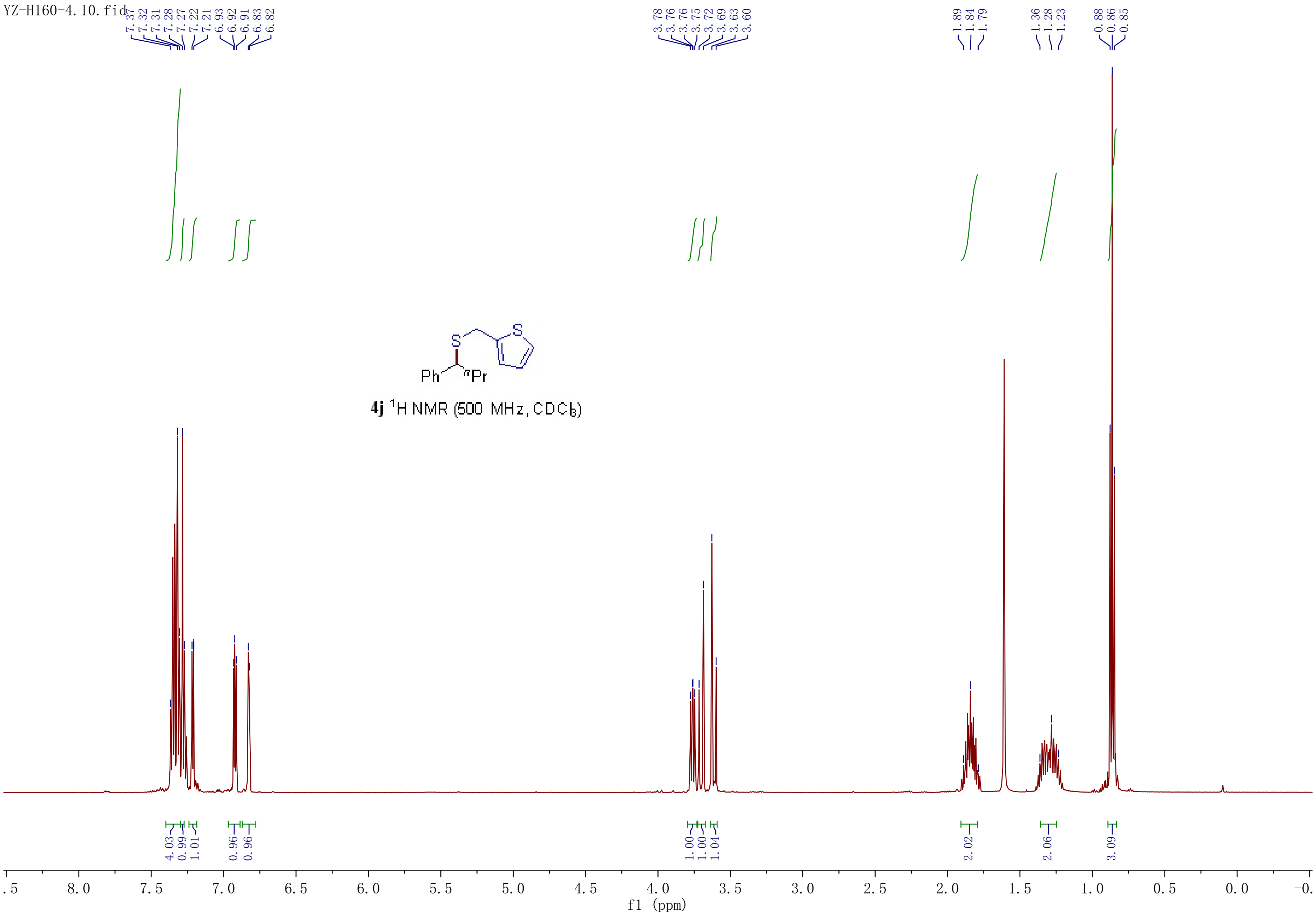

Supplementary Figure 184. ^{1}H NMR (500 MHz, $CDCl_3$) spectra for compound **4j**

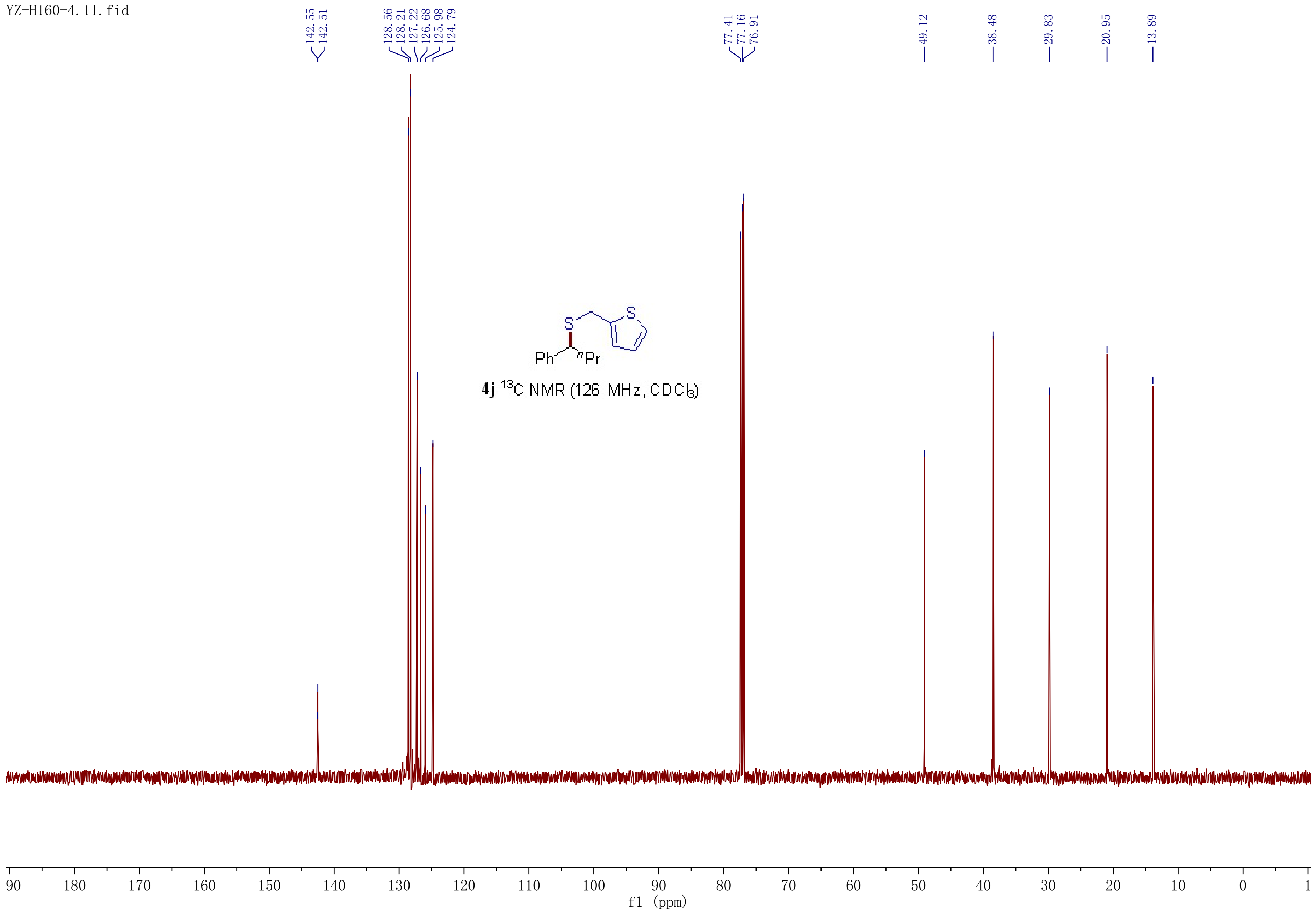

Supplementary Figure 185. ^{13}C NMR (126 MHz, $CDCl_3$) spectra for compound **4j**

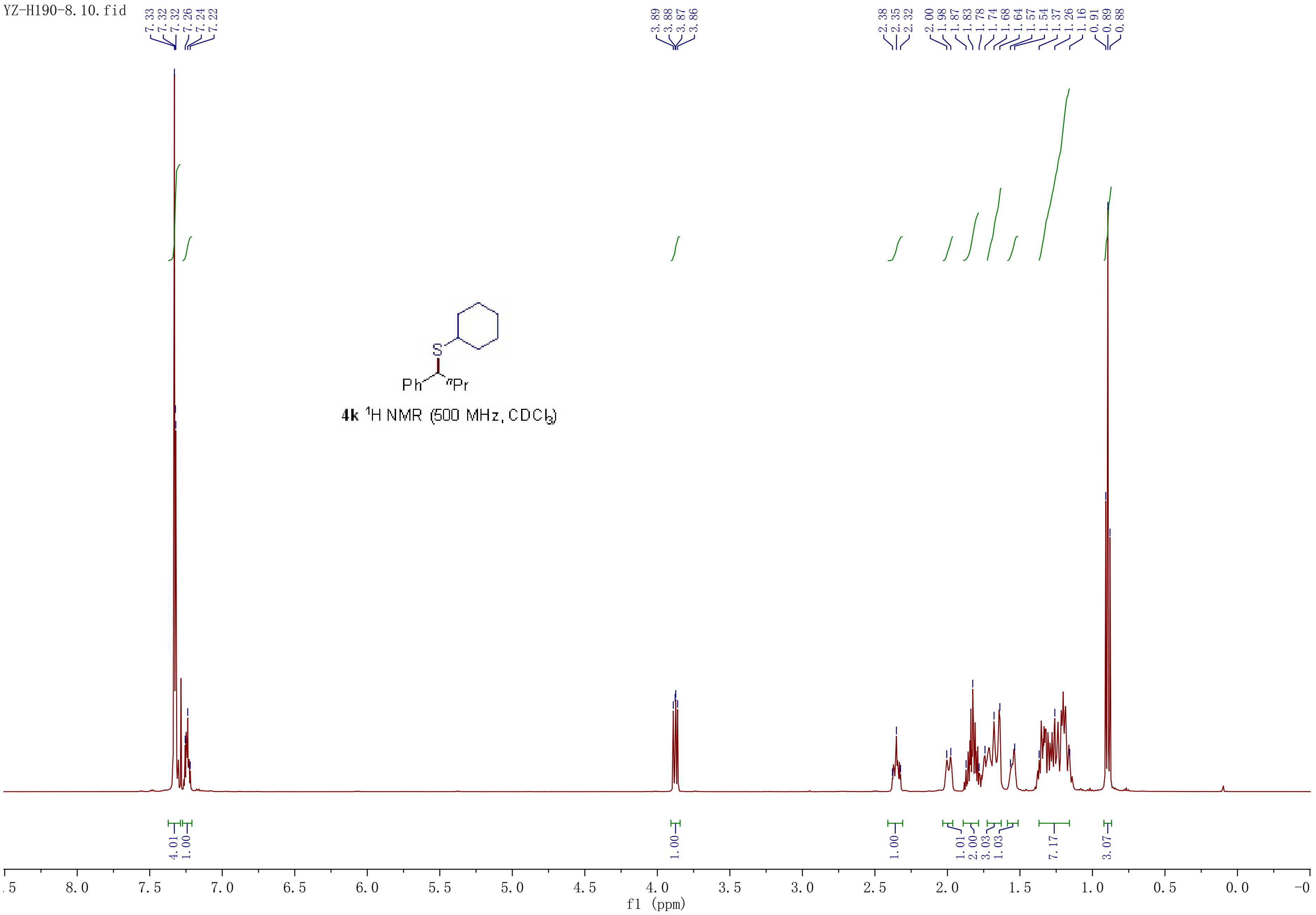

Supplementary Figure 186. ^{1}H NMR (500 MHz, $CDCl_3$) spectra for compound **4k**

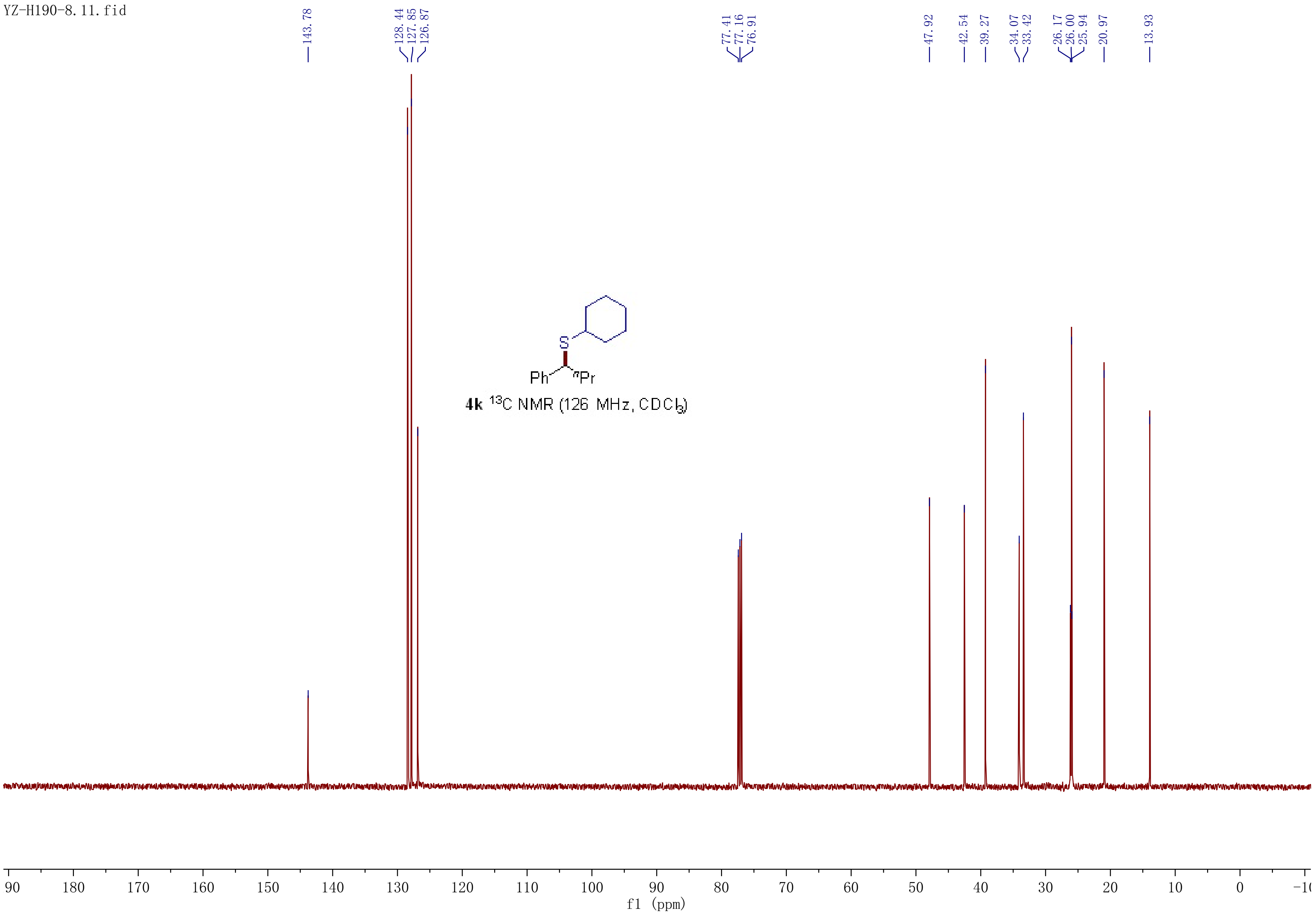

Supplementary Figure 187. ^{13}C NMR (126 MHz, $CDCl_3$) spectra for compound **4k**

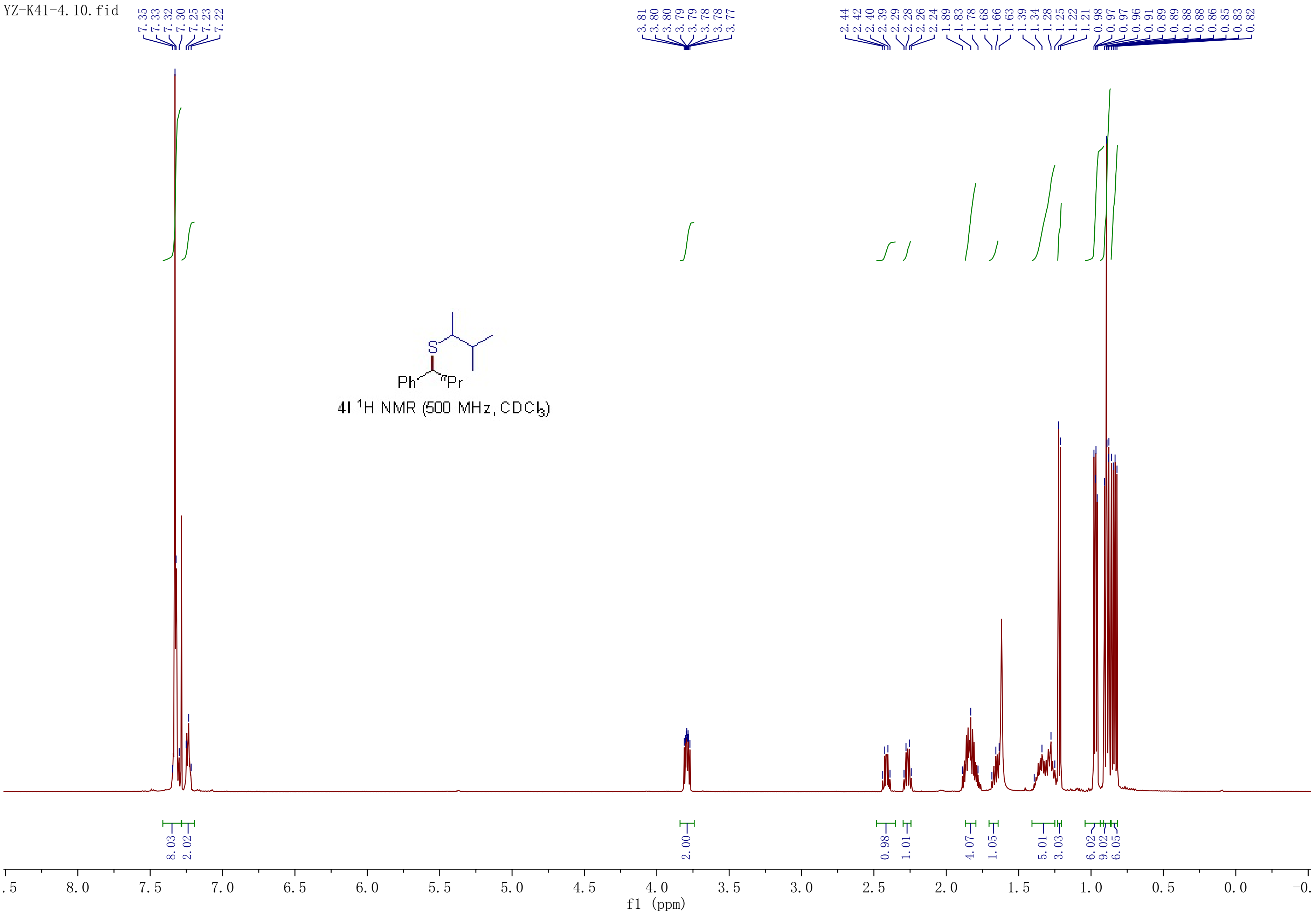

Supplementary Figure 188. ^{1}H NMR (500 MHz, $CDCl_3$) spectra for compound **4l**

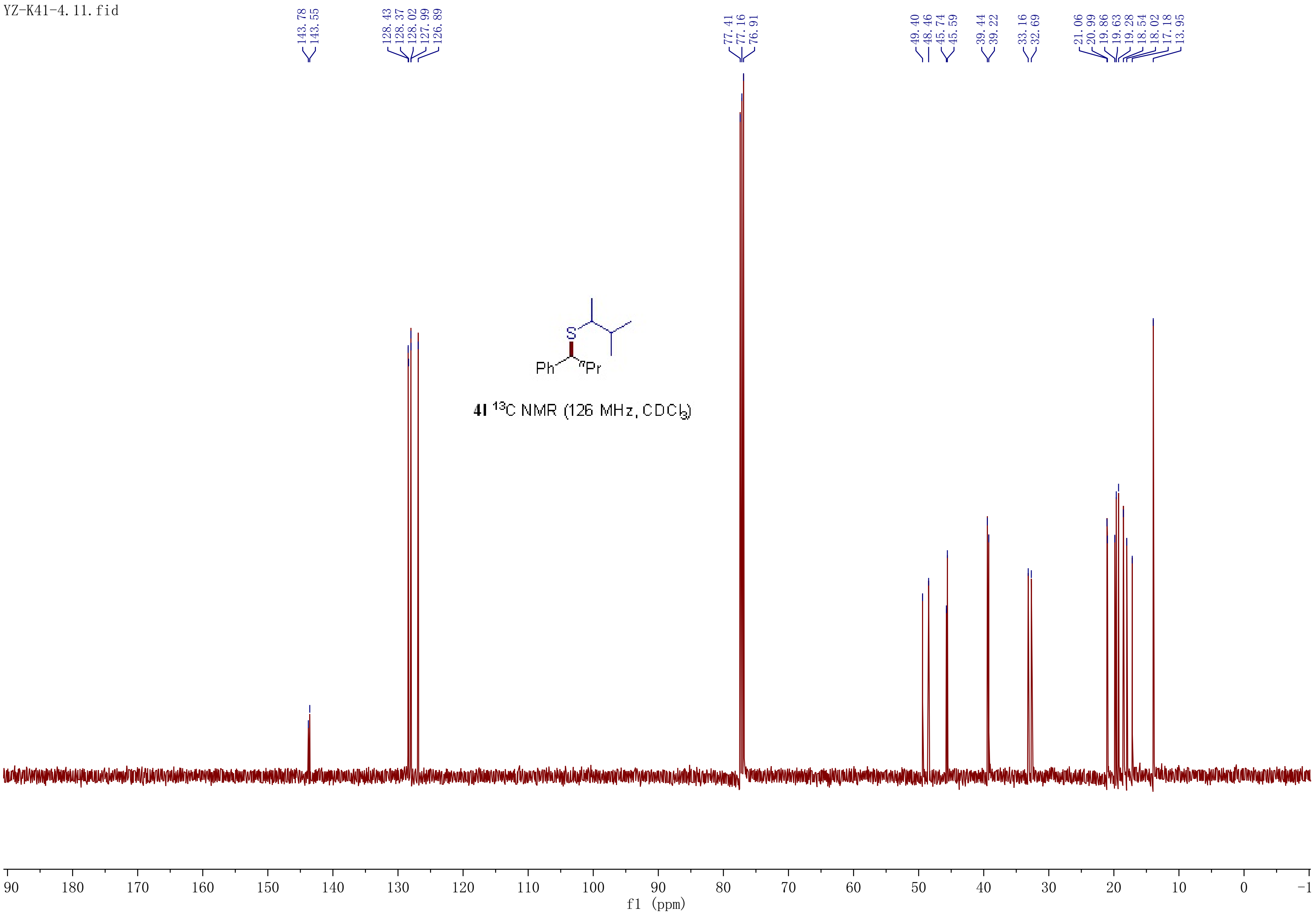

Supplementary Figure 189. ^{13}C NMR (126 MHz, $CDCl_3$) spectra for compound **4l**

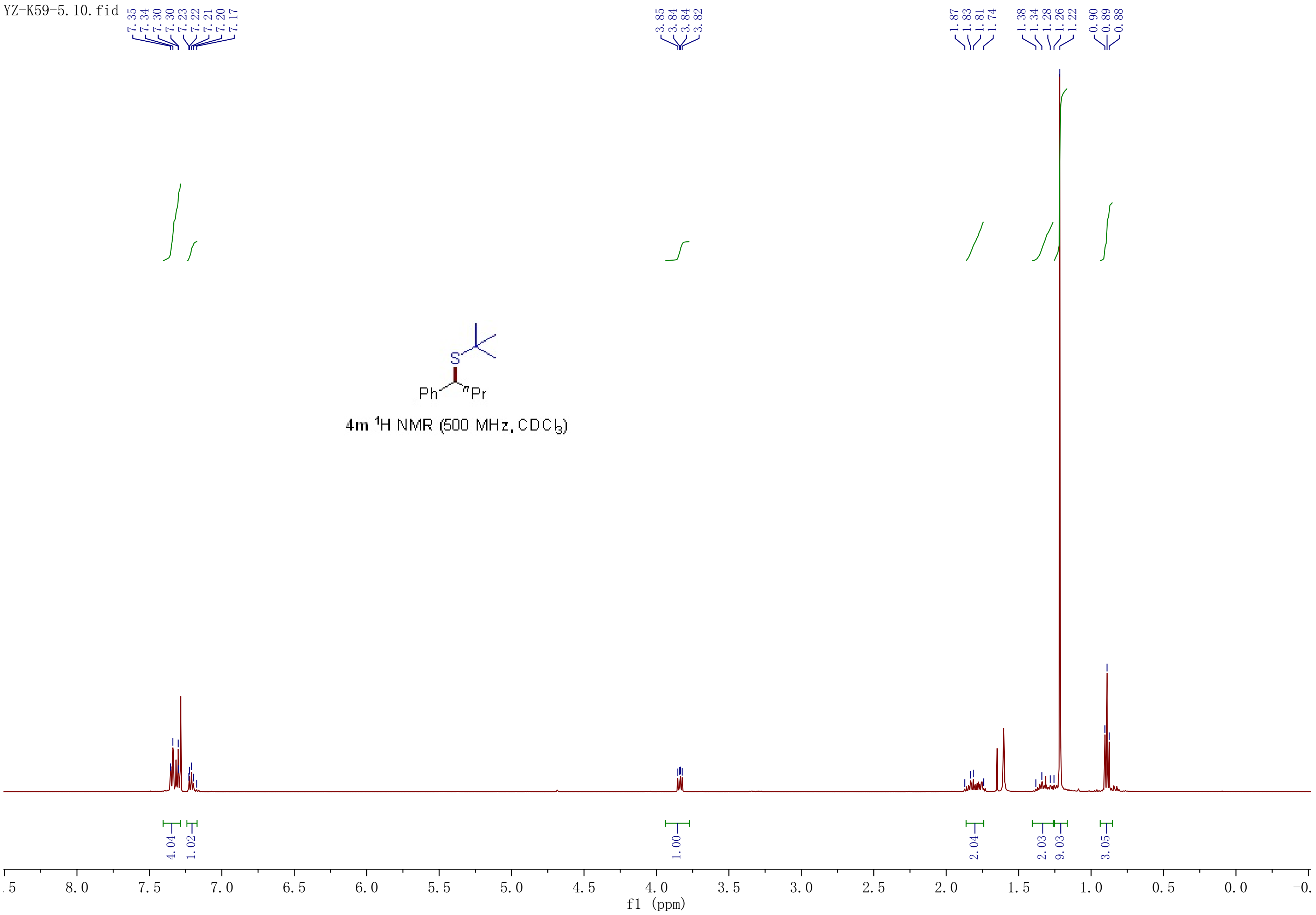

Supplementary Figure 190. ^{1}H NMR (500 MHz, $CDCl_3$) spectra for compound **4m**

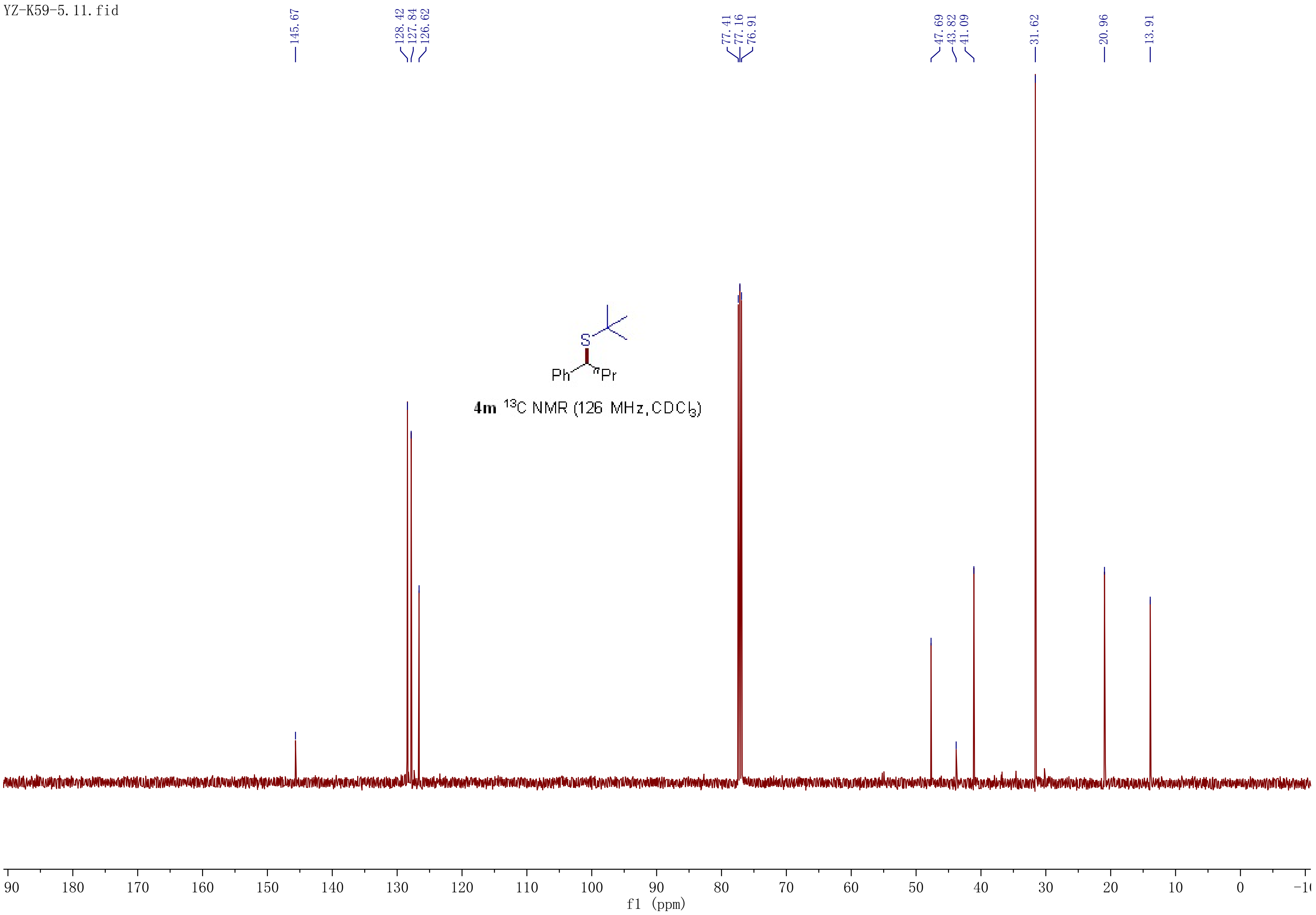

Supplementary Figure 191 ^{13}C NMR (126 MHz, $CDCl_3$) spectra for compound **4m**

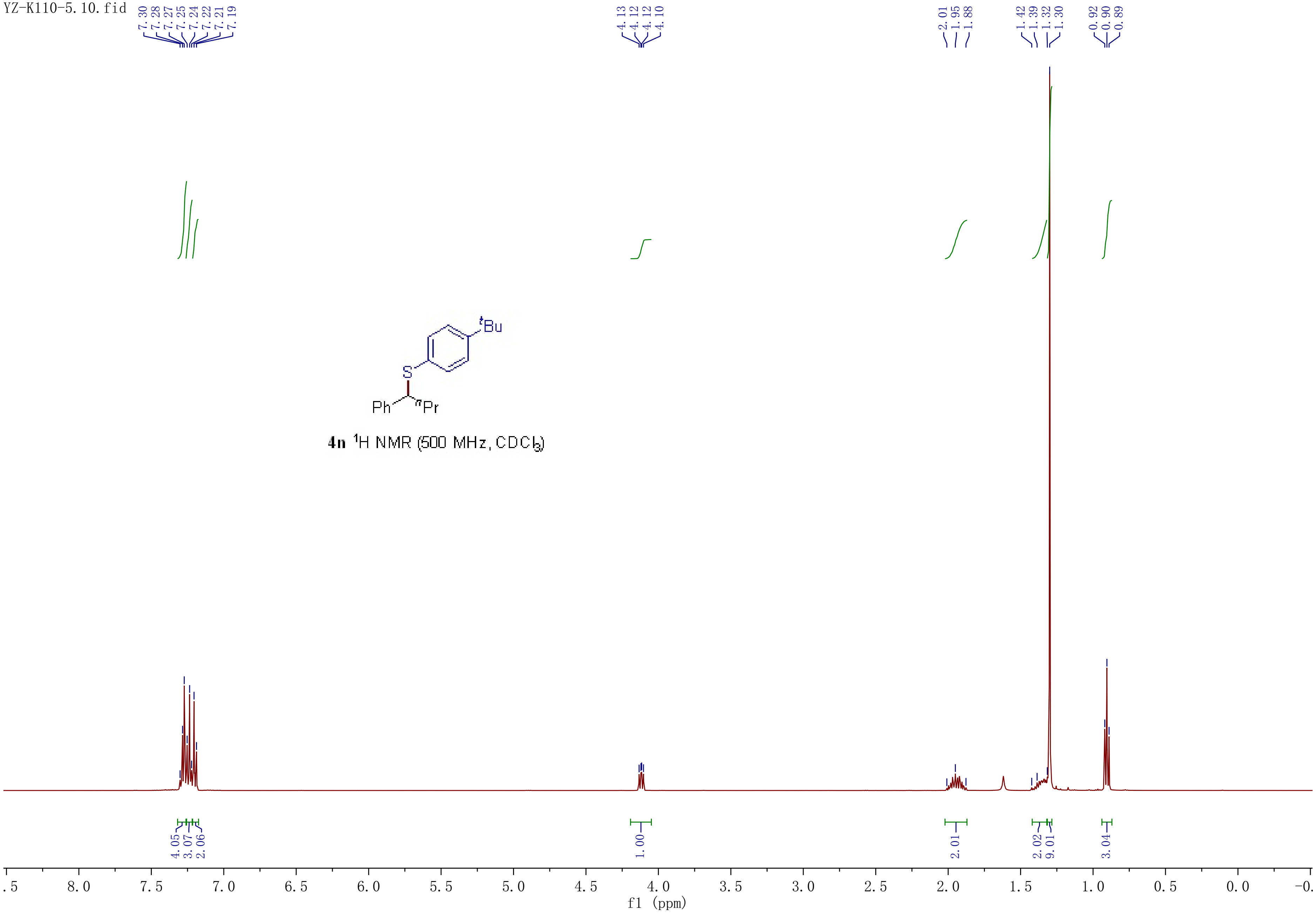

Supplementary Figure 192. ^{1}H NMR (500 MHz, $CDCl_3$) spectra for compound **4n**

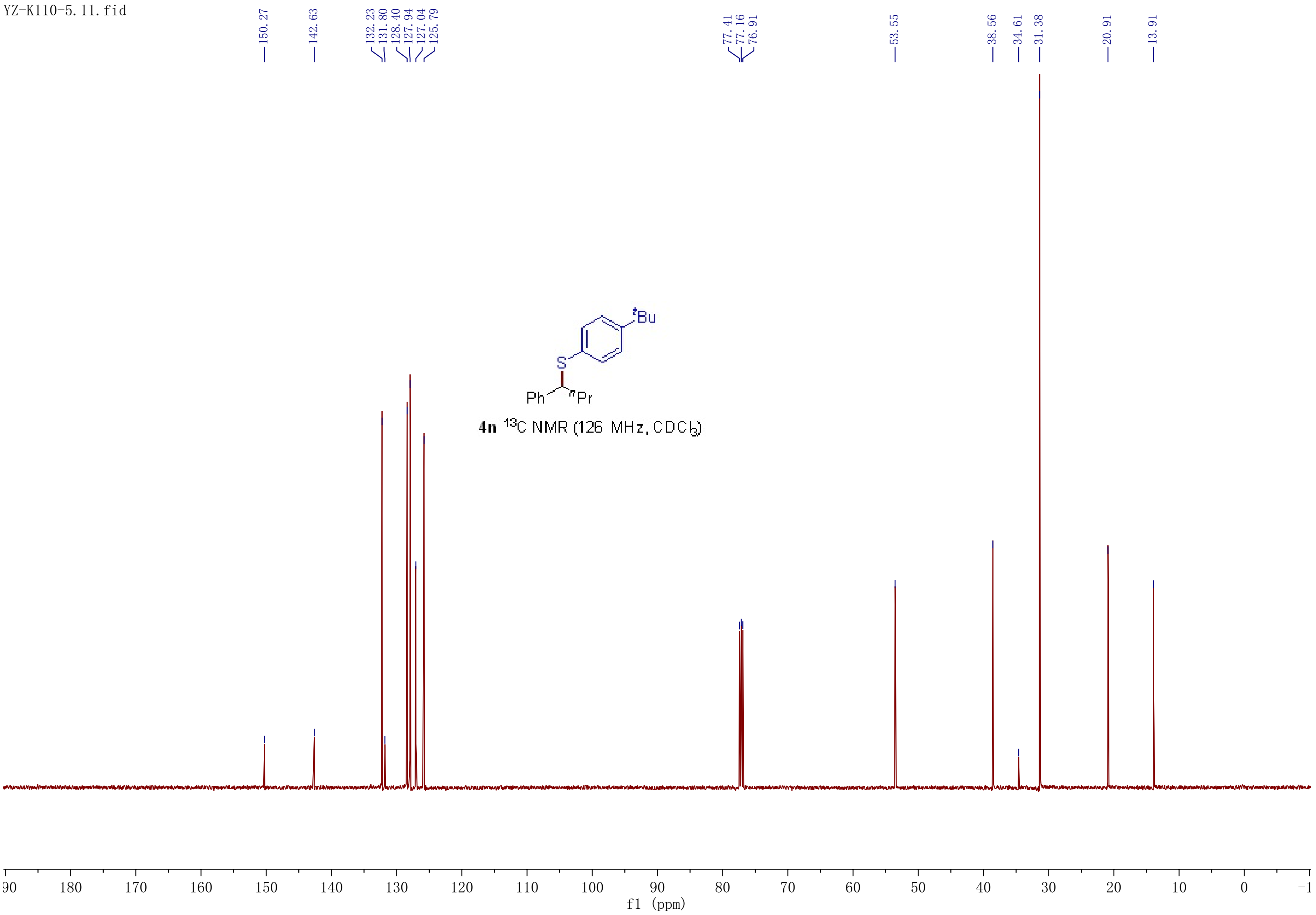

Supplementary Figure 193. ^{13}C NMR (126 MHz, $CDCl_3$) spectra for compound **4n**

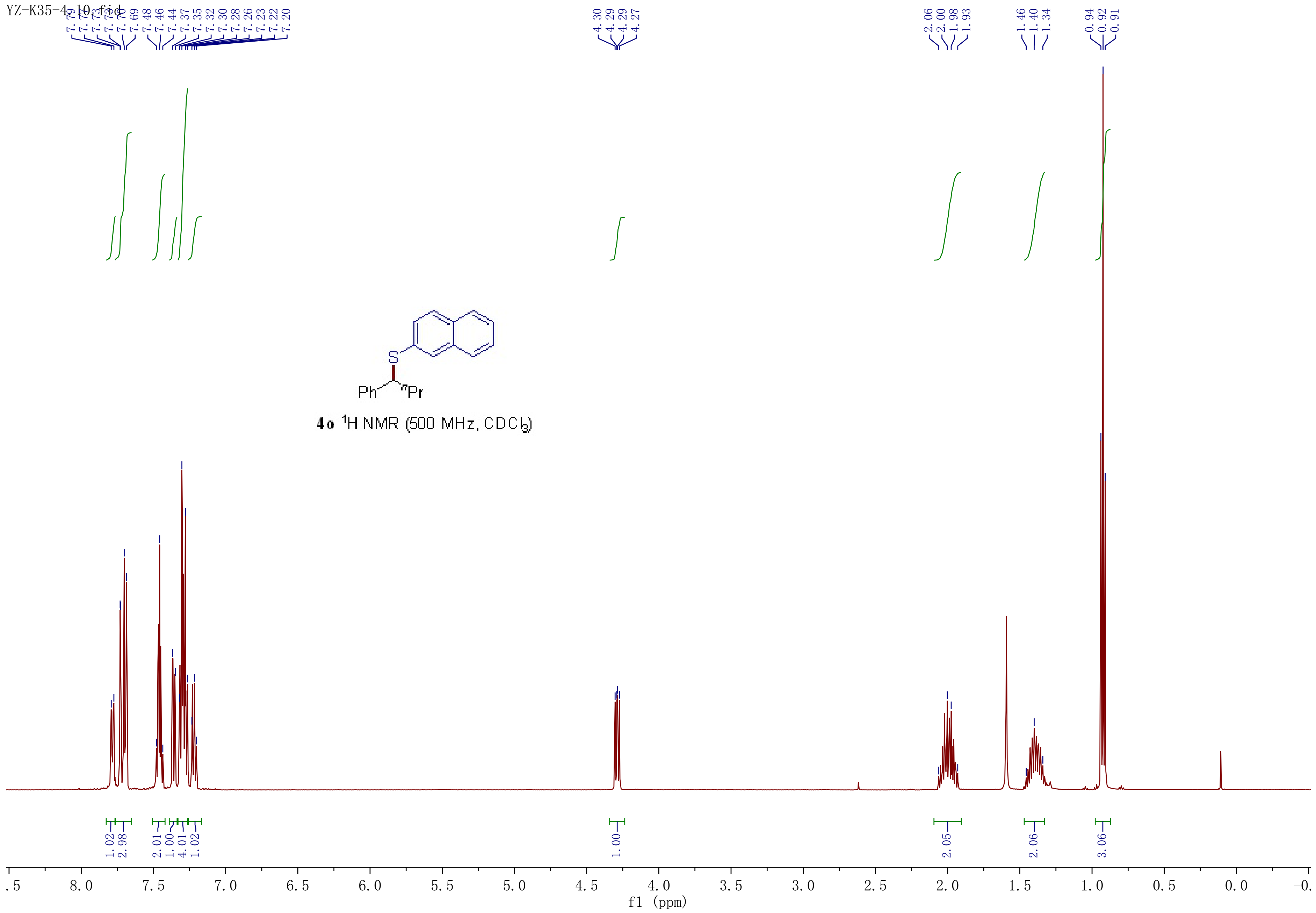

Supplementary Figure 194. ^{1}H NMR (500 MHz, $CDCl_3$) spectra for compound **4o**

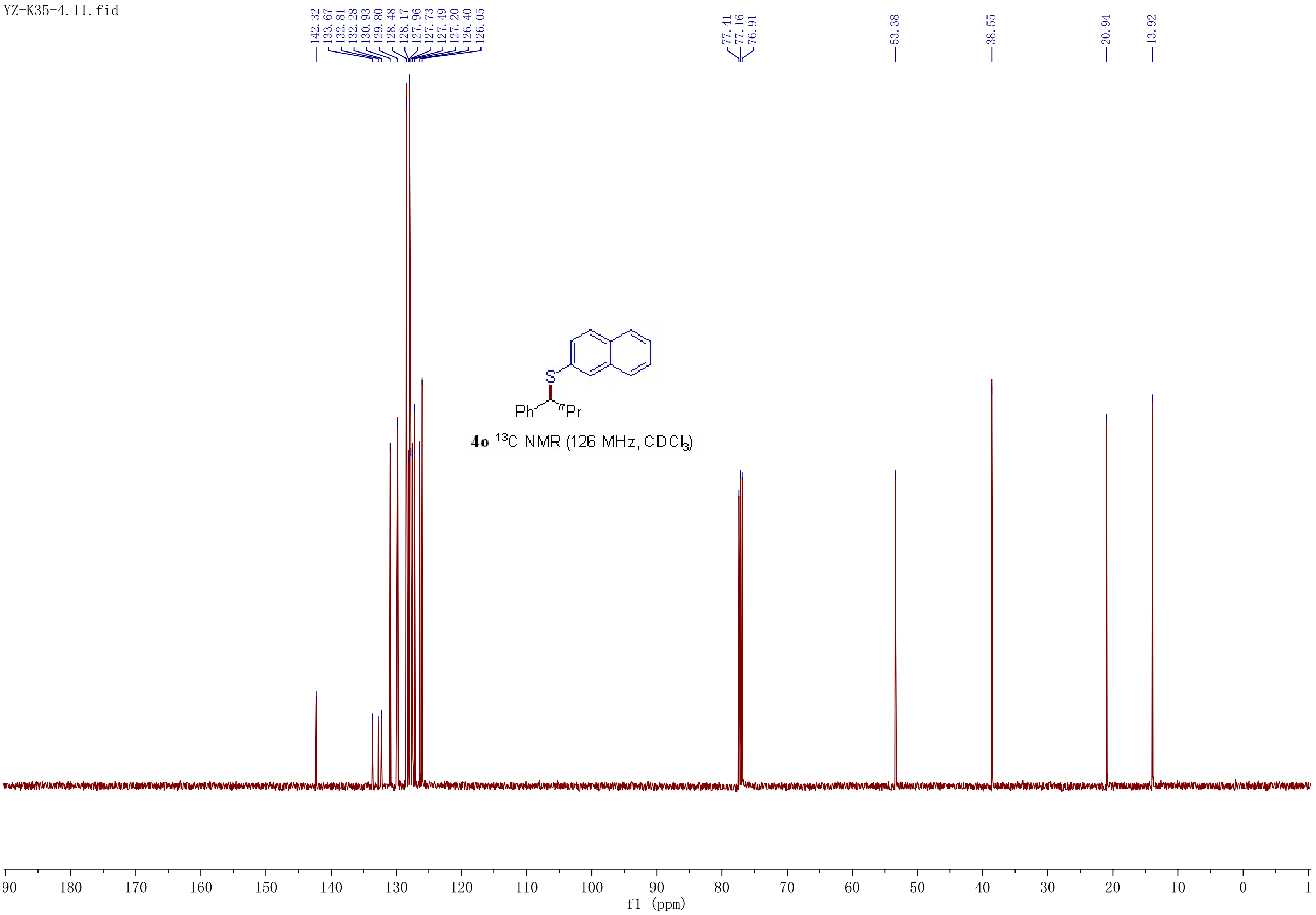

Supplementary Figure 195. ^{13}C NMR (126 MHz, $CDCl_3$) spectra for compound **4o**

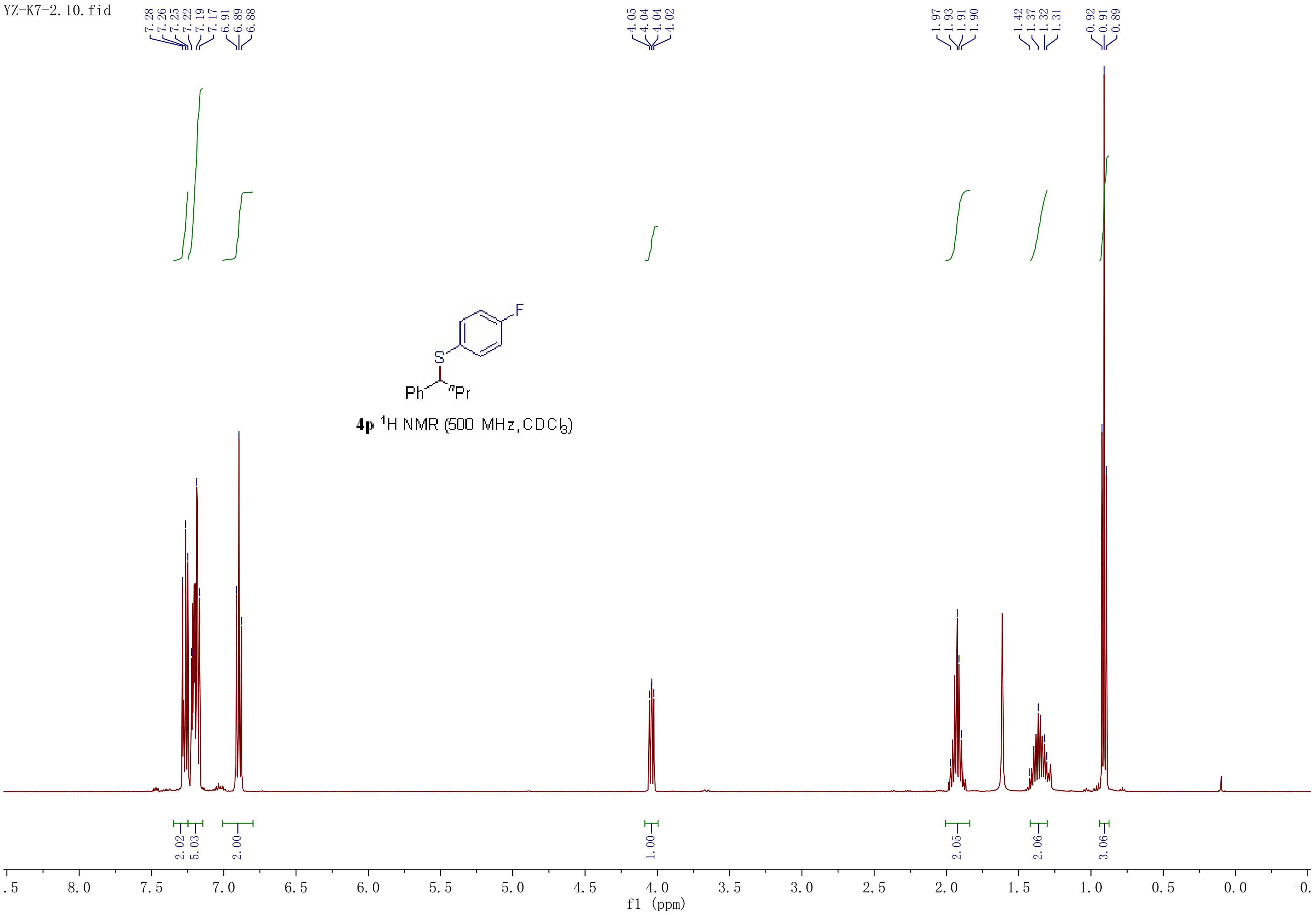

Supplementary Figure 196. ^{1}H NMR (500 MHz, $CDCl_3$) spectra for compound **4p**

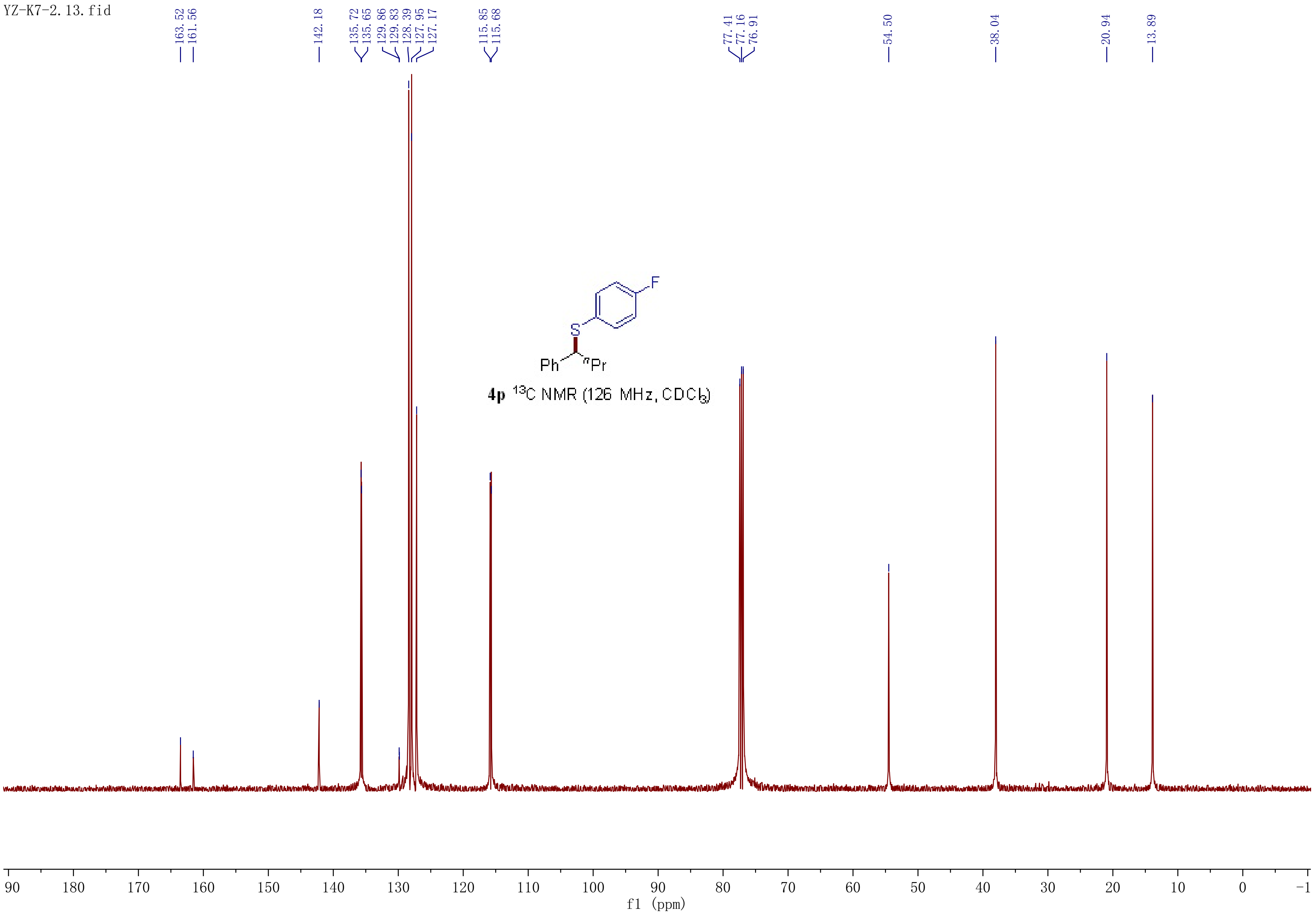

Supplementary Figure 197. ^{13}C NMR (126 MHz, $CDCl_3$) spectra for compound **4p**

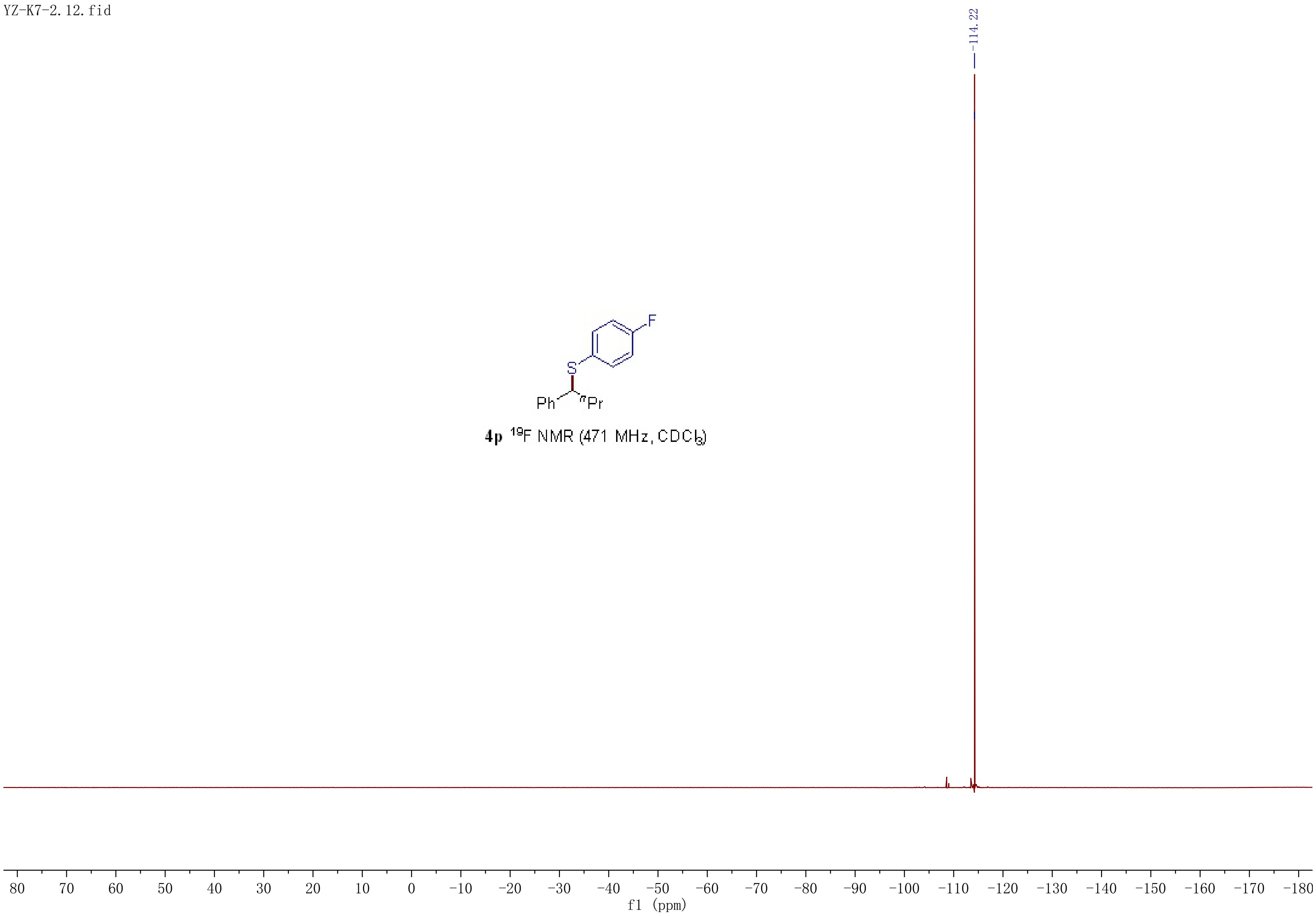

Supplementary Figure 198. ^{19}F NMR (471 MHz, $CDCl_3$) spectra for compound **4p**

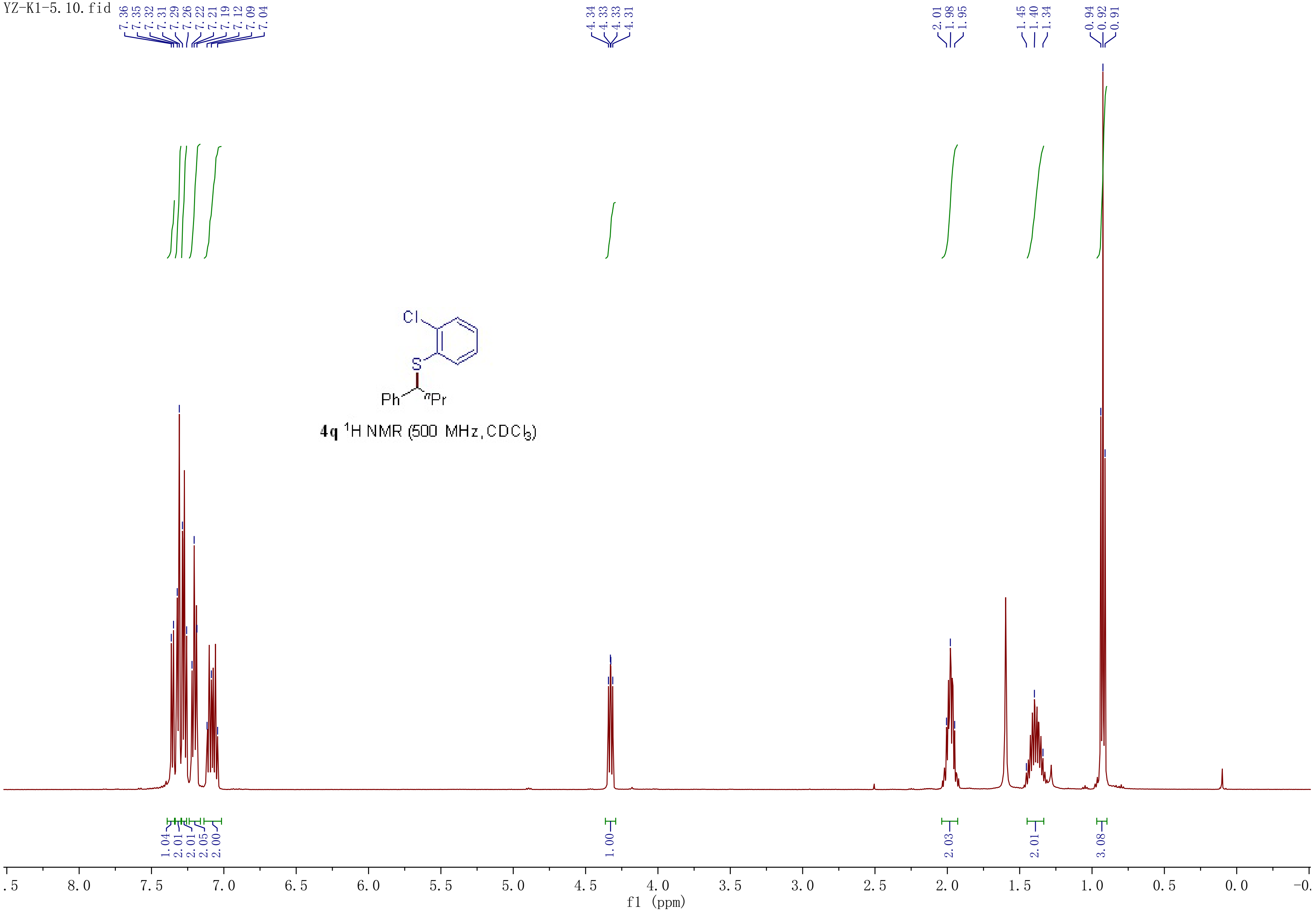

Supplementary Figure 199. ^{1}H NMR (500 MHz, $CDCl_3$) spectra for compound **4q**

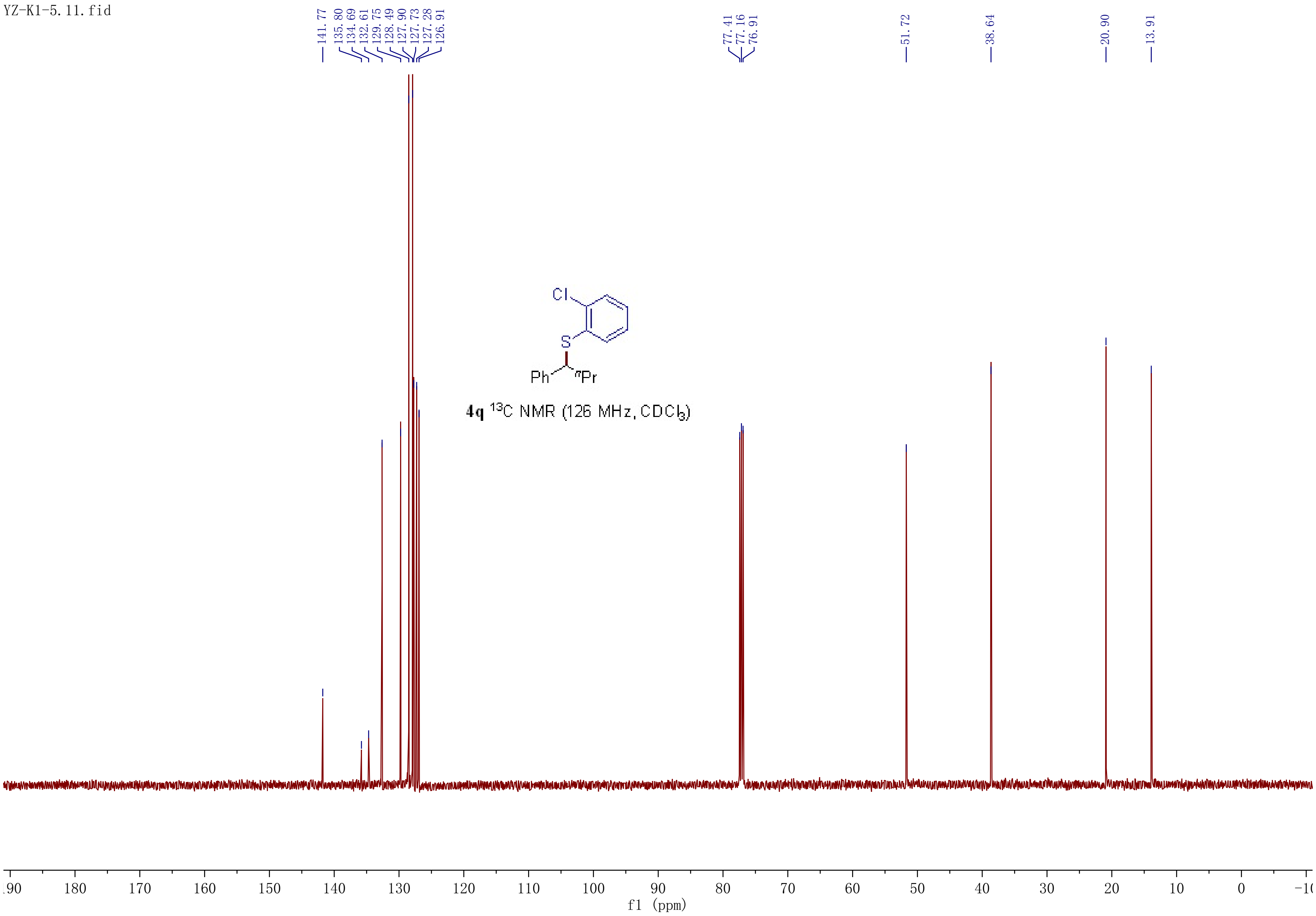

Supplementary Figure 200. ^{13}C NMR (126 MHz, $CDCl_3$) spectra for compound **4q**

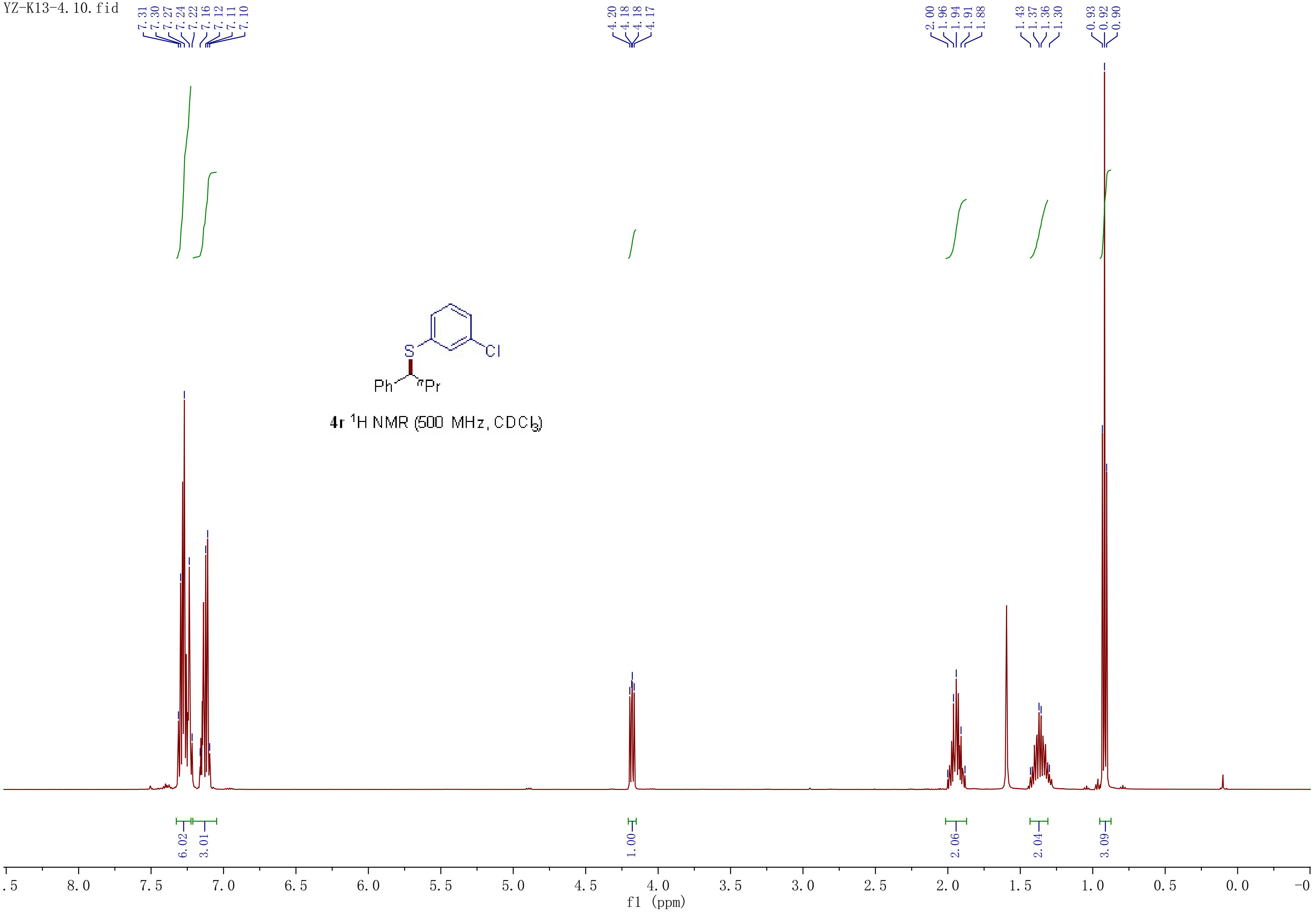

Supplementary Figure 201. ^{1}H NMR (500 MHz, $CDCl_3$) spectra for compound **4r**

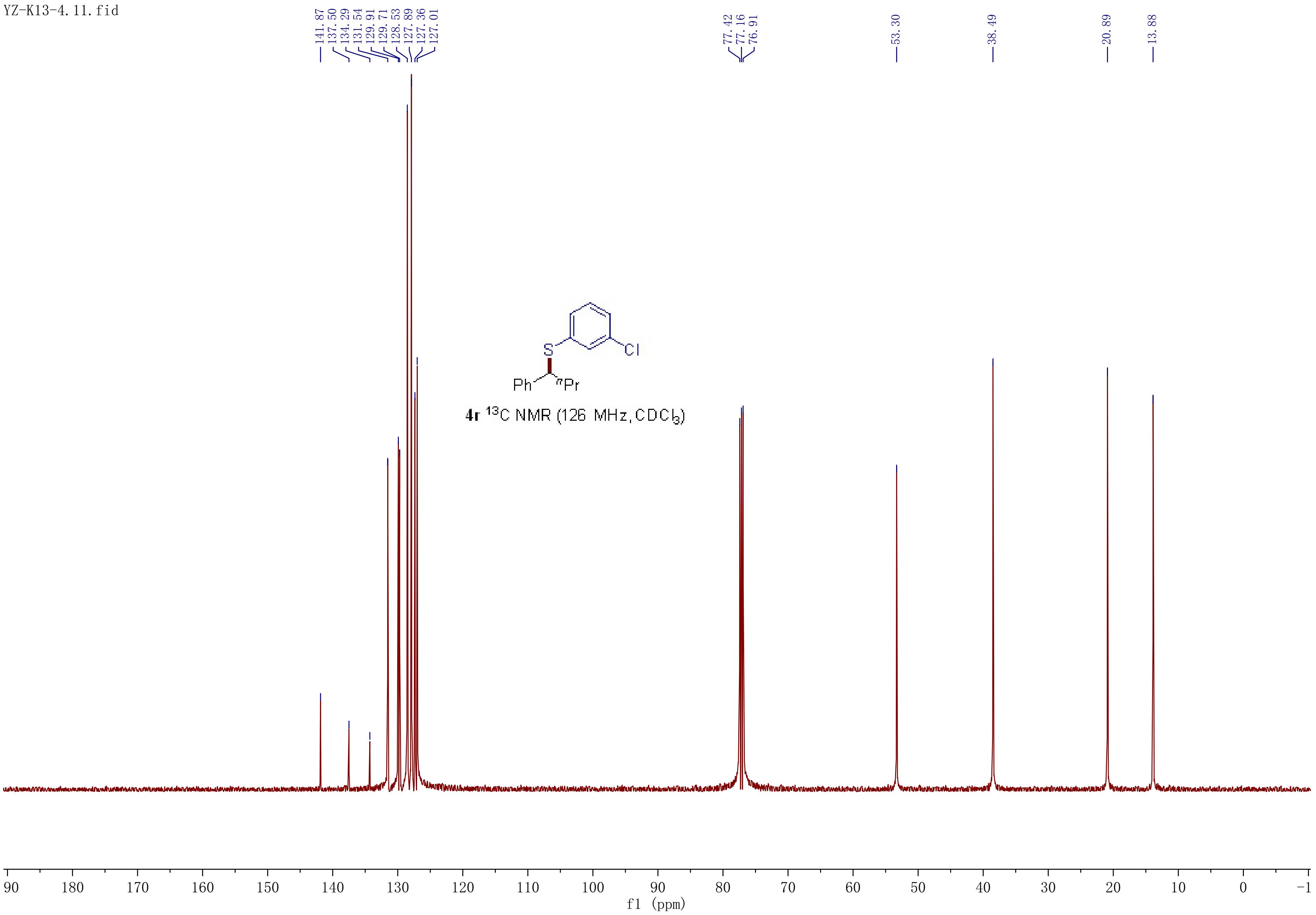

Supplementary Figure 202. ^{13}C NMR (126 MHz, $CDCl_3$) spectra for compound **4r**

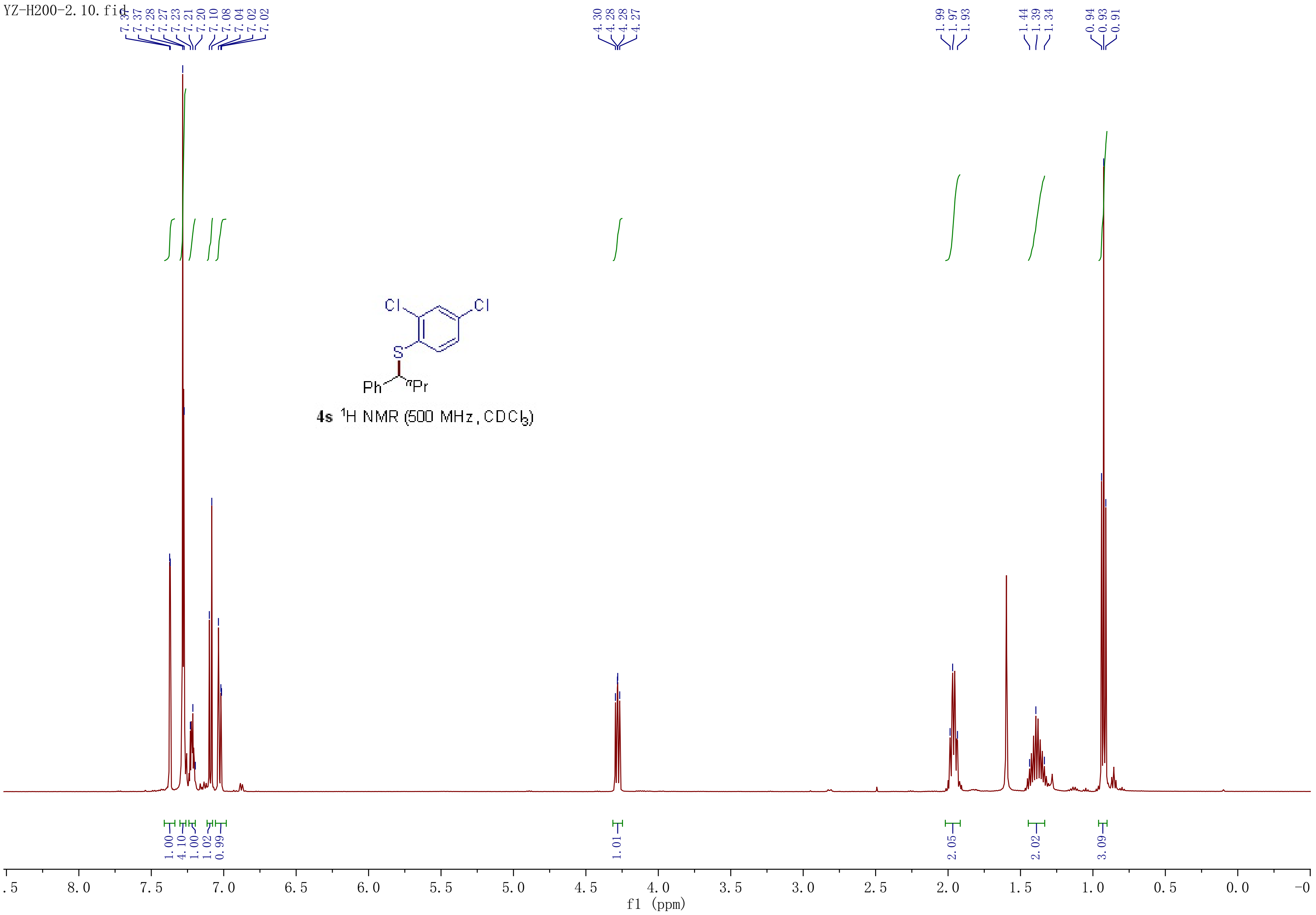

Supplementary Figure 203. ^{1}H NMR (500 MHz, $CDCl_3$) spectra for compound **4s**

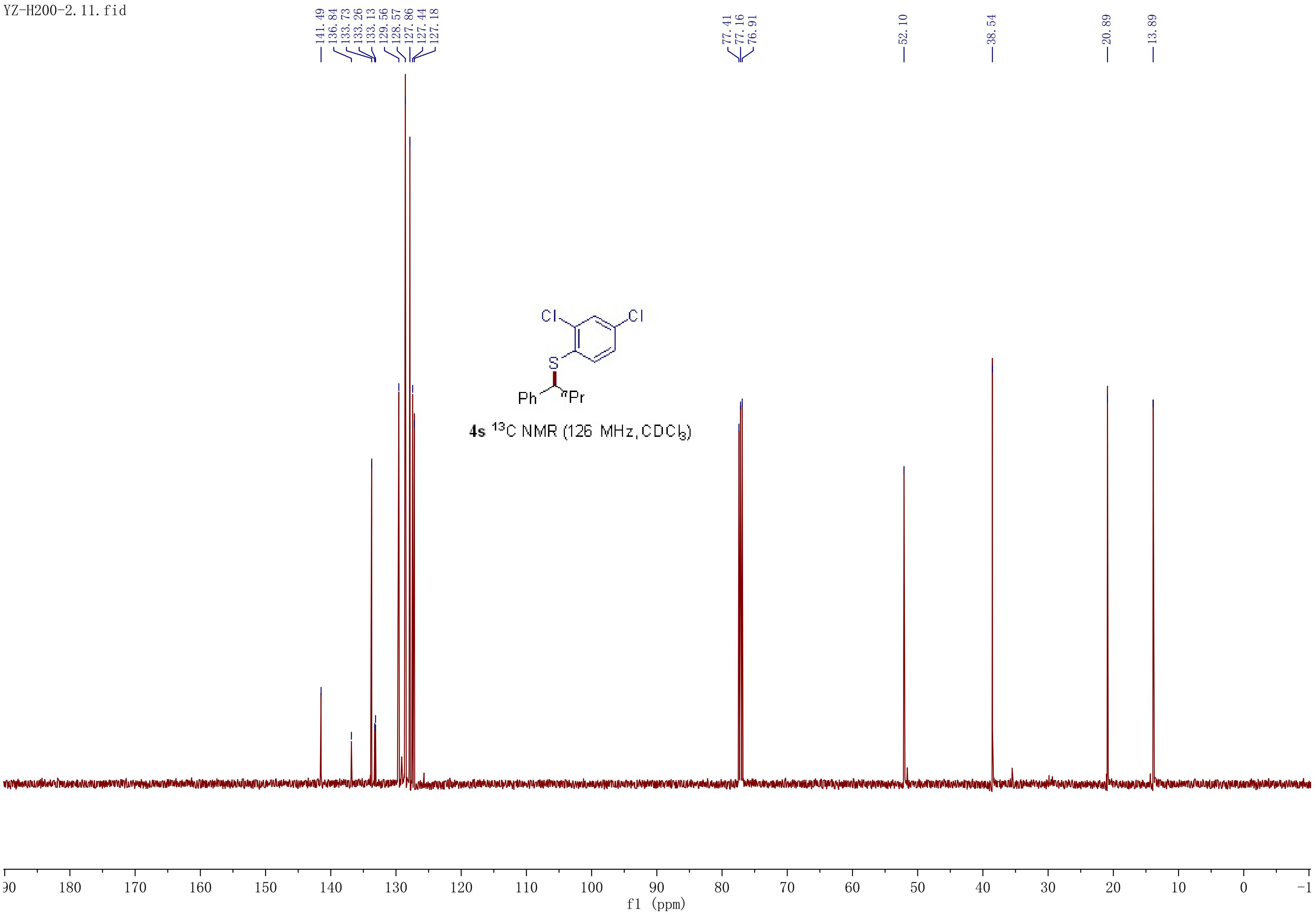

Supplementary Figure 204. ^{13}C NMR (126 MHz, CDCl$_3$) spectra for compound **4s**

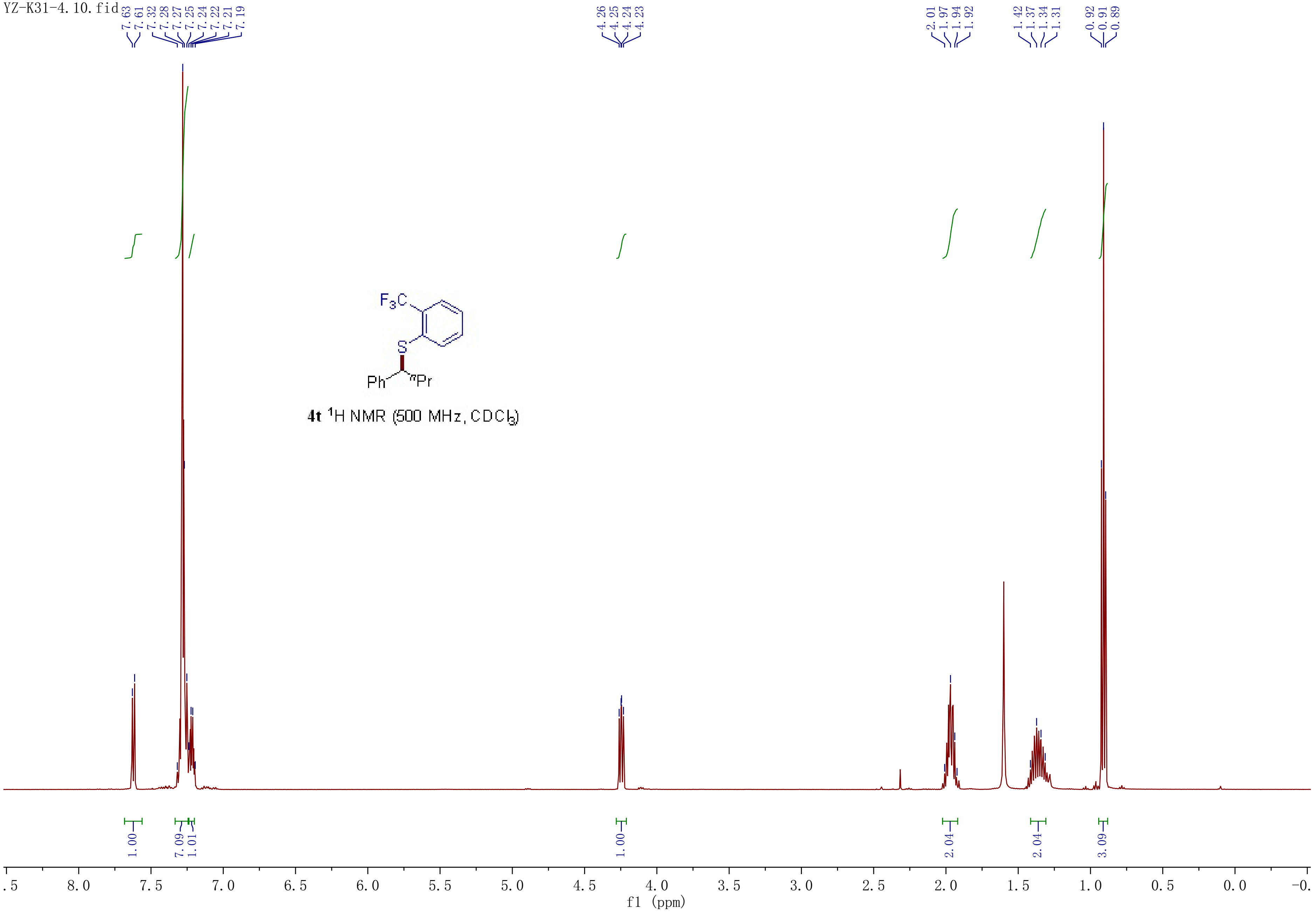

Supplementary Figure 205. ^{1}H NMR (500 MHz, $CDCl_3$) spectra for compound **4t**

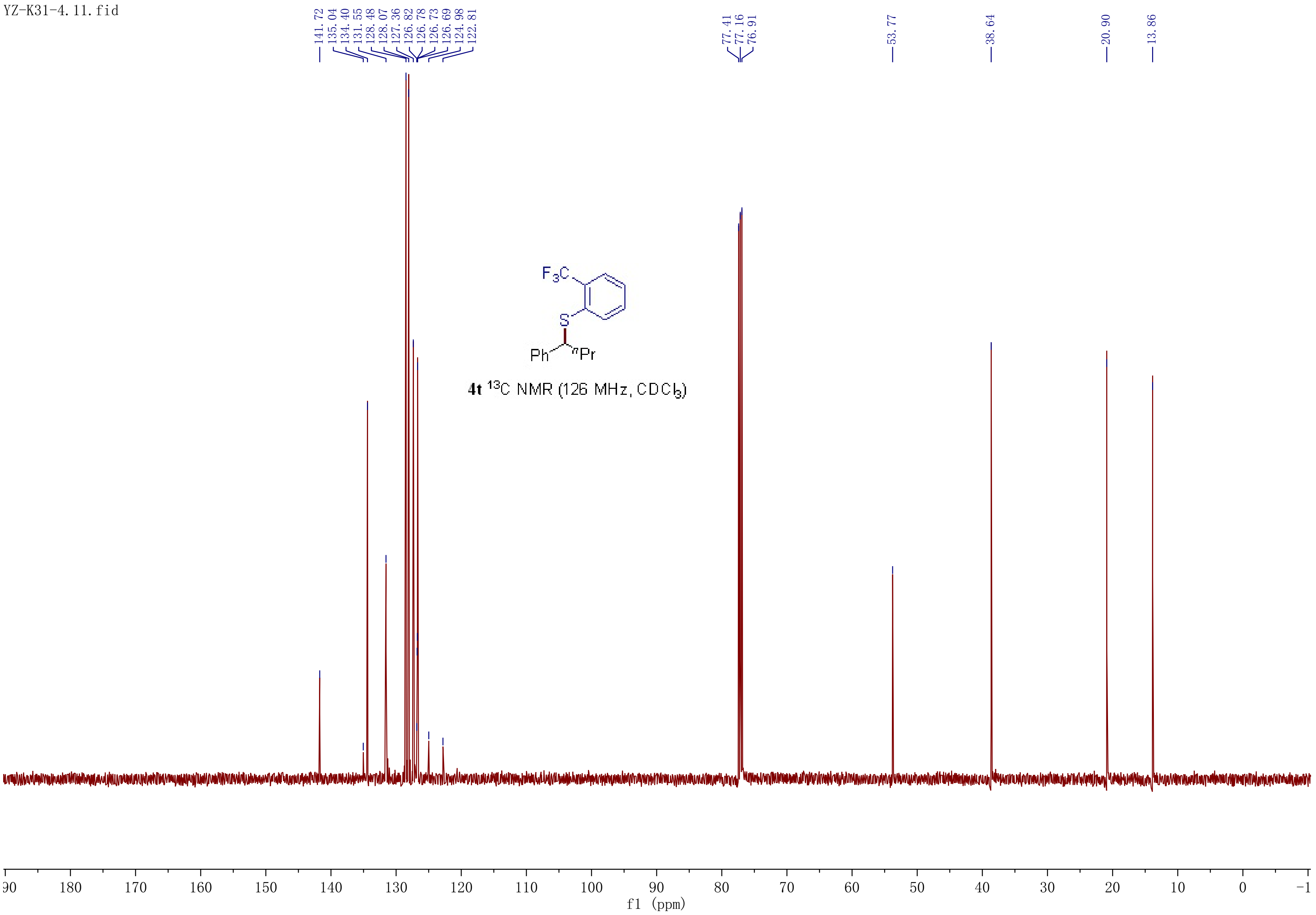

Supplementary Figure 206. ^{13}C NMR (126 MHz, $CDCl_3$) spectra for compound **4t**

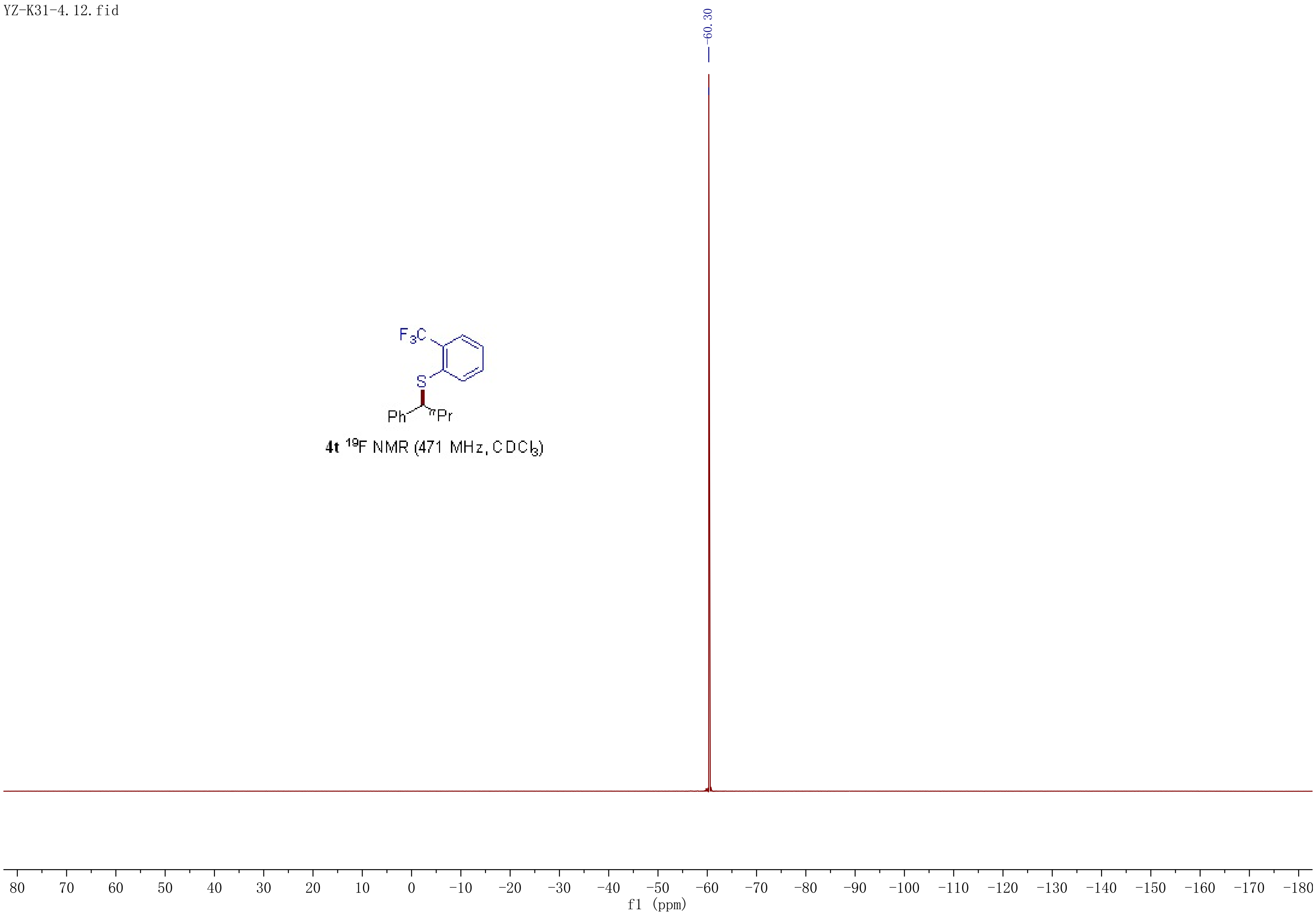

Supplementary Figure 207. ^{19}F NMR (471 MHz, $CDCl_3$) spectra for compound **4t**

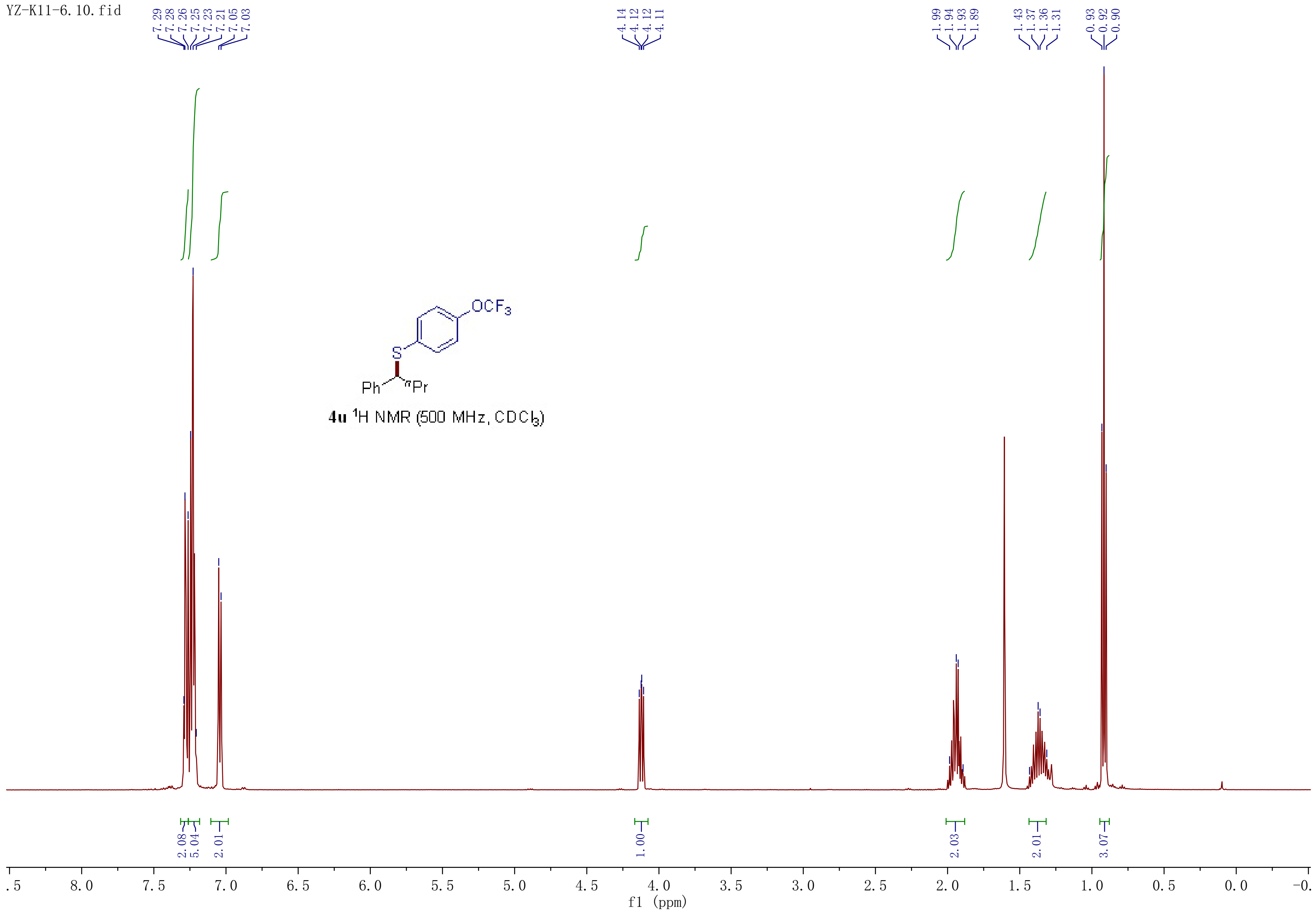

Supplementary Figure 208. ^{1}H NMR (500 MHz, $CDCl_3$) spectra for compound **4u**

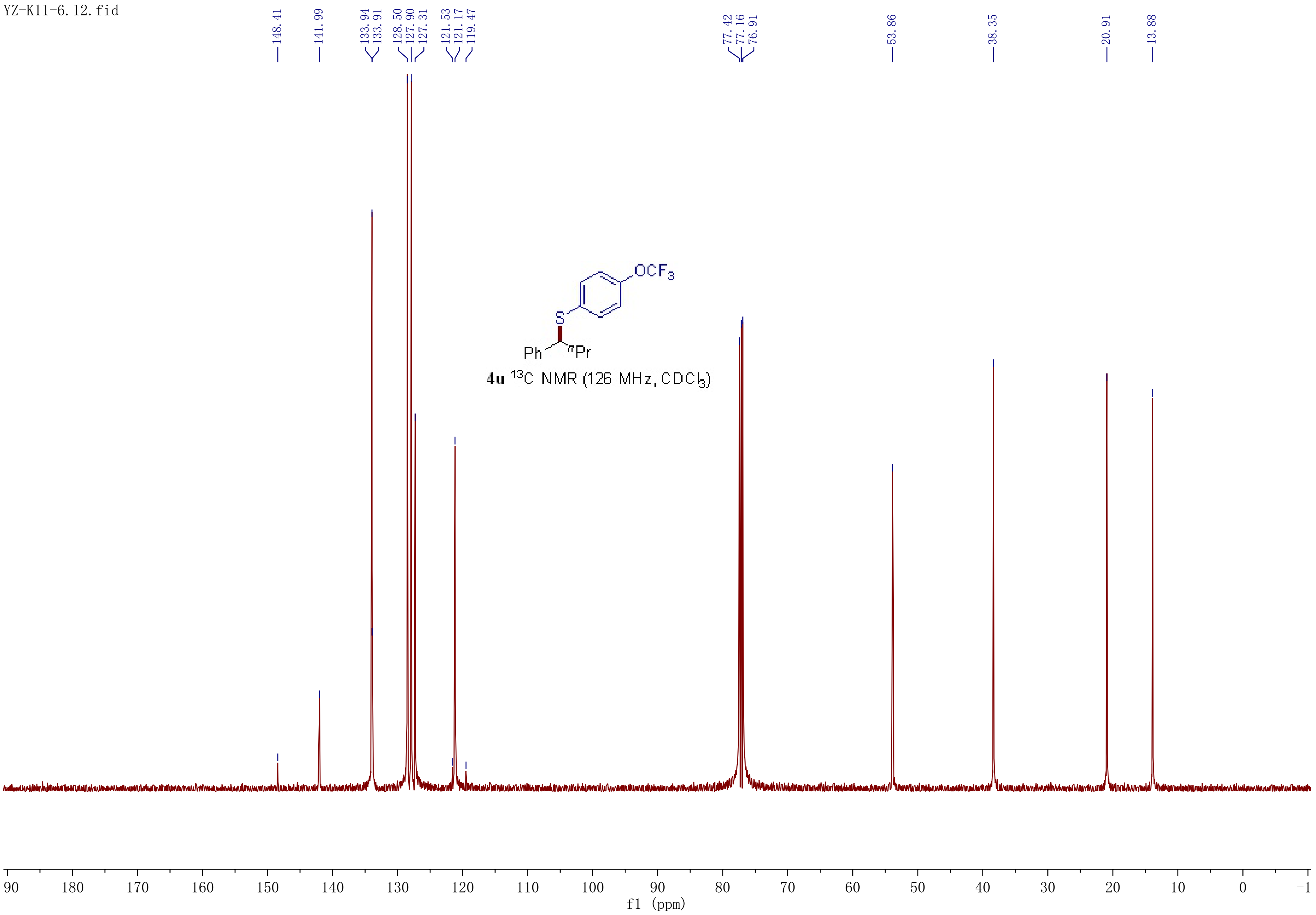

Supplementary Figure 209. ^{13}C NMR (126 MHz, $CDCl_3$) spectra for compound **4u**

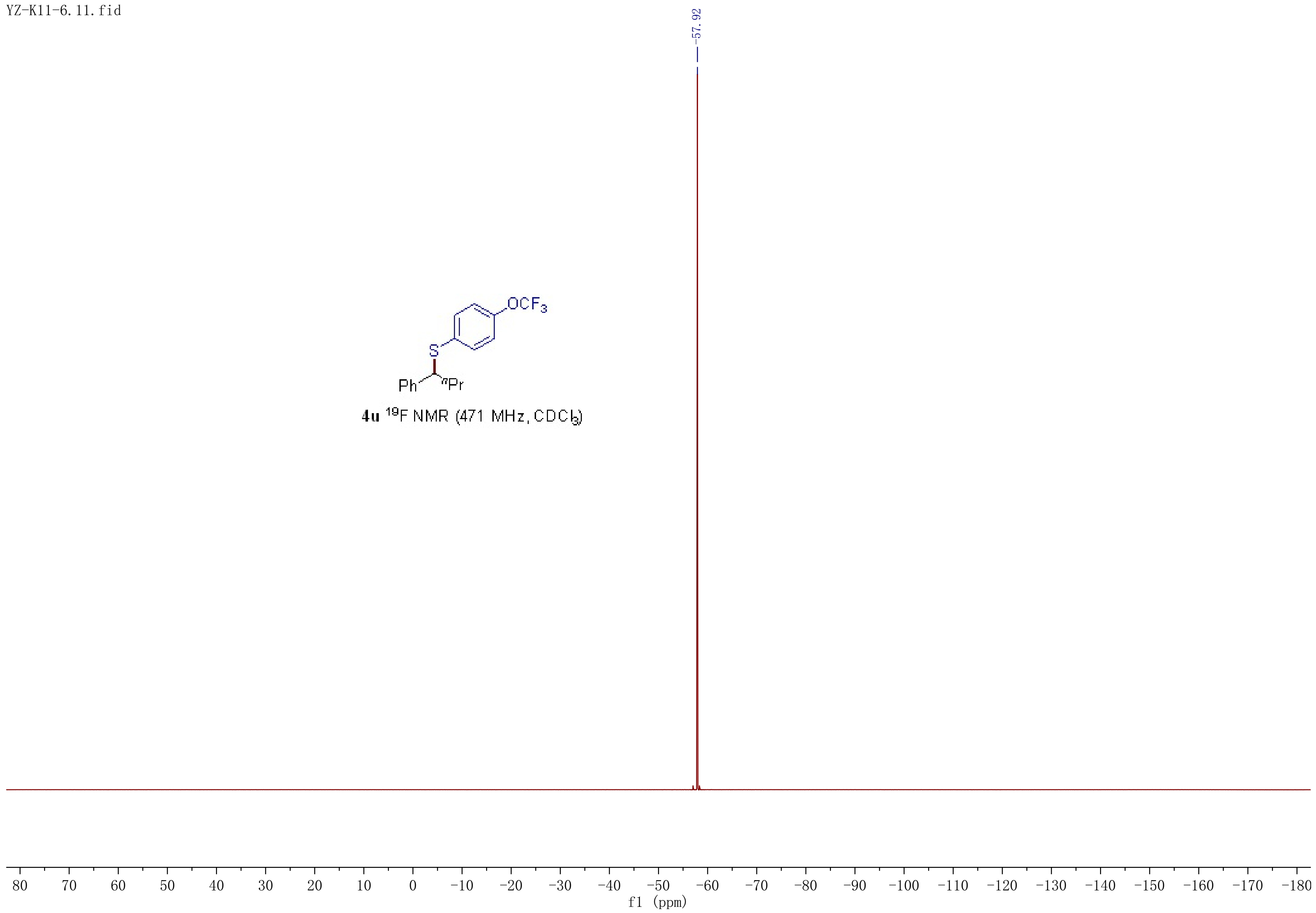

Supplementary Figure 210. ^{19}F NMR (471 MHz, $CDCl_3$) spectra for compound **4u**

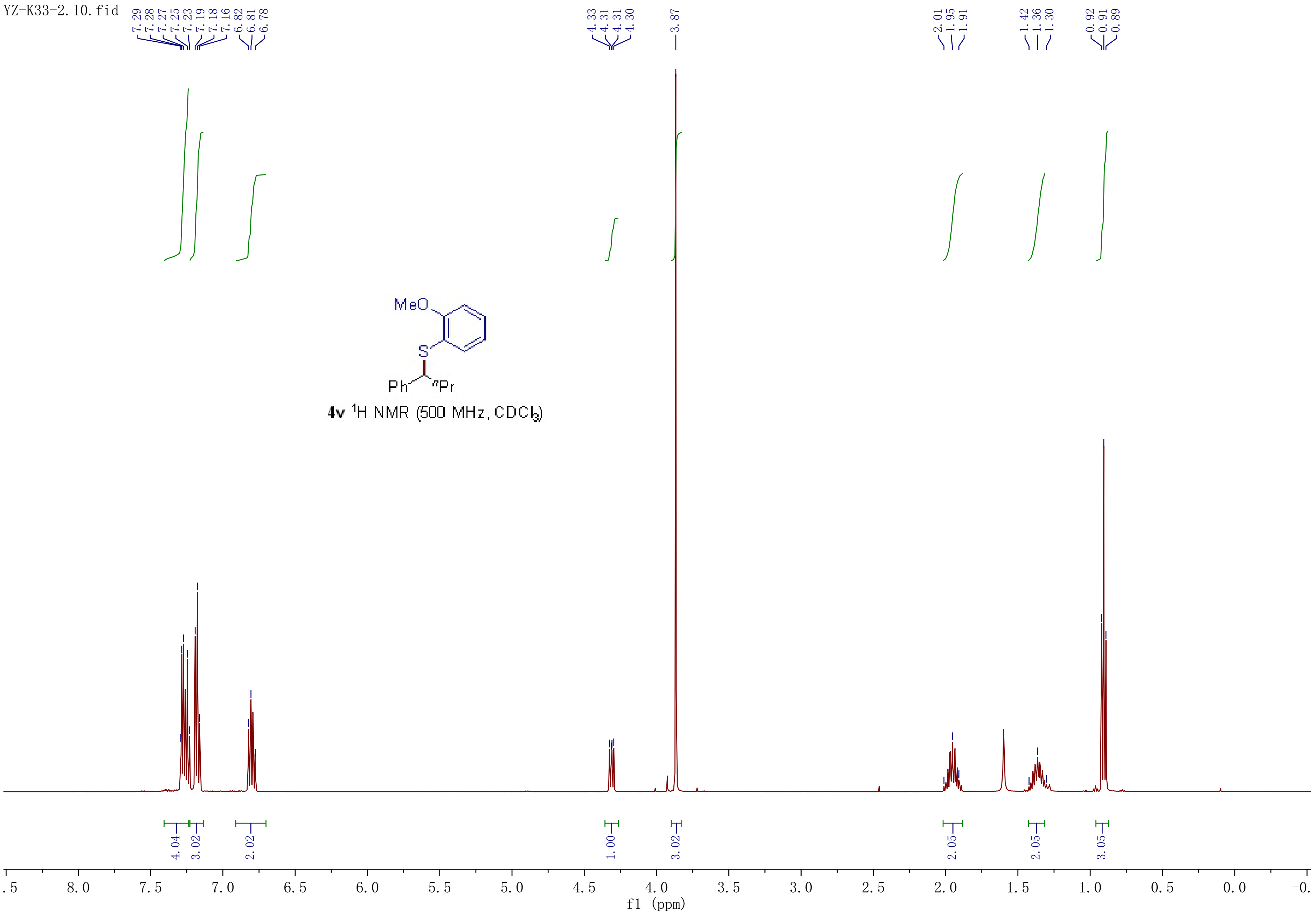

Supplementary Figure 211. ^{1}H NMR (500 MHz, $CDCl_3$) spectra for compound **4v**

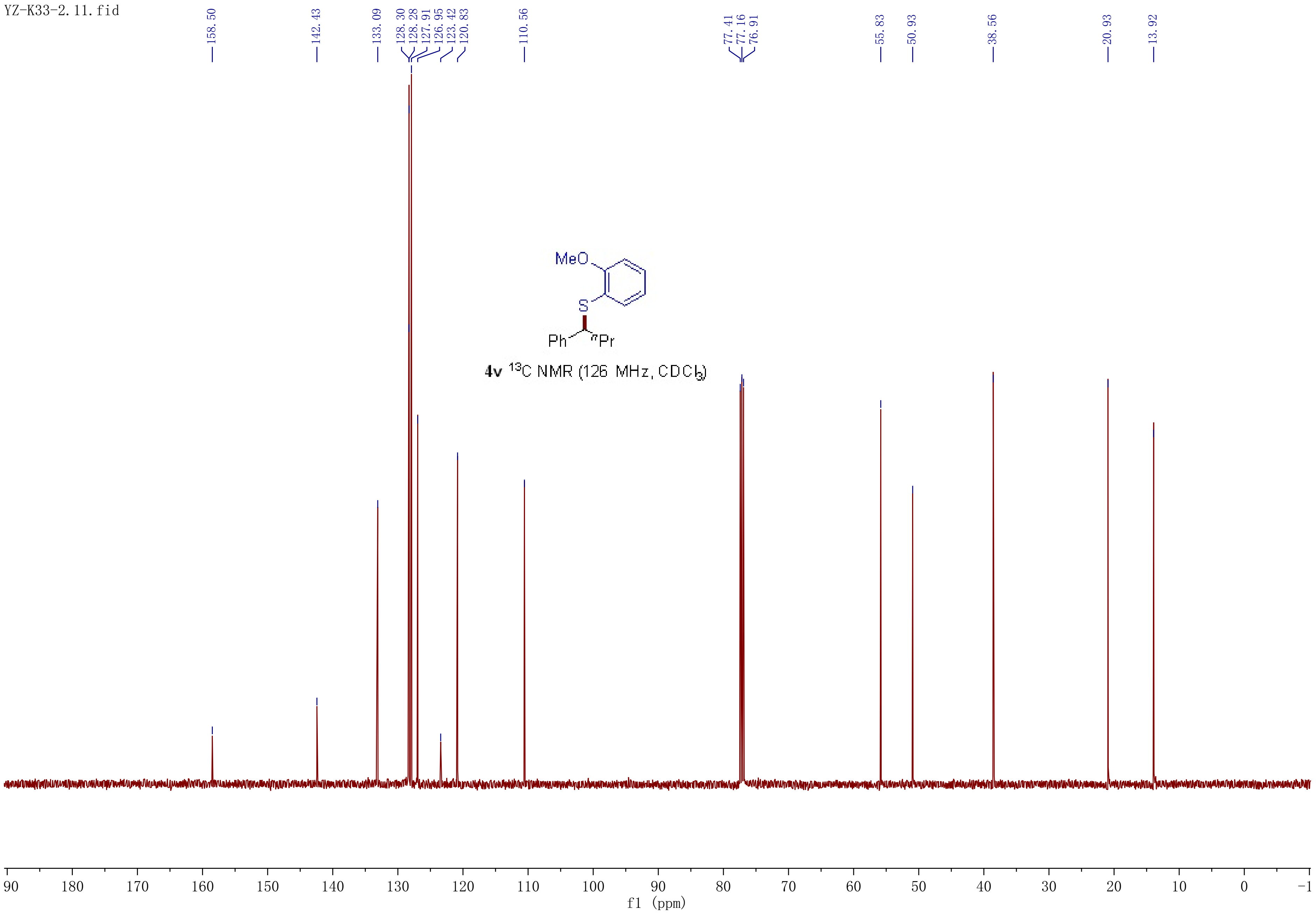

Supplementary Figure 212. ^{13}C NMR (126 MHz, $CDCl_3$) spectra for compound **4v**

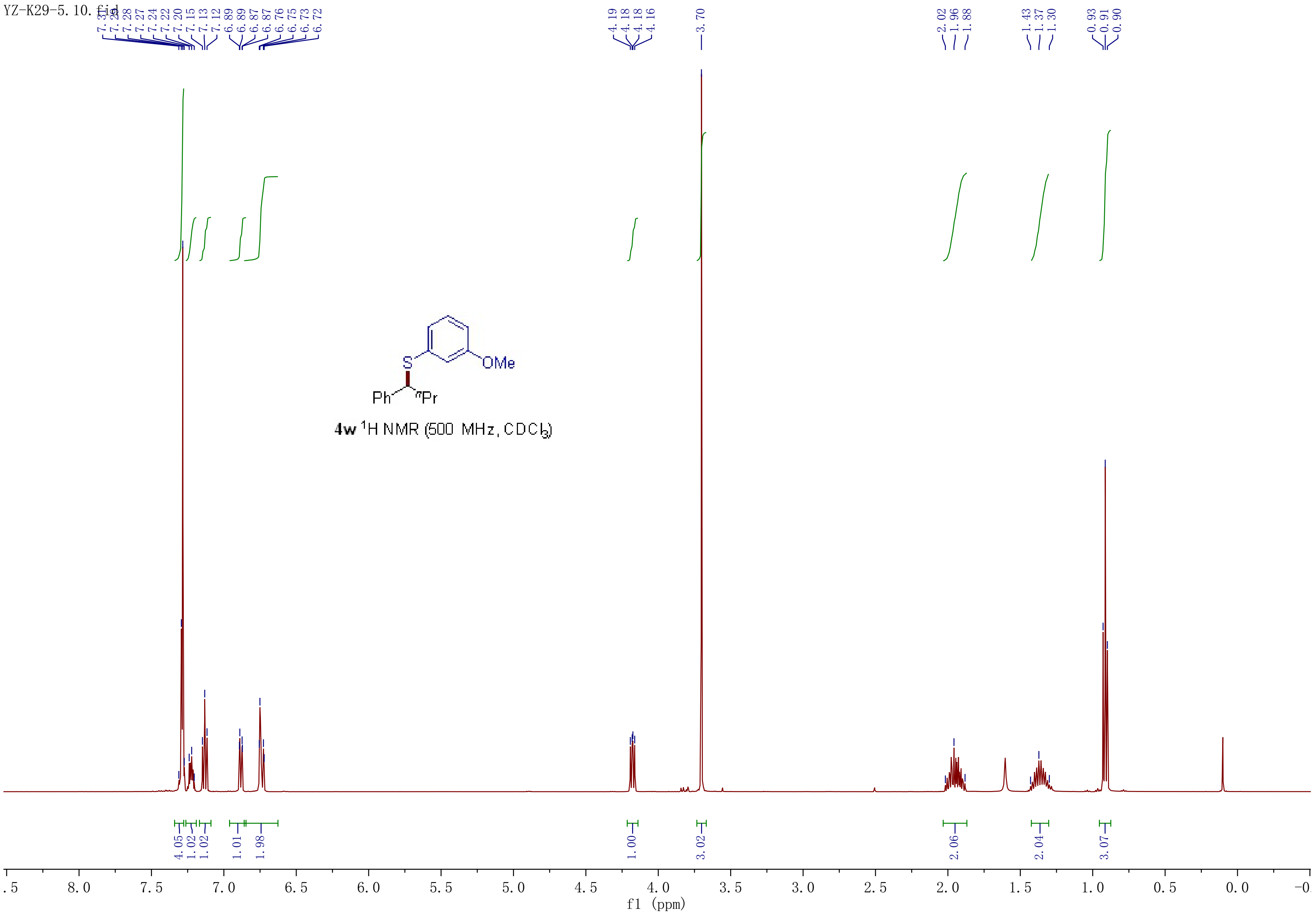

Supplementary Figure 213. ¹H NMR (500 MHz, $CDCl_3$) spectra for compound **4w**

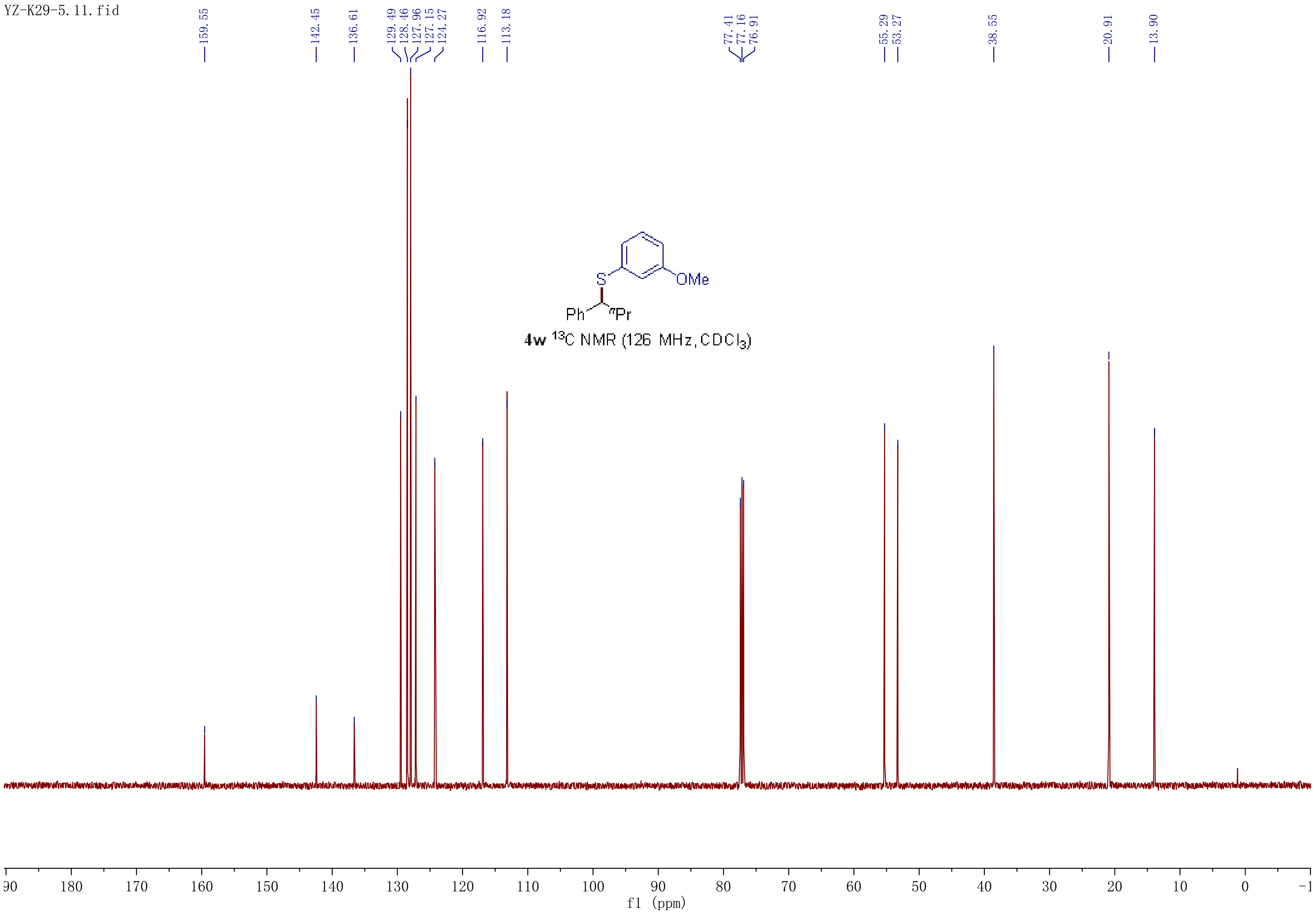

Supplementary Figure 214. ^{13}C NMR (126 MHz, $CDCl_3$) spectra for compound **4w**

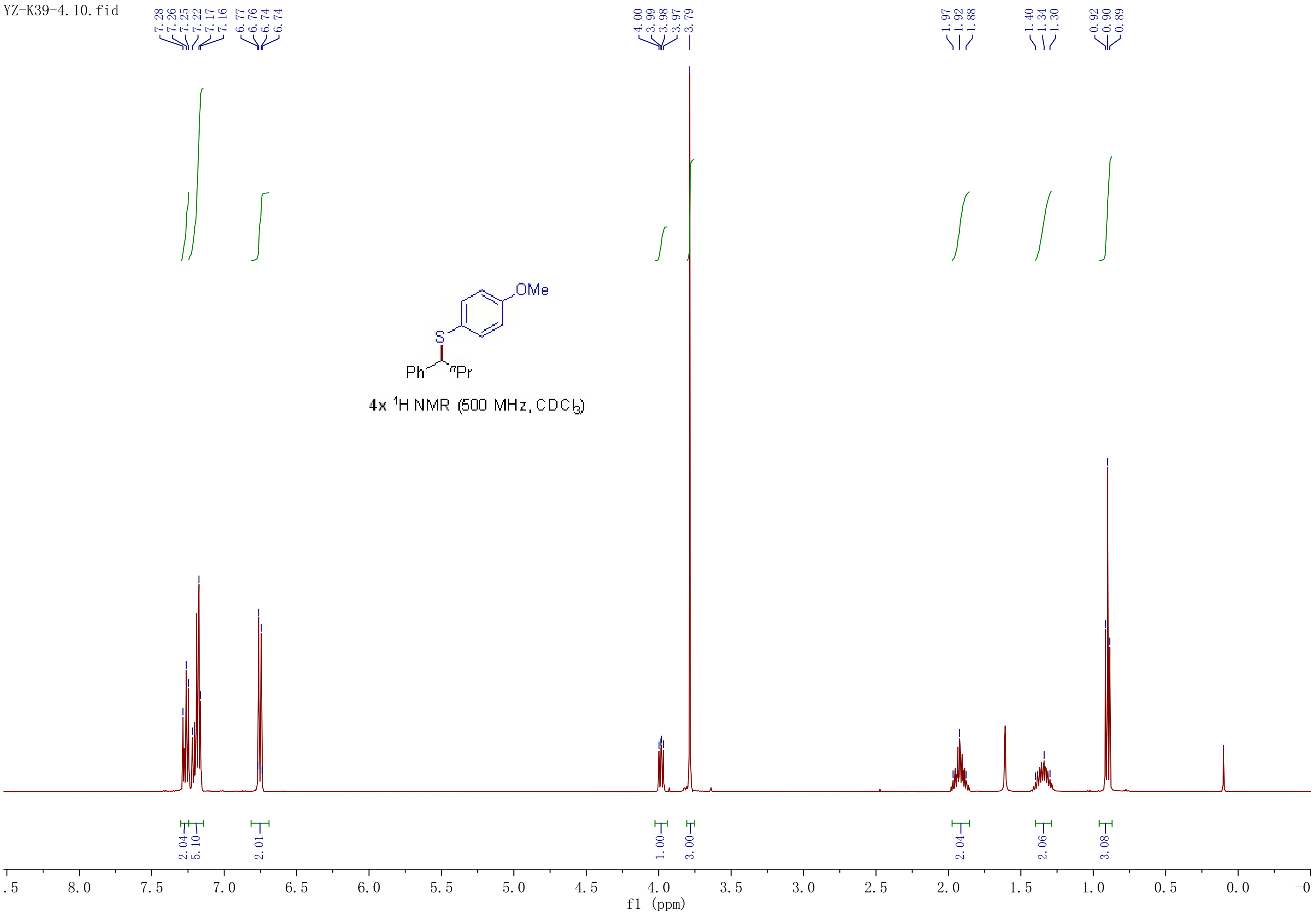

Supplementary Figure 215. ^{1}H NMR (500 MHz, $CDCl_3$) spectra for compound **4x**

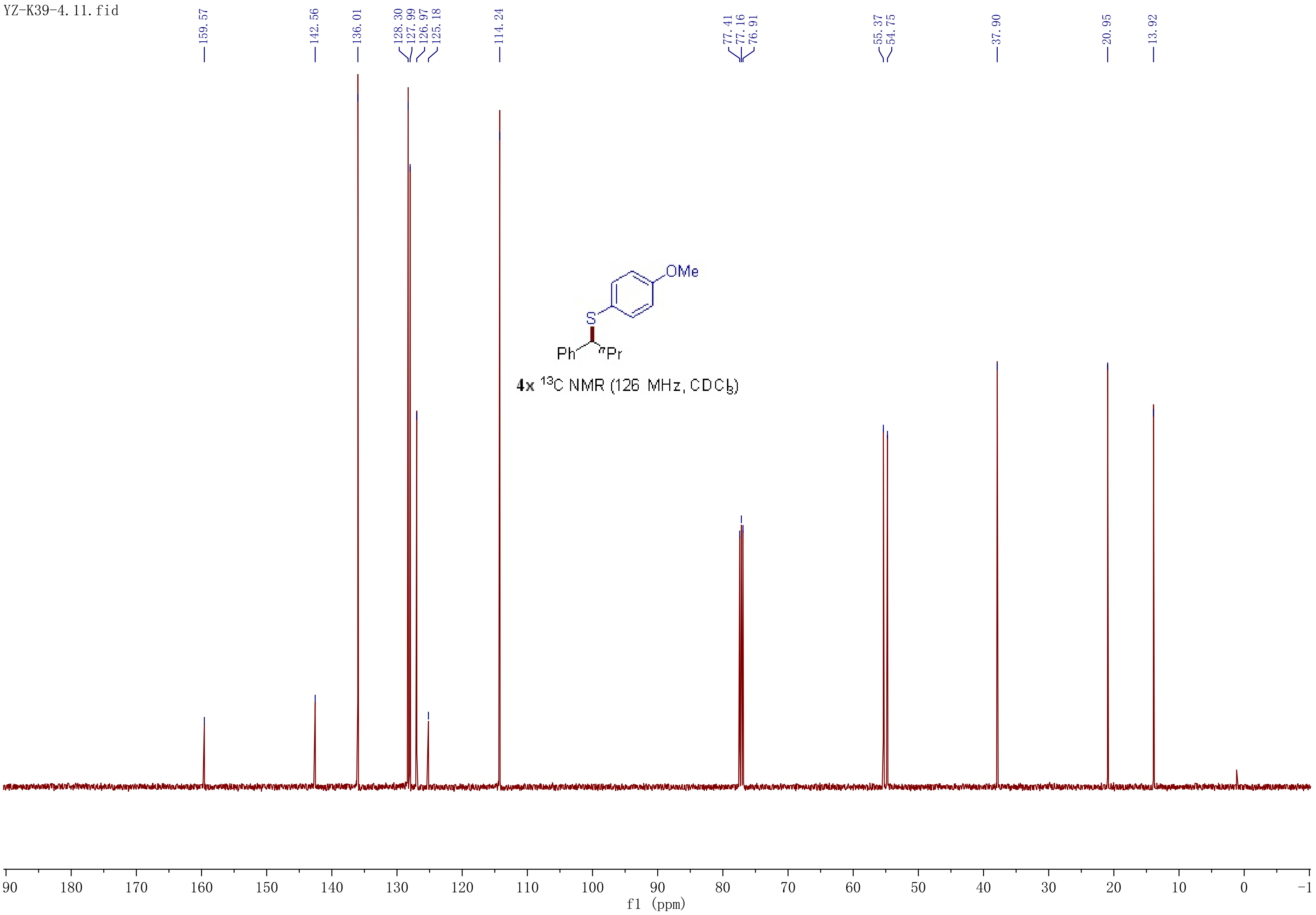

Supplementary Figure 216. ^{13}C NMR (126 MHz, $CDCl_3$) spectra for compound **4x**

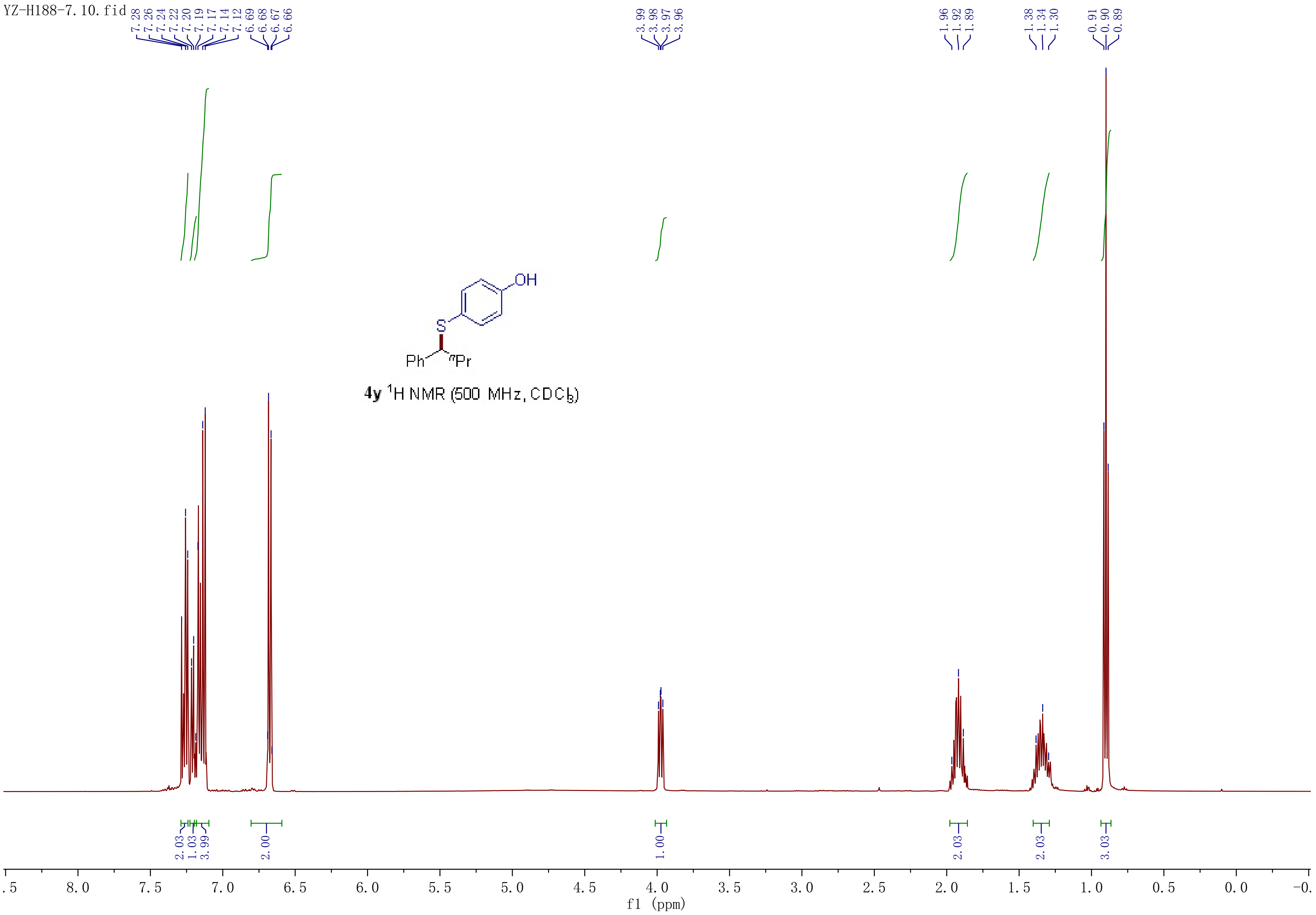

Supplementary Figure 217. ^{1}H NMR (500 MHz, $CDCl_3$) spectra for compound **4y**

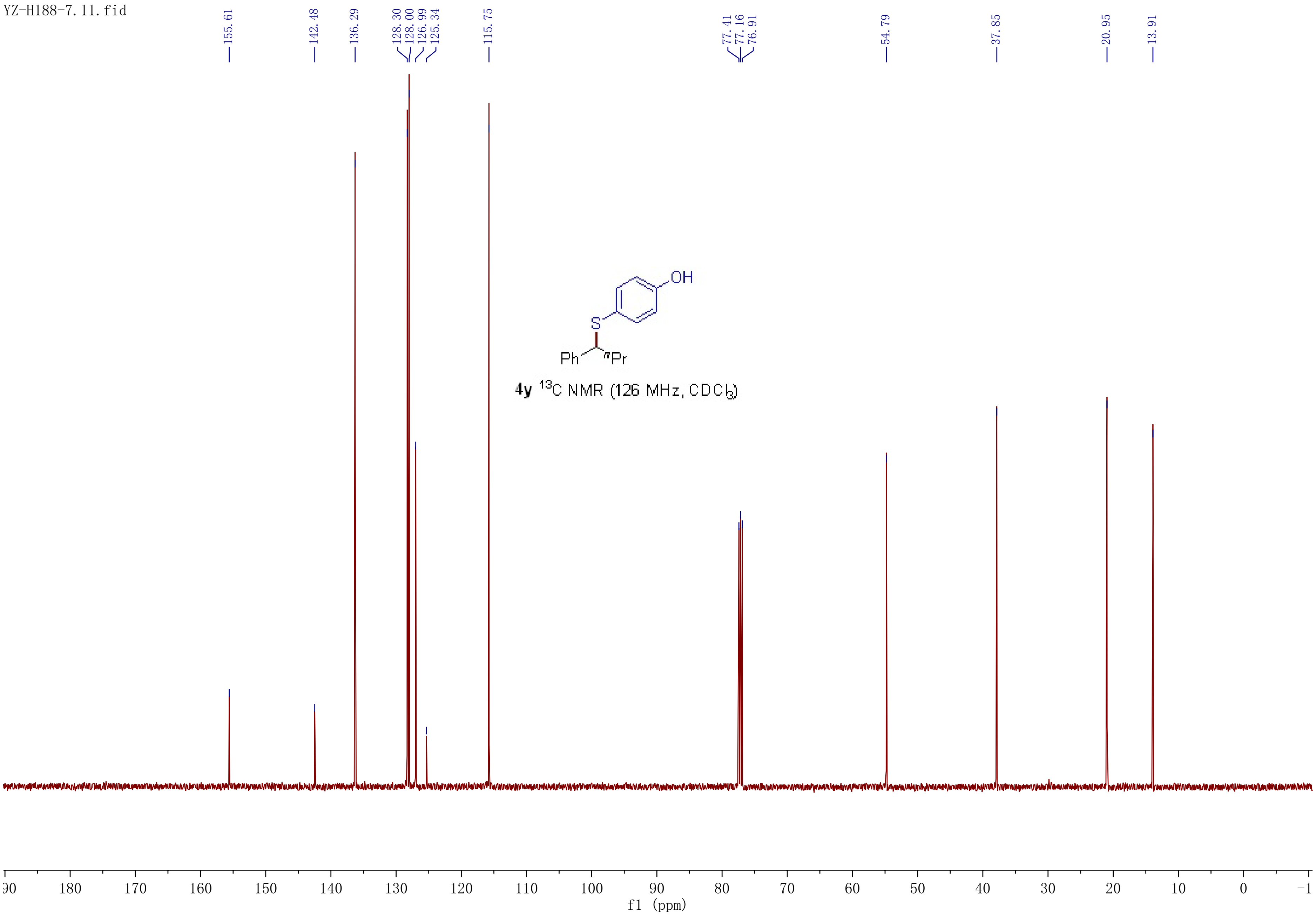

Supplementary Figure 218. ^{13}C NMR (126 MHz, $CDCl_3$) spectra for compound **4y**

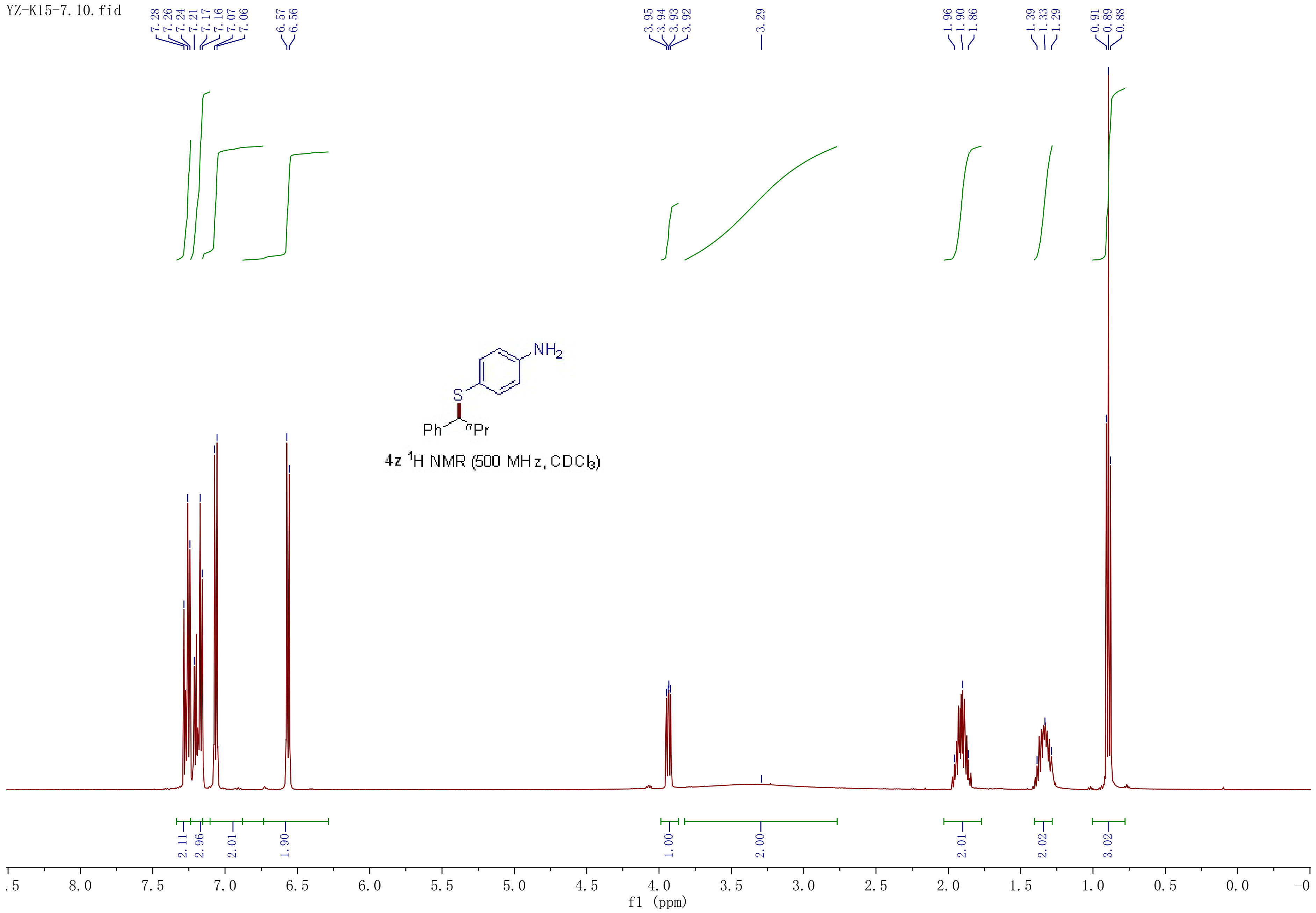

Supplementary Figure 219. ^{1}H NMR (500 MHz, $CDCl_3$) spectra for compound **4z**

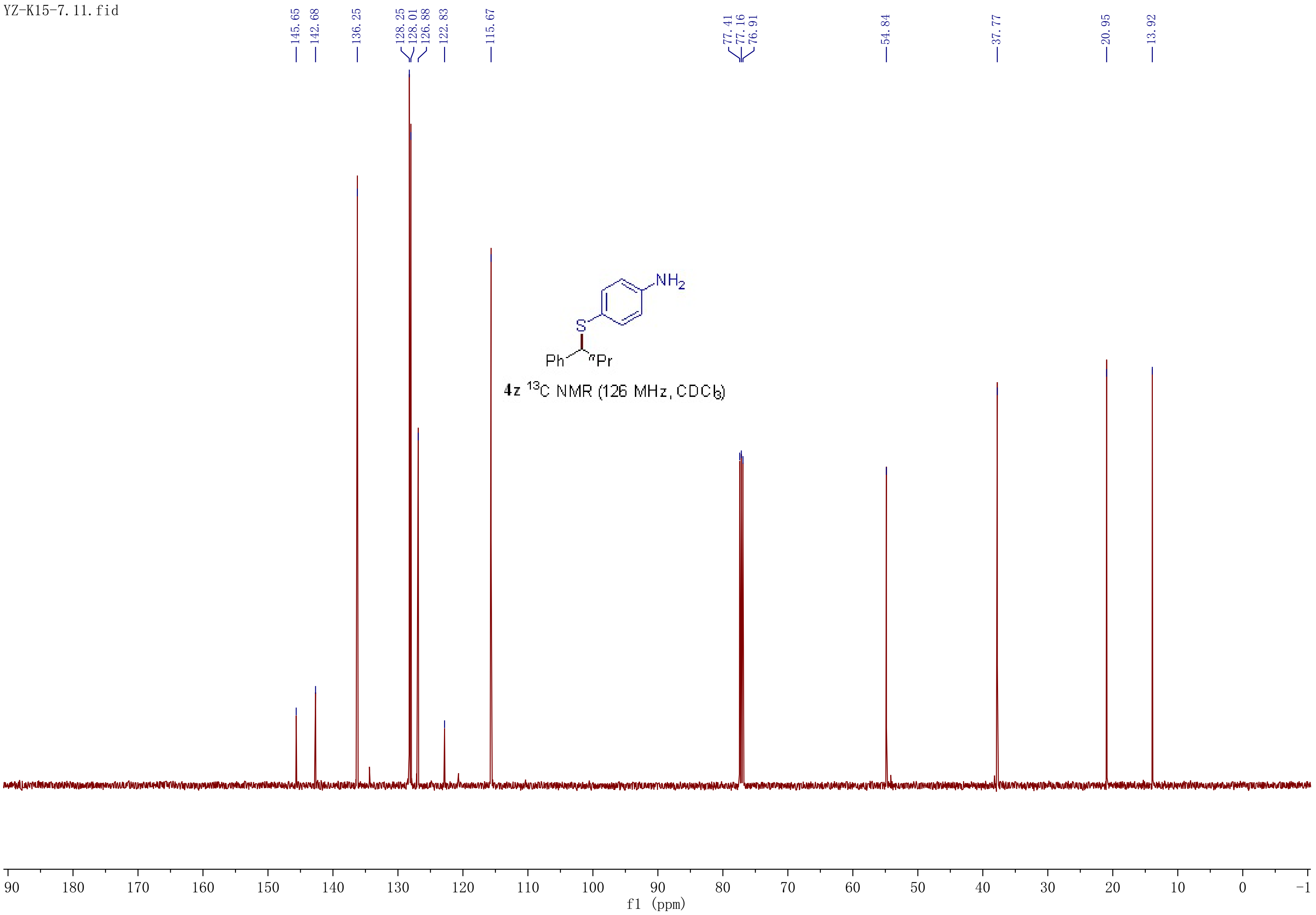

Supplementary Figure 220. ^{13}C NMR (126 MHz, $CDCl_3$) spectra for compound **4z**

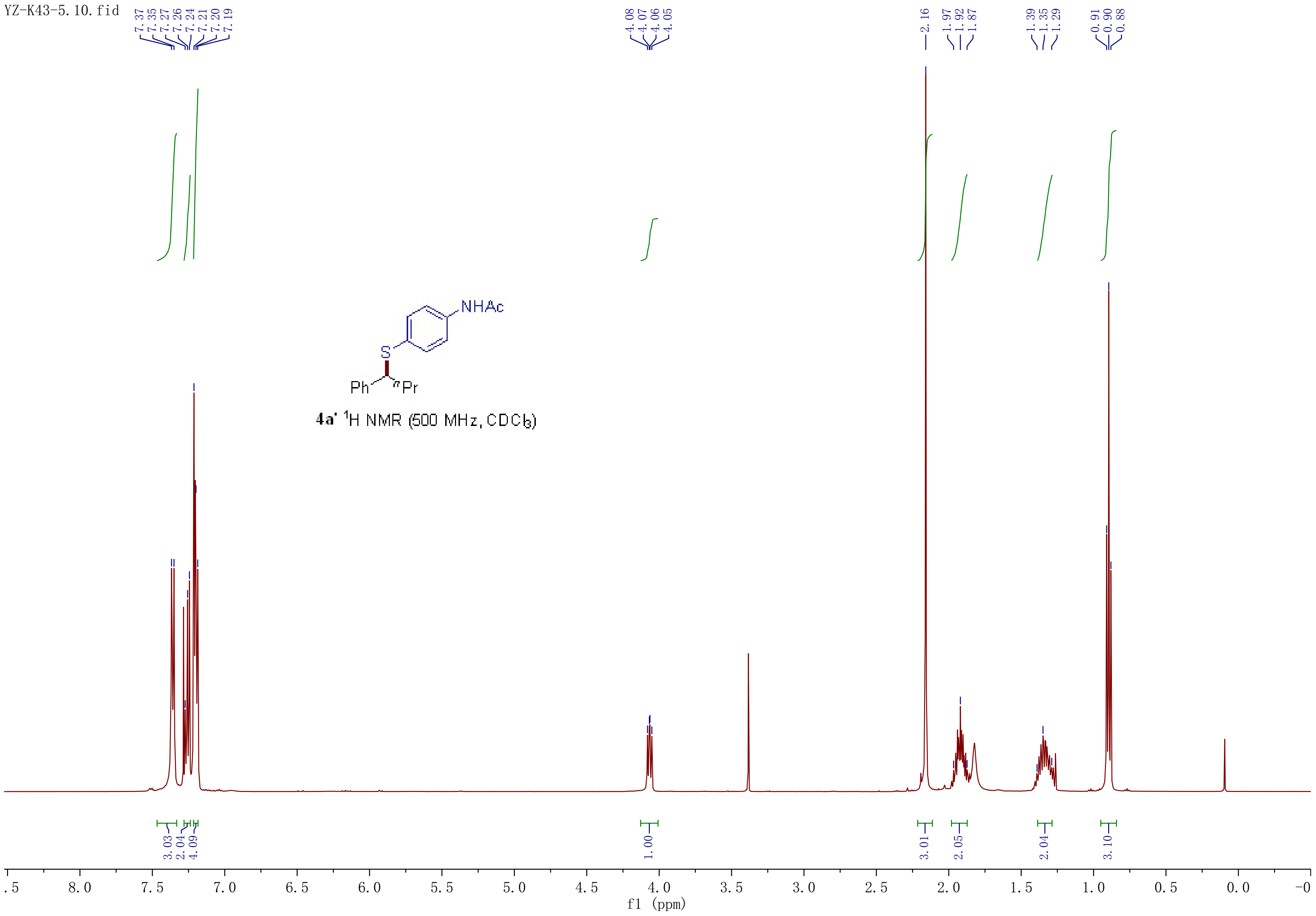

Supplementary Figure 221. ^{1}H NMR (500 MHz, $CDCl_3$) spectra for compound **4a'**

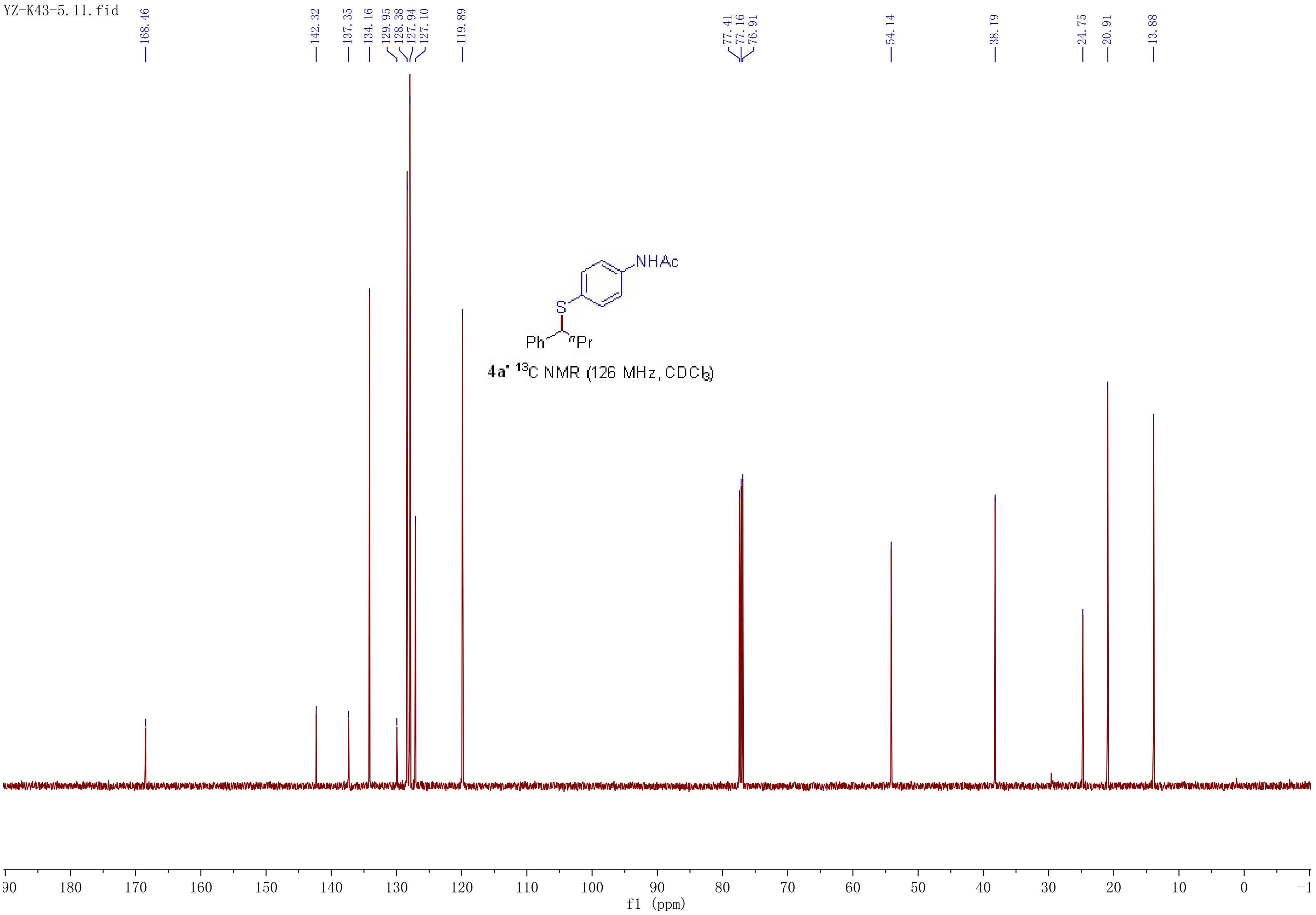

Supplementary Figure 222. ^{13}C NMR (126 MHz, $CDCl_3$) spectra for compound **4a'**

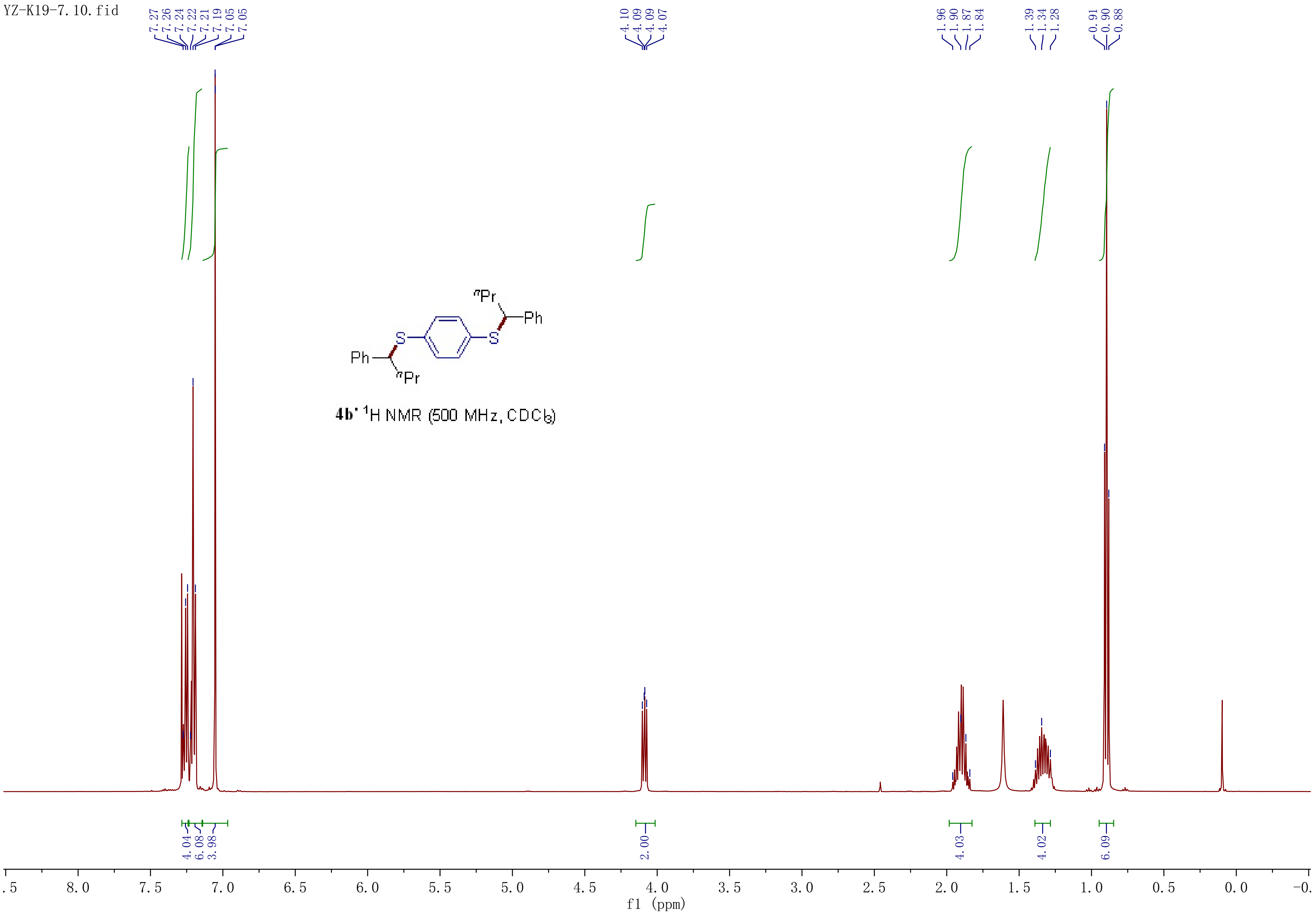

Supplementary Figure 223. ^{1}H NMR (500 MHz, $CDCl_3$) spectra for compound **4b'**

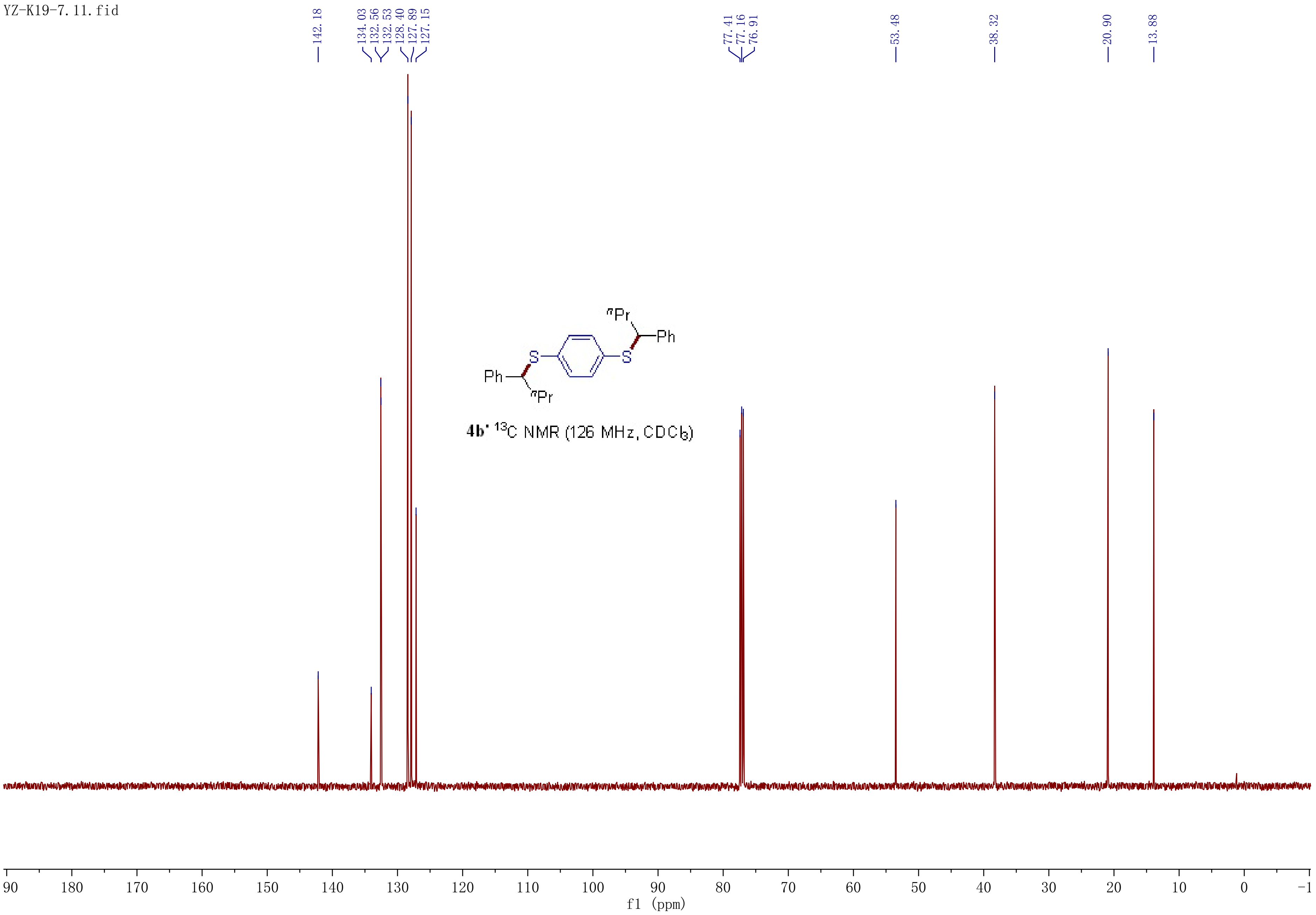

Supplementary Figure 224. ^{13}C NMR (126 MHz, $CDCl_3$) spectra for compound **4b'**

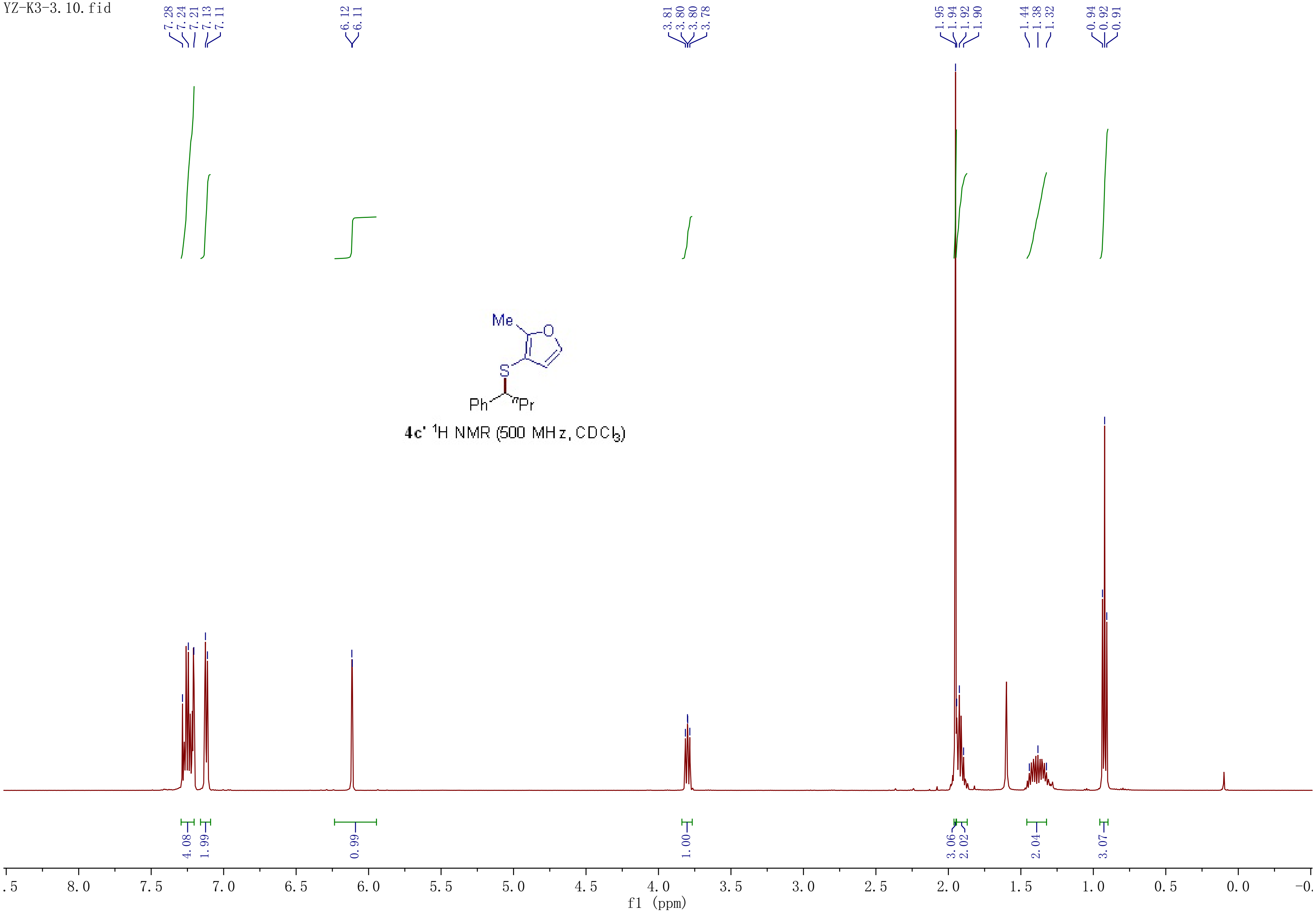

Supplementary Figure 225. ^{1}H NMR (500 MHz, $CDCl_3$) spectra for compound **4c'**

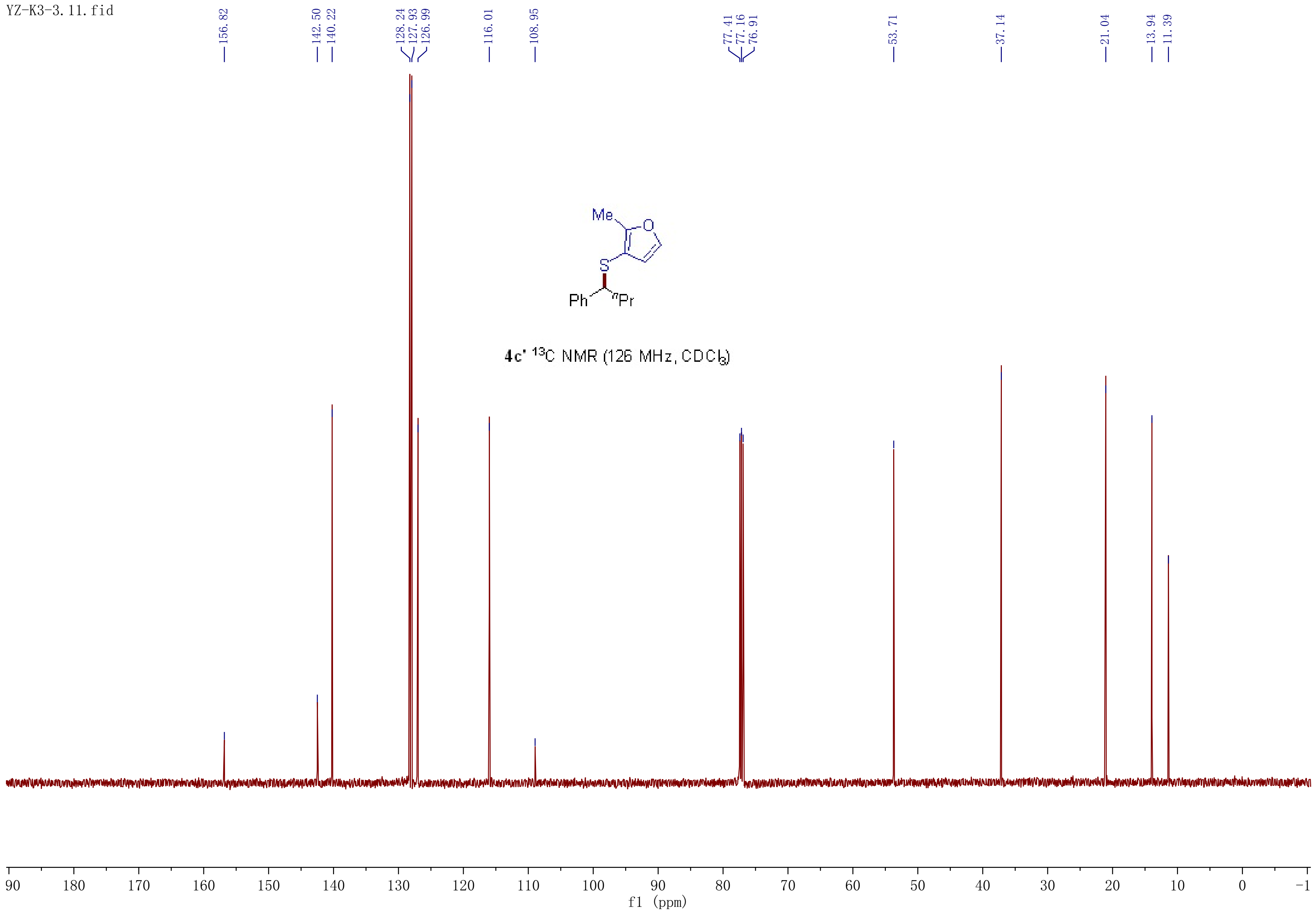

Supplementary Figure 226. ^{13}C NMR (126 MHz, $CDCl_3$) spectra for compound **4c'**

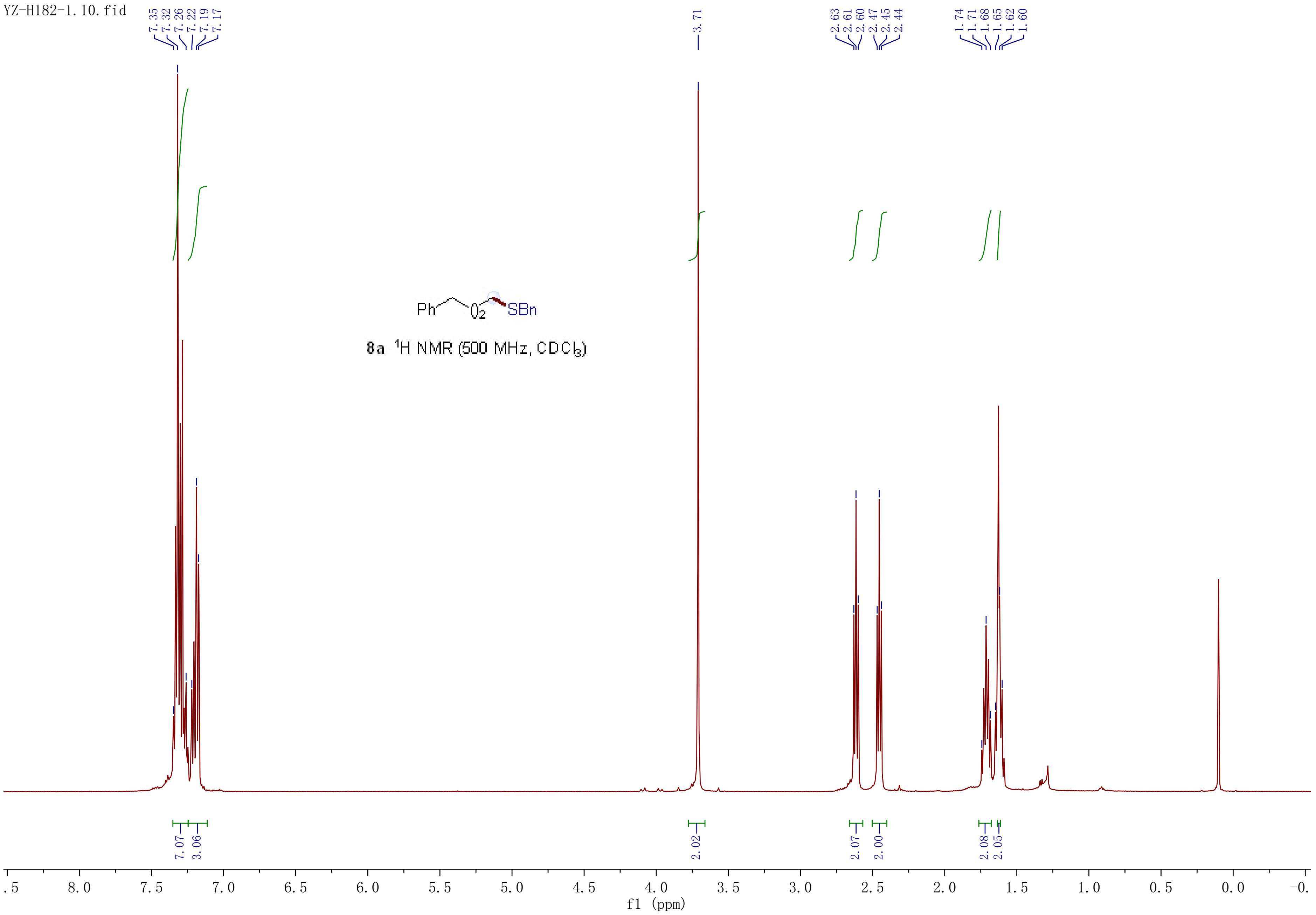

Supplementary Figure 227. ^1H NMR (500 MHz, $CDCl_3$) spectra for compound **8a**

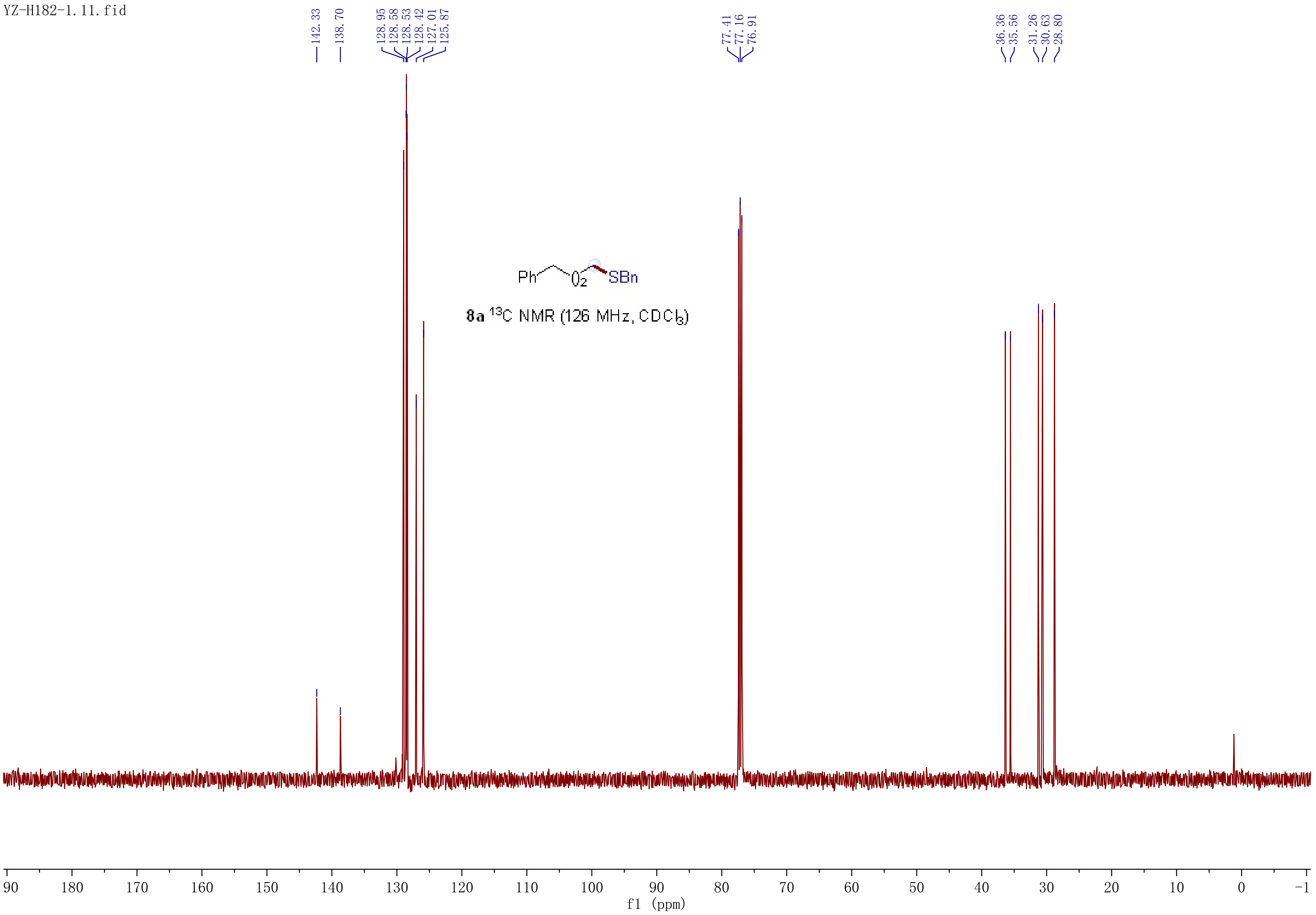

Supplementary Figure 228. ^{13}C NMR (126 MHz, $CDCl_3$) spectra for compound **8a**

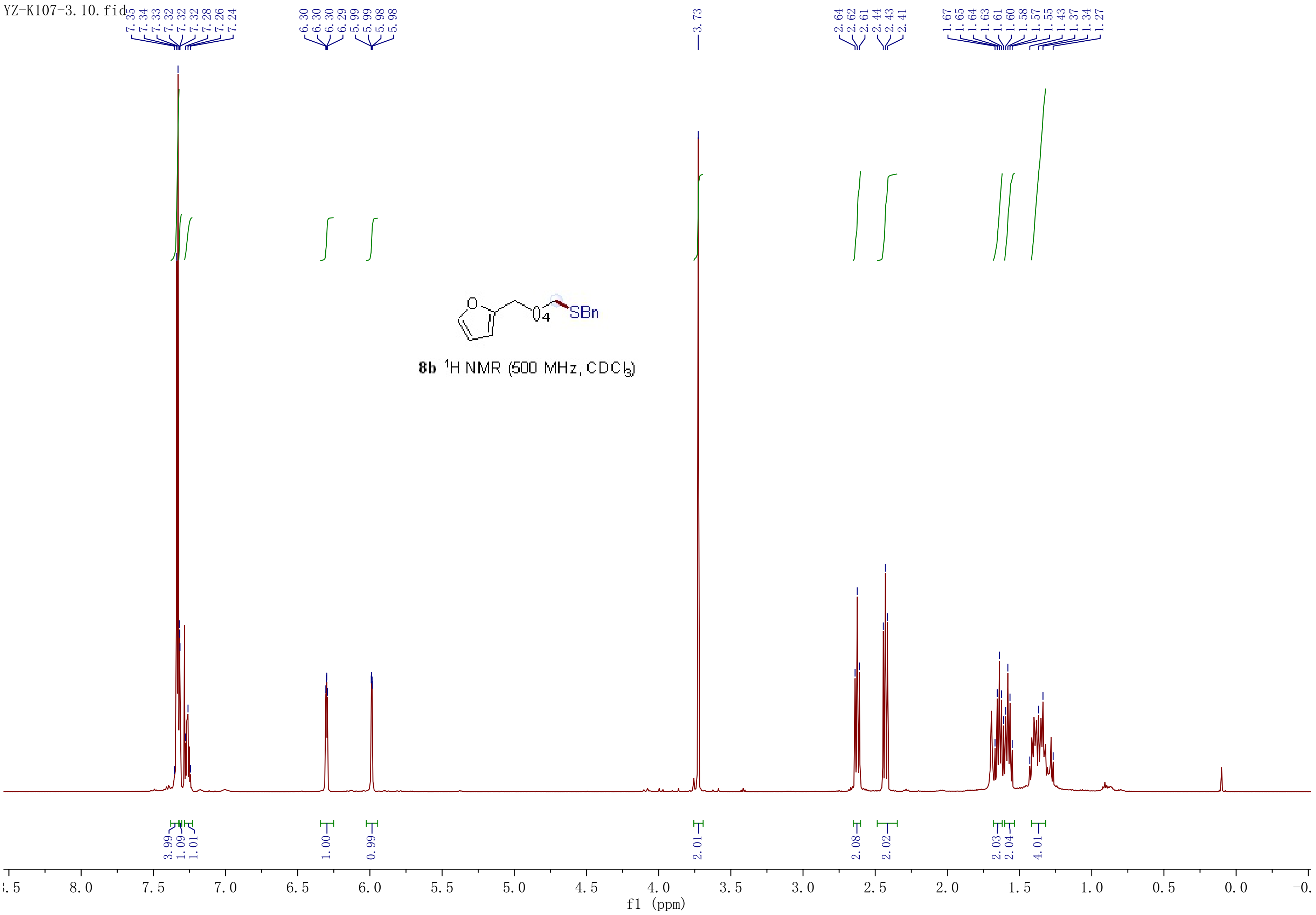

Supplementary Figure 229. ¹H NMR (500 MHz, $CDCl_3$) spectra for compound **8b**

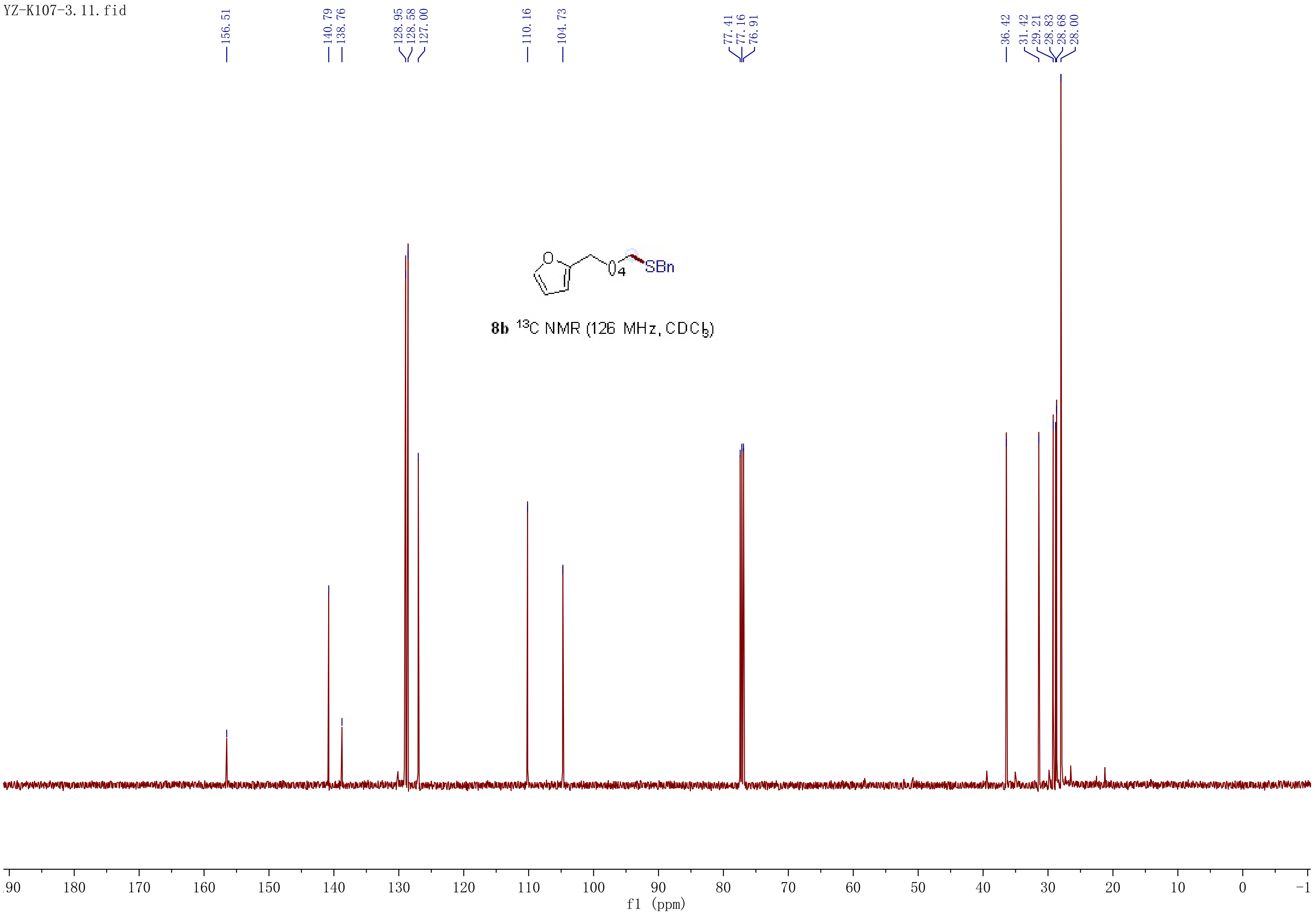

Supplementary Figure 230. ^{13}C NMR (126 MHz, CDCl$_3$) spectra for compound **8b**

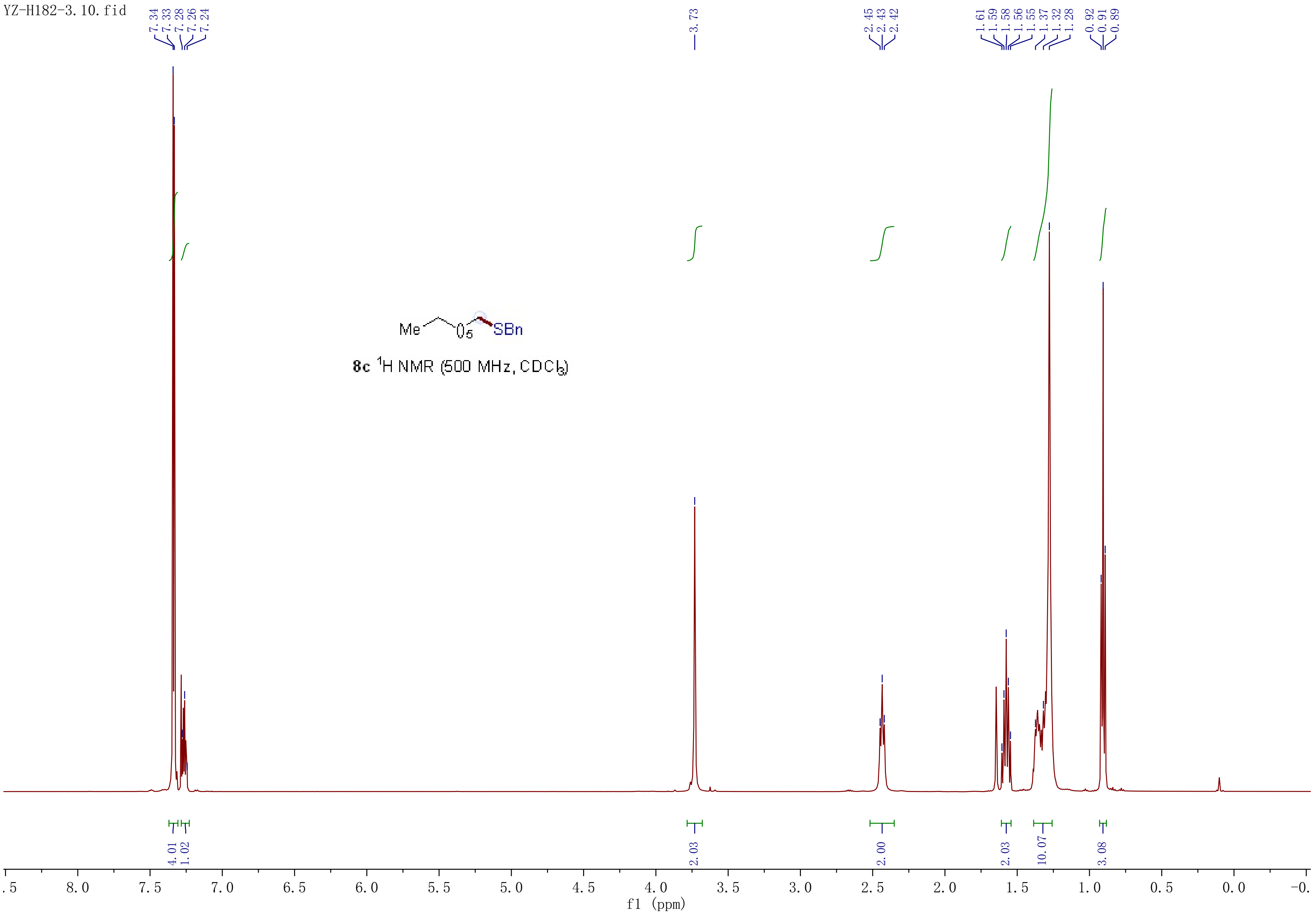

Supplementary Figure 231. ^{1}H NMR (500 MHz, $CDCl_3$) spectra for compound **8c**

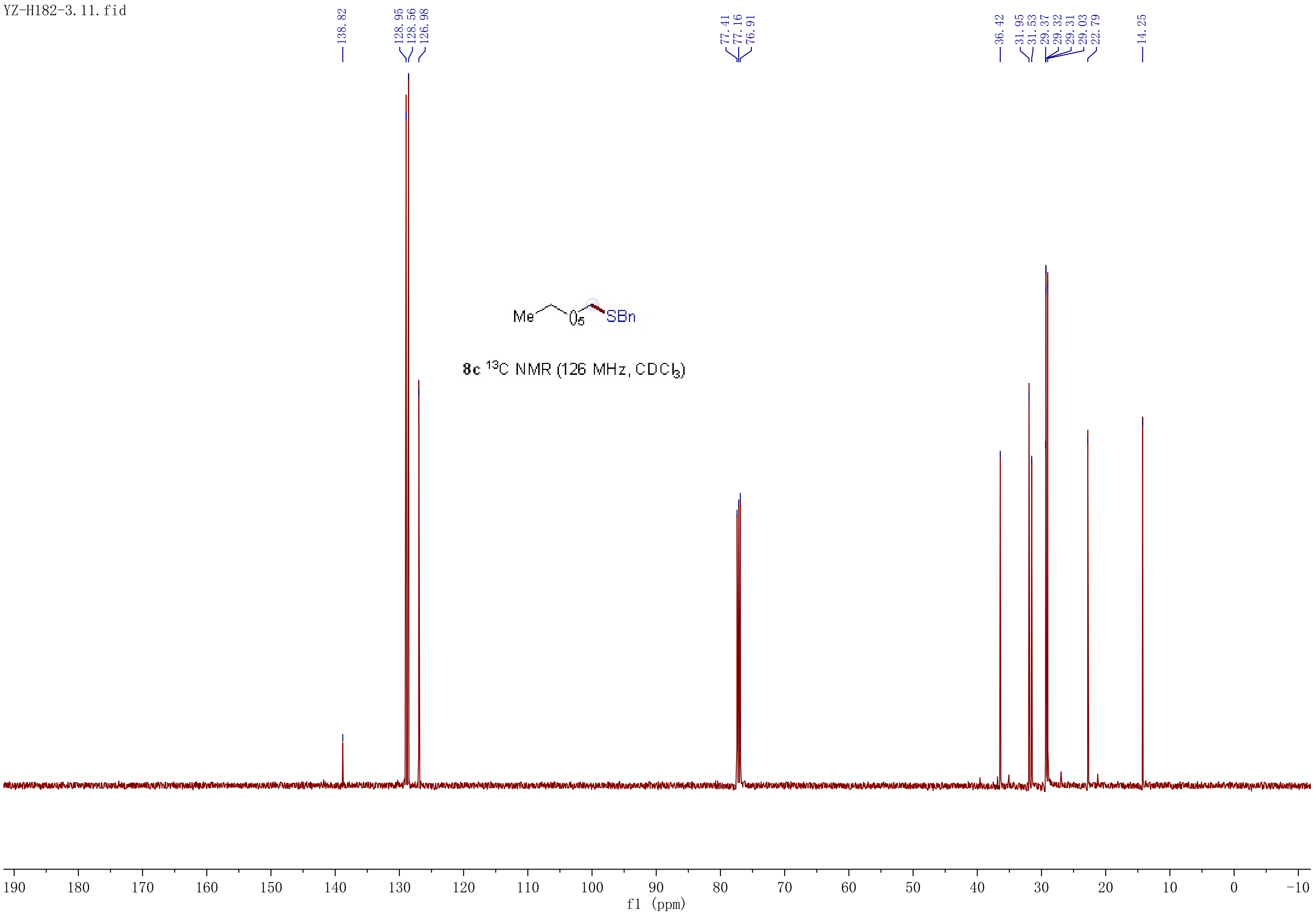

Supplementary Figure 232. ^{13}C NMR (126 MHz, $CDCl_3$) spectra for compound **8c**

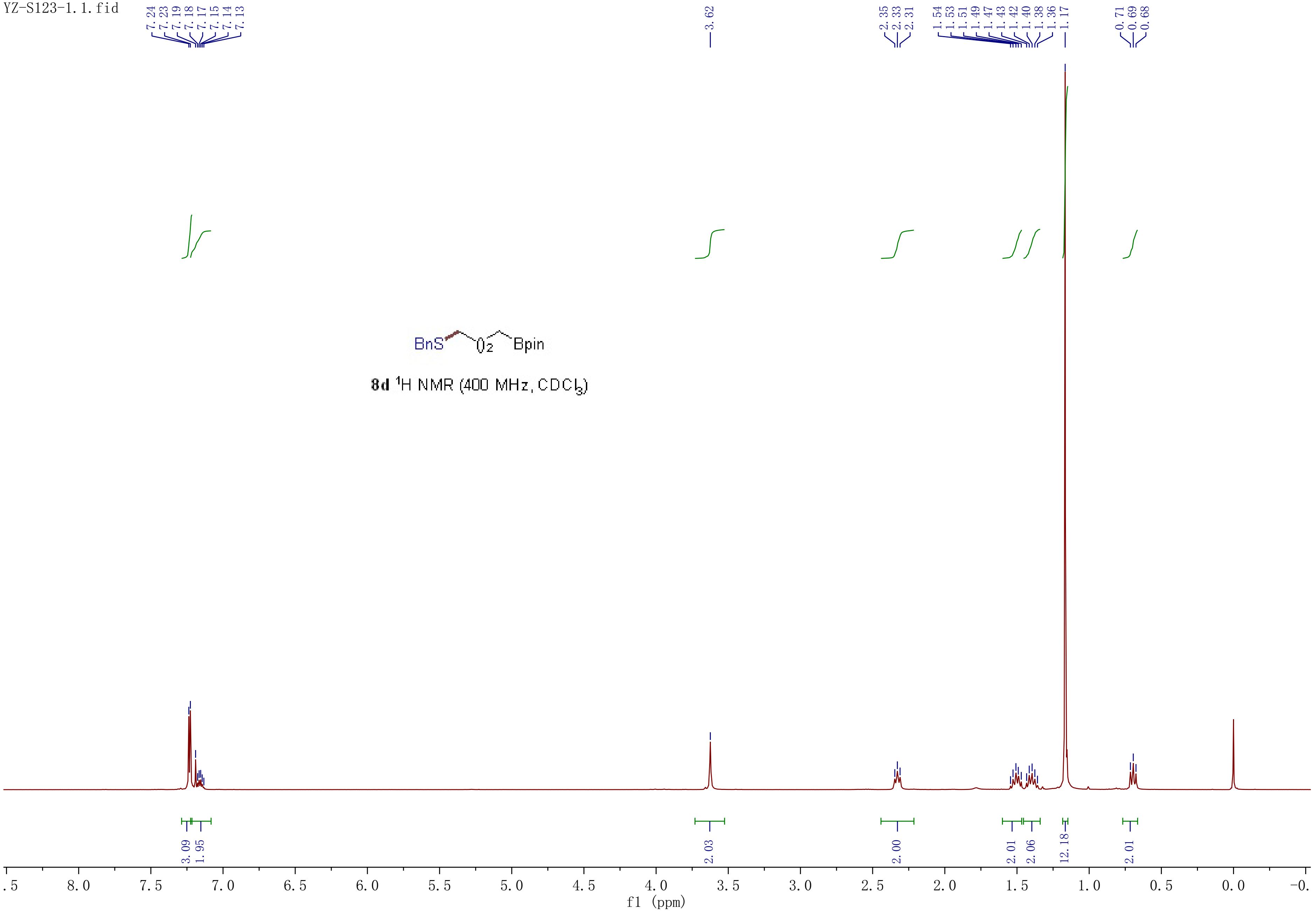

Supplementary Figure 233. ^{1}H NMR (400 MHz, $CDCl_3$) spectra for compound **8d**

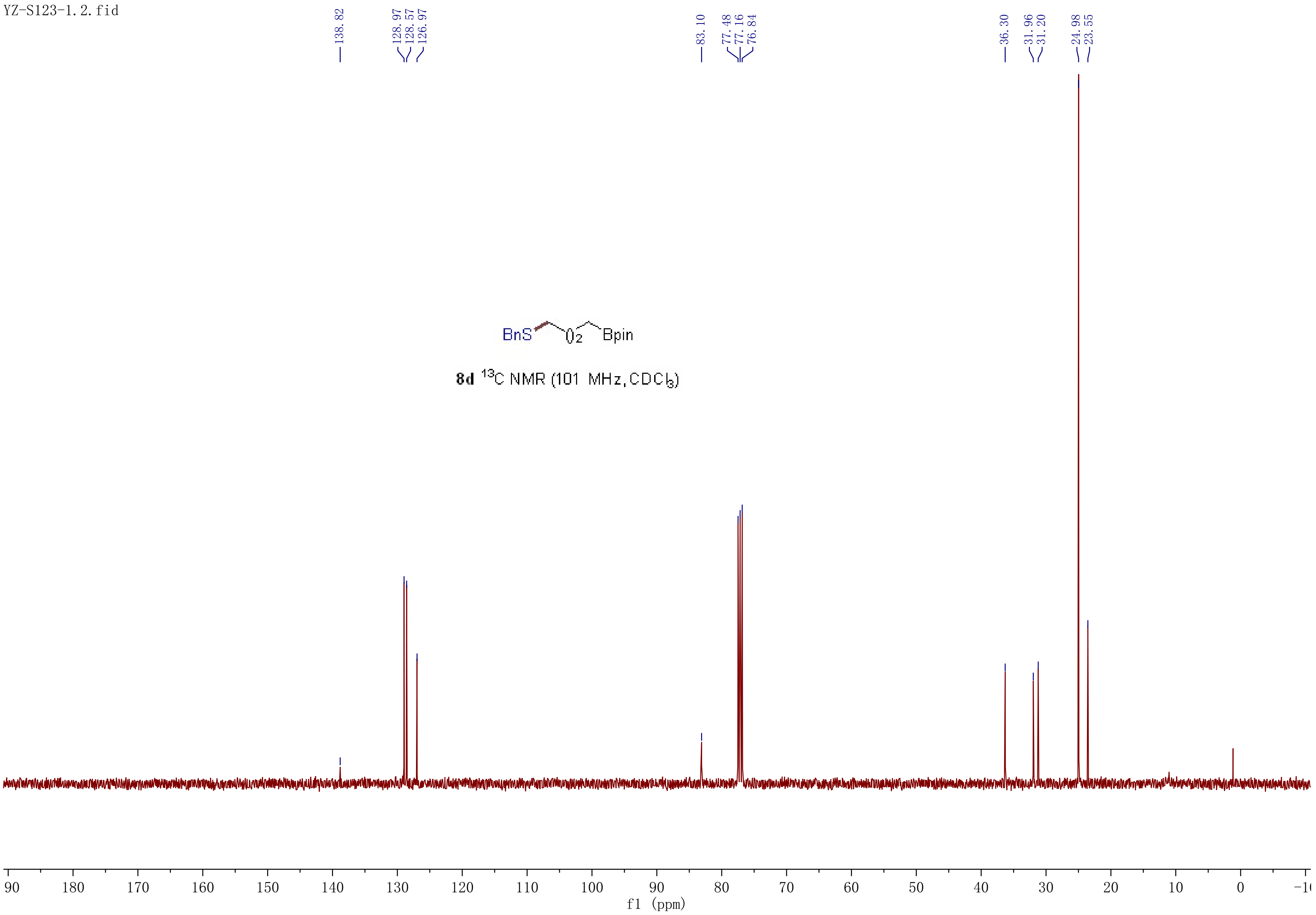

Supplementary Figure 234. ^{13}C NMR (101 MHz, CDCl$_3$) spectra for compound **8d**

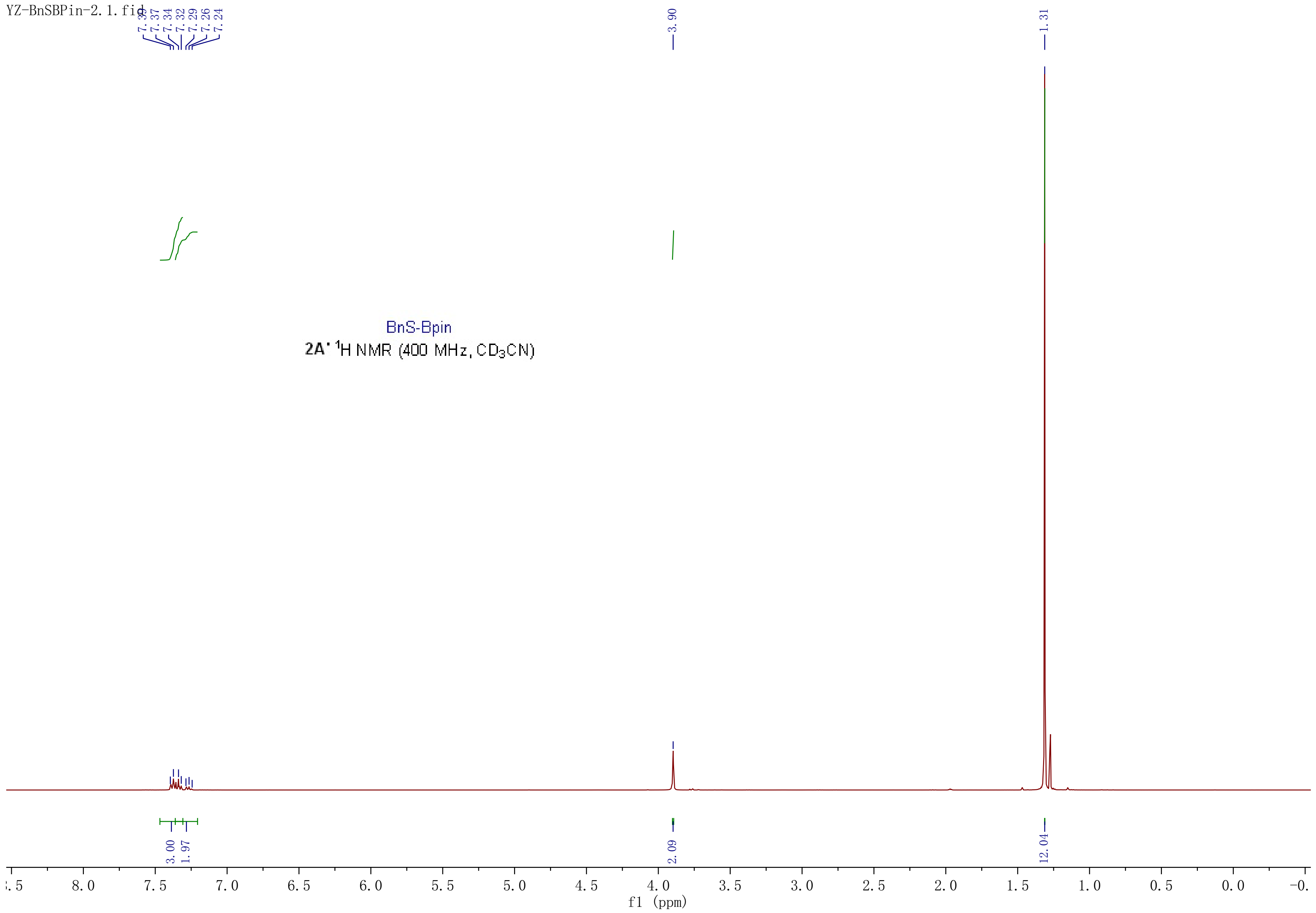

Supplementary Figure 235. ^{1}H NMR (400 MHz, CD_3CN) spectra for compound **2A'**

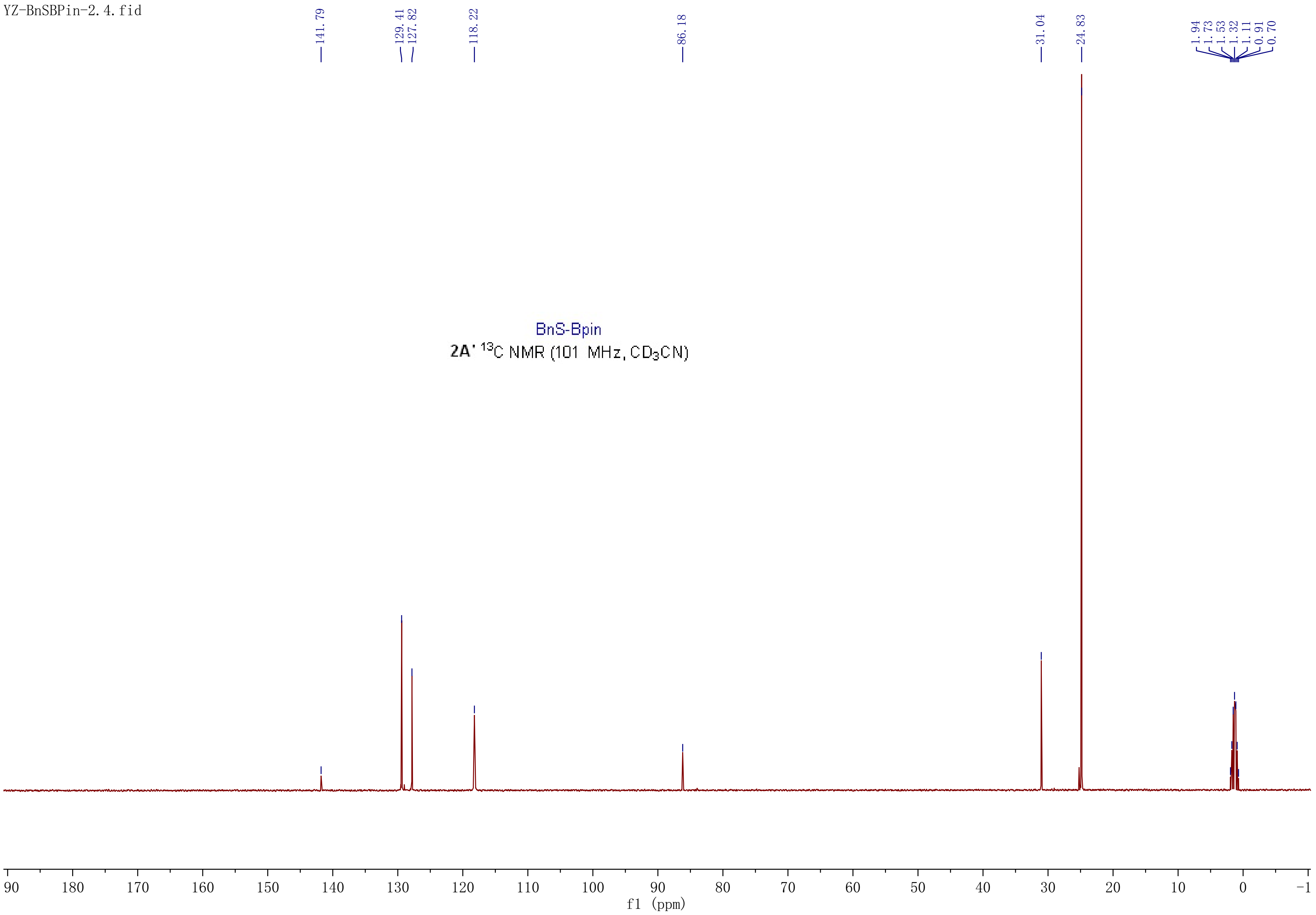

Supplementary Figure 236. ^{13}C NMR (101 MHz, CD_3CN) spectra for compound **2A'**

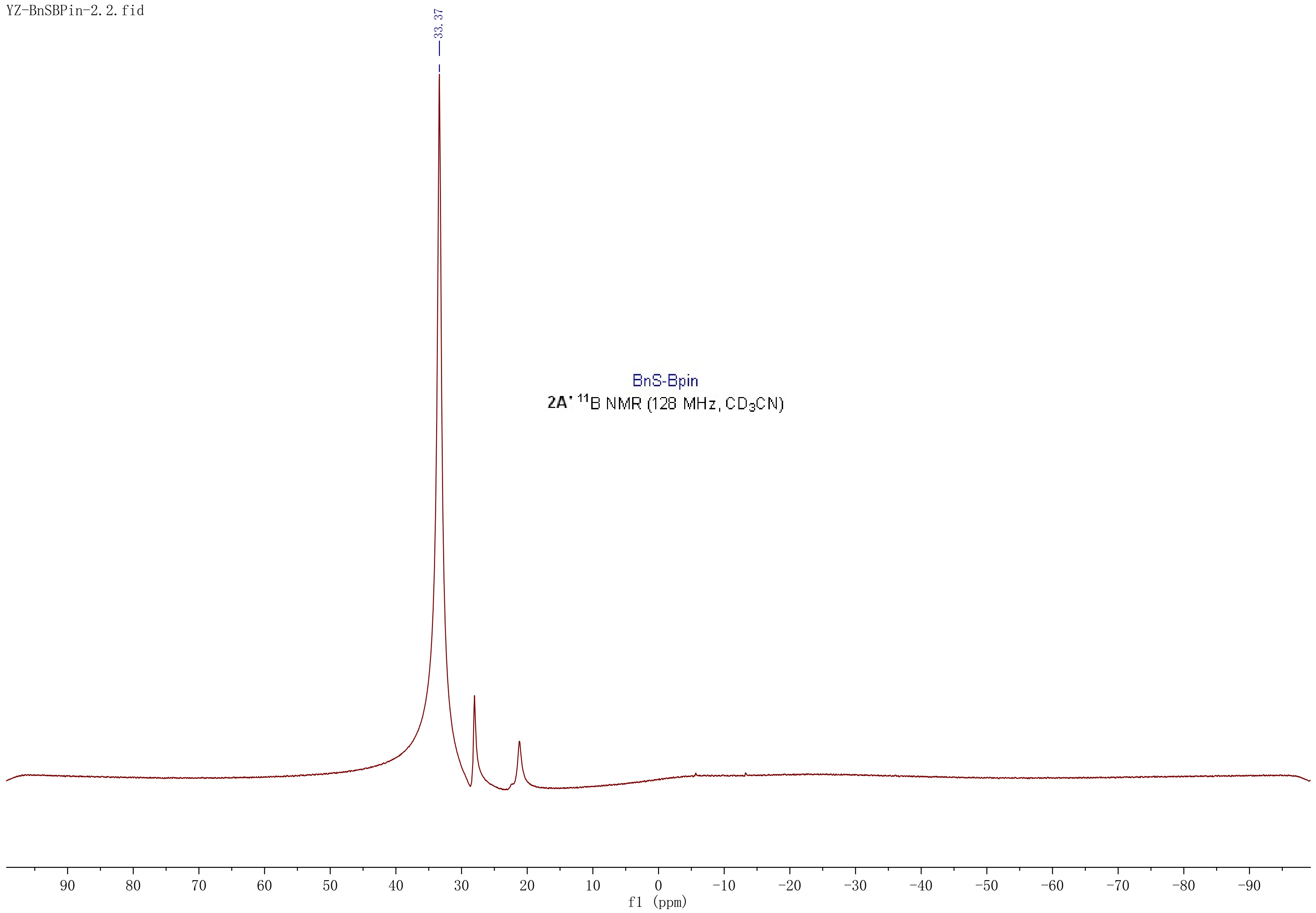

Supplementary Figure 237. ^{11}B NMR (128 MHz, CD_3CN) spectra for compound **2A'**

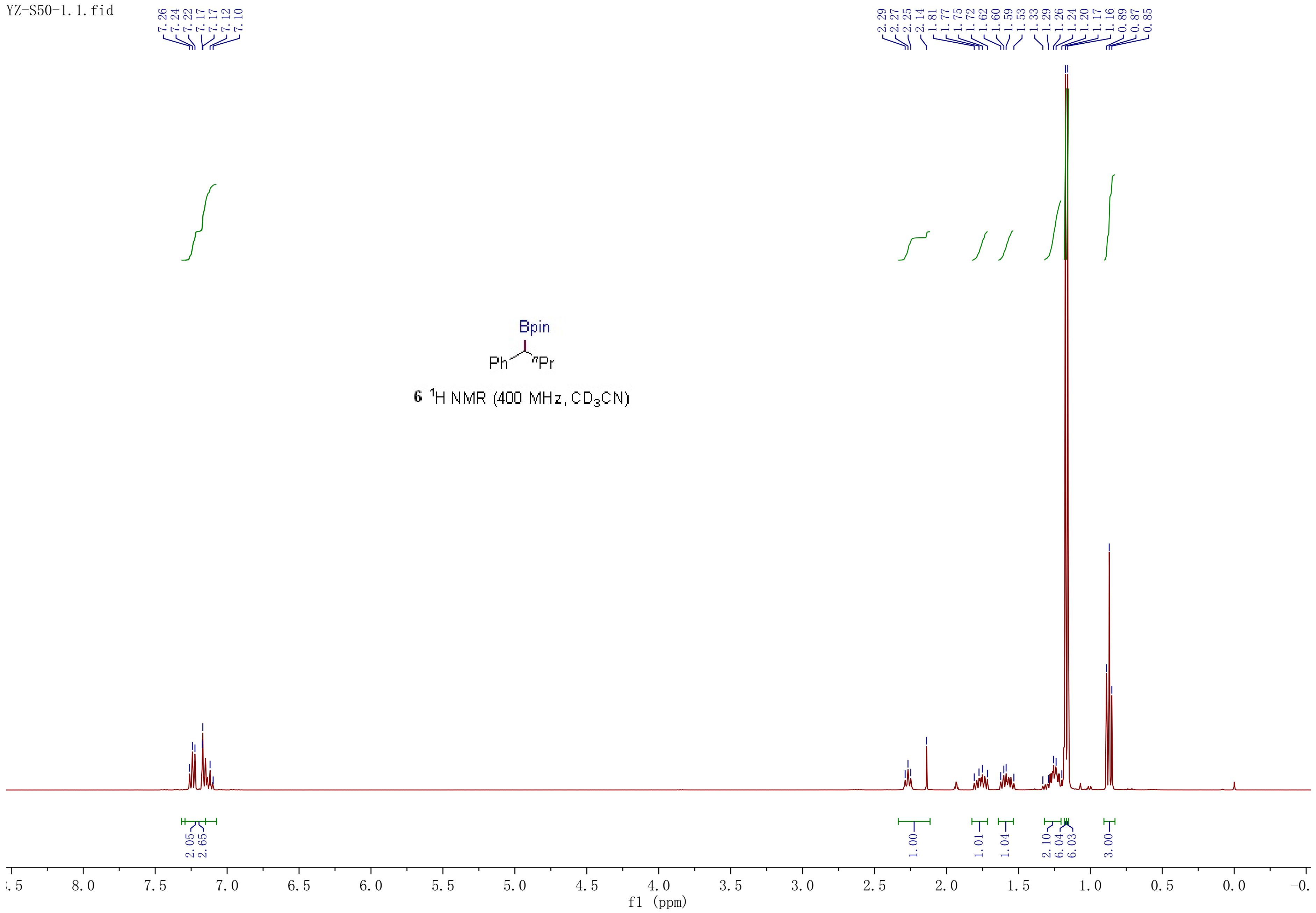

Supplementary Figure 238. ^{1}H NMR (400 MHz, CD_3CN) spectra for compound **6**

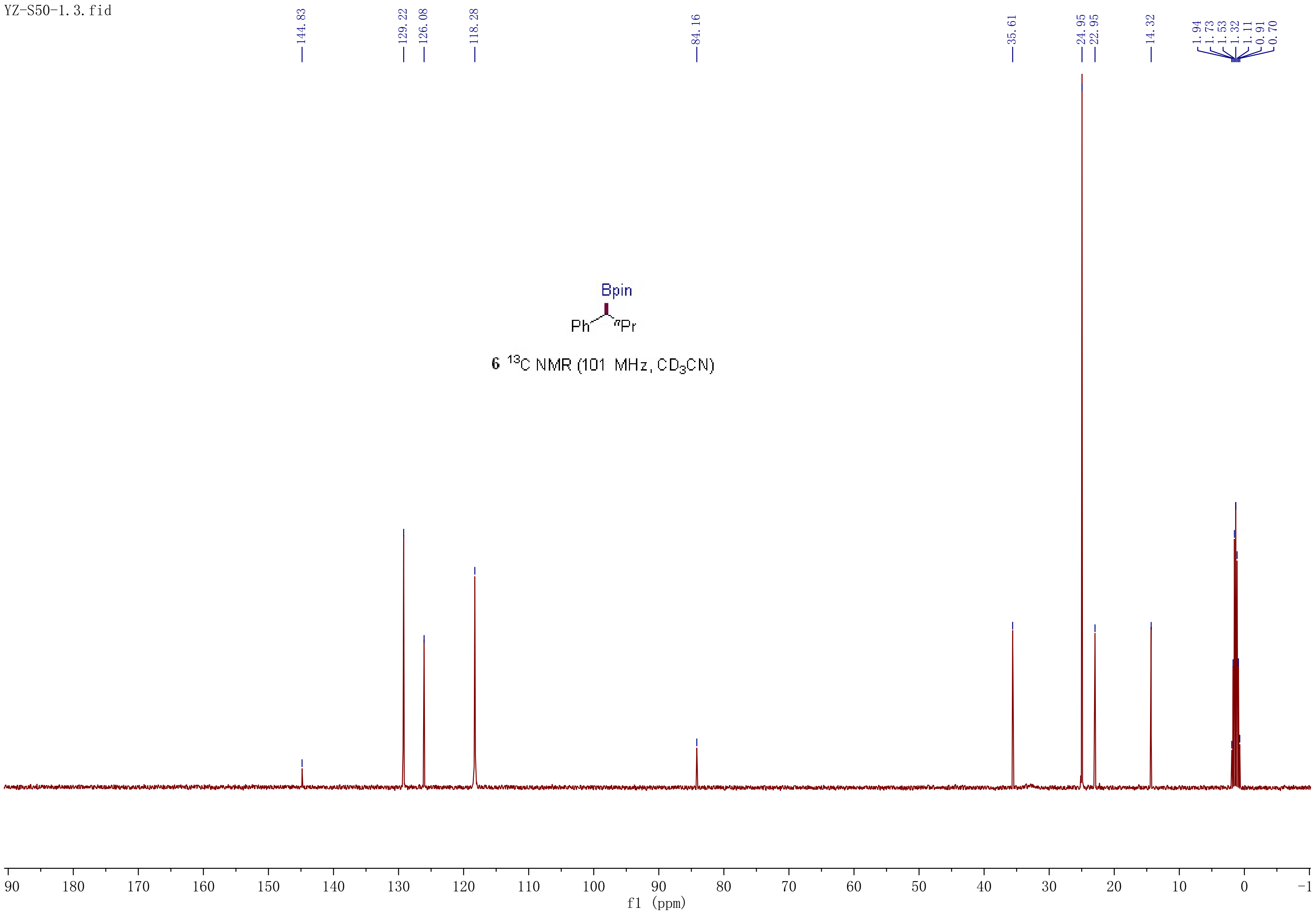

Supplementary Figure 239. ^{13}C NMR (101 MHz, CD_3CN) spectra for compound **6**

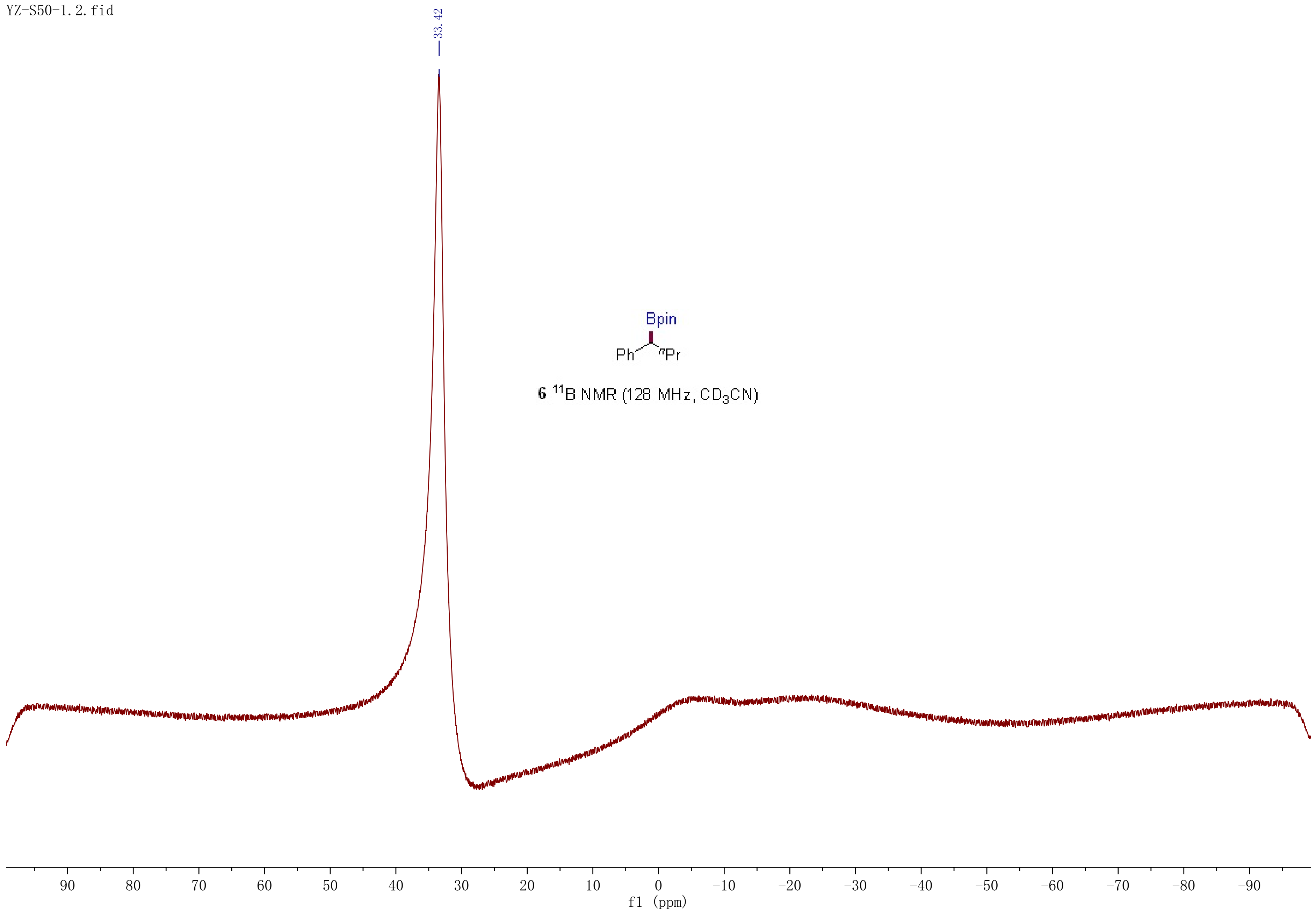

Supplementary Figure 240. ^{11}B NMR (128 MHz, CD_3CN) spectra for compound **6**

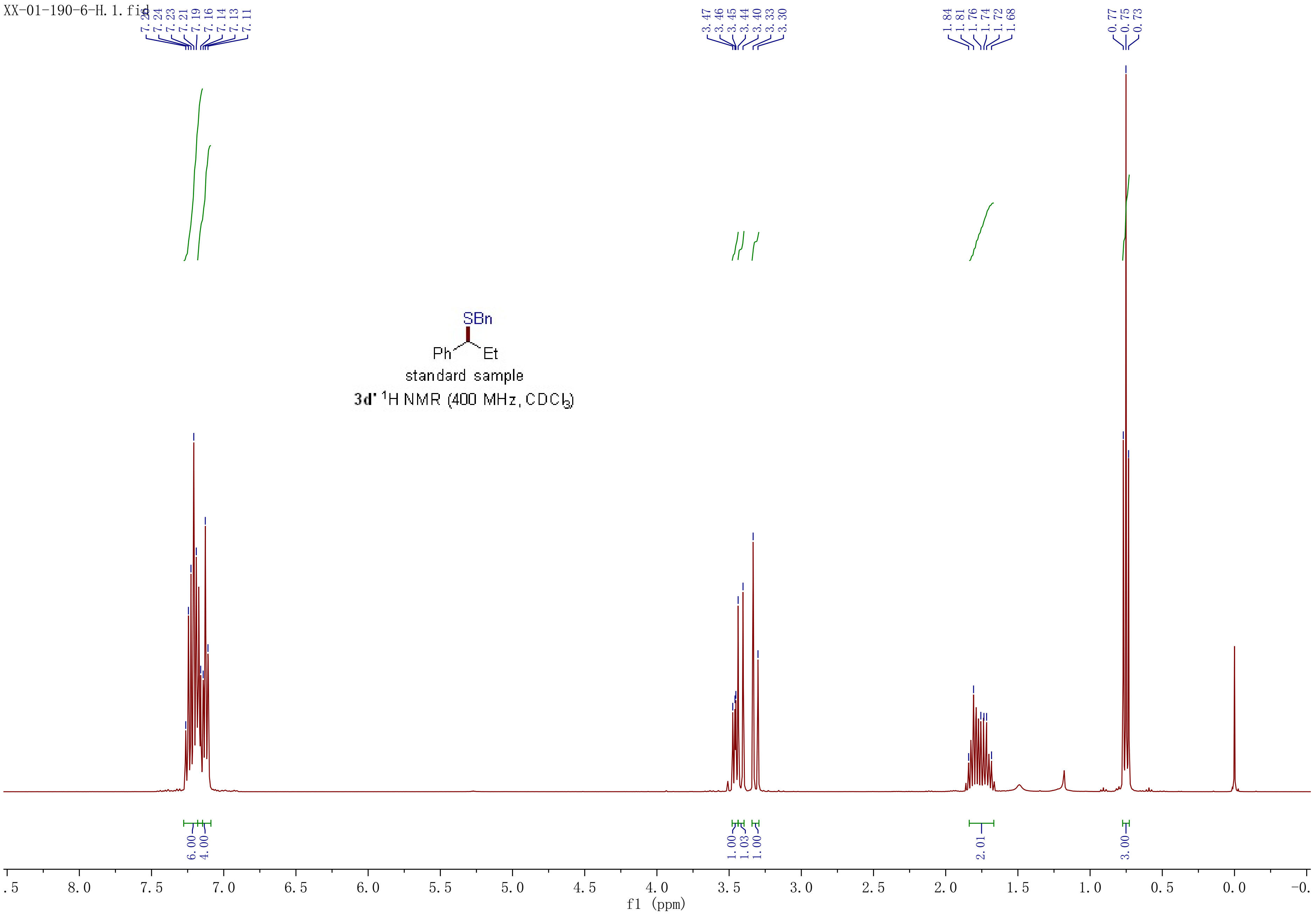

Supplementary Figure 241. ^{1}H NMR (400 MHz, $CDCl_3$) spectra for compound **3d'**

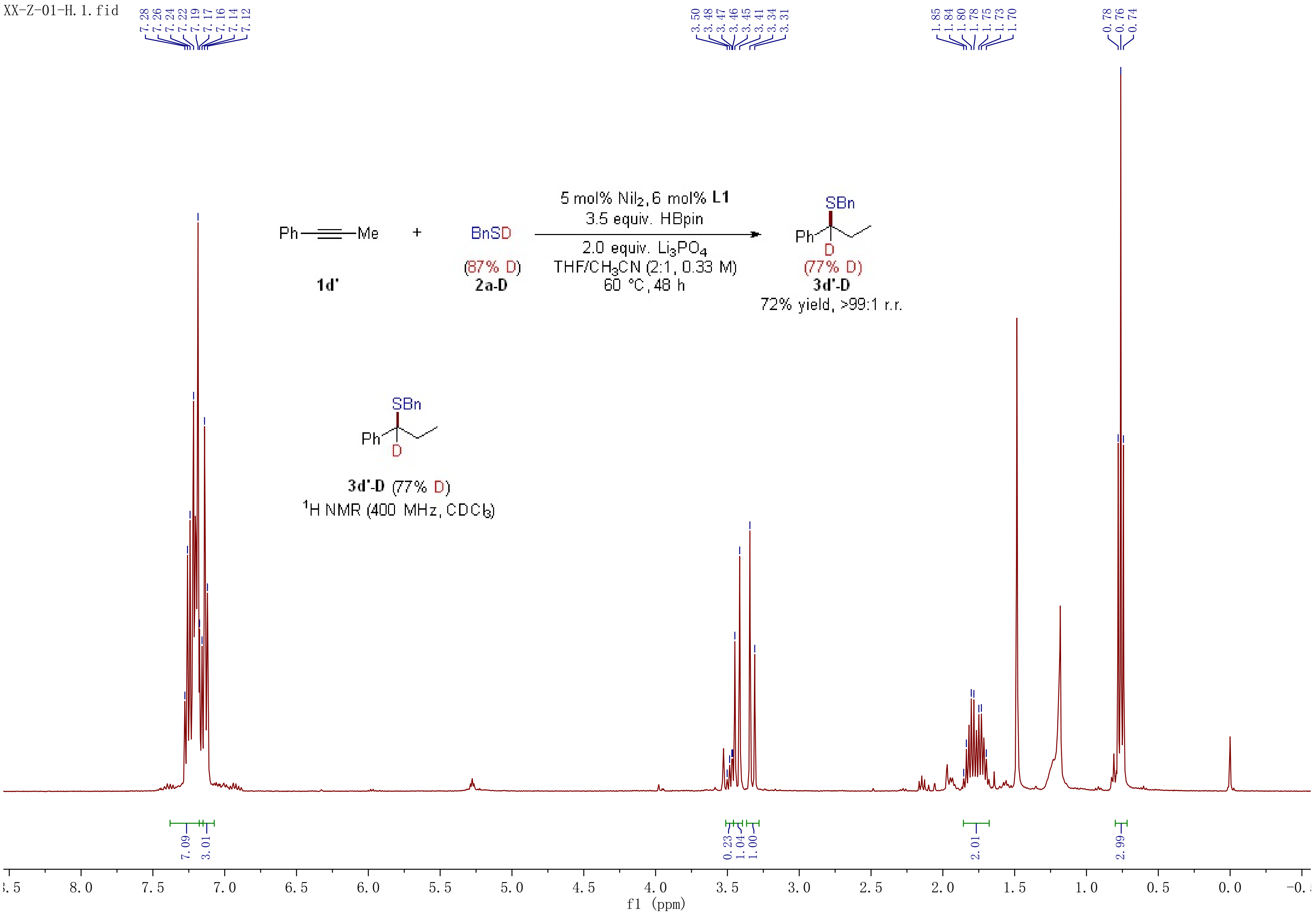

Supplementary Figure 242. ^{1}H NMR (400 MHz, $CDCl_3$) spectra for compound **3d'-D**

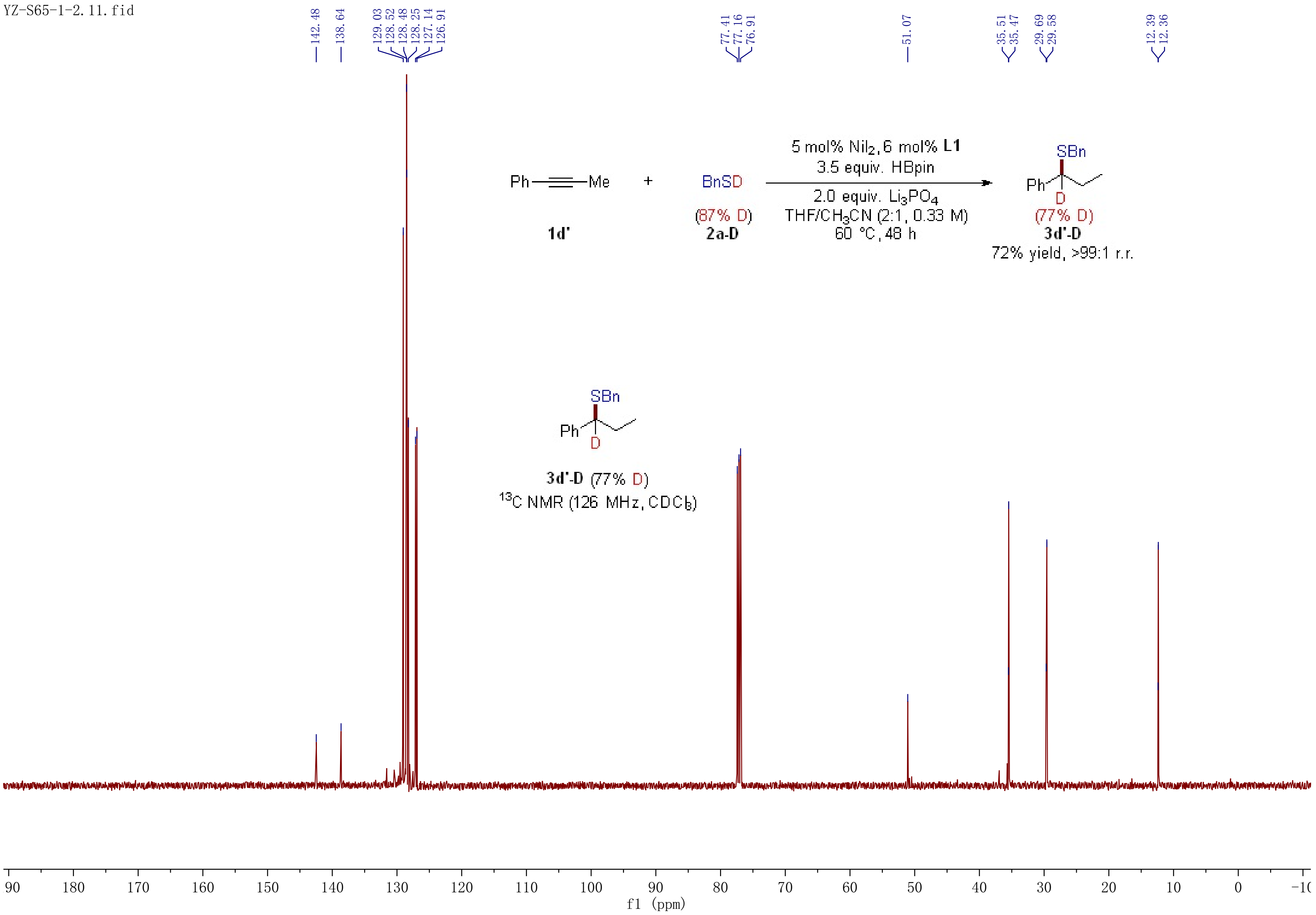

Supplementary Figure 243. ^{13}C NMR (126 MHz, $CDCl_3$) spectra for compound **3d'-D**

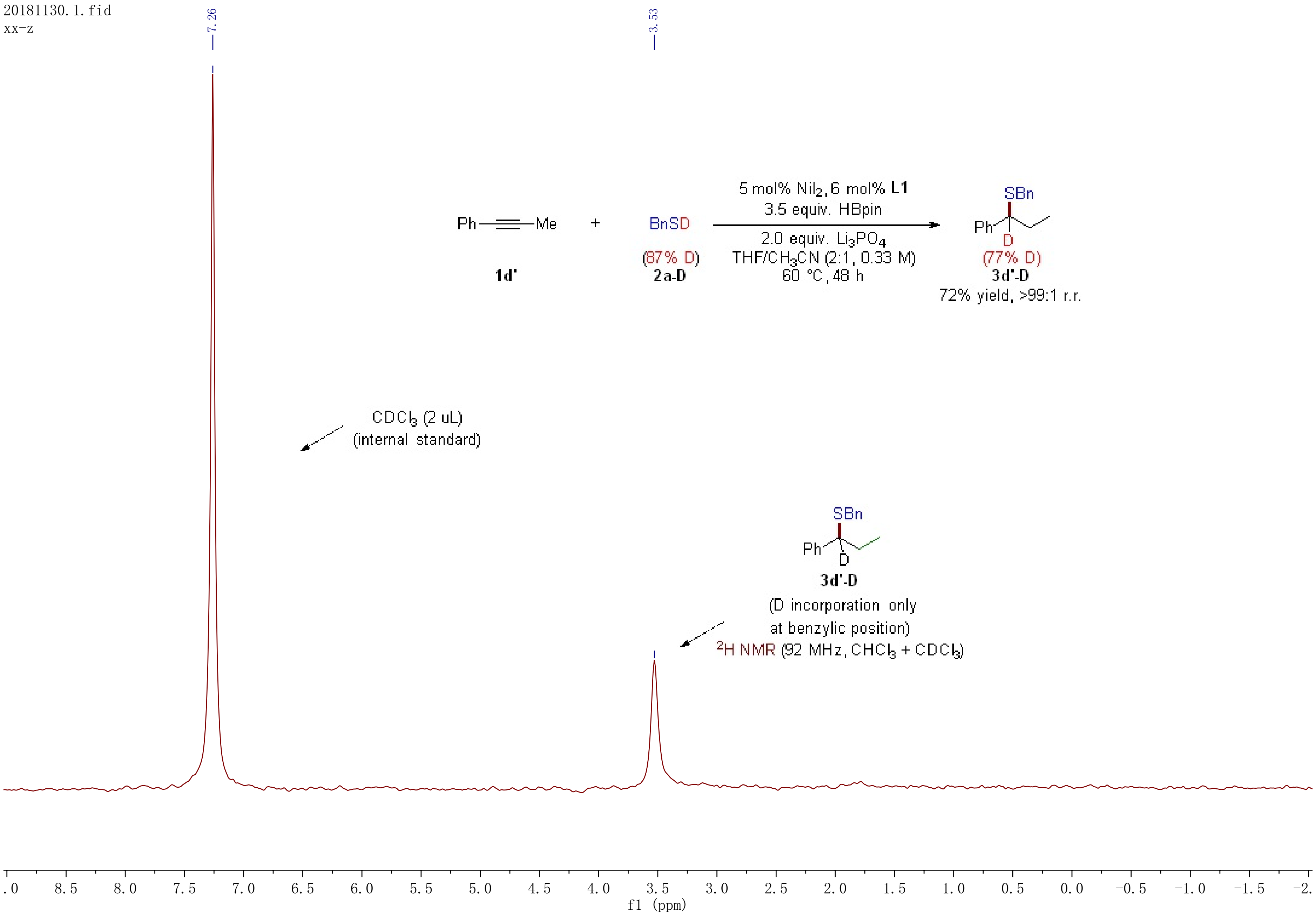

Supplementary Figure 244. ^{2}H NMR (92 MHz, $CHCl_3$+$CDCl_3$) spectra for compound **3d'-D**

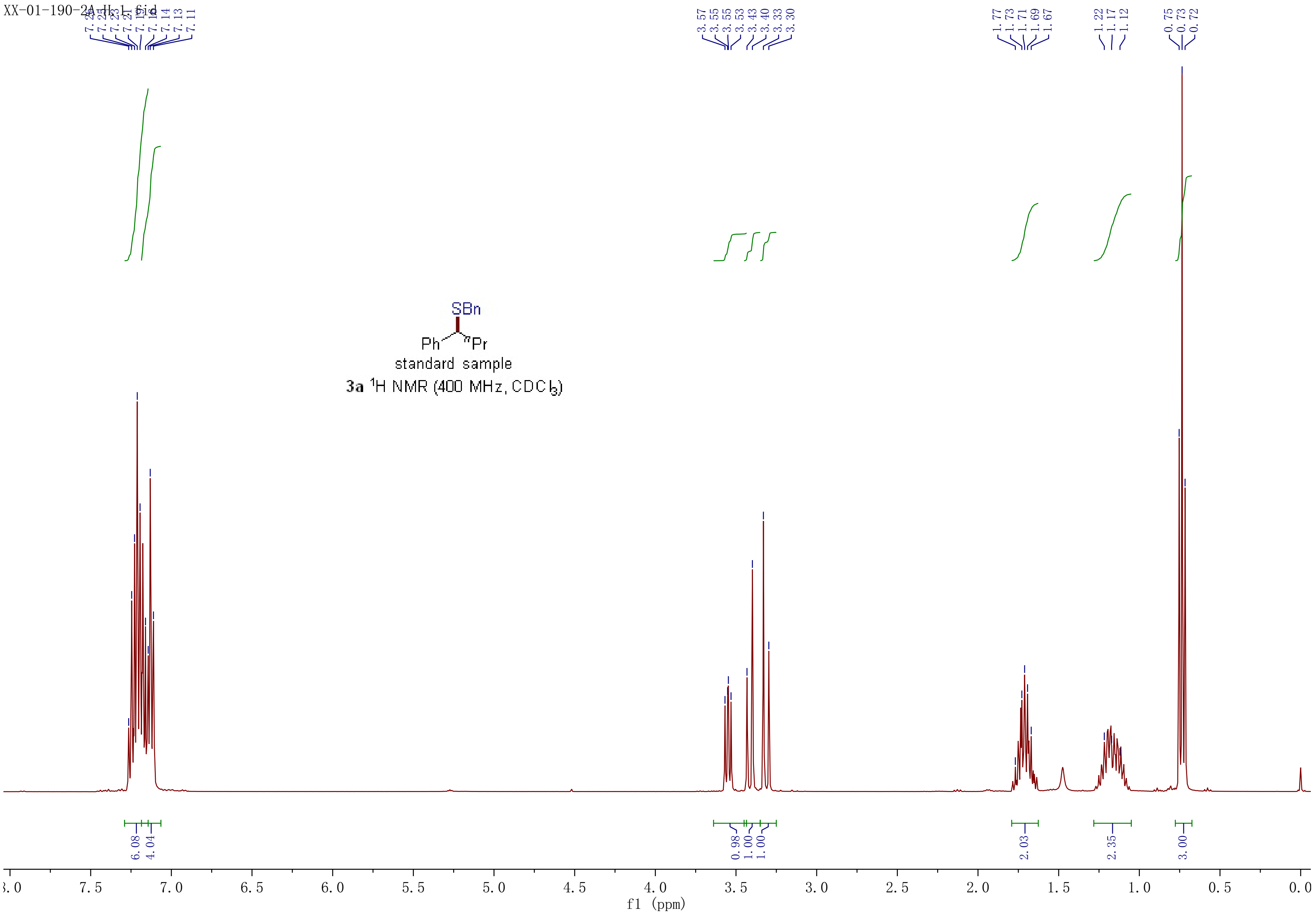

Supplementary Figure 245. ^{1}H NMR (400 MHz, $CDCl_3$) spectra for compound **3a**

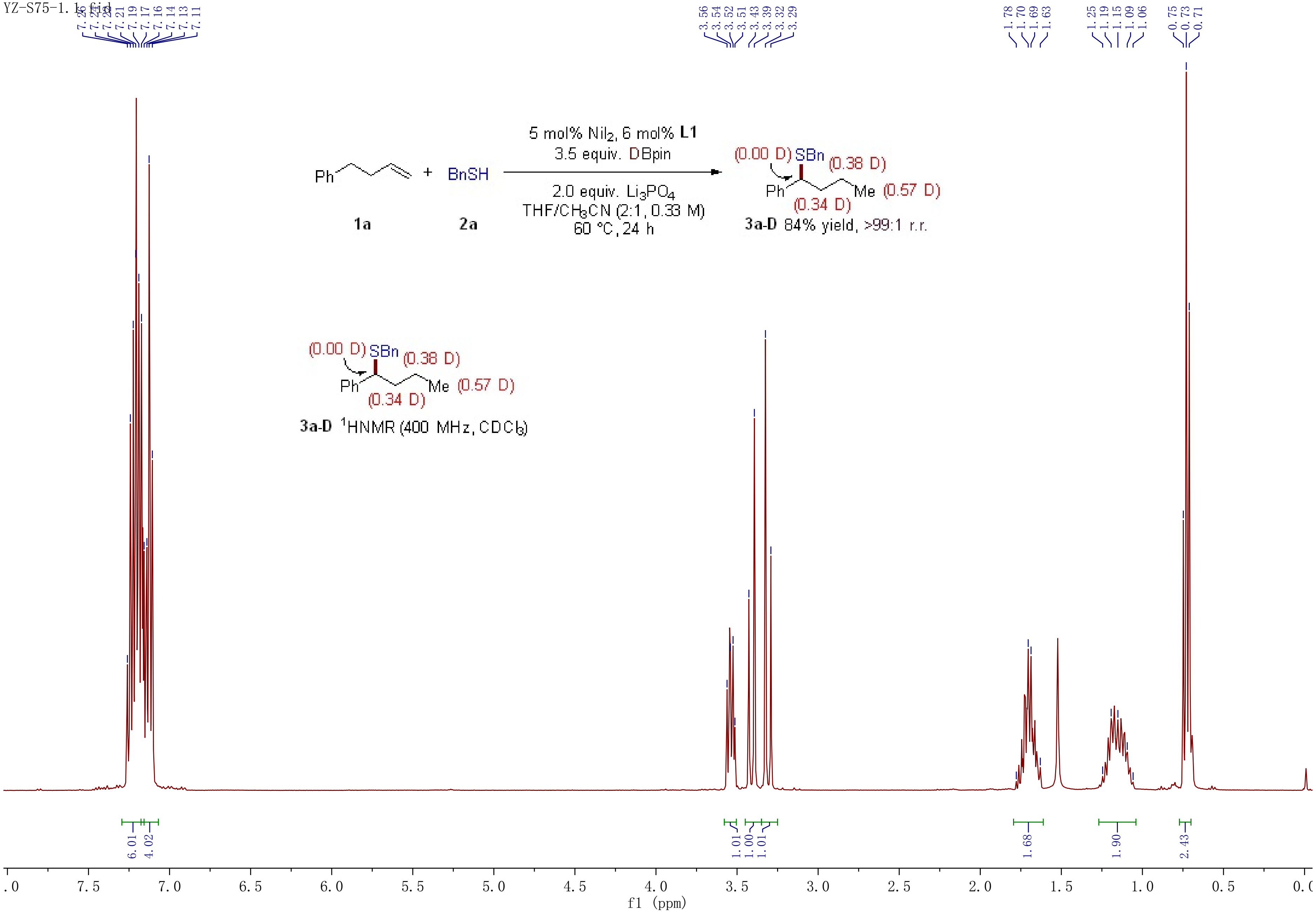

Supplementary Figure 246. ^{1}H NMR (400 MHz, $CDCl_3$) spectra for compound **3a-D**

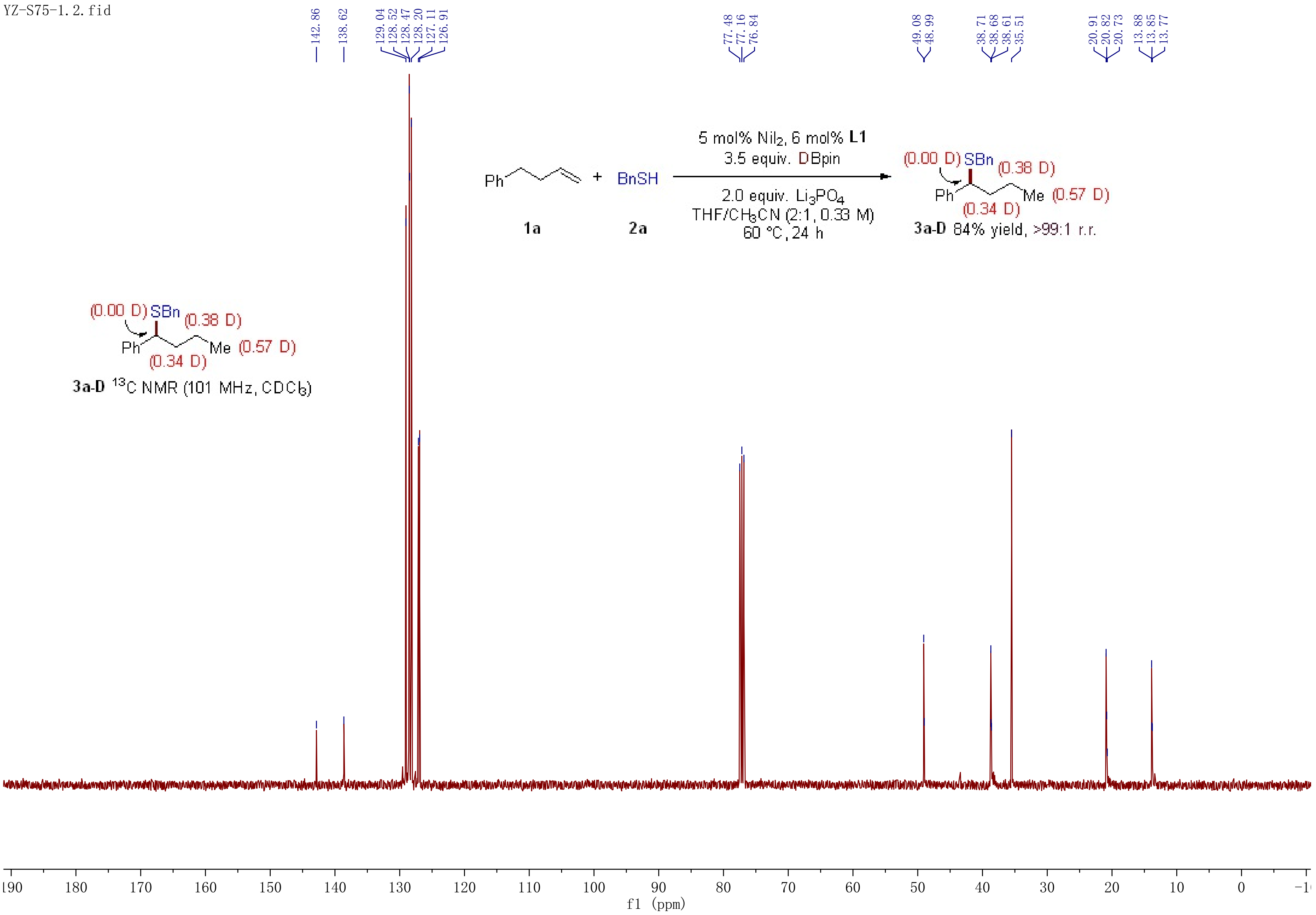

Supplementary Figure 247. ^{13}C NMR (101 MHz, CDCl$_3$) spectra for compound **3a-D**

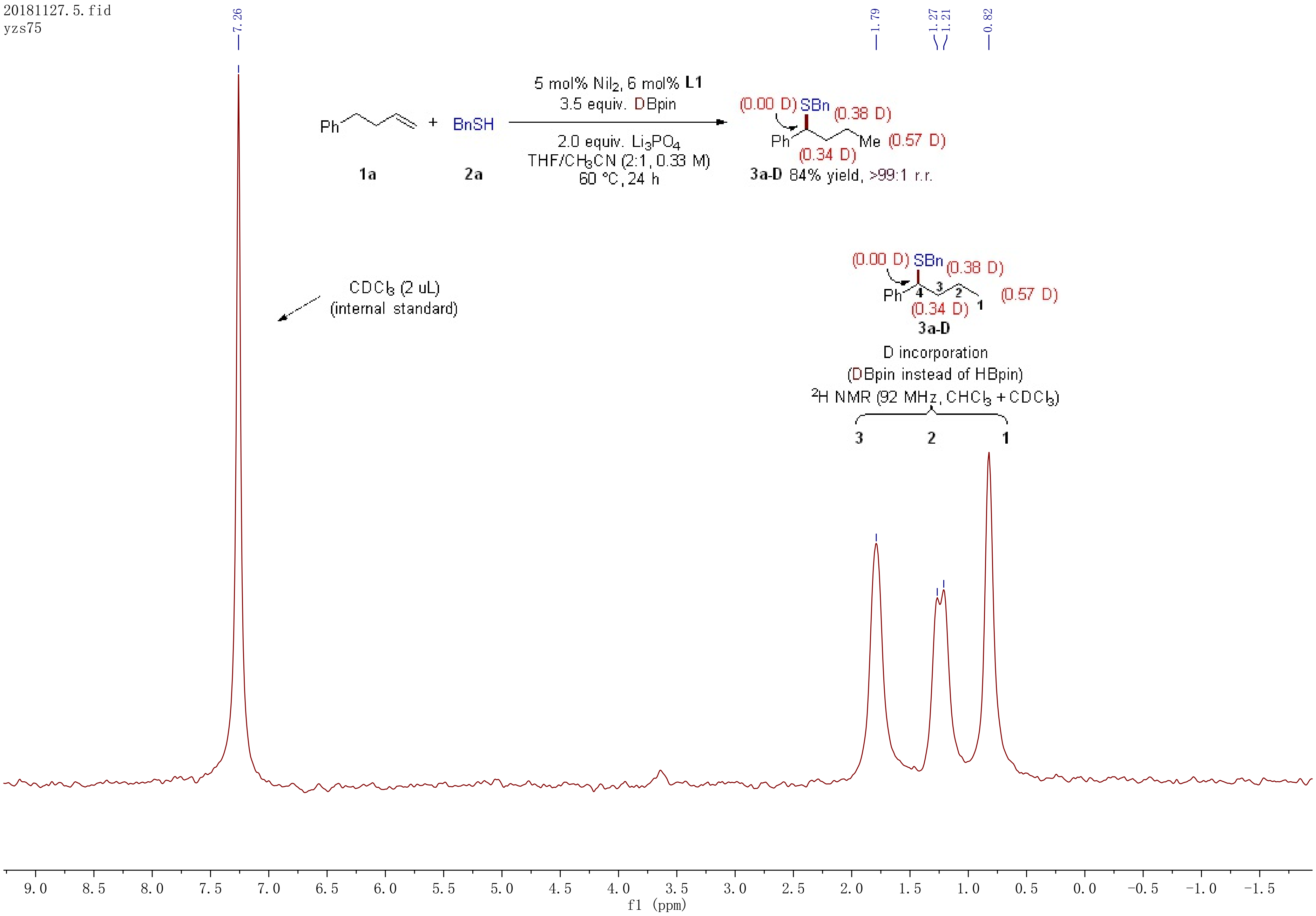

Supplementary Figure 248. ^{2}H NMR (92 MHz, $CHCl_3$+$CDCl_3$) spectra for compound **3a-D**

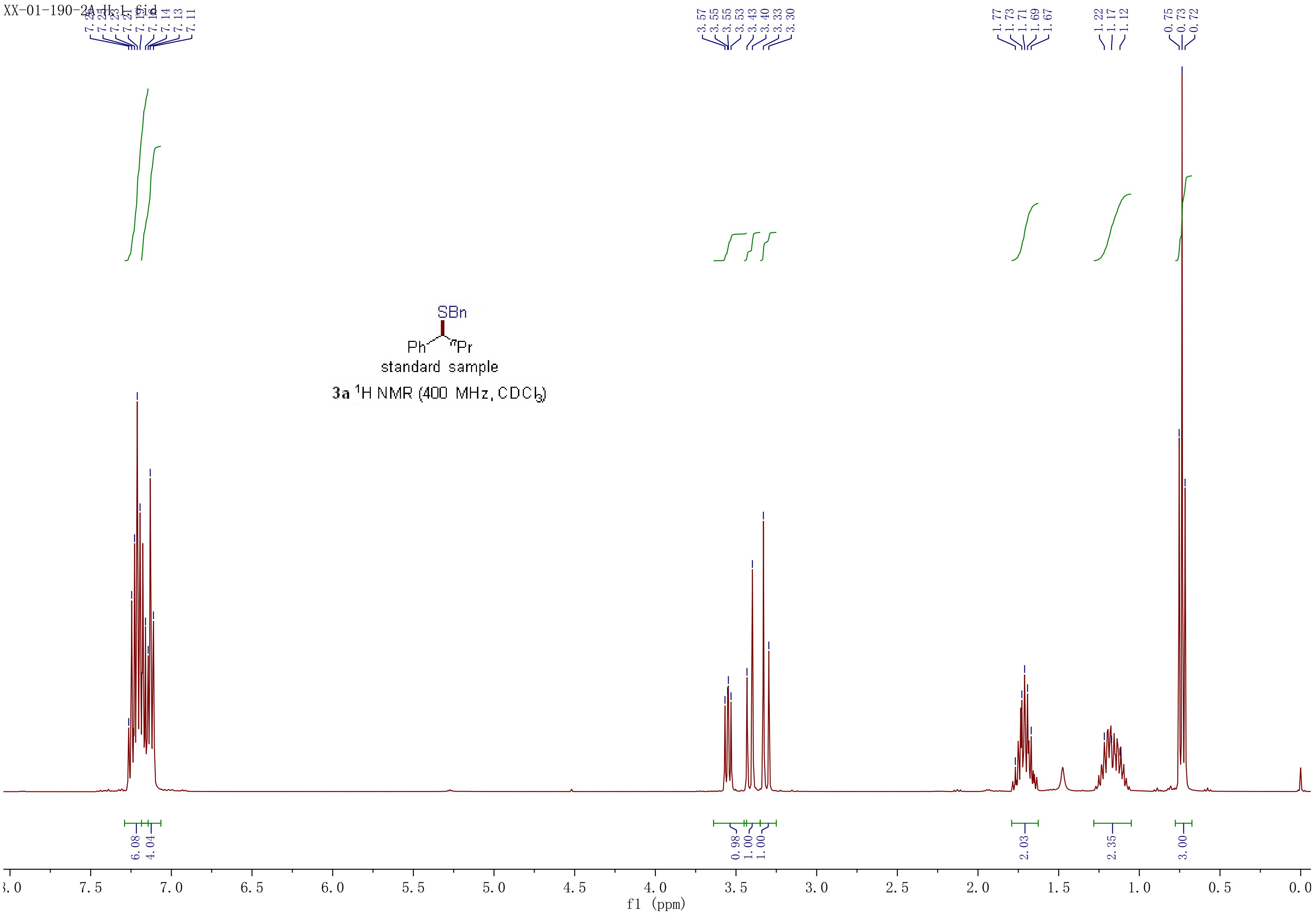

Supplementary Figure 249. ^{1}H NMR (400 MHz, $CDCl_3$) spectra for compound **3a**

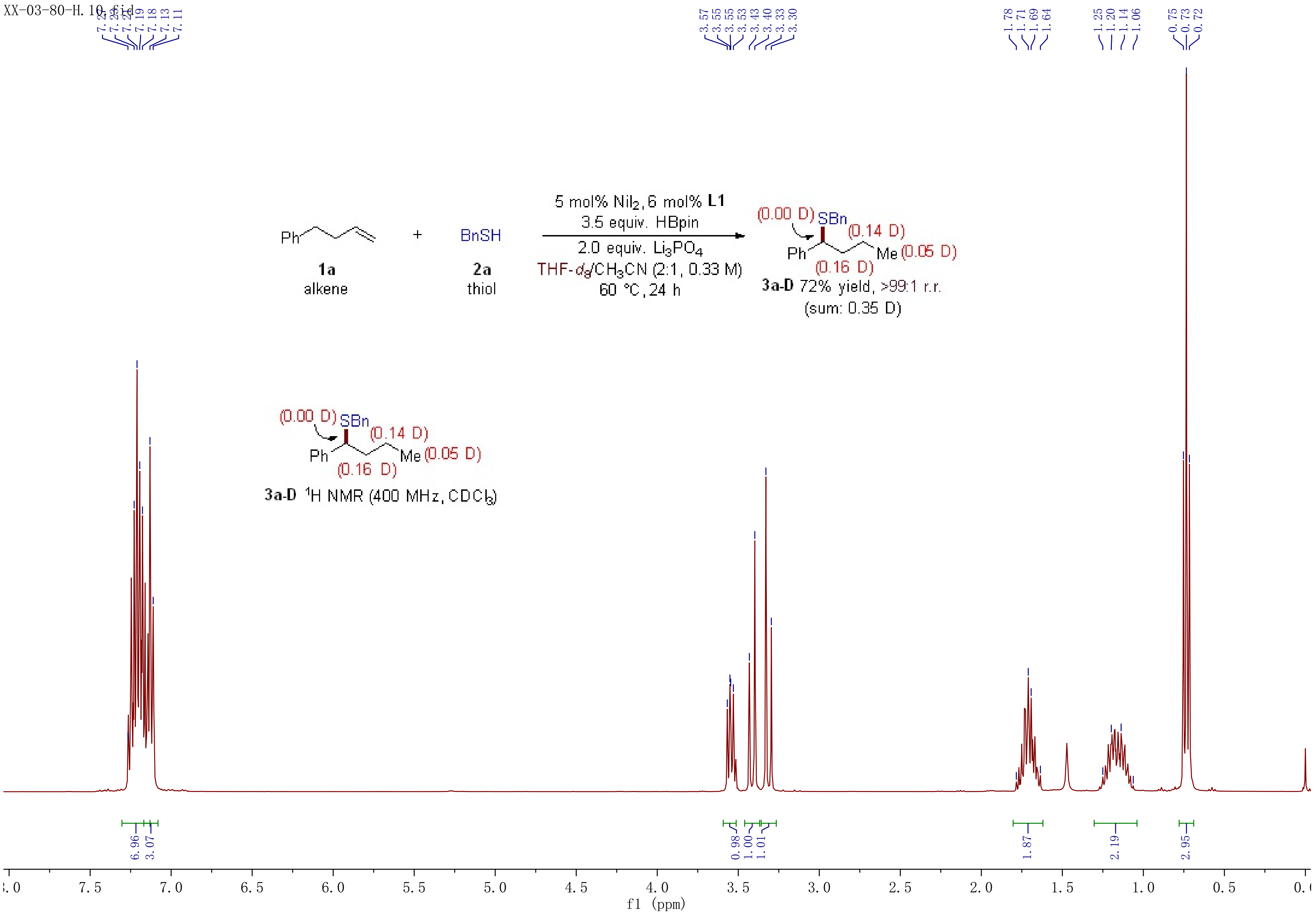

Supplementary Figure 250. ^{1}H NMR (400 MHz, $CDCl_3$) spectra for compound **3a-D**

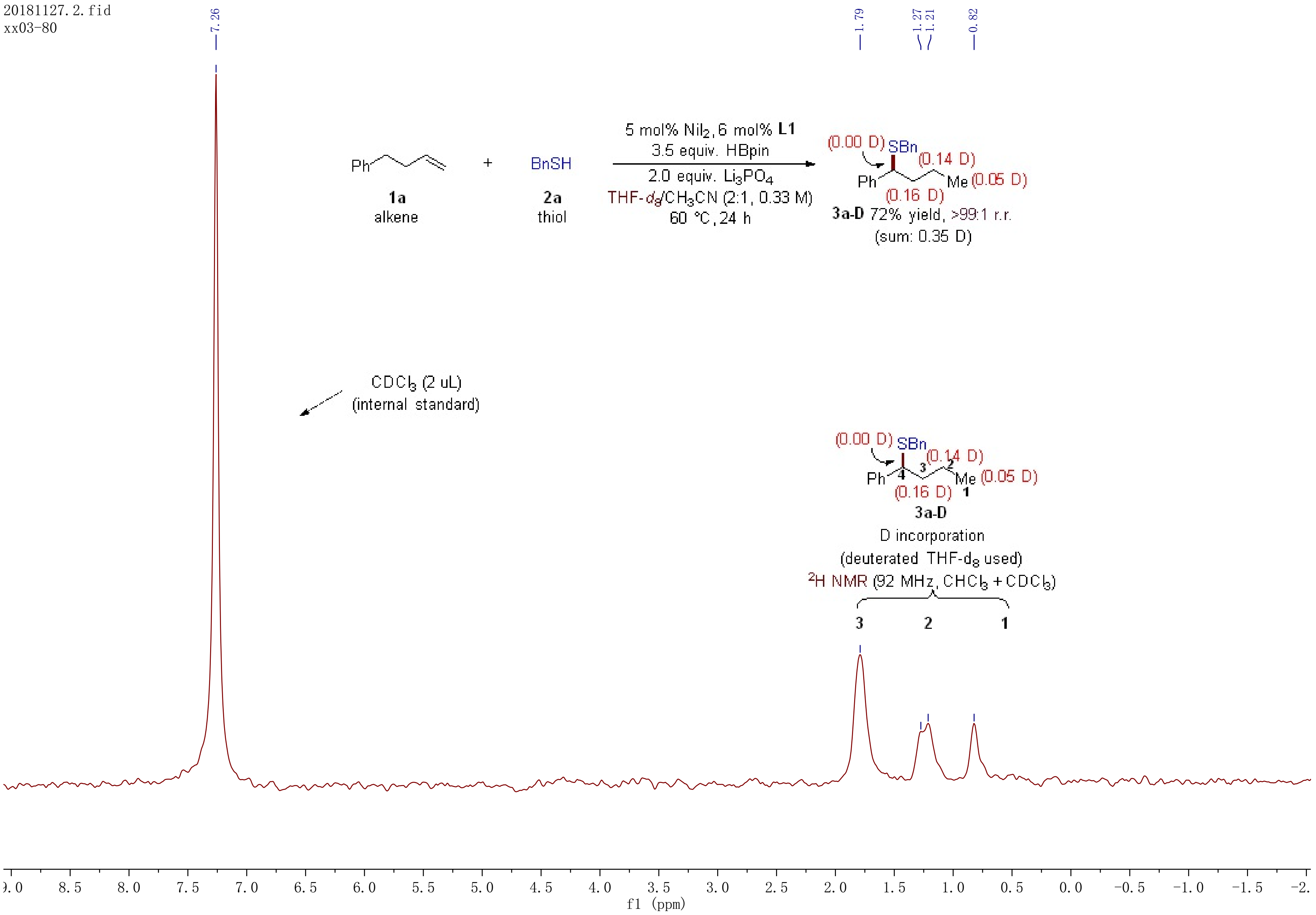

Supplementary Figure 251. ^{2}H NMR (92 MHz, $CHCl_3$+$CDCl_3$) spectra for compound **3a-D**

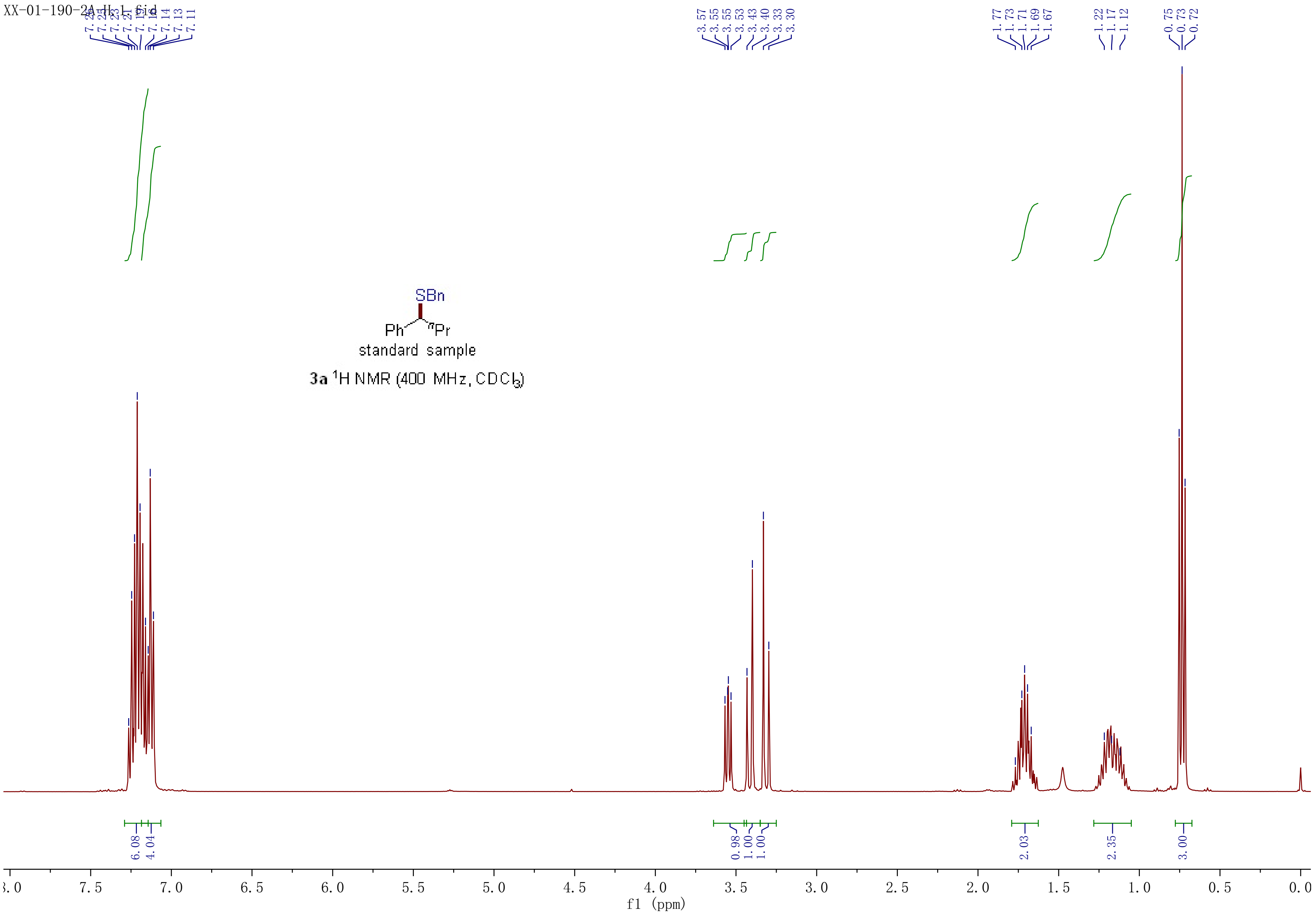

Supplementary Figure 252. ^{1}H NMR (400 MHz, $CDCl_3$) spectra for compound **3a**

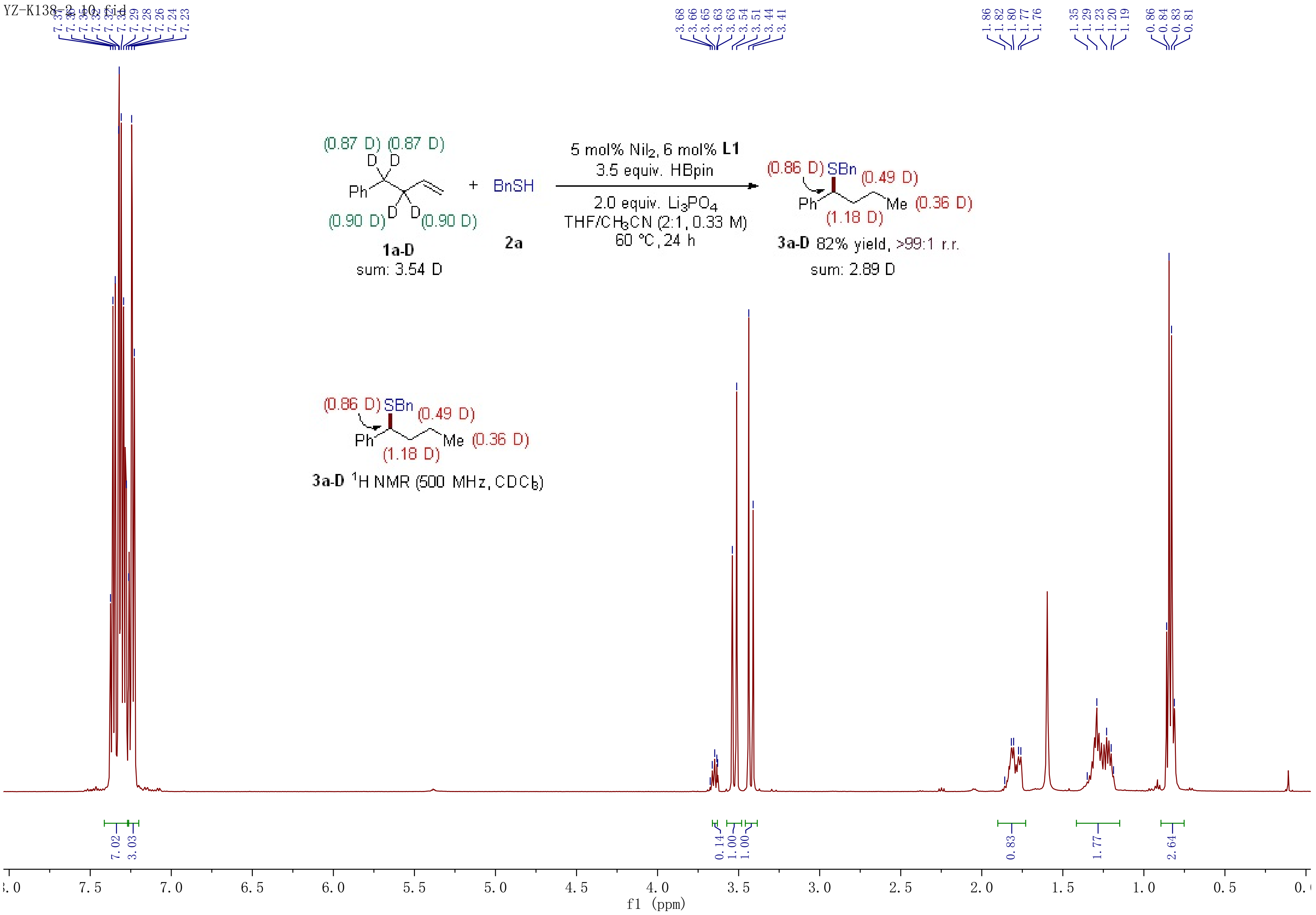

Supplementary Figure 253. ^{1}H NMR (500 MHz, $CDCl_3$) spectra for compound **3a-D**

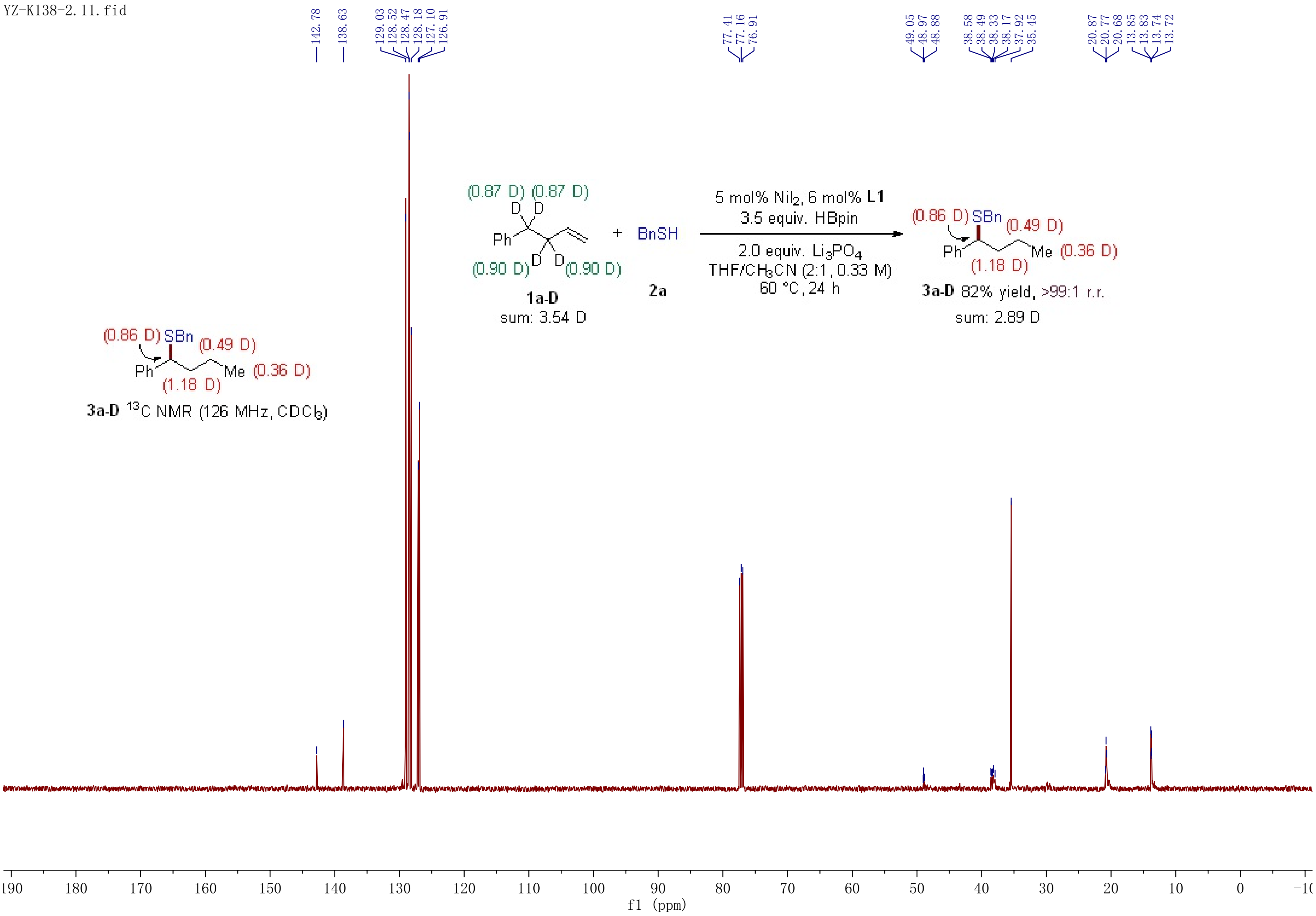

Supplementary Figure 254. ^{13}C NMR (126 MHz, CDCl$_3$) spectra for compound **3a-D**

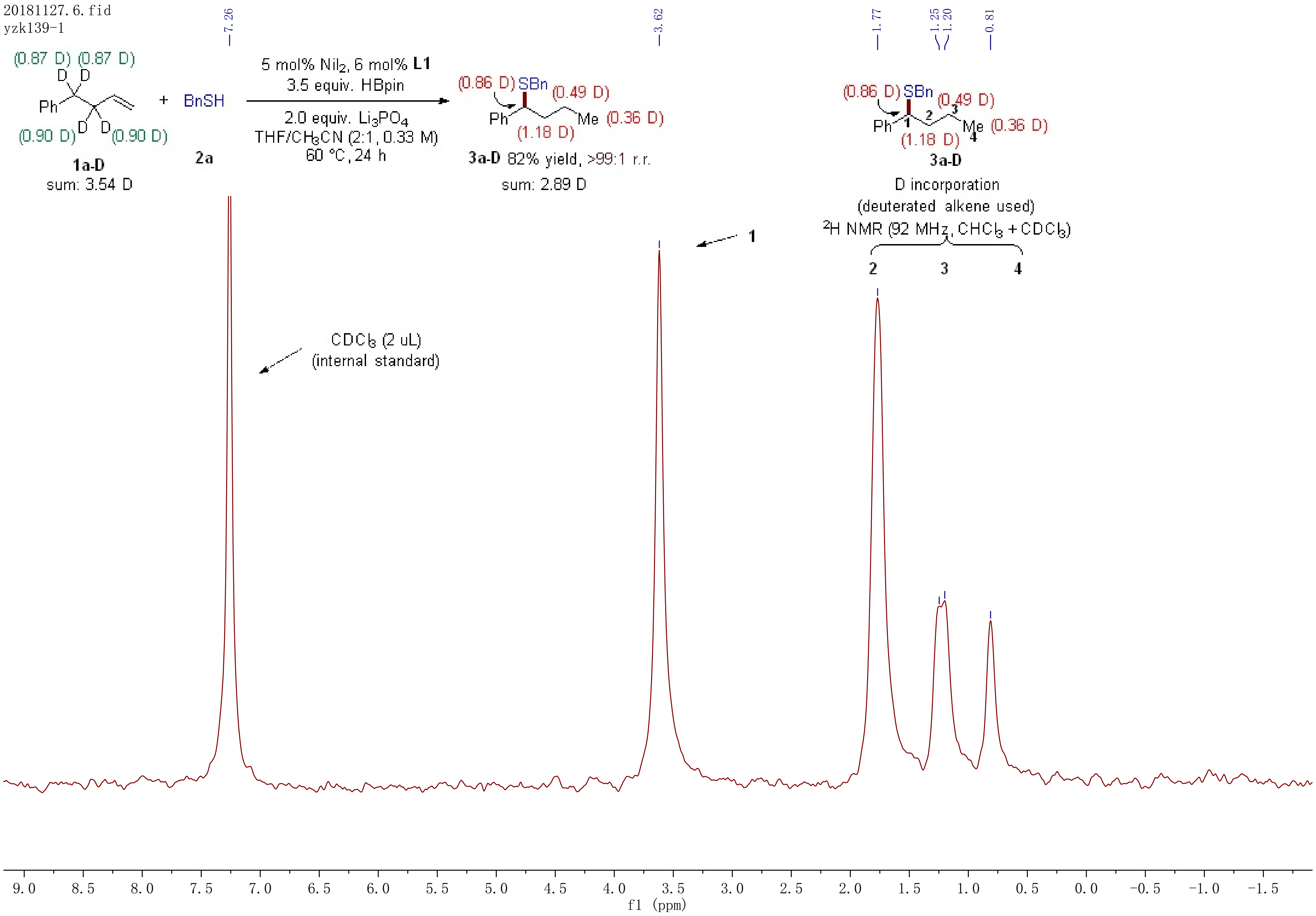

Supplementary Figure 255. ^{2}H NMR (92 MHz, $CHCl_3$+$CDCl_3$) spectra for compound **3a-D**

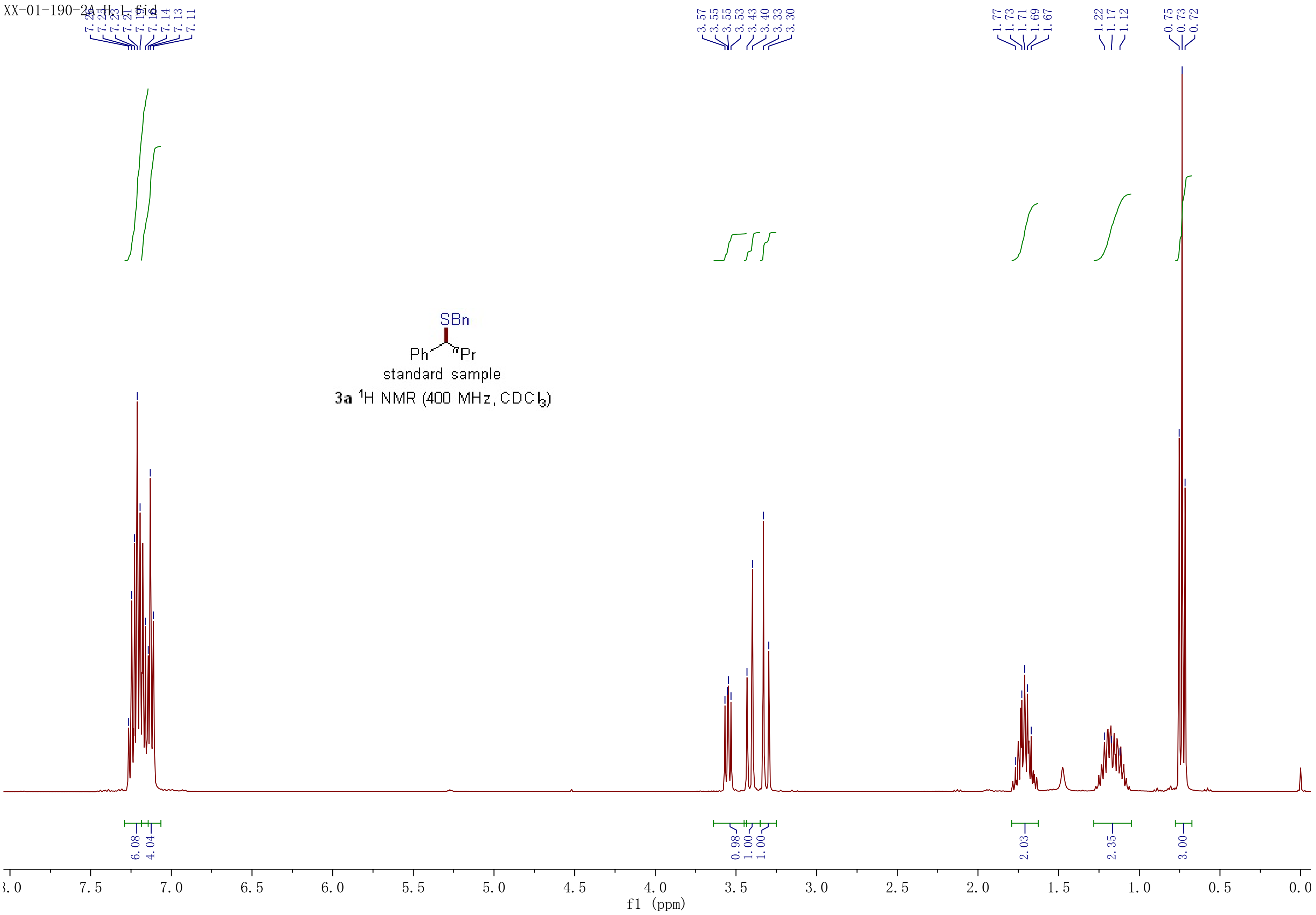

Supplementary Figure 256. ^{1}H NMR (400 MHz, $CDCl_3$) spectra for compound **3a**

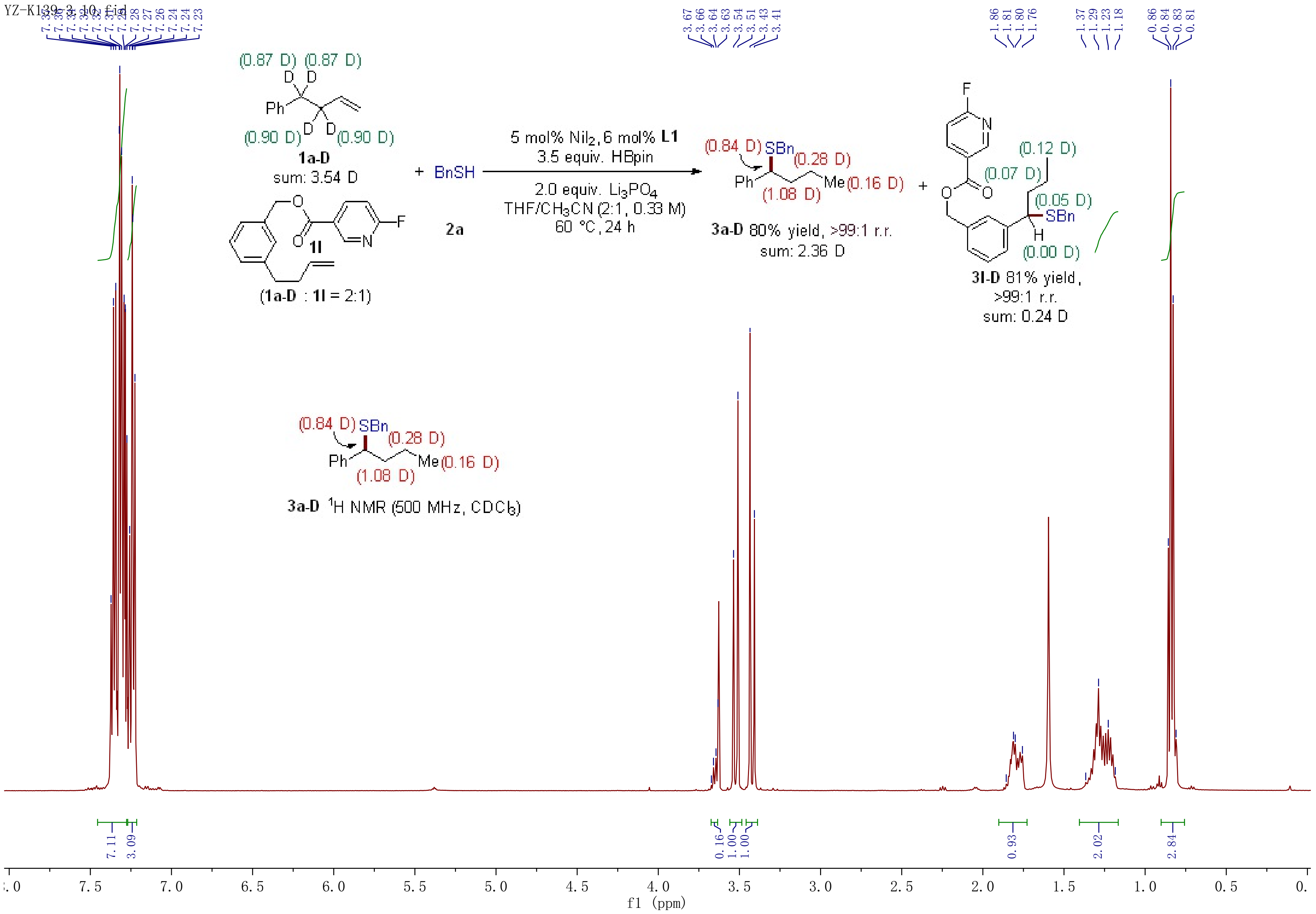

Supplementary Figure 257. ^{1}H NMR (500 MHz, $CDCl_3$) spectra for compound **3a-D**

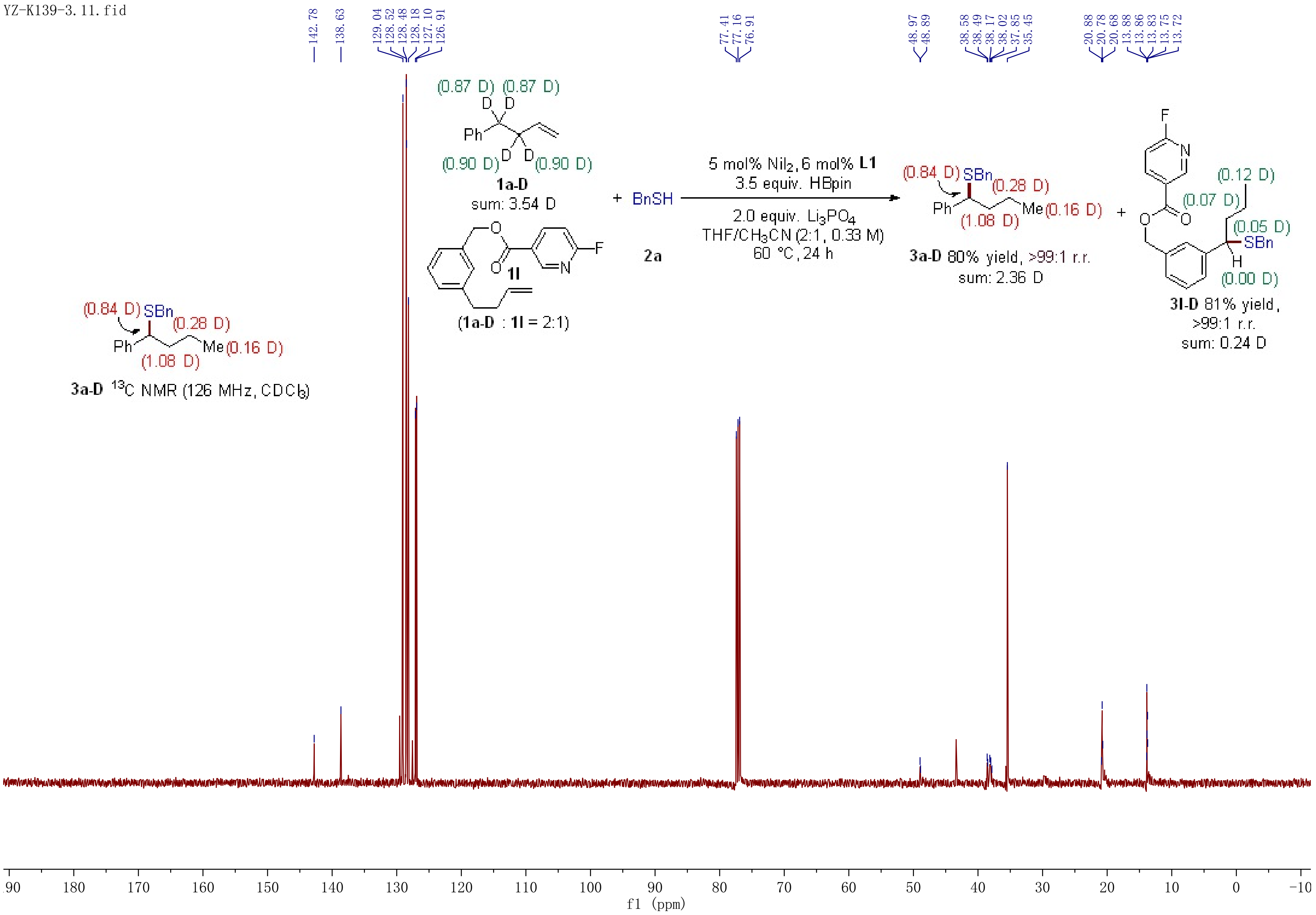

Supplementary Figure 258. ^{13}C NMR (126 MHz, CDCl$_3$) spectra for compound **3a-D**

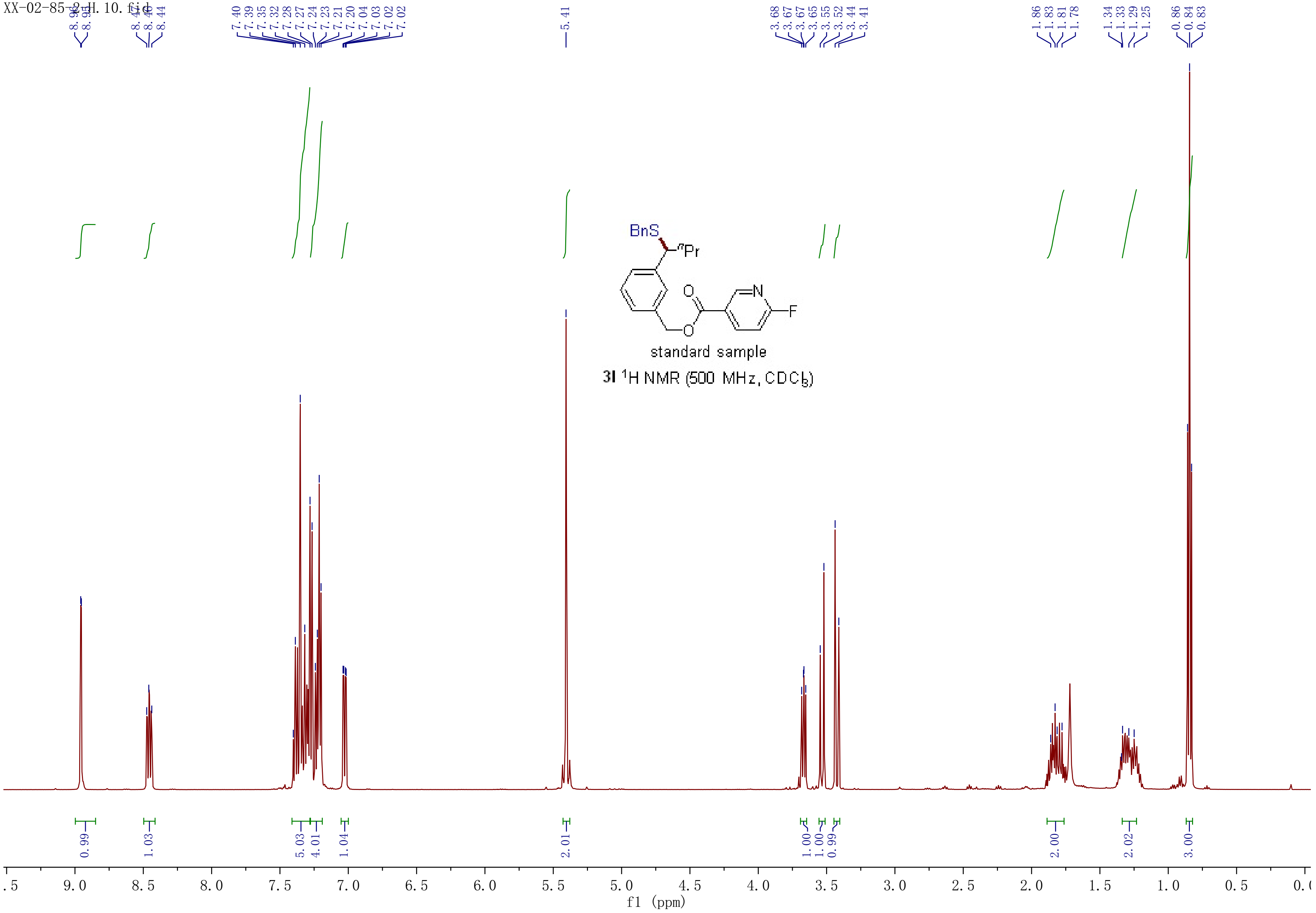

Supplementary Figure 259. ^{1}H NMR (500 MHz, $CDCl_3$) spectra for compound **3l**

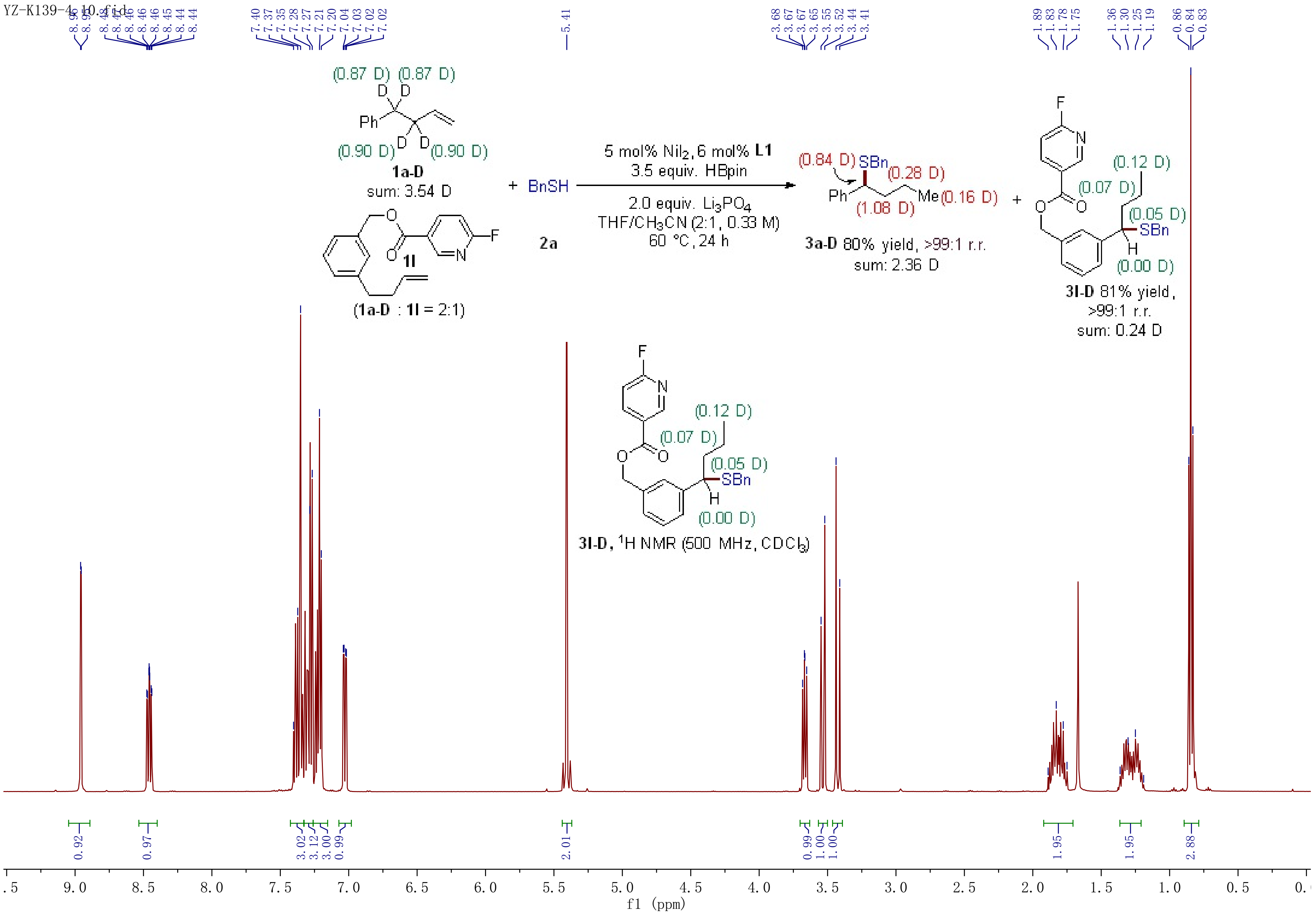

Supplementary Figure 260. ^{1}H NMR (500 MHz, $CDCl_3$) spectra for compound **3l-D**

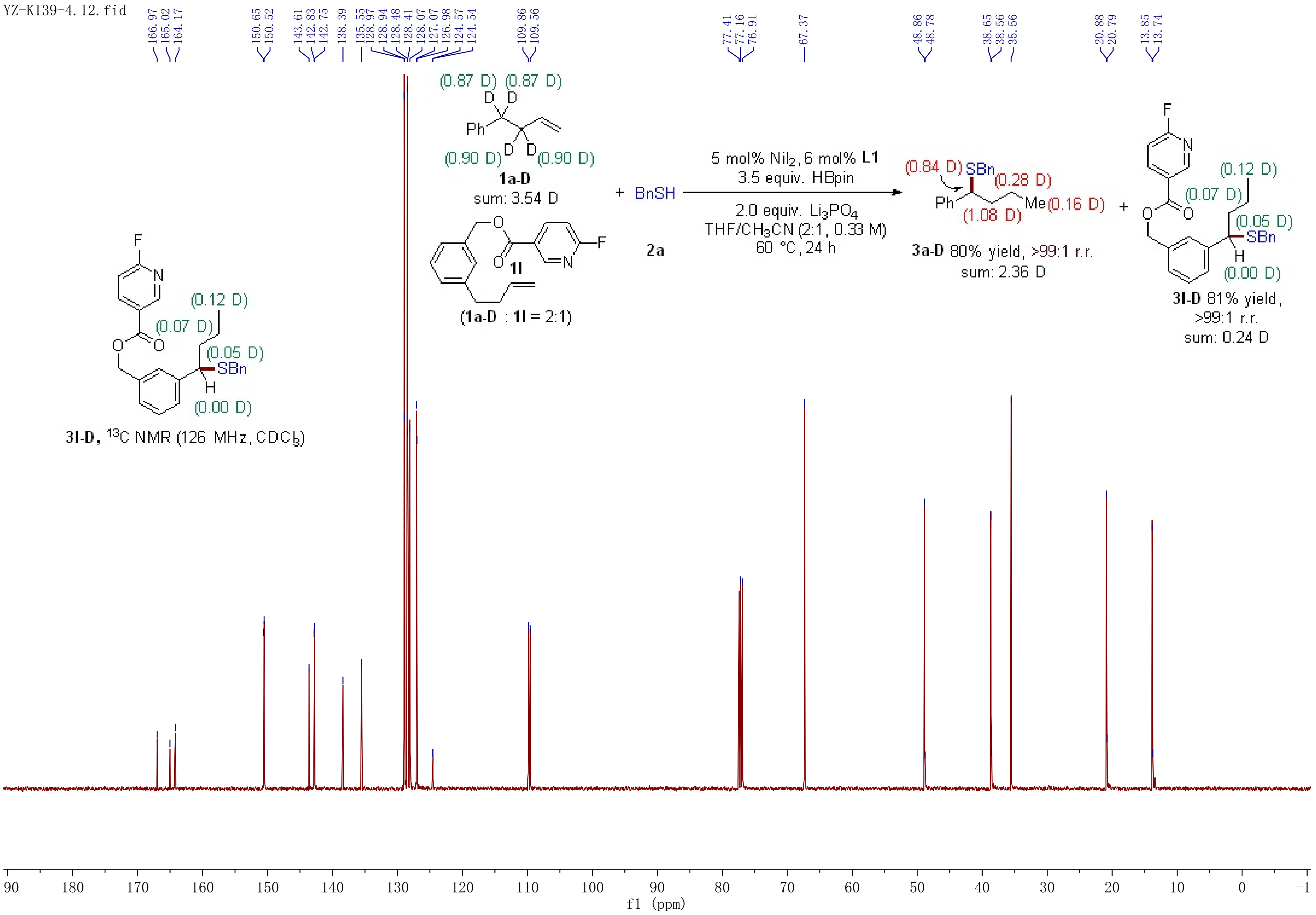

Supplementary Figure 261. ^{13}C NMR (126 MHz, $CDCl_3$) spectra for compound **3l-D**

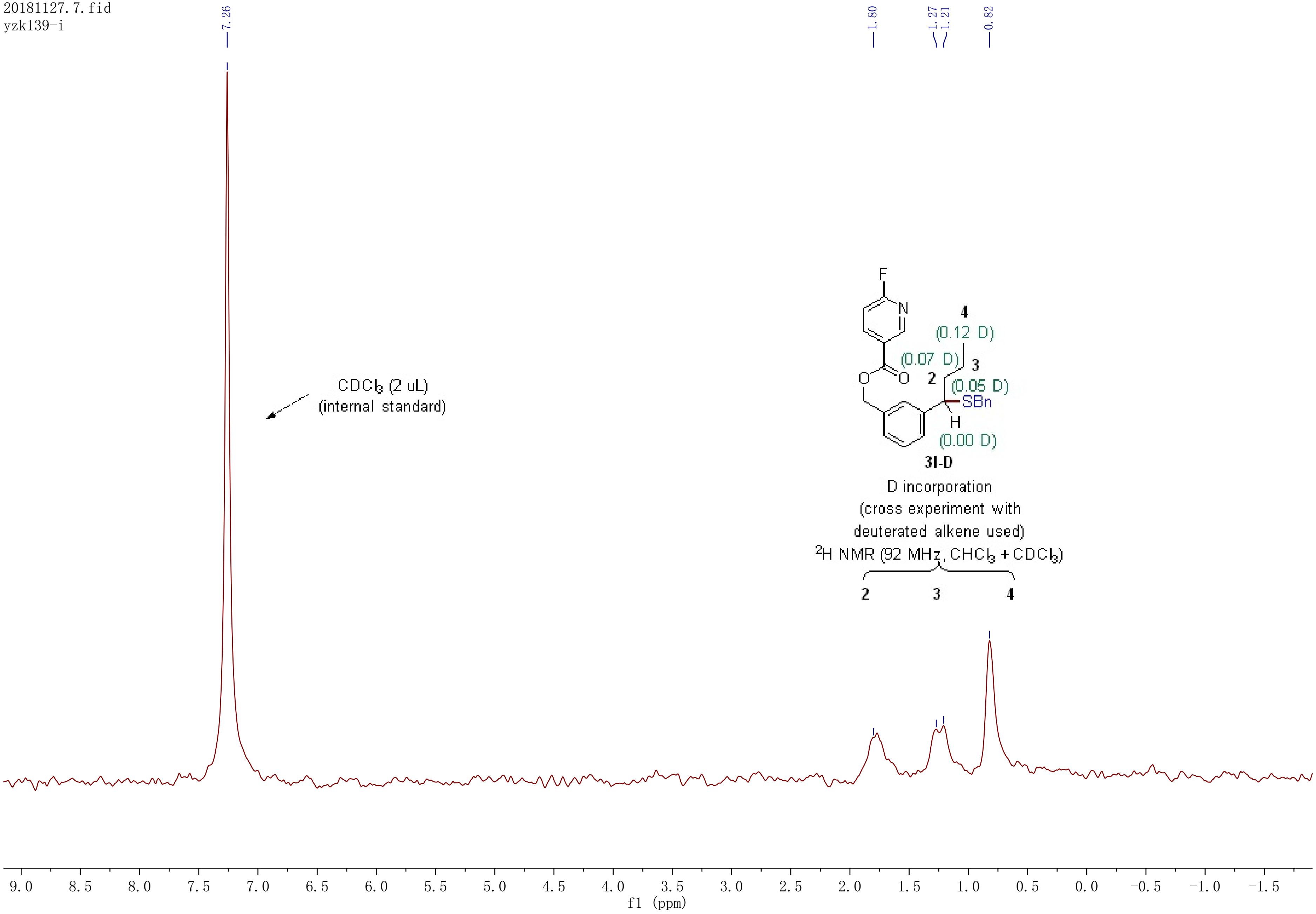

Supplementary Figure 262. ^{2}H NMR (92 MHz, $CHCl_3$+$CDCl_3$) spectra for compound **3l-D**

Supplementary References

1. Huang, J., Yan, W., Tan, C., Wu, W. & Jiang, H. Palladium-catalyzed regioselective hydroboration of aryl alkenes with B_2pin_2. *Chem*. *Commun*. **54**, 1770–1773 (2018).

2. Guo, J., Cheng, B., Shen, X. & Lu, Z. Cobalt-catalyzed asymmetric sequential hydroboration/hydrogenation of internal alkynes. *J*. *Am*. *Chem*. *Soc*. **139**, 15316–15319 (2017).

3. Civit, M. G., Sanz, X., Vogels, C. M., Webb, J. D., Geier, S. J., Decken, A., Bo, C., Westcott, S. A. & Fernández, E. Thioboration of α,β-unsaturated ketones and aldehydes toward the synthesis of β-sulfido carbonyl compounds. *J*. *Org*. *Chem*. **80**, 2148–2154 (2015).

4. Fernández-Salas, J. A., Manzini, S. & Nolan, S. P. Efficient ruthenium-catalysed S-S, S-Si and S-B bond forming reactions. *Chem*. *Commun*. **49**, 5829–5831 (2013).
